# Seismic performance evaluation of ancient Tibetan residential structures and energy dissipation of Mortise-Tenon Joints

**DOI:** 10.1371/journal.pone.0334654

**Published:** 2025-10-27

**Authors:** Jie Tang, Jingran Xu, Dewen Liu

**Affiliations:** 1 School of Engineering, Lishui University, Lishui, China; 2 Yantai Research Institute of Harbin Engineering University, Harbin, China; 3 Krirk University, Bangkok, Thailand; Universiti Teknologi Malaysia, MALAYSIA

## Abstract

Tibet, situated in western China, is renowned for its highland landscape, rich culture, and profound religious heritage. Tibetan-style ancient houses, typical dwellings in this region, reflect the Tibetan people’s adaptation to the plateau environment and hold significant cultural, historical, and ecological value. This study used theoretical and finite element analyses to compare the seismic performance of Tibetan houses under various conditions. It evaluated the effects of three types of seismic waves—with and without earth walls—having peak accelerations of 0.4 g and 0.6 g, focusing on interstory displacement angles and top accelerations. Finite element analyses were also conducted on through-tenon and straight-tenon joints to examine stress distributions and hysteresis curves for transverse and smooth grain orientations. Results showed that earth walls significantly enhanced the stiffness and seismic stability of the timber frame. Additionally, through-tenons exhibited fuller hysteresis curves than straight tenons, demonstrating superior energy dissipation.

## 1. Introduction

Tibet, nestled in the western part of China, is renowned for its distinctive plateau landscapes, vibrant ethnic culture, and profound religious heritage.Situated at the convergence of tectonic plates, the Tibetan Plateau endures recurrent seismic activities marked by low-frequency, high-intensity ground motions. Local rammed earth materials, shaped by the high-altitude environment, exhibit distinct mechanical behaviors. Notably, during the 2013 Lushan earthquake, traditional Tibetan rammed earth dwellings in Luding County demonstrated remarkable resilience, remaining structurally intact despite developing cracks—effectively dissipating seismic energy through micro-crack friction. Similarly, following the 1951 Chamdo earthquake, rammed earth structures sustained damage primarily in their upper sections without catastrophic collapse. Compellingly, data from the Tibet Seismological Bureau reveals that 72% of rammed earth houses in villages affected by magnitude 5 + earthquakes over the past five decades required only minor repairs. These observations underscore the unique seismic performance of rammed earth walls in the plateau, thereby justifying the urgent need for in-depth investigations into their mechanical characteristics.

**Fig 1:**
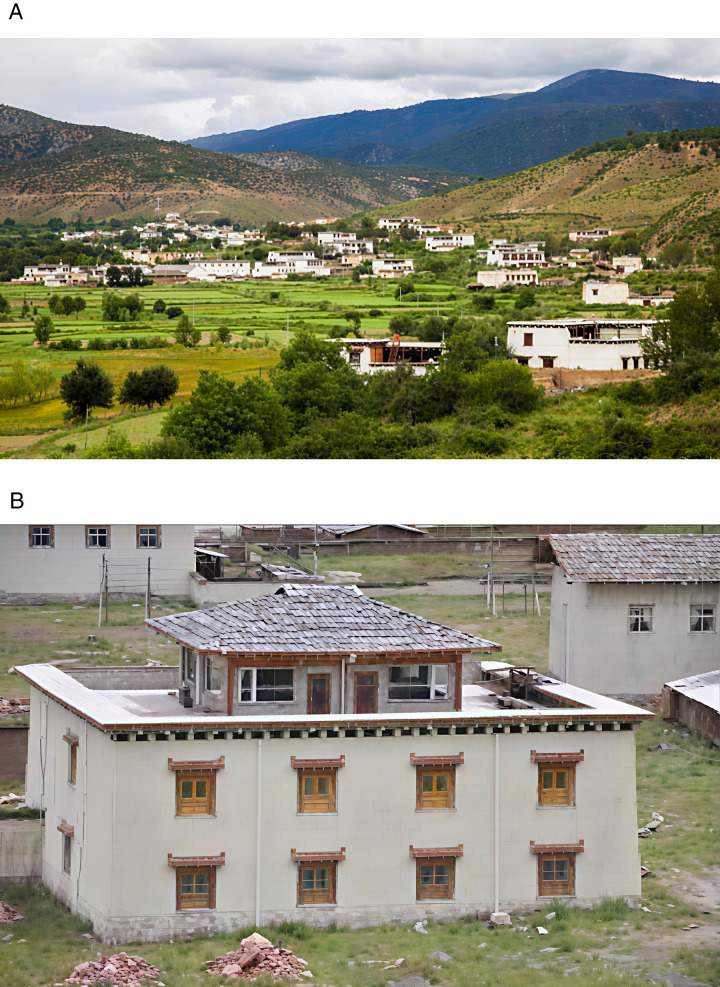
Exterior view of the Tibetan-style ancient houses in the Tibetan area. (a) Map of villages in Tibetan areas as a whole (b) Tibetan-style ancient house.

**Fig 2:**
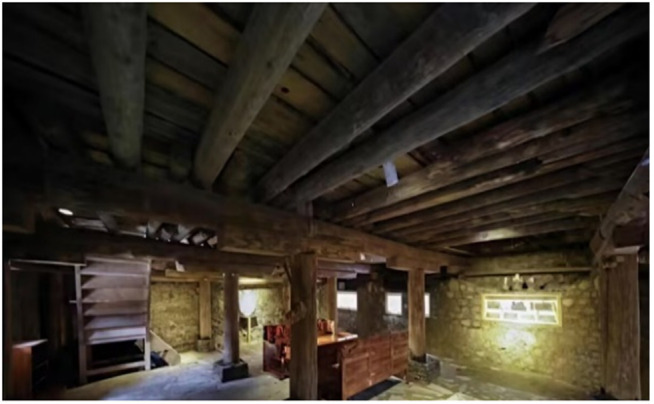
Framing plan of the interior wood structure of Tibetan-style ancient house.

On this enchanting land, Tibetan – style ancient houses, as exemplary representatives of traditional dwellings, vividly showcase the remarkable wisdom of the Tibetan people in harmoniously co – existing with the natural environment. The unique architectural style of these ancient Tibetan – style houses is a seamless integration of the intricate internal wooden structure and the sturdy external earth wall. The internal wooden structure features beams and pillars that penetrate the walls. The pillars stand firmly atop the ground – floor lifting beams, jointly constructing a highly efficient wooden – frame load – bearing system. This time – honed structural design has endued ancient Tibetan houses with remarkable seismic resilience in the earthquake – prone plateau regions. It is a vivid testament to the profound comprehension of mechanical principles and the masterful craftsmanship of ancient Tibetan artisans. The external earth walls of these houses are equally remarkable. Typically constructed with stones and coated with yellow mud or other protective substances, these walls not only safeguard the structure effectively but also impart an antique charm to the building. The outer walls gradually taper upwards, forming a characteristic trapezoidal structure. This design not only offers high aesthetic value but also significantly contributes to reducing the building’s weight and lowering its center of gravity, thereby enhancing the overall stability of the ancient Tibetan – style houses. Traditional ancient Tibetan – style houses are generally two – or three – story structures, boasting compact and functional internal layouts complete with courtyards and wells. These features meet the diverse daily needs of the residents, as depicted in Fig 1(a), (b) and Fig 2. Nonetheless, due to historical constraints, the construction of most ancient Tibetan – style houses relied heavily on the experiences of predecessors and the personal concepts of builders, lacking standardized guidelines. This has inevitably imposed substantial challenges on the current conservation and restoration efforts of these ancient Tibetan houses. In earthquake – prone areas, the seismic performance of these ancient houses is crucial for the preservation and inheritance of these structures, which are repositories of rich historical and cultural values. Consequently, delving deep into the various types of mortise – and – tenon joints and considering the influence of earth walls on the seismic performance of ancient Tibetan houses is of utmost practical importance for the continuation and safeguarding of this ancient architectural heritage.

In recent years, as the focus on the preservation and restoration of ancient architecture has intensified, the enhancement of the seismic resilience of wooden – structured buildings has emerged as a prominent area of research. This pursuit holds profound significance for the conservation and renovation of ancient Tibetan dwellings. A wealth of research findings has furnished us with invaluable insights and references. Maria Adelaide Parisi [1] proposed different criteria for seismic reinforcement of floor slabs and carpentry joints. Ashkan Hashemi [2] and others used metal fasteners such as nails, rivets, screws, or bolts to make traditional light wood frame buildings usually with traditional plywood sheathing Shear walls are used as the main lateral load resisting members. During earthquakes, these structures are prone to non-negligible plasticity deformations in the fasteners, which can lead to drastic stiffness degradation. The development of a novel lateral load resisting system, which consists of swinging wooden walls and elastic sliding friction (RSF) joints as fixed connections, is presented in detail. Verification of the seismic performance of the system is achieved through nonlinear cycling and dynamic simulations. In order to evaluate the efficiency of the proposed concept at the system level, a five-story prototype building was designed based on the Displacement Based Design (DBD) program. The results confirm the good seismic performance of the structure in terms of ductility, energy dissipation and self-centering behavior. David Ugalde [3] and others in a review of seismic techniques for wood structures state that wood structures have a variety of various properties that are well known and that traditional wood structures have very superior seismic performance. Faggiano B [4] and others, in view of the development of heavy seismic timber structures, in the context of modern seismic design methods, allowing wood structures to dissipate a portion of the seismic energy has become an effective solution. Since wood is an elastic and brittle material, the energy dissipation function should be transferred to the connectors through plastic deformation of the steel connectors. However, the joints are the main structural elements and play an important role in carrying the design loads. Yang Chun [5] and others studied the seismic performance of mortise and tenon joints of wooden structures of Southeast Guizhou Province residential houses through low-week reciprocating loading experiments, and found that the joints of the wooden structures can, to a certain extent, play the strength and ductility of the wood itself. Shen Yinlan [6] et al. investigated the damage mode, loading stiffness, deformation, bending moment angle hysteresis response, skeleton curve, strength and energy dissipation capacity and other mechanical properties of the traditional wood structure through the proposed static low-week reciprocating loading test, and found that the damage to tenon joints manifested in the form of mortise and tenon pull-out, mortise and tenon and mortise extruded and deformed; compared with the intact joints, the bending capacity, loading stiffness, and energy dissipation capacity of the defective steamed bun tenon joints were significantly reduced. Qiang Mingli [7] and others conducted a study on Tibetan sloping roof house dwellings, focusing on the structure of the column frame layer, and found that the structural connection method is mainly mortise and tenon and flat pendulum floating shelves, which has a certain seismic damping effect and good structural ductility.

Currently, numerous scholars’ research on brick-and-timber structure has borne abundant fruits [8–15]. Wood-frame construction is experiencing a thriving development trend both globally and within China. With the continuous advancement of technology and robust policy support, it is quite foreseeable that wood – frame buildings will assume an even more crucial role in the construction industry in the future [16–20]. However, when it comes to the research on ancient Tibetan dwellings, the majority of studies predominantly concentrate on aspects such as their structural forms and architectural art. There is a conspicuous shortage of in – depth exploration into their structural performance. This situation has presented certain challenges to the conservation and upkeep of ancient Tibetan houses. In light of this, this paper delves into the joint theory of wood structures and contrasts it with the post – processing results of ABAQUS finite element analysis, aiming to offer more targeted approaches for the conservation and maintenance of ancient Tibetan dwellings. Specifically, three distinct types of seismic waveforms are utilized to conduct time – history analyses of ancient Tibetan dwellings, considering both the scenarios with and without the influence of earth walls, under accelerations of 0.4g and 0.6g. Additionally, static analyses are performed on through – tenon and straight – tenon joints (Through-tenon joints are structural connections where the tenon penetrates through the mortise and extends beyond it, while straight-tenon joints (also known as blind-tenon joints) typically refer to connections where the tenon meets the mortise perpendicularly without penetrating through to the other side.), and the relevant data are systematically processed. The findings of this research are anticipated to provide a vital theoretical foundation for the conservation, maintenance, and seismic performance assessment of ancient Tibetan dwellings. Subsequently, they will offer practical reference value for actual conservation and maintenance efforts, thereby facilitating the better inheritance and continuation of ancient Tibetan dwellings in the new era.

## 2. Materials and construction

### 2.1. Physical and mechanical properties of wood

**Fig 3:**
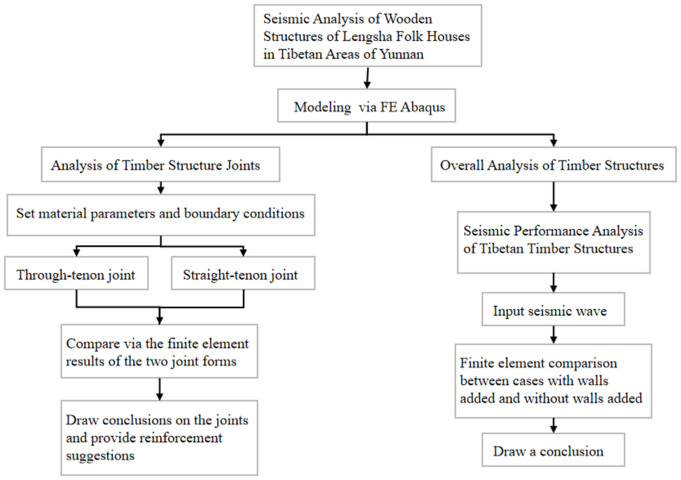
Technology road map.

Wood is an anisotropic biomass material, and its strength varies in different directions [21–24]. Based on existing literature and practical considerations, fir wood presents distinct advantages as a building material due to its availability and suitability. This study focuses on fir as the primary material of interest. Table 1, derived from the appendix on the principles of wood structures [25], details the physical and mechanical properties of fir. Fig 3 shows the technical roadmap.

**Table 1:** Physical and mechanical properties of fir.

Tree species	Compressive strength of smooth grain (N/mm^2^)	Bending strength (MPa)	Flexural modulus of elasticity (1000N/mm^2^)	Parallel shear strength (N/mm^2^)	Cross grain compressive strength (N/mm^2^)					Parallel grain tensile strength (N/mm^2^)
					Topical			Full		
				Diameter	Chord surface	Diameter	Chord surface	Diameter	Chord surface	
Fir	39.7	81.2	10.7	4.9	5.7	4.1	3.7	3.2	3.1	103.3

### 2.2. Constitutive relation and failure characteristics of fir wood

Fir, as the primary material of the wooden structure in Tibetan-style ancient houses, exhibits distinct anisotropic mechanical behavior, which is characterized by a constitutive model that integrates elastic and plastic phases. In the elastic stage, its mechanical properties are defined by engineering constants corresponding to three directions: longitudinal (parallel to the grain), radial, and tangential (perpendicular to the grain). Specifically, the compressive strength parallel to the grain is 39.7 N/mm², the bending strength is 81.2 N/mm², and the parallel grain tensile strength reaches 103.3 N/mm², while the transverse mechanical properties (e.g., radial and tangential compressive strengths ranging from 3.1 to 4.1 N/mm²) are significantly lower. In finite element simulations, the elastic behavior is described by assigning these directional parameters, and the plastic phase is characterized by specifying yield stress ratios for each direction, reflecting the material’s heterogeneous response under load.

The failure characteristics of fir are closely related to its grain orientation. Under transverse loading, the material is prone to cracking due to low tensile strength, making it vulnerable to failure at stress concentrations, such as the edges of beam-column interfaces. For mortise-tenon joints, failure criteria include excessive slip (observed in straight tenons under cyclic loading) and stress exceeding the transverse yield limit, which leads to deformation or detachment of the mortise and tenon. In the elastic-plastic stage, the stress distribution becomes trapezoidal when the transverse compressive stress reaches the yield strength (σ_C,R_), and the tangent modulus (E₂ = 0.38E_RC_) is used to describe the post-yield behavior, ensuring accurate simulation of energy dissipation and stiffness degradation.

### 2.3. Construction conditions, model of Tibetan-style ancient house and joint

#### 2.3.1. Construction features.

**Fig 4:**
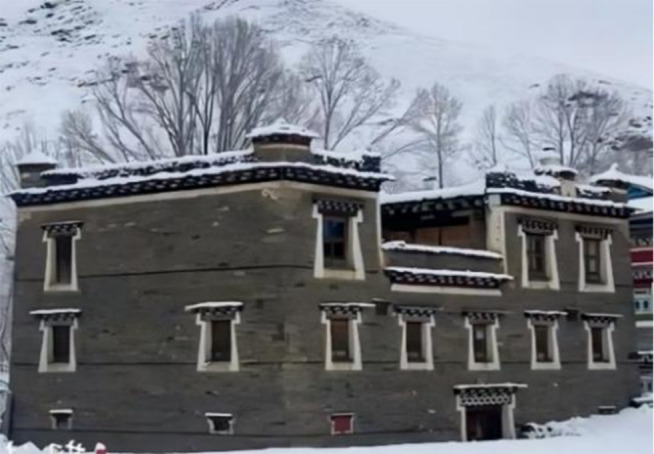
Tibetan-style ancient house building.

**Fig 5:**
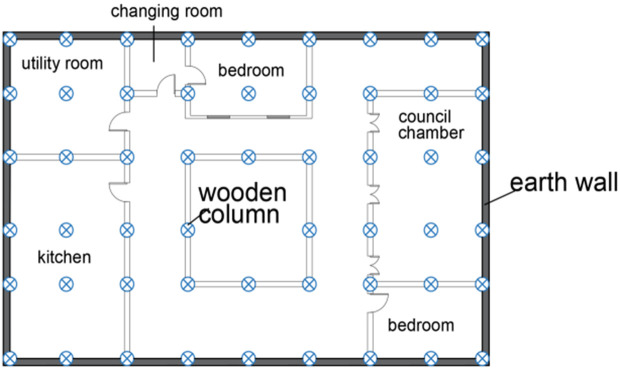
Schematic floor plan.

The structure of the building is divided into four vertical components: the first floor column-frame, the second floor column-frame, the third floor column-frame, and the roof. The plan dimensions of the Tibetan dwelling are 21,600 mm by 14,000 mm, with a total height of 12,900 mm. The column arrangement consists of a 9 × 6 configuration on the first floor and a 9 × 7 configuration on both the second and third floors, typically maintaining a one-to-one correspondence between the columns of adjacent layers. The columns are circular in cross-section, with a diameter of 300 mm. The gable columns on the first and second floors of the patio also have a diameter of 300 mm. Architectural drawings are presented in Fig 4 and Fig 5.

The wooden structure of the Tibetan dwelling is designed to withstand a seismic intensity of 8 degrees, categorized under the second group for seismic design, with the site classified as category two.According to Article 3.2.4 of the Code for Seismic Design of Buildings GB/T 50011–2010 (2024 Edition) [26], the seismic fortification intensity, design basic seismic acceleration value, and design seismic group for the central areas of major towns (county-level and above towns) in China are specified. In Tibet Autonomous Region, areas such as Pulan, Nielamu, and Saga have a seismic fortification intensity of Degree 8, a design basic seismic acceleration of 0.2g, and belong to the second design seismic group. The site category being Class II is determined in accordance with the relevant provisions of the General Code for Seismic Design of Buildings and Municipal Engineering GB 55002−2021 [27].

#### 2.3.2. Model of Tibetan-style ancient house and joint.

**Fig 6:**
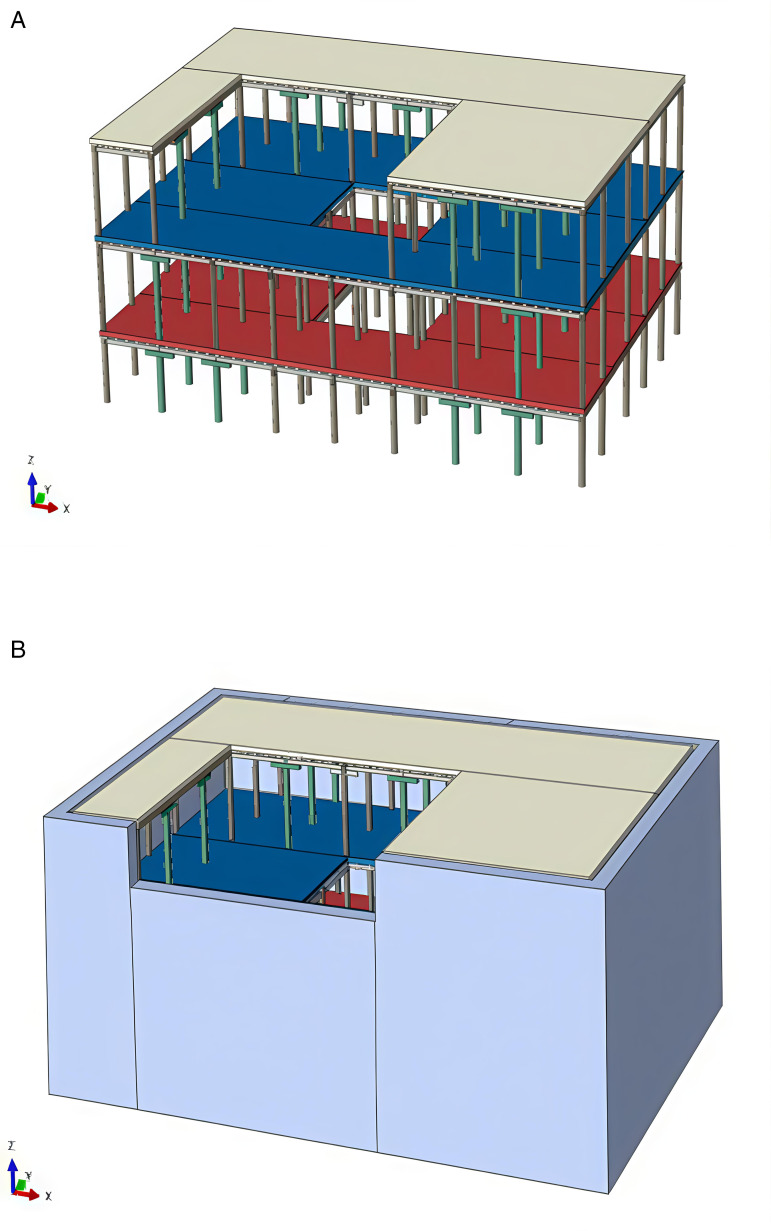
Schematic diagram of the model. (a) Structures that do not take into account the action of earth wall (b) Structures taking into account the role of earth wall.

Simplifications of the structural model can be made within reasonable limits. Wooden beams and columns are connected using various joint types, including beam-column lap joints, mortise and tenon joints, and right-angle mortise and tenon joints. This approach enhances the accuracy of the model through appropriate mesh division. Solid elements were employed in the finite element analysis to accurately capture the three-dimensional stress distribution and complex deformation characteristics of wooden structures and earth wall components, particularly at the mortise-tenon joints where significant stress concentrations occur. The schematic diagram of the model, illustrating both scenarios—with and without the consideration of the earth wall—is presented in Fig 6 (a) and (b).

**Fig 7:**
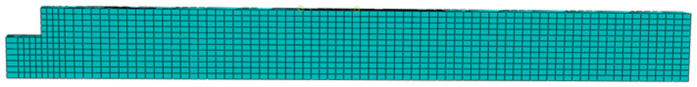
Network division of beams.

**Fig 8:**
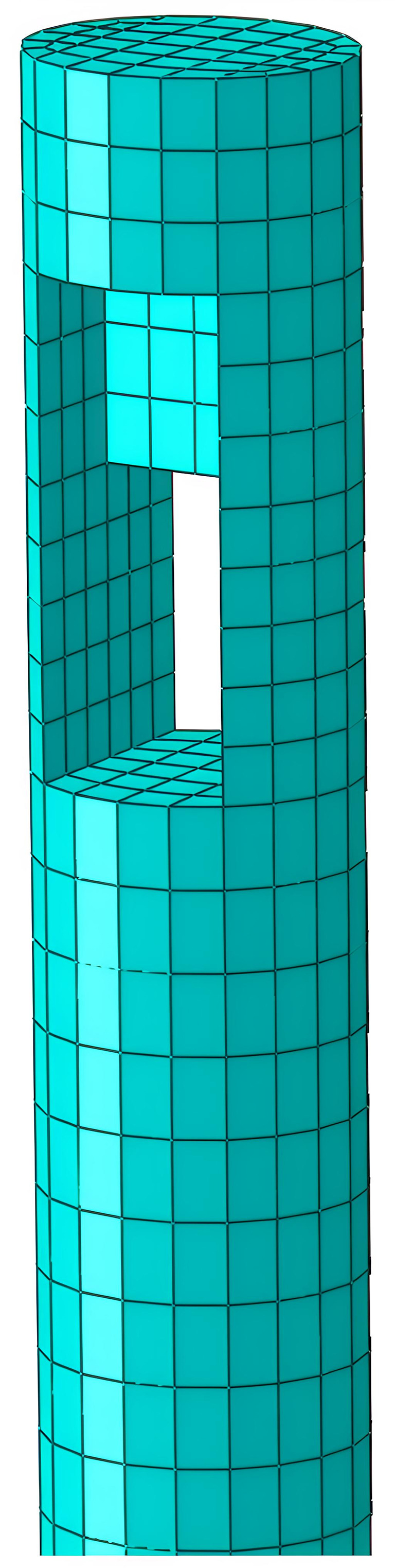
Network division of columns.

**Fig 9:**
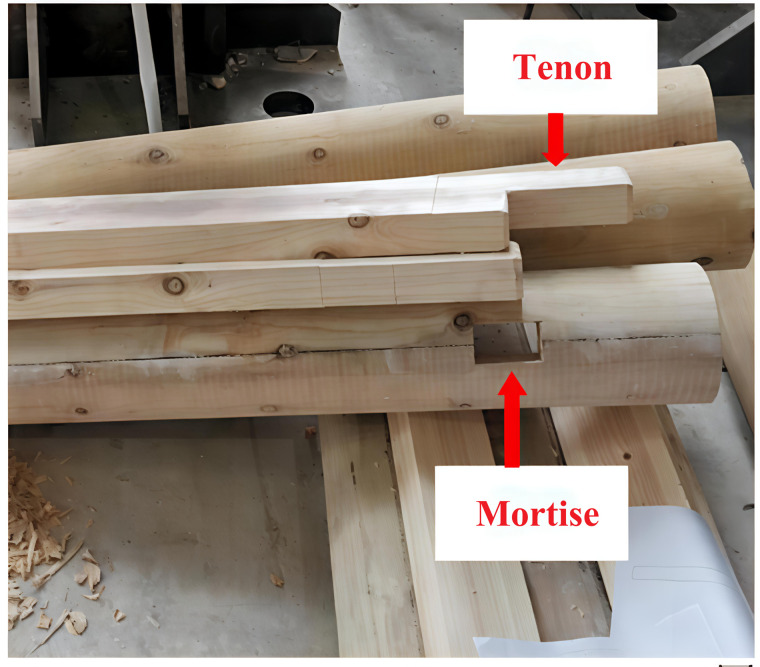
Actual images of tenons and mortises.

In finite element analysis (FEA), the mortise-and-tenon joints of timber structures act as pivotal load-bearing and stress-transferring interfaces. Their mechanical response governs the structural integrity and performance of the entire framework. During mesh generation, the spatial discretization density directly influences the accuracy of numerical solutions. Given the pronounced stress concentrations typically observed at beam-column connections in timber frameworks, a refined mesh strategy is imperative at these critical junctures to capture localized stress gradients and nonlinear deformation patterns. Fig 7 and 8 depict the mesh configuration of beams and columns, while Fig 9 provides a photographic reference of an actual mortise-and-tenon joint.

The anisotropy of wood arises primarily from the unique arrangement of its fiber structure. At the macroscopic level, wood can be categorized into three basic directions: longitudinal, radial, and tangential. In the longitudinal direction, which aligns with the growth of the trunk (i.e., the direction of the smooth grain), the fibers are closely aligned, providing the wood with maximum strength and stiffness. Conversely, the cross-section perpendicular to the growth direction can be divided into two additional directions: radial, which extends along the annual rings, and tangential, which runs tangentially to the rings. The fibers in these two directions are arranged more sparsely, resulting in varying degrees of reduced mechanical properties. This structural variability causes wood to exhibit different behaviors depending on the direction of the applied forces. Typically, wood possesses the highest tensile strength in the smooth grain direction [28–30], while exhibiting relatively lower strength in the radial and tangential directions. Therefore, it is essential to account for these anisotropic properties when assigning directions to beams and columns.

**Fig 10:**
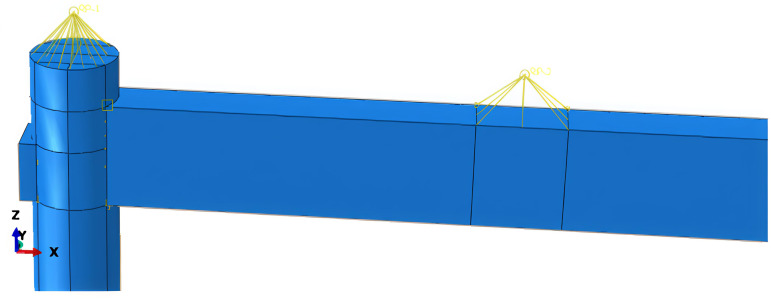
Setting vertical loads and connection points.

**Fig 11:**
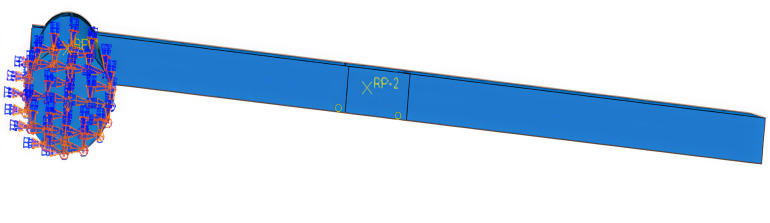
Securing connections for the base.

**Fig 12:**
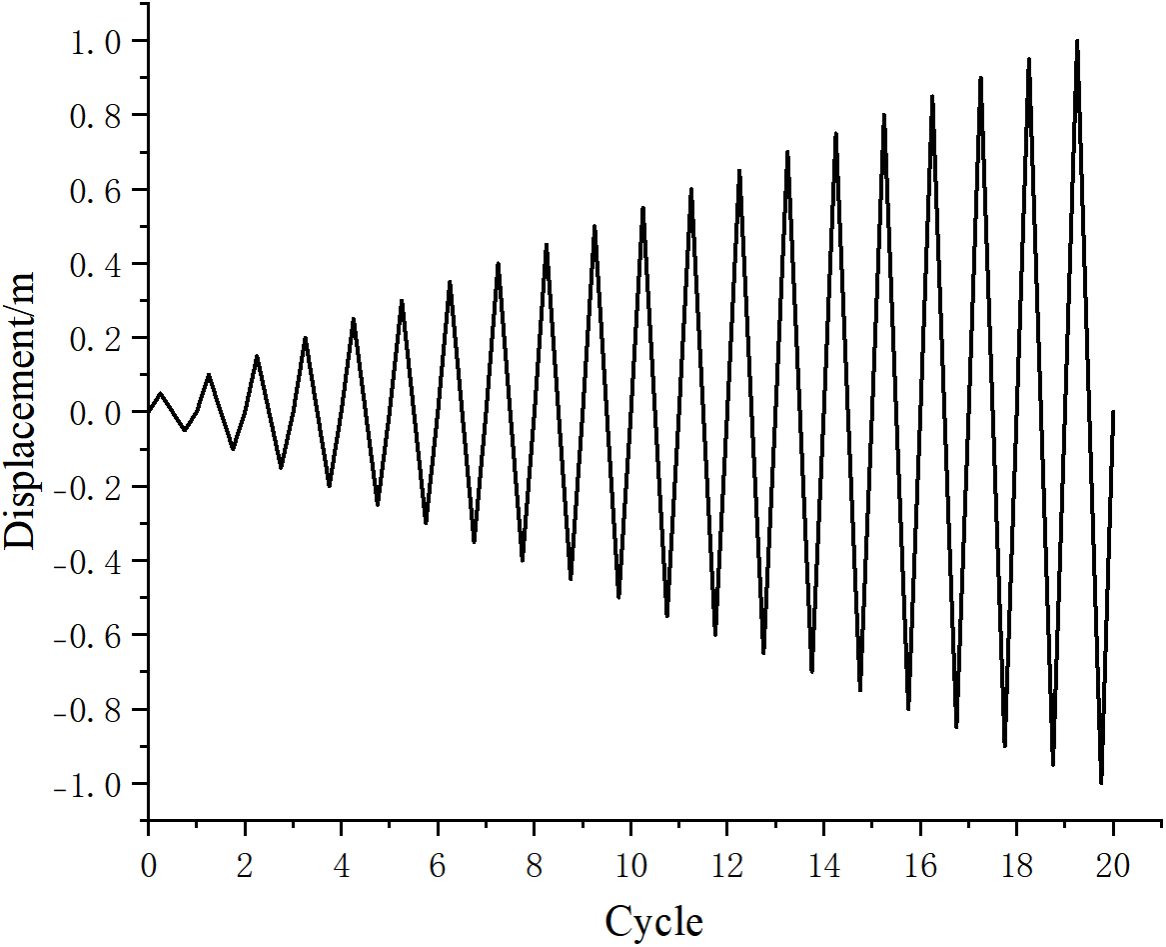
Reciprocating loading curve.

Wood is a heterogeneous material. In finite element software, its elastic behavior is typically characterized by defining engineering constants, while the plastic phase is described by specifying the yield stress ratios for each direction within the material properties options of the finite element model. To accurately represent the actual forces on the wooden column, vertical loads are applied above the column, and friction contact surfaces are established between the beam and the column. The interaction between nodes mainly involves the normal and tangential actions of each contact surface. In this paper, the “hard” contact model and “penalty” friction are selected to define the mechanical behavior between the mortise and tenon. Surface-to-surface contact is adopted for each contact surface, and the sliding magnitude is finite sliding. To prevent the slave surface from penetrating the master surface during loading, which may lead to non-convergence of results, the contact surface of the mortise is defined as the master surface, and the contact surface of the tenon is defined as the slave surface. These settings enhance the representation of the real-world conditions, allowing for more effective friction energy dissipation within the mortise and tenon structure. The configurations for vertical loads and friction surfaces are illustrated in Fig 10. The bottom of the wooden column is set with a fixed connection. The purpose of the fixed connection at the bottom is to restrict the translation and rotation of the wooden column in three directions. The fixed connection setting of the base is shown in Fig 11. The hysteretic loading curve is shown in Fig 12 [31].

## 3. Static analysis

### 3.1. Theory of penetrating mortise and tenon joints

#### 3.1.1. Basic assumptions.

**Fig 13:**
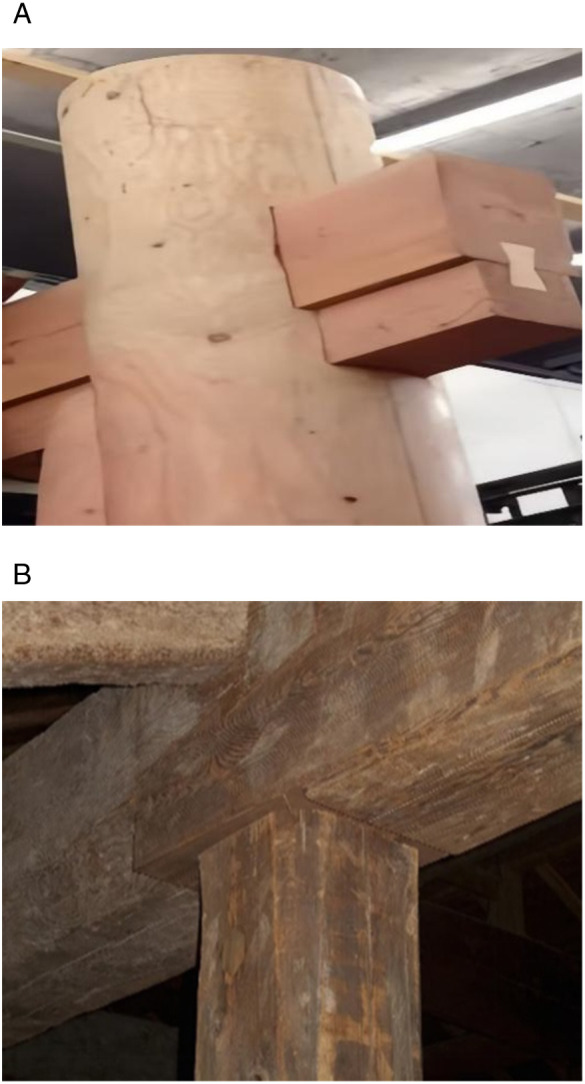
Exterior view of mortise and tenon joint. (a) Through-tenon beam-column joint (b) Straight tenon beam-column joint.

Mortise and tenon joints are subjected to repeated pulling and compression, leading to significant deformation at the joints and increased susceptibility to damage at critical and vulnerable points. These joints are essential connecting elements in wood frame buildings. In Fig 13 (a) and (b), one can observe the classic types of mortise and tenon joints that were characteristic of ancient Tibetan building structures. It is assumed that the mortise and tenon joints at the joints of the wooden structure exhibit minimal slipping, allowing the penetrating mortise to contribute to the stability of the structure. Generally, penetrating tenons are more stable than straight tenons; thus, in our model assumptions, we consider that the interface between the beam tenon and the head of the post remains intact. Based on theoretical mechanics formulas [32], the following basic assumptions are made:

(1) The rotation of a wood structural joint typically occurs at the center of the through-pin location. The wood structural joint is considered semi-rigid, with the assumption of minimal deformation.
(2) It is assumed that the mortise and tenon of a component will deform due to the frictional interactions of the components. When displacement occurs at the interface between the beam tenon and the column, smooth grain compression will take place at the column interface, while transverse grain compression will occur at the beam tenon. Literature indicates that the modulus of elasticity of wood under compression in the smooth grain direction is significantly greater than that in the transverse grain direction.
(3) For simplification of calculations, it is assumed that the joints of the wood structure are semi-rigid and can undergo rigid-body motion, allowing us to ignore the deformation of the mortise and tenon joints during cyclic loading.
(4) In engineering design and mechanical analysis, systems are often simplified to facilitate calculations and understanding. A common assumption is that the friction at the lateral contact surfaces is negligible, particularly when considering the low friction between mortise and tenon joints. This assumption simplifies the problem, making the analysis more intuitive and feasible.

#### 3.1.2. Equations for bending moments and angles at penetrating tenon joints.

1) Elasticity stage
  (1) The plane geometry condition is established as a prerequisite. In the through mortise joint, the angle between the direction of rotation at the connection point of the wooden beam and column and the horizontal plane is denoted as *θ*. When the joint is subjected to a vertical downward load, the plane mechanical relationships governing the deformation process at the body-structure connection are illustrated in Fig 13 (a), (b). Let $\;I_{A},I_{B}$ represent the lengths of the bottom and top surfaces of the mortise that are subject to compression; $\;\delta_{A},\;\delta_{B}$ denote the maximum compressive deformation at these surfaces; h be the height of the tenon in the wooden beam; and d represent the diameter of the wooden beam and column.

**Fig 14:**
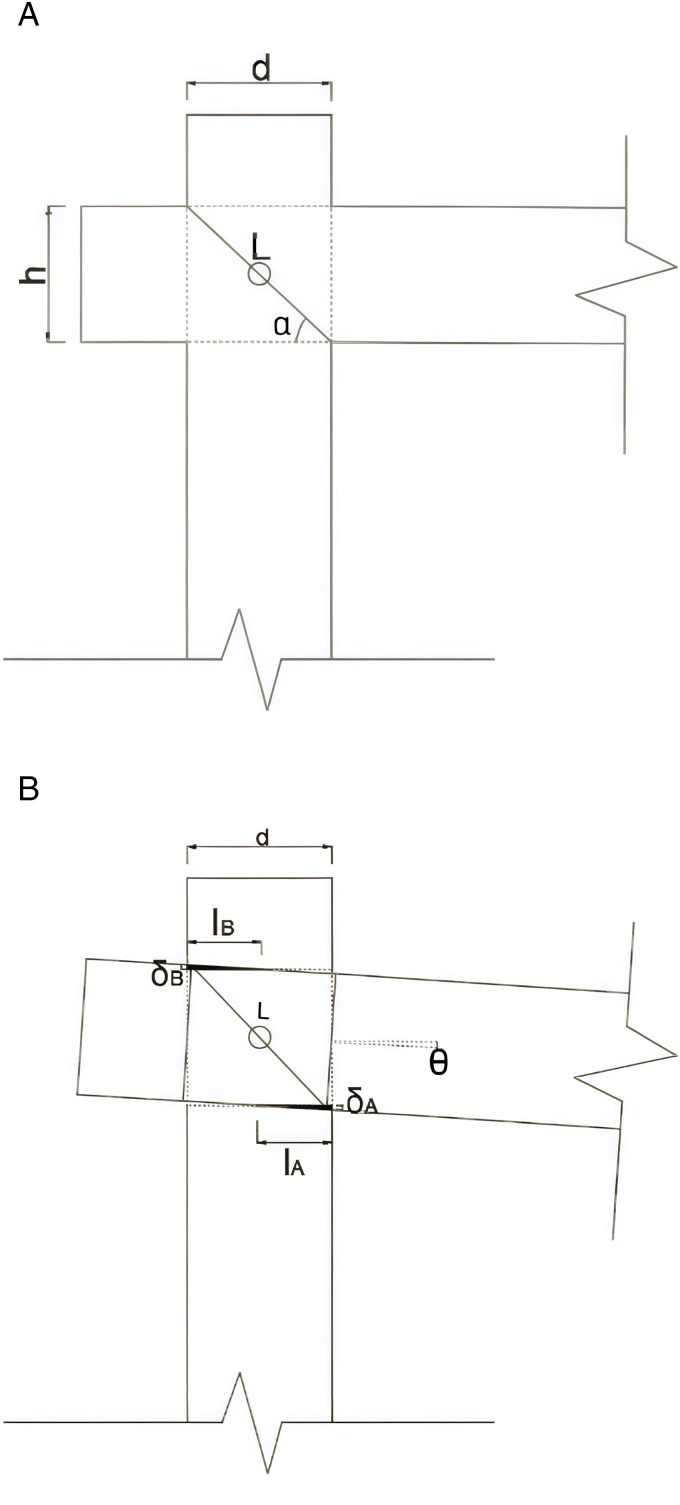
Schematic diagram before and after plane deformation. (a) Pre-action (b) Post-action.


From the geometric relationship shown in Fig 14, Eq. 1 can be derived, whose, $L=\sqrt{h^{2}+d^{2}}$, a=arctan(h/d). Since the center of the penetrating mortise is the geometric center of the wood structural joint, the lengths of the regions where the upper and lower portions of the beam and column are in compression contact are theoretically equal. Thus, we can conclude that these lengths are the same, and therefore the conclusion can be obtained as $l_{A}=l_{B}$. Then one can substitute $\delta_{A}=l_{A}\tan\theta$ and $\delta_{B}=l_{B}\tan\theta \;$ into Eq. 1 to obtan Eq. 2 as follow.

$$\left(h+\delta_{A}+\delta_{B}\right)\sin\left(\alpha -\theta \right)=L\sin\alpha$$ (1)

$$l_{A}=l_{B}=\frac{L\sin\alpha }{2\tan\theta \left(\sin\left(\alpha -\theta \right)-h\right)}$$ (2)

**Fig 15:**
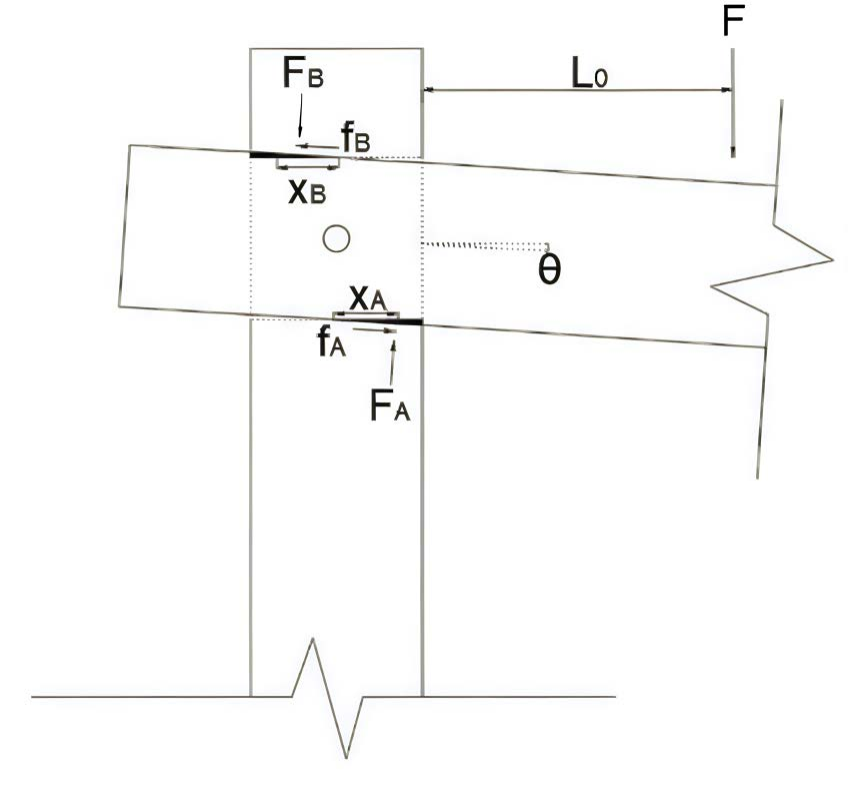
Joint force diagram.

(2) Equilibrium Condition: The angle between the wooden beam of the through mortise joint and the horizontal direction is denoted as *θ*. The schematic diagram of the wooden structure joint under the action of a vertical downward load is shown in Fig 15. The contact surfaces at the through mortise joint can be disregarded due to the minimal moments of friction in the compression zones. Therefore, in this mechanical state, the center of the penetrating part of the joint serves as the geometric origin for calculating moments.

The equilibrium of forces at the joint can be described by Eq. 3. In this equation, $\;F_{A}$ and $\;F_{B}$ denote the vertical forces in the compression contact regions at the upper and lower parts of the beam-column, respectively. As shown in Eq. 4, it is evident that the distances from the left and right sides of the column to the force arms, which are associated with the upper and lower frictional contacts of the beam-column, can be derived. Eq. 5 allows us to obtain these distances. In this context, the friction at the upper and lower contact surfaces is represented by $\;f_{A}$ and $f_{B}$, respectively, with *μ* being the friction coefficient at the joint contact surfaces. Upon reviewing the literature and relevant specifications, it is found that the typical value for *μ* is 0.24.


$$F\left(l_{0}+\frac{d}{\text{2}}\right)=\left(f_{A}\frac{h}{\text{2}}+f_{B}\frac{h}{\text{2}}+F_{A}x_{A}+F_{B}x_{b}\right)$$ (3)

$$x_{A}=\frac{d}{\text{2}}-\frac{l_{A}}{\text{3}},x_{B}=\frac{d}{\text{2}}-\frac{l_{B}}{\text{3}}$$ (4)

$$f_{A}=\mu F_{A},f_{B}=\mu F_{B}$$ (5)

**Fig 16:**
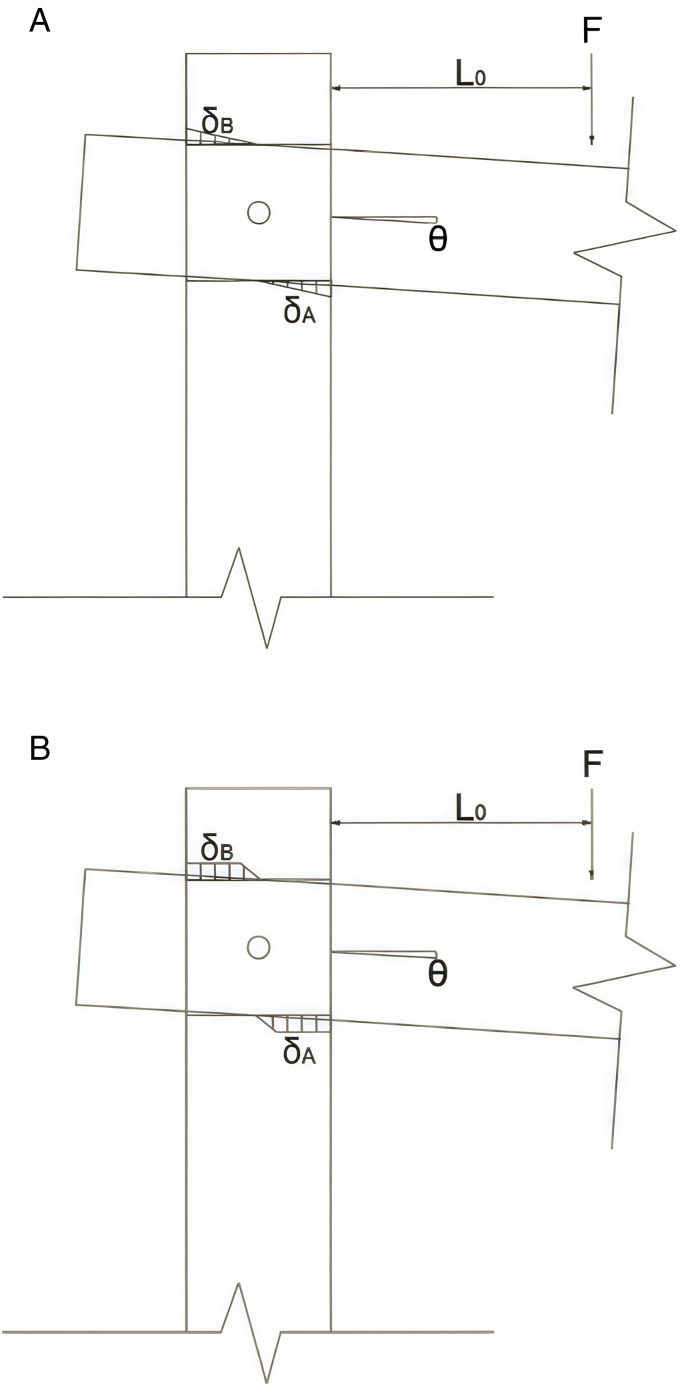
Joint elastic and plastic phases. (a) Resilient phase of wood structures (b) Elasto-plastic phase of wood structures.


(3) Physical Conditions of Wood Structure joints: When the wooden beam is angled relative to the horizontal direction, let this angle be denoted as θ. At this moment, the stress condition of the wooden structure joint is in the elastic phase, as shown in Fig 16(a). We assume that the elastic modulus for the transverse compression between the wooden column and beam is $E_{Rc}$ and the width of the contact area is denoted as b. When a vertical load is applied to the joint, the wooden beam initially enters the elastic stage. At this stage, the compressive stresses at the upper and lower surfaces of the beam-column contact do not exceed the yield strength of the wood in the transverse direction. The maximum compressive stresses at the upper and lower surfaces are denoted as $\sigma_{A}=\frac{E_{Rc}\sigma_{A}}{h}$ and $\;\sigma_{B}=\frac{E_{Rc}\sigma_{B}}{h}$. By applying the equilibrium conditions of forces and utilizing the aforementioned formulas, we can derive the mechanical relationships for the contact surfaces in the upper and lower compression zones. The forces are inversely proportional to the height, as indicated in Eq. 6 and 7.

$$F_{A}=\frac{\sigma_{A}l_{A}b}{\text{2}}=\frac{E_{Rc}b\tan\theta }{2h}l_{A}^{2}$$ (6)

$$F_{B}=\frac{\sigma_{B}l_{B}b}{\text{2}}=\frac{E_{Rc}b\tan\theta }{2h}l_{B}^{2}$$ (7)

(4) Through the mechanical theory of the elastic phase of wood, we can analyze the wooden structure of beams and columns, particularly the mortise and tenon joints. By examining the conditions of these joints, we can derive the numerical values for the lengths of the compression zones in the upper and lower halves of the beams and columns, denoted as $l_{A}=l_{B}.$ Additionally, we can determine the forces $\;F_{A}\;,\;\;F_{B}$ exerted in the upper and lower halves of the beams and columns, respectively, based on the physical conditions at the joint indicated as (3). These values can then be substituted into the equations of equilibrium to calculate the vertical force *F* acting on the beam. Since the distance of *F* from the mortise and tenon interface is *L*_*0*_, we can use the bending moment formula to find the bending moment of the structure at this point, which is given by $\;M=FL_{0}$.

2) Elasto-plasticity of through-tenon joints

When the through mortise joint is in a plastic-elastic state, the plane geometry and force equilibrium conditions at the connection between the beam and the wooden column are nearly identical to those in the elastic state, as referenced by the conditions and theories discussed earlier. In the plastic-elastic state of the beam-column connection, the stress state of the upper and lower halves in contact is illustrated in Fig 16(b). Assuming the transverse yield strength of the wood structure is $\sigma_{C,R}$, the prerequisite for yield strength is that the maximum compressive stress is reached at the contact points of the upper and lower halves of the beam and column connection. From the state diagram in Fig 16(b), it can be observed that the compressive stress region has a trapezoidal shape.

For wooden beams and columns connected at the pressure point, we assume that the stress distribution can be described by the theoretical derivation of the stress formula: $\;\sigma =\sigma_{C,R}+E_{2}\left(\frac{\sigma }{h}-\frac{\sigma_{C,R}}{E_{RC}}\right)$. Using this formula, we can calculate the stresses $\sigma_{A}$ and $\;\sigma_{B}$ according to Eq. 8 and Eq. 9. In these equations, $E_{2}$ represents the tangent modulus of wood, and literature review indicates that $\;E_{2}=0.38E_{RC}$ Assuming that the compressive stresses at the connection between the wooden beam and post have reached the yield strength at positions with lengths $l_{A0}$ and $l_{B0}$, we can calculate these lengths as follows: $l_{A0}=l_{A}-\frac{\sigma_{C,R}h\tan\theta }{E_{RC}}$ and $\;l_{B0}=l_{A}-\frac{\sigma_{C,R}h\tan\theta }{E_{RC}}$. From these formulas, we can determine the compressive stresses at the nodal joints, $F_{A}$ and $F_{B}$.

To determine the force arm of the compressive stress, it is essential to locate the centroid of the stress’s image shape. The values of the force arms for $F_{A}$ and $F_{B}$ can be derived using the formulas presented in Eq. 12 and Eq. 13. With the given conditions, the lengths of the upper and lower pressurized portions of the connection between the wooden beam and column, denoted as $\;l_{A},l_{B}$, along with the pressurized stresses $F_{A}$ and $F_{B},$ are articulated in Eq. 10 and Eq. 11. These can be resolved by substituting $l_{A},l_{B}$ into Eq. 8 and Eq. 9. The final bending moment is then calculated in a manner analogous to that used for the elastic phase.


$$\sigma_{A}=\sigma_{C,R}+\left(\frac{\delta_{A}}{h}-\frac{\sigma_{C,R}}{E_{RC}}\right)E_{2}=\sigma_{C,R}+\left(\frac{l_{A}\tan\theta }{h}-\frac{\sigma_{C,R}}{E_{RC}}\right)E_{2}$$ (8)

$$\sigma_{B}=\sigma_{C,R}+\left(\frac{\delta_{B}}{h}-\frac{\sigma_{C,R}}{E_{RC}}\right)E_{2}=\sigma_{C,R}+\left(\frac{l_{B}\tan\theta }{h}-\frac{\sigma_{C,R}}{E_{RC}}\right)E_{2}$$ (9)

$$F_{A}=\frac{\sigma_{C,R}b}{\text{2}}\left(l_{A}+l_{A0}\right)+\frac{\left(\sigma_{A}-\sigma_{C,R}\right)b}{\text{2}}l_{A0}$$ (10)

$$F_{B}=\frac{\sigma_{C,R}b}{\text{2}}\left(l_{B}+l_{B0}\right)+\frac{\left(\sigma_{B}-\sigma_{C,R}\right)b}{\text{2}}l_{B0}$$ (11)

$$x_{A}=\frac{d}{\text{2}}-\frac{\frac{\sigma_{C,R}}{\text{2}}l_{A0}^{2}+\sigma_{C,R}\left(l_{A}-l_{A0}\right)\left(\frac{\text{1}}{\text{3}}l_{A}+\frac{\text{1}}{\text{2}}l_{A0}\right)+\left(\sigma_{A}-\sigma_{C,R}\right)\frac{l_{A0}^{2}}{\text{6}}}{\frac{F_{A}}{b}}$$ (12)

$$x_{B}=\frac{d}{\text{2}}-\frac{\frac{\sigma_{C,R}}{\text{2}}l_{B0}^{2}+\sigma_{C,R}\left(l_{B}-l_{B0}\right)\left(\frac{\text{1}}{\text{3}}l_{B}+\frac{\text{1}}{\text{2}}l_{B0}\right)+\left(\sigma_{B}-\sigma_{C,R}\right)\frac{l_{B0}^{2}}{\text{6}}}{\frac{F_{B}}{b}}$$ (13)

### 3.2. Finite element analysis of mortise – tenon joints in Tibetan-style ancient house

#### 3.2.1. Horizontal and smooth stresses.

**Fig 17:**
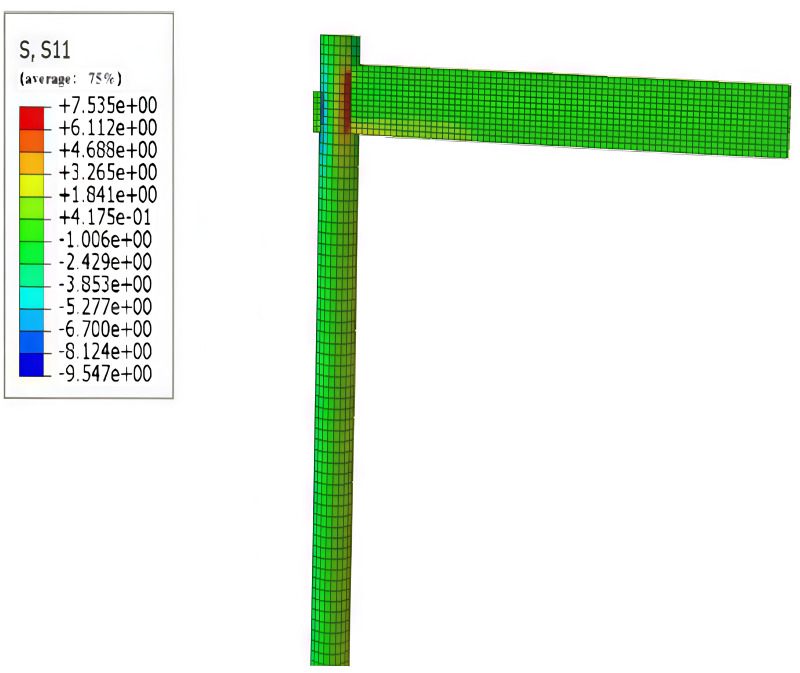
Maximum stress along grain.

**Fig 18:**
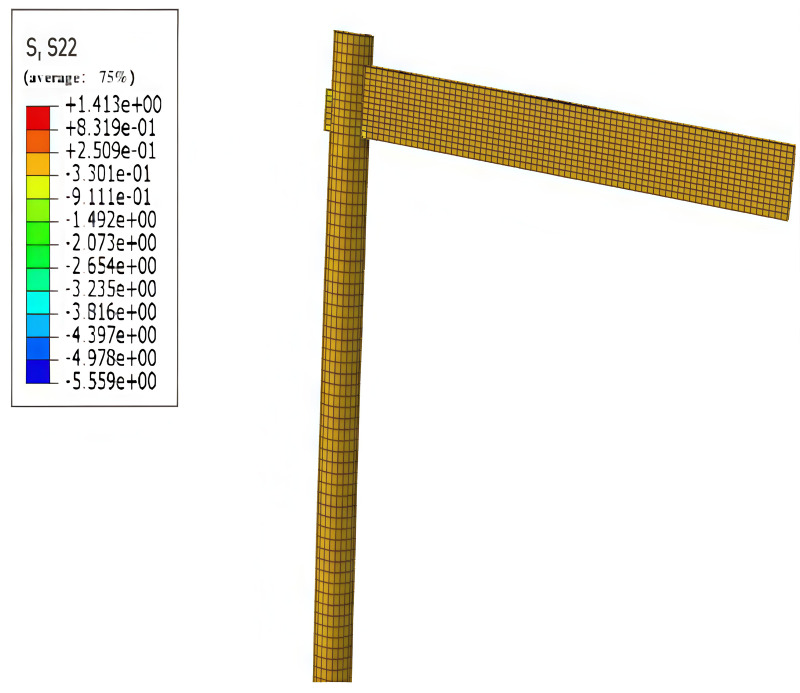
Maximum transverse stress.

Wood is an anisotropic material, exhibiting high tensile and compressive strengths along the smooth grain direction. However, its strength is significantly diminished in the case of transverse grain, particularly regarding tensile strength, where the transverse grain is prone to cracking and faults. Therefore, it is advisable to avoid tensile stress conditions in wood with transverse grain as much as possible during structural design. This concept is illustrated in Fig 17 and Fig 18 below.

The data in the Figures indicate that wood exhibits higher tensile, compressive, and flexural strengths in the down-grain direction compared to the cross-grain direction. This alignment reinforces that the assignment of cross-sectional directions in the modeling setup is correct, consistent with the observation that wood structures experience greater stresses in the down-grain direction than in the cross-grain direction.

#### 3.2.2. Stresses in mortise and tenon joints and slippage.

**Fig 19:**
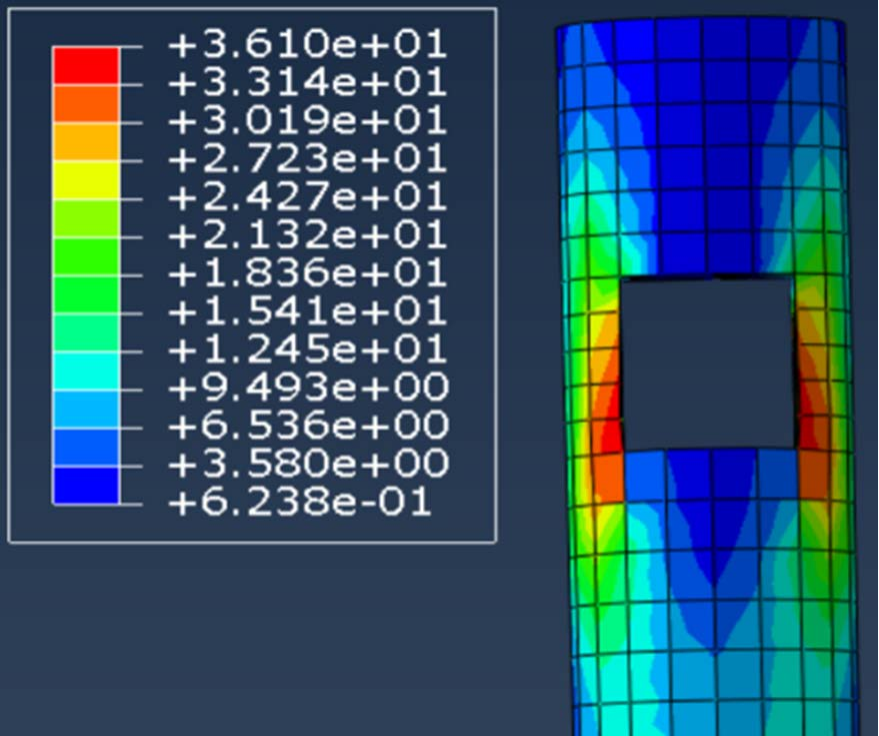
Column stress.

By comparing the stress distributions for through and straight tenons, the locations of the highest stresses in wood-frame joints can be identified. As shown in Fig 19, elevated stresses are observed at the left and right ends of the column interface. Additionally, the middle and lower sections of the column-beam joint exhibit significant stress concentrations. Therefore, when selecting reinforcement for the columns, it is advisable to focus on reinforcing the areas around the left and right ends of the interface. Reinforcement could include the incorporation of a ring-shaped steel plate at the interface to enhance structural integrity. This research outcome holds profound significance for the conservation and restoration of ancient Tibetan dwellings. It offers meticulous technical guidance for these preservation efforts, enabling the reinforcement of crucial structural components without compromising the original architectural charm. By doing so, it effectively bolsters the stability and safety of these ancient Tibetan abodes, ensuring the enduring continuity of the rich historical and cultural values they embody.

**Fig 20:**
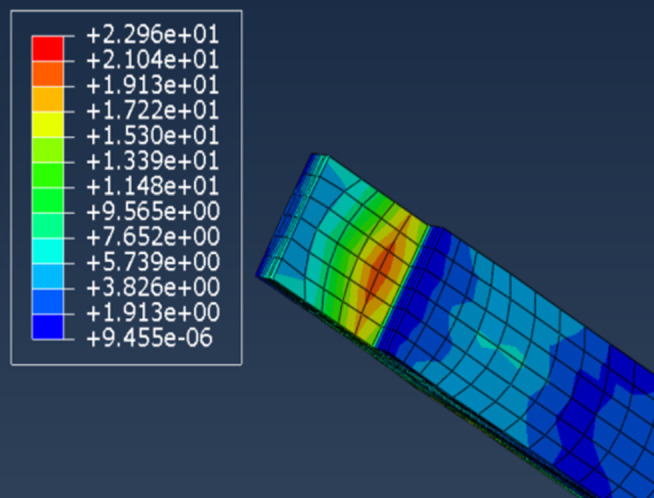
Beam stress.

The stress distribution for the beams is illustrated in Fig 20, indicating that the highest stresses are generally concentrated at the lower edges of the beams near the column openings and at the internal joints of the mortise and tenon connections. This offers a highly precise pinpointing for the conservation and restoration undertakings of ancient Tibetan dwellings, endowing these endeavors with a crucial navigational marker.

**Fig 21:**
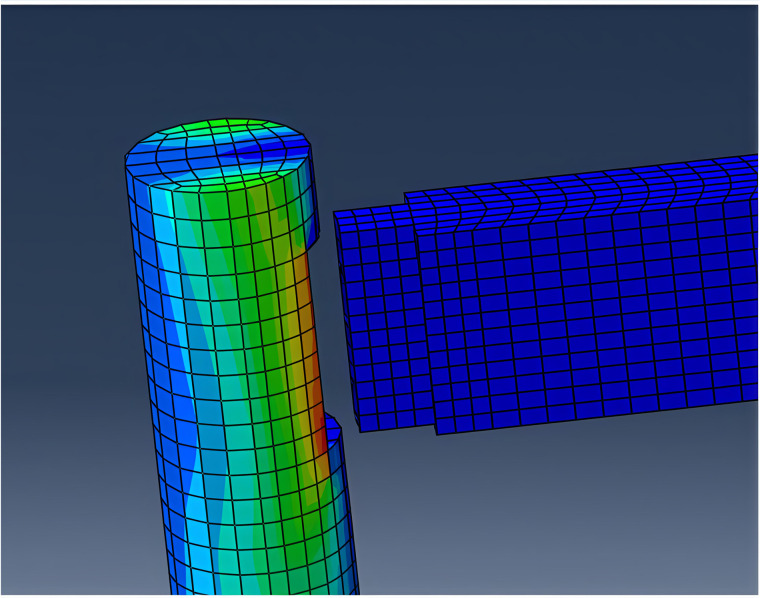
Straight tenon slip.

Under cyclic loading, the straight tenon joints, as shown in Fig 21, exhibited a slipping condition after 12 seconds, causing the beam to become ineffective during the reciprocation process. In contrast, the penetrating mortise and tenon joints did not exhibit any slipping throughout the entire loading process. Therefore, when it comes to the conservation and restoration of ancient Tibetan dwellings, the employment of through – tenon joints should be given precedence. For those extant structures that currently utilize straight tenon joints, a comprehensive assessment of the joint conditions is essential. Specifically, for the straight tenon joints prone to slippage, appropriate measures such as reinforcement or conversion into through – tenon structures should be implemented. This approach aims to enhance the stability and dependability of the wooden structure, thereby ensuring the long – term preservation of these ancient Tibetan abodes.

#### 3.2.3. Hysteresis curves of joints.

**Fig 22:**
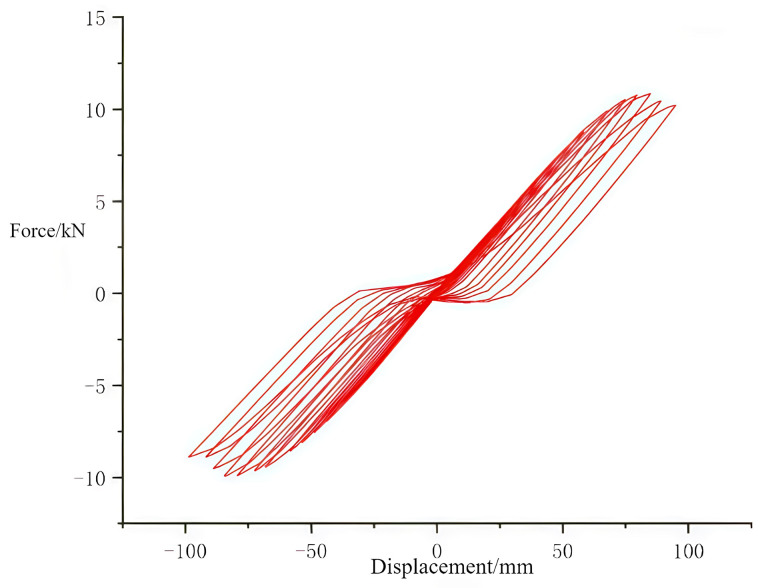
Hysteresis curve for through tenon.

**Fig 23:**
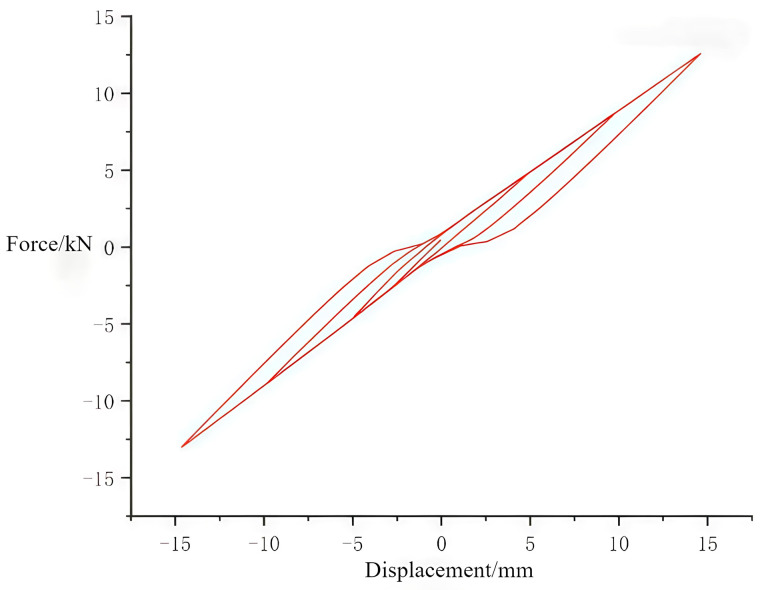
Hysteresis curve for straight tenon.

The role of the hysteresis curve primarily reflects the deformation characteristics of the structure during the application of reciprocating vertical loads, including structural stiffness degradation and energy-dissipation capacity [33–37]. It serves as the theoretical basis for analyzing whether the structure can return to its original state and its response to nonlinear actions. The area enclosed by the cycles in the load-displacement graphs of the hysteresis curve indicates the energy absorbed by the structure during reciprocal motion [38–39]. Fig 22 and Fig 23 display the hysteresis curves for the through tenon and straight tenon, respectively. It can be observed that the hysteresis curves for the through tenon are fuller than those for the straight tenon. Finite element analysis indicates that the through tenon demonstrates superior frictional energy dissipation in the mortise and tenon system. Therefore, in practical engineering applications, adopting the through tenon connection is more appropriate.

## 4. Dynamic analysis

### 4.1. Selection of seismic waves

When choosing seismic waves for Tibetan-style ancient house, the selection criteria should be grounded in the site category where these dwellings are situated, along with crucial parameters like the self – period of the dwellings and the base shear force derived from modal analysis. Earthquakes are highly intricate dynamic phenomena, with their magnitudes and directions of action being inherently uncertain. Consequently, during the structural time – history analysis of ancient Tibetan dwellings, multiple distinct seismic waves are introduced. By comprehensively evaluating the finite – element analysis results generated under the influence of these waves, the conclusions reached can possess a certain level of generality. This holds significant guiding value for the practical conservation and restoration projects of ancient Tibetan dwellings. It can contribute to enhancing the safety and stability of these dwellings in earthquake – prone environments.

**Fig 24:**
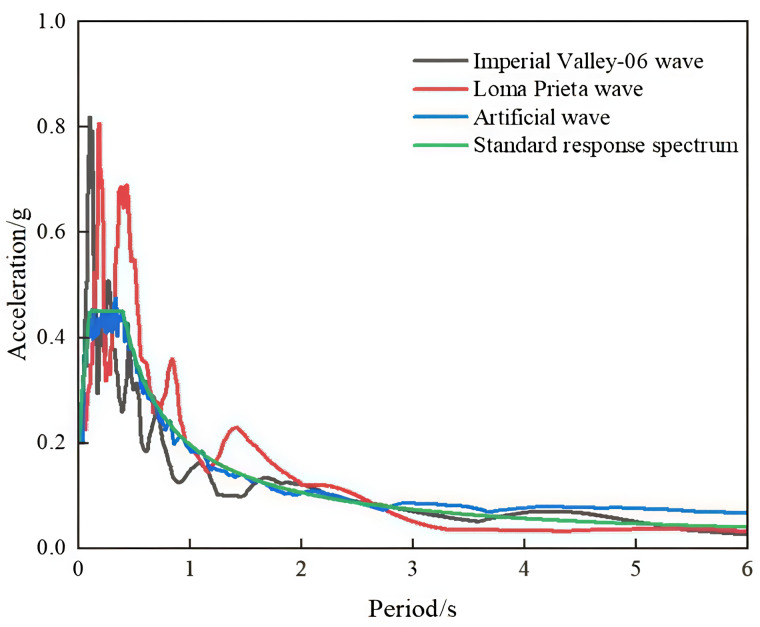
Loaded acceleration response spectrum.

When an earthquake occurs, the energy released is influenced not only by time but also by various factors, including the characteristics of buildings, soil, and the structure itself. The self-resonance period of this wooden structure is determined according to the seismic code and site classification in the Tibetan region, following modal analysis. Seismic waves were selected in accordance with the regulations outlined in the Building Seismic Design Code: GB50011−2010 leading to adjustments in the peak acceleration of the seismic waves listed in Table 2 to 0.4 g and 0.6 g. This adjustment ensures that the structure can withstand rare or very rare earthquakes while mimicking actual working conditions. Consequently, this approach enables analysis of the structure’s response under significant seismic loading. The acceleration response spectra are illustrated in Fig 24.

**Table 2:** Selected seismic wave information.

Serial number	Magnitude	Time	Name	Fault distance (km)	Station	Vs_30 (m/s)	PGA (g)	PGV (cm/s)
1	6.53	1979	Imperial Valley-06	10.42	Bonds Corner	208.71	0.168	16.07
2	6.93	1989	Loma Prieta	70.90	Anderson Dam	190.42	0.341	10.87
3	\	\	Art Wave	\	\	\	0.100	12.73

### 4.2. Seismic performance analysis of Tibetan-style ancient house

#### 4.2.1. Modal analysis.

**Fig 25:**
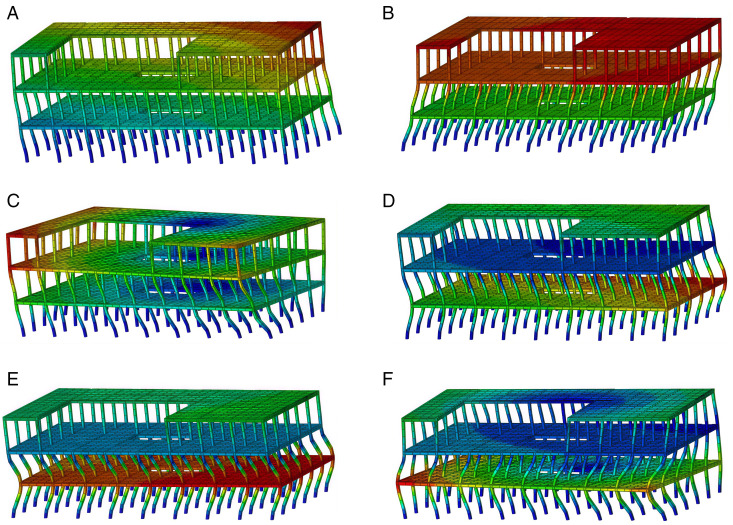
Modal of wood structure in Tibetan area. (a) First-order modes of Tibetan-style ancient house (b) Second-order modes of Tibetan-style ancient house (c)Third-order modes of the Tibetan-style ancient house (d) Fourth-order modes of Tibetan-style ancient house (e) Fifth-order modes of Tibetan-style ancient house (f) Sixth-order modes of Tibetan-style ancient house.

The self-resonance period of the Tibetan-style ancient house, obtained through finite element analysis, is presented in Table 3. Wooden structures differ from concrete structures in that they generally have lower structural mass. Additionally, the mortise and tenon joints effectively maintain structural integrity and energy dissipation, thereby prolonging the self-resonance period of the wooden structure. Consequently, these characteristics are associated with enhanced seismic performance [40], helping to minimize the adverse effects of earthquakes on the structure. Fig 25 illustrates the sixth-order modal analysis of the Tibetan-style ancient house.

**Table 3:** Self-oscillating cycle conditions of the Tibetan-style ancient house(s).

Order of modal analysis of the Tibetan-style ancient house	Self-resonance period of the Tibetan-style ancient house(s)
1	2.33
2	2.32
3	1.88
4	0.80
5	0.78
6	0.69

The six modal diagrams presented in Fig 25 demonstrate that the model aligns well with the general vibrational behavior of the structure. A comparison of the six models reveals that the modal analysis predominantly exhibits translational motion, with no significant localized vibrations observed. This indicates that the model is both reasonable and reliable.

#### 4.2.2. Angle of displacement between storeys of Tibetan-style ancient house.

**Fig 26:**
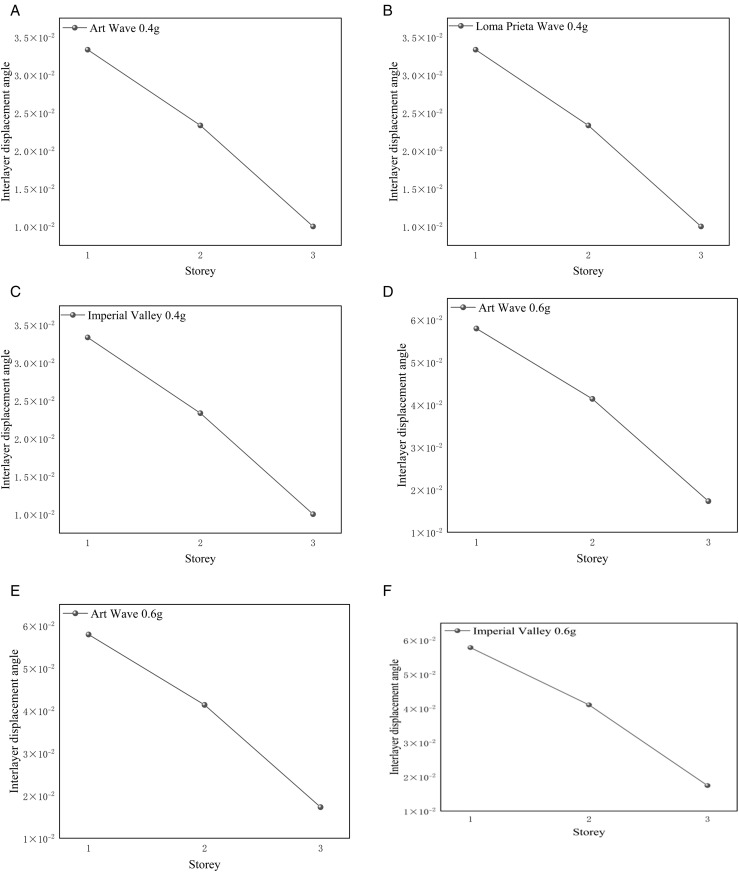
Interlayer displacement angle of Tibetan-style ancient house without considering the effect of earth wall. (a) Interlayer displacement angle of the Tibetan-style ancient house at the 0.4 g A-W wave (b) Interlayer displacement angle of the Tibetan-style ancient house at the 0.4 g L-P wave (c) Interlayer displacement angle of the Tibetan-style ancient house at the 0.4 g I-V wave (d) Interlayer displacement angle of the Tibetan-style ancient house at the 0.6 g A-W wave (e) Interlayer displacement angle of the Tibetan-style ancient house at the 0.6 g L-P wave (f) Interlayer displacement angle of the Tibetan-style ancient house at the 0.6 g I-V wave.

**Fig 27:**
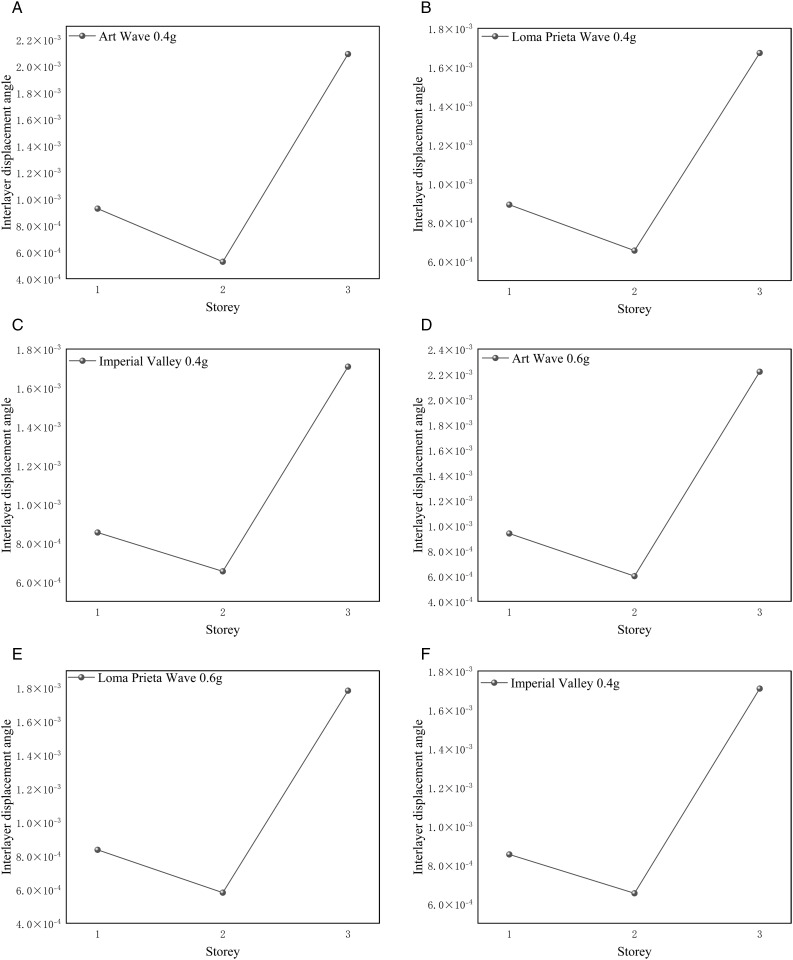
Interlayer displacement angle of Tibetan-style ancient house considering the action of earth wall. (a) Interlayer displacement angle of the Tibetan-style ancient house at the 0.4 g A-W wave (b) Interlayer displacement angle of the Tibetan-style ancient house at the 0.4 g L-P wave (c) Interlayer displacement angle of the Tibetan-style ancient house at the 0.4 g I-V wave (d) Interlayer displacement angle of the Tibetan-style ancient house at the 0.6 g A-W wave (e) Interlayer displacement angle of the Tibetan-style ancient house at the 0.6 g L-P wave (f) Interlayer displacement angle of the Tibetan-style ancient house at the 0.6 g I-V wave.

This project involves the design of a three-story wooden structure. Typically, the inter-story displacement angle should be maintained within a reasonable range to ensure that the building or structure possesses sufficient stiffness and stability under seismic and other dynamic loads. This project calculates the inter-story displacement angle as the ratio of the maximum horizontal displacement between floors to the height of the floor, based on the elastic method, for three types of seismic waves at 0.4 g and 0.6 g. The inter-story displacement angle is represented as Δu/h. Excessive displacement between stories may lead to structural failure, posing significant risks to personal safety and property. Therefore, the inter-story displacement angle is a crucial parameter in seismic design that requires careful control and evaluation. The Technical Standard for Prefabricated Timber Structure Buildings (GB/T 51233−2016) [41] specifies the range of inter-story drift angles for timber structures. For multi-storey and high-rise timber buildings, the inter-story drift angle shall not exceed 1/350. Fig 26 (a)-(f) and Fig 27 (a)-(f) illustrate the inter-story displacement angles of the Tibetan house under working conditions of 0.4 g and 0.6 g, comparing models with and without consideration of the earth wall.

Fig 26 (a), (b), and (c) indicate that the selected Tibetan-style ancient house, without considering the role of the earth wall, exhibits a maximum displacement angle of 0.033 during the seismic time history analysis for three seismic waves at 0.4 g. This value aligns with the normative limit of 1/30. As noted in the modal analysis of Section 4.2.1, the structure primarily exhibits translational motion during the seismic event. The bases of the columns on the third floor experience the highest loads and shear performance, resulting in greater inter-layer displacement at this level compared to the other floors. This observation is consistent with the structural performance indicated by the time history analysis. Fig 26 (d), (e), and (f) illustrate the inter-layer displacement angle of the Tibetan-style ancient house under the seismic action of 0.6 g. The effects of the 0.6 g seismic wave are more pronounced than those at 0.4 g, as indicated by the increased inter-layer displacement values observed in the comparative analysis. Fig 27(a)–(f) shows the inter-layer displacement angles for the Tibetan-style ancient house when considering the action of the earth wall. With the inclusion of the earth wall, the structure demonstrates smaller inter-layer displacements for both the 0.4 g and 0.6 g cases compared to scenarios without the earth wall. Using the formula for the inter-layer displacement angle, it is evident that at a given storey height, the primary factor affecting the inter-layer displacement angle is the inter-layer displacement itself. Therefore, it is believed that a wooden structure incorporating earth walls will enhance the stiffness of the overall system, providing lateral resistance against translation and improving structural stability.

Based on the analysis above, the following conclusions can be drawn:

(1) The base of the Tibetan-style ancient house experiences the highest vertical load and shear force. Therefore, enhancing the strength at the base, implementing reinforcement, or improving stability through seismic isolation measures is recommended.
(2) Incorporating walls into the wooden structure can enhance its overall stability, thereby further improving the seismic performance of the wooden framework.

#### 4.2.3. Acceleration at the top of the Tibetan-style ancient house.

**Fig 28:**
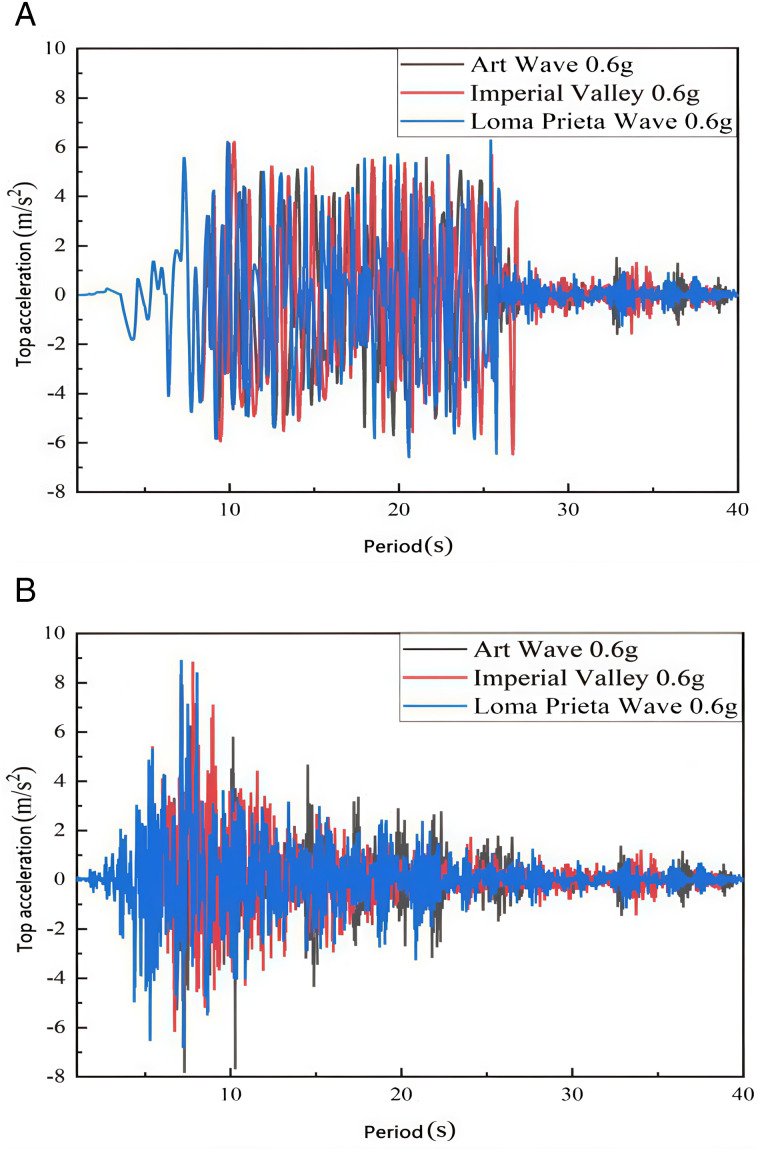
Acceleration at 0.4 g vs. 0.6 g Tibetan-style ancient house roof level. (a) Top acceleration without consideration of earth wall (b) Consideration of top acceleration of earth wall.

The acceleration at the top floor of the selected Tibetan dwelling is analyzed with and without considering the action of the earth wall. Generally, the number of floors significantly affects acceleration, exhibiting a linear relationship between the acceleration of each floor and that of the top floor. Therefore, examining the top-floor acceleration for comparative analysis more accurately reflects real conditions. Fig 28 (a) and (b) illustrate the roof acceleration of the Tibetan-style ancient house under 0.4 g and 0.6 g seismic waves, both considering and not considering the role of the earth wall.

Based on Fig 28 (a) and (b), the top floor acceleration of the Tibetan-style ancient house is assessed in both scenarios: with and without the addition of the earth wall. When the acceleration amplitude increases from 0.4 g to 0.6 g, the inter-layer acceleration also rises. As discussed in the previous subsection, the stiffness of the Tibetan-style ancient house without the earth wall is lower than that with the earth wall. Consequently, the fluctuations in top acceleration are greater in the absence of the earth wall, indicating that the earth wall effectively enhances the stiffness of the wooden structure, improving its ability to resist seismic forces and increasing overall stability. Furthermore, temporal observations reveal that with the addition of the earth wall, the acceleration at the top level of the structure gradually decreases during the first half of the time period. This behavior contrasts with the acceleration profile observed without the earth wall. Therefore, within the context of designing the wooden structures of ancient Tibetan dwellings, the incorporation of earth walls serves as a viable strategy to enhance structural stability. This addition not only augments the seismic performance of the dwellings, thereby safeguarding their integrity and the safety of inhabitants during seismic events and other natural disasters but also plays a crucial role in preserving the historical and cultural values enshrined within these structures. By reinforcing the structural integrity, we ensure the longevity and continued relevance of these ancient edifices as cultural heritage, passing down their unique architectural and historical significance to future generations.

#### 4.2.4. Localized stresses in Tibetan-style ancient house.

**Fig 29:**
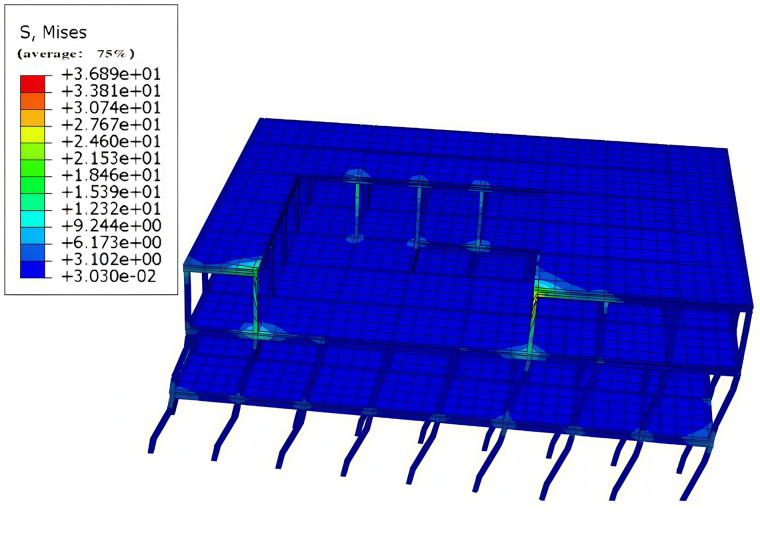
Maximum stresses in the Tibetan-style ancient house under operating conditions.

The maximum structural stress under the selected working conditions is illustrated in Fig 29, with a peak value of 36 MPa. During seismic events, significant stiffness changes occur on the third floor of the Tibetan residence, where the maximum stress is observed. The Fig. indicates that the two front columns near the hollow opening on the third floor experienced the highest stress, while the three columns at the back of the hollow opening also exhibit considerable stress. This phenomenon suggests that areas surrounding the hollow openings are subjected to greater stress. Therefore, reinforcing the side columns at these openings can enhance the seismic performance of the structure. Additionally, the overall seismic performance of the Tibetan residence can be further improved by incorporating the earth wall. This finding holds profound significance for the repair and restoration of ancient Tibetan dwellings. It serves as a scientific cornerstone for implementing targeted reinforcement measures during the restoration process. By doing so, it ensures that these ancient edifices remain robust and secure in the contemporary context, thereby safeguarding the unique historical and cultural legacies they embody. Moreover, it offers crucial guidance for the meticulous restoration efforts, enabling the preservation and revival of traditional architectural characteristics to the greatest extent possible. This not only facilitates the seamless transmission of Tibetan dwellings in their complete and authentic forms across generations but also cements their position as vital conduits for cultural heritage and exchange.

## 5. Discussion

The findings underscore the critical role of earth walls and through-tenon joints in enhancing seismic resilience. However, several methodological limitations should be noted. The finite element model, while capturing basic mechanical behaviors, relies on linear elastic assumptions for wood, potentially underrepresenting nonlinear effects like plastic deformation. Additionally, the seismic wave simulations, though based on code-compliant waveforms, may not fully replicate the complexity of real earthquakes, including variable frequencies and soil-structure interactions. Future research could incorporate advanced nonlinear material models and field monitoring to bridge these gaps, thereby refining the accuracy of seismic performance predictions for Tibetan-style ancient houses.

## 6. Conclusion

The following conclusions can be drawn from the simulation and analysis of two different structures, specifically through tenon joints and straight tenon joints:

(1) Seismic Performance of Tibetan-style ancient houses: The wooden structure of Tibetan-style ancient houses demonstrates certain seismic performance, achieving a level of stability. Under 0.4 g seismic waves, the stiffness of the Tibetan-style ancient house without the earth wall is lower than that with the earth wall. This reduced stiffness leads to greater displacement during an earthquake. The addition of the earth wall compensates for the structural rigidity deficiency. In the case of 0.6 g seismic waves, the Tibetan-style ancient house without the earth wall may exhibit instability; however, adding the earth wall significantly enhances the overall integrity and stiffness of the structure.
(2) Impact of Earth Walls on Acceleration and Stability: Finite element data indicate that there are instances when the top acceleration with the earth wall is greater than without it. Nevertheless, the addition of the earth wall drastically reduces the structure’s exposure to accelerations from earthquakes, shortening the duration of acceleration effects on the structure. Consequently, the earth walls increase the structure’s resistance to seismic forces. Local maximum stresses occur at floors with stiffness mutations and at structural joints, suggesting that reinforcing the side columns at these joints can improve seismic performance. It is evident that earth walls play a vital role in the stability of wooden structures.
(3) Modal analysis results indicate that the structure features a relatively long natural vibration period and primarily exhibits translational motion. This implies that such structures are likely to avoid the dominant frequency components of seismic waves during an earthquake, thereby reducing the risk of resonance—further validating the inherent seismic advantages of traditional wooden structures.
(4) Local stress analysis of the structure reveals that the front and rear columns adjacent to the hollow openings on the third floor experience relatively high stress. This suggests that during seismic reinforcement, in addition to enhancing the overall stiffness of the structure, targeted strengthening of components in these stress-concentrated areas is necessary.
(5) Anisotropic Properties and Interface Assignment: To fully exhibit the anisotropic properties of wood, it is essential to establish the correct interface assignment directions. The mortise and tenon joints are critical areas where maximum stresses occur at the connections between wood beams and columns. Therefore, a finer mesh should be utilized at the mortise and tenon connections to accurately depict stresses. The stress cloud analysis reveals that maximum stresses occur at the edges of the beam-column interface.
(6) Performance of Mortise and Tenon joints: Straight mortise and tenon joints exhibited loss of function and slippage of the beam during cyclic loading. A comparison of the hysteresis curves shows that the curve for the through tenon is fuller than that for the straight tenon, indicating that the through tenon possesses superior energy dissipation capacity compared to the straight tenon.

## Supporting information

S1 File Job-2–2000.doc File. Finite element models with and without earth walls. (DOC)

S2 File Job-EL200.doc File. Finite element model of earthquake with earth walls. (DOC)

S3 File Job-el400.doc File. Finite element model of earthquake without earth walls. (DOC)

S3 File Job-tenon1.doc File. Finite element model of through tenons. (DOCX)

S4 File Job-tenon2.doc File. Finite element model of straight tenons. (DOC)

S5 File Job-tenon3.doc File. Cyclic loading model of through tenons. (DOC)

S6 File Job-tenon4.doc File. Cyclic loading model of straight tenons. (DOC)
