# Supplementary material for: Seismic performance evaluation of ancient Tibetan residential structures and energy dissipation of Mortise-Tenon Joints
Source: PLoS One. 2025 Oct 27;20(10):e0334654. doi: 10.1371/journal.pone.0334654 (PMC12558541; doi:10.1371/journal.pone.0334654)
Supplement: S1 File — (DOC) [file pone.0334654.s001.doc]

*Heading

** Job name: Job-2-2000 Model name: Model-1

** Generated by: Abaqus/CAE 2023.HF1

*Preprint, echo=NO, model=NO, history=NO, contact=NO

**

** PARTS

**

*Part, name=LUANJIA

*Node

1, 24200., 200., 2700.

2, 24200., 0., 2700.

3, 24200., 0., 0.

4, 24200., 200., 0.

5, 24000., 0., 2700.

6, 24000., 0., 0.

7, 24000., 200., 0.

8, 24000., 200., 2700.

9, 24000., 15200., 2700.

10, 24200., 15200., 2700.

11, 24200., 15000., 2700.

12, 24000., 15000., 2700.

13, 24000., 3200., 2700.

14, 24200., 3200., 2700.

15, 24200., 3000., 2700.

16, 24000., 3000., 2700.

17, 24000., 6000., 2700.

18, 24200., 6000., 2700.

19, 24000., 6200., 2700.

20, 24200., 6200., 2700.

21, 24000., 9000., 2700.

22, 24200., 9000., 2700.

23, 24000., 9200., 2700.

24, 24200., 9200., 2700.

25, 24000., 12000., 2700.

26, 24200., 12000., 2700.

27, 24000., 12200., 2700.

28, 24200., 12200., 2700.

29, 24200., 15200., 3000.

30, 24200., 15000., 3000.

31, 24000., 15000., 3000.

32, 24000., 15200., 3000.

33, 24000., 12200., 3000.

34, 24200., 12200., 3000.

35, 24000., 12000., 3000.

36, 24200., 12000., 3000.

37, 24000., 9200., 3000.

38, 24200., 9200., 3000.

39, 24000., 9000., 3000.

40, 24200., 9000., 3000.

41, 24000., 6200., 3000.

42, 24200., 6200., 3000.

43, 24000., 6000., 3000.

44, 24200., 6000., 3000.

45, 24000., 3200., 3000.

46, 24200., 3200., 3000.

47, 24000., 3000., 3000.

48, 24200., 3000., 3000.

49, 24000., 200., 3000.

50, 24200., 200., 3000.

51, 24000., 0., 3000.

52, 24200., 0., 3000.

53, 24000., 15000., 2920.

54, 24000., 15200., 2920.

55, 24000., 3000., 2920.

56, 24000., 3200., 2920.

57, 24000., 6000., 2920.

58, 24000., 6200., 2920.

59, 24000., 9000., 2920.

60, 24000., 9200., 2920.

61, 24000., 12000., 2920.

62, 24000., 12200., 2920.

63, 24000., 200., 2920.

64, 24000., 0., 2920.

65, 24000., 15200., 0.

66, 24000., 15000., 0.

67, 24200., 15200., 0.

68, 24200., 15000., 0.

69, 24000., 12200., 0.

70, 24000., 12000., 0.

71, 24200., 12200., 0.

72, 24200., 12000., 0.

73, 24000., 9200., 0.

74, 24000., 9000., 0.

75, 24200., 9200., 0.

76, 24200., 9000., 0.

77, 24000., 6200., 0.

78, 24000., 6000., 0.

79, 24200., 6200., 0.

80, 24200., 6000., 0.

81, 24200., 15000., 5700.

82, 24000., 15000., 5700.

83, 24000., 15200., 5700.

84, 24200., 15200., 5700.

85, 24200., 12000., 5700.

86, 24000., 12000., 5700.

87, 24000., 12200., 5700.

88, 24200., 12200., 5700.

89, 24200., 9000., 5700.

90, 24000., 9000., 5700.

91, 24000., 9200., 5700.

92, 24200., 9200., 5700.

93, 24200., 6000., 5700.

94, 24000., 6000., 5700.

95, 24000., 6200., 5700.

96, 24200., 6200., 5700.

97, 24200., 3000., 5700.

98, 24000., 3000., 5700.

99, 24000., 3200., 5700.

100, 24200., 3200., 5700.

101, 24000., 200., 5700.

102, 24200., 200., 5700.

103, 24200., 0., 5700.

104, 24000., 0., 5700.

105, 24200., 12000., 6000.

106, 24200., 12200., 6000.

107, 24200., 12200., 8700.

108, 24200., 12000., 8700.

109, 24000., 12200., 6000.

110, 24000., 12200., 8700.

111, 24000., 12000., 8700.

112, 24000., 12000., 6000.

113, 24200., 15200., 6000.

114, 24200., 15000., 6000.

115, 24000., 15000., 6000.

116, 24000., 15200., 6000.

117, 24000., 9200., 6000.

118, 24200., 9200., 6000.

119, 24000., 9000., 6000.

120, 24200., 9000., 6000.

121, 24000., 6200., 6000.

122, 24200., 6200., 6000.

123, 24000., 6000., 6000.

124, 24200., 6000., 6000.

125, 24000., 3200., 6000.

126, 24200., 3200., 6000.

127, 24000., 3000., 6000.

128, 24200., 3000., 6000.

129, 24000., 200., 6000.

130, 24200., 200., 6000.

131, 24000., 0., 6000.

132, 24200., 0., 6000.

133, 24000., 15000., 5920.

134, 24000., 15200., 5920.

135, 24000., 3000., 5920.

136, 24000., 3200., 5920.

137, 24000., 6000., 5920.

138, 24000., 6200., 5920.

139, 24000., 9000., 5920.

140, 24000., 9200., 5920.

141, 24000., 12000., 5920.

142, 24000., 12200., 5920.

143, 24000., 200., 5920.

144, 24000., 0., 5920.

145, 24200., 15000., 8700.

146, 24000., 15000., 8700.

147, 24000., 15200., 8700.

148, 24200., 15200., 8700.

149, 24200., 9000., 8700.

150, 24000., 9000., 8700.

151, 24000., 9200., 8700.

152, 24200., 9200., 8700.

153, 24200., 6000., 8700.

154, 24000., 6000., 8700.

155, 24000., 6200., 8700.

156, 24200., 6200., 8700.

157, 24200., 3000., 8700.

158, 24000., 3000., 8700.

159, 24000., 3200., 8700.

160, 24200., 3200., 8700.

161, 24000., 200., 8700.

162, 24200., 200., 8700.

163, 24200., 0., 8700.

164, 24000., 0., 8700.

165, 24200., 200., 9000.

166, 24200., 3000., 9000.

167, 24200., 3200., 9000.

168, 24200., 6000., 9000.

169, 24200., 6200., 9000.

170, 24200., 9000., 9000.

171, 24200., 9200., 9000.

172, 24200., 12000., 9000.

173, 24200., 12200., 9000.

174, 24200., 15000., 9000.

175, 24200., 15200., 9000.

176, 24200., 0., 9000.

177, 24000., 200., 8920.

178, 24000., 0., 8920.

179, 24000., 3000., 8920.

180, 24000., 3200., 8920.

181, 24000., 6000., 8920.

182, 24000., 6200., 8920.

183, 24000., 9000., 8920.

184, 24000., 9200., 8920.

185, 24000., 12000., 8920.

186, 24000., 12200., 8920.

187, 24000., 15000., 8920.

188, 24000., 15200., 8920.

189, 24000., 0., 9000.

190, 24000., 15200., 9000.

191, 9000., 9000., 8920.

192, 6200., 9000., 8920.

193, 6200., 9000., 8700.

194, 9000., 9000., 8700.

195, 6200., 9200., 8920.

196, 6200., 9200., 8700.

197, 9000., 9200., 8700.

198, 9000., 9200., 8920.

199, 6200., 12200., 8920.

200, 9000., 12200., 8920.

201, 9000., 12200., 8700.

202, 6200., 12200., 8700.

203, 9000., 12000., 8920.

204, 9000., 12000., 8700.

205, 6200., 12000., 8700.

206, 6200., 12000., 8920.

207, 9000., 6200., 8700.

208, 9000., 6000., 8700.

209, 6200., 6000., 8700.

210, 6200., 6200., 8700.

211, 9000., 6200., 8920.

212, 9000., 6000., 8920.

213, 6200., 6000., 8920.

214, 6200., 6200., 8920.

215, 6200., 15200., 8920.

216, 9000., 15200., 8920.

217, 9000., 15000., 8920.

218, 6200., 15000., 8920.

219, 9000., 15200., 9000.

220, 9000., 15000., 9000.

221, 9000., 12200., 9000.

222, 9000., 12000., 9000.

223, 9000., 9200., 9000.

224, 9000., 9000., 9000.

225, 9000., 6200., 9000.

226, 9000., 6000., 9000.

227, 6200., 6000., 9000.

228, 6200., 15200., 9000.

229, 6200., 15000., 9000.

230, 6200., 12200., 9000.

231, 6200., 12000., 9000.

232, 6200., 9200., 9000.

233, 6200., 9000., 9000.

234, 6200., 6200., 9000.

235, 9200., 15200., 8920.

236, 12000., 15200., 8920.

237, 12000., 15200., 8700.

238, 9200., 15200., 8700.

239, 12000., 15000., 8920.

240, 12000., 15000., 8700.

241, 9200., 15000., 8700.

242, 9200., 15000., 8920.

243, 12000., 12000., 8920.

244, 9200., 12000., 8920.

245, 9200., 12000., 8700.

246, 12000., 12000., 8700.

247, 9200., 12200., 8920.

248, 9200., 12200., 8700.

249, 12000., 12200., 8700.

250, 12000., 12200., 8920.

251, 12000., 6200., 8700.

252, 12000., 6000., 8700.

253, 9200., 6000., 8700.

254, 9200., 6200., 8700.

255, 12000., 6200., 8920.

256, 12000., 6000., 8920.

257, 9200., 6000., 8920.

258, 9200., 6200., 8920.

259, 12000., 9000., 8920.

260, 9200., 9000., 8920.

261, 9200., 9000., 8700.

262, 12000., 9000., 8700.

263, 9200., 9200., 8920.

264, 9200., 9200., 8700.

265, 12000., 9200., 8700.

266, 12000., 9200., 8920.

267, 15000., 9000., 8920.

268, 12200., 9000., 8920.

269, 12200., 9000., 8700.

270, 15000., 9000., 8700.

271, 12200., 9200., 8920.

272, 12200., 9200., 8700.

273, 15000., 9200., 8700.

274, 15000., 9200., 8920.

275, 12200., 12200., 8920.

276, 15000., 12200., 8920.

277, 15000., 12200., 8700.

278, 12200., 12200., 8700.

279, 15000., 12000., 8920.

280, 15000., 12000., 8700.

281, 12200., 12000., 8700.

282, 12200., 12000., 8920.

283, 12200., 15200., 8920.

284, 15000., 15200., 8920.

285, 15000., 15000., 8920.

286, 12200., 15000., 8920.

287, 12200., 6200., 8920.

288, 15000., 6200., 8920.

289, 15000., 6000., 8920.

290, 12200., 6000., 8920.

291, 15000., 15200., 9000.

292, 15000., 15000., 9000.

293, 15000., 12200., 9000.

294, 15000., 12000., 9000.

295, 15000., 9200., 9000.

296, 15000., 9000., 9000.

297, 15000., 6200., 9000.

298, 15000., 6000., 9000.

299, 12200., 6000., 9000.

300, 12200., 15200., 9000.

301, 12200., 15000., 9000.

302, 12200., 12200., 9000.

303, 12200., 12000., 9000.

304, 12200., 9200., 9000.

305, 12200., 9000., 9000.

306, 12200., 6200., 9000.

307, 15000., 6200., 8700.

308, 15000., 6000., 8700.

309, 12200., 6000., 8700.

310, 12200., 6200., 8700.

311, 12000., 0., 2920.

312, 9200., 0., 2920.

313, 9200., 0., 2700.

314, 12000., 0., 2700.

315, 9200., 200., 2920.

316, 9200., 200., 2700.

317, 12000., 200., 2700.

318, 12000., 200., 2920.

319, 9200., 3200., 2920.

320, 12000., 3200., 2920.

321, 12000., 3200., 2700.

322, 9200., 3200., 2700.

323, 12000., 3000., 2920.

324, 12000., 3000., 2700.

325, 9200., 3000., 2700.

326, 9200., 3000., 2920.

327, 12000., 9000., 2920.

328, 9200., 9000., 2920.

329, 9200., 9000., 2700.

330, 12000., 9000., 2700.

331, 9200., 9200., 2920.

332, 9200., 9200., 2700.

333, 12000., 9200., 2700.

334, 12000., 9200., 2920.

335, 12000., 12000., 2920.

336, 9200., 12000., 2920.

337, 9200., 12000., 2700.

338, 12000., 12000., 2700.

339, 9200., 12200., 2920.

340, 9200., 12200., 2700.

341, 12000., 12200., 2700.

342, 12000., 12200., 2920.

343, 12000., 15000., 2920.

344, 9200., 15000., 2920.

345, 9200., 15000., 2700.

346, 12000., 15000., 2700.

347, 9200., 15200., 2920.

348, 9200., 15200., 2700.

349, 12000., 15200., 2700.

350, 12000., 15200., 2920.

351, 9200., 6200., 2920.

352, 12000., 6200., 2920.

353, 12000., 6200., 2700.

354, 9200., 6200., 2700.

355, 12000., 6000., 2920.

356, 12000., 6000., 2700.

357, 9200., 6000., 2700.

358, 9200., 6000., 2920.

359, 9000., 0., 2920.

360, 6200., 0., 2920.

361, 6200., 0., 2700.

362, 9000., 0., 2700.

363, 6200., 200., 2920.

364, 6200., 200., 2700.

365, 9000., 200., 2700.

366, 9000., 200., 2920.

367, 6200., 3200., 2920.

368, 9000., 3200., 2920.

369, 9000., 3200., 2700.

370, 6200., 3200., 2700.

371, 9000., 3000., 2920.

372, 9000., 3000., 2700.

373, 6200., 3000., 2700.

374, 6200., 3000., 2920.

375, 9000., 6000., 2920.

376, 6200., 6000., 2920.

377, 6200., 6000., 2700.

378, 9000., 6000., 2700.

379, 6200., 6200., 2920.

380, 6200., 6200., 2700.

381, 9000., 6200., 2700.

382, 9000., 6200., 2920.

383, 9000., 9000., 2920.

384, 6200., 9000., 2920.

385, 6200., 9000., 2700.

386, 9000., 9000., 2700.

387, 6200., 9200., 2920.

388, 6200., 9200., 2700.

389, 9000., 9200., 2700.

390, 9000., 9200., 2920.

391, 9000., 12000., 2920.

392, 6200., 12000., 2920.

393, 6200., 12000., 2700.

394, 9000., 12000., 2700.

395, 6200., 12200., 2920.

396, 6200., 12200., 2700.

397, 9000., 12200., 2700.

398, 9000., 12200., 2920.

399, 6200., 15200., 2920.

400, 9000., 15200., 2920.

401, 9000., 15000., 2920.

402, 6200., 15000., 2920.

403, 9000., 15200., 3000.

404, 9000., 15000., 3000.

405, 9000., 12200., 3000.

406, 9000., 12000., 3000.

407, 9000., 9200., 3000.

408, 9000., 9000., 3000.

409, 9000., 6200., 3000.

410, 9000., 6000., 3000.

411, 9000., 3200., 3000.

412, 9000., 3000., 3000.

413, 9000., 200., 3000.

414, 9000., 0., 3000.

415, 6200., 0., 3000.

416, 6200., 15200., 3000.

417, 6200., 15000., 3000.

418, 6200., 12200., 3000.

419, 6200., 12000., 3000.

420, 6200., 9200., 3000.

421, 6200., 9000., 3000.

422, 6200., 6200., 3000.

423, 6200., 6000., 3000.

424, 6200., 3200., 3000.

425, 6200., 3000., 3000.

426, 6200., 200., 3000.

427, 6200., 15200., 5920.

428, 9000., 15200., 5920.

429, 9000., 15200., 5700.

430, 6200., 15200., 5700.

431, 9000., 15000., 5920.

432, 9000., 15000., 5700.

433, 6200., 15000., 5700.

434, 6200., 15000., 5920.

435, 9000., 12000., 5920.

436, 6200., 12000., 5920.

437, 6200., 12000., 5700.

438, 9000., 12000., 5700.

439, 6200., 12200., 5920.

440, 6200., 12200., 5700.

441, 9000., 12200., 5700.

442, 9000., 12200., 5920.

443, 9000., 200., 5700.

444, 9000., 0., 5700.

445, 6200., 0., 5700.

446, 6200., 200., 5700.

447, 9000., 200., 5920.

448, 9000., 0., 5920.

449, 6200., 0., 5920.

450, 6200., 200., 5920.

451, 9000., 3000., 5920.

452, 6200., 3000., 5920.

453, 6200., 3000., 5700.

454, 9000., 3000., 5700.

455, 6200., 3200., 5920.

456, 6200., 3200., 5700.

457, 9000., 3200., 5700.

458, 9000., 3200., 5920.

459, 9000., 6000., 5920.

460, 6200., 6000., 5920.

461, 6200., 6000., 5700.

462, 9000., 6000., 5700.

463, 6200., 6200., 5920.

464, 6200., 6200., 5700.

465, 9000., 6200., 5700.

466, 9000., 6200., 5920.

467, 9000., 9000., 5920.

468, 6200., 9000., 5920.

469, 6200., 9000., 5700.

470, 9000., 9000., 5700.

471, 6200., 9200., 5920.

472, 6200., 9200., 5700.

473, 9000., 9200., 5700.

474, 9000., 9200., 5920.

475, 12000., 0., 5920.

476, 9200., 0., 5920.

477, 9200., 0., 5700.

478, 12000., 0., 5700.

479, 9200., 200., 5920.

480, 9200., 200., 5700.

481, 12000., 200., 5700.

482, 12000., 200., 5920.

483, 9200., 3200., 5920.

484, 12000., 3200., 5920.

485, 12000., 3200., 5700.

486, 9200., 3200., 5700.

487, 12000., 3000., 5920.

488, 12000., 3000., 5700.

489, 9200., 3000., 5700.

490, 9200., 3000., 5920.

491, 12000., 12000., 5920.

492, 9200., 12000., 5920.

493, 9200., 12000., 5700.

494, 12000., 12000., 5700.

495, 9200., 12200., 5920.

496, 9200., 12200., 5700.

497, 12000., 12200., 5700.

498, 12000., 12200., 5920.

499, 12000., 15000., 5920.

500, 9200., 15000., 5920.

501, 9200., 15000., 5700.

502, 12000., 15000., 5700.

503, 9200., 15200., 5920.

504, 9200., 15200., 5700.

505, 12000., 15200., 5700.

506, 12000., 15200., 5920.

507, 9200., 9200., 5920.

508, 12000., 9200., 5920.

509, 12000., 9200., 5700.

510, 9200., 9200., 5700.

511, 12000., 9000., 5920.

512, 12000., 9000., 5700.

513, 9200., 9000., 5700.

514, 9200., 9000., 5920.

515, 9200., 6200., 5920.

516, 12000., 6200., 5920.

517, 12000., 6200., 5700.

518, 9200., 6200., 5700.

519, 12000., 6000., 5920.

520, 12000., 6000., 5700.

521, 9200., 6000., 5700.

522, 9200., 6000., 5920.

523, 15000., 0., 2920.

524, 12200., 0., 2920.

525, 12200., 0., 2700.

526, 15000., 0., 2700.

527, 12200., 200., 2920.

528, 12200., 200., 2700.

529, 15000., 200., 2700.

530, 15000., 200., 2920.

531, 12200., 3200., 2920.

532, 15000., 3200., 2920.

533, 15000., 3200., 2700.

534, 12200., 3200., 2700.

535, 15000., 3000., 2920.

536, 15000., 3000., 2700.

537, 12200., 3000., 2700.

538, 12200., 3000., 2920.

539, 15000., 6000., 2920.

540, 12200., 6000., 2920.

541, 12200., 6000., 2700.

542, 15000., 6000., 2700.

543, 12200., 6200., 2920.

544, 12200., 6200., 2700.

545, 15000., 6200., 2700.

546, 15000., 6200., 2920.

547, 15000., 9000., 2920.

548, 12200., 9000., 2920.

549, 12200., 9000., 2700.

550, 15000., 9000., 2700.

551, 12200., 9200., 2920.

552, 12200., 9200., 2700.

553, 15000., 9200., 2700.

554, 15000., 9200., 2920.

555, 15000., 12000., 2920.

556, 12200., 12000., 2920.

557, 12200., 12000., 2700.

558, 15000., 12000., 2700.

559, 12200., 12200., 2920.

560, 12200., 12200., 2700.

561, 15000., 12200., 2700.

562, 15000., 12200., 2920.

563, 12200., 15200., 2920.

564, 15000., 15200., 2920.

565, 15000., 15000., 2920.

566, 12200., 15000., 2920.

567, 15000., 15200., 3000.

568, 15000., 15000., 3000.

569, 15000., 12200., 3000.

570, 15000., 12000., 3000.

571, 15000., 9200., 3000.

572, 15000., 9000., 3000.

573, 15000., 6200., 3000.

574, 15000., 6000., 3000.

575, 15000., 3200., 3000.

576, 15000., 3000., 3000.

577, 15000., 200., 3000.

578, 15000., 0., 3000.

579, 12200., 0., 3000.

580, 12200., 15200., 3000.

581, 12200., 15000., 3000.

582, 12200., 12200., 3000.

583, 12200., 12000., 3000.

584, 12200., 9200., 3000.

585, 12200., 9000., 3000.

586, 12200., 6200., 3000.

587, 12200., 6000., 3000.

588, 12200., 3200., 3000.

589, 12200., 3000., 3000.

590, 12200., 200., 3000.

591, 15000., 0., 5920.

592, 12200., 0., 5920.

593, 12200., 0., 5700.

594, 15000., 0., 5700.

595, 12200., 200., 5920.

596, 12200., 200., 5700.

597, 15000., 200., 5700.

598, 15000., 200., 5920.

599, 12200., 3200., 5920.

600, 15000., 3200., 5920.

601, 15000., 3200., 5700.

602, 12200., 3200., 5700.

603, 15000., 3000., 5920.

604, 15000., 3000., 5700.

605, 12200., 3000., 5700.

606, 12200., 3000., 5920.

607, 15000., 6000., 5920.

608, 12200., 6000., 5920.

609, 12200., 6000., 5700.

610, 15000., 6000., 5700.

611, 12200., 6200., 5920.

612, 12200., 6200., 5700.

613, 15000., 6200., 5700.

614, 15000., 6200., 5920.

615, 15000., 9000., 5920.

616, 12200., 9000., 5920.

617, 12200., 9000., 5700.

618, 15000., 9000., 5700.

619, 12200., 9200., 5920.

620, 12200., 9200., 5700.

621, 15000., 9200., 5700.

622, 15000., 9200., 5920.

623, 15000., 12000., 5920.

624, 12200., 12000., 5920.

625, 12200., 12000., 5700.

626, 15000., 12000., 5700.

627, 12200., 12200., 5920.

628, 12200., 12200., 5700.

629, 15000., 12200., 5700.

630, 15000., 12200., 5920.

631, 12200., 15200., 5920.

632, 15000., 15200., 5920.

633, 15000., 15000., 5920.

634, 12200., 15000., 5920.

635, 15000., 15200., 6000.

636, 15000., 15000., 6000.

637, 15000., 12200., 6000.

638, 15000., 12000., 6000.

639, 15000., 9200., 6000.

640, 15000., 9000., 6000.

641, 15000., 6200., 6000.

642, 15000., 6000., 6000.

643, 15000., 3200., 6000.

644, 15000., 3000., 6000.

645, 15000., 200., 6000.

646, 15000., 0., 6000.

647, 12200., 0., 6000.

648, 12200., 15200., 6000.

649, 12200., 15000., 6000.

650, 12200., 12200., 6000.

651, 12200., 12000., 6000.

652, 12200., 9200., 6000.

653, 12200., 9000., 6000.

654, 12200., 6200., 6000.

655, 12200., 6000., 6000.

656, 12200., 3200., 6000.

657, 12200., 3000., 6000.

658, 12200., 200., 6000.

659, 15200., 15200., 8920.

660, 18000., 15200., 8920.

661, 18000., 15200., 8700.

662, 15200., 15200., 8700.

663, 18000., 15000., 8920.

664, 18000., 15000., 8700.

665, 15200., 15000., 8700.

666, 15200., 15000., 8920.

667, 18000., 12000., 8920.

668, 15200., 12000., 8920.

669, 15200., 12000., 8700.

670, 18000., 12000., 8700.

671, 15200., 12200., 8920.

672, 15200., 12200., 8700.

673, 18000., 12200., 8700.

674, 18000., 12200., 8920.

675, 15200., 0., 8700.

676, 15200., 200., 8700.

677, 18000., 200., 8700.

678, 18000., 0., 8700.

679, 15200., 0., 8920.

680, 15200., 200., 8920.

681, 18000., 200., 8920.

682, 18000., 0., 8920.

683, 18000., 3000., 8920.

684, 15200., 3000., 8920.

685, 15200., 3000., 8700.

686, 18000., 3000., 8700.

687, 15200., 3200., 8920.

688, 15200., 3200., 8700.

689, 18000., 3200., 8700.

690, 18000., 3200., 8920.

691, 18000., 6000., 8920.

692, 15200., 6000., 8920.

693, 15200., 6000., 8700.

694, 18000., 6000., 8700.

695, 15200., 6200., 8920.

696, 15200., 6200., 8700.

697, 18000., 6200., 8700.

698, 18000., 6200., 8920.

699, 18000., 9000., 8920.

700, 15200., 9000., 8920.

701, 15200., 9000., 8700.

702, 18000., 9000., 8700.

703, 15200., 9200., 8920.

704, 15200., 9200., 8700.

705, 18000., 9200., 8700.

706, 18000., 9200., 8920.

707, 15200., 15200., 5920.

708, 18000., 15200., 5920.

709, 18000., 15200., 5700.

710, 15200., 15200., 5700.

711, 18000., 15000., 5920.

712, 18000., 15000., 5700.

713, 15200., 15000., 5700.

714, 15200., 15000., 5920.

715, 18000., 12000., 5920.

716, 15200., 12000., 5920.

717, 15200., 12000., 5700.

718, 18000., 12000., 5700.

719, 15200., 12200., 5920.

720, 15200., 12200., 5700.

721, 18000., 12200., 5700.

722, 18000., 12200., 5920.

723, 18000., 6000., 5920.

724, 15200., 6000., 5920.

725, 15200., 6000., 5700.

726, 18000., 6000., 5700.

727, 15200., 6200., 5920.

728, 15200., 6200., 5700.

729, 18000., 6200., 5700.

730, 18000., 6200., 5920.

731, 18000., 9000., 5920.

732, 15200., 9000., 5920.

733, 15200., 9000., 5700.

734, 18000., 9000., 5700.

735, 15200., 9200., 5920.

736, 15200., 9200., 5700.

737, 18000., 9200., 5700.

738, 18000., 9200., 5920.

739, 15200., 3200., 5920.

740, 18000., 3200., 5920.

741, 18000., 3200., 5700.

742, 15200., 3200., 5700.

743, 18000., 3000., 5920.

744, 18000., 3000., 5700.

745, 15200., 3000., 5700.

746, 15200., 3000., 5920.

747, 18000., 0., 5920.

748, 15200., 0., 5920.

749, 15200., 200., 5920.

750, 18000., 200., 5920.

751, 15200., 0., 6000.

752, 15200., 200., 6000.

753, 15200., 3000., 6000.

754, 15200., 3200., 6000.

755, 15200., 6000., 6000.

756, 15200., 6200., 6000.

757, 15200., 9000., 6000.

758, 15200., 9200., 6000.

759, 15200., 12000., 6000.

760, 15200., 12200., 6000.

761, 15200., 15000., 6000.

762, 15200., 15200., 6000.

763, 18000., 15200., 6000.

764, 18000., 0., 6000.

765, 18000., 200., 6000.

766, 18000., 3000., 6000.

767, 18000., 3200., 6000.

768, 18000., 6000., 6000.

769, 18000., 6200., 6000.

770, 18000., 9000., 6000.

771, 18000., 9200., 6000.

772, 18000., 12000., 6000.

773, 18000., 12200., 6000.

774, 18000., 15000., 6000.

775, 18000., 0., 2920.

776, 15200., 0., 2920.

777, 15200., 0., 2700.

778, 18000., 0., 2700.

779, 15200., 200., 2920.

780, 15200., 200., 2700.

781, 18000., 200., 2700.

782, 18000., 200., 2920.

783, 15200., 3200., 2920.

784, 18000., 3200., 2920.

785, 18000., 3200., 2700.

786, 15200., 3200., 2700.

787, 18000., 3000., 2920.

788, 18000., 3000., 2700.

789, 15200., 3000., 2700.

790, 15200., 3000., 2920.

791, 18000., 6000., 2920.

792, 15200., 6000., 2920.

793, 15200., 6000., 2700.

794, 18000., 6000., 2700.

795, 15200., 6200., 2920.

796, 15200., 6200., 2700.

797, 18000., 6200., 2700.

798, 18000., 6200., 2920.

799, 18000., 9000., 2920.

800, 15200., 9000., 2920.

801, 15200., 9000., 2700.

802, 18000., 9000., 2700.

803, 15200., 9200., 2920.

804, 15200., 9200., 2700.

805, 18000., 9200., 2700.

806, 18000., 9200., 2920.

807, 18000., 12000., 2920.

808, 15200., 12000., 2920.

809, 15200., 12000., 2700.

810, 18000., 12000., 2700.

811, 15200., 12200., 2920.

812, 15200., 12200., 2700.

813, 18000., 12200., 2700.

814, 18000., 12200., 2920.

815, 15200., 15200., 2920.

816, 18000., 15200., 2920.

817, 18000., 15000., 2920.

818, 15200., 15000., 2920.

819, 18000., 15200., 3000.

820, 18000., 15000., 3000.

821, 18000., 12200., 3000.

822, 18000., 12000., 3000.

823, 18000., 9200., 3000.

824, 18000., 9000., 3000.

825, 18000., 6200., 3000.

826, 18000., 6000., 3000.

827, 18000., 3200., 3000.

828, 18000., 3000., 3000.

829, 18000., 200., 3000.

830, 18000., 0., 3000.

831, 15200., 0., 3000.

832, 15200., 15200., 3000.

833, 15200., 15000., 3000.

834, 15200., 12200., 3000.

835, 15200., 12000., 3000.

836, 15200., 9200., 3000.

837, 15200., 9000., 3000.

838, 15200., 6200., 3000.

839, 15200., 6000., 3000.

840, 15200., 3200., 3000.

841, 15200., 3000., 3000.

842, 15200., 200., 3000.

843, 18200., 6200., 2920.

844, 21000., 6200., 2920.

845, 21000., 6200., 2700.

846, 18200., 6200., 2700.

847, 21000., 6000., 2920.

848, 21000., 6000., 2700.

849, 18200., 6000., 2700.

850, 18200., 6000., 2920.

851, 21000., 3000., 2920.

852, 18200., 3000., 2920.

853, 18200., 3000., 2700.

854, 21000., 3000., 2700.

855, 18200., 3200., 2920.

856, 18200., 3200., 2700.

857, 21000., 3200., 2700.

858, 21000., 3200., 2920.

859, 21000., 15000., 2920.

860, 18200., 15000., 2920.

861, 18200., 15000., 2700.

862, 21000., 15000., 2700.

863, 18200., 15200., 2920.

864, 18200., 15200., 2700.

865, 21000., 15200., 2700.

866, 21000., 15200., 2920.

867, 18200., 12200., 2920.

868, 21000., 12200., 2920.

869, 21000., 12200., 2700.

870, 18200., 12200., 2700.

871, 21000., 12000., 2920.

872, 21000., 12000., 2700.

873, 18200., 12000., 2700.

874, 18200., 12000., 2920.

875, 21000., 0., 2920.

876, 18200., 0., 2920.

877, 18200., 200., 2920.

878, 21000., 200., 2920.

879, 21000., 9000., 2920.

880, 18200., 9000., 2920.

881, 18200., 9200., 2920.

882, 21000., 9200., 2920.

883, 18200., 0., 3000.

884, 18200., 200., 3000.

885, 18200., 3000., 3000.

886, 18200., 3200., 3000.

887, 18200., 6000., 3000.

888, 18200., 6200., 3000.

889, 18200., 9000., 3000.

890, 18200., 9200., 3000.

891, 18200., 12000., 3000.

892, 18200., 12200., 3000.

893, 18200., 15000., 3000.

894, 18200., 15200., 3000.

895, 21000., 15200., 3000.

896, 21000., 0., 3000.

897, 21000., 200., 3000.

898, 21000., 3000., 3000.

899, 21000., 3200., 3000.

900, 21000., 6000., 3000.

901, 21000., 6200., 3000.

902, 21000., 9000., 3000.

903, 21000., 9200., 3000.

904, 21000., 12000., 3000.

905, 21000., 12200., 3000.

906, 21000., 15000., 3000.

907, 21000., 9200., 2700.

908, 18200., 9200., 2700.

909, 21000., 9000., 2700.

910, 18200., 9000., 2700.

911, 21000., 0., 5920.

912, 18200., 0., 5920.

913, 18200., 0., 5700.

914, 21000., 0., 5700.

915, 18200., 200., 5920.

916, 18200., 200., 5700.

917, 21000., 200., 5700.

918, 21000., 200., 5920.

919, 18200., 3200., 5920.

920, 21000., 3200., 5920.

921, 21000., 3200., 5700.

922, 18200., 3200., 5700.

923, 21000., 3000., 5920.

924, 21000., 3000., 5700.

925, 18200., 3000., 5700.

926, 18200., 3000., 5920.

927, 21000., 12000., 5920.

928, 18200., 12000., 5920.

929, 18200., 12000., 5700.

930, 21000., 12000., 5700.

931, 18200., 12200., 5920.

932, 18200., 12200., 5700.

933, 21000., 12200., 5700.

934, 21000., 12200., 5920.

935, 21000., 15000., 5920.

936, 18200., 15000., 5920.

937, 18200., 15000., 5700.

938, 21000., 15000., 5700.

939, 18200., 15200., 5920.

940, 18200., 15200., 5700.

941, 21000., 15200., 5700.

942, 21000., 15200., 5920.

943, 18200., 9200., 5920.

944, 21000., 9200., 5920.

945, 21000., 9200., 5700.

946, 18200., 9200., 5700.

947, 21000., 9000., 5920.

948, 21000., 9000., 5700.

949, 18200., 9000., 5700.

950, 18200., 9000., 5920.

951, 18200., 6200., 5920.

952, 21000., 6200., 5920.

953, 21000., 6200., 5700.

954, 18200., 6200., 5700.

955, 21000., 6000., 5920.

956, 21000., 6000., 5700.

957, 18200., 6000., 5700.

958, 18200., 6000., 5920.

959, 18200., 15200., 8920.

960, 21000., 15200., 8920.

961, 21000., 15200., 8700.

962, 18200., 15200., 8700.

963, 21000., 15000., 8920.

964, 21000., 15000., 8700.

965, 18200., 15000., 8700.

966, 18200., 15000., 8920.

967, 21000., 12000., 8920.

968, 18200., 12000., 8920.

969, 18200., 12000., 8700.

970, 21000., 12000., 8700.

971, 18200., 12200., 8920.

972, 18200., 12200., 8700.

973, 21000., 12200., 8700.

974, 21000., 12200., 8920.

975, 21000., 200., 8700.

976, 21000., 0., 8700.

977, 18200., 0., 8700.

978, 18200., 200., 8700.

979, 21000., 200., 8920.

980, 21000., 0., 8920.

981, 18200., 0., 8920.

982, 18200., 200., 8920.

983, 21000., 3000., 8920.

984, 18200., 3000., 8920.

985, 18200., 3000., 8700.

986, 21000., 3000., 8700.

987, 18200., 3200., 8920.

988, 18200., 3200., 8700.

989, 21000., 3200., 8700.

990, 21000., 3200., 8920.

991, 21000., 6000., 8920.

992, 18200., 6000., 8920.

993, 18200., 6000., 8700.

994, 21000., 6000., 8700.

995, 18200., 6200., 8920.

996, 18200., 6200., 8700.

997, 21000., 6200., 8700.

998, 21000., 6200., 8920.

999, 21000., 9000., 8920.

1000, 18200., 9000., 8920.

1001, 18200., 9000., 8700.

1002, 21000., 9000., 8700.

1003, 18200., 9200., 8920.

1004, 18200., 9200., 8700.

1005, 21000., 9200., 8700.

1006, 21000., 9200., 8920.

1007, 21200., 3200., 2920.

1008, 21200., 3200., 2700.

1009, 21200., 3000., 2700.

1010, 21200., 3000., 2920.

1011, 21200., 6200., 2920.

1012, 21200., 6200., 2700.

1013, 21200., 6000., 2700.

1014, 21200., 6000., 2920.

1015, 21200., 15200., 2920.

1016, 21200., 15000., 2920.

1017, 21200., 15000., 2700.

1018, 21200., 15200., 2700.

1019, 21200., 12200., 2920.

1020, 21200., 12200., 2700.

1021, 21200., 12000., 2700.

1022, 21200., 12000., 2920.

1023, 21200., 9200., 2920.

1024, 21200., 9200., 2700.

1025, 21200., 9000., 2700.

1026, 21200., 9000., 2920.

1027, 21200., 200., 2920.

1028, 21200., 0., 2920.

1029, 21200., 15200., 3000.

1030, 21200., 15000., 3000.

1031, 21200., 12200., 3000.

1032, 21200., 12000., 3000.

1033, 21200., 9200., 3000.

1034, 21200., 9000., 3000.

1035, 21200., 6200., 3000.

1036, 21200., 6000., 3000.

1037, 21200., 3200., 3000.

1038, 21200., 3000., 3000.

1039, 21200., 200., 3000.

1040, 21200., 0., 3000.

1041, 21200., 200., 5920.

1042, 21200., 200., 5700.

1043, 21200., 0., 5700.

1044, 21200., 0., 5920.

1045, 21200., 3000., 5920.

1046, 21200., 3000., 5700.

1047, 21200., 3200., 5920.

1048, 21200., 3200., 5700.

1049, 21200., 15000., 5920.

1050, 21200., 15000., 5700.

1051, 21200., 15200., 5920.

1052, 21200., 15200., 5700.

1053, 21200., 12200., 5920.

1054, 21200., 12200., 5700.

1055, 21200., 12000., 5700.

1056, 21200., 12000., 5920.

1057, 21200., 9200., 5920.

1058, 21200., 9200., 5700.

1059, 21200., 9000., 5700.

1060, 21200., 9000., 5920.

1061, 21200., 6200., 5920.

1062, 21200., 6200., 5700.

1063, 21200., 6000., 5700.

1064, 21200., 6000., 5920.

1065, 21200., 200., 8920.

1066, 21200., 200., 8700.

1067, 21200., 0., 8700.

1068, 21200., 0., 8920.

1069, 21200., 3000., 8920.

1070, 21200., 3000., 8700.

1071, 21200., 3200., 8920.

1072, 21200., 3200., 8700.

1073, 21200., 9200., 8920.

1074, 21200., 9200., 8700.

1075, 21200., 9000., 8700.

1076, 21200., 9000., 8920.

1077, 21200., 6200., 8920.

1078, 21200., 6200., 8700.

1079, 21200., 6000., 8700.

1080, 21200., 6000., 8920.

1081, 21200., 15000., 8920.

1082, 21200., 15000., 8700.

1083, 21200., 15200., 8920.

1084, 21200., 15200., 8700.

1085, 21200., 12000., 8920.

1086, 21200., 12200., 8920.

1087, 21200., 0., 9000.

1088, 21200., 15200., 9000.

1089, 21200., 15000., 9000.

1090, 21200., 12200., 9000.

1091, 21200., 12000., 9000.

1092, 21200., 9200., 9000.

1093, 21200., 9000., 9000.

1094, 21200., 6200., 9000.

1095, 21200., 6000., 9000.

1096, 21200., 3200., 9000.

1097, 21200., 3000., 9000.

1098, 21200., 200., 9000.

1099, 21000., 3000., 9000.

1100, 21000., 200., 9000.

1101, 21200., 3000., 6000.

1102, 21200., 200., 6000.

1103, 21000., 200., 6000.

1104, 21000., 3000., 6000.

1105, 21000., 200., 2700.

1106, 21200., 200., 2700.

1107, 21000., 6000., 6000.

1108, 21000., 3200., 6000.

1109, 21200., 6000., 6000.

1110, 21200., 3200., 6000.

1111, 21200., 6200., 6000.

1112, 21200., 6000., 0.

1113, 21200., 6200., 0.

1114, 21000., 6000., 9000.

1115, 21000., 6200., 9000.

1116, 21000., 6200., 6000.

1117, 21000., 6200., 0.

1118, 21000., 6000., 0.

1119, 21000., 9000., 9000.

1120, 21000., 9000., 6000.

1121, 21200., 9000., 6000.

1122, 21000., 12000., 6000.

1123, 21000., 9200., 6000.

1124, 21200., 12000., 6000.

1125, 21200., 9200., 6000.

1126, 21200., 12000., 8700.

1127, 21200., 12200., 8700.

1128, 21200., 12200., 6000.

1129, 21200., 12000., 0.

1130, 21200., 12200., 0.

1131, 21000., 12200., 9000.

1132, 21000., 12000., 9000.

1133, 21000., 12200., 6000.

1134, 21000., 12200., 0.

1135, 21000., 12000., 0.

1136, 21000., 15000., 9000.

1137, 21200., 15000., 6000.

1138, 21000., 15000., 6000.

1139, 21200., 15200., 6000.

1140, 21200., 15000., 0.

1141, 21200., 15200., 0.

1142, 21000., 15200., 9000.

1143, 21000., 15200., 6000.

1144, 21000., 15200., 0.

1145, 21000., 15000., 0.

1146, 21200., 200., 0.

1147, 21000., 200., 0.

1148, 21200., 0., 6000.

1149, 21200., 0., 2700.

1150, 21200., 0., 0.

1151, 21000., 0., 0.

1152, 21000., 0., 9000.

1153, 21000., 0., 6000.

1154, 21000., 0., 2700.

1155, 21000., 3000., 0.

1156, 21000., 3200., 0.

1157, 21000., 3200., 9000.

1158, 21200., 3200., 0.

1159, 21200., 3000., 0.

1160, 21200., 9000., 0.

1161, 21200., 9200., 0.

1162, 21000., 9200., 9000.

1163, 21000., 9200., 0.

1164, 21000., 9000., 0.

1165, 18200., 200., 2700.

1166, 18200., 200., 6000.

1167, 18200., 3000., 6000.

1168, 18000., 200., 5700.

1169, 18200., 200., 0.

1170, 18000., 200., 0.

1171, 18000., 200., 9000.

1172, 18200., 200., 9000.

1173, 18000., 0., 0.

1174, 18000., 0., 5700.

1175, 18000., 0., 9000.

1176, 18200., 0., 9000.

1177, 18200., 0., 0.

1178, 18200., 0., 2700.

1179, 18200., 0., 6000.

1180, 18200., 3200., 9000.

1181, 18200., 6000., 9000.

1182, 18000., 3200., 9000.

1183, 18000., 6000., 9000.

1184, 18200., 3200., 6000.

1185, 18200., 6000., 6000.

1186, 18200., 6200., 6000.

1187, 18200., 9000., 6000.

1188, 18200., 6200., 9000.

1189, 18200., 6000., 0.

1190, 18200., 6200., 0.

1191, 18000., 6200., 9000.

1192, 18000., 6200., 0.

1193, 18000., 6000., 0.

1194, 18200., 12000., 6000.

1195, 18200., 9200., 6000.

1196, 18200., 9200., 0.

1197, 18200., 9000., 0.

1198, 18200., 9200., 9000.

1199, 18200., 9000., 9000.

1200, 18000., 9000., 0.

1201, 18000., 9200., 0.

1202, 18000., 9000., 9000.

1203, 18000., 9200., 9000.

1204, 18000., 12200., 9000.

1205, 18000., 15000., 9000.

1206, 18200., 15000., 9000.

1207, 18200., 12200., 9000.

1208, 18200., 15000., 6000.

1209, 18200., 12200., 6000.

1210, 18000., 15000., 2700.

1211, 18200., 3000., 9000.

1212, 18000., 3000., 9000.

1213, 18000., 12000., 9000.

1214, 18200., 12000., 9000.

1215, 18200., 12200., 0.

1216, 18200., 12000., 0.

1217, 18000., 12000., 0.

1218, 18000., 12200., 0.

1219, 18000., 15000., 0.

1220, 18200., 15000., 0.

1221, 18200., 15200., 0.

1222, 18200., 15200., 6000.

1223, 18200., 15200., 9000.

1224, 18000., 15200., 9000.

1225, 18000., 15200., 0.

1226, 18000., 15200., 2700.

1227, 15200., 200., 5700.

1228, 15200., 3000., 9000.

1229, 15200., 3200., 9000.

1230, 15000., 3200., 9000.

1231, 15000., 3000., 9000.

1232, 15200., 3200., 0.

1233, 15000., 3200., 0.

1234, 15000., 3000., 0.

1235, 15200., 3000., 0.

1236, 15000., 3000., 8700.

1237, 15000., 3200., 8700.

1238, 15200., 6000., 9000.

1239, 15200., 9000., 9000.

1240, 15200., 6200., 9000.

1241, 15200., 12000., 9000.

1242, 15200., 12200., 9000.

1243, 15200., 12000., 0.

1244, 15200., 12200., 0.

1245, 15000., 12200., 0.

1246, 15000., 12000., 0.

1247, 15000., 15000., 2700.

1248, 15200., 15000., 2700.

1249, 15200., 15000., 9000.

1250, 15000., 15000., 8700.

1251, 15000., 15000., 5700.

1252, 15000., 15000., 0.

1253, 15200., 15000., 0.

1254, 15000., 15200., 8700.

1255, 15000., 15200., 5700.

1256, 15000., 15200., 2700.

1257, 15000., 15200., 0.

1258, 15200., 15200., 0.

1259, 15200., 15200., 9000.

1260, 15200., 15200., 2700.

1261, 15200., 200., 0.

1262, 15000., 200., 0.

1263, 15000., 200., 8700.

1264, 15000., 200., 9000.

1265, 15200., 200., 9000.

1266, 15000., 0., 0.

1267, 15000., 0., 9000.

1268, 15200., 0., 9000.

1269, 15200., 0., 0.

1270, 15200., 0., 5700.

1271, 15000., 6200., 0.

1272, 15000., 6000., 0.

1273, 15200., 6000., 0.

1274, 15200., 6200., 0.

1275, 15200., 9200., 9000.

1276, 12000., 200., 3000.

1277, 12000., 3000., 3000.

1278, 12200., 200., 0.

1279, 12000., 200., 0.

1280, 12000., 200., 6000.

1281, 12000., 0., 0.

1282, 12000., 0., 3000.

1283, 12000., 0., 6000.

1284, 12200., 0., 0.

1285, 12000., 3200., 6000.

1286, 12000., 6000., 6000.

1287, 12000., 6000., 3000.

1288, 12000., 3200., 3000.

1289, 12000., 6200., 9000.

1290, 12000., 9000., 9000.

1291, 12000., 6200., 6000.

1292, 12000., 9000., 6000.

1293, 12000., 6200., 3000.

1294, 12000., 9000., 3000.

1295, 12000., 9200., 9000.

1296, 12000., 12000., 9000.

1297, 12000., 9200., 6000.

1298, 12000., 12000., 6000.

1299, 12000., 12000., 3000.

1300, 12000., 9200., 3000.

1301, 12200., 15000., 8700.

1302, 12000., 12200., 9000.

1303, 12000., 15000., 9000.

1304, 12200., 15000., 5700.

1305, 12000., 12200., 6000.

1306, 12000., 15000., 6000.

1307, 12000., 12200., 3000.

1308, 12000., 15000., 3000.

1309, 12200., 15000., 2700.

1310, 12000., 3000., 6000.

1311, 12000., 3200., 0.

1312, 12000., 3000., 0.

1313, 12200., 3000., 0.

1314, 12200., 3200., 0.

1315, 12000., 6000., 0.

1316, 12200., 6000., 0.

1317, 12000., 6000., 9000.

1318, 12200., 6200., 0.

1319, 12000., 6200., 0.

1320, 12000., 9200., 0.

1321, 12000., 9000., 0.

1322, 12200., 9000., 0.

1323, 12200., 9200., 0.

1324, 12000., 12000., 0.

1325, 12000., 12200., 0.

1326, 12200., 12200., 0.

1327, 12200., 12000., 0.

1328, 12000., 15000., 0.

1329, 12200., 15000., 0.

1330, 12000., 15200., 9000.

1331, 12000., 15200., 6000.

1332, 12000., 15200., 3000.

1333, 12000., 15200., 0.

1334, 12200., 15200., 0.

1335, 12200., 15200., 8700.

1336, 12200., 15200., 5700.

1337, 12200., 15200., 2700.

1338, 9200., 12200., 3000.

1339, 9200., 15000., 3000.

1340, 9000., 15000., 2700.

1341, 9200., 12200., 6000.

1342, 9200., 15000., 6000.

1343, 9000., 12200., 6000.

1344, 9000., 15000., 6000.

1345, 9000., 12000., 6000.

1346, 9000., 12200., 0.

1347, 9000., 12000., 0.

1348, 9200., 12200., 9000.

1349, 9200., 12000., 9000.

1350, 9200., 12000., 6000.

1351, 9200., 12000., 3000.

1352, 9200., 12000., 0.

1353, 9200., 12200., 0.

1354, 9200., 9200., 3000.

1355, 9200., 9200., 6000.

1356, 9000., 9200., 6000.

1357, 9000., 9000., 6000.

1358, 9000., 9200., 0.

1359, 9000., 9000., 0.

1360, 9200., 9200., 9000.

1361, 9200., 9000., 9000.

1362, 9200., 9000., 6000.

1363, 9200., 9000., 3000.

1364, 9200., 9000., 0.

1365, 9200., 9200., 0.

1366, 9200., 6200., 3000.

1367, 9000., 6200., 6000.

1368, 9200., 6200., 6000.

1369, 9000., 6000., 0.

1370, 9200., 6000., 0.

1371, 9200., 6000., 3000.

1372, 9200., 6000., 6000.

1373, 9000., 6000., 6000.

1374, 9200., 6000., 9000.

1375, 9200., 6200., 0.

1376, 9000., 6200., 0.

1377, 9200., 6200., 9000.

1378, 9200., 3200., 3000.

1379, 9200., 3200., 6000.

1380, 9200., 3000., 6000.

1381, 9200., 3000., 3000.

1382, 9200., 3000., 0.

1383, 9200., 3200., 0.

1384, 9000., 3000., 6000.

1385, 9000., 3200., 6000.

1386, 9000., 3200., 0.

1387, 9000., 3000., 0.

1388, 9200., 200., 3000.

1389, 9200., 200., 6000.

1390, 9200., 0., 6000.

1391, 9200., 0., 3000.

1392, 9200., 0., 0.

1393, 9200., 200., 0.

1394, 9000., 0., 6000.

1395, 9000., 200., 6000.

1396, 9000., 200., 0.

1397, 9000., 0., 0.

1398, 9000., 15000., 8700.

1399, 9200., 15000., 9000.

1400, 9000., 15000., 0.

1401, 9200., 15000., 0.

1402, 9000., 15200., 8700.

1403, 9000., 15200., 6000.

1404, 9000., 15200., 2700.

1405, 9000., 15200., 0.

1406, 9200., 15200., 0.

1407, 9200., 15200., 9000.

1408, 9200., 15200., 6000.

1409, 9200., 15200., 3000.

1410, 0., 12000., 2700.

1411, 0., 12200., 2700.

1412, 0., 12200., 0.

1413, 0., 12000., 0.

1414, 200., 12200., 2700.

1415, 200., 12200., 0.

1416, 200., 12000., 0.

1417, 200., 12000., 2700.

1418, 0., 15200., 2700.

1419, 0., 15000., 2700.

1420, 0., 9200., 2700.

1421, 0., 9000., 2700.

1422, 0., 6200., 2700.

1423, 0., 6000., 2700.

1424, 0., 3200., 2700.

1425, 0., 3000., 2700.

1426, 0., 200., 2700.

1427, 0., 0., 2700.

1428, 0., 15000., 3000.

1429, 0., 12200., 3000.

1430, 0., 12000., 3000.

1431, 0., 9200., 3000.

1432, 0., 9000., 3000.

1433, 0., 6200., 3000.

1434, 0., 6000., 3000.

1435, 0., 3200., 3000.

1436, 0., 3000., 3000.

1437, 0., 200., 3000.

1438, 0., 0., 3000.

1439, 0., 15200., 3000.

1440, 200., 15000., 2700.

1441, 200., 15000., 2920.

1442, 200., 15200., 2920.

1443, 200., 15200., 2700.

1444, 200., 12200., 2920.

1445, 200., 12000., 2920.

1446, 200., 9200., 2920.

1447, 200., 9200., 2700.

1448, 200., 9000., 2920.

1449, 200., 9000., 2700.

1450, 200., 6200., 2920.

1451, 200., 6200., 2700.

1452, 200., 6000., 2920.

1453, 200., 6000., 2700.

1454, 200., 3200., 2920.

1455, 200., 3200., 2700.

1456, 200., 3000., 2920.

1457, 200., 3000., 2700.

1458, 200., 200., 2920.

1459, 200., 200., 2700.

1460, 200., 0., 2920.

1461, 200., 0., 2700.

1462, 200., 200., 3000.

1463, 200., 3000., 3000.

1464, 200., 3200., 3000.

1465, 200., 6000., 3000.

1466, 200., 6200., 3000.

1467, 200., 9000., 3000.

1468, 200., 9200., 3000.

1469, 200., 12000., 3000.

1470, 200., 12200., 3000.

1471, 200., 15000., 3000.

1472, 200., 15200., 3000.

1473, 200., 0., 3000.

1474, 0., 15000., 0.

1475, 200., 15000., 0.

1476, 0., 15200., 0.

1477, 200., 15200., 0.

1478, 200., 200., 0.

1479, 0., 200., 0.

1480, 200., 0., 0.

1481, 0., 0., 0.

1482, 200., 3200., 0.

1483, 0., 3200., 0.

1484, 200., 3000., 0.

1485, 0., 3000., 0.

1486, 200., 6200., 0.

1487, 0., 6200., 0.

1488, 200., 6000., 0.

1489, 0., 6000., 0.

1490, 200., 15000., 5700.

1491, 0., 15000., 5700.

1492, 0., 15200., 5700.

1493, 200., 15200., 5700.

1494, 200., 12000., 5700.

1495, 0., 12000., 5700.

1496, 0., 12200., 5700.

1497, 200., 12200., 5700.

1498, 200., 9000., 5700.

1499, 0., 9000., 5700.

1500, 0., 9200., 5700.

1501, 200., 9200., 5700.

1502, 200., 6000., 5700.

1503, 0., 6000., 5700.

1504, 0., 6200., 5700.

1505, 200., 6200., 5700.

1506, 200., 3000., 5700.

1507, 0., 3000., 5700.

1508, 0., 3200., 5700.

1509, 200., 3200., 5700.

1510, 200., 200., 5700.

1511, 200., 0., 5700.

1512, 0., 0., 5700.

1513, 0., 200., 5700.

1514, 0., 15200., 6000.

1515, 0., 15000., 6000.

1516, 0., 15000., 8700.

1517, 0., 15200., 8700.

1518, 200., 15000., 6000.

1519, 200., 15000., 8700.

1520, 200., 15200., 8700.

1521, 200., 15200., 6000.

1522, 0., 0., 6000.

1523, 0., 200., 6000.

1524, 200., 200., 6000.

1525, 200., 0., 6000.

1526, 200., 3000., 6000.

1527, 0., 3000., 6000.

1528, 200., 3200., 6000.

1529, 0., 3200., 6000.

1530, 200., 6000., 6000.

1531, 0., 6000., 6000.

1532, 200., 6200., 6000.

1533, 0., 6200., 6000.

1534, 200., 9000., 6000.

1535, 0., 9000., 6000.

1536, 200., 9200., 6000.

1537, 0., 9200., 6000.

1538, 200., 12000., 6000.

1539, 0., 12000., 6000.

1540, 200., 12200., 6000.

1541, 0., 12200., 6000.

1542, 200., 200., 5920.

1543, 200., 0., 5920.

1544, 200., 12200., 5920.

1545, 200., 12000., 5920.

1546, 200., 9200., 5920.

1547, 200., 9000., 5920.

1548, 200., 6200., 5920.

1549, 200., 6000., 5920.

1550, 200., 3200., 5920.

1551, 200., 3000., 5920.

1552, 200., 15000., 5920.

1553, 200., 15200., 5920.

1554, 0., 200., 8700.

1555, 200., 200., 8700.

1556, 200., 0., 8700.

1557, 0., 0., 8700.

1558, 0., 3200., 8700.

1559, 200., 3200., 8700.

1560, 200., 3000., 8700.

1561, 0., 3000., 8700.

1562, 0., 6200., 8700.

1563, 200., 6200., 8700.

1564, 200., 6000., 8700.

1565, 0., 6000., 8700.

1566, 0., 9200., 8700.

1567, 200., 9200., 8700.

1568, 200., 9000., 8700.

1569, 0., 9000., 8700.

1570, 200., 9000., 0.

1571, 0., 9000., 0.

1572, 0., 9200., 0.

1573, 200., 9200., 0.

1574, 0., 12200., 8700.

1575, 200., 12200., 8700.

1576, 200., 12000., 8700.

1577, 0., 12000., 8700.

1578, 3000., 9000., 2920.

1579, 3000., 9000., 2700.

1580, 3000., 9200., 2700.

1581, 3000., 9200., 2920.

1582, 3000., 12000., 2920.

1583, 3000., 12000., 2700.

1584, 3000., 12200., 2700.

1585, 3000., 12200., 2920.

1586, 3000., 6200., 2920.

1587, 3000., 6200., 2700.

1588, 3000., 6000., 2920.

1589, 3000., 6000., 2700.

1590, 3000., 15200., 2920.

1591, 3000., 15000., 2920.

1592, 3000., 15200., 2700.

1593, 3000., 15000., 2700.

1594, 3000., 0., 2920.

1595, 3000., 0., 2700.

1596, 3000., 200., 2700.

1597, 3000., 200., 2920.

1598, 3000., 3200., 2920.

1599, 3000., 3000., 2920.

1600, 3000., 15200., 3000.

1601, 3000., 0., 3000.

1602, 3000., 200., 3000.

1603, 3000., 3000., 3000.

1604, 3000., 3200., 3000.

1605, 3000., 6000., 3000.

1606, 3000., 6200., 3000.

1607, 3000., 9000., 3000.

1608, 3000., 9200., 3000.

1609, 3000., 12000., 3000.

1610, 3000., 12200., 3000.

1611, 3000., 15000., 3000.

1612, 3000., 15000., 5920.

1613, 3000., 15000., 5700.

1614, 3000., 15200., 5700.

1615, 3000., 15200., 5920.

1616, 3000., 12000., 5920.

1617, 3000., 12200., 5920.

1618, 3000., 12200., 5700.

1619, 3000., 12000., 5700.

1620, 3000., 0., 5920.

1621, 3000., 0., 5700.

1622, 3000., 200., 5700.

1623, 3000., 200., 5920.

1624, 3000., 3000., 5920.

1625, 3000., 3200., 5920.

1626, 3000., 3200., 5700.

1627, 3000., 3000., 5700.

1628, 3000., 6000., 5920.

1629, 3000., 6200., 5920.

1630, 3000., 6200., 5700.

1631, 3000., 6000., 5700.

1632, 3000., 9000., 5920.

1633, 3000., 9200., 5920.

1634, 3000., 9200., 5700.

1635, 3000., 9000., 5700.

1636, 3000., 9000., 8920.

1637, 200., 9000., 8920.

1638, 3000., 9000., 8700.

1639, 200., 9200., 8920.

1640, 3000., 9200., 8700.

1641, 3000., 9200., 8920.

1642, 3000., 12000., 8920.

1643, 3000., 12200., 8920.

1644, 3000., 12200., 8700.

1645, 3000., 12000., 8700.

1646, 200., 12200., 8920.

1647, 200., 12000., 8920.

1648, 200., 200., 8920.

1649, 200., 0., 8920.

1650, 3000., 0., 8920.

1651, 3000., 0., 8700.

1652, 3000., 200., 8700.

1653, 3000., 200., 8920.

1654, 3000., 3000., 8920.

1655, 3000., 3200., 8920.

1656, 3000., 3200., 8700.

1657, 3000., 3000., 8700.

1658, 200., 3200., 8920.

1659, 200., 3000., 8920.

1660, 3000., 6000., 8920.

1661, 3000., 6200., 8920.

1662, 3000., 6200., 8700.

1663, 3000., 6000., 8700.

1664, 200., 6200., 8920.

1665, 200., 6000., 8920.

1666, 3000., 0., 9000.

1667, 3000., 200., 9000.

1668, 3000., 6200., 9000.

1669, 3000., 9000., 9000.

1670, 3000., 6000., 9000.

1671, 3000., 3200., 9000.

1672, 3000., 3000., 9000.

1673, 3000., 9200., 9000.

1674, 3000., 12000., 9000.

1675, 3000., 12200., 9000.

1676, 3000., 15000., 9000.

1677, 3000., 15000., 8920.

1678, 3000., 15200., 8920.

1679, 3000., 15200., 9000.

1680, 200., 0., 9000.

1681, 200., 15200., 9000.

1682, 200., 15200., 8920.

1683, 200., 15000., 8920.

1684, 3200., 12200., 8920.

1685, 6000., 12200., 8920.

1686, 6000., 12200., 8700.

1687, 3200., 12200., 8700.

1688, 6000., 12000., 8920.

1689, 6000., 12000., 8700.

1690, 3200., 12000., 8700.

1691, 3200., 12000., 8920.

1692, 6000., 9000., 8920.

1693, 3200., 9000., 8920.

1694, 3200., 9000., 8700.

1695, 6000., 9000., 8700.

1696, 3200., 9200., 8920.

1697, 3200., 9200., 8700.

1698, 6000., 9200., 8700.

1699, 6000., 9200., 8920.

1700, 6000., 6000., 8920.

1701, 3200., 6000., 8920.

1702, 3200., 6200., 8920.

1703, 6000., 6200., 8920.

1704, 6000., 15000., 8920.

1705, 3200., 15000., 8920.

1706, 3200., 15200., 8920.

1707, 6000., 15200., 8920.

1708, 3200., 6000., 9000.

1709, 3200., 6200., 9000.

1710, 3200., 9000., 9000.

1711, 3200., 9200., 9000.

1712, 3200., 12000., 9000.

1713, 3200., 12200., 9000.

1714, 3200., 15000., 9000.

1715, 3200., 15200., 9000.

1716, 6000., 15200., 9000.

1717, 6000., 6000., 9000.

1718, 6000., 6200., 9000.

1719, 6000., 9000., 9000.

1720, 6000., 9200., 9000.

1721, 6000., 12000., 9000.

1722, 6000., 12200., 9000.

1723, 6000., 15000., 9000.

1724, 3200., 6000., 8700.

1725, 3200., 6200., 8700.

1726, 6000., 6200., 8700.

1727, 6000., 6000., 8700.

1728, 6000., 0., 5920.

1729, 3200., 0., 5920.

1730, 3200., 0., 5700.

1731, 6000., 0., 5700.

1732, 3200., 200., 5920.

1733, 3200., 200., 5700.

1734, 6000., 200., 5700.

1735, 6000., 200., 5920.

1736, 3200., 3200., 5920.

1737, 6000., 3200., 5920.

1738, 6000., 3200., 5700.

1739, 3200., 3200., 5700.

1740, 6000., 3000., 5920.

1741, 6000., 3000., 5700.

1742, 3200., 3000., 5700.

1743, 3200., 3000., 5920.

1744, 6000., 12000., 5920.

1745, 3200., 12000., 5920.

1746, 3200., 12000., 5700.

1747, 6000., 12000., 5700.

1748, 3200., 12200., 5920.

1749, 3200., 12200., 5700.

1750, 6000., 12200., 5700.

1751, 6000., 12200., 5920.

1752, 6000., 15000., 5920.

1753, 3200., 15000., 5920.

1754, 3200., 15000., 5700.

1755, 6000., 15000., 5700.

1756, 3200., 15200., 5920.

1757, 3200., 15200., 5700.

1758, 6000., 15200., 5700.

1759, 6000., 15200., 5920.

1760, 3200., 9200., 5920.

1761, 6000., 9200., 5920.

1762, 6000., 9200., 5700.

1763, 3200., 9200., 5700.

1764, 6000., 9000., 5920.

1765, 6000., 9000., 5700.

1766, 3200., 9000., 5700.

1767, 3200., 9000., 5920.

1768, 3200., 6200., 5920.

1769, 6000., 6200., 5920.

1770, 6000., 6200., 5700.

1771, 3200., 6200., 5700.

1772, 6000., 6000., 5920.

1773, 6000., 6000., 5700.

1774, 3200., 6000., 5700.

1775, 3200., 6000., 5920.

1776, 6000., 0., 2920.

1777, 3200., 0., 2920.

1778, 3200., 0., 2700.

1779, 6000., 0., 2700.

1780, 3200., 200., 2920.

1781, 3200., 200., 2700.

1782, 6000., 200., 2700.

1783, 6000., 200., 2920.

1784, 3200., 3200., 2920.

1785, 6000., 3200., 2920.

1786, 6000., 3200., 2700.

1787, 3200., 3200., 2700.

1788, 6000., 3000., 2920.

1789, 6000., 3000., 2700.

1790, 3200., 3000., 2700.

1791, 3200., 3000., 2920.

1792, 6000., 9000., 2920.

1793, 3200., 9000., 2920.

1794, 3200., 9000., 2700.

1795, 6000., 9000., 2700.

1796, 3200., 9200., 2920.

1797, 3200., 9200., 2700.

1798, 6000., 9200., 2700.

1799, 6000., 9200., 2920.

1800, 6000., 12000., 2920.

1801, 3200., 12000., 2920.

1802, 3200., 12000., 2700.

1803, 6000., 12000., 2700.

1804, 3200., 12200., 2920.

1805, 3200., 12200., 2700.

1806, 6000., 12200., 2700.

1807, 6000., 12200., 2920.

1808, 6000., 15000., 2920.

1809, 3200., 15000., 2920.

1810, 3200., 15000., 2700.

1811, 6000., 15000., 2700.

1812, 3200., 15200., 2920.

1813, 3200., 15200., 2700.

1814, 6000., 15200., 2700.

1815, 6000., 15200., 2920.

1816, 3200., 6200., 2920.

1817, 6000., 6200., 2920.

1818, 6000., 6200., 2700.

1819, 3200., 6200., 2700.

1820, 6000., 6000., 2920.

1821, 6000., 6000., 2700.

1822, 3200., 6000., 2700.

1823, 3200., 6000., 2920.

1824, 6000., 15000., 8700.

1825, 6200., 15000., 8700.

1826, 6200., 15000., 6000.

1827, 6200., 12200., 6000.

1828, 6000., 12200., 6000.

1829, 6000., 15000., 6000.

1830, 6000., 15000., 0.

1831, 6200., 15000., 0.

1832, 6200., 15000., 2700.

1833, 6000., 15000., 3000.

1834, 6200., 15200., 0.

1835, 6200., 15200., 2700.

1836, 6200., 15200., 6000.

1837, 6200., 15200., 8700.

1838, 6000., 15200., 0.

1839, 6000., 15200., 3000.

1840, 6000., 15200., 6000.

1841, 6000., 15200., 8700.

1842, 6000., 9200., 6000.

1843, 6000., 12000., 6000.

1844, 6200., 12000., 6000.

1845, 6200., 9200., 6000.

1846, 6000., 9200., 3000.

1847, 6000., 12000., 3000.

1848, 6000., 6200., 3000.

1849, 6000., 9000., 3000.

1850, 6200., 6200., 6000.

1851, 6000., 6200., 6000.

1852, 6000., 9000., 6000.

1853, 6200., 9000., 6000.

1854, 6000., 9200., 0.

1855, 6000., 9000., 0.

1856, 6200., 9000., 0.

1857, 6200., 9200., 0.

1858, 6000., 3200., 6000.

1859, 6000., 6000., 6000.

1860, 6200., 6000., 6000.

1861, 6200., 3200., 6000.

1862, 6200., 6200., 0.

1863, 6000., 6200., 0.

1864, 6000., 6000., 3000.

1865, 6000., 6000., 0.

1866, 6200., 6000., 0.

1867, 6000., 200., 3000.

1868, 6000., 3000., 3000.

1869, 6200., 3000., 6000.

1870, 6200., 3000., 0.

1871, 6200., 3200., 0.

1872, 6000., 3000., 6000.

1873, 6000., 3200., 3000.

1874, 6000., 3200., 0.

1875, 6000., 3000., 0.

1876, 6200., 12000., 0.

1877, 6200., 12200., 0.

1878, 6000., 12200., 3000.

1879, 6000., 12200., 0.

1880, 6000., 12000., 0.

1881, 6000., 200., 6000.

1882, 6200., 200., 6000.

1883, 6200., 200., 0.

1884, 6000., 200., 0.

1885, 6000., 0., 0.

1886, 6000., 0., 3000.

1887, 6000., 0., 6000.

1888, 6200., 0., 6000.

1889, 6200., 0., 0.

1890, 3200., 15000., 3000.

1891, 3200., 12200., 3000.

1892, 3000., 12200., 6000.

1893, 3000., 15000., 6000.

1894, 3200., 15000., 6000.

1895, 3200., 12200., 6000.

1896, 3200., 12000., 6000.

1897, 3200., 12000., 3000.

1898, 3200., 12000., 0.

1899, 3200., 12200., 0.

1900, 3000., 12000., 6000.

1901, 3000., 12200., 0.

1902, 3000., 12000., 0.

1903, 3200., 9200., 3000.

1904, 3000., 9200., 6000.

1905, 3200., 9200., 6000.

1906, 3200., 9000., 6000.

1907, 3200., 9000., 3000.

1908, 3200., 9000., 0.

1909, 3200., 9200., 0.

1910, 3000., 9000., 6000.

1911, 3000., 9200., 0.

1912, 3000., 9000., 0.

1913, 3200., 6200., 6000.

1914, 3000., 6200., 6000.

1915, 3200., 6200., 3000.

1916, 3200., 3200., 9000.

1917, 3200., 3200., 8700.

1918, 3000., 3200., 6000.

1919, 3000., 6000., 6000.

1920, 3200., 6000., 6000.

1921, 3200., 3200., 6000.

1922, 3000., 3200., 2700.

1923, 3200., 6000., 3000.

1924, 3200., 3200., 3000.

1925, 3200., 200., 6000.

1926, 3200., 3000., 6000.

1927, 3000., 3000., 6000.

1928, 3000., 200., 6000.

1929, 3200., 3000., 9000.

1930, 3200., 3000., 8700.

1931, 3200., 200., 8700.

1932, 3200., 200., 9000.

1933, 3200., 200., 3000.

1934, 3200., 200., 0.

1935, 3000., 200., 0.

1936, 3200., 0., 9000.

1937, 3200., 0., 6000.

1938, 3200., 0., 3000.

1939, 3200., 0., 0.

1940, 3000., 0., 0.

1941, 3000., 0., 6000.

1942, 3200., 3000., 0.

1943, 3000., 3000., 0.

1944, 3000., 3200., 0.

1945, 3200., 3200., 0.

1946, 3200., 3000., 3000.

1947, 3000., 3000., 2700.

1948, 3200., 6000., 0.

1949, 3200., 6200., 0.

1950, 3000., 6200., 0.

1951, 3000., 6000., 0.

1952, 3200., 15000., 8700.

1953, 3000., 15000., 8700.

1954, 3000., 15000., 0.

1955, 3200., 15000., 0.

1956, 3000., 15200., 8700.

1957, 3000., 15200., 6000.

1958, 3000., 15200., 0.

1959, 3200., 15200., 0.

1960, 3200., 15200., 8700.

1961, 3200., 15200., 6000.

1962, 3200., 15200., 3000.

1963, 24000., 3200., 0.

1964, 24000., 3000., 0.

1965, 24200., 3200., 0.

1966, 24200., 3000., 0.

1967, 18200., 3000., 0.

1968, 18200., 3200., 0.

1969, 18000., 3200., 0.

1970, 18000., 3000., 0.

1971, 15000., 9000., 0.

1972, 15000., 9200., 0.

1973, 15200., 9200., 0.

1974, 15200., 9000., 0.

1975, 0., 15200., 9000.

1976, 0., 0., 9000.

1977, 0., 200., 9000.

1978, 0., 3000., 9000.

1979, 0., 3200., 9000.

1980, 0., 6000., 9000.

1981, 0., 6200., 9000.

1982, 0., 9000., 9000.

1983, 0., 9200., 9000.

1984, 0., 12000., 9000.

1985, 0., 12200., 9000.

1986, 0., 15000., 9000.

1987, 24200., 0., 2160.

1988, 24200., 0., 1620.

1989, 24200., 0., 1080.

1990, 24200., 0., 540.

1991, 24200., 200., 540.

1992, 24200., 200., 1080.

1993, 24200., 200., 1620.

1994, 24200., 200., 2160.

1995, 24000., 0., 540.

1996, 24000., 0., 1080.

1997, 24000., 0., 1620.

1998, 24000., 0., 2160.

1999, 24000., 200., 2160.

2000, 24000., 200., 1620.

2001, 24000., 200., 1080.

2002, 24000., 200., 540.

2003, 24200., 5533.3335, 2700.

2004, 24200., 5066.6665, 2700.

2005, 24200., 4600., 2700.

2006, 24200., 4133.3335, 2700.

2007, 24200., 3666.66675, 2700.

2008, 24000., 3666.66675, 2700.

2009, 24000., 4133.3335, 2700.

2010, 24000., 4600., 2700.

2011, 24000., 5066.6665, 2700.

2012, 24000., 5533.3335, 2700.

2013, 24000., 6666.6665, 2700.

2014, 24000., 7133.3335, 2700.

2015, 24000., 7600., 2700.

2016, 24000., 8066.6665, 2700.

2017, 24000., 8533.33301, 2700.

2018, 24200., 8533.33301, 2700.

2019, 24200., 8066.6665, 2700.

2020, 24200., 7600., 2700.

2021, 24200., 7133.3335, 2700.

2022, 24200., 6666.6665, 2700.

2023, 24200., 11533.333, 2700.

2024, 24200., 11066.667, 2700.

2025, 24200., 10600., 2700.

2026, 24200., 10133.333, 2700.

2027, 24200., 9666.66699, 2700.

2028, 24000., 9666.66699, 2700.

2029, 24000., 10133.333, 2700.

2030, 24000., 10600., 2700.

2031, 24000., 11066.667, 2700.

2032, 24000., 11533.333, 2700.

2033, 24000., 12666.667, 2700.

2034, 24000., 13133.333, 2700.

2035, 24000., 13600., 2700.

2036, 24000., 14066.667, 2700.

2037, 24000., 14533.333, 2700.

2038, 24200., 14533.333, 2700.

2039, 24200., 14066.667, 2700.

2040, 24200., 13600., 2700.

2041, 24200., 13133.333, 2700.

2042, 24200., 12666.667, 2700.

2043, 24000., 666.666687, 2700.

2044, 24000., 1133.33337, 2700.

2045, 24000., 1600., 2700.

2046, 24000., 2066.66675, 2700.

2047, 24000., 2533.33325, 2700.

2048, 24200., 2533.33325, 2700.

2049, 24200., 2066.66675, 2700.

2050, 24200., 1600., 2700.

2051, 24200., 1133.33337, 2700.

2052, 24200., 666.666687, 2700.

2053, 24000., 14533.333, 3000.

2054, 24000., 14066.667, 3000.

2055, 24000., 13600., 3000.

2056, 24000., 13133.333, 3000.

2057, 24000., 12666.667, 3000.

2058, 24200., 12666.667, 3000.

2059, 24200., 13133.333, 3000.

2060, 24200., 13600., 3000.

2061, 24200., 14066.667, 3000.

2062, 24200., 14533.333, 3000.

2063, 24000., 11533.333, 3000.

2064, 24000., 11066.667, 3000.

2065, 24000., 10600., 3000.

2066, 24000., 10133.333, 3000.

2067, 24000., 9666.66699, 3000.

2068, 24200., 9666.66699, 3000.

2069, 24200., 10133.333, 3000.

2070, 24200., 10600., 3000.

2071, 24200., 11066.667, 3000.

2072, 24200., 11533.333, 3000.

2073, 24000., 8533.33301, 3000.

2074, 24000., 8066.6665, 3000.

2075, 24000., 7600., 3000.

2076, 24000., 7133.3335, 3000.

2077, 24000., 6666.6665, 3000.

2078, 24200., 6666.6665, 3000.

2079, 24200., 7133.3335, 3000.

2080, 24200., 7600., 3000.

2081, 24200., 8066.6665, 3000.

2082, 24200., 8533.33301, 3000.

2083, 24000., 5533.3335, 3000.

2084, 24000., 5066.6665, 3000.

2085, 24000., 4600., 3000.

2086, 24000., 4133.3335, 3000.

2087, 24000., 3666.66675, 3000.

2088, 24200., 3666.66675, 3000.

2089, 24200., 4133.3335, 3000.

2090, 24200., 4600., 3000.

2091, 24200., 5066.6665, 3000.

2092, 24200., 5533.3335, 3000.

2093, 24000., 2533.33325, 3000.

2094, 24000., 2066.66675, 3000.

2095, 24000., 1600., 3000.

2096, 24000., 1133.33337, 3000.

2097, 24000., 666.666687, 3000.

2098, 24200., 666.666687, 3000.

2099, 24200., 1133.33337, 3000.

2100, 24200., 1600., 3000.

2101, 24200., 2066.66675, 3000.

2102, 24200., 2533.33325, 3000.

2103, 24000., 3666.66675, 2920.

2104, 24000., 4133.3335, 2920.

2105, 24000., 4600., 2920.

2106, 24000., 5066.6665, 2920.

2107, 24000., 5533.3335, 2920.

2108, 24000., 6666.6665, 2920.

2109, 24000., 7133.3335, 2920.

2110, 24000., 7600., 2920.

2111, 24000., 8066.6665, 2920.

2112, 24000., 8533.33301, 2920.

2113, 24000., 9666.66699, 2920.

2114, 24000., 10133.333, 2920.

2115, 24000., 10600., 2920.

2116, 24000., 11066.667, 2920.

2117, 24000., 11533.333, 2920.

2118, 24000., 12666.667, 2920.

2119, 24000., 13133.333, 2920.

2120, 24000., 13600., 2920.

2121, 24000., 14066.667, 2920.

2122, 24000., 14533.333, 2920.

2123, 24000., 666.666687, 2920.

2124, 24000., 1133.33337, 2920.

2125, 24000., 1600., 2920.

2126, 24000., 2066.66675, 2920.

2127, 24000., 2533.33325, 2920.

2128, 24200., 0., 2850.

2129, 24200., 15200., 2850.

2130, 24000., 15200., 2160.

2131, 24000., 15200., 1620.

2132, 24000., 15200., 1080.

2133, 24000., 15200., 540.

2134, 24000., 15000., 540.

2135, 24000., 15000., 1080.

2136, 24000., 15000., 1620.

2137, 24000., 15000., 2160.

2138, 24200., 15200., 540.

2139, 24200., 15200., 1080.

2140, 24200., 15200., 1620.

2141, 24200., 15200., 2160.

2142, 24200., 15000., 2160.

2143, 24200., 15000., 1620.

2144, 24200., 15000., 1080.

2145, 24200., 15000., 540.

2146, 24000., 12200., 2160.

2147, 24000., 12200., 1620.

2148, 24000., 12200., 1080.

2149, 24000., 12200., 540.

2150, 24000., 12000., 540.

2151, 24000., 12000., 1080.

2152, 24000., 12000., 1620.

2153, 24000., 12000., 2160.

2154, 24200., 12200., 540.

2155, 24200., 12200., 1080.

2156, 24200., 12200., 1620.

2157, 24200., 12200., 2160.

2158, 24200., 12000., 2160.

2159, 24200., 12000., 1620.

2160, 24200., 12000., 1080.

2161, 24200., 12000., 540.

2162, 24000., 9200., 2160.

2163, 24000., 9200., 1620.

2164, 24000., 9200., 1080.

2165, 24000., 9200., 540.

2166, 24000., 9000., 540.

2167, 24000., 9000., 1080.

2168, 24000., 9000., 1620.

2169, 24000., 9000., 2160.

2170, 24200., 9200., 540.

2171, 24200., 9200., 1080.

2172, 24200., 9200., 1620.

2173, 24200., 9200., 2160.

2174, 24200., 9000., 2160.

2175, 24200., 9000., 1620.

2176, 24200., 9000., 1080.

2177, 24200., 9000., 540.

2178, 24000., 6200., 2160.

2179, 24000., 6200., 1620.

2180, 24000., 6200., 1080.

2181, 24000., 6200., 540.

2182, 24000., 6000., 540.

2183, 24000., 6000., 1080.

2184, 24000., 6000., 1620.

2185, 24000., 6000., 2160.

2186, 24200., 6200., 540.

2187, 24200., 6200., 1080.

2188, 24200., 6200., 1620.

2189, 24200., 6200., 2160.

2190, 24200., 6000., 2160.

2191, 24200., 6000., 1620.

2192, 24200., 6000., 1080.

2193, 24200., 6000., 540.

2194, 24000., 15000., 5160.

2195, 24000., 15000., 4620.

2196, 24000., 15000., 4080.

2197, 24000., 15000., 3540.

2198, 24200., 15000., 3540.

2199, 24200., 15000., 4080.

2200, 24200., 15000., 4620.

2201, 24200., 15000., 5160.

2202, 24000., 15200., 3540.

2203, 24000., 15200., 4080.

2204, 24000., 15200., 4620.

2205, 24000., 15200., 5160.

2206, 24200., 15200., 5160.

2207, 24200., 15200., 4620.

2208, 24200., 15200., 4080.

2209, 24200., 15200., 3540.

2210, 24000., 12000., 5160.

2211, 24000., 12000., 4620.

2212, 24000., 12000., 4080.

2213, 24000., 12000., 3540.

2214, 24200., 12000., 3540.

2215, 24200., 12000., 4080.

2216, 24200., 12000., 4620.

2217, 24200., 12000., 5160.

2218, 24000., 12200., 3540.

2219, 24000., 12200., 4080.

2220, 24000., 12200., 4620.

2221, 24000., 12200., 5160.

2222, 24200., 12200., 5160.

2223, 24200., 12200., 4620.

2224, 24200., 12200., 4080.

2225, 24200., 12200., 3540.

2226, 24000., 9000., 5160.

2227, 24000., 9000., 4620.

2228, 24000., 9000., 4080.

2229, 24000., 9000., 3540.

2230, 24200., 9000., 3540.

2231, 24200., 9000., 4080.

2232, 24200., 9000., 4620.

2233, 24200., 9000., 5160.

2234, 24000., 9200., 3540.

2235, 24000., 9200., 4080.

2236, 24000., 9200., 4620.

2237, 24000., 9200., 5160.

2238, 24200., 9200., 5160.

2239, 24200., 9200., 4620.

2240, 24200., 9200., 4080.

2241, 24200., 9200., 3540.

2242, 24000., 6000., 5160.

2243, 24000., 6000., 4620.

2244, 24000., 6000., 4080.

2245, 24000., 6000., 3540.

2246, 24200., 6000., 3540.

2247, 24200., 6000., 4080.

2248, 24200., 6000., 4620.

2249, 24200., 6000., 5160.

2250, 24000., 6200., 3540.

2251, 24000., 6200., 4080.

2252, 24000., 6200., 4620.

2253, 24000., 6200., 5160.

2254, 24200., 6200., 5160.

2255, 24200., 6200., 4620.

2256, 24200., 6200., 4080.

2257, 24200., 6200., 3540.

2258, 24000., 3000., 5160.

2259, 24000., 3000., 4620.

2260, 24000., 3000., 4080.

2261, 24000., 3000., 3540.

2262, 24200., 3000., 3540.

2263, 24200., 3000., 4080.

2264, 24200., 3000., 4620.

2265, 24200., 3000., 5160.

2266, 24000., 3200., 3540.

2267, 24000., 3200., 4080.

2268, 24000., 3200., 4620.

2269, 24000., 3200., 5160.

2270, 24200., 3200., 5160.

2271, 24200., 3200., 4620.

2272, 24200., 3200., 4080.

2273, 24200., 3200., 3540.

2274, 24200., 200., 5160.

2275, 24200., 200., 4620.

2276, 24200., 200., 4080.

2277, 24200., 200., 3540.

2278, 24000., 200., 3540.

2279, 24000., 200., 4080.

2280, 24000., 200., 4620.

2281, 24000., 200., 5160.

2282, 24200., 0., 3540.

2283, 24200., 0., 4080.

2284, 24200., 0., 4620.

2285, 24200., 0., 5160.

2286, 24000., 0., 5160.

2287, 24000., 0., 4620.

2288, 24000., 0., 4080.

2289, 24000., 0., 3540.

2290, 24200., 12200., 6540.

2291, 24200., 12200., 7080.

2292, 24200., 12200., 7620.

2293, 24200., 12200., 8160.

2294, 24200., 12000., 8160.

2295, 24200., 12000., 7620.

2296, 24200., 12000., 7080.

2297, 24200., 12000., 6540.

2298, 24000., 12200., 8160.

2299, 24000., 12200., 7620.

2300, 24000., 12200., 7080.

2301, 24000., 12200., 6540.

2302, 24000., 12000., 6540.

2303, 24000., 12000., 7080.

2304, 24000., 12000., 7620.

2305, 24000., 12000., 8160.

2306, 24200., 5533.3335, 5700.

2307, 24200., 5066.6665, 5700.

2308, 24200., 4600., 5700.

2309, 24200., 4133.3335, 5700.

2310, 24200., 3666.66675, 5700.

2311, 24000., 3666.66675, 5700.

2312, 24000., 4133.3335, 5700.

2313, 24000., 4600., 5700.

2314, 24000., 5066.6665, 5700.

2315, 24000., 5533.3335, 5700.

2316, 24200., 8533.33301, 5700.

2317, 24200., 8066.6665, 5700.

2318, 24200., 7600., 5700.

2319, 24200., 7133.3335, 5700.

2320, 24200., 6666.6665, 5700.

2321, 24000., 6666.6665, 5700.

2322, 24000., 7133.3335, 5700.

2323, 24000., 7600., 5700.

2324, 24000., 8066.6665, 5700.

2325, 24000., 8533.33301, 5700.

2326, 24200., 11533.333, 5700.

2327, 24200., 11066.667, 5700.

2328, 24200., 10600., 5700.

2329, 24200., 10133.333, 5700.

2330, 24200., 9666.66699, 5700.

2331, 24000., 9666.66699, 5700.

2332, 24000., 10133.333, 5700.

2333, 24000., 10600., 5700.

2334, 24000., 11066.667, 5700.

2335, 24000., 11533.333, 5700.

2336, 24200., 14533.333, 5700.

2337, 24200., 14066.667, 5700.

2338, 24200., 13600., 5700.

2339, 24200., 13133.333, 5700.

2340, 24200., 12666.667, 5700.

2341, 24000., 12666.667, 5700.

2342, 24000., 13133.333, 5700.

2343, 24000., 13600., 5700.

2344, 24000., 14066.667, 5700.

2345, 24000., 14533.333, 5700.

2346, 24200., 2533.33325, 5700.

2347, 24200., 2066.66675, 5700.

2348, 24200., 1600., 5700.

2349, 24200., 1133.33337, 5700.

2350, 24200., 666.666687, 5700.

2351, 24000., 666.666687, 5700.

2352, 24000., 1133.33337, 5700.

2353, 24000., 1600., 5700.

2354, 24000., 2066.66675, 5700.

2355, 24000., 2533.33325, 5700.

2356, 24000., 14533.333, 6000.

2357, 24000., 14066.667, 6000.

2358, 24000., 13600., 6000.

2359, 24000., 13133.333, 6000.

2360, 24000., 12666.667, 6000.

2361, 24200., 12666.667, 6000.

2362, 24200., 13133.333, 6000.

2363, 24200., 13600., 6000.

2364, 24200., 14066.667, 6000.

2365, 24200., 14533.333, 6000.

2366, 24000., 11533.333, 6000.

2367, 24000., 11066.667, 6000.

2368, 24000., 10600., 6000.

2369, 24000., 10133.333, 6000.

2370, 24000., 9666.66699, 6000.

2371, 24200., 9666.66699, 6000.

2372, 24200., 10133.333, 6000.

2373, 24200., 10600., 6000.

2374, 24200., 11066.667, 6000.

2375, 24200., 11533.333, 6000.

2376, 24000., 8533.33301, 6000.

2377, 24000., 8066.6665, 6000.

2378, 24000., 7600., 6000.

2379, 24000., 7133.3335, 6000.

2380, 24000., 6666.6665, 6000.

2381, 24200., 6666.6665, 6000.

2382, 24200., 7133.3335, 6000.

2383, 24200., 7600., 6000.

2384, 24200., 8066.6665, 6000.

2385, 24200., 8533.33301, 6000.

2386, 24000., 5533.3335, 6000.

2387, 24000., 5066.6665, 6000.

2388, 24000., 4600., 6000.

2389, 24000., 4133.3335, 6000.

2390, 24000., 3666.66675, 6000.

2391, 24200., 3666.66675, 6000.

2392, 24200., 4133.3335, 6000.

2393, 24200., 4600., 6000.

2394, 24200., 5066.6665, 6000.

2395, 24200., 5533.3335, 6000.

2396, 24000., 2533.33325, 6000.

2397, 24000., 2066.66675, 6000.

2398, 24000., 1600., 6000.

2399, 24000., 1133.33337, 6000.

2400, 24000., 666.666687, 6000.

2401, 24200., 666.666687, 6000.

2402, 24200., 1133.33337, 6000.

2403, 24200., 1600., 6000.

2404, 24200., 2066.66675, 6000.

2405, 24200., 2533.33325, 6000.

2406, 24000., 3666.66675, 5920.

2407, 24000., 4133.3335, 5920.

2408, 24000., 4600., 5920.

2409, 24000., 5066.6665, 5920.

2410, 24000., 5533.3335, 5920.

2411, 24000., 6666.6665, 5920.

2412, 24000., 7133.3335, 5920.

2413, 24000., 7600., 5920.

2414, 24000., 8066.6665, 5920.

2415, 24000., 8533.33301, 5920.

2416, 24000., 9666.66699, 5920.

2417, 24000., 10133.333, 5920.

2418, 24000., 10600., 5920.

2419, 24000., 11066.667, 5920.

2420, 24000., 11533.333, 5920.

2421, 24000., 12666.667, 5920.

2422, 24000., 13133.333, 5920.

2423, 24000., 13600., 5920.

2424, 24000., 14066.667, 5920.

2425, 24000., 14533.333, 5920.

2426, 24000., 666.666687, 5920.

2427, 24000., 1133.33337, 5920.

2428, 24000., 1600., 5920.

2429, 24000., 2066.66675, 5920.

2430, 24000., 2533.33325, 5920.

2431, 24200., 0., 5850.

2432, 24200., 15200., 5850.

2433, 24000., 15000., 8160.

2434, 24000., 15000., 7620.

2435, 24000., 15000., 7080.

2436, 24000., 15000., 6540.

2437, 24200., 15000., 6540.

2438, 24200., 15000., 7080.

2439, 24200., 15000., 7620.

2440, 24200., 15000., 8160.

2441, 24000., 15200., 6540.

2442, 24000., 15200., 7080.

2443, 24000., 15200., 7620.

2444, 24000., 15200., 8160.

2445, 24200., 15200., 8160.

2446, 24200., 15200., 7620.

2447, 24200., 15200., 7080.

2448, 24200., 15200., 6540.

2449, 24000., 9000., 8160.

2450, 24000., 9000., 7620.

2451, 24000., 9000., 7080.

2452, 24000., 9000., 6540.

2453, 24200., 9000., 6540.

2454, 24200., 9000., 7080.

2455, 24200., 9000., 7620.

2456, 24200., 9000., 8160.

2457, 24000., 9200., 6540.

2458, 24000., 9200., 7080.

2459, 24000., 9200., 7620.

2460, 24000., 9200., 8160.

2461, 24200., 9200., 8160.

2462, 24200., 9200., 7620.

2463, 24200., 9200., 7080.

2464, 24200., 9200., 6540.

2465, 24000., 6000., 8160.

2466, 24000., 6000., 7620.

2467, 24000., 6000., 7080.

2468, 24000., 6000., 6540.

2469, 24200., 6000., 6540.

2470, 24200., 6000., 7080.

2471, 24200., 6000., 7620.

2472, 24200., 6000., 8160.

2473, 24000., 6200., 6540.

2474, 24000., 6200., 7080.

2475, 24000., 6200., 7620.

2476, 24000., 6200., 8160.

2477, 24200., 6200., 8160.

2478, 24200., 6200., 7620.

2479, 24200., 6200., 7080.

2480, 24200., 6200., 6540.

2481, 24000., 3000., 8160.

2482, 24000., 3000., 7620.

2483, 24000., 3000., 7080.

2484, 24000., 3000., 6540.

2485, 24200., 3000., 6540.

2486, 24200., 3000., 7080.

2487, 24200., 3000., 7620.

2488, 24200., 3000., 8160.

2489, 24000., 3200., 6540.

2490, 24000., 3200., 7080.

2491, 24000., 3200., 7620.

2492, 24000., 3200., 8160.

2493, 24200., 3200., 8160.

2494, 24200., 3200., 7620.

2495, 24200., 3200., 7080.

2496, 24200., 3200., 6540.

2497, 24000., 0., 6540.

2498, 24000., 0., 7080.

2499, 24000., 0., 7620.

2500, 24000., 0., 8160.

2501, 24000., 200., 8160.

2502, 24000., 200., 7620.

2503, 24000., 200., 7080.

2504, 24000., 200., 6540.

2505, 24200., 0., 8160.

2506, 24200., 0., 7620.

2507, 24200., 0., 7080.

2508, 24200., 0., 6540.

2509, 24200., 200., 6540.

2510, 24200., 200., 7080.

2511, 24200., 200., 7620.

2512, 24200., 200., 8160.

2513, 24200., 666.666687, 8700.

2514, 24200., 1133.33337, 8700.

2515, 24200., 1600., 8700.

2516, 24200., 2066.66675, 8700.

2517, 24200., 2533.33325, 8700.

2518, 24200., 3760., 8700.

2519, 24200., 4320., 8700.

2520, 24200., 4880., 8700.

2521, 24200., 5440., 8700.

2522, 24200., 6666.6665, 8700.

2523, 24200., 7133.3335, 8700.

2524, 24200., 7600., 8700.

2525, 24200., 8066.6665, 8700.

2526, 24200., 8533.33301, 8700.

2527, 24200., 9666.66699, 8700.

2528, 24200., 10133.333, 8700.

2529, 24200., 10600., 8700.

2530, 24200., 11066.667, 8700.

2531, 24200., 11533.333, 8700.

2532, 24200., 12666.667, 8700.

2533, 24200., 13133.333, 8700.

2534, 24200., 13600., 8700.

2535, 24200., 14066.667, 8700.

2536, 24200., 14533.333, 8700.

2537, 24200., 15200., 8850.

2538, 24200., 2440., 9000.

2539, 24200., 1880., 9000.

2540, 24200., 1320., 9000.

2541, 24200., 760., 9000.

2542, 24200., 5440., 9000.

2543, 24200., 4880., 9000.

2544, 24200., 4320., 9000.

2545, 24200., 3760., 9000.

2546, 24200., 8440., 9000.

2547, 24200., 7880., 9000.

2548, 24200., 7320., 9000.

2549, 24200., 6760., 9000.

2550, 24200., 11533.333, 9000.

2551, 24200., 11066.667, 9000.

2552, 24200., 10600., 9000.

2553, 24200., 10133.333, 9000.

2554, 24200., 9666.66699, 9000.

2555, 24200., 14533.333, 9000.

2556, 24200., 14066.667, 9000.

2557, 24200., 13600., 9000.

2558, 24200., 13133.333, 9000.

2559, 24200., 12666.667, 9000.

2560, 24200., 15133.333, 9000.

2561, 24200., 15066.667, 9000.

2562, 24200., 0., 8850.

2563, 24000., 666.666687, 8920.

2564, 24000., 1133.33337, 8920.

2565, 24000., 1600., 8920.

2566, 24000., 2066.66675, 8920.

2567, 24000., 2533.33325, 8920.

2568, 24000., 2533.33325, 8700.

2569, 24000., 2066.66675, 8700.

2570, 24000., 1600., 8700.

2571, 24000., 1133.33337, 8700.

2572, 24000., 666.666687, 8700.

2573, 24000., 3760., 8920.

2574, 24000., 4320., 8920.

2575, 24000., 4880., 8920.

2576, 24000., 5440., 8920.

2577, 24000., 5440., 8700.

2578, 24000., 4880., 8700.

2579, 24000., 4320., 8700.

2580, 24000., 3760., 8700.

2581, 24000., 6666.6665, 8920.

2582, 24000., 7133.3335, 8920.

2583, 24000., 7600., 8920.

2584, 24000., 8066.6665, 8920.

2585, 24000., 8533.33301, 8920.

2586, 24000., 8533.33301, 8700.

2587, 24000., 8066.6665, 8700.

2588, 24000., 7600., 8700.

2589, 24000., 7133.3335, 8700.

2590, 24000., 6666.6665, 8700.

2591, 24000., 9666.66699, 8920.

2592, 24000., 10133.333, 8920.

2593, 24000., 10600., 8920.

2594, 24000., 11066.667, 8920.

2595, 24000., 11533.333, 8920.

2596, 24000., 11533.333, 8700.

2597, 24000., 11066.667, 8700.

2598, 24000., 10600., 8700.

2599, 24000., 10133.333, 8700.

2600, 24000., 9666.66699, 8700.

2601, 24000., 12666.667, 8920.

2602, 24000., 13133.333, 8920.

2603, 24000., 13600., 8920.

2604, 24000., 14066.667, 8920.

2605, 24000., 14533.333, 8920.

2606, 24000., 14533.333, 8700.

2607, 24000., 14066.667, 8700.

2608, 24000., 13600., 8700.

2609, 24000., 13133.333, 8700.

2610, 24000., 12666.667, 8700.

2611, 24000., 434.285706, 9000.

2612, 24000., 868.571411, 9000.

2613, 24000., 1302.85718, 9000.

2614, 24000., 1737.14282, 9000.

2615, 24000., 2171.42847, 9000.

2616, 24000., 2605.71436, 9000.

2617, 24000., 3040., 9000.

2618, 24000., 3474.28564, 9000.

2619, 24000., 3908.57153, 9000.

2620, 24000., 4342.85693, 9000.

2621, 24000., 4777.14307, 9000.

2622, 24000., 5211.42871, 9000.

2623, 24000., 5645.71436, 9000.

2624, 24000., 6080., 9000.

2625, 24000., 6514.28564, 9000.

2626, 24000., 6948.57129, 9000.

2627, 24000., 7382.85693, 9000.

2628, 24000., 7817.14307, 9000.

2629, 24000., 8251.42871, 9000.

2630, 24000., 8685.71387, 9000.

2631, 24000., 9120., 9000.

2632, 24000., 9554.28613, 9000.

2633, 24000., 9988.57129, 9000.

2634, 24000., 10422.8574, 9000.

2635, 24000., 10857.1426, 9000.

2636, 24000., 11291.4287, 9000.

2637, 24000., 11725.7139, 9000.

2638, 24000., 12160., 9000.

2639, 24000., 12594.2861, 9000.

2640, 24000., 13028.5713, 9000.

2641, 24000., 13462.8574, 9000.

2642, 24000., 13897.1426, 9000.

2643, 24000., 14331.4287, 9000.

2644, 24000., 14765.7139, 9000.

2645, 8533.33301, 9000., 8920.

2646, 8066.6665, 9000., 8920.

2647, 7600., 9000., 8920.

2648, 7133.3335, 9000., 8920.

2649, 6666.6665, 9000., 8920.

2650, 6666.6665, 9000., 8700.

2651, 7133.3335, 9000., 8700.

2652, 7600., 9000., 8700.

2653, 8066.6665, 9000., 8700.

2654, 8533.33301, 9000., 8700.

2655, 6666.6665, 9200., 8920.

2656, 7133.3335, 9200., 8920.

2657, 7600., 9200., 8920.

2658, 8066.6665, 9200., 8920.

2659, 8533.33301, 9200., 8920.

2660, 8533.33301, 9200., 8700.

2661, 8066.6665, 9200., 8700.

2662, 7600., 9200., 8700.

2663, 7133.3335, 9200., 8700.

2664, 6666.6665, 9200., 8700.

2665, 6666.6665, 12200., 8920.

2666, 7133.3335, 12200., 8920.

2667, 7600., 12200., 8920.

2668, 8066.6665, 12200., 8920.

2669, 8533.33301, 12200., 8920.

2670, 8533.33301, 12200., 8700.

2671, 8066.6665, 12200., 8700.

2672, 7600., 12200., 8700.

2673, 7133.3335, 12200., 8700.

2674, 6666.6665, 12200., 8700.

2675, 8533.33301, 12000., 8920.

2676, 8066.6665, 12000., 8920.

2677, 7600., 12000., 8920.

2678, 7133.3335, 12000., 8920.

2679, 6666.6665, 12000., 8920.

2680, 6666.6665, 12000., 8700.

2681, 7133.3335, 12000., 8700.

2682, 7600., 12000., 8700.

2683, 8066.6665, 12000., 8700.

2684, 8533.33301, 12000., 8700.

2685, 8533.33301, 6000., 8700.

2686, 8066.6665, 6000., 8700.

2687, 7600., 6000., 8700.

2688, 7133.3335, 6000., 8700.

2689, 6666.6665, 6000., 8700.

2690, 6666.6665, 6200., 8700.

2691, 7133.3335, 6200., 8700.

2692, 7600., 6200., 8700.

2693, 8066.6665, 6200., 8700.

2694, 8533.33301, 6200., 8700.

2695, 8533.33301, 6000., 8920.

2696, 8066.6665, 6000., 8920.

2697, 7600., 6000., 8920.

2698, 7133.3335, 6000., 8920.

2699, 6666.6665, 6000., 8920.

2700, 6666.6665, 6200., 8920.

2701, 7133.3335, 6200., 8920.

2702, 7600., 6200., 8920.

2703, 8066.6665, 6200., 8920.

2704, 8533.33301, 6200., 8920.

2705, 6666.6665, 15200., 8920.

2706, 7133.3335, 15200., 8920.

2707, 7600., 15200., 8920.

2708, 8066.6665, 15200., 8920.

2709, 8533.33301, 15200., 8920.

2710, 8533.33301, 15000., 8920.

2711, 8066.6665, 15000., 8920.

2712, 7600., 15000., 8920.

2713, 7133.3335, 15000., 8920.

2714, 6666.6665, 15000., 8920.

2715, 9000., 14533.333, 8920.

2716, 9000., 14066.667, 8920.

2717, 9000., 13600., 8920.

2718, 9000., 13133.333, 8920.

2719, 9000., 12666.667, 8920.

2720, 6200., 12666.667, 8920.

2721, 6200., 13133.333, 8920.

2722, 6200., 13600., 8920.

2723, 6200., 14066.667, 8920.

2724, 6200., 14533.333, 8920.

2725, 9000., 11533.333, 8920.

2726, 9000., 11066.667, 8920.

2727, 9000., 10600., 8920.

2728, 9000., 10133.333, 8920.

2729, 9000., 9666.66699, 8920.

2730, 6200., 9666.66699, 8920.

2731, 6200., 10133.333, 8920.

2732, 6200., 10600., 8920.

2733, 6200., 11066.667, 8920.

2734, 6200., 11533.333, 8920.

2735, 9000., 8533.33301, 8920.

2736, 9000., 8066.6665, 8920.

2737, 9000., 7600., 8920.

2738, 9000., 7133.3335, 8920.

2739, 9000., 6666.6665, 8920.

2740, 6200., 6666.6665, 8920.

2741, 6200., 7133.3335, 8920.

2742, 6200., 7600., 8920.

2743, 6200., 8066.6665, 8920.

2744, 6200., 8533.33301, 8920.

2745, 8533.33301, 15200., 9000.

2746, 8066.6665, 15200., 9000.

2747, 7600., 15200., 9000.

2748, 7133.3335, 15200., 9000.

2749, 6666.6665, 15200., 9000.

2750, 9000., 12666.667, 9000.

2751, 9000., 13133.333, 9000.

2752, 9000., 13600., 9000.

2753, 9000., 14066.667, 9000.

2754, 9000., 14533.333, 9000.

2755, 9000., 9666.66699, 9000.

2756, 9000., 10133.333, 9000.

2757, 9000., 10600., 9000.

2758, 9000., 11066.667, 9000.

2759, 9000., 11533.333, 9000.

2760, 9000., 6666.6665, 9000.

2761, 9000., 7133.3335, 9000.

2762, 9000., 7600., 9000.

2763, 9000., 8066.6665, 9000.

2764, 9000., 8533.33301, 9000.

2765, 6666.6665, 6000., 9000.

2766, 7133.3335, 6000., 9000.

2767, 7600., 6000., 9000.

2768, 8066.6665, 6000., 9000.

2769, 8533.33301, 6000., 9000.

2770, 6200., 14533.333, 9000.

2771, 6200., 14066.667, 9000.

2772, 6200., 13600., 9000.

2773, 6200., 13133.333, 9000.

2774, 6200., 12666.667, 9000.

2775, 6200., 11533.333, 9000.

2776, 6200., 11066.667, 9000.

2777, 6200., 10600., 9000.

2778, 6200., 10133.333, 9000.

2779, 6200., 9666.66699, 9000.

2780, 6200., 8533.33301, 9000.

2781, 6200., 8066.6665, 9000.

2782, 6200., 7600., 9000.

2783, 6200., 7133.3335, 9000.

2784, 6200., 6666.6665, 9000.

2785, 9666.66699, 15200., 8920.

2786, 10133.333, 15200., 8920.

2787, 10600., 15200., 8920.

2788, 11066.667, 15200., 8920.

2789, 11533.333, 15200., 8920.

2790, 11533.333, 15200., 8700.

2791, 11066.667, 15200., 8700.

2792, 10600., 15200., 8700.

2793, 10133.333, 15200., 8700.

2794, 9666.66699, 15200., 8700.

2795, 11533.333, 15000., 8920.

2796, 11066.667, 15000., 8920.

2797, 10600., 15000., 8920.

2798, 10133.333, 15000., 8920.

2799, 9666.66699, 15000., 8920.

2800, 9666.66699, 15000., 8700.

2801, 10133.333, 15000., 8700.

2802, 10600., 15000., 8700.

2803, 11066.667, 15000., 8700.

2804, 11533.333, 15000., 8700.

2805, 11533.333, 12000., 8920.

2806, 11066.667, 12000., 8920.

2807, 10600., 12000., 8920.

2808, 10133.333, 12000., 8920.

2809, 9666.66699, 12000., 8920.

2810, 9666.66699, 12000., 8700.

2811, 10133.333, 12000., 8700.

2812, 10600., 12000., 8700.

2813, 11066.667, 12000., 8700.

2814, 11533.333, 12000., 8700.

2815, 9666.66699, 12200., 8920.

2816, 10133.333, 12200., 8920.

2817, 10600., 12200., 8920.

2818, 11066.667, 12200., 8920.

2819, 11533.333, 12200., 8920.

2820, 11533.333, 12200., 8700.

2821, 11066.667, 12200., 8700.

2822, 10600., 12200., 8700.

2823, 10133.333, 12200., 8700.

2824, 9666.66699, 12200., 8700.

2825, 11533.333, 6000., 8700.

2826, 11066.667, 6000., 8700.

2827, 10600., 6000., 8700.

2828, 10133.333, 6000., 8700.

2829, 9666.66699, 6000., 8700.

2830, 9666.66699, 6200., 8700.

2831, 10133.333, 6200., 8700.

2832, 10600., 6200., 8700.

2833, 11066.667, 6200., 8700.

2834, 11533.333, 6200., 8700.

2835, 11533.333, 6000., 8920.

2836, 11066.667, 6000., 8920.

2837, 10600., 6000., 8920.

2838, 10133.333, 6000., 8920.

2839, 9666.66699, 6000., 8920.

2840, 9666.66699, 6200., 8920.

2841, 10133.333, 6200., 8920.

2842, 10600., 6200., 8920.

2843, 11066.667, 6200., 8920.

2844, 11533.333, 6200., 8920.

2845, 11533.333, 9000., 8920.

2846, 11066.667, 9000., 8920.

2847, 10600., 9000., 8920.

2848, 10133.333, 9000., 8920.

2849, 9666.66699, 9000., 8920.

2850, 9666.66699, 9000., 8700.

2851, 10133.333, 9000., 8700.

2852, 10600., 9000., 8700.

2853, 11066.667, 9000., 8700.

2854, 11533.333, 9000., 8700.

2855, 9666.66699, 9200., 8920.

2856, 10133.333, 9200., 8920.

2857, 10600., 9200., 8920.

2858, 11066.667, 9200., 8920.

2859, 11533.333, 9200., 8920.

2860, 11533.333, 9200., 8700.

2861, 11066.667, 9200., 8700.

2862, 10600., 9200., 8700.

2863, 10133.333, 9200., 8700.

2864, 9666.66699, 9200., 8700.

2865, 14533.333, 9000., 8920.

2866, 14066.667, 9000., 8920.

2867, 13600., 9000., 8920.

2868, 13133.333, 9000., 8920.

2869, 12666.667, 9000., 8920.

2870, 12666.667, 9000., 8700.

2871, 13133.333, 9000., 8700.

2872, 13600., 9000., 8700.

2873, 14066.667, 9000., 8700.

2874, 14533.333, 9000., 8700.

2875, 12666.667, 9200., 8920.

2876, 13133.333, 9200., 8920.

2877, 13600., 9200., 8920.

2878, 14066.667, 9200., 8920.

2879, 14533.333, 9200., 8920.

2880, 14533.333, 9200., 8700.

2881, 14066.667, 9200., 8700.

2882, 13600., 9200., 8700.

2883, 13133.333, 9200., 8700.

2884, 12666.667, 9200., 8700.

2885, 12666.667, 12200., 8920.

2886, 13133.333, 12200., 8920.

2887, 13600., 12200., 8920.

2888, 14066.667, 12200., 8920.

2889, 14533.333, 12200., 8920.

2890, 14533.333, 12200., 8700.

2891, 14066.667, 12200., 8700.

2892, 13600., 12200., 8700.

2893, 13133.333, 12200., 8700.

2894, 12666.667, 12200., 8700.

2895, 14533.333, 12000., 8920.

2896, 14066.667, 12000., 8920.

2897, 13600., 12000., 8920.

2898, 13133.333, 12000., 8920.

2899, 12666.667, 12000., 8920.

2900, 12666.667, 12000., 8700.

2901, 13133.333, 12000., 8700.

2902, 13600., 12000., 8700.

2903, 14066.667, 12000., 8700.

2904, 14533.333, 12000., 8700.

2905, 12666.667, 15200., 8920.

2906, 13133.333, 15200., 8920.

2907, 13600., 15200., 8920.

2908, 14066.667, 15200., 8920.

2909, 14533.333, 15200., 8920.

2910, 14533.333, 15000., 8920.

2911, 14066.667, 15000., 8920.

2912, 13600., 15000., 8920.

2913, 13133.333, 15000., 8920.

2914, 12666.667, 15000., 8920.

2915, 12200., 12666.667, 8920.

2916, 12200., 13133.333, 8920.

2917, 12200., 13600., 8920.

2918, 12200., 14066.667, 8920.

2919, 12200., 14533.333, 8920.

2920, 15000., 14533.333, 8920.

2921, 15000., 14066.667, 8920.

2922, 15000., 13600., 8920.

2923, 15000., 13133.333, 8920.

2924, 15000., 12666.667, 8920.

2925, 12200., 9666.66699, 8920.

2926, 12200., 10133.333, 8920.

2927, 12200., 10600., 8920.

2928, 12200., 11066.667, 8920.

2929, 12200., 11533.333, 8920.

2930, 15000., 11533.333, 8920.

2931, 15000., 11066.667, 8920.

2932, 15000., 10600., 8920.

2933, 15000., 10133.333, 8920.

2934, 15000., 9666.66699, 8920.

2935, 12200., 6666.6665, 8920.

2936, 12200., 7133.3335, 8920.

2937, 12200., 7600., 8920.

2938, 12200., 8066.6665, 8920.

2939, 12200., 8533.33301, 8920.

2940, 15000., 8533.33301, 8920.

2941, 15000., 8066.6665, 8920.

2942, 15000., 7600., 8920.

2943, 15000., 7133.3335, 8920.

2944, 15000., 6666.6665, 8920.

2945, 14533.333, 6200., 8920.

2946, 14066.667, 6200., 8920.

2947, 13600., 6200., 8920.

2948, 13133.333, 6200., 8920.

2949, 12666.667, 6200., 8920.

2950, 14533.333, 6000., 8920.

2951, 14066.667, 6000., 8920.

2952, 13600., 6000., 8920.

2953, 13133.333, 6000., 8920.

2954, 12666.667, 6000., 8920.

2955, 14533.333, 15200., 9000.

2956, 14066.667, 15200., 9000.

2957, 13600., 15200., 9000.

2958, 13133.333, 15200., 9000.

2959, 12666.667, 15200., 9000.

2960, 15000., 12666.667, 9000.

2961, 15000., 13133.333, 9000.

2962, 15000., 13600., 9000.

2963, 15000., 14066.667, 9000.

2964, 15000., 14533.333, 9000.

2965, 15000., 9666.66699, 9000.

2966, 15000., 10133.333, 9000.

2967, 15000., 10600., 9000.

2968, 15000., 11066.667, 9000.

2969, 15000., 11533.333, 9000.

2970, 15000., 6666.6665, 9000.

2971, 15000., 7133.3335, 9000.

2972, 15000., 7600., 9000.

2973, 15000., 8066.6665, 9000.

2974, 15000., 8533.33301, 9000.

2975, 12666.667, 6000., 9000.

2976, 13133.333, 6000., 9000.

2977, 13600., 6000., 9000.

2978, 14066.667, 6000., 9000.

2979, 14533.333, 6000., 9000.

2980, 12200., 14533.333, 9000.

2981, 12200., 14066.667, 9000.

2982, 12200., 13600., 9000.

2983, 12200., 13133.333, 9000.

2984, 12200., 12666.667, 9000.

2985, 12200., 11533.333, 9000.

2986, 12200., 11066.667, 9000.

2987, 12200., 10600., 9000.

2988, 12200., 10133.333, 9000.

2989, 12200., 9666.66699, 9000.

2990, 12200., 8533.33301, 9000.

2991, 12200., 8066.6665, 9000.

2992, 12200., 7600., 9000.

2993, 12200., 7133.3335, 9000.

2994, 12200., 6666.6665, 9000.

2995, 14533.333, 6000., 8700.

2996, 14066.667, 6000., 8700.

2997, 13600., 6000., 8700.

2998, 13133.333, 6000., 8700.

2999, 12666.667, 6000., 8700.

3000, 12666.667, 6200., 8700.

3001, 13133.333, 6200., 8700.

3002, 13600., 6200., 8700.

3003, 14066.667, 6200., 8700.

3004, 14533.333, 6200., 8700.

3005, 11533.333, 0., 2920.

3006, 11066.667, 0., 2920.

3007, 10600., 0., 2920.

3008, 10133.333, 0., 2920.

3009, 9666.66699, 0., 2920.

3010, 9666.66699, 0., 2700.

3011, 10133.333, 0., 2700.

3012, 10600., 0., 2700.

3013, 11066.667, 0., 2700.

3014, 11533.333, 0., 2700.

3015, 9666.66699, 200., 2920.

3016, 10133.333, 200., 2920.

3017, 10600., 200., 2920.

3018, 11066.667, 200., 2920.

3019, 11533.333, 200., 2920.

3020, 11533.333, 200., 2700.

3021, 11066.667, 200., 2700.

3022, 10600., 200., 2700.

3023, 10133.333, 200., 2700.

3024, 9666.66699, 200., 2700.

3025, 9666.66699, 3200., 2920.

3026, 10133.333, 3200., 2920.

3027, 10600., 3200., 2920.

3028, 11066.667, 3200., 2920.

3029, 11533.333, 3200., 2920.

3030, 11533.333, 3200., 2700.

3031, 11066.667, 3200., 2700.

3032, 10600., 3200., 2700.

3033, 10133.333, 3200., 2700.

3034, 9666.66699, 3200., 2700.

3035, 11533.333, 3000., 2920.

3036, 11066.667, 3000., 2920.

3037, 10600., 3000., 2920.

3038, 10133.333, 3000., 2920.

3039, 9666.66699, 3000., 2920.

3040, 9666.66699, 3000., 2700.

3041, 10133.333, 3000., 2700.

3042, 10600., 3000., 2700.

3043, 11066.667, 3000., 2700.

3044, 11533.333, 3000., 2700.

3045, 11533.333, 9000., 2920.

3046, 11066.667, 9000., 2920.

3047, 10600., 9000., 2920.

3048, 10133.333, 9000., 2920.

3049, 9666.66699, 9000., 2920.

3050, 9666.66699, 9000., 2700.

3051, 10133.333, 9000., 2700.

3052, 10600., 9000., 2700.

3053, 11066.667, 9000., 2700.

3054, 11533.333, 9000., 2700.

3055, 9666.66699, 9200., 2920.

3056, 10133.333, 9200., 2920.

3057, 10600., 9200., 2920.

3058, 11066.667, 9200., 2920.

3059, 11533.333, 9200., 2920.

3060, 11533.333, 9200., 2700.

3061, 11066.667, 9200., 2700.

3062, 10600., 9200., 2700.

3063, 10133.333, 9200., 2700.

3064, 9666.66699, 9200., 2700.

3065, 11533.333, 12000., 2920.

3066, 11066.667, 12000., 2920.

3067, 10600., 12000., 2920.

3068, 10133.333, 12000., 2920.

3069, 9666.66699, 12000., 2920.

3070, 9666.66699, 12000., 2700.

3071, 10133.333, 12000., 2700.

3072, 10600., 12000., 2700.

3073, 11066.667, 12000., 2700.

3074, 11533.333, 12000., 2700.

3075, 9666.66699, 12200., 2920.

3076, 10133.333, 12200., 2920.

3077, 10600., 12200., 2920.

3078, 11066.667, 12200., 2920.

3079, 11533.333, 12200., 2920.

3080, 11533.333, 12200., 2700.

3081, 11066.667, 12200., 2700.

3082, 10600., 12200., 2700.

3083, 10133.333, 12200., 2700.

3084, 9666.66699, 12200., 2700.

3085, 11533.333, 15000., 2920.

3086, 11066.667, 15000., 2920.

3087, 10600., 15000., 2920.

3088, 10133.333, 15000., 2920.

3089, 9666.66699, 15000., 2920.

3090, 9666.66699, 15000., 2700.

3091, 10133.333, 15000., 2700.

3092, 10600., 15000., 2700.

3093, 11066.667, 15000., 2700.

3094, 11533.333, 15000., 2700.

3095, 9666.66699, 15200., 2920.

3096, 10133.333, 15200., 2920.

3097, 10600., 15200., 2920.

3098, 11066.667, 15200., 2920.

3099, 11533.333, 15200., 2920.

3100, 11533.333, 15200., 2700.

3101, 11066.667, 15200., 2700.

3102, 10600., 15200., 2700.

3103, 10133.333, 15200., 2700.

3104, 9666.66699, 15200., 2700.

3105, 9666.66699, 6200., 2920.

3106, 10133.333, 6200., 2920.

3107, 10600., 6200., 2920.

3108, 11066.667, 6200., 2920.

3109, 11533.333, 6200., 2920.

3110, 11533.333, 6200., 2700.

3111, 11066.667, 6200., 2700.

3112, 10600., 6200., 2700.

3113, 10133.333, 6200., 2700.

3114, 9666.66699, 6200., 2700.

3115, 11533.333, 6000., 2920.

3116, 11066.667, 6000., 2920.

3117, 10600., 6000., 2920.

3118, 10133.333, 6000., 2920.

3119, 9666.66699, 6000., 2920.

3120, 9666.66699, 6000., 2700.

3121, 10133.333, 6000., 2700.

3122, 10600., 6000., 2700.

3123, 11066.667, 6000., 2700.

3124, 11533.333, 6000., 2700.

3125, 8533.33301, 0., 2920.

3126, 8066.6665, 0., 2920.

3127, 7600., 0., 2920.

3128, 7133.3335, 0., 2920.

3129, 6666.6665, 0., 2920.

3130, 6666.6665, 0., 2700.

3131, 7133.3335, 0., 2700.

3132, 7600., 0., 2700.

3133, 8066.6665, 0., 2700.

3134, 8533.33301, 0., 2700.

3135, 6666.6665, 200., 2920.

3136, 7133.3335, 200., 2920.

3137, 7600., 200., 2920.

3138, 8066.6665, 200., 2920.

3139, 8533.33301, 200., 2920.

3140, 8533.33301, 200., 2700.

3141, 8066.6665, 200., 2700.

3142, 7600., 200., 2700.

3143, 7133.3335, 200., 2700.

3144, 6666.6665, 200., 2700.

3145, 6666.6665, 3200., 2920.

3146, 7133.3335, 3200., 2920.

3147, 7600., 3200., 2920.

3148, 8066.6665, 3200., 2920.

3149, 8533.33301, 3200., 2920.

3150, 8533.33301, 3200., 2700.

3151, 8066.6665, 3200., 2700.

3152, 7600., 3200., 2700.

3153, 7133.3335, 3200., 2700.

3154, 6666.6665, 3200., 2700.

3155, 8533.33301, 3000., 2920.

3156, 8066.6665, 3000., 2920.

3157, 7600., 3000., 2920.

3158, 7133.3335, 3000., 2920.

3159, 6666.6665, 3000., 2920.

3160, 6666.6665, 3000., 2700.

3161, 7133.3335, 3000., 2700.

3162, 7600., 3000., 2700.

3163, 8066.6665, 3000., 2700.

3164, 8533.33301, 3000., 2700.

3165, 8533.33301, 6000., 2920.

3166, 8066.6665, 6000., 2920.

3167, 7600., 6000., 2920.

3168, 7133.3335, 6000., 2920.

3169, 6666.6665, 6000., 2920.

3170, 6666.6665, 6000., 2700.

3171, 7133.3335, 6000., 2700.

3172, 7600., 6000., 2700.

3173, 8066.6665, 6000., 2700.

3174, 8533.33301, 6000., 2700.

3175, 6666.6665, 6200., 2920.

3176, 7133.3335, 6200., 2920.

3177, 7600., 6200., 2920.

3178, 8066.6665, 6200., 2920.

3179, 8533.33301, 6200., 2920.

3180, 8533.33301, 6200., 2700.

3181, 8066.6665, 6200., 2700.

3182, 7600., 6200., 2700.

3183, 7133.3335, 6200., 2700.

3184, 6666.6665, 6200., 2700.

3185, 8533.33301, 9000., 2920.

3186, 8066.6665, 9000., 2920.

3187, 7600., 9000., 2920.

3188, 7133.3335, 9000., 2920.

3189, 6666.6665, 9000., 2920.

3190, 6666.6665, 9000., 2700.

3191, 7133.3335, 9000., 2700.

3192, 7600., 9000., 2700.

3193, 8066.6665, 9000., 2700.

3194, 8533.33301, 9000., 2700.

3195, 6666.6665, 9200., 2920.

3196, 7133.3335, 9200., 2920.

3197, 7600., 9200., 2920.

3198, 8066.6665, 9200., 2920.

3199, 8533.33301, 9200., 2920.

3200, 8533.33301, 9200., 2700.

3201, 8066.6665, 9200., 2700.

3202, 7600., 9200., 2700.

3203, 7133.3335, 9200., 2700.

3204, 6666.6665, 9200., 2700.

3205, 8533.33301, 12000., 2920.

3206, 8066.6665, 12000., 2920.

3207, 7600., 12000., 2920.

3208, 7133.3335, 12000., 2920.

3209, 6666.6665, 12000., 2920.

3210, 6666.6665, 12000., 2700.

3211, 7133.3335, 12000., 2700.

3212, 7600., 12000., 2700.

3213, 8066.6665, 12000., 2700.

3214, 8533.33301, 12000., 2700.

3215, 6666.6665, 12200., 2920.

3216, 7133.3335, 12200., 2920.

3217, 7600., 12200., 2920.

3218, 8066.6665, 12200., 2920.

3219, 8533.33301, 12200., 2920.

3220, 8533.33301, 12200., 2700.

3221, 8066.6665, 12200., 2700.

3222, 7600., 12200., 2700.

3223, 7133.3335, 12200., 2700.

3224, 6666.6665, 12200., 2700.

3225, 6666.6665, 15200., 2920.

3226, 7133.3335, 15200., 2920.

3227, 7600., 15200., 2920.

3228, 8066.6665, 15200., 2920.

3229, 8533.33301, 15200., 2920.

3230, 8533.33301, 15000., 2920.

3231, 8066.6665, 15000., 2920.

3232, 7600., 15000., 2920.

3233, 7133.3335, 15000., 2920.

3234, 6666.6665, 15000., 2920.

3235, 9000., 14533.333, 2920.

3236, 9000., 14066.667, 2920.

3237, 9000., 13600., 2920.

3238, 9000., 13133.333, 2920.

3239, 9000., 12666.667, 2920.

3240, 6200., 12666.667, 2920.

3241, 6200., 13133.333, 2920.

3242, 6200., 13600., 2920.

3243, 6200., 14066.667, 2920.

3244, 6200., 14533.333, 2920.

3245, 9000., 11533.333, 2920.

3246, 9000., 11066.667, 2920.

3247, 9000., 10600., 2920.

3248, 9000., 10133.333, 2920.

3249, 9000., 9666.66699, 2920.

3250, 6200., 9666.66699, 2920.

3251, 6200., 10133.333, 2920.

3252, 6200., 10600., 2920.

3253, 6200., 11066.667, 2920.

3254, 6200., 11533.333, 2920.

3255, 9000., 8533.33301, 2920.

3256, 9000., 8066.6665, 2920.

3257, 9000., 7600., 2920.

3258, 9000., 7133.3335, 2920.

3259, 9000., 6666.6665, 2920.

3260, 6200., 6666.6665, 2920.

3261, 6200., 7133.3335, 2920.

3262, 6200., 7600., 2920.

3263, 6200., 8066.6665, 2920.

3264, 6200., 8533.33301, 2920.

3265, 9000., 5533.3335, 2920.

3266, 9000., 5066.6665, 2920.

3267, 9000., 4600., 2920.

3268, 9000., 4133.3335, 2920.

3269, 9000., 3666.66675, 2920.

3270, 6200., 3666.66675, 2920.

3271, 6200., 4133.3335, 2920.

3272, 6200., 4600., 2920.

3273, 6200., 5066.6665, 2920.

3274, 6200., 5533.3335, 2920.

3275, 6200., 666.666687, 2920.

3276, 6200., 1133.33337, 2920.

3277, 6200., 1600., 2920.

3278, 6200., 2066.66675, 2920.

3279, 6200., 2533.33325, 2920.

3280, 9000., 2533.33325, 2920.

3281, 9000., 2066.66675, 2920.

3282, 9000., 1600., 2920.

3283, 9000., 1133.33337, 2920.

3284, 9000., 666.666687, 2920.

3285, 8533.33301, 15200., 3000.

3286, 8066.6665, 15200., 3000.

3287, 7600., 15200., 3000.

3288, 7133.3335, 15200., 3000.

3289, 6666.6665, 15200., 3000.

3290, 9000., 12666.667, 3000.

3291, 9000., 13133.333, 3000.

3292, 9000., 13600., 3000.

3293, 9000., 14066.667, 3000.

3294, 9000., 14533.333, 3000.

3295, 9000., 9666.66699, 3000.

3296, 9000., 10133.333, 3000.

3297, 9000., 10600., 3000.

3298, 9000., 11066.667, 3000.

3299, 9000., 11533.333, 3000.

3300, 9000., 6666.6665, 3000.

3301, 9000., 7133.3335, 3000.

3302, 9000., 7600., 3000.

3303, 9000., 8066.6665, 3000.

3304, 9000., 8533.33301, 3000.

3305, 9000., 3666.66675, 3000.

3306, 9000., 4133.3335, 3000.

3307, 9000., 4600., 3000.

3308, 9000., 5066.6665, 3000.

3309, 9000., 5533.3335, 3000.

3310, 9000., 666.666687, 3000.

3311, 9000., 1133.33337, 3000.

3312, 9000., 1600., 3000.

3313, 9000., 2066.66675, 3000.

3314, 9000., 2533.33325, 3000.

3315, 6666.6665, 0., 3000.

3316, 7133.3335, 0., 3000.

3317, 7600., 0., 3000.

3318, 8066.6665, 0., 3000.

3319, 8533.33301, 0., 3000.

3320, 6200., 14533.333, 3000.

3321, 6200., 14066.667, 3000.

3322, 6200., 13600., 3000.

3323, 6200., 13133.333, 3000.

3324, 6200., 12666.667, 3000.

3325, 6200., 11533.333, 3000.

3326, 6200., 11066.667, 3000.

3327, 6200., 10600., 3000.

3328, 6200., 10133.333, 3000.

3329, 6200., 9666.66699, 3000.

3330, 6200., 8533.33301, 3000.

3331, 6200., 8066.6665, 3000.

3332, 6200., 7600., 3000.

3333, 6200., 7133.3335, 3000.

3334, 6200., 6666.6665, 3000.

3335, 6200., 5533.3335, 3000.

3336, 6200., 5066.6665, 3000.

3337, 6200., 4600., 3000.

3338, 6200., 4133.3335, 3000.

3339, 6200., 3666.66675, 3000.

3340, 6200., 2533.33325, 3000.

3341, 6200., 2066.66675, 3000.

3342, 6200., 1600., 3000.

3343, 6200., 1133.33337, 3000.

3344, 6200., 666.666687, 3000.

3345, 6666.6665, 15200., 5920.

3346, 7133.3335, 15200., 5920.

3347, 7600., 15200., 5920.

3348, 8066.6665, 15200., 5920.

3349, 8533.33301, 15200., 5920.

3350, 8533.33301, 15200., 5700.

3351, 8066.6665, 15200., 5700.

3352, 7600., 15200., 5700.

3353, 7133.3335, 15200., 5700.

3354, 6666.6665, 15200., 5700.

3355, 8533.33301, 15000., 5920.

3356, 8066.6665, 15000., 5920.

3357, 7600., 15000., 5920.

3358, 7133.3335, 15000., 5920.

3359, 6666.6665, 15000., 5920.

3360, 6666.6665, 15000., 5700.

3361, 7133.3335, 15000., 5700.

3362, 7600., 15000., 5700.

3363, 8066.6665, 15000., 5700.

3364, 8533.33301, 15000., 5700.

3365, 8533.33301, 12000., 5920.

3366, 8066.6665, 12000., 5920.

3367, 7600., 12000., 5920.

3368, 7133.3335, 12000., 5920.

3369, 6666.6665, 12000., 5920.

3370, 6666.6665, 12000., 5700.

3371, 7133.3335, 12000., 5700.

3372, 7600., 12000., 5700.

3373, 8066.6665, 12000., 5700.

3374, 8533.33301, 12000., 5700.

3375, 6666.6665, 12200., 5920.

3376, 7133.3335, 12200., 5920.

3377, 7600., 12200., 5920.

3378, 8066.6665, 12200., 5920.

3379, 8533.33301, 12200., 5920.

3380, 8533.33301, 12200., 5700.

3381, 8066.6665, 12200., 5700.

3382, 7600., 12200., 5700.

3383, 7133.3335, 12200., 5700.

3384, 6666.6665, 12200., 5700.

3385, 8533.33301, 0., 5700.

3386, 8066.6665, 0., 5700.

3387, 7600., 0., 5700.

3388, 7133.3335, 0., 5700.

3389, 6666.6665, 0., 5700.

3390, 6666.6665, 200., 5700.

3391, 7133.3335, 200., 5700.

3392, 7600., 200., 5700.

3393, 8066.6665, 200., 5700.

3394, 8533.33301, 200., 5700.

3395, 8533.33301, 0., 5920.

3396, 8066.6665, 0., 5920.

3397, 7600., 0., 5920.

3398, 7133.3335, 0., 5920.

3399, 6666.6665, 0., 5920.

3400, 6666.6665, 200., 5920.

3401, 7133.3335, 200., 5920.

3402, 7600., 200., 5920.

3403, 8066.6665, 200., 5920.

3404, 8533.33301, 200., 5920.

3405, 8533.33301, 3000., 5920.

3406, 8066.6665, 3000., 5920.

3407, 7600., 3000., 5920.

3408, 7133.3335, 3000., 5920.

3409, 6666.6665, 3000., 5920.

3410, 6666.6665, 3000., 5700.

3411, 7133.3335, 3000., 5700.

3412, 7600., 3000., 5700.

3413, 8066.6665, 3000., 5700.

3414, 8533.33301, 3000., 5700.

3415, 6666.6665, 3200., 5920.

3416, 7133.3335, 3200., 5920.

3417, 7600., 3200., 5920.

3418, 8066.6665, 3200., 5920.

3419, 8533.33301, 3200., 5920.

3420, 8533.33301, 3200., 5700.

3421, 8066.6665, 3200., 5700.

3422, 7600., 3200., 5700.

3423, 7133.3335, 3200., 5700.

3424, 6666.6665, 3200., 5700.

3425, 8533.33301, 6000., 5920.

3426, 8066.6665, 6000., 5920.

3427, 7600., 6000., 5920.

3428, 7133.3335, 6000., 5920.

3429, 6666.6665, 6000., 5920.

3430, 6666.6665, 6000., 5700.

3431, 7133.3335, 6000., 5700.

3432, 7600., 6000., 5700.

3433, 8066.6665, 6000., 5700.

3434, 8533.33301, 6000., 5700.

3435, 6666.6665, 6200., 5920.

3436, 7133.3335, 6200., 5920.

3437, 7600., 6200., 5920.

3438, 8066.6665, 6200., 5920.

3439, 8533.33301, 6200., 5920.

3440, 8533.33301, 6200., 5700.

3441, 8066.6665, 6200., 5700.

3442, 7600., 6200., 5700.

3443, 7133.3335, 6200., 5700.

3444, 6666.6665, 6200., 5700.

3445, 8533.33301, 9000., 5920.

3446, 8066.6665, 9000., 5920.

3447, 7600., 9000., 5920.

3448, 7133.3335, 9000., 5920.

3449, 6666.6665, 9000., 5920.

3450, 6666.6665, 9000., 5700.

3451, 7133.3335, 9000., 5700.

3452, 7600., 9000., 5700.

3453, 8066.6665, 9000., 5700.

3454, 8533.33301, 9000., 5700.

3455, 6666.6665, 9200., 5920.

3456, 7133.3335, 9200., 5920.

3457, 7600., 9200., 5920.

3458, 8066.6665, 9200., 5920.

3459, 8533.33301, 9200., 5920.

3460, 8533.33301, 9200., 5700.

3461, 8066.6665, 9200., 5700.

3462, 7600., 9200., 5700.

3463, 7133.3335, 9200., 5700.

3464, 6666.6665, 9200., 5700.

3465, 11533.333, 0., 5920.

3466, 11066.667, 0., 5920.

3467, 10600., 0., 5920.

3468, 10133.333, 0., 5920.

3469, 9666.66699, 0., 5920.

3470, 9666.66699, 0., 5700.

3471, 10133.333, 0., 5700.

3472, 10600., 0., 5700.

3473, 11066.667, 0., 5700.

3474, 11533.333, 0., 5700.

3475, 9666.66699, 200., 5920.

3476, 10133.333, 200., 5920.

3477, 10600., 200., 5920.

3478, 11066.667, 200., 5920.

3479, 11533.333, 200., 5920.

3480, 11533.333, 200., 5700.

3481, 11066.667, 200., 5700.

3482, 10600., 200., 5700.

3483, 10133.333, 200., 5700.

3484, 9666.66699, 200., 5700.

3485, 9666.66699, 3200., 5920.

3486, 10133.333, 3200., 5920.

3487, 10600., 3200., 5920.

3488, 11066.667, 3200., 5920.

3489, 11533.333, 3200., 5920.

3490, 11533.333, 3200., 5700.

3491, 11066.667, 3200., 5700.

3492, 10600., 3200., 5700.

3493, 10133.333, 3200., 5700.

3494, 9666.66699, 3200., 5700.

3495, 11533.333, 3000., 5920.

3496, 11066.667, 3000., 5920.

3497, 10600., 3000., 5920.

3498, 10133.333, 3000., 5920.

3499, 9666.66699, 3000., 5920.

3500, 9666.66699, 3000., 5700.

3501, 10133.333, 3000., 5700.

3502, 10600., 3000., 5700.

3503, 11066.667, 3000., 5700.

3504, 11533.333, 3000., 5700.

3505, 11533.333, 12000., 5920.

3506, 11066.667, 12000., 5920.

3507, 10600., 12000., 5920.

3508, 10133.333, 12000., 5920.

3509, 9666.66699, 12000., 5920.

3510, 9666.66699, 12000., 5700.

3511, 10133.333, 12000., 5700.

3512, 10600., 12000., 5700.

3513, 11066.667, 12000., 5700.

3514, 11533.333, 12000., 5700.

3515, 9666.66699, 12200., 5920.

3516, 10133.333, 12200., 5920.

3517, 10600., 12200., 5920.

3518, 11066.667, 12200., 5920.

3519, 11533.333, 12200., 5920.

3520, 11533.333, 12200., 5700.

3521, 11066.667, 12200., 5700.

3522, 10600., 12200., 5700.

3523, 10133.333, 12200., 5700.

3524, 9666.66699, 12200., 5700.

3525, 11533.333, 15000., 5920.

3526, 11066.667, 15000., 5920.

3527, 10600., 15000., 5920.

3528, 10133.333, 15000., 5920.

3529, 9666.66699, 15000., 5920.

3530, 9666.66699, 15000., 5700.

3531, 10133.333, 15000., 5700.

3532, 10600., 15000., 5700.

3533, 11066.667, 15000., 5700.

3534, 11533.333, 15000., 5700.

3535, 9666.66699, 15200., 5920.

3536, 10133.333, 15200., 5920.

3537, 10600., 15200., 5920.

3538, 11066.667, 15200., 5920.

3539, 11533.333, 15200., 5920.

3540, 11533.333, 15200., 5700.

3541, 11066.667, 15200., 5700.

3542, 10600., 15200., 5700.

3543, 10133.333, 15200., 5700.

3544, 9666.66699, 15200., 5700.

3545, 9666.66699, 9200., 5920.

3546, 10133.333, 9200., 5920.

3547, 10600., 9200., 5920.

3548, 11066.667, 9200., 5920.

3549, 11533.333, 9200., 5920.

3550, 11533.333, 9200., 5700.

3551, 11066.667, 9200., 5700.

3552, 10600., 9200., 5700.

3553, 10133.333, 9200., 5700.

3554, 9666.66699, 9200., 5700.

3555, 11533.333, 9000., 5920.

3556, 11066.667, 9000., 5920.

3557, 10600., 9000., 5920.

3558, 10133.333, 9000., 5920.

3559, 9666.66699, 9000., 5920.

3560, 9666.66699, 9000., 5700.

3561, 10133.333, 9000., 5700.

3562, 10600., 9000., 5700.

3563, 11066.667, 9000., 5700.

3564, 11533.333, 9000., 5700.

3565, 9666.66699, 6200., 5920.

3566, 10133.333, 6200., 5920.

3567, 10600., 6200., 5920.

3568, 11066.667, 6200., 5920.

3569, 11533.333, 6200., 5920.

3570, 11533.333, 6200., 5700.

3571, 11066.667, 6200., 5700.

3572, 10600., 6200., 5700.

3573, 10133.333, 6200., 5700.

3574, 9666.66699, 6200., 5700.

3575, 11533.333, 6000., 5920.

3576, 11066.667, 6000., 5920.

3577, 10600., 6000., 5920.

3578, 10133.333, 6000., 5920.

3579, 9666.66699, 6000., 5920.

3580, 9666.66699, 6000., 5700.

3581, 10133.333, 6000., 5700.

3582, 10600., 6000., 5700.

3583, 11066.667, 6000., 5700.

3584, 11533.333, 6000., 5700.

3585, 14533.333, 0., 2920.

3586, 14066.667, 0., 2920.

3587, 13600., 0., 2920.

3588, 13133.333, 0., 2920.

3589, 12666.667, 0., 2920.

3590, 12666.667, 0., 2700.

3591, 13133.333, 0., 2700.

3592, 13600., 0., 2700.

3593, 14066.667, 0., 2700.

3594, 14533.333, 0., 2700.

3595, 12666.667, 200., 2920.

3596, 13133.333, 200., 2920.

3597, 13600., 200., 2920.

3598, 14066.667, 200., 2920.

3599, 14533.333, 200., 2920.

3600, 14533.333, 200., 2700.

3601, 14066.667, 200., 2700.

3602, 13600., 200., 2700.

3603, 13133.333, 200., 2700.

3604, 12666.667, 200., 2700.

3605, 12666.667, 3200., 2920.

3606, 13133.333, 3200., 2920.

3607, 13600., 3200., 2920.

3608, 14066.667, 3200., 2920.

3609, 14533.333, 3200., 2920.

3610, 14533.333, 3200., 2700.

3611, 14066.667, 3200., 2700.

3612, 13600., 3200., 2700.

3613, 13133.333, 3200., 2700.

3614, 12666.667, 3200., 2700.

3615, 14533.333, 3000., 2920.

3616, 14066.667, 3000., 2920.

3617, 13600., 3000., 2920.

3618, 13133.333, 3000., 2920.

3619, 12666.667, 3000., 2920.

3620, 12666.667, 3000., 2700.

3621, 13133.333, 3000., 2700.

3622, 13600., 3000., 2700.

3623, 14066.667, 3000., 2700.

3624, 14533.333, 3000., 2700.

3625, 14533.333, 6000., 2920.

3626, 14066.667, 6000., 2920.

3627, 13600., 6000., 2920.

3628, 13133.333, 6000., 2920.

3629, 12666.667, 6000., 2920.

3630, 12666.667, 6000., 2700.

3631, 13133.333, 6000., 2700.

3632, 13600., 6000., 2700.

3633, 14066.667, 6000., 2700.

3634, 14533.333, 6000., 2700.

3635, 12666.667, 6200., 2920.

3636, 13133.333, 6200., 2920.

3637, 13600., 6200., 2920.

3638, 14066.667, 6200., 2920.

3639, 14533.333, 6200., 2920.

3640, 14533.333, 6200., 2700.

3641, 14066.667, 6200., 2700.

3642, 13600., 6200., 2700.

3643, 13133.333, 6200., 2700.

3644, 12666.667, 6200., 2700.

3645, 14533.333, 9000., 2920.

3646, 14066.667, 9000., 2920.

3647, 13600., 9000., 2920.

3648, 13133.333, 9000., 2920.

3649, 12666.667, 9000., 2920.

3650, 12666.667, 9000., 2700.

3651, 13133.333, 9000., 2700.

3652, 13600., 9000., 2700.

3653, 14066.667, 9000., 2700.

3654, 14533.333, 9000., 2700.

3655, 12666.667, 9200., 2920.

3656, 13133.333, 9200., 2920.

3657, 13600., 9200., 2920.

3658, 14066.667, 9200., 2920.

3659, 14533.333, 9200., 2920.

3660, 14533.333, 9200., 2700.

3661, 14066.667, 9200., 2700.

3662, 13600., 9200., 2700.

3663, 13133.333, 9200., 2700.

3664, 12666.667, 9200., 2700.

3665, 14533.333, 12000., 2920.

3666, 14066.667, 12000., 2920.

3667, 13600., 12000., 2920.

3668, 13133.333, 12000., 2920.

3669, 12666.667, 12000., 2920.

3670, 12666.667, 12000., 2700.

3671, 13133.333, 12000., 2700.

3672, 13600., 12000., 2700.

3673, 14066.667, 12000., 2700.

3674, 14533.333, 12000., 2700.

3675, 12666.667, 12200., 2920.

3676, 13133.333, 12200., 2920.

3677, 13600., 12200., 2920.

3678, 14066.667, 12200., 2920.

3679, 14533.333, 12200., 2920.

3680, 14533.333, 12200., 2700.

3681, 14066.667, 12200., 2700.

3682, 13600., 12200., 2700.

3683, 13133.333, 12200., 2700.

3684, 12666.667, 12200., 2700.

3685, 12666.667, 15200., 2920.

3686, 13133.333, 15200., 2920.

3687, 13600., 15200., 2920.

3688, 14066.667, 15200., 2920.

3689, 14533.333, 15200., 2920.

3690, 14533.333, 15000., 2920.

3691, 14066.667, 15000., 2920.

3692, 13600., 15000., 2920.

3693, 13133.333, 15000., 2920.

3694, 12666.667, 15000., 2920.

3695, 15000., 14533.333, 2920.

3696, 15000., 14066.667, 2920.

3697, 15000., 13600., 2920.

3698, 15000., 13133.333, 2920.

3699, 15000., 12666.667, 2920.

3700, 12200., 12666.667, 2920.

3701, 12200., 13133.333, 2920.

3702, 12200., 13600., 2920.

3703, 12200., 14066.667, 2920.

3704, 12200., 14533.333, 2920.

3705, 15000., 11533.333, 2920.

3706, 15000., 11066.667, 2920.

3707, 15000., 10600., 2920.

3708, 15000., 10133.333, 2920.

3709, 15000., 9666.66699, 2920.

3710, 12200., 9666.66699, 2920.

3711, 12200., 10133.333, 2920.

3712, 12200., 10600., 2920.

3713, 12200., 11066.667, 2920.

3714, 12200., 11533.333, 2920.

3715, 15000., 8533.33301, 2920.

3716, 15000., 8066.6665, 2920.

3717, 15000., 7600., 2920.

3718, 15000., 7133.3335, 2920.

3719, 15000., 6666.6665, 2920.

3720, 12200., 6666.6665, 2920.

3721, 12200., 7133.3335, 2920.

3722, 12200., 7600., 2920.

3723, 12200., 8066.6665, 2920.

3724, 12200., 8533.33301, 2920.

3725, 15000., 5533.3335, 2920.

3726, 15000., 5066.6665, 2920.

3727, 15000., 4600., 2920.

3728, 15000., 4133.3335, 2920.

3729, 15000., 3666.66675, 2920.

3730, 12200., 3666.66675, 2920.

3731, 12200., 4133.3335, 2920.

3732, 12200., 4600., 2920.

3733, 12200., 5066.6665, 2920.

3734, 12200., 5533.3335, 2920.

3735, 12200., 666.666687, 2920.

3736, 12200., 1133.33337, 2920.

3737, 12200., 1600., 2920.

3738, 12200., 2066.66675, 2920.

3739, 12200., 2533.33325, 2920.

3740, 15000., 2533.33325, 2920.

3741, 15000., 2066.66675, 2920.

3742, 15000., 1600., 2920.

3743, 15000., 1133.33337, 2920.

3744, 15000., 666.666687, 2920.

3745, 14533.333, 15200., 3000.

3746, 14066.667, 15200., 3000.

3747, 13600., 15200., 3000.

3748, 13133.333, 15200., 3000.

3749, 12666.667, 15200., 3000.

3750, 15000., 12666.667, 3000.

3751, 15000., 13133.333, 3000.

3752, 15000., 13600., 3000.

3753, 15000., 14066.667, 3000.

3754, 15000., 14533.333, 3000.

3755, 15000., 9666.66699, 3000.

3756, 15000., 10133.333, 3000.

3757, 15000., 10600., 3000.

3758, 15000., 11066.667, 3000.

3759, 15000., 11533.333, 3000.

3760, 15000., 6666.6665, 3000.

3761, 15000., 7133.3335, 3000.

3762, 15000., 7600., 3000.

3763, 15000., 8066.6665, 3000.

3764, 15000., 8533.33301, 3000.

3765, 15000., 3666.66675, 3000.

3766, 15000., 4133.3335, 3000.

3767, 15000., 4600., 3000.

3768, 15000., 5066.6665, 3000.

3769, 15000., 5533.3335, 3000.

3770, 15000., 666.666687, 3000.

3771, 15000., 1133.33337, 3000.

3772, 15000., 1600., 3000.

3773, 15000., 2066.66675, 3000.

3774, 15000., 2533.33325, 3000.

3775, 12666.667, 0., 3000.

3776, 13133.333, 0., 3000.

3777, 13600., 0., 3000.

3778, 14066.667, 0., 3000.

3779, 14533.333, 0., 3000.

3780, 12200., 14533.333, 3000.

3781, 12200., 14066.667, 3000.

3782, 12200., 13600., 3000.

3783, 12200., 13133.333, 3000.

3784, 12200., 12666.667, 3000.

3785, 12200., 11533.333, 3000.

3786, 12200., 11066.667, 3000.

3787, 12200., 10600., 3000.

3788, 12200., 10133.333, 3000.

3789, 12200., 9666.66699, 3000.

3790, 12200., 8533.33301, 3000.

3791, 12200., 8066.6665, 3000.

3792, 12200., 7600., 3000.

3793, 12200., 7133.3335, 3000.

3794, 12200., 6666.6665, 3000.

3795, 12200., 5533.3335, 3000.

3796, 12200., 5066.6665, 3000.

3797, 12200., 4600., 3000.

3798, 12200., 4133.3335, 3000.

3799, 12200., 3666.66675, 3000.

3800, 12200., 2533.33325, 3000.

3801, 12200., 2066.66675, 3000.

3802, 12200., 1600., 3000.

3803, 12200., 1133.33337, 3000.

3804, 12200., 666.666687, 3000.

3805, 14533.333, 0., 5920.

3806, 14066.667, 0., 5920.

3807, 13600., 0., 5920.

3808, 13133.333, 0., 5920.

3809, 12666.667, 0., 5920.

3810, 12666.667, 0., 5700.

3811, 13133.333, 0., 5700.

3812, 13600., 0., 5700.

3813, 14066.667, 0., 5700.

3814, 14533.333, 0., 5700.

3815, 12666.667, 200., 5920.

3816, 13133.333, 200., 5920.

3817, 13600., 200., 5920.

3818, 14066.667, 200., 5920.

3819, 14533.333, 200., 5920.

3820, 14533.333, 200., 5700.

3821, 14066.667, 200., 5700.

3822, 13600., 200., 5700.

3823, 13133.333, 200., 5700.

3824, 12666.667, 200., 5700.

3825, 12666.667, 3200., 5920.

3826, 13133.333, 3200., 5920.

3827, 13600., 3200., 5920.

3828, 14066.667, 3200., 5920.

3829, 14533.333, 3200., 5920.

3830, 14533.333, 3200., 5700.

3831, 14066.667, 3200., 5700.

3832, 13600., 3200., 5700.

3833, 13133.333, 3200., 5700.

3834, 12666.667, 3200., 5700.

3835, 14533.333, 3000., 5920.

3836, 14066.667, 3000., 5920.

3837, 13600., 3000., 5920.

3838, 13133.333, 3000., 5920.

3839, 12666.667, 3000., 5920.

3840, 12666.667, 3000., 5700.

3841, 13133.333, 3000., 5700.

3842, 13600., 3000., 5700.

3843, 14066.667, 3000., 5700.

3844, 14533.333, 3000., 5700.

3845, 14533.333, 6000., 5920.

3846, 14066.667, 6000., 5920.

3847, 13600., 6000., 5920.

3848, 13133.333, 6000., 5920.

3849, 12666.667, 6000., 5920.

3850, 12666.667, 6000., 5700.

3851, 13133.333, 6000., 5700.

3852, 13600., 6000., 5700.

3853, 14066.667, 6000., 5700.

3854, 14533.333, 6000., 5700.

3855, 12666.667, 6200., 5920.

3856, 13133.333, 6200., 5920.

3857, 13600., 6200., 5920.

3858, 14066.667, 6200., 5920.

3859, 14533.333, 6200., 5920.

3860, 14533.333, 6200., 5700.

3861, 14066.667, 6200., 5700.

3862, 13600., 6200., 5700.

3863, 13133.333, 6200., 5700.

3864, 12666.667, 6200., 5700.

3865, 14533.333, 9000., 5920.

3866, 14066.667, 9000., 5920.

3867, 13600., 9000., 5920.

3868, 13133.333, 9000., 5920.

3869, 12666.667, 9000., 5920.

3870, 12666.667, 9000., 5700.

3871, 13133.333, 9000., 5700.

3872, 13600., 9000., 5700.

3873, 14066.667, 9000., 5700.

3874, 14533.333, 9000., 5700.

3875, 12666.667, 9200., 5920.

3876, 13133.333, 9200., 5920.

3877, 13600., 9200., 5920.

3878, 14066.667, 9200., 5920.

3879, 14533.333, 9200., 5920.

3880, 14533.333, 9200., 5700.

3881, 14066.667, 9200., 5700.

3882, 13600., 9200., 5700.

3883, 13133.333, 9200., 5700.

3884, 12666.667, 9200., 5700.

3885, 14533.333, 12000., 5920.

3886, 14066.667, 12000., 5920.

3887, 13600., 12000., 5920.

3888, 13133.333, 12000., 5920.

3889, 12666.667, 12000., 5920.

3890, 12666.667, 12000., 5700.

3891, 13133.333, 12000., 5700.

3892, 13600., 12000., 5700.

3893, 14066.667, 12000., 5700.

3894, 14533.333, 12000., 5700.

3895, 12666.667, 12200., 5920.

3896, 13133.333, 12200., 5920.

3897, 13600., 12200., 5920.

3898, 14066.667, 12200., 5920.

3899, 14533.333, 12200., 5920.

3900, 14533.333, 12200., 5700.

3901, 14066.667, 12200., 5700.

3902, 13600., 12200., 5700.

3903, 13133.333, 12200., 5700.

3904, 12666.667, 12200., 5700.

3905, 12666.667, 15200., 5920.

3906, 13133.333, 15200., 5920.

3907, 13600., 15200., 5920.

3908, 14066.667, 15200., 5920.

3909, 14533.333, 15200., 5920.

3910, 14533.333, 15000., 5920.

3911, 14066.667, 15000., 5920.

3912, 13600., 15000., 5920.

3913, 13133.333, 15000., 5920.

3914, 12666.667, 15000., 5920.

3915, 15000., 14533.333, 5920.

3916, 15000., 14066.667, 5920.

3917, 15000., 13600., 5920.

3918, 15000., 13133.333, 5920.

3919, 15000., 12666.667, 5920.

3920, 12200., 12666.667, 5920.

3921, 12200., 13133.333, 5920.

3922, 12200., 13600., 5920.

3923, 12200., 14066.667, 5920.

3924, 12200., 14533.333, 5920.

3925, 15000., 11533.333, 5920.

3926, 15000., 11066.667, 5920.

3927, 15000., 10600., 5920.

3928, 15000., 10133.333, 5920.

3929, 15000., 9666.66699, 5920.

3930, 12200., 9666.66699, 5920.

3931, 12200., 10133.333, 5920.

3932, 12200., 10600., 5920.

3933, 12200., 11066.667, 5920.

3934, 12200., 11533.333, 5920.

3935, 15000., 8533.33301, 5920.

3936, 15000., 8066.6665, 5920.

3937, 15000., 7600., 5920.

3938, 15000., 7133.3335, 5920.

3939, 15000., 6666.6665, 5920.

3940, 12200., 6666.6665, 5920.

3941, 12200., 7133.3335, 5920.

3942, 12200., 7600., 5920.

3943, 12200., 8066.6665, 5920.

3944, 12200., 8533.33301, 5920.

3945, 15000., 5533.3335, 5920.

3946, 15000., 5066.6665, 5920.

3947, 15000., 4600., 5920.

3948, 15000., 4133.3335, 5920.

3949, 15000., 3666.66675, 5920.

3950, 12200., 3666.66675, 5920.

3951, 12200., 4133.3335, 5920.

3952, 12200., 4600., 5920.

3953, 12200., 5066.6665, 5920.

3954, 12200., 5533.3335, 5920.

3955, 15000., 2533.33325, 5920.

3956, 15000., 2066.66675, 5920.

3957, 15000., 1600., 5920.

3958, 15000., 1133.33337, 5920.

3959, 15000., 666.666687, 5920.

3960, 12200., 666.666687, 5920.

3961, 12200., 1133.33337, 5920.

3962, 12200., 1600., 5920.

3963, 12200., 2066.66675, 5920.

3964, 12200., 2533.33325, 5920.

3965, 14533.333, 15200., 6000.

3966, 14066.667, 15200., 6000.

3967, 13600., 15200., 6000.

3968, 13133.333, 15200., 6000.

3969, 12666.667, 15200., 6000.

3970, 15000., 12666.667, 6000.

3971, 15000., 13133.333, 6000.

3972, 15000., 13600., 6000.

3973, 15000., 14066.667, 6000.

3974, 15000., 14533.333, 6000.

3975, 15000., 9666.66699, 6000.

3976, 15000., 10133.333, 6000.

3977, 15000., 10600., 6000.

3978, 15000., 11066.667, 6000.

3979, 15000., 11533.333, 6000.

3980, 15000., 6666.6665, 6000.

3981, 15000., 7133.3335, 6000.

3982, 15000., 7600., 6000.

3983, 15000., 8066.6665, 6000.

3984, 15000., 8533.33301, 6000.

3985, 15000., 3666.66675, 6000.

3986, 15000., 4133.3335, 6000.

3987, 15000., 4600., 6000.

3988, 15000., 5066.6665, 6000.

3989, 15000., 5533.3335, 6000.

3990, 15000., 666.666687, 6000.

3991, 15000., 1133.33337, 6000.

3992, 15000., 1600., 6000.

3993, 15000., 2066.66675, 6000.

3994, 15000., 2533.33325, 6000.

3995, 12666.667, 0., 6000.

3996, 13133.333, 0., 6000.

3997, 13600., 0., 6000.

3998, 14066.667, 0., 6000.

3999, 14533.333, 0., 6000.

4000, 12200., 14533.333, 6000.

4001, 12200., 14066.667, 6000.

4002, 12200., 13600., 6000.

4003, 12200., 13133.333, 6000.

4004, 12200., 12666.667, 6000.

4005, 12200., 11533.333, 6000.

4006, 12200., 11066.667, 6000.

4007, 12200., 10600., 6000.

4008, 12200., 10133.333, 6000.

4009, 12200., 9666.66699, 6000.

4010, 12200., 8533.33301, 6000.

4011, 12200., 8066.6665, 6000.

4012, 12200., 7600., 6000.

4013, 12200., 7133.3335, 6000.

4014, 12200., 6666.6665, 6000.

4015, 12200., 5533.3335, 6000.

4016, 12200., 5066.6665, 6000.

4017, 12200., 4600., 6000.

4018, 12200., 4133.3335, 6000.

4019, 12200., 3666.66675, 6000.

4020, 12200., 2533.33325, 6000.

4021, 12200., 2066.66675, 6000.

4022, 12200., 1600., 6000.

4023, 12200., 1133.33337, 6000.

4024, 12200., 666.666687, 6000.

4025, 15666.667, 15200., 8920.

4026, 16133.333, 15200., 8920.

4027, 16600., 15200., 8920.

4028, 17066.666, 15200., 8920.

4029, 17533.334, 15200., 8920.

4030, 17533.334, 15200., 8700.

4031, 17066.666, 15200., 8700.

4032, 16600., 15200., 8700.

4033, 16133.333, 15200., 8700.

4034, 15666.667, 15200., 8700.

4035, 17533.334, 15000., 8920.

4036, 17066.666, 15000., 8920.

4037, 16600., 15000., 8920.

4038, 16133.333, 15000., 8920.

4039, 15666.667, 15000., 8920.

4040, 15666.667, 15000., 8700.

4041, 16133.333, 15000., 8700.

4042, 16600., 15000., 8700.

4043, 17066.666, 15000., 8700.

4044, 17533.334, 15000., 8700.

4045, 17533.334, 12000., 8920.

4046, 17066.666, 12000., 8920.

4047, 16600., 12000., 8920.

4048, 16133.333, 12000., 8920.

4049, 15666.667, 12000., 8920.

4050, 15666.667, 12000., 8700.

4051, 16133.333, 12000., 8700.

4052, 16600., 12000., 8700.

4053, 17066.666, 12000., 8700.

4054, 17533.334, 12000., 8700.

4055, 15666.667, 12200., 8920.

4056, 16133.333, 12200., 8920.

4057, 16600., 12200., 8920.

4058, 17066.666, 12200., 8920.

4059, 17533.334, 12200., 8920.

4060, 17533.334, 12200., 8700.

4061, 17066.666, 12200., 8700.

4062, 16600., 12200., 8700.

4063, 16133.333, 12200., 8700.

4064, 15666.667, 12200., 8700.

4065, 15666.667, 200., 8700.

4066, 16133.333, 200., 8700.

4067, 16600., 200., 8700.

4068, 17066.666, 200., 8700.

4069, 17533.334, 200., 8700.

4070, 17533.334, 0., 8700.

4071, 17066.666, 0., 8700.

4072, 16600., 0., 8700.

4073, 16133.333, 0., 8700.

4074, 15666.667, 0., 8700.

4075, 15666.667, 200., 8920.

4076, 16133.333, 200., 8920.

4077, 16600., 200., 8920.

4078, 17066.666, 200., 8920.

4079, 17533.334, 200., 8920.

4080, 17533.334, 0., 8920.

4081, 17066.666, 0., 8920.

4082, 16600., 0., 8920.

4083, 16133.333, 0., 8920.

4084, 15666.667, 0., 8920.

4085, 17533.334, 3000., 8920.

4086, 17066.666, 3000., 8920.

4087, 16600., 3000., 8920.

4088, 16133.333, 3000., 8920.

4089, 15666.667, 3000., 8920.

4090, 15666.667, 3000., 8700.

4091, 16133.333, 3000., 8700.

4092, 16600., 3000., 8700.

4093, 17066.666, 3000., 8700.

4094, 17533.334, 3000., 8700.

4095, 15666.667, 3200., 8920.

4096, 16133.333, 3200., 8920.

4097, 16600., 3200., 8920.

4098, 17066.666, 3200., 8920.

4099, 17533.334, 3200., 8920.

4100, 17533.334, 3200., 8700.

4101, 17066.666, 3200., 8700.

4102, 16600., 3200., 8700.

4103, 16133.333, 3200., 8700.

4104, 15666.667, 3200., 8700.

4105, 17533.334, 6000., 8920.

4106, 17066.666, 6000., 8920.

4107, 16600., 6000., 8920.

4108, 16133.333, 6000., 8920.

4109, 15666.667, 6000., 8920.

4110, 15666.667, 6000., 8700.

4111, 16133.333, 6000., 8700.

4112, 16600., 6000., 8700.

4113, 17066.666, 6000., 8700.

4114, 17533.334, 6000., 8700.

4115, 15666.667, 6200., 8920.

4116, 16133.333, 6200., 8920.

4117, 16600., 6200., 8920.

4118, 17066.666, 6200., 8920.

4119, 17533.334, 6200., 8920.

4120, 17533.334, 6200., 8700.

4121, 17066.666, 6200., 8700.

4122, 16600., 6200., 8700.

4123, 16133.333, 6200., 8700.

4124, 15666.667, 6200., 8700.

4125, 17533.334, 9000., 8920.

4126, 17066.666, 9000., 8920.

4127, 16600., 9000., 8920.

4128, 16133.333, 9000., 8920.

4129, 15666.667, 9000., 8920.

4130, 15666.667, 9000., 8700.

4131, 16133.333, 9000., 8700.

4132, 16600., 9000., 8700.

4133, 17066.666, 9000., 8700.

4134, 17533.334, 9000., 8700.

4135, 15666.667, 9200., 8920.

4136, 16133.333, 9200., 8920.

4137, 16600., 9200., 8920.

4138, 17066.666, 9200., 8920.

4139, 17533.334, 9200., 8920.

4140, 17533.334, 9200., 8700.

4141, 17066.666, 9200., 8700.

4142, 16600., 9200., 8700.

4143, 16133.333, 9200., 8700.

4144, 15666.667, 9200., 8700.

4145, 15666.667, 15200., 5920.

4146, 16133.333, 15200., 5920.

4147, 16600., 15200., 5920.

4148, 17066.666, 15200., 5920.

4149, 17533.334, 15200., 5920.

4150, 17533.334, 15200., 5700.

4151, 17066.666, 15200., 5700.

4152, 16600., 15200., 5700.

4153, 16133.333, 15200., 5700.

4154, 15666.667, 15200., 5700.

4155, 17533.334, 15000., 5920.

4156, 17066.666, 15000., 5920.

4157, 16600., 15000., 5920.

4158, 16133.333, 15000., 5920.

4159, 15666.667, 15000., 5920.

4160, 15666.667, 15000., 5700.

4161, 16133.333, 15000., 5700.

4162, 16600., 15000., 5700.

4163, 17066.666, 15000., 5700.

4164, 17533.334, 15000., 5700.

4165, 17533.334, 12000., 5920.

4166, 17066.666, 12000., 5920.

4167, 16600., 12000., 5920.

4168, 16133.333, 12000., 5920.

4169, 15666.667, 12000., 5920.

4170, 15666.667, 12000., 5700.

4171, 16133.333, 12000., 5700.

4172, 16600., 12000., 5700.

4173, 17066.666, 12000., 5700.

4174, 17533.334, 12000., 5700.

4175, 15666.667, 12200., 5920.

4176, 16133.333, 12200., 5920.

4177, 16600., 12200., 5920.

4178, 17066.666, 12200., 5920.

4179, 17533.334, 12200., 5920.

4180, 17533.334, 12200., 5700.

4181, 17066.666, 12200., 5700.

4182, 16600., 12200., 5700.

4183, 16133.333, 12200., 5700.

4184, 15666.667, 12200., 5700.

4185, 17533.334, 6000., 5920.

4186, 17066.666, 6000., 5920.

4187, 16600., 6000., 5920.

4188, 16133.333, 6000., 5920.

4189, 15666.667, 6000., 5920.

4190, 15666.667, 6000., 5700.

4191, 16133.333, 6000., 5700.

4192, 16600., 6000., 5700.

4193, 17066.666, 6000., 5700.

4194, 17533.334, 6000., 5700.

4195, 15666.667, 6200., 5920.

4196, 16133.333, 6200., 5920.

4197, 16600., 6200., 5920.

4198, 17066.666, 6200., 5920.

4199, 17533.334, 6200., 5920.

4200, 17533.334, 6200., 5700.

4201, 17066.666, 6200., 5700.

4202, 16600., 6200., 5700.

4203, 16133.333, 6200., 5700.

4204, 15666.667, 6200., 5700.

4205, 17533.334, 9000., 5920.

4206, 17066.666, 9000., 5920.

4207, 16600., 9000., 5920.

4208, 16133.333, 9000., 5920.

4209, 15666.667, 9000., 5920.

4210, 15666.667, 9000., 5700.

4211, 16133.333, 9000., 5700.

4212, 16600., 9000., 5700.

4213, 17066.666, 9000., 5700.

4214, 17533.334, 9000., 5700.

4215, 15666.667, 9200., 5920.

4216, 16133.333, 9200., 5920.

4217, 16600., 9200., 5920.

4218, 17066.666, 9200., 5920.

4219, 17533.334, 9200., 5920.

4220, 17533.334, 9200., 5700.

4221, 17066.666, 9200., 5700.

4222, 16600., 9200., 5700.

4223, 16133.333, 9200., 5700.

4224, 15666.667, 9200., 5700.

4225, 15666.667, 3200., 5920.

4226, 16133.333, 3200., 5920.

4227, 16600., 3200., 5920.

4228, 17066.666, 3200., 5920.

4229, 17533.334, 3200., 5920.

4230, 17533.334, 3200., 5700.

4231, 17066.666, 3200., 5700.

4232, 16600., 3200., 5700.

4233, 16133.333, 3200., 5700.

4234, 15666.667, 3200., 5700.

4235, 17533.334, 3000., 5920.

4236, 17066.666, 3000., 5920.

4237, 16600., 3000., 5920.

4238, 16133.333, 3000., 5920.

4239, 15666.667, 3000., 5920.

4240, 15666.667, 3000., 5700.

4241, 16133.333, 3000., 5700.

4242, 16600., 3000., 5700.

4243, 17066.666, 3000., 5700.

4244, 17533.334, 3000., 5700.

4245, 17533.334, 0., 5920.

4246, 17066.666, 0., 5920.

4247, 16600., 0., 5920.

4248, 16133.333, 0., 5920.

4249, 15666.667, 0., 5920.

4250, 15666.667, 200., 5920.

4251, 16133.333, 200., 5920.

4252, 16600., 200., 5920.

4253, 17066.666, 200., 5920.

4254, 17533.334, 200., 5920.

4255, 18000., 2533.33325, 5920.

4256, 18000., 2066.66675, 5920.

4257, 18000., 1600., 5920.

4258, 18000., 1133.33337, 5920.

4259, 18000., 666.666687, 5920.

4260, 15200., 666.666687, 5920.

4261, 15200., 1133.33337, 5920.

4262, 15200., 1600., 5920.

4263, 15200., 2066.66675, 5920.

4264, 15200., 2533.33325, 5920.

4265, 18000., 5533.3335, 5920.

4266, 18000., 5066.6665, 5920.

4267, 18000., 4600., 5920.

4268, 18000., 4133.3335, 5920.

4269, 18000., 3666.66675, 5920.

4270, 15200., 3666.66675, 5920.

4271, 15200., 4133.3335, 5920.

4272, 15200., 4600., 5920.

4273, 15200., 5066.6665, 5920.

4274, 15200., 5533.3335, 5920.

4275, 18000., 8533.33301, 5920.

4276, 18000., 8066.6665, 5920.

4277, 18000., 7600., 5920.

4278, 18000., 7133.3335, 5920.

4279, 18000., 6666.6665, 5920.

4280, 15200., 6666.6665, 5920.

4281, 15200., 7133.3335, 5920.

4282, 15200., 7600., 5920.

4283, 15200., 8066.6665, 5920.

4284, 15200., 8533.33301, 5920.

4285, 18000., 11533.333, 5920.

4286, 18000., 11066.667, 5920.

4287, 18000., 10600., 5920.

4288, 18000., 10133.333, 5920.

4289, 18000., 9666.66699, 5920.

4290, 15200., 9666.66699, 5920.

4291, 15200., 10133.333, 5920.

4292, 15200., 10600., 5920.

4293, 15200., 11066.667, 5920.

4294, 15200., 11533.333, 5920.

4295, 18000., 14533.333, 5920.

4296, 18000., 14066.667, 5920.

4297, 18000., 13600., 5920.

4298, 18000., 13133.333, 5920.

4299, 18000., 12666.667, 5920.

4300, 15200., 12666.667, 5920.

4301, 15200., 13133.333, 5920.

4302, 15200., 13600., 5920.

4303, 15200., 14066.667, 5920.

4304, 15200., 14533.333, 5920.

4305, 15666.667, 0., 6000.

4306, 16133.333, 0., 6000.

4307, 16600., 0., 6000.

4308, 17066.666, 0., 6000.

4309, 17533.334, 0., 6000.

4310, 15200., 2533.33325, 6000.

4311, 15200., 2066.66675, 6000.

4312, 15200., 1600., 6000.

4313, 15200., 1133.33337, 6000.

4314, 15200., 666.666687, 6000.

4315, 15200., 5533.3335, 6000.

4316, 15200., 5066.6665, 6000.

4317, 15200., 4600., 6000.

4318, 15200., 4133.3335, 6000.

4319, 15200., 3666.66675, 6000.

4320, 15200., 8533.33301, 6000.

4321, 15200., 8066.6665, 6000.

4322, 15200., 7600., 6000.

4323, 15200., 7133.3335, 6000.

4324, 15200., 6666.6665, 6000.

4325, 15200., 11533.333, 6000.

4326, 15200., 11066.667, 6000.

4327, 15200., 10600., 6000.

4328, 15200., 10133.333, 6000.

4329, 15200., 9666.66699, 6000.

4330, 15200., 14533.333, 6000.

4331, 15200., 14066.667, 6000.

4332, 15200., 13600., 6000.

4333, 15200., 13133.333, 6000.

4334, 15200., 12666.667, 6000.

4335, 17533.334, 15200., 6000.

4336, 17066.666, 15200., 6000.

4337, 16600., 15200., 6000.

4338, 16133.333, 15200., 6000.

4339, 15666.667, 15200., 6000.

4340, 18000., 666.666687, 6000.

4341, 18000., 1133.33337, 6000.

4342, 18000., 1600., 6000.

4343, 18000., 2066.66675, 6000.

4344, 18000., 2533.33325, 6000.

4345, 18000., 3666.66675, 6000.

4346, 18000., 4133.3335, 6000.

4347, 18000., 4600., 6000.

4348, 18000., 5066.6665, 6000.

4349, 18000., 5533.3335, 6000.

4350, 18000., 6666.6665, 6000.

4351, 18000., 7133.3335, 6000.

4352, 18000., 7600., 6000.

4353, 18000., 8066.6665, 6000.

4354, 18000., 8533.33301, 6000.

4355, 18000., 9666.66699, 6000.

4356, 18000., 10133.333, 6000.

4357, 18000., 10600., 6000.

4358, 18000., 11066.667, 6000.

4359, 18000., 11533.333, 6000.

4360, 18000., 12666.667, 6000.

4361, 18000., 13133.333, 6000.

4362, 18000., 13600., 6000.

4363, 18000., 14066.667, 6000.

4364, 18000., 14533.333, 6000.

4365, 17533.334, 0., 2920.

4366, 17066.666, 0., 2920.

4367, 16600., 0., 2920.

4368, 16133.333, 0., 2920.

4369, 15666.667, 0., 2920.

4370, 15666.667, 0., 2700.

4371, 16133.333, 0., 2700.

4372, 16600., 0., 2700.

4373, 17066.666, 0., 2700.

4374, 17533.334, 0., 2700.

4375, 15666.667, 200., 2920.

4376, 16133.333, 200., 2920.

4377, 16600., 200., 2920.

4378, 17066.666, 200., 2920.

4379, 17533.334, 200., 2920.

4380, 17533.334, 200., 2700.

4381, 17066.666, 200., 2700.

4382, 16600., 200., 2700.

4383, 16133.333, 200., 2700.

4384, 15666.667, 200., 2700.

4385, 15666.667, 3200., 2920.

4386, 16133.333, 3200., 2920.

4387, 16600., 3200., 2920.

4388, 17066.666, 3200., 2920.

4389, 17533.334, 3200., 2920.

4390, 17533.334, 3200., 2700.

4391, 17066.666, 3200., 2700.

4392, 16600., 3200., 2700.

4393, 16133.333, 3200., 2700.

4394, 15666.667, 3200., 2700.

4395, 17533.334, 3000., 2920.

4396, 17066.666, 3000., 2920.

4397, 16600., 3000., 2920.

4398, 16133.333, 3000., 2920.

4399, 15666.667, 3000., 2920.

4400, 15666.667, 3000., 2700.

4401, 16133.333, 3000., 2700.

4402, 16600., 3000., 2700.

4403, 17066.666, 3000., 2700.

4404, 17533.334, 3000., 2700.

4405, 17533.334, 6000., 2920.

4406, 17066.666, 6000., 2920.

4407, 16600., 6000., 2920.

4408, 16133.333, 6000., 2920.

4409, 15666.667, 6000., 2920.

4410, 15666.667, 6000., 2700.

4411, 16133.333, 6000., 2700.

4412, 16600., 6000., 2700.

4413, 17066.666, 6000., 2700.

4414, 17533.334, 6000., 2700.

4415, 15666.667, 6200., 2920.

4416, 16133.333, 6200., 2920.

4417, 16600., 6200., 2920.

4418, 17066.666, 6200., 2920.

4419, 17533.334, 6200., 2920.

4420, 17533.334, 6200., 2700.

4421, 17066.666, 6200., 2700.

4422, 16600., 6200., 2700.

4423, 16133.333, 6200., 2700.

4424, 15666.667, 6200., 2700.

4425, 17533.334, 9000., 2920.

4426, 17066.666, 9000., 2920.

4427, 16600., 9000., 2920.

4428, 16133.333, 9000., 2920.

4429, 15666.667, 9000., 2920.

4430, 15666.667, 9000., 2700.

4431, 16133.333, 9000., 2700.

4432, 16600., 9000., 2700.

4433, 17066.666, 9000., 2700.

4434, 17533.334, 9000., 2700.

4435, 15666.667, 9200., 2920.

4436, 16133.333, 9200., 2920.

4437, 16600., 9200., 2920.

4438, 17066.666, 9200., 2920.

4439, 17533.334, 9200., 2920.

4440, 17533.334, 9200., 2700.

4441, 17066.666, 9200., 2700.

4442, 16600., 9200., 2700.

4443, 16133.333, 9200., 2700.

4444, 15666.667, 9200., 2700.

4445, 17533.334, 12000., 2920.

4446, 17066.666, 12000., 2920.

4447, 16600., 12000., 2920.

4448, 16133.333, 12000., 2920.

4449, 15666.667, 12000., 2920.

4450, 15666.667, 12000., 2700.

4451, 16133.333, 12000., 2700.

4452, 16600., 12000., 2700.

4453, 17066.666, 12000., 2700.

4454, 17533.334, 12000., 2700.

4455, 15666.667, 12200., 2920.

4456, 16133.333, 12200., 2920.

4457, 16600., 12200., 2920.

4458, 17066.666, 12200., 2920.

4459, 17533.334, 12200., 2920.

4460, 17533.334, 12200., 2700.

4461, 17066.666, 12200., 2700.

4462, 16600., 12200., 2700.

4463, 16133.333, 12200., 2700.

4464, 15666.667, 12200., 2700.

4465, 15666.667, 15200., 2920.

4466, 16133.333, 15200., 2920.

4467, 16600., 15200., 2920.

4468, 17066.666, 15200., 2920.

4469, 17533.334, 15200., 2920.

4470, 17533.334, 15000., 2920.

4471, 17066.666, 15000., 2920.

4472, 16600., 15000., 2920.

4473, 16133.333, 15000., 2920.

4474, 15666.667, 15000., 2920.

4475, 18000., 14533.333, 2920.

4476, 18000., 14066.667, 2920.

4477, 18000., 13600., 2920.

4478, 18000., 13133.333, 2920.

4479, 18000., 12666.667, 2920.

4480, 15200., 12666.667, 2920.

4481, 15200., 13133.333, 2920.

4482, 15200., 13600., 2920.

4483, 15200., 14066.667, 2920.

4484, 15200., 14533.333, 2920.

4485, 18000., 11533.333, 2920.

4486, 18000., 11066.667, 2920.

4487, 18000., 10600., 2920.

4488, 18000., 10133.333, 2920.

4489, 18000., 9666.66699, 2920.

4490, 15200., 9666.66699, 2920.

4491, 15200., 10133.333, 2920.

4492, 15200., 10600., 2920.

4493, 15200., 11066.667, 2920.

4494, 15200., 11533.333, 2920.

4495, 18000., 8533.33301, 2920.

4496, 18000., 8066.6665, 2920.

4497, 18000., 7600., 2920.

4498, 18000., 7133.3335, 2920.

4499, 18000., 6666.6665, 2920.

4500, 15200., 6666.6665, 2920.

4501, 15200., 7133.3335, 2920.

4502, 15200., 7600., 2920.

4503, 15200., 8066.6665, 2920.

4504, 15200., 8533.33301, 2920.

4505, 18000., 5533.3335, 2920.

4506, 18000., 5066.6665, 2920.

4507, 18000., 4600., 2920.

4508, 18000., 4133.3335, 2920.

4509, 18000., 3666.66675, 2920.

4510, 15200., 3666.66675, 2920.

4511, 15200., 4133.3335, 2920.

4512, 15200., 4600., 2920.

4513, 15200., 5066.6665, 2920.

4514, 15200., 5533.3335, 2920.

4515, 15200., 666.666687, 2920.

4516, 15200., 1133.33337, 2920.

4517, 15200., 1600., 2920.

4518, 15200., 2066.66675, 2920.

4519, 15200., 2533.33325, 2920.

4520, 18000., 2533.33325, 2920.

4521, 18000., 2066.66675, 2920.

4522, 18000., 1600., 2920.

4523, 18000., 1133.33337, 2920.

4524, 18000., 666.666687, 2920.

4525, 17533.334, 15200., 3000.

4526, 17066.666, 15200., 3000.

4527, 16600., 15200., 3000.

4528, 16133.333, 15200., 3000.

4529, 15666.667, 15200., 3000.

4530, 18000., 12666.667, 3000.

4531, 18000., 13133.333, 3000.

4532, 18000., 13600., 3000.

4533, 18000., 14066.667, 3000.

4534, 18000., 14533.333, 3000.

4535, 18000., 9666.66699, 3000.

4536, 18000., 10133.333, 3000.

4537, 18000., 10600., 3000.

4538, 18000., 11066.667, 3000.

4539, 18000., 11533.333, 3000.

4540, 18000., 6666.6665, 3000.

4541, 18000., 7133.3335, 3000.

4542, 18000., 7600., 3000.

4543, 18000., 8066.6665, 3000.

4544, 18000., 8533.33301, 3000.

4545, 18000., 3666.66675, 3000.

4546, 18000., 4133.3335, 3000.

4547, 18000., 4600., 3000.

4548, 18000., 5066.6665, 3000.

4549, 18000., 5533.3335, 3000.

4550, 18000., 666.666687, 3000.

4551, 18000., 1133.33337, 3000.

4552, 18000., 1600., 3000.

4553, 18000., 2066.66675, 3000.

4554, 18000., 2533.33325, 3000.

4555, 15666.667, 0., 3000.

4556, 16133.333, 0., 3000.

4557, 16600., 0., 3000.

4558, 17066.666, 0., 3000.

4559, 17533.334, 0., 3000.

4560, 15200., 14533.333, 3000.

4561, 15200., 14066.667, 3000.

4562, 15200., 13600., 3000.

4563, 15200., 13133.333, 3000.

4564, 15200., 12666.667, 3000.

4565, 15200., 11533.333, 3000.

4566, 15200., 11066.667, 3000.

4567, 15200., 10600., 3000.

4568, 15200., 10133.333, 3000.

4569, 15200., 9666.66699, 3000.

4570, 15200., 8533.33301, 3000.

4571, 15200., 8066.6665, 3000.

4572, 15200., 7600., 3000.

4573, 15200., 7133.3335, 3000.

4574, 15200., 6666.6665, 3000.

4575, 15200., 5533.3335, 3000.

4576, 15200., 5066.6665, 3000.

4577, 15200., 4600., 3000.

4578, 15200., 4133.3335, 3000.

4579, 15200., 3666.66675, 3000.

4580, 15200., 2533.33325, 3000.

4581, 15200., 2066.66675, 3000.

4582, 15200., 1600., 3000.

4583, 15200., 1133.33337, 3000.

4584, 15200., 666.666687, 3000.

4585, 18666.666, 6200., 2920.

4586, 19133.334, 6200., 2920.

4587, 19600., 6200., 2920.

4588, 20066.666, 6200., 2920.

4589, 20533.334, 6200., 2920.

4590, 20533.334, 6200., 2700.

4591, 20066.666, 6200., 2700.

4592, 19600., 6200., 2700.

4593, 19133.334, 6200., 2700.

4594, 18666.666, 6200., 2700.

4595, 20533.334, 6000., 2920.

4596, 20066.666, 6000., 2920.

4597, 19600., 6000., 2920.

4598, 19133.334, 6000., 2920.

4599, 18666.666, 6000., 2920.

4600, 18666.666, 6000., 2700.

4601, 19133.334, 6000., 2700.

4602, 19600., 6000., 2700.

4603, 20066.666, 6000., 2700.

4604, 20533.334, 6000., 2700.

4605, 20533.334, 3000., 2920.

4606, 20066.666, 3000., 2920.

4607, 19600., 3000., 2920.

4608, 19133.334, 3000., 2920.

4609, 18666.666, 3000., 2920.

4610, 18666.666, 3000., 2700.

4611, 19133.334, 3000., 2700.

4612, 19600., 3000., 2700.

4613, 20066.666, 3000., 2700.

4614, 20533.334, 3000., 2700.

4615, 18666.666, 3200., 2920.

4616, 19133.334, 3200., 2920.

4617, 19600., 3200., 2920.

4618, 20066.666, 3200., 2920.

4619, 20533.334, 3200., 2920.

4620, 20533.334, 3200., 2700.

4621, 20066.666, 3200., 2700.

4622, 19600., 3200., 2700.

4623, 19133.334, 3200., 2700.

4624, 18666.666, 3200., 2700.

4625, 20533.334, 15000., 2920.

4626, 20066.666, 15000., 2920.

4627, 19600., 15000., 2920.

4628, 19133.334, 15000., 2920.

4629, 18666.666, 15000., 2920.

4630, 18666.666, 15000., 2700.

4631, 19133.334, 15000., 2700.

4632, 19600., 15000., 2700.

4633, 20066.666, 15000., 2700.

4634, 20533.334, 15000., 2700.

4635, 18666.666, 15200., 2920.

4636, 19133.334, 15200., 2920.

4637, 19600., 15200., 2920.

4638, 20066.666, 15200., 2920.

4639, 20533.334, 15200., 2920.

4640, 20533.334, 15200., 2700.

4641, 20066.666, 15200., 2700.

4642, 19600., 15200., 2700.

4643, 19133.334, 15200., 2700.

4644, 18666.666, 15200., 2700.

4645, 18666.666, 12200., 2920.

4646, 19133.334, 12200., 2920.

4647, 19600., 12200., 2920.

4648, 20066.666, 12200., 2920.

4649, 20533.334, 12200., 2920.

4650, 20533.334, 12200., 2700.

4651, 20066.666, 12200., 2700.

4652, 19600., 12200., 2700.

4653, 19133.334, 12200., 2700.

4654, 18666.666, 12200., 2700.

4655, 20533.334, 12000., 2920.

4656, 20066.666, 12000., 2920.

4657, 19600., 12000., 2920.

4658, 19133.334, 12000., 2920.

4659, 18666.666, 12000., 2920.

4660, 18666.666, 12000., 2700.

4661, 19133.334, 12000., 2700.

4662, 19600., 12000., 2700.

4663, 20066.666, 12000., 2700.

4664, 20533.334, 12000., 2700.

4665, 20533.334, 0., 2920.

4666, 20066.666, 0., 2920.

4667, 19600., 0., 2920.

4668, 19133.334, 0., 2920.

4669, 18666.666, 0., 2920.

4670, 18666.666, 200., 2920.

4671, 19133.334, 200., 2920.

4672, 19600., 200., 2920.

4673, 20066.666, 200., 2920.

4674, 20533.334, 200., 2920.

4675, 21000., 8533.33301, 2920.

4676, 21000., 8066.6665, 2920.

4677, 21000., 7600., 2920.

4678, 21000., 7133.3335, 2920.

4679, 21000., 6666.6665, 2920.

4680, 18200., 6666.6665, 2920.

4681, 18200., 7133.3335, 2920.

4682, 18200., 7600., 2920.

4683, 18200., 8066.6665, 2920.

4684, 18200., 8533.33301, 2920.

4685, 18666.666, 9000., 2920.

4686, 19133.334, 9000., 2920.

4687, 19600., 9000., 2920.

4688, 20066.666, 9000., 2920.

4689, 20533.334, 9000., 2920.

4690, 18666.666, 9200., 2920.

4691, 19133.334, 9200., 2920.

4692, 19600., 9200., 2920.

4693, 20066.666, 9200., 2920.

4694, 20533.334, 9200., 2920.

4695, 21000., 11533.333, 2920.

4696, 21000., 11066.667, 2920.

4697, 21000., 10600., 2920.

4698, 21000., 10133.333, 2920.

4699, 21000., 9666.66699, 2920.

4700, 18200., 9666.66699, 2920.

4701, 18200., 10133.333, 2920.

4702, 18200., 10600., 2920.

4703, 18200., 11066.667, 2920.

4704, 18200., 11533.333, 2920.

4705, 21000., 14533.333, 2920.

4706, 21000., 14066.667, 2920.

4707, 21000., 13600., 2920.

4708, 21000., 13133.333, 2920.

4709, 21000., 12666.667, 2920.

4710, 18200., 12666.667, 2920.

4711, 18200., 13133.333, 2920.

4712, 18200., 13600., 2920.

4713, 18200., 14066.667, 2920.

4714, 18200., 14533.333, 2920.

4715, 21000., 5533.3335, 2920.

4716, 21000., 5066.6665, 2920.

4717, 21000., 4600., 2920.

4718, 21000., 4133.3335, 2920.

4719, 21000., 3666.66675, 2920.

4720, 18200., 3666.66675, 2920.

4721, 18200., 4133.3335, 2920.

4722, 18200., 4600., 2920.

4723, 18200., 5066.6665, 2920.

4724, 18200., 5533.3335, 2920.

4725, 18200., 666.666687, 2920.

4726, 18200., 1133.33337, 2920.

4727, 18200., 1600., 2920.

4728, 18200., 2066.66675, 2920.

4729, 18200., 2533.33325, 2920.

4730, 21000., 2533.33325, 2920.

4731, 21000., 2066.66675, 2920.

4732, 21000., 1600., 2920.

4733, 21000., 1133.33337, 2920.

4734, 21000., 666.666687, 2920.

4735, 18666.666, 0., 3000.

4736, 19133.334, 0., 3000.

4737, 19600., 0., 3000.

4738, 20066.666, 0., 3000.

4739, 20533.334, 0., 3000.

4740, 18200., 2533.33325, 3000.

4741, 18200., 2066.66675, 3000.

4742, 18200., 1600., 3000.

4743, 18200., 1133.33337, 3000.

4744, 18200., 666.666687, 3000.

4745, 18200., 5533.3335, 3000.

4746, 18200., 5066.6665, 3000.

4747, 18200., 4600., 3000.

4748, 18200., 4133.3335, 3000.

4749, 18200., 3666.66675, 3000.

4750, 18200., 8533.33301, 3000.

4751, 18200., 8066.6665, 3000.

4752, 18200., 7600., 3000.

4753, 18200., 7133.3335, 3000.

4754, 18200., 6666.6665, 3000.

4755, 18200., 11533.333, 3000.

4756, 18200., 11066.667, 3000.

4757, 18200., 10600., 3000.

4758, 18200., 10133.333, 3000.

4759, 18200., 9666.66699, 3000.

4760, 18200., 14533.333, 3000.

4761, 18200., 14066.667, 3000.

4762, 18200., 13600., 3000.

4763, 18200., 13133.333, 3000.

4764, 18200., 12666.667, 3000.

4765, 20533.334, 15200., 3000.

4766, 20066.666, 15200., 3000.

4767, 19600., 15200., 3000.

4768, 19133.334, 15200., 3000.

4769, 18666.666, 15200., 3000.

4770, 21000., 666.666687, 3000.

4771, 21000., 1133.33337, 3000.

4772, 21000., 1600., 3000.

4773, 21000., 2066.66675, 3000.

4774, 21000., 2533.33325, 3000.

4775, 21000., 3666.66675, 3000.

4776, 21000., 4133.3335, 3000.

4777, 21000., 4600., 3000.

4778, 21000., 5066.6665, 3000.

4779, 21000., 5533.3335, 3000.

4780, 21000., 6666.6665, 3000.

4781, 21000., 7133.3335, 3000.

4782, 21000., 7600., 3000.

4783, 21000., 8066.6665, 3000.

4784, 21000., 8533.33301, 3000.

4785, 21000., 9666.66699, 3000.

4786, 21000., 10133.333, 3000.

4787, 21000., 10600., 3000.

4788, 21000., 11066.667, 3000.

4789, 21000., 11533.333, 3000.

4790, 21000., 12666.667, 3000.

4791, 21000., 13133.333, 3000.

4792, 21000., 13600., 3000.

4793, 21000., 14066.667, 3000.

4794, 21000., 14533.333, 3000.

4795, 20533.334, 9200., 2700.

4796, 20066.666, 9200., 2700.

4797, 19600., 9200., 2700.

4798, 19133.334, 9200., 2700.

4799, 18666.666, 9200., 2700.

4800, 18666.666, 9000., 2700.

4801, 19133.334, 9000., 2700.

4802, 19600., 9000., 2700.

4803, 20066.666, 9000., 2700.

4804, 20533.334, 9000., 2700.

4805, 20533.334, 0., 5920.

4806, 20066.666, 0., 5920.

4807, 19600., 0., 5920.

4808, 19133.334, 0., 5920.

4809, 18666.666, 0., 5920.

4810, 18666.666, 0., 5700.

4811, 19133.334, 0., 5700.

4812, 19600., 0., 5700.

4813, 20066.666, 0., 5700.

4814, 20533.334, 0., 5700.

4815, 18666.666, 200., 5920.

4816, 19133.334, 200., 5920.

4817, 19600., 200., 5920.

4818, 20066.666, 200., 5920.

4819, 20533.334, 200., 5920.

4820, 20533.334, 200., 5700.

4821, 20066.666, 200., 5700.

4822, 19600., 200., 5700.

4823, 19133.334, 200., 5700.

4824, 18666.666, 200., 5700.

4825, 18666.666, 3200., 5920.

4826, 19133.334, 3200., 5920.

4827, 19600., 3200., 5920.

4828, 20066.666, 3200., 5920.

4829, 20533.334, 3200., 5920.

4830, 20533.334, 3200., 5700.

4831, 20066.666, 3200., 5700.

4832, 19600., 3200., 5700.

4833, 19133.334, 3200., 5700.

4834, 18666.666, 3200., 5700.

4835, 20533.334, 3000., 5920.

4836, 20066.666, 3000., 5920.

4837, 19600., 3000., 5920.

4838, 19133.334, 3000., 5920.

4839, 18666.666, 3000., 5920.

4840, 18666.666, 3000., 5700.

4841, 19133.334, 3000., 5700.

4842, 19600., 3000., 5700.

4843, 20066.666, 3000., 5700.

4844, 20533.334, 3000., 5700.

4845, 20533.334, 12000., 5920.

4846, 20066.666, 12000., 5920.

4847, 19600., 12000., 5920.

4848, 19133.334, 12000., 5920.

4849, 18666.666, 12000., 5920.

4850, 18666.666, 12000., 5700.

4851, 19133.334, 12000., 5700.

4852, 19600., 12000., 5700.

4853, 20066.666, 12000., 5700.

4854, 20533.334, 12000., 5700.

4855, 18666.666, 12200., 5920.

4856, 19133.334, 12200., 5920.

4857, 19600., 12200., 5920.

4858, 20066.666, 12200., 5920.

4859, 20533.334, 12200., 5920.

4860, 20533.334, 12200., 5700.

4861, 20066.666, 12200., 5700.

4862, 19600., 12200., 5700.

4863, 19133.334, 12200., 5700.

4864, 18666.666, 12200., 5700.

4865, 20533.334, 15000., 5920.

4866, 20066.666, 15000., 5920.

4867, 19600., 15000., 5920.

4868, 19133.334, 15000., 5920.

4869, 18666.666, 15000., 5920.

4870, 18666.666, 15000., 5700.

4871, 19133.334, 15000., 5700.

4872, 19600., 15000., 5700.

4873, 20066.666, 15000., 5700.

4874, 20533.334, 15000., 5700.

4875, 18666.666, 15200., 5920.

4876, 19133.334, 15200., 5920.

4877, 19600., 15200., 5920.

4878, 20066.666, 15200., 5920.

4879, 20533.334, 15200., 5920.

4880, 20533.334, 15200., 5700.

4881, 20066.666, 15200., 5700.

4882, 19600., 15200., 5700.

4883, 19133.334, 15200., 5700.

4884, 18666.666, 15200., 5700.

4885, 18666.666, 9200., 5920.

4886, 19133.334, 9200., 5920.

4887, 19600., 9200., 5920.

4888, 20066.666, 9200., 5920.

4889, 20533.334, 9200., 5920.

4890, 20533.334, 9200., 5700.

4891, 20066.666, 9200., 5700.

4892, 19600., 9200., 5700.

4893, 19133.334, 9200., 5700.

4894, 18666.666, 9200., 5700.

4895, 20533.334, 9000., 5920.

4896, 20066.666, 9000., 5920.

4897, 19600., 9000., 5920.

4898, 19133.334, 9000., 5920.

4899, 18666.666, 9000., 5920.

4900, 18666.666, 9000., 5700.

4901, 19133.334, 9000., 5700.

4902, 19600., 9000., 5700.

4903, 20066.666, 9000., 5700.

4904, 20533.334, 9000., 5700.

4905, 18666.666, 6200., 5920.

4906, 19133.334, 6200., 5920.

4907, 19600., 6200., 5920.

4908, 20066.666, 6200., 5920.

4909, 20533.334, 6200., 5920.

4910, 20533.334, 6200., 5700.

4911, 20066.666, 6200., 5700.

4912, 19600., 6200., 5700.

4913, 19133.334, 6200., 5700.

4914, 18666.666, 6200., 5700.

4915, 20533.334, 6000., 5920.

4916, 20066.666, 6000., 5920.

4917, 19600., 6000., 5920.

4918, 19133.334, 6000., 5920.

4919, 18666.666, 6000., 5920.

4920, 18666.666, 6000., 5700.

4921, 19133.334, 6000., 5700.

4922, 19600., 6000., 5700.

4923, 20066.666, 6000., 5700.

4924, 20533.334, 6000., 5700.

4925, 18666.666, 15200., 8920.

4926, 19133.334, 15200., 8920.

4927, 19600., 15200., 8920.

4928, 20066.666, 15200., 8920.

4929, 20533.334, 15200., 8920.

4930, 20533.334, 15200., 8700.

4931, 20066.666, 15200., 8700.

4932, 19600., 15200., 8700.

4933, 19133.334, 15200., 8700.

4934, 18666.666, 15200., 8700.

4935, 20533.334, 15000., 8920.

4936, 20066.666, 15000., 8920.

4937, 19600., 15000., 8920.

4938, 19133.334, 15000., 8920.

4939, 18666.666, 15000., 8920.

4940, 18666.666, 15000., 8700.

4941, 19133.334, 15000., 8700.

4942, 19600., 15000., 8700.

4943, 20066.666, 15000., 8700.

4944, 20533.334, 15000., 8700.

4945, 20533.334, 12000., 8920.

4946, 20066.666, 12000., 8920.

4947, 19600., 12000., 8920.

4948, 19133.334, 12000., 8920.

4949, 18666.666, 12000., 8920.

4950, 18666.666, 12000., 8700.

4951, 19133.334, 12000., 8700.

4952, 19600., 12000., 8700.

4953, 20066.666, 12000., 8700.

4954, 20533.334, 12000., 8700.

4955, 18666.666, 12200., 8920.

4956, 19133.334, 12200., 8920.

4957, 19600., 12200., 8920.

4958, 20066.666, 12200., 8920.

4959, 20533.334, 12200., 8920.

4960, 20533.334, 12200., 8700.

4961, 20066.666, 12200., 8700.

4962, 19600., 12200., 8700.

4963, 19133.334, 12200., 8700.

4964, 18666.666, 12200., 8700.

4965, 20533.334, 0., 8700.

4966, 20066.666, 0., 8700.

4967, 19600., 0., 8700.

4968, 19133.334, 0., 8700.

4969, 18666.666, 0., 8700.

4970, 18666.666, 200., 8700.

4971, 19133.334, 200., 8700.

4972, 19600., 200., 8700.

4973, 20066.666, 200., 8700.

4974, 20533.334, 200., 8700.

4975, 20533.334, 0., 8920.

4976, 20066.666, 0., 8920.

4977, 19600., 0., 8920.

4978, 19133.334, 0., 8920.

4979, 18666.666, 0., 8920.

4980, 18666.666, 200., 8920.

4981, 19133.334, 200., 8920.

4982, 19600., 200., 8920.

4983, 20066.666, 200., 8920.

4984, 20533.334, 200., 8920.

4985, 20533.334, 3000., 8920.

4986, 20066.666, 3000., 8920.

4987, 19600., 3000., 8920.

4988, 19133.334, 3000., 8920.

4989, 18666.666, 3000., 8920.

4990, 18666.666, 3000., 8700.

4991, 19133.334, 3000., 8700.

4992, 19600., 3000., 8700.

4993, 20066.666, 3000., 8700.

4994, 20533.334, 3000., 8700.

4995, 18666.666, 3200., 8920.

4996, 19133.334, 3200., 8920.

4997, 19600., 3200., 8920.

4998, 20066.666, 3200., 8920.

4999, 20533.334, 3200., 8920.

5000, 20533.334, 3200., 8700.

5001, 20066.666, 3200., 8700.

5002, 19600., 3200., 8700.

5003, 19133.334, 3200., 8700.

5004, 18666.666, 3200., 8700.

5005, 20533.334, 6000., 8920.

5006, 20066.666, 6000., 8920.

5007, 19600., 6000., 8920.

5008, 19133.334, 6000., 8920.

5009, 18666.666, 6000., 8920.

5010, 18666.666, 6000., 8700.

5011, 19133.334, 6000., 8700.

5012, 19600., 6000., 8700.

5013, 20066.666, 6000., 8700.

5014, 20533.334, 6000., 8700.

5015, 18666.666, 6200., 8920.

5016, 19133.334, 6200., 8920.

5017, 19600., 6200., 8920.

5018, 20066.666, 6200., 8920.

5019, 20533.334, 6200., 8920.

5020, 20533.334, 6200., 8700.

5021, 20066.666, 6200., 8700.

5022, 19600., 6200., 8700.

5023, 19133.334, 6200., 8700.

5024, 18666.666, 6200., 8700.

5025, 20533.334, 9000., 8920.

5026, 20066.666, 9000., 8920.

5027, 19600., 9000., 8920.

5028, 19133.334, 9000., 8920.

5029, 18666.666, 9000., 8920.

5030, 18666.666, 9000., 8700.

5031, 19133.334, 9000., 8700.

5032, 19600., 9000., 8700.

5033, 20066.666, 9000., 8700.

5034, 20533.334, 9000., 8700.

5035, 18666.666, 9200., 8920.

5036, 19133.334, 9200., 8920.

5037, 19600., 9200., 8920.

5038, 20066.666, 9200., 8920.

5039, 20533.334, 9200., 8920.

5040, 20533.334, 9200., 8700.

5041, 20066.666, 9200., 8700.

5042, 19600., 9200., 8700.

5043, 19133.334, 9200., 8700.

5044, 18666.666, 9200., 8700.

5045, 21666.666, 3000., 2700.

5046, 22133.334, 3000., 2700.

5047, 22600., 3000., 2700.

5048, 23066.666, 3000., 2700.

5049, 23533.334, 3000., 2700.

5050, 23533.334, 3000., 2920.

5051, 23066.666, 3000., 2920.

5052, 22600., 3000., 2920.

5053, 22133.334, 3000., 2920.

5054, 21666.666, 3000., 2920.

5055, 23533.334, 3200., 2700.

5056, 23066.666, 3200., 2700.

5057, 22600., 3200., 2700.

5058, 22133.334, 3200., 2700.

5059, 21666.666, 3200., 2700.

5060, 21666.666, 3200., 2920.

5061, 22133.334, 3200., 2920.

5062, 22600., 3200., 2920.

5063, 23066.666, 3200., 2920.

5064, 23533.334, 3200., 2920.

5065, 21666.666, 6000., 2700.

5066, 22133.334, 6000., 2700.

5067, 22600., 6000., 2700.

5068, 23066.666, 6000., 2700.

5069, 23533.334, 6000., 2700.

5070, 23533.334, 6000., 2920.

5071, 23066.666, 6000., 2920.

5072, 22600., 6000., 2920.

5073, 22133.334, 6000., 2920.

5074, 21666.666, 6000., 2920.

5075, 23533.334, 6200., 2700.

5076, 23066.666, 6200., 2700.

5077, 22600., 6200., 2700.

5078, 22133.334, 6200., 2700.

5079, 21666.666, 6200., 2700.

5080, 21666.666, 6200., 2920.

5081, 22133.334, 6200., 2920.

5082, 22600., 6200., 2920.

5083, 23066.666, 6200., 2920.

5084, 23533.334, 6200., 2920.

5085, 23533.334, 15200., 2700.

5086, 23066.666, 15200., 2700.

5087, 22600., 15200., 2700.

5088, 22133.334, 15200., 2700.

5089, 21666.666, 15200., 2700.

5090, 21666.666, 15200., 2920.

5091, 22133.334, 15200., 2920.

5092, 22600., 15200., 2920.

5093, 23066.666, 15200., 2920.

5094, 23533.334, 15200., 2920.

5095, 21666.666, 15000., 2700.

5096, 22133.334, 15000., 2700.

5097, 22600., 15000., 2700.

5098, 23066.666, 15000., 2700.

5099, 23533.334, 15000., 2700.

5100, 23533.334, 15000., 2920.

5101, 23066.666, 15000., 2920.

5102, 22600., 15000., 2920.

5103, 22133.334, 15000., 2920.

5104, 21666.666, 15000., 2920.

5105, 21666.666, 12000., 2700.

5106, 22133.334, 12000., 2700.

5107, 22600., 12000., 2700.

5108, 23066.666, 12000., 2700.

5109, 23533.334, 12000., 2700.

5110, 23533.334, 12000., 2920.

5111, 23066.666, 12000., 2920.

5112, 22600., 12000., 2920.

5113, 22133.334, 12000., 2920.

5114, 21666.666, 12000., 2920.

5115, 23533.334, 12200., 2700.

5116, 23066.666, 12200., 2700.

5117, 22600., 12200., 2700.

5118, 22133.334, 12200., 2700.

5119, 21666.666, 12200., 2700.

5120, 21666.666, 12200., 2920.

5121, 22133.334, 12200., 2920.

5122, 22600., 12200., 2920.

5123, 23066.666, 12200., 2920.

5124, 23533.334, 12200., 2920.

5125, 21666.666, 9000., 2700.

5126, 22133.334, 9000., 2700.

5127, 22600., 9000., 2700.

5128, 23066.666, 9000., 2700.

5129, 23533.334, 9000., 2700.

5130, 23533.334, 9000., 2920.

5131, 23066.666, 9000., 2920.

5132, 22600., 9000., 2920.

5133, 22133.334, 9000., 2920.

5134, 21666.666, 9000., 2920.

5135, 23533.334, 9200., 2700.

5136, 23066.666, 9200., 2700.

5137, 22600., 9200., 2700.

5138, 22133.334, 9200., 2700.

5139, 21666.666, 9200., 2700.

5140, 21666.666, 9200., 2920.

5141, 22133.334, 9200., 2920.

5142, 22600., 9200., 2920.

5143, 23066.666, 9200., 2920.

5144, 23533.334, 9200., 2920.

5145, 21200., 6666.6665, 2920.

5146, 21200., 7133.3335, 2920.

5147, 21200., 7600., 2920.

5148, 21200., 8066.6665, 2920.

5149, 21200., 8533.33301, 2920.

5150, 21200., 9666.66699, 2920.

5151, 21200., 10133.333, 2920.

5152, 21200., 10600., 2920.

5153, 21200., 11066.667, 2920.

5154, 21200., 11533.333, 2920.

5155, 21200., 12666.667, 2920.

5156, 21200., 13133.333, 2920.

5157, 21200., 13600., 2920.

5158, 21200., 14066.667, 2920.

5159, 21200., 14533.333, 2920.

5160, 21200., 3666.66675, 2920.

5161, 21200., 4133.3335, 2920.

5162, 21200., 4600., 2920.

5163, 21200., 5066.6665, 2920.

5164, 21200., 5533.3335, 2920.

5165, 21200., 666.666687, 2920.

5166, 21200., 1133.33337, 2920.

5167, 21200., 1600., 2920.

5168, 21200., 2066.66675, 2920.

5169, 21200., 2533.33325, 2920.

5170, 23533.334, 200., 2920.

5171, 23066.666, 200., 2920.

5172, 22600., 200., 2920.

5173, 22133.334, 200., 2920.

5174, 21666.666, 200., 2920.

5175, 23533.334, 0., 2920.

5176, 23066.666, 0., 2920.

5177, 22600., 0., 2920.

5178, 22133.334, 0., 2920.

5179, 21666.666, 0., 2920.

5180, 21200., 14533.333, 3000.

5181, 21200., 14066.667, 3000.

5182, 21200., 13600., 3000.

5183, 21200., 13133.333, 3000.

5184, 21200., 12666.667, 3000.

5185, 21200., 11533.333, 3000.

5186, 21200., 11066.667, 3000.

5187, 21200., 10600., 3000.

5188, 21200., 10133.333, 3000.

5189, 21200., 9666.66699, 3000.

5190, 21200., 8533.33301, 3000.

5191, 21200., 8066.6665, 3000.

5192, 21200., 7600., 3000.

5193, 21200., 7133.3335, 3000.

5194, 21200., 6666.6665, 3000.

5195, 21200., 5533.3335, 3000.

5196, 21200., 5066.6665, 3000.

5197, 21200., 4600., 3000.

5198, 21200., 4133.3335, 3000.

5199, 21200., 3666.66675, 3000.

5200, 21200., 2533.33325, 3000.

5201, 21200., 2066.66675, 3000.

5202, 21200., 1600., 3000.

5203, 21200., 1133.33337, 3000.

5204, 21200., 666.666687, 3000.

5205, 21666.666, 0., 3000.

5206, 22133.334, 0., 3000.

5207, 22600., 0., 3000.

5208, 23066.666, 0., 3000.

5209, 23533.334, 0., 3000.

5210, 23533.334, 15200., 3000.

5211, 23066.666, 15200., 3000.

5212, 22600., 15200., 3000.

5213, 22133.334, 15200., 3000.

5214, 21666.666, 15200., 3000.

5215, 21666.666, 0., 5700.

5216, 22133.334, 0., 5700.

5217, 22600., 0., 5700.

5218, 23066.666, 0., 5700.

5219, 23533.334, 0., 5700.

5220, 23533.334, 0., 5920.

5221, 23066.666, 0., 5920.

5222, 22600., 0., 5920.

5223, 22133.334, 0., 5920.

5224, 21666.666, 0., 5920.

5225, 23533.334, 200., 5700.

5226, 23066.666, 200., 5700.

5227, 22600., 200., 5700.

5228, 22133.334, 200., 5700.

5229, 21666.666, 200., 5700.

5230, 21666.666, 200., 5920.

5231, 22133.334, 200., 5920.

5232, 22600., 200., 5920.

5233, 23066.666, 200., 5920.

5234, 23533.334, 200., 5920.

5235, 23533.334, 3000., 5920.

5236, 23066.666, 3000., 5920.

5237, 22600., 3000., 5920.

5238, 22133.334, 3000., 5920.

5239, 21666.666, 3000., 5920.

5240, 21666.666, 3000., 5700.

5241, 22133.334, 3000., 5700.

5242, 22600., 3000., 5700.

5243, 23066.666, 3000., 5700.

5244, 23533.334, 3000., 5700.

5245, 21666.666, 3200., 5920.

5246, 22133.334, 3200., 5920.

5247, 22600., 3200., 5920.

5248, 23066.666, 3200., 5920.

5249, 23533.334, 3200., 5920.

5250, 23533.334, 3200., 5700.

5251, 23066.666, 3200., 5700.

5252, 22600., 3200., 5700.

5253, 22133.334, 3200., 5700.

5254, 21666.666, 3200., 5700.

5255, 23533.334, 15000., 5920.

5256, 23066.666, 15000., 5920.

5257, 22600., 15000., 5920.

5258, 22133.334, 15000., 5920.

5259, 21666.666, 15000., 5920.

5260, 21666.666, 15000., 5700.

5261, 22133.334, 15000., 5700.

5262, 22600., 15000., 5700.

5263, 23066.666, 15000., 5700.

5264, 23533.334, 15000., 5700.

5265, 21666.666, 15200., 5920.

5266, 22133.334, 15200., 5920.

5267, 22600., 15200., 5920.

5268, 23066.666, 15200., 5920.

5269, 23533.334, 15200., 5920.

5270, 23533.334, 15200., 5700.

5271, 23066.666, 15200., 5700.

5272, 22600., 15200., 5700.

5273, 22133.334, 15200., 5700.

5274, 21666.666, 15200., 5700.

5275, 21666.666, 12200., 5920.

5276, 22133.334, 12200., 5920.

5277, 22600., 12200., 5920.

5278, 23066.666, 12200., 5920.

5279, 23533.334, 12200., 5920.

5280, 23533.334, 12200., 5700.

5281, 23066.666, 12200., 5700.

5282, 22600., 12200., 5700.

5283, 22133.334, 12200., 5700.

5284, 21666.666, 12200., 5700.

5285, 23533.334, 12000., 5920.

5286, 23066.666, 12000., 5920.

5287, 22600., 12000., 5920.

5288, 22133.334, 12000., 5920.

5289, 21666.666, 12000., 5920.

5290, 21666.666, 12000., 5700.

5291, 22133.334, 12000., 5700.

5292, 22600., 12000., 5700.

5293, 23066.666, 12000., 5700.

5294, 23533.334, 12000., 5700.

5295, 21666.666, 9200., 5920.

5296, 22133.334, 9200., 5920.

5297, 22600., 9200., 5920.

5298, 23066.666, 9200., 5920.

5299, 23533.334, 9200., 5920.

5300, 23533.334, 9200., 5700.

5301, 23066.666, 9200., 5700.

5302, 22600., 9200., 5700.

5303, 22133.334, 9200., 5700.

5304, 21666.666, 9200., 5700.

5305, 23533.334, 9000., 5920.

5306, 23066.666, 9000., 5920.

5307, 22600., 9000., 5920.

5308, 22133.334, 9000., 5920.

5309, 21666.666, 9000., 5920.

5310, 21666.666, 9000., 5700.

5311, 22133.334, 9000., 5700.

5312, 22600., 9000., 5700.

5313, 23066.666, 9000., 5700.

5314, 23533.334, 9000., 5700.

5315, 21666.666, 6200., 5920.

5316, 22133.334, 6200., 5920.

5317, 22600., 6200., 5920.

5318, 23066.666, 6200., 5920.

5319, 23533.334, 6200., 5920.

5320, 23533.334, 6200., 5700.

5321, 23066.666, 6200., 5700.

5322, 22600., 6200., 5700.

5323, 22133.334, 6200., 5700.

5324, 21666.666, 6200., 5700.

5325, 23533.334, 6000., 5920.

5326, 23066.666, 6000., 5920.

5327, 22600., 6000., 5920.

5328, 22133.334, 6000., 5920.

5329, 21666.666, 6000., 5920.

5330, 21666.666, 6000., 5700.

5331, 22133.334, 6000., 5700.

5332, 22600., 6000., 5700.

5333, 23066.666, 6000., 5700.

5334, 23533.334, 6000., 5700.

5335, 21666.666, 0., 8700.

5336, 22133.334, 0., 8700.

5337, 22600., 0., 8700.

5338, 23066.666, 0., 8700.

5339, 23533.334, 0., 8700.

5340, 23533.334, 0., 8920.

5341, 23066.666, 0., 8920.

5342, 22600., 0., 8920.

5343, 22133.334, 0., 8920.

5344, 21666.666, 0., 8920.

5345, 23533.334, 200., 8700.

5346, 23066.666, 200., 8700.

5347, 22600., 200., 8700.

5348, 22133.334, 200., 8700.

5349, 21666.666, 200., 8700.

5350, 21666.666, 200., 8920.

5351, 22133.334, 200., 8920.

5352, 22600., 200., 8920.

5353, 23066.666, 200., 8920.

5354, 23533.334, 200., 8920.

5355, 23533.334, 3000., 8920.

5356, 23066.666, 3000., 8920.

5357, 22600., 3000., 8920.

5358, 22133.334, 3000., 8920.

5359, 21666.666, 3000., 8920.

5360, 21666.666, 3000., 8700.

5361, 22133.334, 3000., 8700.

5362, 22600., 3000., 8700.

5363, 23066.666, 3000., 8700.

5364, 23533.334, 3000., 8700.

5365, 21666.666, 3200., 8920.

5366, 22133.334, 3200., 8920.

5367, 22600., 3200., 8920.

5368, 23066.666, 3200., 8920.

5369, 23533.334, 3200., 8920.

5370, 23533.334, 3200., 8700.

5371, 23066.666, 3200., 8700.

5372, 22600., 3200., 8700.

5373, 22133.334, 3200., 8700.

5374, 21666.666, 3200., 8700.

5375, 21666.666, 9200., 8920.

5376, 22133.334, 9200., 8920.

5377, 22600., 9200., 8920.

5378, 23066.666, 9200., 8920.

5379, 23533.334, 9200., 8920.

5380, 23533.334, 9200., 8700.

5381, 23066.666, 9200., 8700.

5382, 22600., 9200., 8700.

5383, 22133.334, 9200., 8700.

5384, 21666.666, 9200., 8700.

5385, 23533.334, 9000., 8920.

5386, 23066.666, 9000., 8920.

5387, 22600., 9000., 8920.

5388, 22133.334, 9000., 8920.

5389, 21666.666, 9000., 8920.

5390, 21666.666, 9000., 8700.

5391, 22133.334, 9000., 8700.

5392, 22600., 9000., 8700.

5393, 23066.666, 9000., 8700.

5394, 23533.334, 9000., 8700.

5395, 21666.666, 6200., 8920.

5396, 22133.334, 6200., 8920.

5397, 22600., 6200., 8920.

5398, 23066.666, 6200., 8920.

5399, 23533.334, 6200., 8920.

5400, 23533.334, 6200., 8700.

5401, 23066.666, 6200., 8700.

5402, 22600., 6200., 8700.

5403, 22133.334, 6200., 8700.

5404, 21666.666, 6200., 8700.

5405, 23533.334, 6000., 8920.

5406, 23066.666, 6000., 8920.

5407, 22600., 6000., 8920.

5408, 22133.334, 6000., 8920.

5409, 21666.666, 6000., 8920.

5410, 21666.666, 6000., 8700.

5411, 22133.334, 6000., 8700.

5412, 22600., 6000., 8700.

5413, 23066.666, 6000., 8700.

5414, 23533.334, 6000., 8700.

5415, 23533.334, 15000., 8920.

5416, 23066.666, 15000., 8920.

5417, 22600., 15000., 8920.

5418, 22133.334, 15000., 8920.

5419, 21666.666, 15000., 8920.

5420, 21666.666, 15000., 8700.

5421, 22133.334, 15000., 8700.

5422, 22600., 15000., 8700.

5423, 23066.666, 15000., 8700.

5424, 23533.334, 15000., 8700.

5425, 21666.666, 15200., 8920.

5426, 22133.334, 15200., 8920.

5427, 22600., 15200., 8920.

5428, 23066.666, 15200., 8920.

5429, 23533.334, 15200., 8920.

5430, 23533.334, 15200., 8700.

5431, 23066.666, 15200., 8700.

5432, 22600., 15200., 8700.

5433, 22133.334, 15200., 8700.

5434, 21666.666, 15200., 8700.

5435, 21200., 3760., 8920.

5436, 21200., 4320., 8920.

5437, 21200., 4880., 8920.

5438, 21200., 5440., 8920.

5439, 21200., 6666.6665, 8920.

5440, 21200., 7133.3335, 8920.

5441, 21200., 7600., 8920.

5442, 21200., 8066.6665, 8920.

5443, 21200., 8533.33301, 8920.

5444, 21200., 9666.66699, 8920.

5445, 21200., 10133.333, 8920.

5446, 21200., 10600., 8920.

5447, 21200., 11066.667, 8920.

5448, 21200., 11533.333, 8920.

5449, 21666.666, 12000., 8920.

5450, 22133.334, 12000., 8920.

5451, 22600., 12000., 8920.

5452, 23066.666, 12000., 8920.

5453, 23533.334, 12000., 8920.

5454, 21666.666, 12200., 8920.

5455, 22133.334, 12200., 8920.

5456, 22600., 12200., 8920.

5457, 23066.666, 12200., 8920.

5458, 23533.334, 12200., 8920.

5459, 21200., 12666.667, 8920.

5460, 21200., 13133.333, 8920.

5461, 21200., 13600., 8920.

5462, 21200., 14066.667, 8920.

5463, 21200., 14533.333, 8920.

5464, 21200., 666.666687, 8920.

5465, 21200., 1133.33337, 8920.

5466, 21200., 1600., 8920.

5467, 21200., 2066.66675, 8920.

5468, 21200., 2533.33325, 8920.

5469, 23533.334, 15200., 9000.

5470, 23066.666, 15200., 9000.

5471, 22600., 15200., 9000.

5472, 22133.334, 15200., 9000.

5473, 21666.666, 15200., 9000.

5474, 21666.666, 0., 9000.

5475, 22133.334, 0., 9000.

5476, 22600., 0., 9000.

5477, 23066.666, 0., 9000.

5478, 23533.334, 0., 9000.

5479, 21200., 14533.333, 9000.

5480, 21200., 14066.667, 9000.

5481, 21200., 13600., 9000.

5482, 21200., 13133.333, 9000.

5483, 21200., 12666.667, 9000.

5484, 21200., 11533.333, 9000.

5485, 21200., 11066.667, 9000.

5486, 21200., 10600., 9000.

5487, 21200., 10133.333, 9000.

5488, 21200., 9666.66699, 9000.

5489, 21200., 8533.33301, 9000.

5490, 21200., 8066.6665, 9000.

5491, 21200., 7600., 9000.

5492, 21200., 7133.3335, 9000.

5493, 21200., 6666.6665, 9000.

5494, 21200., 5440., 9000.

5495, 21200., 4880., 9000.

5496, 21200., 4320., 9000.

5497, 21200., 3760., 9000.

5498, 21200., 2533.33325, 9000.

5499, 21200., 2066.66675, 9000.

5500, 21200., 1600., 9000.

5501, 21200., 1133.33337, 9000.

5502, 21200., 666.666687, 9000.

5503, 21000., 2533.33325, 8700.

5504, 21000., 2066.66675, 8700.

5505, 21000., 1600., 8700.

5506, 21000., 1133.33337, 8700.

5507, 21000., 666.666687, 8700.

5508, 21000., 666.666687, 8920.

5509, 21000., 1133.33337, 8920.

5510, 21000., 1600., 8920.

5511, 21000., 2066.66675, 8920.

5512, 21000., 2533.33325, 8920.

5513, 21000., 666.666687, 9000.

5514, 21000., 1133.33337, 9000.

5515, 21000., 1600., 9000.

5516, 21000., 2066.66675, 9000.

5517, 21000., 2533.33325, 9000.

5518, 21200., 666.666687, 8700.

5519, 21200., 1133.33337, 8700.

5520, 21200., 1600., 8700.

5521, 21200., 2066.66675, 8700.

5522, 21200., 2533.33325, 8700.

5523, 21200., 2533.33325, 6000.

5524, 21200., 2066.66675, 6000.

5525, 21200., 1600., 6000.

5526, 21200., 1133.33337, 6000.

5527, 21200., 666.666687, 6000.

5528, 21200., 666.666687, 5920.

5529, 21200., 1133.33337, 5920.

5530, 21200., 1600., 5920.

5531, 21200., 2066.66675, 5920.

5532, 21200., 2533.33325, 5920.

5533, 21200., 666.666687, 5700.

5534, 21200., 1133.33337, 5700.

5535, 21200., 1600., 5700.

5536, 21200., 2066.66675, 5700.

5537, 21200., 2533.33325, 5700.

5538, 21000., 666.666687, 6000.

5539, 21000., 1133.33337, 6000.

5540, 21000., 1600., 6000.

5541, 21000., 2066.66675, 6000.

5542, 21000., 2533.33325, 6000.

5543, 21000., 2533.33325, 5920.

5544, 21000., 2066.66675, 5920.

5545, 21000., 1600., 5920.

5546, 21000., 1133.33337, 5920.

5547, 21000., 666.666687, 5920.

5548, 21000., 2533.33325, 5700.

5549, 21000., 2066.66675, 5700.

5550, 21000., 1600., 5700.

5551, 21000., 1133.33337, 5700.

5552, 21000., 666.666687, 5700.

5553, 21000., 2533.33325, 2700.

5554, 21000., 2066.66675, 2700.

5555, 21000., 1600., 2700.

5556, 21000., 1133.33337, 2700.

5557, 21000., 666.666687, 2700.

5558, 21200., 666.666687, 2700.

5559, 21200., 1133.33337, 2700.

5560, 21200., 1600., 2700.

5561, 21200., 2066.66675, 2700.

5562, 21200., 2533.33325, 2700.

5563, 21200., 3666.66675, 2700.

5564, 21200., 4133.3335, 2700.

5565, 21200., 4600., 2700.

5566, 21200., 5066.6665, 2700.

5567, 21200., 5533.3335, 2700.

5568, 21000., 5533.3335, 2700.

5569, 21000., 5066.6665, 2700.

5570, 21000., 4600., 2700.

5571, 21000., 4133.3335, 2700.

5572, 21000., 3666.66675, 2700.

5573, 21000., 5533.3335, 5700.

5574, 21000., 5066.6665, 5700.

5575, 21000., 4600., 5700.

5576, 21000., 4133.3335, 5700.

5577, 21000., 3666.66675, 5700.

5578, 21000., 3666.66675, 5920.

5579, 21000., 4133.3335, 5920.

5580, 21000., 4600., 5920.

5581, 21000., 5066.6665, 5920.

5582, 21000., 5533.3335, 5920.

5583, 21000., 3666.66675, 6000.

5584, 21000., 4133.3335, 6000.

5585, 21000., 4600., 6000.

5586, 21000., 5066.6665, 6000.

5587, 21000., 5533.3335, 6000.

5588, 21200., 3666.66675, 5700.

5589, 21200., 4133.3335, 5700.

5590, 21200., 4600., 5700.

5591, 21200., 5066.6665, 5700.

5592, 21200., 5533.3335, 5700.

5593, 21200., 5533.3335, 5920.

5594, 21200., 5066.6665, 5920.

5595, 21200., 4600., 5920.

5596, 21200., 4133.3335, 5920.

5597, 21200., 3666.66675, 5920.

5598, 21200., 5533.3335, 6000.

5599, 21200., 5066.6665, 6000.

5600, 21200., 4600., 6000.

5601, 21200., 4133.3335, 6000.

5602, 21200., 3666.66675, 6000.

5603, 21200., 6000., 8160.

5604, 21200., 6000., 7620.

5605, 21200., 6000., 7080.

5606, 21200., 6000., 6540.

5607, 21200., 6200., 6540.

5608, 21200., 6200., 7080.

5609, 21200., 6200., 7620.

5610, 21200., 6200., 8160.

5611, 21200., 6000., 5160.

5612, 21200., 6000., 4620.

5613, 21200., 6000., 4080.

5614, 21200., 6000., 3540.

5615, 21200., 6200., 3540.

5616, 21200., 6200., 4080.

5617, 21200., 6200., 4620.

5618, 21200., 6200., 5160.

5619, 21200., 6000., 2160.

5620, 21200., 6000., 1620.

5621, 21200., 6000., 1080.

5622, 21200., 6000., 540.

5623, 21200., 6200., 540.

5624, 21200., 6200., 1080.

5625, 21200., 6200., 1620.

5626, 21200., 6200., 2160.

5627, 21000., 6200., 8160.

5628, 21000., 6200., 7620.

5629, 21000., 6200., 7080.

5630, 21000., 6200., 6540.

5631, 21000., 6000., 6540.

5632, 21000., 6000., 7080.

5633, 21000., 6000., 7620.

5634, 21000., 6000., 8160.

5635, 21000., 6200., 5160.

5636, 21000., 6200., 4620.

5637, 21000., 6200., 4080.

5638, 21000., 6200., 3540.

5639, 21000., 6000., 3540.

5640, 21000., 6000., 4080.

5641, 21000., 6000., 4620.

5642, 21000., 6000., 5160.

5643, 21000., 6200., 2160.

5644, 21000., 6200., 1620.

5645, 21000., 6200., 1080.

5646, 21000., 6200., 540.

5647, 21000., 6000., 540.

5648, 21000., 6000., 1080.

5649, 21000., 6000., 1620.

5650, 21000., 6000., 2160.

5651, 21200., 6666.6665, 8700.

5652, 21200., 7133.3335, 8700.

5653, 21200., 7600., 8700.

5654, 21200., 8066.6665, 8700.

5655, 21200., 8533.33301, 8700.

5656, 21000., 6666.6665, 9000.

5657, 21000., 7133.3335, 9000.

5658, 21000., 7600., 9000.

5659, 21000., 8066.6665, 9000.

5660, 21000., 8533.33301, 9000.

5661, 21000., 8533.33301, 8920.

5662, 21000., 8066.6665, 8920.

5663, 21000., 7600., 8920.

5664, 21000., 7133.3335, 8920.

5665, 21000., 6666.6665, 8920.

5666, 21000., 8533.33301, 8700.

5667, 21000., 8066.6665, 8700.

5668, 21000., 7600., 8700.

5669, 21000., 7133.3335, 8700.

5670, 21000., 6666.6665, 8700.

5671, 21000., 8533.33301, 5700.

5672, 21000., 8066.6665, 5700.

5673, 21000., 7600., 5700.

5674, 21000., 7133.3335, 5700.

5675, 21000., 6666.6665, 5700.

5676, 21000., 6666.6665, 5920.

5677, 21000., 7133.3335, 5920.

5678, 21000., 7600., 5920.

5679, 21000., 8066.6665, 5920.

5680, 21000., 8533.33301, 5920.

5681, 21000., 6666.6665, 6000.

5682, 21000., 7133.3335, 6000.

5683, 21000., 7600., 6000.

5684, 21000., 8066.6665, 6000.

5685, 21000., 8533.33301, 6000.

5686, 21200., 6666.6665, 5700.

5687, 21200., 7133.3335, 5700.

5688, 21200., 7600., 5700.

5689, 21200., 8066.6665, 5700.

5690, 21200., 8533.33301, 5700.

5691, 21200., 8533.33301, 5920.

5692, 21200., 8066.6665, 5920.

5693, 21200., 7600., 5920.

5694, 21200., 7133.3335, 5920.

5695, 21200., 6666.6665, 5920.

5696, 21200., 8533.33301, 6000.

5697, 21200., 8066.6665, 6000.

5698, 21200., 7600., 6000.

5699, 21200., 7133.3335, 6000.

5700, 21200., 6666.6665, 6000.

5701, 21200., 6666.6665, 2700.

5702, 21200., 7133.3335, 2700.

5703, 21200., 7600., 2700.

5704, 21200., 8066.6665, 2700.

5705, 21200., 8533.33301, 2700.

5706, 21000., 8533.33301, 2700.

5707, 21000., 8066.6665, 2700.

5708, 21000., 7600., 2700.

5709, 21000., 7133.3335, 2700.

5710, 21000., 6666.6665, 2700.

5711, 21200., 9666.66699, 2700.

5712, 21200., 10133.333, 2700.

5713, 21200., 10600., 2700.

5714, 21200., 11066.667, 2700.

5715, 21200., 11533.333, 2700.

5716, 21000., 11533.333, 2700.

5717, 21000., 11066.667, 2700.

5718, 21000., 10600., 2700.

5719, 21000., 10133.333, 2700.

5720, 21000., 9666.66699, 2700.

5721, 21000., 11533.333, 5700.

5722, 21000., 11066.667, 5700.

5723, 21000., 10600., 5700.

5724, 21000., 10133.333, 5700.

5725, 21000., 9666.66699, 5700.

5726, 21000., 9666.66699, 5920.

5727, 21000., 10133.333, 5920.

5728, 21000., 10600., 5920.

5729, 21000., 11066.667, 5920.

5730, 21000., 11533.333, 5920.

5731, 21000., 9666.66699, 6000.

5732, 21000., 10133.333, 6000.

5733, 21000., 10600., 6000.

5734, 21000., 11066.667, 6000.

5735, 21000., 11533.333, 6000.

5736, 21200., 9666.66699, 5700.

5737, 21200., 10133.333, 5700.

5738, 21200., 10600., 5700.

5739, 21200., 11066.667, 5700.

5740, 21200., 11533.333, 5700.

5741, 21200., 11533.333, 5920.

5742, 21200., 11066.667, 5920.

5743, 21200., 10600., 5920.

5744, 21200., 10133.333, 5920.

5745, 21200., 9666.66699, 5920.

5746, 21200., 11533.333, 6000.

5747, 21200., 11066.667, 6000.

5748, 21200., 10600., 6000.

5749, 21200., 10133.333, 6000.

5750, 21200., 9666.66699, 6000.

5751, 21200., 12000., 8160.

5752, 21200., 12000., 7620.

5753, 21200., 12000., 7080.

5754, 21200., 12000., 6540.

5755, 21200., 12200., 6540.

5756, 21200., 12200., 7080.

5757, 21200., 12200., 7620.

5758, 21200., 12200., 8160.

5759, 21200., 12000., 5160.

5760, 21200., 12000., 4620.

5761, 21200., 12000., 4080.

5762, 21200., 12000., 3540.

5763, 21200., 12200., 3540.

5764, 21200., 12200., 4080.

5765, 21200., 12200., 4620.

5766, 21200., 12200., 5160.

5767, 21200., 12000., 2160.

5768, 21200., 12000., 1620.

5769, 21200., 12000., 1080.

5770, 21200., 12000., 540.

5771, 21200., 12200., 540.

5772, 21200., 12200., 1080.

5773, 21200., 12200., 1620.

5774, 21200., 12200., 2160.

5775, 21000., 12200., 8160.

5776, 21000., 12200., 7620.

5777, 21000., 12200., 7080.

5778, 21000., 12200., 6540.

5779, 21000., 12000., 6540.

5780, 21000., 12000., 7080.

5781, 21000., 12000., 7620.

5782, 21000., 12000., 8160.

5783, 21000., 12200., 5160.

5784, 21000., 12200., 4620.

5785, 21000., 12200., 4080.

5786, 21000., 12200., 3540.

5787, 21000., 12000., 3540.

5788, 21000., 12000., 4080.

5789, 21000., 12000., 4620.

5790, 21000., 12000., 5160.

5791, 21000., 12200., 2160.

5792, 21000., 12200., 1620.

5793, 21000., 12200., 1080.

5794, 21000., 12200., 540.

5795, 21000., 12000., 540.

5796, 21000., 12000., 1080.

5797, 21000., 12000., 1620.

5798, 21000., 12000., 2160.

5799, 21000., 14533.333, 8700.

5800, 21000., 14066.667, 8700.

5801, 21000., 13600., 8700.

5802, 21000., 13133.333, 8700.

5803, 21000., 12666.667, 8700.

5804, 21000., 12666.667, 8920.

5805, 21000., 13133.333, 8920.

5806, 21000., 13600., 8920.

5807, 21000., 14066.667, 8920.

5808, 21000., 14533.333, 8920.

5809, 21000., 12666.667, 9000.

5810, 21000., 13133.333, 9000.

5811, 21000., 13600., 9000.

5812, 21000., 14066.667, 9000.

5813, 21000., 14533.333, 9000.

5814, 21200., 12666.667, 8700.

5815, 21200., 13133.333, 8700.

5816, 21200., 13600., 8700.

5817, 21200., 14066.667, 8700.

5818, 21200., 14533.333, 8700.

5819, 21200., 14533.333, 6000.

5820, 21200., 14066.667, 6000.

5821, 21200., 13600., 6000.

5822, 21200., 13133.333, 6000.

5823, 21200., 12666.667, 6000.

5824, 21200., 12666.667, 5920.

5825, 21200., 13133.333, 5920.

5826, 21200., 13600., 5920.

5827, 21200., 14066.667, 5920.

5828, 21200., 14533.333, 5920.

5829, 21200., 12666.667, 5700.

5830, 21200., 13133.333, 5700.

5831, 21200., 13600., 5700.

5832, 21200., 14066.667, 5700.

5833, 21200., 14533.333, 5700.

5834, 21000., 12666.667, 6000.

5835, 21000., 13133.333, 6000.

5836, 21000., 13600., 6000.

5837, 21000., 14066.667, 6000.

5838, 21000., 14533.333, 6000.

5839, 21000., 14533.333, 5920.

5840, 21000., 14066.667, 5920.

5841, 21000., 13600., 5920.

5842, 21000., 13133.333, 5920.

5843, 21000., 12666.667, 5920.

5844, 21000., 14533.333, 5700.

5845, 21000., 14066.667, 5700.

5846, 21000., 13600., 5700.

5847, 21000., 13133.333, 5700.

5848, 21000., 12666.667, 5700.

5849, 21200., 15000., 8160.

5850, 21200., 15000., 7620.

5851, 21200., 15000., 7080.

5852, 21200., 15000., 6540.

5853, 21200., 15200., 6540.

5854, 21200., 15200., 7080.

5855, 21200., 15200., 7620.

5856, 21200., 15200., 8160.

5857, 21200., 15000., 5160.

5858, 21200., 15000., 4620.

5859, 21200., 15000., 4080.

5860, 21200., 15000., 3540.

5861, 21200., 15200., 3540.

5862, 21200., 15200., 4080.

5863, 21200., 15200., 4620.

5864, 21200., 15200., 5160.

5865, 21200., 15000., 2160.

5866, 21200., 15000., 1620.

5867, 21200., 15000., 1080.

5868, 21200., 15000., 540.

5869, 21200., 15200., 540.

5870, 21200., 15200., 1080.

5871, 21200., 15200., 1620.

5872, 21200., 15200., 2160.

5873, 21000., 15200., 8160.

5874, 21000., 15200., 7620.

5875, 21000., 15200., 7080.

5876, 21000., 15200., 6540.

5877, 21000., 15000., 6540.

5878, 21000., 15000., 7080.

5879, 21000., 15000., 7620.

5880, 21000., 15000., 8160.

5881, 21000., 15200., 5160.

5882, 21000., 15200., 4620.

5883, 21000., 15200., 4080.

5884, 21000., 15200., 3540.

5885, 21000., 15000., 3540.

5886, 21000., 15000., 4080.

5887, 21000., 15000., 4620.

5888, 21000., 15000., 5160.

5889, 21000., 15200., 2160.

5890, 21000., 15200., 1620.

5891, 21000., 15200., 1080.

5892, 21000., 15200., 540.

5893, 21000., 15000., 540.

5894, 21000., 15000., 1080.

5895, 21000., 15000., 1620.

5896, 21000., 15000., 2160.

5897, 21200., 200., 8160.

5898, 21200., 200., 7620.

5899, 21200., 200., 7080.

5900, 21200., 200., 6540.

5901, 21000., 200., 6540.

5902, 21000., 200., 7080.

5903, 21000., 200., 7620.

5904, 21000., 200., 8160.

5905, 21200., 200., 5160.

5906, 21200., 200., 4620.

5907, 21200., 200., 4080.

5908, 21200., 200., 3540.

5909, 21000., 200., 3540.

5910, 21000., 200., 4080.

5911, 21000., 200., 4620.

5912, 21000., 200., 5160.

5913, 21000., 200., 540.

5914, 21000., 200., 1080.

5915, 21000., 200., 1620.

5916, 21000., 200., 2160.

5917, 21200., 200., 2160.

5918, 21200., 200., 1620.

5919, 21200., 200., 1080.

5920, 21200., 200., 540.

5921, 21200., 0., 6540.

5922, 21200., 0., 7080.

5923, 21200., 0., 7620.

5924, 21200., 0., 8160.

5925, 21200., 0., 3540.

5926, 21200., 0., 4080.

5927, 21200., 0., 4620.

5928, 21200., 0., 5160.

5929, 21200., 0., 540.

5930, 21200., 0., 1080.

5931, 21200., 0., 1620.

5932, 21200., 0., 2160.

5933, 21000., 0., 8160.

5934, 21000., 0., 7620.

5935, 21000., 0., 7080.

5936, 21000., 0., 6540.

5937, 21000., 0., 5160.

5938, 21000., 0., 4620.

5939, 21000., 0., 4080.

5940, 21000., 0., 3540.

5941, 21000., 0., 2160.

5942, 21000., 0., 1620.

5943, 21000., 0., 1080.

5944, 21000., 0., 540.

5945, 21000., 3200., 2160.

5946, 21000., 3200., 1620.

5947, 21000., 3200., 1080.

5948, 21000., 3200., 540.

5949, 21000., 3000., 540.

5950, 21000., 3000., 1080.

5951, 21000., 3000., 1620.

5952, 21000., 3000., 2160.

5953, 21000., 3200., 5160.

5954, 21000., 3200., 4620.

5955, 21000., 3200., 4080.

5956, 21000., 3200., 3540.

5957, 21000., 3000., 3540.

5958, 21000., 3000., 4080.

5959, 21000., 3000., 4620.

5960, 21000., 3000., 5160.

5961, 21000., 3200., 8160.

5962, 21000., 3200., 7620.

5963, 21000., 3200., 7080.

5964, 21000., 3200., 6540.

5965, 21000., 3000., 6540.

5966, 21000., 3000., 7080.

5967, 21000., 3000., 7620.

5968, 21000., 3000., 8160.

5969, 21200., 3200., 540.

5970, 21200., 3200., 1080.

5971, 21200., 3200., 1620.

5972, 21200., 3200., 2160.

5973, 21200., 3000., 2160.

5974, 21200., 3000., 1620.

5975, 21200., 3000., 1080.

5976, 21200., 3000., 540.

5977, 21200., 3000., 5160.

5978, 21200., 3000., 4620.

5979, 21200., 3000., 4080.

5980, 21200., 3000., 3540.

5981, 21200., 3200., 3540.

5982, 21200., 3200., 4080.

5983, 21200., 3200., 4620.

5984, 21200., 3200., 5160.

5985, 21200., 3000., 8160.

5986, 21200., 3000., 7620.

5987, 21200., 3000., 7080.

5988, 21200., 3000., 6540.

5989, 21200., 3200., 6540.

5990, 21200., 3200., 7080.

5991, 21200., 3200., 7620.

5992, 21200., 3200., 8160.

5993, 21200., 3760., 8700.

5994, 21200., 4320., 8700.

5995, 21200., 4880., 8700.

5996, 21200., 5440., 8700.

5997, 21000., 3760., 9000.

5998, 21000., 4320., 9000.

5999, 21000., 4880., 9000.

6000, 21000., 5440., 9000.

6001, 21000., 5440., 8920.

6002, 21000., 4880., 8920.

6003, 21000., 4320., 8920.

6004, 21000., 3760., 8920.

6005, 21000., 5440., 8700.

6006, 21000., 4880., 8700.

6007, 21000., 4320., 8700.

6008, 21000., 3760., 8700.

6009, 21200., 9000., 8160.

6010, 21200., 9000., 7620.

6011, 21200., 9000., 7080.

6012, 21200., 9000., 6540.

6013, 21200., 9200., 6540.

6014, 21200., 9200., 7080.

6015, 21200., 9200., 7620.

6016, 21200., 9200., 8160.

6017, 21200., 9000., 5160.

6018, 21200., 9000., 4620.

6019, 21200., 9000., 4080.

6020, 21200., 9000., 3540.

6021, 21200., 9200., 3540.

6022, 21200., 9200., 4080.

6023, 21200., 9200., 4620.

6024, 21200., 9200., 5160.

6025, 21200., 9000., 2160.

6026, 21200., 9000., 1620.

6027, 21200., 9000., 1080.

6028, 21200., 9000., 540.

6029, 21200., 9200., 540.

6030, 21200., 9200., 1080.

6031, 21200., 9200., 1620.

6032, 21200., 9200., 2160.

6033, 21000., 9200., 8160.

6034, 21000., 9200., 7620.

6035, 21000., 9200., 7080.

6036, 21000., 9200., 6540.

6037, 21000., 9000., 6540.

6038, 21000., 9000., 7080.

6039, 21000., 9000., 7620.

6040, 21000., 9000., 8160.

6041, 21000., 9200., 5160.

6042, 21000., 9200., 4620.

6043, 21000., 9200., 4080.

6044, 21000., 9200., 3540.

6045, 21000., 9000., 3540.

6046, 21000., 9000., 4080.

6047, 21000., 9000., 4620.

6048, 21000., 9000., 5160.

6049, 21000., 9000., 540.

6050, 21000., 9000., 1080.

6051, 21000., 9000., 1620.

6052, 21000., 9000., 2160.

6053, 21000., 9200., 2160.

6054, 21000., 9200., 1620.

6055, 21000., 9200., 1080.

6056, 21000., 9200., 540.

6057, 21200., 9666.66699, 8700.

6058, 21200., 10133.333, 8700.

6059, 21200., 10600., 8700.

6060, 21200., 11066.667, 8700.

6061, 21200., 11533.333, 8700.

6062, 21000., 9666.66699, 9000.

6063, 21000., 10133.333, 9000.

6064, 21000., 10600., 9000.

6065, 21000., 11066.667, 9000.

6066, 21000., 11533.333, 9000.

6067, 21000., 11533.333, 8920.

6068, 21000., 11066.667, 8920.

6069, 21000., 10600., 8920.

6070, 21000., 10133.333, 8920.

6071, 21000., 9666.66699, 8920.

6072, 21000., 11533.333, 8700.

6073, 21000., 11066.667, 8700.

6074, 21000., 10600., 8700.

6075, 21000., 10133.333, 8700.

6076, 21000., 9666.66699, 8700.

6077, 18200., 666.666687, 2700.

6078, 18200., 1133.33337, 2700.

6079, 18200., 1600., 2700.

6080, 18200., 2066.66675, 2700.

6081, 18200., 2533.33325, 2700.

6082, 18000., 2533.33325, 2700.

6083, 18000., 2066.66675, 2700.

6084, 18000., 1600., 2700.

6085, 18000., 1133.33337, 2700.

6086, 18000., 666.666687, 2700.

6087, 18200., 666.666687, 5920.

6088, 18200., 1133.33337, 5920.

6089, 18200., 1600., 5920.

6090, 18200., 2066.66675, 5920.

6091, 18200., 2533.33325, 5920.

6092, 18200., 2533.33325, 6000.

6093, 18200., 2066.66675, 6000.

6094, 18200., 1600., 6000.

6095, 18200., 1133.33337, 6000.

6096, 18200., 666.666687, 6000.

6097, 18200., 666.666687, 5700.

6098, 18200., 1133.33337, 5700.

6099, 18200., 1600., 5700.

6100, 18200., 2066.66675, 5700.

6101, 18200., 2533.33325, 5700.

6102, 18000., 2533.33325, 5700.

6103, 18000., 2066.66675, 5700.

6104, 18000., 1600., 5700.

6105, 18000., 1133.33337, 5700.

6106, 18000., 666.666687, 5700.

6107, 18000., 200., 540.

6108, 18000., 200., 1080.

6109, 18000., 200., 1620.

6110, 18000., 200., 2160.

6111, 18200., 200., 2160.

6112, 18200., 200., 1620.

6113, 18200., 200., 1080.

6114, 18200., 200., 540.

6115, 18200., 200., 5160.

6116, 18200., 200., 4620.

6117, 18200., 200., 4080.

6118, 18200., 200., 3540.

6119, 18000., 200., 3540.

6120, 18000., 200., 4080.

6121, 18000., 200., 4620.

6122, 18000., 200., 5160.

6123, 18200., 200., 8160.

6124, 18200., 200., 7620.

6125, 18200., 200., 7080.

6126, 18200., 200., 6540.

6127, 18000., 200., 6540.

6128, 18000., 200., 7080.

6129, 18000., 200., 7620.

6130, 18000., 200., 8160.

6131, 18000., 0., 2160.

6132, 18000., 0., 1620.

6133, 18000., 0., 1080.

6134, 18000., 0., 540.

6135, 18000., 0., 5160.

6136, 18000., 0., 4620.

6137, 18000., 0., 4080.

6138, 18000., 0., 3540.

6139, 18000., 0., 8160.

6140, 18000., 0., 7620.

6141, 18000., 0., 7080.

6142, 18000., 0., 6540.

6143, 18200., 0., 540.

6144, 18200., 0., 1080.

6145, 18200., 0., 1620.

6146, 18200., 0., 2160.

6147, 18200., 0., 3540.

6148, 18200., 0., 4080.

6149, 18200., 0., 4620.

6150, 18200., 0., 5160.

6151, 18200., 0., 6540.

6152, 18200., 0., 7080.

6153, 18200., 0., 7620.

6154, 18200., 0., 8160.

6155, 18200., 3760., 8700.

6156, 18200., 4320., 8700.

6157, 18200., 4880., 8700.

6158, 18200., 5440., 8700.

6159, 18200., 5440., 8920.

6160, 18200., 4880., 8920.

6161, 18200., 4320., 8920.

6162, 18200., 3760., 8920.

6163, 18200., 5440., 9000.

6164, 18200., 4880., 9000.

6165, 18200., 4320., 9000.

6166, 18200., 3760., 9000.

6167, 18000., 5440., 8700.

6168, 18000., 4880., 8700.

6169, 18000., 4320., 8700.

6170, 18000., 3760., 8700.

6171, 18000., 3760., 8920.

6172, 18000., 4320., 8920.

6173, 18000., 4880., 8920.

6174, 18000., 5440., 8920.

6175, 18000., 3760., 9000.

6176, 18000., 4320., 9000.

6177, 18000., 4880., 9000.

6178, 18000., 5440., 9000.

6179, 18200., 3666.66675, 5700.

6180, 18200., 4133.3335, 5700.

6181, 18200., 4600., 5700.

6182, 18200., 5066.6665, 5700.

6183, 18200., 5533.3335, 5700.

6184, 18200., 5533.3335, 5920.

6185, 18200., 5066.6665, 5920.

6186, 18200., 4600., 5920.

6187, 18200., 4133.3335, 5920.

6188, 18200., 3666.66675, 5920.

6189, 18200., 5533.3335, 6000.

6190, 18200., 5066.6665, 6000.

6191, 18200., 4600., 6000.

6192, 18200., 4133.3335, 6000.

6193, 18200., 3666.66675, 6000.

6194, 18000., 5533.3335, 5700.

6195, 18000., 5066.6665, 5700.

6196, 18000., 4600., 5700.

6197, 18000., 4133.3335, 5700.

6198, 18000., 3666.66675, 5700.

6199, 18000., 5533.3335, 2700.

6200, 18000., 5066.6665, 2700.

6201, 18000., 4600., 2700.

6202, 18000., 4133.3335, 2700.

6203, 18000., 3666.66675, 2700.

6204, 18200., 3666.66675, 2700.

6205, 18200., 4133.3335, 2700.

6206, 18200., 4600., 2700.

6207, 18200., 5066.6665, 2700.

6208, 18200., 5533.3335, 2700.

6209, 18200., 8533.33301, 2700.

6210, 18200., 8066.6665, 2700.

6211, 18200., 7600., 2700.

6212, 18200., 7133.3335, 2700.

6213, 18200., 6666.6665, 2700.

6214, 18000., 6666.6665, 2700.

6215, 18000., 7133.3335, 2700.

6216, 18000., 7600., 2700.

6217, 18000., 8066.6665, 2700.

6218, 18000., 8533.33301, 2700.

6219, 18200., 6666.6665, 5920.

6220, 18200., 7133.3335, 5920.

6221, 18200., 7600., 5920.

6222, 18200., 8066.6665, 5920.

6223, 18200., 8533.33301, 5920.

6224, 18200., 8533.33301, 6000.

6225, 18200., 8066.6665, 6000.

6226, 18200., 7600., 6000.

6227, 18200., 7133.3335, 6000.

6228, 18200., 6666.6665, 6000.

6229, 18200., 6666.6665, 5700.

6230, 18200., 7133.3335, 5700.

6231, 18200., 7600., 5700.

6232, 18200., 8066.6665, 5700.

6233, 18200., 8533.33301, 5700.

6234, 18000., 8533.33301, 5700.

6235, 18000., 8066.6665, 5700.

6236, 18000., 7600., 5700.

6237, 18000., 7133.3335, 5700.

6238, 18000., 6666.6665, 5700.

6239, 18200., 6000., 8160.

6240, 18200., 6000., 7620.

6241, 18200., 6000., 7080.

6242, 18200., 6000., 6540.

6243, 18200., 6200., 6540.

6244, 18200., 6200., 7080.

6245, 18200., 6200., 7620.

6246, 18200., 6200., 8160.

6247, 18200., 6000., 5160.

6248, 18200., 6000., 4620.

6249, 18200., 6000., 4080.

6250, 18200., 6000., 3540.

6251, 18200., 6200., 3540.

6252, 18200., 6200., 4080.

6253, 18200., 6200., 4620.

6254, 18200., 6200., 5160.

6255, 18200., 6000., 2160.

6256, 18200., 6000., 1620.

6257, 18200., 6000., 1080.

6258, 18200., 6000., 540.

6259, 18200., 6200., 540.

6260, 18200., 6200., 1080.

6261, 18200., 6200., 1620.

6262, 18200., 6200., 2160.

6263, 18000., 6200., 8160.

6264, 18000., 6200., 7620.

6265, 18000., 6200., 7080.

6266, 18000., 6200., 6540.

6267, 18000., 6000., 6540.

6268, 18000., 6000., 7080.

6269, 18000., 6000., 7620.

6270, 18000., 6000., 8160.

6271, 18000., 6200., 5160.

6272, 18000., 6200., 4620.

6273, 18000., 6200., 4080.

6274, 18000., 6200., 3540.

6275, 18000., 6000., 3540.

6276, 18000., 6000., 4080.

6277, 18000., 6000., 4620.

6278, 18000., 6000., 5160.

6279, 18000., 6200., 2160.

6280, 18000., 6200., 1620.

6281, 18000., 6200., 1080.

6282, 18000., 6200., 540.

6283, 18000., 6000., 540.

6284, 18000., 6000., 1080.

6285, 18000., 6000., 1620.

6286, 18000., 6000., 2160.

6287, 18000., 9666.66699, 2700.

6288, 18000., 10133.333, 2700.

6289, 18000., 10600., 2700.

6290, 18000., 11066.667, 2700.

6291, 18000., 11533.333, 2700.

6292, 18200., 11533.333, 2700.

6293, 18200., 11066.667, 2700.

6294, 18200., 10600., 2700.

6295, 18200., 10133.333, 2700.

6296, 18200., 9666.66699, 2700.

6297, 18000., 11533.333, 5700.

6298, 18000., 11066.667, 5700.

6299, 18000., 10600., 5700.

6300, 18000., 10133.333, 5700.

6301, 18000., 9666.66699, 5700.

6302, 18200., 11533.333, 6000.

6303, 18200., 11066.667, 6000.

6304, 18200., 10600., 6000.

6305, 18200., 10133.333, 6000.

6306, 18200., 9666.66699, 6000.

6307, 18200., 9666.66699, 5920.

6308, 18200., 10133.333, 5920.

6309, 18200., 10600., 5920.

6310, 18200., 11066.667, 5920.

6311, 18200., 11533.333, 5920.

6312, 18200., 9666.66699, 5700.

6313, 18200., 10133.333, 5700.

6314, 18200., 10600., 5700.

6315, 18200., 11066.667, 5700.

6316, 18200., 11533.333, 5700.

6317, 18200., 9000., 2160.

6318, 18200., 9000., 1620.

6319, 18200., 9000., 1080.

6320, 18200., 9000., 540.

6321, 18200., 9200., 540.

6322, 18200., 9200., 1080.

6323, 18200., 9200., 1620.

6324, 18200., 9200., 2160.

6325, 18200., 9000., 5160.

6326, 18200., 9000., 4620.

6327, 18200., 9000., 4080.

6328, 18200., 9000., 3540.

6329, 18200., 9200., 3540.

6330, 18200., 9200., 4080.

6331, 18200., 9200., 4620.

6332, 18200., 9200., 5160.

6333, 18200., 9000., 8160.

6334, 18200., 9000., 7620.

6335, 18200., 9000., 7080.

6336, 18200., 9000., 6540.

6337, 18200., 9200., 6540.

6338, 18200., 9200., 7080.

6339, 18200., 9200., 7620.

6340, 18200., 9200., 8160.

6341, 18000., 9000., 540.

6342, 18000., 9000., 1080.

6343, 18000., 9000., 1620.

6344, 18000., 9000., 2160.

6345, 18000., 9200., 2160.

6346, 18000., 9200., 1620.

6347, 18000., 9200., 1080.

6348, 18000., 9200., 540.

6349, 18000., 9200., 5160.

6350, 18000., 9200., 4620.

6351, 18000., 9200., 4080.

6352, 18000., 9200., 3540.

6353, 18000., 9000., 3540.

6354, 18000., 9000., 4080.

6355, 18000., 9000., 4620.

6356, 18000., 9000., 5160.

6357, 18000., 9200., 8160.

6358, 18000., 9200., 7620.

6359, 18000., 9200., 7080.

6360, 18000., 9200., 6540.

6361, 18000., 9000., 6540.

6362, 18000., 9000., 7080.

6363, 18000., 9000., 7620.

6364, 18000., 9000., 8160.

6365, 18000., 12666.667, 9000.

6366, 18000., 13133.333, 9000.

6367, 18000., 13600., 9000.

6368, 18000., 14066.667, 9000.

6369, 18000., 14533.333, 9000.

6370, 18000., 14533.333, 8920.

6371, 18000., 14066.667, 8920.

6372, 18000., 13600., 8920.

6373, 18000., 13133.333, 8920.

6374, 18000., 12666.667, 8920.

6375, 18000., 14533.333, 8700.

6376, 18000., 14066.667, 8700.

6377, 18000., 13600., 8700.

6378, 18000., 13133.333, 8700.

6379, 18000., 12666.667, 8700.

6380, 18200., 14533.333, 9000.

6381, 18200., 14066.667, 9000.

6382, 18200., 13600., 9000.

6383, 18200., 13133.333, 9000.

6384, 18200., 12666.667, 9000.

6385, 18200., 12666.667, 8920.

6386, 18200., 13133.333, 8920.

6387, 18200., 13600., 8920.

6388, 18200., 14066.667, 8920.

6389, 18200., 14533.333, 8920.

6390, 18200., 12666.667, 8700.

6391, 18200., 13133.333, 8700.

6392, 18200., 13600., 8700.

6393, 18200., 14066.667, 8700.

6394, 18200., 14533.333, 8700.

6395, 18000., 14533.333, 5700.

6396, 18000., 14066.667, 5700.

6397, 18000., 13600., 5700.

6398, 18000., 13133.333, 5700.

6399, 18000., 12666.667, 5700.

6400, 18200., 14533.333, 6000.

6401, 18200., 14066.667, 6000.

6402, 18200., 13600., 6000.

6403, 18200., 13133.333, 6000.

6404, 18200., 12666.667, 6000.

6405, 18200., 12666.667, 5920.

6406, 18200., 13133.333, 5920.

6407, 18200., 13600., 5920.

6408, 18200., 14066.667, 5920.

6409, 18200., 14533.333, 5920.

6410, 18200., 12666.667, 5700.

6411, 18200., 13133.333, 5700.

6412, 18200., 13600., 5700.

6413, 18200., 14066.667, 5700.

6414, 18200., 14533.333, 5700.

6415, 18200., 12666.667, 2700.

6416, 18200., 13133.333, 2700.

6417, 18200., 13600., 2700.

6418, 18200., 14066.667, 2700.

6419, 18200., 14533.333, 2700.

6420, 18000., 14533.333, 2700.

6421, 18000., 14066.667, 2700.

6422, 18000., 13600., 2700.

6423, 18000., 13133.333, 2700.

6424, 18000., 12666.667, 2700.

6425, 18200., 666.666687, 8700.

6426, 18200., 1133.33337, 8700.

6427, 18200., 1600., 8700.

6428, 18200., 2066.66675, 8700.

6429, 18200., 2533.33325, 8700.

6430, 18200., 2533.33325, 8920.

6431, 18200., 2066.66675, 8920.

6432, 18200., 1600., 8920.

6433, 18200., 1133.33337, 8920.

6434, 18200., 666.666687, 8920.

6435, 18200., 2533.33325, 9000.

6436, 18200., 2066.66675, 9000.

6437, 18200., 1600., 9000.

6438, 18200., 1133.33337, 9000.

6439, 18200., 666.666687, 9000.

6440, 18000., 2533.33325, 8700.

6441, 18000., 2066.66675, 8700.

6442, 18000., 1600., 8700.

6443, 18000., 1133.33337, 8700.

6444, 18000., 666.666687, 8700.

6445, 18000., 666.666687, 8920.

6446, 18000., 1133.33337, 8920.

6447, 18000., 1600., 8920.

6448, 18000., 2066.66675, 8920.

6449, 18000., 2533.33325, 8920.

6450, 18000., 666.666687, 9000.

6451, 18000., 1133.33337, 9000.

6452, 18000., 1600., 9000.

6453, 18000., 2066.66675, 9000.

6454, 18000., 2533.33325, 9000.

6455, 18200., 6666.6665, 8920.

6456, 18200., 7133.3335, 8920.

6457, 18200., 7600., 8920.

6458, 18200., 8066.6665, 8920.

6459, 18200., 8533.33301, 8920.

6460, 18200., 8533.33301, 9000.

6461, 18200., 8066.6665, 9000.

6462, 18200., 7600., 9000.

6463, 18200., 7133.3335, 9000.

6464, 18200., 6666.6665, 9000.

6465, 18200., 6666.6665, 8700.

6466, 18200., 7133.3335, 8700.

6467, 18200., 7600., 8700.

6468, 18200., 8066.6665, 8700.

6469, 18200., 8533.33301, 8700.

6470, 18000., 8533.33301, 8920.

6471, 18000., 8066.6665, 8920.

6472, 18000., 7600., 8920.

6473, 18000., 7133.3335, 8920.

6474, 18000., 6666.6665, 8920.

6475, 18000., 6666.6665, 9000.

6476, 18000., 7133.3335, 9000.

6477, 18000., 7600., 9000.

6478, 18000., 8066.6665, 9000.

6479, 18000., 8533.33301, 9000.

6480, 18000., 8533.33301, 8700.

6481, 18000., 8066.6665, 8700.

6482, 18000., 7600., 8700.

6483, 18000., 7133.3335, 8700.

6484, 18000., 6666.6665, 8700.

6485, 18000., 11533.333, 8700.

6486, 18000., 11066.667, 8700.

6487, 18000., 10600., 8700.

6488, 18000., 10133.333, 8700.

6489, 18000., 9666.66699, 8700.

6490, 18000., 9666.66699, 8920.

6491, 18000., 10133.333, 8920.

6492, 18000., 10600., 8920.

6493, 18000., 11066.667, 8920.

6494, 18000., 11533.333, 8920.

6495, 18000., 9666.66699, 9000.

6496, 18000., 10133.333, 9000.

6497, 18000., 10600., 9000.

6498, 18000., 11066.667, 9000.

6499, 18000., 11533.333, 9000.

6500, 18200., 9666.66699, 8700.

6501, 18200., 10133.333, 8700.

6502, 18200., 10600., 8700.

6503, 18200., 11066.667, 8700.

6504, 18200., 11533.333, 8700.

6505, 18200., 11533.333, 8920.

6506, 18200., 11066.667, 8920.

6507, 18200., 10600., 8920.

6508, 18200., 10133.333, 8920.

6509, 18200., 9666.66699, 8920.

6510, 18200., 11533.333, 9000.

6511, 18200., 11066.667, 9000.

6512, 18200., 10600., 9000.

6513, 18200., 10133.333, 9000.

6514, 18200., 9666.66699, 9000.

6515, 18200., 12000., 2160.

6516, 18200., 12000., 1620.

6517, 18200., 12000., 1080.

6518, 18200., 12000., 540.

6519, 18200., 12200., 540.

6520, 18200., 12200., 1080.

6521, 18200., 12200., 1620.

6522, 18200., 12200., 2160.

6523, 18200., 12000., 5160.

6524, 18200., 12000., 4620.

6525, 18200., 12000., 4080.

6526, 18200., 12000., 3540.

6527, 18200., 12200., 3540.

6528, 18200., 12200., 4080.

6529, 18200., 12200., 4620.

6530, 18200., 12200., 5160.

6531, 18200., 12000., 8160.

6532, 18200., 12000., 7620.

6533, 18200., 12000., 7080.

6534, 18200., 12000., 6540.

6535, 18200., 12200., 6540.

6536, 18200., 12200., 7080.

6537, 18200., 12200., 7620.

6538, 18200., 12200., 8160.

6539, 18000., 12000., 540.

6540, 18000., 12000., 1080.

6541, 18000., 12000., 1620.

6542, 18000., 12000., 2160.

6543, 18000., 12200., 2160.

6544, 18000., 12200., 1620.

6545, 18000., 12200., 1080.

6546, 18000., 12200., 540.

6547, 18000., 12200., 5160.

6548, 18000., 12200., 4620.

6549, 18000., 12200., 4080.

6550, 18000., 12200., 3540.

6551, 18000., 12000., 3540.

6552, 18000., 12000., 4080.

6553, 18000., 12000., 4620.

6554, 18000., 12000., 5160.

6555, 18000., 12200., 8160.

6556, 18000., 12200., 7620.

6557, 18000., 12200., 7080.

6558, 18000., 12200., 6540.

6559, 18000., 12000., 6540.

6560, 18000., 12000., 7080.

6561, 18000., 12000., 7620.

6562, 18000., 12000., 8160.

6563, 18200., 15000., 540.

6564, 18200., 15000., 1080.

6565, 18200., 15000., 1620.

6566, 18200., 15000., 2160.

6567, 18000., 15000., 2160.

6568, 18000., 15000., 1620.

6569, 18000., 15000., 1080.

6570, 18000., 15000., 540.

6571, 18000., 15000., 5160.

6572, 18000., 15000., 4620.

6573, 18000., 15000., 4080.

6574, 18000., 15000., 3540.

6575, 18200., 15000., 3540.

6576, 18200., 15000., 4080.

6577, 18200., 15000., 4620.

6578, 18200., 15000., 5160.

6579, 18000., 15000., 8160.

6580, 18000., 15000., 7620.

6581, 18000., 15000., 7080.

6582, 18000., 15000., 6540.

6583, 18200., 15000., 6540.

6584, 18200., 15000., 7080.

6585, 18200., 15000., 7620.

6586, 18200., 15000., 8160.

6587, 18200., 15200., 2160.

6588, 18200., 15200., 1620.

6589, 18200., 15200., 1080.

6590, 18200., 15200., 540.

6591, 18200., 15200., 5160.

6592, 18200., 15200., 4620.

6593, 18200., 15200., 4080.

6594, 18200., 15200., 3540.

6595, 18200., 15200., 8160.

6596, 18200., 15200., 7620.

6597, 18200., 15200., 7080.

6598, 18200., 15200., 6540.

6599, 18000., 15200., 540.

6600, 18000., 15200., 1080.

6601, 18000., 15200., 1620.

6602, 18000., 15200., 2160.

6603, 18000., 15200., 3540.

6604, 18000., 15200., 4080.

6605, 18000., 15200., 4620.

6606, 18000., 15200., 5160.

6607, 18000., 15200., 6540.

6608, 18000., 15200., 7080.

6609, 18000., 15200., 7620.

6610, 18000., 15200., 8160.

6611, 15200., 666.666687, 2700.

6612, 15200., 1133.33337, 2700.

6613, 15200., 1600., 2700.

6614, 15200., 2066.66675, 2700.

6615, 15200., 2533.33325, 2700.

6616, 15000., 2533.33325, 2700.

6617, 15000., 2066.66675, 2700.

6618, 15000., 1600., 2700.

6619, 15000., 1133.33337, 2700.

6620, 15000., 666.666687, 2700.

6621, 15200., 2533.33325, 5700.

6622, 15200., 2066.66675, 5700.

6623, 15200., 1600., 5700.

6624, 15200., 1133.33337, 5700.

6625, 15200., 666.666687, 5700.

6626, 15000., 666.666687, 5700.

6627, 15000., 1133.33337, 5700.

6628, 15000., 1600., 5700.

6629, 15000., 2066.66675, 5700.

6630, 15000., 2533.33325, 5700.

6631, 15000., 3000., 8850.

6632, 15000., 3000., 8160.

6633, 15000., 3000., 7620.

6634, 15000., 3000., 7080.

6635, 15000., 3000., 6540.

6636, 15200., 3000., 6540.

6637, 15200., 3000., 7080.

6638, 15200., 3000., 7620.

6639, 15200., 3000., 8160.

6640, 15000., 3000., 5160.

6641, 15000., 3000., 4620.

6642, 15000., 3000., 4080.

6643, 15000., 3000., 3540.

6644, 15200., 3000., 3540.

6645, 15200., 3000., 4080.

6646, 15200., 3000., 4620.

6647, 15200., 3000., 5160.

6648, 15000., 3000., 2160.

6649, 15000., 3000., 1620.

6650, 15000., 3000., 1080.

6651, 15000., 3000., 540.

6652, 15200., 3000., 540.

6653, 15200., 3000., 1080.

6654, 15200., 3000., 1620.

6655, 15200., 3000., 2160.

6656, 15000., 3200., 8850.

6657, 15200., 3200., 8160.

6658, 15200., 3200., 7620.

6659, 15200., 3200., 7080.

6660, 15200., 3200., 6540.

6661, 15000., 3200., 6540.

6662, 15000., 3200., 7080.

6663, 15000., 3200., 7620.

6664, 15000., 3200., 8160.

6665, 15200., 3200., 5160.

6666, 15200., 3200., 4620.

6667, 15200., 3200., 4080.

6668, 15200., 3200., 3540.

6669, 15000., 3200., 3540.

6670, 15000., 3200., 4080.

6671, 15000., 3200., 4620.

6672, 15000., 3200., 5160.

6673, 15200., 3200., 2160.

6674, 15200., 3200., 1620.

6675, 15200., 3200., 1080.

6676, 15200., 3200., 540.

6677, 15000., 3200., 540.

6678, 15000., 3200., 1080.

6679, 15000., 3200., 1620.

6680, 15000., 3200., 2160.

6681, 15200., 5440., 9000.

6682, 15200., 4880., 9000.

6683, 15200., 4320., 9000.

6684, 15200., 3760., 9000.

6685, 15200., 3760., 8920.

6686, 15200., 4320., 8920.

6687, 15200., 4880., 8920.

6688, 15200., 5440., 8920.

6689, 15200., 3760., 8700.

6690, 15200., 4320., 8700.

6691, 15200., 4880., 8700.

6692, 15200., 5440., 8700.

6693, 15000., 3760., 9000.

6694, 15000., 4320., 9000.

6695, 15000., 4880., 9000.

6696, 15000., 5440., 9000.

6697, 15000., 5440., 8700.

6698, 15000., 4880., 8700.

6699, 15000., 4320., 8700.

6700, 15000., 3760., 8700.

6701, 15200., 3666.66675, 5700.

6702, 15200., 4133.3335, 5700.

6703, 15200., 4600., 5700.

6704, 15200., 5066.6665, 5700.

6705, 15200., 5533.3335, 5700.

6706, 15000., 5533.3335, 5700.

6707, 15000., 5066.6665, 5700.

6708, 15000., 4600., 5700.

6709, 15000., 4133.3335, 5700.

6710, 15000., 3666.66675, 5700.

6711, 15000., 5533.3335, 2700.

6712, 15000., 5066.6665, 2700.

6713, 15000., 4600., 2700.

6714, 15000., 4133.3335, 2700.

6715, 15000., 3666.66675, 2700.

6716, 15200., 3666.66675, 2700.

6717, 15200., 4133.3335, 2700.

6718, 15200., 4600., 2700.

6719, 15200., 5066.6665, 2700.

6720, 15200., 5533.3335, 2700.

6721, 15000., 8533.33301, 8700.

6722, 15000., 8066.6665, 8700.

6723, 15000., 7600., 8700.

6724, 15000., 7133.3335, 8700.

6725, 15000., 6666.6665, 8700.

6726, 15200., 6666.6665, 8700.

6727, 15200., 7133.3335, 8700.

6728, 15200., 7600., 8700.

6729, 15200., 8066.6665, 8700.

6730, 15200., 8533.33301, 8700.

6731, 15200., 8533.33301, 8920.

6732, 15200., 8066.6665, 8920.

6733, 15200., 7600., 8920.

6734, 15200., 7133.3335, 8920.

6735, 15200., 6666.6665, 8920.

6736, 15200., 8533.33301, 9000.

6737, 15200., 8066.6665, 9000.

6738, 15200., 7600., 9000.

6739, 15200., 7133.3335, 9000.

6740, 15200., 6666.6665, 9000.

6741, 15000., 8533.33301, 5700.

6742, 15000., 8066.6665, 5700.

6743, 15000., 7600., 5700.

6744, 15000., 7133.3335, 5700.

6745, 15000., 6666.6665, 5700.

6746, 15200., 6666.6665, 5700.

6747, 15200., 7133.3335, 5700.

6748, 15200., 7600., 5700.

6749, 15200., 8066.6665, 5700.

6750, 15200., 8533.33301, 5700.

6751, 15200., 6666.6665, 2700.

6752, 15200., 7133.3335, 2700.

6753, 15200., 7600., 2700.

6754, 15200., 8066.6665, 2700.

6755, 15200., 8533.33301, 2700.

6756, 15000., 8533.33301, 2700.

6757, 15000., 8066.6665, 2700.

6758, 15000., 7600., 2700.

6759, 15000., 7133.3335, 2700.

6760, 15000., 6666.6665, 2700.

6761, 15000., 11533.333, 2700.

6762, 15000., 11066.667, 2700.

6763, 15000., 10600., 2700.

6764, 15000., 10133.333, 2700.

6765, 15000., 9666.66699, 2700.

6766, 15200., 9666.66699, 2700.

6767, 15200., 10133.333, 2700.

6768, 15200., 10600., 2700.

6769, 15200., 11066.667, 2700.

6770, 15200., 11533.333, 2700.

6771, 15200., 11533.333, 5700.

6772, 15200., 11066.667, 5700.

6773, 15200., 10600., 5700.

6774, 15200., 10133.333, 5700.

6775, 15200., 9666.66699, 5700.

6776, 15000., 9666.66699, 5700.

6777, 15000., 10133.333, 5700.

6778, 15000., 10600., 5700.

6779, 15000., 11066.667, 5700.

6780, 15000., 11533.333, 5700.

6781, 15200., 12000., 8160.

6782, 15200., 12000., 7620.

6783, 15200., 12000., 7080.

6784, 15200., 12000., 6540.

6785, 15200., 12200., 6540.

6786, 15200., 12200., 7080.

6787, 15200., 12200., 7620.

6788, 15200., 12200., 8160.

6789, 15200., 12000., 5160.

6790, 15200., 12000., 4620.

6791, 15200., 12000., 4080.

6792, 15200., 12000., 3540.

6793, 15200., 12200., 3540.

6794, 15200., 12200., 4080.

6795, 15200., 12200., 4620.

6796, 15200., 12200., 5160.

6797, 15200., 12000., 2160.

6798, 15200., 12000., 1620.

6799, 15200., 12000., 1080.

6800, 15200., 12000., 540.

6801, 15200., 12200., 540.

6802, 15200., 12200., 1080.

6803, 15200., 12200., 1620.

6804, 15200., 12200., 2160.

6805, 15000., 12200., 8160.

6806, 15000., 12200., 7620.

6807, 15000., 12200., 7080.

6808, 15000., 12200., 6540.

6809, 15000., 12000., 6540.

6810, 15000., 12000., 7080.

6811, 15000., 12000., 7620.

6812, 15000., 12000., 8160.

6813, 15000., 12200., 5160.

6814, 15000., 12200., 4620.

6815, 15000., 12200., 4080.

6816, 15000., 12200., 3540.

6817, 15000., 12000., 3540.

6818, 15000., 12000., 4080.

6819, 15000., 12000., 4620.

6820, 15000., 12000., 5160.

6821, 15000., 12200., 2160.

6822, 15000., 12200., 1620.

6823, 15000., 12200., 1080.

6824, 15000., 12200., 540.

6825, 15000., 12000., 540.

6826, 15000., 12000., 1080.

6827, 15000., 12000., 1620.

6828, 15000., 12000., 2160.

6829, 15000., 14533.333, 2700.

6830, 15000., 14066.667, 2700.

6831, 15000., 13600., 2700.

6832, 15000., 13133.333, 2700.

6833, 15000., 12666.667, 2700.

6834, 15200., 12666.667, 2700.

6835, 15200., 13133.333, 2700.

6836, 15200., 13600., 2700.

6837, 15200., 14066.667, 2700.

6838, 15200., 14533.333, 2700.

6839, 15000., 15000., 8160.

6840, 15000., 15000., 7620.

6841, 15000., 15000., 7080.

6842, 15000., 15000., 6540.

6843, 15200., 15000., 6540.

6844, 15200., 15000., 7080.

6845, 15200., 15000., 7620.

6846, 15200., 15000., 8160.

6847, 15000., 15000., 5160.

6848, 15000., 15000., 4620.

6849, 15000., 15000., 4080.

6850, 15000., 15000., 3540.

6851, 15200., 15000., 3540.

6852, 15200., 15000., 4080.

6853, 15200., 15000., 4620.

6854, 15200., 15000., 5160.

6855, 15000., 15000., 2160.

6856, 15000., 15000., 1620.

6857, 15000., 15000., 1080.

6858, 15000., 15000., 540.

6859, 15200., 15000., 540.

6860, 15200., 15000., 1080.

6861, 15200., 15000., 1620.

6862, 15200., 15000., 2160.

6863, 15000., 15200., 6540.

6864, 15000., 15200., 7080.

6865, 15000., 15200., 7620.

6866, 15000., 15200., 8160.

6867, 15000., 15200., 3540.

6868, 15000., 15200., 4080.

6869, 15000., 15200., 4620.

6870, 15000., 15200., 5160.

6871, 15000., 15200., 540.

6872, 15000., 15200., 1080.

6873, 15000., 15200., 1620.

6874, 15000., 15200., 2160.

6875, 15200., 15200., 8160.

6876, 15200., 15200., 7620.

6877, 15200., 15200., 7080.

6878, 15200., 15200., 6540.

6879, 15200., 15200., 5160.

6880, 15200., 15200., 4620.

6881, 15200., 15200., 4080.

6882, 15200., 15200., 3540.

6883, 15200., 15200., 2160.

6884, 15200., 15200., 1620.

6885, 15200., 15200., 1080.

6886, 15200., 15200., 540.

6887, 15000., 14533.333, 5700.

6888, 15000., 14066.667, 5700.

6889, 15000., 13600., 5700.

6890, 15000., 13133.333, 5700.

6891, 15000., 12666.667, 5700.

6892, 15200., 12666.667, 5700.

6893, 15200., 13133.333, 5700.

6894, 15200., 13600., 5700.

6895, 15200., 14066.667, 5700.

6896, 15200., 14533.333, 5700.

6897, 15000., 200., 540.

6898, 15000., 200., 1080.

6899, 15000., 200., 1620.

6900, 15000., 200., 2160.

6901, 15200., 200., 2160.

6902, 15200., 200., 1620.

6903, 15200., 200., 1080.

6904, 15200., 200., 540.

6905, 15200., 200., 5160.

6906, 15200., 200., 4620.

6907, 15200., 200., 4080.

6908, 15200., 200., 3540.

6909, 15000., 200., 3540.

6910, 15000., 200., 4080.

6911, 15000., 200., 4620.

6912, 15000., 200., 5160.

6913, 15200., 200., 8160.

6914, 15200., 200., 7620.

6915, 15200., 200., 7080.

6916, 15200., 200., 6540.

6917, 15000., 200., 6540.

6918, 15000., 200., 7080.

6919, 15000., 200., 7620.

6920, 15000., 200., 8160.

6921, 15000., 200., 8850.

6922, 15000., 0., 2160.

6923, 15000., 0., 1620.

6924, 15000., 0., 1080.

6925, 15000., 0., 540.

6926, 15000., 0., 5160.

6927, 15000., 0., 4620.

6928, 15000., 0., 4080.

6929, 15000., 0., 3540.

6930, 15000., 0., 8571.42871

6931, 15000., 0., 8142.85693

6932, 15000., 0., 7714.28564

6933, 15000., 0., 7285.71436

6934, 15000., 0., 6857.14307

6935, 15000., 0., 6428.57129

6936, 15200., 0., 540.

6937, 15200., 0., 1080.

6938, 15200., 0., 1620.

6939, 15200., 0., 2160.

6940, 15200., 0., 3540.

6941, 15200., 0., 4080.

6942, 15200., 0., 4620.

6943, 15200., 0., 5160.

6944, 15200., 0., 6540.

6945, 15200., 0., 7080.

6946, 15200., 0., 7620.

6947, 15200., 0., 8160.

6948, 15000., 666.666687, 8700.

6949, 15000., 1133.33337, 8700.

6950, 15000., 1600., 8700.

6951, 15000., 2066.66675, 8700.

6952, 15000., 2533.33325, 8700.

6953, 15200., 2533.33325, 8700.

6954, 15200., 2066.66675, 8700.

6955, 15200., 1600., 8700.

6956, 15200., 1133.33337, 8700.

6957, 15200., 666.666687, 8700.

6958, 15000., 2533.33325, 9000.

6959, 15000., 2066.66675, 9000.

6960, 15000., 1600., 9000.

6961, 15000., 1133.33337, 9000.

6962, 15000., 666.666687, 9000.

6963, 15200., 666.666687, 9000.

6964, 15200., 1133.33337, 9000.

6965, 15200., 1600., 9000.

6966, 15200., 2066.66675, 9000.

6967, 15200., 2533.33325, 9000.

6968, 15200., 2533.33325, 8920.

6969, 15200., 2066.66675, 8920.

6970, 15200., 1600., 8920.

6971, 15200., 1133.33337, 8920.

6972, 15200., 666.666687, 8920.

6973, 15000., 6200., 8160.

6974, 15000., 6200., 7620.

6975, 15000., 6200., 7080.

6976, 15000., 6200., 6540.

6977, 15000., 6000., 6540.

6978, 15000., 6000., 7080.

6979, 15000., 6000., 7620.

6980, 15000., 6000., 8160.

6981, 15000., 6200., 5160.

6982, 15000., 6200., 4620.

6983, 15000., 6200., 4080.

6984, 15000., 6200., 3540.

6985, 15000., 6000., 3540.

6986, 15000., 6000., 4080.

6987, 15000., 6000., 4620.

6988, 15000., 6000., 5160.

6989, 15000., 6200., 2160.

6990, 15000., 6200., 1620.

6991, 15000., 6200., 1080.

6992, 15000., 6200., 540.

6993, 15000., 6000., 540.

6994, 15000., 6000., 1080.

6995, 15000., 6000., 1620.

6996, 15000., 6000., 2160.

6997, 15200., 6000., 8160.

6998, 15200., 6000., 7620.

6999, 15200., 6000., 7080.

7000, 15200., 6000., 6540.

7001, 15200., 6200., 6540.

7002, 15200., 6200., 7080.

7003, 15200., 6200., 7620.

7004, 15200., 6200., 8160.

7005, 15200., 6000., 5160.

7006, 15200., 6000., 4620.

7007, 15200., 6000., 4080.

7008, 15200., 6000., 3540.

7009, 15200., 6200., 3540.

7010, 15200., 6200., 4080.

7011, 15200., 6200., 4620.

7012, 15200., 6200., 5160.

7013, 15200., 6200., 540.

7014, 15200., 6200., 1080.

7015, 15200., 6200., 1620.

7016, 15200., 6200., 2160.

7017, 15200., 6000., 2160.

7018, 15200., 6000., 1620.

7019, 15200., 6000., 1080.

7020, 15200., 6000., 540.

7021, 15000., 11533.333, 8700.

7022, 15000., 11066.667, 8700.

7023, 15000., 10600., 8700.

7024, 15000., 10133.333, 8700.

7025, 15000., 9666.66699, 8700.

7026, 15200., 9666.66699, 8920.

7027, 15200., 10133.333, 8920.

7028, 15200., 10600., 8920.

7029, 15200., 11066.667, 8920.

7030, 15200., 11533.333, 8920.

7031, 15200., 11533.333, 9000.

7032, 15200., 11066.667, 9000.

7033, 15200., 10600., 9000.

7034, 15200., 10133.333, 9000.

7035, 15200., 9666.66699, 9000.

7036, 15200., 9666.66699, 8700.

7037, 15200., 10133.333, 8700.

7038, 15200., 10600., 8700.

7039, 15200., 11066.667, 8700.

7040, 15200., 11533.333, 8700.

7041, 15200., 14533.333, 9000.

7042, 15200., 14066.667, 9000.

7043, 15200., 13600., 9000.

7044, 15200., 13133.333, 9000.

7045, 15200., 12666.667, 9000.

7046, 15200., 12666.667, 8920.

7047, 15200., 13133.333, 8920.

7048, 15200., 13600., 8920.

7049, 15200., 14066.667, 8920.

7050, 15200., 14533.333, 8920.

7051, 15200., 12666.667, 8700.

7052, 15200., 13133.333, 8700.

7053, 15200., 13600., 8700.

7054, 15200., 14066.667, 8700.

7055, 15200., 14533.333, 8700.

7056, 15000., 14533.333, 8700.

7057, 15000., 14066.667, 8700.

7058, 15000., 13600., 8700.

7059, 15000., 13133.333, 8700.

7060, 15000., 12666.667, 8700.

7061, 12200., 666.666687, 2700.

7062, 12200., 1133.33337, 2700.

7063, 12200., 1600., 2700.

7064, 12200., 2066.66675, 2700.

7065, 12200., 2533.33325, 2700.

7066, 12000., 2533.33325, 2700.

7067, 12000., 2066.66675, 2700.

7068, 12000., 1600., 2700.

7069, 12000., 1133.33337, 2700.

7070, 12000., 666.666687, 2700.

7071, 12000., 666.666687, 2920.

7072, 12000., 1133.33337, 2920.

7073, 12000., 1600., 2920.

7074, 12000., 2066.66675, 2920.

7075, 12000., 2533.33325, 2920.

7076, 12000., 666.666687, 3000.

7077, 12000., 1133.33337, 3000.

7078, 12000., 1600., 3000.

7079, 12000., 2066.66675, 3000.

7080, 12000., 2533.33325, 3000.

7081, 12000., 200., 540.

7082, 12000., 200., 1080.

7083, 12000., 200., 1620.

7084, 12000., 200., 2160.

7085, 12200., 200., 2160.

7086, 12200., 200., 1620.

7087, 12200., 200., 1080.

7088, 12200., 200., 540.

7089, 12200., 200., 5160.

7090, 12200., 200., 4620.

7091, 12200., 200., 4080.

7092, 12200., 200., 3540.

7093, 12000., 200., 3540.

7094, 12000., 200., 4080.

7095, 12000., 200., 4620.

7096, 12000., 200., 5160.

7097, 12000., 0., 2160.

7098, 12000., 0., 1620.

7099, 12000., 0., 1080.

7100, 12000., 0., 540.

7101, 12000., 0., 5160.

7102, 12000., 0., 4620.

7103, 12000., 0., 4080.

7104, 12000., 0., 3540.

7105, 12200., 0., 540.

7106, 12200., 0., 1080.

7107, 12200., 0., 1620.

7108, 12200., 0., 2160.

7109, 12200., 0., 3540.

7110, 12200., 0., 4080.

7111, 12200., 0., 4620.

7112, 12200., 0., 5160.

7113, 12200., 3666.66675, 5700.

7114, 12200., 4133.3335, 5700.

7115, 12200., 4600., 5700.

7116, 12200., 5066.6665, 5700.

7117, 12200., 5533.3335, 5700.

7118, 12000., 5533.3335, 5700.

7119, 12000., 5066.6665, 5700.

7120, 12000., 4600., 5700.

7121, 12000., 4133.3335, 5700.

7122, 12000., 3666.66675, 5700.

7123, 12000., 3666.66675, 5920.

7124, 12000., 4133.3335, 5920.

7125, 12000., 4600., 5920.

7126, 12000., 5066.6665, 5920.

7127, 12000., 5533.3335, 5920.

7128, 12000., 3666.66675, 6000.

7129, 12000., 4133.3335, 6000.

7130, 12000., 4600., 6000.

7131, 12000., 5066.6665, 6000.

7132, 12000., 5533.3335, 6000.

7133, 12200., 3666.66675, 2700.

7134, 12200., 4133.3335, 2700.

7135, 12200., 4600., 2700.

7136, 12200., 5066.6665, 2700.

7137, 12200., 5533.3335, 2700.

7138, 12000., 5533.3335, 2920.

7139, 12000., 5066.6665, 2920.

7140, 12000., 4600., 2920.

7141, 12000., 4133.3335, 2920.

7142, 12000., 3666.66675, 2920.

7143, 12000., 3666.66675, 3000.

7144, 12000., 4133.3335, 3000.

7145, 12000., 4600., 3000.

7146, 12000., 5066.6665, 3000.

7147, 12000., 5533.3335, 3000.

7148, 12000., 5533.3335, 2700.

7149, 12000., 5066.6665, 2700.

7150, 12000., 4600., 2700.

7151, 12000., 4133.3335, 2700.

7152, 12000., 3666.66675, 2700.

7153, 12000., 6666.6665, 9000.

7154, 12000., 7133.3335, 9000.

7155, 12000., 7600., 9000.

7156, 12000., 8066.6665, 9000.

7157, 12000., 8533.33301, 9000.

7158, 12000., 8533.33301, 8920.

7159, 12000., 8066.6665, 8920.

7160, 12000., 7600., 8920.

7161, 12000., 7133.3335, 8920.

7162, 12000., 6666.6665, 8920.

7163, 12000., 8533.33301, 8700.

7164, 12000., 8066.6665, 8700.

7165, 12000., 7600., 8700.

7166, 12000., 7133.3335, 8700.

7167, 12000., 6666.6665, 8700.

7168, 12200., 6666.6665, 8700.

7169, 12200., 7133.3335, 8700.

7170, 12200., 7600., 8700.

7171, 12200., 8066.6665, 8700.

7172, 12200., 8533.33301, 8700.

7173, 12000., 6666.6665, 6000.

7174, 12000., 7133.3335, 6000.

7175, 12000., 7600., 6000.

7176, 12000., 8066.6665, 6000.

7177, 12000., 8533.33301, 6000.

7178, 12000., 8533.33301, 5920.

7179, 12000., 8066.6665, 5920.

7180, 12000., 7600., 5920.

7181, 12000., 7133.3335, 5920.

7182, 12000., 6666.6665, 5920.

7183, 12000., 8533.33301, 5700.

7184, 12000., 8066.6665, 5700.

7185, 12000., 7600., 5700.

7186, 12000., 7133.3335, 5700.

7187, 12000., 6666.6665, 5700.

7188, 12200., 6666.6665, 5700.

7189, 12200., 7133.3335, 5700.

7190, 12200., 7600., 5700.

7191, 12200., 8066.6665, 5700.

7192, 12200., 8533.33301, 5700.

7193, 12000., 6666.6665, 3000.

7194, 12000., 7133.3335, 3000.

7195, 12000., 7600., 3000.

7196, 12000., 8066.6665, 3000.

7197, 12000., 8533.33301, 3000.

7198, 12000., 8533.33301, 2920.

7199, 12000., 8066.6665, 2920.

7200, 12000., 7600., 2920.

7201, 12000., 7133.3335, 2920.

7202, 12000., 6666.6665, 2920.

7203, 12000., 8533.33301, 2700.

7204, 12000., 8066.6665, 2700.

7205, 12000., 7600., 2700.

7206, 12000., 7133.3335, 2700.

7207, 12000., 6666.6665, 2700.

7208, 12200., 6666.6665, 2700.

7209, 12200., 7133.3335, 2700.

7210, 12200., 7600., 2700.

7211, 12200., 8066.6665, 2700.

7212, 12200., 8533.33301, 2700.

7213, 12000., 9666.66699, 9000.

7214, 12000., 10133.333, 9000.

7215, 12000., 10600., 9000.

7216, 12000., 11066.667, 9000.

7217, 12000., 11533.333, 9000.

7218, 12000., 11533.333, 8920.

7219, 12000., 11066.667, 8920.

7220, 12000., 10600., 8920.

7221, 12000., 10133.333, 8920.

7222, 12000., 9666.66699, 8920.

7223, 12000., 11533.333, 8700.

7224, 12000., 11066.667, 8700.

7225, 12000., 10600., 8700.

7226, 12000., 10133.333, 8700.

7227, 12000., 9666.66699, 8700.

7228, 12200., 9666.66699, 8700.

7229, 12200., 10133.333, 8700.

7230, 12200., 10600., 8700.

7231, 12200., 11066.667, 8700.

7232, 12200., 11533.333, 8700.

7233, 12000., 9666.66699, 6000.

7234, 12000., 10133.333, 6000.

7235, 12000., 10600., 6000.

7236, 12000., 11066.667, 6000.

7237, 12000., 11533.333, 6000.

7238, 12000., 11533.333, 5920.

7239, 12000., 11066.667, 5920.

7240, 12000., 10600., 5920.

7241, 12000., 10133.333, 5920.

7242, 12000., 9666.66699, 5920.

7243, 12000., 11533.333, 5700.

7244, 12000., 11066.667, 5700.

7245, 12000., 10600., 5700.

7246, 12000., 10133.333, 5700.

7247, 12000., 9666.66699, 5700.

7248, 12200., 9666.66699, 5700.

7249, 12200., 10133.333, 5700.

7250, 12200., 10600., 5700.

7251, 12200., 11066.667, 5700.

7252, 12200., 11533.333, 5700.

7253, 12200., 9666.66699, 2700.

7254, 12200., 10133.333, 2700.

7255, 12200., 10600., 2700.

7256, 12200., 11066.667, 2700.

7257, 12200., 11533.333, 2700.

7258, 12000., 11533.333, 2920.

7259, 12000., 11066.667, 2920.

7260, 12000., 10600., 2920.

7261, 12000., 10133.333, 2920.

7262, 12000., 9666.66699, 2920.

7263, 12000., 9666.66699, 3000.

7264, 12000., 10133.333, 3000.

7265, 12000., 10600., 3000.

7266, 12000., 11066.667, 3000.

7267, 12000., 11533.333, 3000.

7268, 12000., 11533.333, 2700.

7269, 12000., 11066.667, 2700.

7270, 12000., 10600., 2700.

7271, 12000., 10133.333, 2700.

7272, 12000., 9666.66699, 2700.

7273, 12200., 12666.667, 8700.

7274, 12200., 13133.333, 8700.

7275, 12200., 13600., 8700.

7276, 12200., 14066.667, 8700.

7277, 12200., 14533.333, 8700.

7278, 12000., 14533.333, 8700.

7279, 12000., 14066.667, 8700.

7280, 12000., 13600., 8700.

7281, 12000., 13133.333, 8700.

7282, 12000., 12666.667, 8700.

7283, 12000., 12666.667, 8920.

7284, 12000., 13133.333, 8920.

7285, 12000., 13600., 8920.

7286, 12000., 14066.667, 8920.

7287, 12000., 14533.333, 8920.

7288, 12000., 12666.667, 9000.

7289, 12000., 13133.333, 9000.

7290, 12000., 13600., 9000.

7291, 12000., 14066.667, 9000.

7292, 12000., 14533.333, 9000.

7293, 12200., 12666.667, 5700.

7294, 12200., 13133.333, 5700.

7295, 12200., 13600., 5700.

7296, 12200., 14066.667, 5700.

7297, 12200., 14533.333, 5700.

7298, 12000., 14533.333, 5700.

7299, 12000., 14066.667, 5700.

7300, 12000., 13600., 5700.

7301, 12000., 13133.333, 5700.

7302, 12000., 12666.667, 5700.

7303, 12000., 12666.667, 5920.

7304, 12000., 13133.333, 5920.

7305, 12000., 13600., 5920.

7306, 12000., 14066.667, 5920.

7307, 12000., 14533.333, 5920.

7308, 12000., 12666.667, 6000.

7309, 12000., 13133.333, 6000.

7310, 12000., 13600., 6000.

7311, 12000., 14066.667, 6000.

7312, 12000., 14533.333, 6000.

7313, 12000., 12666.667, 3000.

7314, 12000., 13133.333, 3000.

7315, 12000., 13600., 3000.

7316, 12000., 14066.667, 3000.

7317, 12000., 14533.333, 3000.

7318, 12000., 14533.333, 2920.

7319, 12000., 14066.667, 2920.

7320, 12000., 13600., 2920.

7321, 12000., 13133.333, 2920.

7322, 12000., 12666.667, 2920.

7323, 12000., 14533.333, 2700.

7324, 12000., 14066.667, 2700.

7325, 12000., 13600., 2700.

7326, 12000., 13133.333, 2700.

7327, 12000., 12666.667, 2700.

7328, 12200., 12666.667, 2700.

7329, 12200., 13133.333, 2700.

7330, 12200., 13600., 2700.

7331, 12200., 14066.667, 2700.

7332, 12200., 14533.333, 2700.

7333, 12000., 3200., 5160.

7334, 12000., 3200., 4620.

7335, 12000., 3200., 4080.

7336, 12000., 3200., 3540.

7337, 12000., 3000., 3540.

7338, 12000., 3000., 4080.

7339, 12000., 3000., 4620.

7340, 12000., 3000., 5160.

7341, 12000., 3200., 2160.

7342, 12000., 3200., 1620.

7343, 12000., 3200., 1080.

7344, 12000., 3200., 540.

7345, 12000., 3000., 540.

7346, 12000., 3000., 1080.

7347, 12000., 3000., 1620.

7348, 12000., 3000., 2160.

7349, 12200., 3000., 5160.

7350, 12200., 3000., 4620.

7351, 12200., 3000., 4080.

7352, 12200., 3000., 3540.

7353, 12200., 3200., 3540.

7354, 12200., 3200., 4080.

7355, 12200., 3200., 4620.

7356, 12200., 3200., 5160.

7357, 12200., 3200., 540.

7358, 12200., 3200., 1080.

7359, 12200., 3200., 1620.

7360, 12200., 3200., 2160.

7361, 12200., 3000., 2160.

7362, 12200., 3000., 1620.

7363, 12200., 3000., 1080.

7364, 12200., 3000., 540.

7365, 12200., 6000., 540.

7366, 12200., 6000., 1080.

7367, 12200., 6000., 1620.

7368, 12200., 6000., 2160.

7369, 12000., 6000., 2160.

7370, 12000., 6000., 1620.

7371, 12000., 6000., 1080.

7372, 12000., 6000., 540.

7373, 12000., 6000., 5160.

7374, 12000., 6000., 4620.

7375, 12000., 6000., 4080.

7376, 12000., 6000., 3540.

7377, 12200., 6000., 3540.

7378, 12200., 6000., 4080.

7379, 12200., 6000., 4620.

7380, 12200., 6000., 5160.

7381, 12000., 6000., 8160.

7382, 12000., 6000., 7620.

7383, 12000., 6000., 7080.

7384, 12000., 6000., 6540.

7385, 12200., 6000., 6540.

7386, 12200., 6000., 7080.

7387, 12200., 6000., 7620.

7388, 12200., 6000., 8160.

7389, 12200., 6200., 2160.

7390, 12200., 6200., 1620.

7391, 12200., 6200., 1080.

7392, 12200., 6200., 540.

7393, 12000., 6200., 540.

7394, 12000., 6200., 1080.

7395, 12000., 6200., 1620.

7396, 12000., 6200., 2160.

7397, 12200., 6200., 5160.

7398, 12200., 6200., 4620.

7399, 12200., 6200., 4080.

7400, 12200., 6200., 3540.

7401, 12000., 6200., 3540.

7402, 12000., 6200., 4080.

7403, 12000., 6200., 4620.

7404, 12000., 6200., 5160.

7405, 12200., 6200., 8160.

7406, 12200., 6200., 7620.

7407, 12200., 6200., 7080.

7408, 12200., 6200., 6540.

7409, 12000., 6200., 6540.

7410, 12000., 6200., 7080.

7411, 12000., 6200., 7620.

7412, 12000., 6200., 8160.

7413, 12000., 9200., 8160.

7414, 12000., 9200., 7620.

7415, 12000., 9200., 7080.

7416, 12000., 9200., 6540.

7417, 12000., 9000., 6540.

7418, 12000., 9000., 7080.

7419, 12000., 9000., 7620.

7420, 12000., 9000., 8160.

7421, 12000., 9200., 5160.

7422, 12000., 9200., 4620.

7423, 12000., 9200., 4080.

7424, 12000., 9200., 3540.

7425, 12000., 9000., 3540.

7426, 12000., 9000., 4080.

7427, 12000., 9000., 4620.

7428, 12000., 9000., 5160.

7429, 12000., 9200., 2160.

7430, 12000., 9200., 1620.

7431, 12000., 9200., 1080.

7432, 12000., 9200., 540.

7433, 12000., 9000., 540.

7434, 12000., 9000., 1080.

7435, 12000., 9000., 1620.

7436, 12000., 9000., 2160.

7437, 12200., 9000., 8160.

7438, 12200., 9000., 7620.

7439, 12200., 9000., 7080.

7440, 12200., 9000., 6540.

7441, 12200., 9200., 6540.

7442, 12200., 9200., 7080.

7443, 12200., 9200., 7620.

7444, 12200., 9200., 8160.

7445, 12200., 9000., 5160.

7446, 12200., 9000., 4620.

7447, 12200., 9000., 4080.

7448, 12200., 9000., 3540.

7449, 12200., 9200., 3540.

7450, 12200., 9200., 4080.

7451, 12200., 9200., 4620.

7452, 12200., 9200., 5160.

7453, 12200., 9200., 540.

7454, 12200., 9200., 1080.

7455, 12200., 9200., 1620.

7456, 12200., 9200., 2160.

7457, 12200., 9000., 2160.

7458, 12200., 9000., 1620.

7459, 12200., 9000., 1080.

7460, 12200., 9000., 540.

7461, 12000., 12200., 2160.

7462, 12000., 12200., 1620.

7463, 12000., 12200., 1080.

7464, 12000., 12200., 540.

7465, 12000., 12000., 540.

7466, 12000., 12000., 1080.

7467, 12000., 12000., 1620.

7468, 12000., 12000., 2160.

7469, 12000., 12200., 5160.

7470, 12000., 12200., 4620.

7471, 12000., 12200., 4080.

7472, 12000., 12200., 3540.

7473, 12000., 12000., 3540.

7474, 12000., 12000., 4080.

7475, 12000., 12000., 4620.

7476, 12000., 12000., 5160.

7477, 12000., 12200., 8160.

7478, 12000., 12200., 7620.

7479, 12000., 12200., 7080.

7480, 12000., 12200., 6540.

7481, 12000., 12000., 6540.

7482, 12000., 12000., 7080.

7483, 12000., 12000., 7620.

7484, 12000., 12000., 8160.

7485, 12200., 12200., 540.

7486, 12200., 12200., 1080.

7487, 12200., 12200., 1620.

7488, 12200., 12200., 2160.

7489, 12200., 12000., 2160.

7490, 12200., 12000., 1620.

7491, 12200., 12000., 1080.

7492, 12200., 12000., 540.

7493, 12200., 12000., 5160.

7494, 12200., 12000., 4620.

7495, 12200., 12000., 4080.

7496, 12200., 12000., 3540.

7497, 12200., 12200., 3540.

7498, 12200., 12200., 4080.

7499, 12200., 12200., 4620.

7500, 12200., 12200., 5160.

7501, 12200., 12000., 8160.

7502, 12200., 12000., 7620.

7503, 12200., 12000., 7080.

7504, 12200., 12000., 6540.

7505, 12200., 12200., 6540.

7506, 12200., 12200., 7080.

7507, 12200., 12200., 7620.

7508, 12200., 12200., 8160.

7509, 12000., 15000., 8160.

7510, 12000., 15000., 7620.

7511, 12000., 15000., 7080.

7512, 12000., 15000., 6540.

7513, 12200., 15000., 6540.

7514, 12200., 15000., 7080.

7515, 12200., 15000., 7620.

7516, 12200., 15000., 8160.

7517, 12000., 15000., 5160.

7518, 12000., 15000., 4620.

7519, 12000., 15000., 4080.

7520, 12000., 15000., 3540.

7521, 12200., 15000., 3540.

7522, 12200., 15000., 4080.

7523, 12200., 15000., 4620.

7524, 12200., 15000., 5160.

7525, 12200., 15000., 540.

7526, 12200., 15000., 1080.

7527, 12200., 15000., 1620.

7528, 12200., 15000., 2160.

7529, 12000., 15000., 2160.

7530, 12000., 15000., 1620.

7531, 12000., 15000., 1080.

7532, 12000., 15000., 540.

7533, 12000., 15200., 6540.

7534, 12000., 15200., 7080.

7535, 12000., 15200., 7620.

7536, 12000., 15200., 8160.

7537, 12000., 15200., 3540.

7538, 12000., 15200., 4080.

7539, 12000., 15200., 4620.

7540, 12000., 15200., 5160.

7541, 12000., 15200., 540.

7542, 12000., 15200., 1080.

7543, 12000., 15200., 1620.

7544, 12000., 15200., 2160.

7545, 12200., 15200., 8160.

7546, 12200., 15200., 7620.

7547, 12200., 15200., 7080.

7548, 12200., 15200., 6540.

7549, 12200., 15200., 5160.

7550, 12200., 15200., 4620.

7551, 12200., 15200., 4080.

7552, 12200., 15200., 3540.

7553, 12200., 15200., 2160.

7554, 12200., 15200., 1620.

7555, 12200., 15200., 1080.

7556, 12200., 15200., 540.

7557, 9200., 12666.667, 2700.

7558, 9200., 13133.333, 2700.

7559, 9200., 13600., 2700.

7560, 9200., 14066.667, 2700.

7561, 9200., 14533.333, 2700.

7562, 9200., 14533.333, 2920.

7563, 9200., 14066.667, 2920.

7564, 9200., 13600., 2920.

7565, 9200., 13133.333, 2920.

7566, 9200., 12666.667, 2920.

7567, 9200., 14533.333, 3000.

7568, 9200., 14066.667, 3000.

7569, 9200., 13600., 3000.

7570, 9200., 13133.333, 3000.

7571, 9200., 12666.667, 3000.

7572, 9000., 14533.333, 2700.

7573, 9000., 14066.667, 2700.

7574, 9000., 13600., 2700.

7575, 9000., 13133.333, 2700.

7576, 9000., 12666.667, 2700.

7577, 9200., 12666.667, 5700.

7578, 9200., 13133.333, 5700.

7579, 9200., 13600., 5700.

7580, 9200., 14066.667, 5700.

7581, 9200., 14533.333, 5700.

7582, 9200., 14533.333, 5920.

7583, 9200., 14066.667, 5920.

7584, 9200., 13600., 5920.

7585, 9200., 13133.333, 5920.

7586, 9200., 12666.667, 5920.

7587, 9200., 14533.333, 6000.

7588, 9200., 14066.667, 6000.

7589, 9200., 13600., 6000.

7590, 9200., 13133.333, 6000.

7591, 9200., 12666.667, 6000.

7592, 9000., 14533.333, 5700.

7593, 9000., 14066.667, 5700.

7594, 9000., 13600., 5700.

7595, 9000., 13133.333, 5700.

7596, 9000., 12666.667, 5700.

7597, 9000., 12666.667, 5920.

7598, 9000., 13133.333, 5920.

7599, 9000., 13600., 5920.

7600, 9000., 14066.667, 5920.

7601, 9000., 14533.333, 5920.

7602, 9000., 12666.667, 6000.

7603, 9000., 13133.333, 6000.

7604, 9000., 13600., 6000.

7605, 9000., 14066.667, 6000.

7606, 9000., 14533.333, 6000.

7607, 9000., 12200., 8160.

7608, 9000., 12200., 7620.

7609, 9000., 12200., 7080.

7610, 9000., 12200., 6540.

7611, 9000., 12000., 6540.

7612, 9000., 12000., 7080.

7613, 9000., 12000., 7620.

7614, 9000., 12000., 8160.

7615, 9000., 12200., 5160.

7616, 9000., 12200., 4620.

7617, 9000., 12200., 4080.

7618, 9000., 12200., 3540.

7619, 9000., 12000., 3540.

7620, 9000., 12000., 4080.

7621, 9000., 12000., 4620.

7622, 9000., 12000., 5160.

7623, 9000., 12000., 540.

7624, 9000., 12000., 1080.

7625, 9000., 12000., 1620.

7626, 9000., 12000., 2160.

7627, 9000., 12200., 2160.

7628, 9000., 12200., 1620.

7629, 9000., 12200., 1080.

7630, 9000., 12200., 540.

7631, 9200., 12000., 8160.

7632, 9200., 12000., 7620.

7633, 9200., 12000., 7080.

7634, 9200., 12000., 6540.

7635, 9200., 12200., 6540.

7636, 9200., 12200., 7080.

7637, 9200., 12200., 7620.

7638, 9200., 12200., 8160.

7639, 9200., 12000., 5160.

7640, 9200., 12000., 4620.

7641, 9200., 12000., 4080.

7642, 9200., 12000., 3540.

7643, 9200., 12200., 3540.

7644, 9200., 12200., 4080.

7645, 9200., 12200., 4620.

7646, 9200., 12200., 5160.

7647, 9200., 12000., 2160.

7648, 9200., 12000., 1620.

7649, 9200., 12000., 1080.

7650, 9200., 12000., 540.

7651, 9200., 12200., 540.

7652, 9200., 12200., 1080.

7653, 9200., 12200., 1620.

7654, 9200., 12200., 2160.

7655, 9200., 9666.66699, 2700.

7656, 9200., 10133.333, 2700.

7657, 9200., 10600., 2700.

7658, 9200., 11066.667, 2700.

7659, 9200., 11533.333, 2700.

7660, 9200., 11533.333, 2920.

7661, 9200., 11066.667, 2920.

7662, 9200., 10600., 2920.

7663, 9200., 10133.333, 2920.

7664, 9200., 9666.66699, 2920.

7665, 9200., 11533.333, 3000.

7666, 9200., 11066.667, 3000.

7667, 9200., 10600., 3000.

7668, 9200., 10133.333, 3000.

7669, 9200., 9666.66699, 3000.

7670, 9000., 11533.333, 2700.

7671, 9000., 11066.667, 2700.

7672, 9000., 10600., 2700.

7673, 9000., 10133.333, 2700.

7674, 9000., 9666.66699, 2700.

7675, 9200., 9666.66699, 5700.

7676, 9200., 10133.333, 5700.

7677, 9200., 10600., 5700.

7678, 9200., 11066.667, 5700.

7679, 9200., 11533.333, 5700.

7680, 9200., 11533.333, 5920.

7681, 9200., 11066.667, 5920.

7682, 9200., 10600., 5920.

7683, 9200., 10133.333, 5920.

7684, 9200., 9666.66699, 5920.

7685, 9200., 11533.333, 6000.

7686, 9200., 11066.667, 6000.

7687, 9200., 10600., 6000.

7688, 9200., 10133.333, 6000.

7689, 9200., 9666.66699, 6000.

7690, 9000., 11533.333, 5700.

7691, 9000., 11066.667, 5700.

7692, 9000., 10600., 5700.

7693, 9000., 10133.333, 5700.

7694, 9000., 9666.66699, 5700.

7695, 9000., 9666.66699, 5920.

7696, 9000., 10133.333, 5920.

7697, 9000., 10600., 5920.

7698, 9000., 11066.667, 5920.

7699, 9000., 11533.333, 5920.

7700, 9000., 9666.66699, 6000.

7701, 9000., 10133.333, 6000.

7702, 9000., 10600., 6000.

7703, 9000., 11066.667, 6000.

7704, 9000., 11533.333, 6000.

7705, 9000., 9200., 8160.

7706, 9000., 9200., 7620.

7707, 9000., 9200., 7080.

7708, 9000., 9200., 6540.

7709, 9000., 9000., 6540.

7710, 9000., 9000., 7080.

7711, 9000., 9000., 7620.

7712, 9000., 9000., 8160.

7713, 9000., 9200., 5160.

7714, 9000., 9200., 4620.

7715, 9000., 9200., 4080.

7716, 9000., 9200., 3540.

7717, 9000., 9000., 3540.

7718, 9000., 9000., 4080.

7719, 9000., 9000., 4620.

7720, 9000., 9000., 5160.

7721, 9000., 9000., 540.

7722, 9000., 9000., 1080.

7723, 9000., 9000., 1620.

7724, 9000., 9000., 2160.

7725, 9000., 9200., 2160.

7726, 9000., 9200., 1620.

7727, 9000., 9200., 1080.

7728, 9000., 9200., 540.

7729, 9200., 9000., 8160.

7730, 9200., 9000., 7620.

7731, 9200., 9000., 7080.

7732, 9200., 9000., 6540.

7733, 9200., 9200., 6540.

7734, 9200., 9200., 7080.

7735, 9200., 9200., 7620.

7736, 9200., 9200., 8160.

7737, 9200., 9000., 5160.

7738, 9200., 9000., 4620.

7739, 9200., 9000., 4080.

7740, 9200., 9000., 3540.

7741, 9200., 9200., 3540.

7742, 9200., 9200., 4080.

7743, 9200., 9200., 4620.

7744, 9200., 9200., 5160.

7745, 9200., 9000., 2160.

7746, 9200., 9000., 1620.

7747, 9200., 9000., 1080.

7748, 9200., 9000., 540.

7749, 9200., 9200., 540.

7750, 9200., 9200., 1080.

7751, 9200., 9200., 1620.

7752, 9200., 9200., 2160.

7753, 9000., 8533.33301, 2700.

7754, 9000., 8066.6665, 2700.

7755, 9000., 7600., 2700.

7756, 9000., 7133.3335, 2700.

7757, 9000., 6666.6665, 2700.

7758, 9200., 8533.33301, 3000.

7759, 9200., 8066.6665, 3000.

7760, 9200., 7600., 3000.

7761, 9200., 7133.3335, 3000.

7762, 9200., 6666.6665, 3000.

7763, 9200., 6666.6665, 2920.

7764, 9200., 7133.3335, 2920.

7765, 9200., 7600., 2920.

7766, 9200., 8066.6665, 2920.

7767, 9200., 8533.33301, 2920.

7768, 9200., 6666.6665, 2700.

7769, 9200., 7133.3335, 2700.

7770, 9200., 7600., 2700.

7771, 9200., 8066.6665, 2700.

7772, 9200., 8533.33301, 2700.

7773, 9000., 6666.6665, 6000.

7774, 9000., 7133.3335, 6000.

7775, 9000., 7600., 6000.

7776, 9000., 8066.6665, 6000.

7777, 9000., 8533.33301, 6000.

7778, 9000., 8533.33301, 5920.

7779, 9000., 8066.6665, 5920.

7780, 9000., 7600., 5920.

7781, 9000., 7133.3335, 5920.

7782, 9000., 6666.6665, 5920.

7783, 9000., 8533.33301, 5700.

7784, 9000., 8066.6665, 5700.

7785, 9000., 7600., 5700.

7786, 9000., 7133.3335, 5700.

7787, 9000., 6666.6665, 5700.

7788, 9200., 8533.33301, 6000.

7789, 9200., 8066.6665, 6000.

7790, 9200., 7600., 6000.

7791, 9200., 7133.3335, 6000.

7792, 9200., 6666.6665, 6000.

7793, 9200., 6666.6665, 5920.

7794, 9200., 7133.3335, 5920.

7795, 9200., 7600., 5920.

7796, 9200., 8066.6665, 5920.

7797, 9200., 8533.33301, 5920.

7798, 9200., 6666.6665, 5700.

7799, 9200., 7133.3335, 5700.

7800, 9200., 7600., 5700.

7801, 9200., 8066.6665, 5700.

7802, 9200., 8533.33301, 5700.

7803, 9200., 6000., 540.

7804, 9200., 6000., 1080.

7805, 9200., 6000., 1620.

7806, 9200., 6000., 2160.

7807, 9000., 6000., 2160.

7808, 9000., 6000., 1620.

7809, 9000., 6000., 1080.

7810, 9000., 6000., 540.

7811, 9000., 6000., 5160.

7812, 9000., 6000., 4620.

7813, 9000., 6000., 4080.

7814, 9000., 6000., 3540.

7815, 9200., 6000., 3540.

7816, 9200., 6000., 4080.

7817, 9200., 6000., 4620.

7818, 9200., 6000., 5160.

7819, 9000., 6000., 8160.

7820, 9000., 6000., 7620.

7821, 9000., 6000., 7080.

7822, 9000., 6000., 6540.

7823, 9200., 6000., 6540.

7824, 9200., 6000., 7080.

7825, 9200., 6000., 7620.

7826, 9200., 6000., 8160.

7827, 9200., 6200., 2160.

7828, 9200., 6200., 1620.

7829, 9200., 6200., 1080.

7830, 9200., 6200., 540.

7831, 9000., 6200., 540.

7832, 9000., 6200., 1080.

7833, 9000., 6200., 1620.

7834, 9000., 6200., 2160.

7835, 9200., 6200., 5160.

7836, 9200., 6200., 4620.

7837, 9200., 6200., 4080.

7838, 9200., 6200., 3540.

7839, 9000., 6200., 3540.

7840, 9000., 6200., 4080.

7841, 9000., 6200., 4620.

7842, 9000., 6200., 5160.

7843, 9200., 6200., 8160.

7844, 9200., 6200., 7620.

7845, 9200., 6200., 7080.

7846, 9200., 6200., 6540.

7847, 9000., 6200., 6540.

7848, 9000., 6200., 7080.

7849, 9000., 6200., 7620.

7850, 9000., 6200., 8160.

7851, 9200., 3666.66675, 2700.

7852, 9200., 4133.3335, 2700.

7853, 9200., 4600., 2700.

7854, 9200., 5066.6665, 2700.

7855, 9200., 5533.3335, 2700.

7856, 9200., 5533.3335, 2920.

7857, 9200., 5066.6665, 2920.

7858, 9200., 4600., 2920.

7859, 9200., 4133.3335, 2920.

7860, 9200., 3666.66675, 2920.

7861, 9200., 5533.3335, 3000.

7862, 9200., 5066.6665, 3000.

7863, 9200., 4600., 3000.

7864, 9200., 4133.3335, 3000.

7865, 9200., 3666.66675, 3000.

7866, 9000., 5533.3335, 2700.

7867, 9000., 5066.6665, 2700.

7868, 9000., 4600., 2700.

7869, 9000., 4133.3335, 2700.

7870, 9000., 3666.66675, 2700.

7871, 9200., 3000., 5160.

7872, 9200., 3000., 4620.

7873, 9200., 3000., 4080.

7874, 9200., 3000., 3540.

7875, 9200., 3200., 3540.

7876, 9200., 3200., 4080.

7877, 9200., 3200., 4620.

7878, 9200., 3200., 5160.

7879, 9200., 3000., 2160.

7880, 9200., 3000., 1620.

7881, 9200., 3000., 1080.

7882, 9200., 3000., 540.

7883, 9200., 3200., 540.

7884, 9200., 3200., 1080.

7885, 9200., 3200., 1620.

7886, 9200., 3200., 2160.

7887, 9000., 3200., 5160.

7888, 9000., 3200., 4620.

7889, 9000., 3200., 4080.

7890, 9000., 3200., 3540.

7891, 9000., 3000., 3540.

7892, 9000., 3000., 4080.

7893, 9000., 3000., 4620.

7894, 9000., 3000., 5160.

7895, 9000., 3200., 2160.

7896, 9000., 3200., 1620.

7897, 9000., 3200., 1080.

7898, 9000., 3200., 540.

7899, 9000., 3000., 540.

7900, 9000., 3000., 1080.

7901, 9000., 3000., 1620.

7902, 9000., 3000., 2160.

7903, 9000., 2533.33325, 2700.

7904, 9000., 2066.66675, 2700.

7905, 9000., 1600., 2700.

7906, 9000., 1133.33337, 2700.

7907, 9000., 666.666687, 2700.

7908, 9200., 2533.33325, 3000.

7909, 9200., 2066.66675, 3000.

7910, 9200., 1600., 3000.

7911, 9200., 1133.33337, 3000.

7912, 9200., 666.666687, 3000.

7913, 9200., 666.666687, 2920.

7914, 9200., 1133.33337, 2920.

7915, 9200., 1600., 2920.

7916, 9200., 2066.66675, 2920.

7917, 9200., 2533.33325, 2920.

7918, 9200., 666.666687, 2700.

7919, 9200., 1133.33337, 2700.

7920, 9200., 1600., 2700.

7921, 9200., 2066.66675, 2700.

7922, 9200., 2533.33325, 2700.

7923, 9200., 0., 5160.

7924, 9200., 0., 4620.

7925, 9200., 0., 4080.

7926, 9200., 0., 3540.

7927, 9200., 200., 3540.

7928, 9200., 200., 4080.

7929, 9200., 200., 4620.

7930, 9200., 200., 5160.

7931, 9200., 0., 2160.

7932, 9200., 0., 1620.

7933, 9200., 0., 1080.

7934, 9200., 0., 540.

7935, 9200., 200., 540.

7936, 9200., 200., 1080.

7937, 9200., 200., 1620.

7938, 9200., 200., 2160.

7939, 9000., 200., 5160.

7940, 9000., 200., 4620.

7941, 9000., 200., 4080.

7942, 9000., 200., 3540.

7943, 9000., 0., 3540.

7944, 9000., 0., 4080.

7945, 9000., 0., 4620.

7946, 9000., 0., 5160.

7947, 9000., 200., 2160.

7948, 9000., 200., 1620.

7949, 9000., 200., 1080.

7950, 9000., 200., 540.

7951, 9000., 0., 540.

7952, 9000., 0., 1080.

7953, 9000., 0., 1620.

7954, 9000., 0., 2160.

7955, 9200., 3666.66675, 5700.

7956, 9200., 4133.3335, 5700.

7957, 9200., 4600., 5700.

7958, 9200., 5066.6665, 5700.

7959, 9200., 5533.3335, 5700.

7960, 9200., 5533.3335, 5920.

7961, 9200., 5066.6665, 5920.

7962, 9200., 4600., 5920.

7963, 9200., 4133.3335, 5920.

7964, 9200., 3666.66675, 5920.

7965, 9200., 5533.3335, 6000.

7966, 9200., 5066.6665, 6000.

7967, 9200., 4600., 6000.

7968, 9200., 4133.3335, 6000.

7969, 9200., 3666.66675, 6000.

7970, 9000., 5533.3335, 5700.

7971, 9000., 5066.6665, 5700.

7972, 9000., 4600., 5700.

7973, 9000., 4133.3335, 5700.

7974, 9000., 3666.66675, 5700.

7975, 9000., 3666.66675, 5920.

7976, 9000., 4133.3335, 5920.

7977, 9000., 4600., 5920.

7978, 9000., 5066.6665, 5920.

7979, 9000., 5533.3335, 5920.

7980, 9000., 3666.66675, 6000.

7981, 9000., 4133.3335, 6000.

7982, 9000., 4600., 6000.

7983, 9000., 5066.6665, 6000.

7984, 9000., 5533.3335, 6000.

7985, 9000., 666.666687, 6000.

7986, 9000., 1133.33337, 6000.

7987, 9000., 1600., 6000.

7988, 9000., 2066.66675, 6000.

7989, 9000., 2533.33325, 6000.

7990, 9000., 2533.33325, 5920.

7991, 9000., 2066.66675, 5920.

7992, 9000., 1600., 5920.

7993, 9000., 1133.33337, 5920.

7994, 9000., 666.666687, 5920.

7995, 9000., 2533.33325, 5700.

7996, 9000., 2066.66675, 5700.

7997, 9000., 1600., 5700.

7998, 9000., 1133.33337, 5700.

7999, 9000., 666.666687, 5700.

8000, 9200., 2533.33325, 6000.

8001, 9200., 2066.66675, 6000.

8002, 9200., 1600., 6000.

8003, 9200., 1133.33337, 6000.

8004, 9200., 666.666687, 6000.

8005, 9200., 666.666687, 5920.

8006, 9200., 1133.33337, 5920.

8007, 9200., 1600., 5920.

8008, 9200., 2066.66675, 5920.

8009, 9200., 2533.33325, 5920.

8010, 9200., 666.666687, 5700.

8011, 9200., 1133.33337, 5700.

8012, 9200., 1600., 5700.

8013, 9200., 2066.66675, 5700.

8014, 9200., 2533.33325, 5700.

8015, 9000., 11533.333, 8700.

8016, 9000., 11066.667, 8700.

8017, 9000., 10600., 8700.

8018, 9000., 10133.333, 8700.

8019, 9000., 9666.66699, 8700.

8020, 9200., 11533.333, 9000.

8021, 9200., 11066.667, 9000.

8022, 9200., 10600., 9000.

8023, 9200., 10133.333, 9000.

8024, 9200., 9666.66699, 9000.

8025, 9200., 9666.66699, 8920.

8026, 9200., 10133.333, 8920.

8027, 9200., 10600., 8920.

8028, 9200., 11066.667, 8920.

8029, 9200., 11533.333, 8920.

8030, 9200., 9666.66699, 8700.

8031, 9200., 10133.333, 8700.

8032, 9200., 10600., 8700.

8033, 9200., 11066.667, 8700.

8034, 9200., 11533.333, 8700.

8035, 9000., 14533.333, 8700.

8036, 9000., 14066.667, 8700.

8037, 9000., 13600., 8700.

8038, 9000., 13133.333, 8700.

8039, 9000., 12666.667, 8700.

8040, 9200., 14533.333, 9000.

8041, 9200., 14066.667, 9000.

8042, 9200., 13600., 9000.

8043, 9200., 13133.333, 9000.

8044, 9200., 12666.667, 9000.

8045, 9200., 12666.667, 8920.

8046, 9200., 13133.333, 8920.

8047, 9200., 13600., 8920.

8048, 9200., 14066.667, 8920.

8049, 9200., 14533.333, 8920.

8050, 9200., 12666.667, 8700.

8051, 9200., 13133.333, 8700.

8052, 9200., 13600., 8700.

8053, 9200., 14066.667, 8700.

8054, 9200., 14533.333, 8700.

8055, 9000., 15000., 8160.

8056, 9000., 15000., 7620.

8057, 9000., 15000., 7080.

8058, 9000., 15000., 6540.

8059, 9200., 15000., 6540.

8060, 9200., 15000., 7080.

8061, 9200., 15000., 7620.

8062, 9200., 15000., 8160.

8063, 9000., 15000., 5160.

8064, 9000., 15000., 4620.

8065, 9000., 15000., 4080.

8066, 9000., 15000., 3540.

8067, 9200., 15000., 3540.

8068, 9200., 15000., 4080.

8069, 9200., 15000., 4620.

8070, 9200., 15000., 5160.

8071, 9200., 15000., 540.

8072, 9200., 15000., 1080.

8073, 9200., 15000., 1620.

8074, 9200., 15000., 2160.

8075, 9000., 15000., 2160.

8076, 9000., 15000., 1620.

8077, 9000., 15000., 1080.

8078, 9000., 15000., 540.

8079, 9000., 15200., 6540.

8080, 9000., 15200., 7080.

8081, 9000., 15200., 7620.

8082, 9000., 15200., 8160.

8083, 9000., 15200., 3540.

8084, 9000., 15200., 4080.

8085, 9000., 15200., 4620.

8086, 9000., 15200., 5160.

8087, 9000., 15200., 540.

8088, 9000., 15200., 1080.

8089, 9000., 15200., 1620.

8090, 9000., 15200., 2160.

8091, 9200., 15200., 8160.

8092, 9200., 15200., 7620.

8093, 9200., 15200., 7080.

8094, 9200., 15200., 6540.

8095, 9200., 15200., 5160.

8096, 9200., 15200., 4620.

8097, 9200., 15200., 4080.

8098, 9200., 15200., 3540.

8099, 9200., 15200., 2160.

8100, 9200., 15200., 1620.

8101, 9200., 15200., 1080.

8102, 9200., 15200., 540.

8103, 0., 12200., 2160.

8104, 0., 12200., 1620.

8105, 0., 12200., 1080.

8106, 0., 12200., 540.

8107, 0., 12000., 540.

8108, 0., 12000., 1080.

8109, 0., 12000., 1620.

8110, 0., 12000., 2160.

8111, 200., 12200., 540.

8112, 200., 12200., 1080.

8113, 200., 12200., 1620.

8114, 200., 12200., 2160.

8115, 200., 12000., 2160.

8116, 200., 12000., 1620.

8117, 200., 12000., 1080.

8118, 200., 12000., 540.

8119, 0., 14533.333, 2700.

8120, 0., 14066.667, 2700.

8121, 0., 13600., 2700.

8122, 0., 13133.333, 2700.

8123, 0., 12666.667, 2700.

8124, 0., 11533.333, 2700.

8125, 0., 11066.667, 2700.

8126, 0., 10600., 2700.

8127, 0., 10133.333, 2700.

8128, 0., 9666.66699, 2700.

8129, 0., 8533.33301, 2700.

8130, 0., 8066.6665, 2700.

8131, 0., 7600., 2700.

8132, 0., 7133.3335, 2700.

8133, 0., 6666.6665, 2700.

8134, 0., 5533.3335, 2700.

8135, 0., 5066.6665, 2700.

8136, 0., 4600., 2700.

8137, 0., 4133.3335, 2700.

8138, 0., 3666.66675, 2700.

8139, 0., 2533.33325, 2700.

8140, 0., 2066.66675, 2700.

8141, 0., 1600., 2700.

8142, 0., 1133.33337, 2700.

8143, 0., 666.666687, 2700.

8144, 0., 0., 2850.

8145, 0., 12666.667, 3000.

8146, 0., 13133.333, 3000.

8147, 0., 13600., 3000.

8148, 0., 14066.667, 3000.

8149, 0., 14533.333, 3000.

8150, 0., 9666.66699, 3000.

8151, 0., 10133.333, 3000.

8152, 0., 10600., 3000.

8153, 0., 11066.667, 3000.

8154, 0., 11533.333, 3000.

8155, 0., 6666.6665, 3000.

8156, 0., 7133.3335, 3000.

8157, 0., 7600., 3000.

8158, 0., 8066.6665, 3000.

8159, 0., 8533.33301, 3000.

8160, 0., 3666.66675, 3000.

8161, 0., 4133.3335, 3000.

8162, 0., 4600., 3000.

8163, 0., 5066.6665, 3000.

8164, 0., 5533.3335, 3000.

8165, 0., 666.666687, 3000.

8166, 0., 1133.33337, 3000.

8167, 0., 1600., 3000.

8168, 0., 2066.66675, 3000.

8169, 0., 2533.33325, 3000.

8170, 0., 15200., 2850.

8171, 200., 14533.333, 2920.

8172, 200., 14066.667, 2920.

8173, 200., 13600., 2920.

8174, 200., 13133.333, 2920.

8175, 200., 12666.667, 2920.

8176, 200., 12666.667, 2700.

8177, 200., 13133.333, 2700.

8178, 200., 13600., 2700.

8179, 200., 14066.667, 2700.

8180, 200., 14533.333, 2700.

8181, 200., 11533.333, 2920.

8182, 200., 11066.667, 2920.

8183, 200., 10600., 2920.

8184, 200., 10133.333, 2920.

8185, 200., 9666.66699, 2920.

8186, 200., 9666.66699, 2700.

8187, 200., 10133.333, 2700.

8188, 200., 10600., 2700.

8189, 200., 11066.667, 2700.

8190, 200., 11533.333, 2700.

8191, 200., 8533.33301, 2920.

8192, 200., 8066.6665, 2920.

8193, 200., 7600., 2920.

8194, 200., 7133.3335, 2920.

8195, 200., 6666.6665, 2920.

8196, 200., 6666.6665, 2700.

8197, 200., 7133.3335, 2700.

8198, 200., 7600., 2700.

8199, 200., 8066.6665, 2700.

8200, 200., 8533.33301, 2700.

8201, 200., 5533.3335, 2920.

8202, 200., 5066.6665, 2920.

8203, 200., 4600., 2920.

8204, 200., 4133.3335, 2920.

8205, 200., 3666.66675, 2920.

8206, 200., 3666.66675, 2700.

8207, 200., 4133.3335, 2700.

8208, 200., 4600., 2700.

8209, 200., 5066.6665, 2700.

8210, 200., 5533.3335, 2700.

8211, 200., 2533.33325, 2920.

8212, 200., 2066.66675, 2920.

8213, 200., 1600., 2920.

8214, 200., 1133.33337, 2920.

8215, 200., 666.666687, 2920.

8216, 200., 666.666687, 2700.

8217, 200., 1133.33337, 2700.

8218, 200., 1600., 2700.

8219, 200., 2066.66675, 2700.

8220, 200., 2533.33325, 2700.

8221, 200., 2533.33325, 3000.

8222, 200., 2066.66675, 3000.

8223, 200., 1600., 3000.

8224, 200., 1133.33337, 3000.

8225, 200., 666.666687, 3000.

8226, 200., 5533.3335, 3000.

8227, 200., 5066.6665, 3000.

8228, 200., 4600., 3000.

8229, 200., 4133.3335, 3000.

8230, 200., 3666.66675, 3000.

8231, 200., 8533.33301, 3000.

8232, 200., 8066.6665, 3000.

8233, 200., 7600., 3000.

8234, 200., 7133.3335, 3000.

8235, 200., 6666.6665, 3000.

8236, 200., 11533.333, 3000.

8237, 200., 11066.667, 3000.

8238, 200., 10600., 3000.

8239, 200., 10133.333, 3000.

8240, 200., 9666.66699, 3000.

8241, 200., 14533.333, 3000.

8242, 200., 14066.667, 3000.

8243, 200., 13600., 3000.

8244, 200., 13133.333, 3000.

8245, 200., 12666.667, 3000.

8246, 0., 15000., 2160.

8247, 0., 15000., 1620.

8248, 0., 15000., 1080.

8249, 0., 15000., 540.

8250, 200., 15000., 540.

8251, 200., 15000., 1080.

8252, 200., 15000., 1620.

8253, 200., 15000., 2160.

8254, 0., 15200., 540.

8255, 0., 15200., 1080.

8256, 0., 15200., 1620.

8257, 0., 15200., 2160.

8258, 200., 15200., 2160.

8259, 200., 15200., 1620.

8260, 200., 15200., 1080.

8261, 200., 15200., 540.

8262, 200., 200., 2160.

8263, 200., 200., 1620.

8264, 200., 200., 1080.

8265, 200., 200., 540.

8266, 0., 200., 540.

8267, 0., 200., 1080.

8268, 0., 200., 1620.

8269, 0., 200., 2160.

8270, 200., 0., 540.

8271, 200., 0., 1080.

8272, 200., 0., 1620.

8273, 200., 0., 2160.

8274, 0., 0., 2160.

8275, 0., 0., 1620.

8276, 0., 0., 1080.

8277, 0., 0., 540.

8278, 200., 3200., 2160.

8279, 200., 3200., 1620.

8280, 200., 3200., 1080.

8281, 200., 3200., 540.

8282, 0., 3200., 540.

8283, 0., 3200., 1080.

8284, 0., 3200., 1620.

8285, 0., 3200., 2160.

8286, 200., 3000., 540.

8287, 200., 3000., 1080.

8288, 200., 3000., 1620.

8289, 200., 3000., 2160.

8290, 0., 3000., 2160.

8291, 0., 3000., 1620.

8292, 0., 3000., 1080.

8293, 0., 3000., 540.

8294, 200., 6200., 2160.

8295, 200., 6200., 1620.

8296, 200., 6200., 1080.

8297, 200., 6200., 540.

8298, 0., 6200., 540.

8299, 0., 6200., 1080.

8300, 0., 6200., 1620.

8301, 0., 6200., 2160.

8302, 200., 6000., 540.

8303, 200., 6000., 1080.

8304, 200., 6000., 1620.

8305, 200., 6000., 2160.

8306, 0., 6000., 2160.

8307, 0., 6000., 1620.

8308, 0., 6000., 1080.

8309, 0., 6000., 540.

8310, 0., 15000., 5160.

8311, 0., 15000., 4620.

8312, 0., 15000., 4080.

8313, 0., 15000., 3540.

8314, 200., 15000., 3540.

8315, 200., 15000., 4080.

8316, 200., 15000., 4620.

8317, 200., 15000., 5160.

8318, 0., 15200., 3540.

8319, 0., 15200., 4080.

8320, 0., 15200., 4620.

8321, 0., 15200., 5160.

8322, 200., 15200., 5160.

8323, 200., 15200., 4620.

8324, 200., 15200., 4080.

8325, 200., 15200., 3540.

8326, 0., 12000., 5160.

8327, 0., 12000., 4620.

8328, 0., 12000., 4080.

8329, 0., 12000., 3540.

8330, 200., 12000., 3540.

8331, 200., 12000., 4080.

8332, 200., 12000., 4620.

8333, 200., 12000., 5160.

8334, 0., 12200., 3540.

8335, 0., 12200., 4080.

8336, 0., 12200., 4620.

8337, 0., 12200., 5160.

8338, 200., 12200., 5160.

8339, 200., 12200., 4620.

8340, 200., 12200., 4080.

8341, 200., 12200., 3540.

8342, 0., 9000., 5160.

8343, 0., 9000., 4620.

8344, 0., 9000., 4080.

8345, 0., 9000., 3540.

8346, 200., 9000., 3540.

8347, 200., 9000., 4080.

8348, 200., 9000., 4620.

8349, 200., 9000., 5160.

8350, 0., 9200., 3540.

8351, 0., 9200., 4080.

8352, 0., 9200., 4620.

8353, 0., 9200., 5160.

8354, 200., 9200., 5160.

8355, 200., 9200., 4620.

8356, 200., 9200., 4080.

8357, 200., 9200., 3540.

8358, 0., 6000., 5160.

8359, 0., 6000., 4620.

8360, 0., 6000., 4080.

8361, 0., 6000., 3540.

8362, 200., 6000., 3540.

8363, 200., 6000., 4080.

8364, 200., 6000., 4620.

8365, 200., 6000., 5160.

8366, 0., 6200., 3540.

8367, 0., 6200., 4080.

8368, 0., 6200., 4620.

8369, 0., 6200., 5160.

8370, 200., 6200., 5160.

8371, 200., 6200., 4620.

8372, 200., 6200., 4080.

8373, 200., 6200., 3540.

8374, 0., 3000., 5160.

8375, 0., 3000., 4620.

8376, 0., 3000., 4080.

8377, 0., 3000., 3540.

8378, 200., 3000., 3540.

8379, 200., 3000., 4080.

8380, 200., 3000., 4620.

8381, 200., 3000., 5160.

8382, 0., 3200., 3540.

8383, 0., 3200., 4080.

8384, 0., 3200., 4620.

8385, 0., 3200., 5160.

8386, 200., 3200., 5160.

8387, 200., 3200., 4620.

8388, 200., 3200., 4080.

8389, 200., 3200., 3540.

8390, 0., 200., 3540.

8391, 0., 200., 4080.

8392, 0., 200., 4620.

8393, 0., 200., 5160.

8394, 200., 200., 5160.

8395, 200., 200., 4620.

8396, 200., 200., 4080.

8397, 200., 200., 3540.

8398, 0., 0., 5160.

8399, 0., 0., 4620.

8400, 0., 0., 4080.

8401, 0., 0., 3540.

8402, 200., 0., 3540.

8403, 200., 0., 4080.

8404, 200., 0., 4620.

8405, 200., 0., 5160.

8406, 0., 15000., 6540.

8407, 0., 15000., 7080.

8408, 0., 15000., 7620.

8409, 0., 15000., 8160.

8410, 0., 15200., 8160.

8411, 0., 15200., 7620.

8412, 0., 15200., 7080.

8413, 0., 15200., 6540.

8414, 200., 15000., 8160.

8415, 200., 15000., 7620.

8416, 200., 15000., 7080.

8417, 200., 15000., 6540.

8418, 200., 15200., 6540.

8419, 200., 15200., 7080.

8420, 200., 15200., 7620.

8421, 200., 15200., 8160.

8422, 200., 11533.333, 5700.

8423, 200., 11066.667, 5700.

8424, 200., 10600., 5700.

8425, 200., 10133.333, 5700.

8426, 200., 9666.66699, 5700.

8427, 0., 9666.66699, 5700.

8428, 0., 10133.333, 5700.

8429, 0., 10600., 5700.

8430, 0., 11066.667, 5700.

8431, 0., 11533.333, 5700.

8432, 200., 8533.33301, 5700.

8433, 200., 8066.6665, 5700.

8434, 200., 7600., 5700.

8435, 200., 7133.3335, 5700.

8436, 200., 6666.6665, 5700.

8437, 0., 6666.6665, 5700.

8438, 0., 7133.3335, 5700.

8439, 0., 7600., 5700.

8440, 0., 8066.6665, 5700.

8441, 0., 8533.33301, 5700.

8442, 200., 5533.3335, 5700.

8443, 200., 5066.6665, 5700.

8444, 200., 4600., 5700.

8445, 200., 4133.3335, 5700.

8446, 200., 3666.66675, 5700.

8447, 0., 3666.66675, 5700.

8448, 0., 4133.3335, 5700.

8449, 0., 4600., 5700.

8450, 0., 5066.6665, 5700.

8451, 0., 5533.3335, 5700.

8452, 0., 666.666687, 5700.

8453, 0., 1133.33337, 5700.

8454, 0., 1600., 5700.

8455, 0., 2066.66675, 5700.

8456, 0., 2533.33325, 5700.

8457, 200., 2533.33325, 5700.

8458, 200., 2066.66675, 5700.

8459, 200., 1600., 5700.

8460, 200., 1133.33337, 5700.

8461, 200., 666.666687, 5700.

8462, 0., 12666.667, 5700.

8463, 0., 13133.333, 5700.

8464, 0., 13600., 5700.

8465, 0., 14066.667, 5700.

8466, 0., 14533.333, 5700.

8467, 200., 14533.333, 5700.

8468, 200., 14066.667, 5700.

8469, 200., 13600., 5700.

8470, 200., 13133.333, 5700.

8471, 200., 12666.667, 5700.

8472, 200., 666.666687, 6000.

8473, 200., 1133.33337, 6000.

8474, 200., 1600., 6000.

8475, 200., 2066.66675, 6000.

8476, 200., 2533.33325, 6000.

8477, 0., 2533.33325, 6000.

8478, 0., 2066.66675, 6000.

8479, 0., 1600., 6000.

8480, 0., 1133.33337, 6000.

8481, 0., 666.666687, 6000.

8482, 200., 3666.66675, 6000.

8483, 200., 4133.3335, 6000.

8484, 200., 4600., 6000.

8485, 200., 5066.6665, 6000.

8486, 200., 5533.3335, 6000.

8487, 0., 5533.3335, 6000.

8488, 0., 5066.6665, 6000.

8489, 0., 4600., 6000.

8490, 0., 4133.3335, 6000.

8491, 0., 3666.66675, 6000.

8492, 200., 6666.6665, 6000.

8493, 200., 7133.3335, 6000.

8494, 200., 7600., 6000.

8495, 200., 8066.6665, 6000.

8496, 200., 8533.33301, 6000.

8497, 0., 8533.33301, 6000.

8498, 0., 8066.6665, 6000.

8499, 0., 7600., 6000.

8500, 0., 7133.3335, 6000.

8501, 0., 6666.6665, 6000.

8502, 200., 9666.66699, 6000.

8503, 200., 10133.333, 6000.

8504, 200., 10600., 6000.

8505, 200., 11066.667, 6000.

8506, 200., 11533.333, 6000.

8507, 0., 11533.333, 6000.

8508, 0., 11066.667, 6000.

8509, 0., 10600., 6000.

8510, 0., 10133.333, 6000.

8511, 0., 9666.66699, 6000.

8512, 200., 12666.667, 6000.

8513, 200., 13133.333, 6000.

8514, 200., 13600., 6000.

8515, 200., 14066.667, 6000.

8516, 200., 14533.333, 6000.

8517, 0., 14533.333, 6000.

8518, 0., 14066.667, 6000.

8519, 0., 13600., 6000.

8520, 0., 13133.333, 6000.

8521, 0., 12666.667, 6000.

8522, 200., 11533.333, 5920.

8523, 200., 11066.667, 5920.

8524, 200., 10600., 5920.

8525, 200., 10133.333, 5920.

8526, 200., 9666.66699, 5920.

8527, 200., 8533.33301, 5920.

8528, 200., 8066.6665, 5920.

8529, 200., 7600., 5920.

8530, 200., 7133.3335, 5920.

8531, 200., 6666.6665, 5920.

8532, 200., 5533.3335, 5920.

8533, 200., 5066.6665, 5920.

8534, 200., 4600., 5920.

8535, 200., 4133.3335, 5920.

8536, 200., 3666.66675, 5920.

8537, 200., 2533.33325, 5920.

8538, 200., 2066.66675, 5920.

8539, 200., 1600., 5920.

8540, 200., 1133.33337, 5920.

8541, 200., 666.666687, 5920.

8542, 200., 14533.333, 5920.

8543, 200., 14066.667, 5920.

8544, 200., 13600., 5920.

8545, 200., 13133.333, 5920.

8546, 200., 12666.667, 5920.

8547, 0., 15200., 5850.

8548, 0., 0., 5850.

8549, 200., 200., 8160.

8550, 200., 200., 7620.

8551, 200., 200., 7080.

8552, 200., 200., 6540.

8553, 0., 200., 6540.

8554, 0., 200., 7080.

8555, 0., 200., 7620.

8556, 0., 200., 8160.

8557, 200., 0., 6540.

8558, 200., 0., 7080.

8559, 200., 0., 7620.

8560, 200., 0., 8160.

8561, 0., 0., 8160.

8562, 0., 0., 7620.

8563, 0., 0., 7080.

8564, 0., 0., 6540.

8565, 200., 3200., 8160.

8566, 200., 3200., 7620.

8567, 200., 3200., 7080.

8568, 200., 3200., 6540.

8569, 0., 3200., 6540.

8570, 0., 3200., 7080.

8571, 0., 3200., 7620.

8572, 0., 3200., 8160.

8573, 200., 3000., 6540.

8574, 200., 3000., 7080.

8575, 200., 3000., 7620.

8576, 200., 3000., 8160.

8577, 0., 3000., 8160.

8578, 0., 3000., 7620.

8579, 0., 3000., 7080.

8580, 0., 3000., 6540.

8581, 200., 6200., 8160.

8582, 200., 6200., 7620.

8583, 200., 6200., 7080.

8584, 200., 6200., 6540.

8585, 0., 6200., 6540.

8586, 0., 6200., 7080.

8587, 0., 6200., 7620.

8588, 0., 6200., 8160.

8589, 200., 6000., 6540.

8590, 200., 6000., 7080.

8591, 200., 6000., 7620.

8592, 200., 6000., 8160.

8593, 0., 6000., 8160.

8594, 0., 6000., 7620.

8595, 0., 6000., 7080.

8596, 0., 6000., 6540.

8597, 200., 9200., 8160.

8598, 200., 9200., 7620.

8599, 200., 9200., 7080.

8600, 200., 9200., 6540.

8601, 0., 9200., 6540.

8602, 0., 9200., 7080.

8603, 0., 9200., 7620.

8604, 0., 9200., 8160.

8605, 200., 9000., 6540.

8606, 200., 9000., 7080.

8607, 200., 9000., 7620.

8608, 200., 9000., 8160.

8609, 0., 9000., 8160.

8610, 0., 9000., 7620.

8611, 0., 9000., 7080.

8612, 0., 9000., 6540.

8613, 200., 9200., 540.

8614, 200., 9200., 1080.

8615, 200., 9200., 1620.

8616, 200., 9200., 2160.

8617, 200., 9000., 2160.

8618, 200., 9000., 1620.

8619, 200., 9000., 1080.

8620, 200., 9000., 540.

8621, 0., 9200., 2160.

8622, 0., 9200., 1620.

8623, 0., 9200., 1080.

8624, 0., 9200., 540.

8625, 0., 9000., 540.

8626, 0., 9000., 1080.

8627, 0., 9000., 1620.

8628, 0., 9000., 2160.

8629, 200., 12200., 8160.

8630, 200., 12200., 7620.

8631, 200., 12200., 7080.

8632, 200., 12200., 6540.

8633, 0., 12200., 6540.

8634, 0., 12200., 7080.

8635, 0., 12200., 7620.

8636, 0., 12200., 8160.

8637, 200., 12000., 6540.

8638, 200., 12000., 7080.

8639, 200., 12000., 7620.

8640, 200., 12000., 8160.

8641, 0., 12000., 8160.

8642, 0., 12000., 7620.

8643, 0., 12000., 7080.

8644, 0., 12000., 6540.

8645, 2533.33325, 9200., 2700.

8646, 2066.66675, 9200., 2700.

8647, 1600., 9200., 2700.

8648, 1133.33337, 9200., 2700.

8649, 666.666687, 9200., 2700.

8650, 666.666687, 9200., 2920.

8651, 1133.33337, 9200., 2920.

8652, 1600., 9200., 2920.

8653, 2066.66675, 9200., 2920.

8654, 2533.33325, 9200., 2920.

8655, 666.666687, 9000., 2700.

8656, 1133.33337, 9000., 2700.

8657, 1600., 9000., 2700.

8658, 2066.66675, 9000., 2700.

8659, 2533.33325, 9000., 2700.

8660, 2533.33325, 9000., 2920.

8661, 2066.66675, 9000., 2920.

8662, 1600., 9000., 2920.

8663, 1133.33337, 9000., 2920.

8664, 666.666687, 9000., 2920.

8665, 2533.33325, 12000., 2920.

8666, 2066.66675, 12000., 2920.

8667, 1600., 12000., 2920.

8668, 1133.33337, 12000., 2920.

8669, 666.666687, 12000., 2920.

8670, 666.666687, 12000., 2700.

8671, 1133.33337, 12000., 2700.

8672, 1600., 12000., 2700.

8673, 2066.66675, 12000., 2700.

8674, 2533.33325, 12000., 2700.

8675, 666.666687, 12200., 2920.

8676, 1133.33337, 12200., 2920.

8677, 1600., 12200., 2920.

8678, 2066.66675, 12200., 2920.

8679, 2533.33325, 12200., 2920.

8680, 2533.33325, 12200., 2700.

8681, 2066.66675, 12200., 2700.

8682, 1600., 12200., 2700.

8683, 1133.33337, 12200., 2700.

8684, 666.666687, 12200., 2700.

8685, 666.666687, 6200., 2920.

8686, 1133.33337, 6200., 2920.

8687, 1600., 6200., 2920.

8688, 2066.66675, 6200., 2920.

8689, 2533.33325, 6200., 2920.

8690, 2533.33325, 6200., 2700.

8691, 2066.66675, 6200., 2700.

8692, 1600., 6200., 2700.

8693, 1133.33337, 6200., 2700.

8694, 666.666687, 6200., 2700.

8695, 2533.33325, 6000., 2920.

8696, 2066.66675, 6000., 2920.

8697, 1600., 6000., 2920.

8698, 1133.33337, 6000., 2920.

8699, 666.666687, 6000., 2920.

8700, 666.666687, 6000., 2700.

8701, 1133.33337, 6000., 2700.

8702, 1600., 6000., 2700.

8703, 2066.66675, 6000., 2700.

8704, 2533.33325, 6000., 2700.

8705, 666.666687, 15200., 2920.

8706, 1133.33337, 15200., 2920.

8707, 1600., 15200., 2920.

8708, 2066.66675, 15200., 2920.

8709, 2533.33325, 15200., 2920.

8710, 2533.33325, 15000., 2920.

8711, 2066.66675, 15000., 2920.

8712, 1600., 15000., 2920.

8713, 1133.33337, 15000., 2920.

8714, 666.666687, 15000., 2920.

8715, 666.666687, 15200., 2700.

8716, 1133.33337, 15200., 2700.

8717, 1600., 15200., 2700.

8718, 2066.66675, 15200., 2700.

8719, 2533.33325, 15200., 2700.

8720, 2533.33325, 15000., 2700.

8721, 2066.66675, 15000., 2700.

8722, 1600., 15000., 2700.

8723, 1133.33337, 15000., 2700.

8724, 666.666687, 15000., 2700.

8725, 2533.33325, 0., 2920.

8726, 2066.66675, 0., 2920.

8727, 1600., 0., 2920.

8728, 1133.33337, 0., 2920.

8729, 666.666687, 0., 2920.

8730, 666.666687, 0., 2700.

8731, 1133.33337, 0., 2700.

8732, 1600., 0., 2700.

8733, 2066.66675, 0., 2700.

8734, 2533.33325, 0., 2700.

8735, 666.666687, 200., 2920.

8736, 1133.33337, 200., 2920.

8737, 1600., 200., 2920.

8738, 2066.66675, 200., 2920.

8739, 2533.33325, 200., 2920.

8740, 2533.33325, 200., 2700.

8741, 2066.66675, 200., 2700.

8742, 1600., 200., 2700.

8743, 1133.33337, 200., 2700.

8744, 666.666687, 200., 2700.

8745, 3000., 8533.33301, 2920.

8746, 3000., 8066.6665, 2920.

8747, 3000., 7600., 2920.

8748, 3000., 7133.3335, 2920.

8749, 3000., 6666.6665, 2920.

8750, 3000., 5533.3335, 2920.

8751, 3000., 5066.6665, 2920.

8752, 3000., 4600., 2920.

8753, 3000., 4133.3335, 2920.

8754, 3000., 3666.66675, 2920.

8755, 2533.33325, 3200., 2920.

8756, 2066.66675, 3200., 2920.

8757, 1600., 3200., 2920.

8758, 1133.33337, 3200., 2920.

8759, 666.666687, 3200., 2920.

8760, 2533.33325, 3000., 2920.

8761, 2066.66675, 3000., 2920.

8762, 1600., 3000., 2920.

8763, 1133.33337, 3000., 2920.

8764, 666.666687, 3000., 2920.

8765, 3000., 2533.33325, 2920.

8766, 3000., 2066.66675, 2920.

8767, 3000., 1600., 2920.

8768, 3000., 1133.33337, 2920.

8769, 3000., 666.666687, 2920.

8770, 3000., 11533.333, 2920.

8771, 3000., 11066.667, 2920.

8772, 3000., 10600., 2920.

8773, 3000., 10133.333, 2920.

8774, 3000., 9666.66699, 2920.

8775, 3000., 14533.333, 2920.

8776, 3000., 14066.667, 2920.

8777, 3000., 13600., 2920.

8778, 3000., 13133.333, 2920.

8779, 3000., 12666.667, 2920.

8780, 666.666687, 0., 3000.

8781, 1133.33337, 0., 3000.

8782, 1600., 0., 3000.

8783, 2066.66675, 0., 3000.

8784, 2533.33325, 0., 3000.

8785, 2533.33325, 15200., 3000.

8786, 2066.66675, 15200., 3000.

8787, 1600., 15200., 3000.

8788, 1133.33337, 15200., 3000.

8789, 666.666687, 15200., 3000.

8790, 3000., 666.666687, 3000.

8791, 3000., 1133.33337, 3000.

8792, 3000., 1600., 3000.

8793, 3000., 2066.66675, 3000.

8794, 3000., 2533.33325, 3000.

8795, 3000., 3666.66675, 3000.

8796, 3000., 4133.3335, 3000.

8797, 3000., 4600., 3000.

8798, 3000., 5066.6665, 3000.

8799, 3000., 5533.3335, 3000.

8800, 3000., 6666.6665, 3000.

8801, 3000., 7133.3335, 3000.

8802, 3000., 7600., 3000.

8803, 3000., 8066.6665, 3000.

8804, 3000., 8533.33301, 3000.

8805, 3000., 9666.66699, 3000.

8806, 3000., 10133.333, 3000.

8807, 3000., 10600., 3000.

8808, 3000., 11066.667, 3000.

8809, 3000., 11533.333, 3000.

8810, 3000., 12666.667, 3000.

8811, 3000., 13133.333, 3000.

8812, 3000., 13600., 3000.

8813, 3000., 14066.667, 3000.

8814, 3000., 14533.333, 3000.

8815, 2533.33325, 15000., 5920.

8816, 2066.66675, 15000., 5920.

8817, 1600., 15000., 5920.

8818, 1133.33337, 15000., 5920.

8819, 666.666687, 15000., 5920.

8820, 666.666687, 15000., 5700.

8821, 1133.33337, 15000., 5700.

8822, 1600., 15000., 5700.

8823, 2066.66675, 15000., 5700.

8824, 2533.33325, 15000., 5700.

8825, 666.666687, 15200., 5920.

8826, 1133.33337, 15200., 5920.

8827, 1600., 15200., 5920.

8828, 2066.66675, 15200., 5920.

8829, 2533.33325, 15200., 5920.

8830, 2533.33325, 15200., 5700.

8831, 2066.66675, 15200., 5700.

8832, 1600., 15200., 5700.

8833, 1133.33337, 15200., 5700.

8834, 666.666687, 15200., 5700.

8835, 666.666687, 12000., 5700.

8836, 1133.33337, 12000., 5700.

8837, 1600., 12000., 5700.

8838, 2066.66675, 12000., 5700.

8839, 2533.33325, 12000., 5700.

8840, 2533.33325, 12000., 5920.

8841, 2066.66675, 12000., 5920.

8842, 1600., 12000., 5920.

8843, 1133.33337, 12000., 5920.

8844, 666.666687, 12000., 5920.

8845, 2533.33325, 12200., 5700.

8846, 2066.66675, 12200., 5700.

8847, 1600., 12200., 5700.

8848, 1133.33337, 12200., 5700.

8849, 666.666687, 12200., 5700.

8850, 666.666687, 12200., 5920.

8851, 1133.33337, 12200., 5920.

8852, 1600., 12200., 5920.

8853, 2066.66675, 12200., 5920.

8854, 2533.33325, 12200., 5920.

8855, 2533.33325, 200., 5700.

8856, 2066.66675, 200., 5700.

8857, 1600., 200., 5700.

8858, 1133.33337, 200., 5700.

8859, 666.666687, 200., 5700.

8860, 666.666687, 200., 5920.

8861, 1133.33337, 200., 5920.

8862, 1600., 200., 5920.

8863, 2066.66675, 200., 5920.

8864, 2533.33325, 200., 5920.

8865, 666.666687, 0., 5700.

8866, 1133.33337, 0., 5700.

8867, 1600., 0., 5700.

8868, 2066.66675, 0., 5700.

8869, 2533.33325, 0., 5700.

8870, 2533.33325, 0., 5920.

8871, 2066.66675, 0., 5920.

8872, 1600., 0., 5920.

8873, 1133.33337, 0., 5920.

8874, 666.666687, 0., 5920.

8875, 666.666687, 3000., 5700.

8876, 1133.33337, 3000., 5700.

8877, 1600., 3000., 5700.

8878, 2066.66675, 3000., 5700.

8879, 2533.33325, 3000., 5700.

8880, 2533.33325, 3000., 5920.

8881, 2066.66675, 3000., 5920.

8882, 1600., 3000., 5920.

8883, 1133.33337, 3000., 5920.

8884, 666.666687, 3000., 5920.

8885, 2533.33325, 3200., 5700.

8886, 2066.66675, 3200., 5700.

8887, 1600., 3200., 5700.

8888, 1133.33337, 3200., 5700.

8889, 666.666687, 3200., 5700.

8890, 666.666687, 3200., 5920.

8891, 1133.33337, 3200., 5920.

8892, 1600., 3200., 5920.

8893, 2066.66675, 3200., 5920.

8894, 2533.33325, 3200., 5920.

8895, 666.666687, 6000., 5700.

8896, 1133.33337, 6000., 5700.

8897, 1600., 6000., 5700.

8898, 2066.66675, 6000., 5700.

8899, 2533.33325, 6000., 5700.

8900, 2533.33325, 6000., 5920.

8901, 2066.66675, 6000., 5920.

8902, 1600., 6000., 5920.

8903, 1133.33337, 6000., 5920.

8904, 666.666687, 6000., 5920.

8905, 2533.33325, 6200., 5700.

8906, 2066.66675, 6200., 5700.

8907, 1600., 6200., 5700.

8908, 1133.33337, 6200., 5700.

8909, 666.666687, 6200., 5700.

8910, 666.666687, 6200., 5920.

8911, 1133.33337, 6200., 5920.

8912, 1600., 6200., 5920.

8913, 2066.66675, 6200., 5920.

8914, 2533.33325, 6200., 5920.

8915, 666.666687, 9000., 5700.

8916, 1133.33337, 9000., 5700.

8917, 1600., 9000., 5700.

8918, 2066.66675, 9000., 5700.

8919, 2533.33325, 9000., 5700.

8920, 2533.33325, 9000., 5920.

8921, 2066.66675, 9000., 5920.

8922, 1600., 9000., 5920.

8923, 1133.33337, 9000., 5920.

8924, 666.666687, 9000., 5920.

8925, 2533.33325, 9200., 5700.

8926, 2066.66675, 9200., 5700.

8927, 1600., 9200., 5700.

8928, 1133.33337, 9200., 5700.

8929, 666.666687, 9200., 5700.

8930, 666.666687, 9200., 5920.

8931, 1133.33337, 9200., 5920.

8932, 1600., 9200., 5920.

8933, 2066.66675, 9200., 5920.

8934, 2533.33325, 9200., 5920.

8935, 2533.33325, 9000., 8920.

8936, 2066.66675, 9000., 8920.

8937, 1600., 9000., 8920.

8938, 1133.33337, 9000., 8920.

8939, 666.666687, 9000., 8920.

8940, 666.666687, 9000., 8700.

8941, 1133.33337, 9000., 8700.

8942, 1600., 9000., 8700.

8943, 2066.66675, 9000., 8700.

8944, 2533.33325, 9000., 8700.

8945, 666.666687, 9200., 8920.

8946, 1133.33337, 9200., 8920.

8947, 1600., 9200., 8920.

8948, 2066.66675, 9200., 8920.

8949, 2533.33325, 9200., 8920.

8950, 2533.33325, 9200., 8700.

8951, 2066.66675, 9200., 8700.

8952, 1600., 9200., 8700.

8953, 1133.33337, 9200., 8700.

8954, 666.666687, 9200., 8700.

8955, 666.666687, 12000., 8700.

8956, 1133.33337, 12000., 8700.

8957, 1600., 12000., 8700.

8958, 2066.66675, 12000., 8700.

8959, 2533.33325, 12000., 8700.

8960, 2533.33325, 12000., 8920.

8961, 2066.66675, 12000., 8920.

8962, 1600., 12000., 8920.

8963, 1133.33337, 12000., 8920.

8964, 666.666687, 12000., 8920.

8965, 2533.33325, 12200., 8700.

8966, 2066.66675, 12200., 8700.

8967, 1600., 12200., 8700.

8968, 1133.33337, 12200., 8700.

8969, 666.666687, 12200., 8700.

8970, 666.666687, 12200., 8920.

8971, 1133.33337, 12200., 8920.

8972, 1600., 12200., 8920.

8973, 2066.66675, 12200., 8920.

8974, 2533.33325, 12200., 8920.

8975, 2533.33325, 200., 8700.

8976, 2066.66675, 200., 8700.

8977, 1600., 200., 8700.

8978, 1133.33337, 200., 8700.

8979, 666.666687, 200., 8700.

8980, 666.666687, 200., 8920.

8981, 1133.33337, 200., 8920.

8982, 1600., 200., 8920.

8983, 2066.66675, 200., 8920.

8984, 2533.33325, 200., 8920.

8985, 666.666687, 0., 8700.

8986, 1133.33337, 0., 8700.

8987, 1600., 0., 8700.

8988, 2066.66675, 0., 8700.

8989, 2533.33325, 0., 8700.

8990, 2533.33325, 0., 8920.

8991, 2066.66675, 0., 8920.

8992, 1600., 0., 8920.

8993, 1133.33337, 0., 8920.

8994, 666.666687, 0., 8920.

8995, 666.666687, 3000., 8700.

8996, 1133.33337, 3000., 8700.

8997, 1600., 3000., 8700.

8998, 2066.66675, 3000., 8700.

8999, 2533.33325, 3000., 8700.

9000, 2533.33325, 3000., 8920.

9001, 2066.66675, 3000., 8920.

9002, 1600., 3000., 8920.

9003, 1133.33337, 3000., 8920.

9004, 666.666687, 3000., 8920.

9005, 2533.33325, 3200., 8700.

9006, 2066.66675, 3200., 8700.

9007, 1600., 3200., 8700.

9008, 1133.33337, 3200., 8700.

9009, 666.666687, 3200., 8700.

9010, 666.666687, 3200., 8920.

9011, 1133.33337, 3200., 8920.

9012, 1600., 3200., 8920.

9013, 2066.66675, 3200., 8920.

9014, 2533.33325, 3200., 8920.

9015, 666.666687, 6000., 8700.

9016, 1133.33337, 6000., 8700.

9017, 1600., 6000., 8700.

9018, 2066.66675, 6000., 8700.

9019, 2533.33325, 6000., 8700.

9020, 2533.33325, 6000., 8920.

9021, 2066.66675, 6000., 8920.

9022, 1600., 6000., 8920.

9023, 1133.33337, 6000., 8920.

9024, 666.666687, 6000., 8920.

9025, 2533.33325, 6200., 8700.

9026, 2066.66675, 6200., 8700.

9027, 1600., 6200., 8700.

9028, 1133.33337, 6200., 8700.

9029, 666.666687, 6200., 8700.

9030, 666.666687, 6200., 8920.

9031, 1133.33337, 6200., 8920.

9032, 1600., 6200., 8920.

9033, 2066.66675, 6200., 8920.

9034, 2533.33325, 6200., 8920.

9035, 3000., 6666.6665, 9000.

9036, 3000., 7133.3335, 9000.

9037, 3000., 7600., 9000.

9038, 3000., 8066.6665, 9000.

9039, 3000., 8533.33301, 9000.

9040, 3000., 8533.33301, 8920.

9041, 3000., 8066.6665, 8920.

9042, 3000., 7600., 8920.

9043, 3000., 7133.3335, 8920.

9044, 3000., 6666.6665, 8920.

9045, 3000., 3760., 9000.

9046, 3000., 4320., 9000.

9047, 3000., 4880., 9000.

9048, 3000., 5440., 9000.

9049, 3000., 5440., 8920.

9050, 3000., 4880., 8920.

9051, 3000., 4320., 8920.

9052, 3000., 3760., 8920.

9053, 3000., 666.666687, 9000.

9054, 3000., 1133.33337, 9000.

9055, 3000., 1600., 9000.

9056, 3000., 2066.66675, 9000.

9057, 3000., 2533.33325, 9000.

9058, 3000., 2533.33325, 8920.

9059, 3000., 2066.66675, 8920.

9060, 3000., 1600., 8920.

9061, 3000., 1133.33337, 8920.

9062, 3000., 666.666687, 8920.

9063, 3000., 9666.66699, 9000.

9064, 3000., 10133.333, 9000.

9065, 3000., 10600., 9000.

9066, 3000., 11066.667, 9000.

9067, 3000., 11533.333, 9000.

9068, 3000., 11533.333, 8920.

9069, 3000., 11066.667, 8920.

9070, 3000., 10600., 8920.

9071, 3000., 10133.333, 8920.

9072, 3000., 9666.66699, 8920.

9073, 3000., 12666.667, 9000.

9074, 3000., 13133.333, 9000.

9075, 3000., 13600., 9000.

9076, 3000., 14066.667, 9000.

9077, 3000., 14533.333, 9000.

9078, 3000., 14533.333, 8920.

9079, 3000., 14066.667, 8920.

9080, 3000., 13600., 8920.

9081, 3000., 13133.333, 8920.

9082, 3000., 12666.667, 8920.

9083, 200., 14765.7139, 9000.

9084, 200., 14331.4287, 9000.

9085, 200., 13897.1426, 9000.

9086, 200., 13462.8574, 9000.

9087, 200., 13028.5713, 9000.

9088, 200., 12594.2861, 9000.

9089, 200., 12160., 9000.

9090, 200., 11725.7139, 9000.

9091, 200., 11291.4287, 9000.

9092, 200., 10857.1426, 9000.

9093, 200., 10422.8574, 9000.

9094, 200., 9988.57129, 9000.

9095, 200., 9554.28613, 9000.

9096, 200., 9120., 9000.

9097, 200., 8685.71387, 9000.

9098, 200., 8251.42871, 9000.

9099, 200., 7817.14307, 9000.

9100, 200., 7382.85693, 9000.

9101, 200., 6948.57129, 9000.

9102, 200., 6514.28564, 9000.

9103, 200., 6080., 9000.

9104, 200., 5645.71436, 9000.

9105, 200., 5211.42871, 9000.

9106, 200., 4777.14307, 9000.

9107, 200., 4342.85693, 9000.

9108, 200., 3908.57153, 9000.

9109, 200., 3474.28564, 9000.

9110, 200., 3040., 9000.

9111, 200., 2605.71436, 9000.

9112, 200., 2171.42847, 9000.

9113, 200., 1737.14282, 9000.

9114, 200., 1302.85718, 9000.

9115, 200., 868.571411, 9000.

9116, 200., 434.285706, 9000.

9117, 200., 666.666687, 8920.

9118, 200., 1133.33337, 8920.

9119, 200., 1600., 8920.

9120, 200., 2066.66675, 8920.

9121, 200., 2533.33325, 8920.

9122, 200., 3760., 8920.

9123, 200., 4320., 8920.

9124, 200., 4880., 8920.

9125, 200., 5440., 8920.

9126, 200., 6666.6665, 8920.

9127, 200., 7133.3335, 8920.

9128, 200., 7600., 8920.

9129, 200., 8066.6665, 8920.

9130, 200., 8533.33301, 8920.

9131, 200., 9666.66699, 8920.

9132, 200., 10133.333, 8920.

9133, 200., 10600., 8920.

9134, 200., 11066.667, 8920.

9135, 200., 11533.333, 8920.

9136, 200., 12666.667, 8920.

9137, 200., 13133.333, 8920.

9138, 200., 13600., 8920.

9139, 200., 14066.667, 8920.

9140, 200., 14533.333, 8920.

9141, 666.666687, 15000., 8920.

9142, 1133.33337, 15000., 8920.

9143, 1600., 15000., 8920.

9144, 2066.66675, 15000., 8920.

9145, 2533.33325, 15000., 8920.

9146, 666.666687, 15200., 8920.

9147, 1133.33337, 15200., 8920.

9148, 1600., 15200., 8920.

9149, 2066.66675, 15200., 8920.

9150, 2533.33325, 15200., 8920.

9151, 2533.33325, 15200., 9000.

9152, 2066.66675, 15200., 9000.

9153, 1600., 15200., 9000.

9154, 1133.33337, 15200., 9000.

9155, 666.666687, 15200., 9000.

9156, 666.666687, 0., 9000.

9157, 1133.33337, 0., 9000.

9158, 1600., 0., 9000.

9159, 2066.66675, 0., 9000.

9160, 2533.33325, 0., 9000.

9161, 3666.66675, 12200., 8920.

9162, 4133.3335, 12200., 8920.

9163, 4600., 12200., 8920.

9164, 5066.6665, 12200., 8920.

9165, 5533.3335, 12200., 8920.

9166, 5533.3335, 12200., 8700.

9167, 5066.6665, 12200., 8700.

9168, 4600., 12200., 8700.

9169, 4133.3335, 12200., 8700.

9170, 3666.66675, 12200., 8700.

9171, 5533.3335, 12000., 8920.

9172, 5066.6665, 12000., 8920.

9173, 4600., 12000., 8920.

9174, 4133.3335, 12000., 8920.

9175, 3666.66675, 12000., 8920.

9176, 3666.66675, 12000., 8700.

9177, 4133.3335, 12000., 8700.

9178, 4600., 12000., 8700.

9179, 5066.6665, 12000., 8700.

9180, 5533.3335, 12000., 8700.

9181, 5533.3335, 9000., 8920.

9182, 5066.6665, 9000., 8920.

9183, 4600., 9000., 8920.

9184, 4133.3335, 9000., 8920.

9185, 3666.66675, 9000., 8920.

9186, 3666.66675, 9000., 8700.

9187, 4133.3335, 9000., 8700.

9188, 4600., 9000., 8700.

9189, 5066.6665, 9000., 8700.

9190, 5533.3335, 9000., 8700.

9191, 3666.66675, 9200., 8920.

9192, 4133.3335, 9200., 8920.

9193, 4600., 9200., 8920.

9194, 5066.6665, 9200., 8920.

9195, 5533.3335, 9200., 8920.

9196, 5533.3335, 9200., 8700.

9197, 5066.6665, 9200., 8700.

9198, 4600., 9200., 8700.

9199, 4133.3335, 9200., 8700.

9200, 3666.66675, 9200., 8700.

9201, 5533.3335, 6000., 8920.

9202, 5066.6665, 6000., 8920.

9203, 4600., 6000., 8920.

9204, 4133.3335, 6000., 8920.

9205, 3666.66675, 6000., 8920.

9206, 3666.66675, 6200., 8920.

9207, 4133.3335, 6200., 8920.

9208, 4600., 6200., 8920.

9209, 5066.6665, 6200., 8920.

9210, 5533.3335, 6200., 8920.

9211, 6000., 8533.33301, 8920.

9212, 6000., 8066.6665, 8920.

9213, 6000., 7600., 8920.

9214, 6000., 7133.3335, 8920.

9215, 6000., 6666.6665, 8920.

9216, 3200., 6666.6665, 8920.

9217, 3200., 7133.3335, 8920.

9218, 3200., 7600., 8920.

9219, 3200., 8066.6665, 8920.

9220, 3200., 8533.33301, 8920.

9221, 6000., 11533.333, 8920.

9222, 6000., 11066.667, 8920.

9223, 6000., 10600., 8920.

9224, 6000., 10133.333, 8920.

9225, 6000., 9666.66699, 8920.

9226, 3200., 9666.66699, 8920.

9227, 3200., 10133.333, 8920.

9228, 3200., 10600., 8920.

9229, 3200., 11066.667, 8920.

9230, 3200., 11533.333, 8920.

9231, 6000., 14533.333, 8920.

9232, 6000., 14066.667, 8920.

9233, 6000., 13600., 8920.

9234, 6000., 13133.333, 8920.

9235, 6000., 12666.667, 8920.

9236, 3200., 12666.667, 8920.

9237, 3200., 13133.333, 8920.

9238, 3200., 13600., 8920.

9239, 3200., 14066.667, 8920.

9240, 3200., 14533.333, 8920.

9241, 3666.66675, 15000., 8920.

9242, 4133.3335, 15000., 8920.

9243, 4600., 15000., 8920.

9244, 5066.6665, 15000., 8920.

9245, 5533.3335, 15000., 8920.

9246, 3666.66675, 15200., 8920.

9247, 4133.3335, 15200., 8920.

9248, 4600., 15200., 8920.

9249, 5066.6665, 15200., 8920.

9250, 5533.3335, 15200., 8920.

9251, 3666.66675, 6000., 9000.

9252, 4133.3335, 6000., 9000.

9253, 4600., 6000., 9000.

9254, 5066.6665, 6000., 9000.

9255, 5533.3335, 6000., 9000.

9256, 3200., 8533.33301, 9000.

9257, 3200., 8066.6665, 9000.

9258, 3200., 7600., 9000.

9259, 3200., 7133.3335, 9000.

9260, 3200., 6666.6665, 9000.

9261, 3200., 11533.333, 9000.

9262, 3200., 11066.667, 9000.

9263, 3200., 10600., 9000.

9264, 3200., 10133.333, 9000.

9265, 3200., 9666.66699, 9000.

9266, 3200., 14533.333, 9000.

9267, 3200., 14066.667, 9000.

9268, 3200., 13600., 9000.

9269, 3200., 13133.333, 9000.

9270, 3200., 12666.667, 9000.

9271, 5533.3335, 15200., 9000.

9272, 5066.6665, 15200., 9000.

9273, 4600., 15200., 9000.

9274, 4133.3335, 15200., 9000.

9275, 3666.66675, 15200., 9000.

9276, 6000., 6666.6665, 9000.

9277, 6000., 7133.3335, 9000.

9278, 6000., 7600., 9000.

9279, 6000., 8066.6665, 9000.

9280, 6000., 8533.33301, 9000.

9281, 6000., 9666.66699, 9000.

9282, 6000., 10133.333, 9000.

9283, 6000., 10600., 9000.

9284, 6000., 11066.667, 9000.

9285, 6000., 11533.333, 9000.

9286, 6000., 12666.667, 9000.

9287, 6000., 13133.333, 9000.

9288, 6000., 13600., 9000.

9289, 6000., 14066.667, 9000.

9290, 6000., 14533.333, 9000.

9291, 3666.66675, 6200., 8700.

9292, 4133.3335, 6200., 8700.

9293, 4600., 6200., 8700.

9294, 5066.6665, 6200., 8700.

9295, 5533.3335, 6200., 8700.

9296, 5533.3335, 6000., 8700.

9297, 5066.6665, 6000., 8700.

9298, 4600., 6000., 8700.

9299, 4133.3335, 6000., 8700.

9300, 3666.66675, 6000., 8700.

9301, 5533.3335, 0., 5920.

9302, 5066.6665, 0., 5920.

9303, 4600., 0., 5920.

9304, 4133.3335, 0., 5920.

9305, 3666.66675, 0., 5920.

9306, 3666.66675, 0., 5700.

9307, 4133.3335, 0., 5700.

9308, 4600., 0., 5700.

9309, 5066.6665, 0., 5700.

9310, 5533.3335, 0., 5700.

9311, 3666.66675, 200., 5920.

9312, 4133.3335, 200., 5920.

9313, 4600., 200., 5920.

9314, 5066.6665, 200., 5920.

9315, 5533.3335, 200., 5920.

9316, 5533.3335, 200., 5700.

9317, 5066.6665, 200., 5700.

9318, 4600., 200., 5700.

9319, 4133.3335, 200., 5700.

9320, 3666.66675, 200., 5700.

9321, 3666.66675, 3200., 5920.

9322, 4133.3335, 3200., 5920.

9323, 4600., 3200., 5920.

9324, 5066.6665, 3200., 5920.

9325, 5533.3335, 3200., 5920.

9326, 5533.3335, 3200., 5700.

9327, 5066.6665, 3200., 5700.

9328, 4600., 3200., 5700.

9329, 4133.3335, 3200., 5700.

9330, 3666.66675, 3200., 5700.

9331, 5533.3335, 3000., 5920.

9332, 5066.6665, 3000., 5920.

9333, 4600., 3000., 5920.

9334, 4133.3335, 3000., 5920.

9335, 3666.66675, 3000., 5920.

9336, 3666.66675, 3000., 5700.

9337, 4133.3335, 3000., 5700.

9338, 4600., 3000., 5700.

9339, 5066.6665, 3000., 5700.

9340, 5533.3335, 3000., 5700.

9341, 5533.3335, 12000., 5920.

9342, 5066.6665, 12000., 5920.

9343, 4600., 12000., 5920.

9344, 4133.3335, 12000., 5920.

9345, 3666.66675, 12000., 5920.

9346, 3666.66675, 12000., 5700.

9347, 4133.3335, 12000., 5700.

9348, 4600., 12000., 5700.

9349, 5066.6665, 12000., 5700.

9350, 5533.3335, 12000., 5700.

9351, 3666.66675, 12200., 5920.

9352, 4133.3335, 12200., 5920.

9353, 4600., 12200., 5920.

9354, 5066.6665, 12200., 5920.

9355, 5533.3335, 12200., 5920.

9356, 5533.3335, 12200., 5700.

9357, 5066.6665, 12200., 5700.

9358, 4600., 12200., 5700.

9359, 4133.3335, 12200., 5700.

9360, 3666.66675, 12200., 5700.

9361, 5533.3335, 15000., 5920.

9362, 5066.6665, 15000., 5920.

9363, 4600., 15000., 5920.

9364, 4133.3335, 15000., 5920.

9365, 3666.66675, 15000., 5920.

9366, 3666.66675, 15000., 5700.

9367, 4133.3335, 15000., 5700.

9368, 4600., 15000., 5700.

9369, 5066.6665, 15000., 5700.

9370, 5533.3335, 15000., 5700.

9371, 3666.66675, 15200., 5920.

9372, 4133.3335, 15200., 5920.

9373, 4600., 15200., 5920.

9374, 5066.6665, 15200., 5920.

9375, 5533.3335, 15200., 5920.

9376, 5533.3335, 15200., 5700.

9377, 5066.6665, 15200., 5700.

9378, 4600., 15200., 5700.

9379, 4133.3335, 15200., 5700.

9380, 3666.66675, 15200., 5700.

9381, 3666.66675, 9200., 5920.

9382, 4133.3335, 9200., 5920.

9383, 4600., 9200., 5920.

9384, 5066.6665, 9200., 5920.

9385, 5533.3335, 9200., 5920.

9386, 5533.3335, 9200., 5700.

9387, 5066.6665, 9200., 5700.

9388, 4600., 9200., 5700.

9389, 4133.3335, 9200., 5700.

9390, 3666.66675, 9200., 5700.

9391, 5533.3335, 9000., 5920.

9392, 5066.6665, 9000., 5920.

9393, 4600., 9000., 5920.

9394, 4133.3335, 9000., 5920.

9395, 3666.66675, 9000., 5920.

9396, 3666.66675, 9000., 5700.

9397, 4133.3335, 9000., 5700.

9398, 4600., 9000., 5700.

9399, 5066.6665, 9000., 5700.

9400, 5533.3335, 9000., 5700.

9401, 3666.66675, 6200., 5920.

9402, 4133.3335, 6200., 5920.

9403, 4600., 6200., 5920.

9404, 5066.6665, 6200., 5920.

9405, 5533.3335, 6200., 5920.

9406, 5533.3335, 6200., 5700.

9407, 5066.6665, 6200., 5700.

9408, 4600., 6200., 5700.

9409, 4133.3335, 6200., 5700.

9410, 3666.66675, 6200., 5700.

9411, 5533.3335, 6000., 5920.

9412, 5066.6665, 6000., 5920.

9413, 4600., 6000., 5920.

9414, 4133.3335, 6000., 5920.

9415, 3666.66675, 6000., 5920.

9416, 3666.66675, 6000., 5700.

9417, 4133.3335, 6000., 5700.

9418, 4600., 6000., 5700.

9419, 5066.6665, 6000., 5700.

9420, 5533.3335, 6000., 5700.

9421, 5533.3335, 0., 2920.

9422, 5066.6665, 0., 2920.

9423, 4600., 0., 2920.

9424, 4133.3335, 0., 2920.

9425, 3666.66675, 0., 2920.

9426, 3666.66675, 0., 2700.

9427, 4133.3335, 0., 2700.

9428, 4600., 0., 2700.

9429, 5066.6665, 0., 2700.

9430, 5533.3335, 0., 2700.

9431, 3666.66675, 200., 2920.

9432, 4133.3335, 200., 2920.

9433, 4600., 200., 2920.

9434, 5066.6665, 200., 2920.

9435, 5533.3335, 200., 2920.

9436, 5533.3335, 200., 2700.

9437, 5066.6665, 200., 2700.

9438, 4600., 200., 2700.

9439, 4133.3335, 200., 2700.

9440, 3666.66675, 200., 2700.

9441, 3666.66675, 3200., 2920.

9442, 4133.3335, 3200., 2920.

9443, 4600., 3200., 2920.

9444, 5066.6665, 3200., 2920.

9445, 5533.3335, 3200., 2920.

9446, 5533.3335, 3200., 2700.

9447, 5066.6665, 3200., 2700.

9448, 4600., 3200., 2700.

9449, 4133.3335, 3200., 2700.

9450, 3666.66675, 3200., 2700.

9451, 5533.3335, 3000., 2920.

9452, 5066.6665, 3000., 2920.

9453, 4600., 3000., 2920.

9454, 4133.3335, 3000., 2920.

9455, 3666.66675, 3000., 2920.

9456, 3666.66675, 3000., 2700.

9457, 4133.3335, 3000., 2700.

9458, 4600., 3000., 2700.

9459, 5066.6665, 3000., 2700.

9460, 5533.3335, 3000., 2700.

9461, 5533.3335, 9000., 2920.

9462, 5066.6665, 9000., 2920.

9463, 4600., 9000., 2920.

9464, 4133.3335, 9000., 2920.

9465, 3666.66675, 9000., 2920.

9466, 3666.66675, 9000., 2700.

9467, 4133.3335, 9000., 2700.

9468, 4600., 9000., 2700.

9469, 5066.6665, 9000., 2700.

9470, 5533.3335, 9000., 2700.

9471, 3666.66675, 9200., 2920.

9472, 4133.3335, 9200., 2920.

9473, 4600., 9200., 2920.

9474, 5066.6665, 9200., 2920.

9475, 5533.3335, 9200., 2920.

9476, 5533.3335, 9200., 2700.

9477, 5066.6665, 9200., 2700.

9478, 4600., 9200., 2700.

9479, 4133.3335, 9200., 2700.

9480, 3666.66675, 9200., 2700.

9481, 5533.3335, 12000., 2920.

9482, 5066.6665, 12000., 2920.

9483, 4600., 12000., 2920.

9484, 4133.3335, 12000., 2920.

9485, 3666.66675, 12000., 2920.

9486, 3666.66675, 12000., 2700.

9487, 4133.3335, 12000., 2700.

9488, 4600., 12000., 2700.

9489, 5066.6665, 12000., 2700.

9490, 5533.3335, 12000., 2700.

9491, 3666.66675, 12200., 2920.

9492, 4133.3335, 12200., 2920.

9493, 4600., 12200., 2920.

9494, 5066.6665, 12200., 2920.

9495, 5533.3335, 12200., 2920.

9496, 5533.3335, 12200., 2700.

9497, 5066.6665, 12200., 2700.

9498, 4600., 12200., 2700.

9499, 4133.3335, 12200., 2700.

9500, 3666.66675, 12200., 2700.

9501, 5533.3335, 15000., 2920.

9502, 5066.6665, 15000., 2920.

9503, 4600., 15000., 2920.

9504, 4133.3335, 15000., 2920.

9505, 3666.66675, 15000., 2920.

9506, 3666.66675, 15000., 2700.

9507, 4133.3335, 15000., 2700.

9508, 4600., 15000., 2700.

9509, 5066.6665, 15000., 2700.

9510, 5533.3335, 15000., 2700.

9511, 3666.66675, 15200., 2920.

9512, 4133.3335, 15200., 2920.

9513, 4600., 15200., 2920.

9514, 5066.6665, 15200., 2920.

9515, 5533.3335, 15200., 2920.

9516, 5533.3335, 15200., 2700.

9517, 5066.6665, 15200., 2700.

9518, 4600., 15200., 2700.

9519, 4133.3335, 15200., 2700.

9520, 3666.66675, 15200., 2700.

9521, 3666.66675, 6200., 2920.

9522, 4133.3335, 6200., 2920.

9523, 4600., 6200., 2920.

9524, 5066.6665, 6200., 2920.

9525, 5533.3335, 6200., 2920.

9526, 5533.3335, 6200., 2700.

9527, 5066.6665, 6200., 2700.

9528, 4600., 6200., 2700.

9529, 4133.3335, 6200., 2700.

9530, 3666.66675, 6200., 2700.

9531, 5533.3335, 6000., 2920.

9532, 5066.6665, 6000., 2920.

9533, 4600., 6000., 2920.

9534, 4133.3335, 6000., 2920.

9535, 3666.66675, 6000., 2920.

9536, 3666.66675, 6000., 2700.

9537, 4133.3335, 6000., 2700.

9538, 4600., 6000., 2700.

9539, 5066.6665, 6000., 2700.

9540, 5533.3335, 6000., 2700.

9541, 6000., 14533.333, 8700.

9542, 6000., 14066.667, 8700.

9543, 6000., 13600., 8700.

9544, 6000., 13133.333, 8700.

9545, 6000., 12666.667, 8700.

9546, 6200., 12666.667, 8700.

9547, 6200., 13133.333, 8700.

9548, 6200., 13600., 8700.

9549, 6200., 14066.667, 8700.

9550, 6200., 14533.333, 8700.

9551, 6200., 14533.333, 6000.

9552, 6200., 14066.667, 6000.

9553, 6200., 13600., 6000.

9554, 6200., 13133.333, 6000.

9555, 6200., 12666.667, 6000.

9556, 6200., 12666.667, 5920.

9557, 6200., 13133.333, 5920.

9558, 6200., 13600., 5920.

9559, 6200., 14066.667, 5920.

9560, 6200., 14533.333, 5920.

9561, 6200., 12666.667, 5700.

9562, 6200., 13133.333, 5700.

9563, 6200., 13600., 5700.

9564, 6200., 14066.667, 5700.

9565, 6200., 14533.333, 5700.

9566, 6000., 12666.667, 6000.

9567, 6000., 13133.333, 6000.

9568, 6000., 13600., 6000.

9569, 6000., 14066.667, 6000.

9570, 6000., 14533.333, 6000.

9571, 6000., 14533.333, 5920.

9572, 6000., 14066.667, 5920.

9573, 6000., 13600., 5920.

9574, 6000., 13133.333, 5920.

9575, 6000., 12666.667, 5920.

9576, 6000., 14533.333, 5700.

9577, 6000., 14066.667, 5700.

9578, 6000., 13600., 5700.

9579, 6000., 13133.333, 5700.

9580, 6000., 12666.667, 5700.

9581, 6200., 15000., 540.

9582, 6200., 15000., 1080.

9583, 6200., 15000., 1620.

9584, 6200., 15000., 2160.

9585, 6000., 15000., 2160.

9586, 6000., 15000., 1620.

9587, 6000., 15000., 1080.

9588, 6000., 15000., 540.

9589, 6000., 15000., 5160.

9590, 6000., 15000., 4620.

9591, 6000., 15000., 4080.

9592, 6000., 15000., 3540.

9593, 6200., 15000., 3540.

9594, 6200., 15000., 4080.

9595, 6200., 15000., 4620.

9596, 6200., 15000., 5160.

9597, 6000., 15000., 8160.

9598, 6000., 15000., 7620.

9599, 6000., 15000., 7080.

9600, 6000., 15000., 6540.

9601, 6200., 15000., 6540.

9602, 6200., 15000., 7080.

9603, 6200., 15000., 7620.

9604, 6200., 15000., 8160.

9605, 6200., 15200., 2160.

9606, 6200., 15200., 1620.

9607, 6200., 15200., 1080.

9608, 6200., 15200., 540.

9609, 6200., 15200., 5160.

9610, 6200., 15200., 4620.

9611, 6200., 15200., 4080.

9612, 6200., 15200., 3540.

9613, 6200., 15200., 8160.

9614, 6200., 15200., 7620.

9615, 6200., 15200., 7080.

9616, 6200., 15200., 6540.

9617, 6000., 15200., 540.

9618, 6000., 15200., 1080.

9619, 6000., 15200., 1620.

9620, 6000., 15200., 2160.

9621, 6000., 15200., 3540.

9622, 6000., 15200., 4080.

9623, 6000., 15200., 4620.

9624, 6000., 15200., 5160.

9625, 6000., 15200., 6540.

9626, 6000., 15200., 7080.

9627, 6000., 15200., 7620.

9628, 6000., 15200., 8160.

9629, 6000., 11533.333, 8700.

9630, 6000., 11066.667, 8700.

9631, 6000., 10600., 8700.

9632, 6000., 10133.333, 8700.

9633, 6000., 9666.66699, 8700.

9634, 6200., 9666.66699, 8700.

9635, 6200., 10133.333, 8700.

9636, 6200., 10600., 8700.

9637, 6200., 11066.667, 8700.

9638, 6200., 11533.333, 8700.

9639, 6000., 11533.333, 5700.

9640, 6000., 11066.667, 5700.

9641, 6000., 10600., 5700.

9642, 6000., 10133.333, 5700.

9643, 6000., 9666.66699, 5700.

9644, 6000., 9666.66699, 5920.

9645, 6000., 10133.333, 5920.

9646, 6000., 10600., 5920.

9647, 6000., 11066.667, 5920.

9648, 6000., 11533.333, 5920.

9649, 6000., 9666.66699, 6000.

9650, 6000., 10133.333, 6000.

9651, 6000., 10600., 6000.

9652, 6000., 11066.667, 6000.

9653, 6000., 11533.333, 6000.

9654, 6200., 9666.66699, 5700.

9655, 6200., 10133.333, 5700.

9656, 6200., 10600., 5700.

9657, 6200., 11066.667, 5700.

9658, 6200., 11533.333, 5700.

9659, 6200., 11533.333, 5920.

9660, 6200., 11066.667, 5920.

9661, 6200., 10600., 5920.

9662, 6200., 10133.333, 5920.

9663, 6200., 9666.66699, 5920.

9664, 6200., 11533.333, 6000.

9665, 6200., 11066.667, 6000.

9666, 6200., 10600., 6000.

9667, 6200., 10133.333, 6000.

9668, 6200., 9666.66699, 6000.

9669, 6200., 9666.66699, 2700.

9670, 6200., 10133.333, 2700.

9671, 6200., 10600., 2700.

9672, 6200., 11066.667, 2700.

9673, 6200., 11533.333, 2700.

9674, 6000., 9666.66699, 3000.

9675, 6000., 10133.333, 3000.

9676, 6000., 10600., 3000.

9677, 6000., 11066.667, 3000.

9678, 6000., 11533.333, 3000.

9679, 6000., 11533.333, 2920.

9680, 6000., 11066.667, 2920.

9681, 6000., 10600., 2920.

9682, 6000., 10133.333, 2920.

9683, 6000., 9666.66699, 2920.

9684, 6000., 11533.333, 2700.

9685, 6000., 11066.667, 2700.

9686, 6000., 10600., 2700.

9687, 6000., 10133.333, 2700.

9688, 6000., 9666.66699, 2700.

9689, 6200., 6666.6665, 2700.

9690, 6200., 7133.3335, 2700.

9691, 6200., 7600., 2700.

9692, 6200., 8066.6665, 2700.

9693, 6200., 8533.33301, 2700.

9694, 6000., 6666.6665, 3000.

9695, 6000., 7133.3335, 3000.

9696, 6000., 7600., 3000.

9697, 6000., 8066.6665, 3000.

9698, 6000., 8533.33301, 3000.

9699, 6000., 8533.33301, 2920.

9700, 6000., 8066.6665, 2920.

9701, 6000., 7600., 2920.

9702, 6000., 7133.3335, 2920.

9703, 6000., 6666.6665, 2920.

9704, 6000., 8533.33301, 2700.

9705, 6000., 8066.6665, 2700.

9706, 6000., 7600., 2700.

9707, 6000., 7133.3335, 2700.

9708, 6000., 6666.6665, 2700.

9709, 6200., 8533.33301, 5700.

9710, 6200., 8066.6665, 5700.

9711, 6200., 7600., 5700.

9712, 6200., 7133.3335, 5700.

9713, 6200., 6666.6665, 5700.

9714, 6000., 6666.6665, 5700.

9715, 6000., 7133.3335, 5700.

9716, 6000., 7600., 5700.

9717, 6000., 8066.6665, 5700.

9718, 6000., 8533.33301, 5700.

9719, 6200., 6666.6665, 6000.

9720, 6200., 7133.3335, 6000.

9721, 6200., 7600., 6000.

9722, 6200., 8066.6665, 6000.

9723, 6200., 8533.33301, 6000.

9724, 6000., 8533.33301, 6000.

9725, 6000., 8066.6665, 6000.

9726, 6000., 7600., 6000.

9727, 6000., 7133.3335, 6000.

9728, 6000., 6666.6665, 6000.

9729, 6200., 8533.33301, 5920.

9730, 6200., 8066.6665, 5920.

9731, 6200., 7600., 5920.

9732, 6200., 7133.3335, 5920.

9733, 6200., 6666.6665, 5920.

9734, 6000., 6666.6665, 5920.

9735, 6000., 7133.3335, 5920.

9736, 6000., 7600., 5920.

9737, 6000., 8066.6665, 5920.

9738, 6000., 8533.33301, 5920.

9739, 6000., 9200., 8160.

9740, 6000., 9200., 7620.

9741, 6000., 9200., 7080.

9742, 6000., 9200., 6540.

9743, 6000., 9000., 6540.

9744, 6000., 9000., 7080.

9745, 6000., 9000., 7620.

9746, 6000., 9000., 8160.

9747, 6000., 9200., 5160.

9748, 6000., 9200., 4620.

9749, 6000., 9200., 4080.

9750, 6000., 9200., 3540.

9751, 6000., 9000., 3540.

9752, 6000., 9000., 4080.

9753, 6000., 9000., 4620.

9754, 6000., 9000., 5160.

9755, 6000., 9200., 2160.

9756, 6000., 9200., 1620.

9757, 6000., 9200., 1080.

9758, 6000., 9200., 540.

9759, 6000., 9000., 540.

9760, 6000., 9000., 1080.

9761, 6000., 9000., 1620.

9762, 6000., 9000., 2160.

9763, 6200., 9000., 8160.

9764, 6200., 9000., 7620.

9765, 6200., 9000., 7080.

9766, 6200., 9000., 6540.

9767, 6200., 9200., 6540.

9768, 6200., 9200., 7080.

9769, 6200., 9200., 7620.

9770, 6200., 9200., 8160.

9771, 6200., 9000., 5160.

9772, 6200., 9000., 4620.

9773, 6200., 9000., 4080.

9774, 6200., 9000., 3540.

9775, 6200., 9200., 3540.

9776, 6200., 9200., 4080.

9777, 6200., 9200., 4620.

9778, 6200., 9200., 5160.

9779, 6200., 9200., 540.

9780, 6200., 9200., 1080.

9781, 6200., 9200., 1620.

9782, 6200., 9200., 2160.

9783, 6200., 9000., 2160.

9784, 6200., 9000., 1620.

9785, 6200., 9000., 1080.

9786, 6200., 9000., 540.

9787, 6000., 5533.3335, 5700.

9788, 6000., 5066.6665, 5700.

9789, 6000., 4600., 5700.

9790, 6000., 4133.3335, 5700.

9791, 6000., 3666.66675, 5700.

9792, 6000., 3666.66675, 5920.

9793, 6000., 4133.3335, 5920.

9794, 6000., 4600., 5920.

9795, 6000., 5066.6665, 5920.

9796, 6000., 5533.3335, 5920.

9797, 6000., 3666.66675, 6000.

9798, 6000., 4133.3335, 6000.

9799, 6000., 4600., 6000.

9800, 6000., 5066.6665, 6000.

9801, 6000., 5533.3335, 6000.

9802, 6200., 3666.66675, 5700.

9803, 6200., 4133.3335, 5700.

9804, 6200., 4600., 5700.

9805, 6200., 5066.6665, 5700.

9806, 6200., 5533.3335, 5700.

9807, 6200., 5533.3335, 5920.

9808, 6200., 5066.6665, 5920.

9809, 6200., 4600., 5920.

9810, 6200., 4133.3335, 5920.

9811, 6200., 3666.66675, 5920.

9812, 6200., 5533.3335, 6000.

9813, 6200., 5066.6665, 6000.

9814, 6200., 4600., 6000.

9815, 6200., 4133.3335, 6000.

9816, 6200., 3666.66675, 6000.

9817, 6200., 6200., 8160.

9818, 6200., 6200., 7620.

9819, 6200., 6200., 7080.

9820, 6200., 6200., 6540.

9821, 6000., 6200., 6540.

9822, 6000., 6200., 7080.

9823, 6000., 6200., 7620.

9824, 6000., 6200., 8160.

9825, 6200., 6200., 5160.

9826, 6200., 6200., 4620.

9827, 6200., 6200., 4080.

9828, 6200., 6200., 3540.

9829, 6000., 6200., 3540.

9830, 6000., 6200., 4080.

9831, 6000., 6200., 4620.

9832, 6000., 6200., 5160.

9833, 6200., 6200., 2160.

9834, 6200., 6200., 1620.

9835, 6200., 6200., 1080.

9836, 6200., 6200., 540.

9837, 6000., 6200., 540.

9838, 6000., 6200., 1080.

9839, 6000., 6200., 1620.

9840, 6000., 6200., 2160.

9841, 6200., 6000., 6540.

9842, 6200., 6000., 7080.

9843, 6200., 6000., 7620.

9844, 6200., 6000., 8160.

9845, 6000., 6000., 8160.

9846, 6000., 6000., 7620.

9847, 6000., 6000., 7080.

9848, 6000., 6000., 6540.

9849, 6000., 6000., 5160.

9850, 6000., 6000., 4620.

9851, 6000., 6000., 4080.

9852, 6000., 6000., 3540.

9853, 6200., 6000., 3540.

9854, 6200., 6000., 4080.

9855, 6200., 6000., 4620.

9856, 6200., 6000., 5160.

9857, 6200., 6000., 540.

9858, 6200., 6000., 1080.

9859, 6200., 6000., 1620.

9860, 6200., 6000., 2160.

9861, 6000., 6000., 2160.

9862, 6000., 6000., 1620.

9863, 6000., 6000., 1080.

9864, 6000., 6000., 540.

9865, 6200., 666.666687, 2700.

9866, 6200., 1133.33337, 2700.

9867, 6200., 1600., 2700.

9868, 6200., 2066.66675, 2700.

9869, 6200., 2533.33325, 2700.

9870, 6000., 666.666687, 3000.

9871, 6000., 1133.33337, 3000.

9872, 6000., 1600., 3000.

9873, 6000., 2066.66675, 3000.

9874, 6000., 2533.33325, 3000.

9875, 6000., 2533.33325, 2920.

9876, 6000., 2066.66675, 2920.

9877, 6000., 1600., 2920.

9878, 6000., 1133.33337, 2920.

9879, 6000., 666.666687, 2920.

9880, 6000., 2533.33325, 2700.

9881, 6000., 2066.66675, 2700.

9882, 6000., 1600., 2700.

9883, 6000., 1133.33337, 2700.

9884, 6000., 666.666687, 2700.

9885, 6200., 3000., 5160.

9886, 6200., 3000., 4620.

9887, 6200., 3000., 4080.

9888, 6200., 3000., 3540.

9889, 6200., 3200., 3540.

9890, 6200., 3200., 4080.

9891, 6200., 3200., 4620.

9892, 6200., 3200., 5160.

9893, 6200., 3200., 540.

9894, 6200., 3200., 1080.

9895, 6200., 3200., 1620.

9896, 6200., 3200., 2160.

9897, 6200., 3000., 2160.

9898, 6200., 3000., 1620.

9899, 6200., 3000., 1080.

9900, 6200., 3000., 540.

9901, 6000., 3200., 5160.

9902, 6000., 3200., 4620.

9903, 6000., 3200., 4080.

9904, 6000., 3200., 3540.

9905, 6000., 3000., 3540.

9906, 6000., 3000., 4080.

9907, 6000., 3000., 4620.

9908, 6000., 3000., 5160.

9909, 6000., 3200., 2160.

9910, 6000., 3200., 1620.

9911, 6000., 3200., 1080.

9912, 6000., 3200., 540.

9913, 6000., 3000., 540.

9914, 6000., 3000., 1080.

9915, 6000., 3000., 1620.

9916, 6000., 3000., 2160.

9917, 6200., 12000., 8160.

9918, 6200., 12000., 7620.

9919, 6200., 12000., 7080.

9920, 6200., 12000., 6540.

9921, 6200., 12200., 6540.

9922, 6200., 12200., 7080.

9923, 6200., 12200., 7620.

9924, 6200., 12200., 8160.

9925, 6200., 12000., 5160.

9926, 6200., 12000., 4620.

9927, 6200., 12000., 4080.

9928, 6200., 12000., 3540.

9929, 6200., 12200., 3540.

9930, 6200., 12200., 4080.

9931, 6200., 12200., 4620.

9932, 6200., 12200., 5160.

9933, 6200., 12200., 540.

9934, 6200., 12200., 1080.

9935, 6200., 12200., 1620.

9936, 6200., 12200., 2160.

9937, 6200., 12000., 2160.

9938, 6200., 12000., 1620.

9939, 6200., 12000., 1080.

9940, 6200., 12000., 540.

9941, 6000., 12200., 8160.

9942, 6000., 12200., 7620.

9943, 6000., 12200., 7080.

9944, 6000., 12200., 6540.

9945, 6000., 12000., 6540.

9946, 6000., 12000., 7080.

9947, 6000., 12000., 7620.

9948, 6000., 12000., 8160.

9949, 6000., 12200., 5160.

9950, 6000., 12200., 4620.

9951, 6000., 12200., 4080.

9952, 6000., 12200., 3540.

9953, 6000., 12000., 3540.

9954, 6000., 12000., 4080.

9955, 6000., 12000., 4620.

9956, 6000., 12000., 5160.

9957, 6000., 12200., 2160.

9958, 6000., 12200., 1620.

9959, 6000., 12200., 1080.

9960, 6000., 12200., 540.

9961, 6000., 12000., 540.

9962, 6000., 12000., 1080.

9963, 6000., 12000., 1620.

9964, 6000., 12000., 2160.

9965, 6000., 8533.33301, 8700.

9966, 6000., 8066.6665, 8700.

9967, 6000., 7600., 8700.

9968, 6000., 7133.3335, 8700.

9969, 6000., 6666.6665, 8700.

9970, 6200., 6666.6665, 8700.

9971, 6200., 7133.3335, 8700.

9972, 6200., 7600., 8700.

9973, 6200., 8066.6665, 8700.

9974, 6200., 8533.33301, 8700.

9975, 6000., 5533.3335, 2920.

9976, 6000., 5066.6665, 2920.

9977, 6000., 4600., 2920.

9978, 6000., 4133.3335, 2920.

9979, 6000., 3666.66675, 2920.

9980, 6000., 3666.66675, 3000.

9981, 6000., 4133.3335, 3000.

9982, 6000., 4600., 3000.

9983, 6000., 5066.6665, 3000.

9984, 6000., 5533.3335, 3000.

9985, 6000., 5533.3335, 2700.

9986, 6000., 5066.6665, 2700.

9987, 6000., 4600., 2700.

9988, 6000., 4133.3335, 2700.

9989, 6000., 3666.66675, 2700.

9990, 6200., 3666.66675, 2700.

9991, 6200., 4133.3335, 2700.

9992, 6200., 4600., 2700.

9993, 6200., 5066.6665, 2700.

9994, 6200., 5533.3335, 2700.

9995, 6000., 2533.33325, 5920.

9996, 6000., 2066.66675, 5920.

9997, 6000., 1600., 5920.

9998, 6000., 1133.33337, 5920.

9999, 6000., 666.666687, 5920.

10000, 6000., 666.666687, 6000.

10001, 6000., 1133.33337, 6000.

10002, 6000., 1600., 6000.

10003, 6000., 2066.66675, 6000.

10004, 6000., 2533.33325, 6000.

10005, 6000., 2533.33325, 5700.

10006, 6000., 2066.66675, 5700.

10007, 6000., 1600., 5700.

10008, 6000., 1133.33337, 5700.

10009, 6000., 666.666687, 5700.

10010, 6200., 666.666687, 5920.

10011, 6200., 1133.33337, 5920.

10012, 6200., 1600., 5920.

10013, 6200., 2066.66675, 5920.

10014, 6200., 2533.33325, 5920.

10015, 6200., 2533.33325, 6000.

10016, 6200., 2066.66675, 6000.

10017, 6200., 1600., 6000.

10018, 6200., 1133.33337, 6000.

10019, 6200., 666.666687, 6000.

10020, 6200., 666.666687, 5700.

10021, 6200., 1133.33337, 5700.

10022, 6200., 1600., 5700.

10023, 6200., 2066.66675, 5700.

10024, 6200., 2533.33325, 5700.

10025, 6000., 200., 540.

10026, 6000., 200., 1080.

10027, 6000., 200., 1620.

10028, 6000., 200., 2160.

10029, 6200., 200., 2160.

10030, 6200., 200., 1620.

10031, 6200., 200., 1080.

10032, 6200., 200., 540.

10033, 6200., 200., 5160.

10034, 6200., 200., 4620.

10035, 6200., 200., 4080.

10036, 6200., 200., 3540.

10037, 6000., 200., 3540.

10038, 6000., 200., 4080.

10039, 6000., 200., 4620.

10040, 6000., 200., 5160.

10041, 6000., 0., 2160.

10042, 6000., 0., 1620.

10043, 6000., 0., 1080.

10044, 6000., 0., 540.

10045, 6000., 0., 5160.

10046, 6000., 0., 4620.

10047, 6000., 0., 4080.

10048, 6000., 0., 3540.

10049, 6200., 0., 540.

10050, 6200., 0., 1080.

10051, 6200., 0., 1620.

10052, 6200., 0., 2160.

10053, 6200., 0., 3540.

10054, 6200., 0., 4080.

10055, 6200., 0., 4620.

10056, 6200., 0., 5160.

10057, 3200., 14533.333, 2700.

10058, 3200., 14066.667, 2700.

10059, 3200., 13600., 2700.

10060, 3200., 13133.333, 2700.

10061, 3200., 12666.667, 2700.

10062, 3000., 12666.667, 2700.

10063, 3000., 13133.333, 2700.

10064, 3000., 13600., 2700.

10065, 3000., 14066.667, 2700.

10066, 3000., 14533.333, 2700.

10067, 3200., 12666.667, 3000.

10068, 3200., 13133.333, 3000.

10069, 3200., 13600., 3000.

10070, 3200., 14066.667, 3000.

10071, 3200., 14533.333, 3000.

10072, 3200., 14533.333, 2920.

10073, 3200., 14066.667, 2920.

10074, 3200., 13600., 2920.

10075, 3200., 13133.333, 2920.

10076, 3200., 12666.667, 2920.

10077, 3000., 12666.667, 6000.

10078, 3000., 13133.333, 6000.

10079, 3000., 13600., 6000.

10080, 3000., 14066.667, 6000.

10081, 3000., 14533.333, 6000.

10082, 3000., 14533.333, 5920.

10083, 3000., 14066.667, 5920.

10084, 3000., 13600., 5920.

10085, 3000., 13133.333, 5920.

10086, 3000., 12666.667, 5920.

10087, 3000., 14533.333, 5700.

10088, 3000., 14066.667, 5700.

10089, 3000., 13600., 5700.

10090, 3000., 13133.333, 5700.

10091, 3000., 12666.667, 5700.

10092, 3200., 14533.333, 6000.

10093, 3200., 14066.667, 6000.

10094, 3200., 13600., 6000.

10095, 3200., 13133.333, 6000.

10096, 3200., 12666.667, 6000.

10097, 3200., 12666.667, 5920.

10098, 3200., 13133.333, 5920.

10099, 3200., 13600., 5920.

10100, 3200., 14066.667, 5920.

10101, 3200., 14533.333, 5920.

10102, 3200., 12666.667, 5700.

10103, 3200., 13133.333, 5700.

10104, 3200., 13600., 5700.

10105, 3200., 14066.667, 5700.

10106, 3200., 14533.333, 5700.

10107, 3200., 12000., 8160.

10108, 3200., 12000., 7620.

10109, 3200., 12000., 7080.

10110, 3200., 12000., 6540.

10111, 3200., 12200., 6540.

10112, 3200., 12200., 7080.

10113, 3200., 12200., 7620.

10114, 3200., 12200., 8160.

10115, 3200., 12000., 5160.

10116, 3200., 12000., 4620.

10117, 3200., 12000., 4080.

10118, 3200., 12000., 3540.

10119, 3200., 12200., 3540.

10120, 3200., 12200., 4080.

10121, 3200., 12200., 4620.

10122, 3200., 12200., 5160.

10123, 3200., 12000., 2160.

10124, 3200., 12000., 1620.

10125, 3200., 12000., 1080.

10126, 3200., 12000., 540.

10127, 3200., 12200., 540.

10128, 3200., 12200., 1080.

10129, 3200., 12200., 1620.

10130, 3200., 12200., 2160.

10131, 3000., 12200., 8160.

10132, 3000., 12200., 7620.

10133, 3000., 12200., 7080.

10134, 3000., 12200., 6540.

10135, 3000., 12000., 6540.

10136, 3000., 12000., 7080.

10137, 3000., 12000., 7620.

10138, 3000., 12000., 8160.

10139, 3000., 12200., 5160.

10140, 3000., 12200., 4620.

10141, 3000., 12200., 4080.

10142, 3000., 12200., 3540.

10143, 3000., 12000., 3540.

10144, 3000., 12000., 4080.

10145, 3000., 12000., 4620.

10146, 3000., 12000., 5160.

10147, 3000., 12200., 2160.

10148, 3000., 12200., 1620.

10149, 3000., 12200., 1080.

10150, 3000., 12200., 540.

10151, 3000., 12000., 540.

10152, 3000., 12000., 1080.

10153, 3000., 12000., 1620.

10154, 3000., 12000., 2160.

10155, 3000., 9666.66699, 2700.

10156, 3000., 10133.333, 2700.

10157, 3000., 10600., 2700.

10158, 3000., 11066.667, 2700.

10159, 3000., 11533.333, 2700.

10160, 3200., 11533.333, 2700.

10161, 3200., 11066.667, 2700.

10162, 3200., 10600., 2700.

10163, 3200., 10133.333, 2700.

10164, 3200., 9666.66699, 2700.

10165, 3200., 9666.66699, 3000.

10166, 3200., 10133.333, 3000.

10167, 3200., 10600., 3000.

10168, 3200., 11066.667, 3000.

10169, 3200., 11533.333, 3000.

10170, 3200., 11533.333, 2920.

10171, 3200., 11066.667, 2920.

10172, 3200., 10600., 2920.

10173, 3200., 10133.333, 2920.

10174, 3200., 9666.66699, 2920.

10175, 3000., 9666.66699, 6000.

10176, 3000., 10133.333, 6000.

10177, 3000., 10600., 6000.

10178, 3000., 11066.667, 6000.

10179, 3000., 11533.333, 6000.

10180, 3000., 11533.333, 5920.

10181, 3000., 11066.667, 5920.

10182, 3000., 10600., 5920.

10183, 3000., 10133.333, 5920.

10184, 3000., 9666.66699, 5920.

10185, 3000., 11533.333, 5700.

10186, 3000., 11066.667, 5700.

10187, 3000., 10600., 5700.

10188, 3000., 10133.333, 5700.

10189, 3000., 9666.66699, 5700.

10190, 3200., 11533.333, 6000.

10191, 3200., 11066.667, 6000.

10192, 3200., 10600., 6000.

10193, 3200., 10133.333, 6000.

10194, 3200., 9666.66699, 6000.

10195, 3200., 9666.66699, 5920.

10196, 3200., 10133.333, 5920.

10197, 3200., 10600., 5920.

10198, 3200., 11066.667, 5920.

10199, 3200., 11533.333, 5920.

10200, 3200., 9666.66699, 5700.

10201, 3200., 10133.333, 5700.

10202, 3200., 10600., 5700.

10203, 3200., 11066.667, 5700.

10204, 3200., 11533.333, 5700.

10205, 3200., 9000., 8160.

10206, 3200., 9000., 7620.

10207, 3200., 9000., 7080.

10208, 3200., 9000., 6540.

10209, 3200., 9200., 6540.

10210, 3200., 9200., 7080.

10211, 3200., 9200., 7620.

10212, 3200., 9200., 8160.

10213, 3200., 9000., 5160.

10214, 3200., 9000., 4620.

10215, 3200., 9000., 4080.

10216, 3200., 9000., 3540.

10217, 3200., 9200., 3540.

10218, 3200., 9200., 4080.

10219, 3200., 9200., 4620.

10220, 3200., 9200., 5160.

10221, 3200., 9000., 2160.

10222, 3200., 9000., 1620.

10223, 3200., 9000., 1080.

10224, 3200., 9000., 540.

10225, 3200., 9200., 540.

10226, 3200., 9200., 1080.

10227, 3200., 9200., 1620.

10228, 3200., 9200., 2160.

10229, 3000., 9200., 8160.

10230, 3000., 9200., 7620.

10231, 3000., 9200., 7080.

10232, 3000., 9200., 6540.

10233, 3000., 9000., 6540.

10234, 3000., 9000., 7080.

10235, 3000., 9000., 7620.

10236, 3000., 9000., 8160.

10237, 3000., 9200., 5160.

10238, 3000., 9200., 4620.

10239, 3000., 9200., 4080.

10240, 3000., 9200., 3540.

10241, 3000., 9000., 3540.

10242, 3000., 9000., 4080.

10243, 3000., 9000., 4620.

10244, 3000., 9000., 5160.

10245, 3000., 9200., 2160.

10246, 3000., 9200., 1620.

10247, 3000., 9200., 1080.

10248, 3000., 9200., 540.

10249, 3000., 9000., 540.

10250, 3000., 9000., 1080.

10251, 3000., 9000., 1620.

10252, 3000., 9000., 2160.

10253, 3000., 8533.33301, 8700.

10254, 3000., 8066.6665, 8700.

10255, 3000., 7600., 8700.

10256, 3000., 7133.3335, 8700.

10257, 3000., 6666.6665, 8700.

10258, 3200., 6666.6665, 8700.

10259, 3200., 7133.3335, 8700.

10260, 3200., 7600., 8700.

10261, 3200., 8066.6665, 8700.

10262, 3200., 8533.33301, 8700.

10263, 3200., 6666.6665, 5700.

10264, 3200., 7133.3335, 5700.

10265, 3200., 7600., 5700.

10266, 3200., 8066.6665, 5700.

10267, 3200., 8533.33301, 5700.

10268, 3200., 8533.33301, 5920.

10269, 3200., 8066.6665, 5920.

10270, 3200., 7600., 5920.

10271, 3200., 7133.3335, 5920.

10272, 3200., 6666.6665, 5920.

10273, 3200., 8533.33301, 6000.

10274, 3200., 8066.6665, 6000.

10275, 3200., 7600., 6000.

10276, 3200., 7133.3335, 6000.

10277, 3200., 6666.6665, 6000.

10278, 3000., 8533.33301, 5700.

10279, 3000., 8066.6665, 5700.

10280, 3000., 7600., 5700.

10281, 3000., 7133.3335, 5700.

10282, 3000., 6666.6665, 5700.

10283, 3000., 6666.6665, 5920.

10284, 3000., 7133.3335, 5920.

10285, 3000., 7600., 5920.

10286, 3000., 8066.6665, 5920.

10287, 3000., 8533.33301, 5920.

10288, 3000., 6666.6665, 6000.

10289, 3000., 7133.3335, 6000.

10290, 3000., 7600., 6000.

10291, 3000., 8066.6665, 6000.

10292, 3000., 8533.33301, 6000.

10293, 3000., 8533.33301, 2700.

10294, 3000., 8066.6665, 2700.

10295, 3000., 7600., 2700.

10296, 3000., 7133.3335, 2700.

10297, 3000., 6666.6665, 2700.

10298, 3200., 8533.33301, 3000.

10299, 3200., 8066.6665, 3000.

10300, 3200., 7600., 3000.

10301, 3200., 7133.3335, 3000.

10302, 3200., 6666.6665, 3000.

10303, 3200., 6666.6665, 2920.

10304, 3200., 7133.3335, 2920.

10305, 3200., 7600., 2920.

10306, 3200., 8066.6665, 2920.

10307, 3200., 8533.33301, 2920.

10308, 3200., 6666.6665, 2700.

10309, 3200., 7133.3335, 2700.

10310, 3200., 7600., 2700.

10311, 3200., 8066.6665, 2700.

10312, 3200., 8533.33301, 2700.

10313, 3200., 3200., 8850.

10314, 3200., 3760., 8700.

10315, 3200., 4320., 8700.

10316, 3200., 4880., 8700.

10317, 3200., 5440., 8700.

10318, 3200., 5440., 9000.

10319, 3200., 4880., 9000.

10320, 3200., 4320., 9000.

10321, 3200., 3760., 9000.

10322, 3000., 5440., 8700.

10323, 3000., 4880., 8700.

10324, 3000., 4320., 8700.

10325, 3000., 3760., 8700.

10326, 3000., 3666.66675, 6000.

10327, 3000., 4133.3335, 6000.

10328, 3000., 4600., 6000.

10329, 3000., 5066.6665, 6000.

10330, 3000., 5533.3335, 6000.

10331, 3000., 5533.3335, 5920.

10332, 3000., 5066.6665, 5920.

10333, 3000., 4600., 5920.

10334, 3000., 4133.3335, 5920.

10335, 3000., 3666.66675, 5920.

10336, 3000., 5533.3335, 5700.

10337, 3000., 5066.6665, 5700.

10338, 3000., 4600., 5700.

10339, 3000., 4133.3335, 5700.

10340, 3000., 3666.66675, 5700.

10341, 3200., 5533.3335, 6000.

10342, 3200., 5066.6665, 6000.

10343, 3200., 4600., 6000.

10344, 3200., 4133.3335, 6000.

10345, 3200., 3666.66675, 6000.

10346, 3200., 3666.66675, 5920.

10347, 3200., 4133.3335, 5920.

10348, 3200., 4600., 5920.

10349, 3200., 5066.6665, 5920.

10350, 3200., 5533.3335, 5920.

10351, 3200., 3666.66675, 5700.

10352, 3200., 4133.3335, 5700.

10353, 3200., 4600., 5700.

10354, 3200., 5066.6665, 5700.

10355, 3200., 5533.3335, 5700.

10356, 3000., 5533.3335, 2700.

10357, 3000., 5066.6665, 2700.

10358, 3000., 4600., 2700.

10359, 3000., 4133.3335, 2700.

10360, 3000., 3666.66675, 2700.

10361, 3200., 5533.3335, 3000.

10362, 3200., 5066.6665, 3000.

10363, 3200., 4600., 3000.

10364, 3200., 4133.3335, 3000.

10365, 3200., 3666.66675, 3000.

10366, 3200., 3666.66675, 2920.

10367, 3200., 4133.3335, 2920.

10368, 3200., 4600., 2920.

10369, 3200., 5066.6665, 2920.

10370, 3200., 5533.3335, 2920.

10371, 3200., 3666.66675, 2700.

10372, 3200., 4133.3335, 2700.

10373, 3200., 4600., 2700.

10374, 3200., 5066.6665, 2700.

10375, 3200., 5533.3335, 2700.

10376, 3200., 666.666687, 5920.

10377, 3200., 1133.33337, 5920.

10378, 3200., 1600., 5920.

10379, 3200., 2066.66675, 5920.

10380, 3200., 2533.33325, 5920.

10381, 3200., 2533.33325, 6000.

10382, 3200., 2066.66675, 6000.

10383, 3200., 1600., 6000.

10384, 3200., 1133.33337, 6000.

10385, 3200., 666.666687, 6000.

10386, 3200., 666.666687, 5700.

10387, 3200., 1133.33337, 5700.

10388, 3200., 1600., 5700.

10389, 3200., 2066.66675, 5700.

10390, 3200., 2533.33325, 5700.

10391, 3000., 2533.33325, 5920.

10392, 3000., 2066.66675, 5920.

10393, 3000., 1600., 5920.

10394, 3000., 1133.33337, 5920.

10395, 3000., 666.666687, 5920.

10396, 3000., 666.666687, 6000.

10397, 3000., 1133.33337, 6000.

10398, 3000., 1600., 6000.

10399, 3000., 2066.66675, 6000.

10400, 3000., 2533.33325, 6000.

10401, 3000., 2533.33325, 5700.

10402, 3000., 2066.66675, 5700.

10403, 3000., 1600., 5700.

10404, 3000., 1133.33337, 5700.

10405, 3000., 666.666687, 5700.

10406, 3000., 2533.33325, 8700.

10407, 3000., 2066.66675, 8700.

10408, 3000., 1600., 8700.

10409, 3000., 1133.33337, 8700.

10410, 3000., 666.666687, 8700.

10411, 3200., 2533.33325, 9000.

10412, 3200., 2066.66675, 9000.

10413, 3200., 1600., 9000.

10414, 3200., 1133.33337, 9000.

10415, 3200., 666.666687, 9000.

10416, 3200., 3000., 8850.

10417, 3200., 666.666687, 8700.

10418, 3200., 1133.33337, 8700.

10419, 3200., 1600., 8700.

10420, 3200., 2066.66675, 8700.

10421, 3200., 2533.33325, 8700.

10422, 3200., 200., 8850.

10423, 3200., 200., 8160.

10424, 3200., 200., 7620.

10425, 3200., 200., 7080.

10426, 3200., 200., 6540.

10427, 3000., 200., 6540.

10428, 3000., 200., 7080.

10429, 3000., 200., 7620.

10430, 3000., 200., 8160.

10431, 3200., 200., 5160.

10432, 3200., 200., 4620.

10433, 3200., 200., 4080.

10434, 3200., 200., 3540.

10435, 3000., 200., 3540.

10436, 3000., 200., 4080.

10437, 3000., 200., 4620.

10438, 3000., 200., 5160.

10439, 3000., 200., 540.

10440, 3000., 200., 1080.

10441, 3000., 200., 1620.

10442, 3000., 200., 2160.

10443, 3200., 200., 2160.

10444, 3200., 200., 1620.

10445, 3200., 200., 1080.

10446, 3200., 200., 540.

10447, 3200., 0., 6428.57129

10448, 3200., 0., 6857.14307

10449, 3200., 0., 7285.71436

10450, 3200., 0., 7714.28564

10451, 3200., 0., 8142.85693

10452, 3200., 0., 8571.42871

10453, 3200., 0., 3540.

10454, 3200., 0., 4080.

10455, 3200., 0., 4620.

10456, 3200., 0., 5160.

10457, 3200., 0., 540.

10458, 3200., 0., 1080.

10459, 3200., 0., 1620.

10460, 3200., 0., 2160.

10461, 3000., 0., 8160.

10462, 3000., 0., 7620.

10463, 3000., 0., 7080.

10464, 3000., 0., 6540.

10465, 3000., 0., 5160.

10466, 3000., 0., 4620.

10467, 3000., 0., 4080.

10468, 3000., 0., 3540.

10469, 3000., 0., 2160.

10470, 3000., 0., 1620.

10471, 3000., 0., 1080.

10472, 3000., 0., 540.

10473, 3200., 3200., 6540.

10474, 3200., 3200., 7080.

10475, 3200., 3200., 7620.

10476, 3200., 3200., 8160.

10477, 3200., 3000., 8160.

10478, 3200., 3000., 7620.

10479, 3200., 3000., 7080.

10480, 3200., 3000., 6540.

10481, 3200., 3000., 5160.

10482, 3200., 3000., 4620.

10483, 3200., 3000., 4080.

10484, 3200., 3000., 3540.

10485, 3200., 3200., 3540.

10486, 3200., 3200., 4080.

10487, 3200., 3200., 4620.

10488, 3200., 3200., 5160.

10489, 3200., 3200., 540.

10490, 3200., 3200., 1080.

10491, 3200., 3200., 1620.

10492, 3200., 3200., 2160.

10493, 3200., 3000., 2160.

10494, 3200., 3000., 1620.

10495, 3200., 3000., 1080.

10496, 3200., 3000., 540.

10497, 3000., 3200., 8160.

10498, 3000., 3200., 7620.

10499, 3000., 3200., 7080.

10500, 3000., 3200., 6540.

10501, 3000., 3000., 6540.

10502, 3000., 3000., 7080.

10503, 3000., 3000., 7620.

10504, 3000., 3000., 8160.

10505, 3000., 3200., 5160.

10506, 3000., 3200., 4620.

10507, 3000., 3200., 4080.

10508, 3000., 3200., 3540.

10509, 3000., 3000., 3540.

10510, 3000., 3000., 4080.

10511, 3000., 3000., 4620.

10512, 3000., 3000., 5160.

10513, 3000., 3200., 2160.

10514, 3000., 3200., 1620.

10515, 3000., 3200., 1080.

10516, 3000., 3200., 540.

10517, 3000., 3000., 540.

10518, 3000., 3000., 1080.

10519, 3000., 3000., 1620.

10520, 3000., 3000., 2160.

10521, 3200., 6000., 8160.

10522, 3200., 6000., 7620.

10523, 3200., 6000., 7080.

10524, 3200., 6000., 6540.

10525, 3200., 6200., 6540.

10526, 3200., 6200., 7080.

10527, 3200., 6200., 7620.

10528, 3200., 6200., 8160.

10529, 3200., 6000., 5160.

10530, 3200., 6000., 4620.

10531, 3200., 6000., 4080.

10532, 3200., 6000., 3540.

10533, 3200., 6200., 3540.

10534, 3200., 6200., 4080.

10535, 3200., 6200., 4620.

10536, 3200., 6200., 5160.

10537, 3200., 6200., 540.

10538, 3200., 6200., 1080.

10539, 3200., 6200., 1620.

10540, 3200., 6200., 2160.

10541, 3200., 6000., 2160.

10542, 3200., 6000., 1620.

10543, 3200., 6000., 1080.

10544, 3200., 6000., 540.

10545, 3000., 6200., 8160.

10546, 3000., 6200., 7620.

10547, 3000., 6200., 7080.

10548, 3000., 6200., 6540.

10549, 3000., 6000., 6540.

10550, 3000., 6000., 7080.

10551, 3000., 6000., 7620.

10552, 3000., 6000., 8160.

10553, 3000., 6200., 5160.

10554, 3000., 6200., 4620.

10555, 3000., 6200., 4080.

10556, 3000., 6200., 3540.

10557, 3000., 6000., 3540.

10558, 3000., 6000., 4080.

10559, 3000., 6000., 4620.

10560, 3000., 6000., 5160.

10561, 3000., 6200., 2160.

10562, 3000., 6200., 1620.

10563, 3000., 6200., 1080.

10564, 3000., 6200., 540.

10565, 3000., 6000., 540.

10566, 3000., 6000., 1080.

10567, 3000., 6000., 1620.

10568, 3000., 6000., 2160.

10569, 3200., 9666.66699, 8700.

10570, 3200., 10133.333, 8700.

10571, 3200., 10600., 8700.

10572, 3200., 11066.667, 8700.

10573, 3200., 11533.333, 8700.

10574, 3000., 11533.333, 8700.

10575, 3000., 11066.667, 8700.

10576, 3000., 10600., 8700.

10577, 3000., 10133.333, 8700.

10578, 3000., 9666.66699, 8700.

10579, 3200., 12666.667, 8700.

10580, 3200., 13133.333, 8700.

10581, 3200., 13600., 8700.

10582, 3200., 14066.667, 8700.

10583, 3200., 14533.333, 8700.

10584, 3000., 14533.333, 8700.

10585, 3000., 14066.667, 8700.

10586, 3000., 13600., 8700.

10587, 3000., 13133.333, 8700.

10588, 3000., 12666.667, 8700.

10589, 3000., 15000., 8160.

10590, 3000., 15000., 7620.

10591, 3000., 15000., 7080.

10592, 3000., 15000., 6540.

10593, 3200., 15000., 6540.

10594, 3200., 15000., 7080.

10595, 3200., 15000., 7620.

10596, 3200., 15000., 8160.

10597, 3000., 15000., 5160.

10598, 3000., 15000., 4620.

10599, 3000., 15000., 4080.

10600, 3000., 15000., 3540.

10601, 3200., 15000., 3540.

10602, 3200., 15000., 4080.

10603, 3200., 15000., 4620.

10604, 3200., 15000., 5160.

10605, 3200., 15000., 540.

10606, 3200., 15000., 1080.

10607, 3200., 15000., 1620.

10608, 3200., 15000., 2160.

10609, 3000., 15000., 2160.

10610, 3000., 15000., 1620.

10611, 3000., 15000., 1080.

10612, 3000., 15000., 540.

10613, 3000., 15200., 6540.

10614, 3000., 15200., 7080.

10615, 3000., 15200., 7620.

10616, 3000., 15200., 8160.

10617, 3000., 15200., 3540.

10618, 3000., 15200., 4080.

10619, 3000., 15200., 4620.

10620, 3000., 15200., 5160.

10621, 3000., 15200., 540.

10622, 3000., 15200., 1080.

10623, 3000., 15200., 1620.

10624, 3000., 15200., 2160.

10625, 3200., 15200., 8160.

10626, 3200., 15200., 7620.

10627, 3200., 15200., 7080.

10628, 3200., 15200., 6540.

10629, 3200., 15200., 5160.

10630, 3200., 15200., 4620.

10631, 3200., 15200., 4080.

10632, 3200., 15200., 3540.

10633, 3200., 15200., 2160.

10634, 3200., 15200., 1620.

10635, 3200., 15200., 1080.

10636, 3200., 15200., 540.

10637, 23533.334, 15200., 6000.

10638, 23066.666, 15200., 6000.

10639, 22600., 15200., 6000.

10640, 22133.334, 15200., 6000.

10641, 21666.666, 15200., 6000.

10642, 21666.666, 0., 6000.

10643, 22133.334, 0., 6000.

10644, 22600., 0., 6000.

10645, 23066.666, 0., 6000.

10646, 23533.334, 0., 6000.

10647, 24000., 3200., 2160.

10648, 24000., 3200., 1620.

10649, 24000., 3200., 1080.

10650, 24000., 3200., 540.

10651, 24000., 3000., 540.

10652, 24000., 3000., 1080.

10653, 24000., 3000., 1620.

10654, 24000., 3000., 2160.

10655, 24200., 3200., 540.

10656, 24200., 3200., 1080.

10657, 24200., 3200., 1620.

10658, 24200., 3200., 2160.

10659, 24200., 3000., 2160.

10660, 24200., 3000., 1620.

10661, 24200., 3000., 1080.

10662, 24200., 3000., 540.

10663, 23533.334, 200., 2700.

10664, 23066.666, 200., 2700.

10665, 22600., 200., 2700.

10666, 22133.334, 200., 2700.

10667, 21666.666, 200., 2700.

10668, 21666.666, 0., 2700.

10669, 22133.334, 0., 2700.

10670, 22600., 0., 2700.

10671, 23066.666, 0., 2700.

10672, 23533.334, 0., 2700.

10673, 23533.334, 12200., 8700.

10674, 23066.666, 12200., 8700.

10675, 22600., 12200., 8700.

10676, 22133.334, 12200., 8700.

10677, 21666.666, 12200., 8700.

10678, 21666.666, 12000., 8700.

10679, 22133.334, 12000., 8700.

10680, 22600., 12000., 8700.

10681, 23066.666, 12000., 8700.

10682, 23533.334, 12000., 8700.

10683, 18666.666, 0., 6000.

10684, 19133.334, 0., 6000.

10685, 19600., 0., 6000.

10686, 20066.666, 0., 6000.

10687, 20533.334, 0., 6000.

10688, 20533.334, 15200., 6000.

10689, 20066.666, 15200., 6000.

10690, 19600., 15200., 6000.

10691, 19133.334, 15200., 6000.

10692, 18666.666, 15200., 6000.

10693, 20533.334, 15200., 9000.

10694, 20066.666, 15200., 9000.

10695, 19600., 15200., 9000.

10696, 19133.334, 15200., 9000.

10697, 18666.666, 15200., 9000.

10698, 18666.666, 0., 9000.

10699, 19133.334, 0., 9000.

10700, 19600., 0., 9000.

10701, 20066.666, 0., 9000.

10702, 20533.334, 0., 9000.

10703, 20533.334, 200., 2700.

10704, 20066.666, 200., 2700.

10705, 19600., 200., 2700.

10706, 19133.334, 200., 2700.

10707, 18666.666, 200., 2700.

10708, 18666.666, 0., 2700.

10709, 19133.334, 0., 2700.

10710, 19600., 0., 2700.

10711, 20066.666, 0., 2700.

10712, 20533.334, 0., 2700.

10713, 17533.334, 200., 5700.

10714, 17066.666, 200., 5700.

10715, 16600., 200., 5700.

10716, 16133.333, 200., 5700.

10717, 15666.667, 200., 5700.

10718, 15666.667, 0., 5700.

10719, 16133.333, 0., 5700.

10720, 16600., 0., 5700.

10721, 17066.666, 0., 5700.

10722, 17533.334, 0., 5700.

10723, 15666.667, 15000., 2700.

10724, 16133.333, 15000., 2700.

10725, 16600., 15000., 2700.

10726, 17066.666, 15000., 2700.

10727, 17533.334, 15000., 2700.

10728, 17533.334, 15200., 2700.

10729, 17066.666, 15200., 2700.

10730, 16600., 15200., 2700.

10731, 16133.333, 15200., 2700.

10732, 15666.667, 15200., 2700.

10733, 17533.334, 15200., 9000.

10734, 17066.666, 15200., 9000.

10735, 16600., 15200., 9000.

10736, 16133.333, 15200., 9000.

10737, 15666.667, 15200., 9000.

10738, 15666.667, 0., 9000.

10739, 16133.333, 0., 9000.

10740, 16600., 0., 9000.

10741, 17066.666, 0., 9000.

10742, 17533.334, 0., 9000.

10743, 18200., 3000., 8160.

10744, 18200., 3000., 7620.

10745, 18200., 3000., 7080.

10746, 18200., 3000., 6540.

10747, 18200., 3200., 6540.

10748, 18200., 3200., 7080.

10749, 18200., 3200., 7620.

10750, 18200., 3200., 8160.

10751, 18200., 3000., 5160.

10752, 18200., 3000., 4620.

10753, 18200., 3000., 4080.

10754, 18200., 3000., 3540.

10755, 18200., 3200., 3540.

10756, 18200., 3200., 4080.

10757, 18200., 3200., 4620.

10758, 18200., 3200., 5160.

10759, 18200., 3000., 2160.

10760, 18200., 3000., 1620.

10761, 18200., 3000., 1080.

10762, 18200., 3000., 540.

10763, 18200., 3200., 540.

10764, 18200., 3200., 1080.

10765, 18200., 3200., 1620.

10766, 18200., 3200., 2160.

10767, 18000., 3200., 8160.

10768, 18000., 3200., 7620.

10769, 18000., 3200., 7080.

10770, 18000., 3200., 6540.

10771, 18000., 3000., 6540.

10772, 18000., 3000., 7080.

10773, 18000., 3000., 7620.

10774, 18000., 3000., 8160.

10775, 18000., 3200., 5160.

10776, 18000., 3200., 4620.

10777, 18000., 3200., 4080.

10778, 18000., 3200., 3540.

10779, 18000., 3000., 3540.

10780, 18000., 3000., 4080.

10781, 18000., 3000., 4620.

10782, 18000., 3000., 5160.

10783, 18000., 3000., 540.

10784, 18000., 3000., 1080.

10785, 18000., 3000., 1620.

10786, 18000., 3000., 2160.

10787, 18000., 3200., 2160.

10788, 18000., 3200., 1620.

10789, 18000., 3200., 1080.

10790, 18000., 3200., 540.

10791, 15000., 9200., 2160.

10792, 15000., 9200., 1620.

10793, 15000., 9200., 1080.

10794, 15000., 9200., 540.

10795, 15000., 9000., 540.

10796, 15000., 9000., 1080.

10797, 15000., 9000., 1620.

10798, 15000., 9000., 2160.

10799, 15000., 9200., 5160.

10800, 15000., 9200., 4620.

10801, 15000., 9200., 4080.

10802, 15000., 9200., 3540.

10803, 15000., 9000., 3540.

10804, 15000., 9000., 4080.

10805, 15000., 9000., 4620.

10806, 15000., 9000., 5160.

10807, 15000., 9200., 8160.

10808, 15000., 9200., 7620.

10809, 15000., 9200., 7080.

10810, 15000., 9200., 6540.

10811, 15000., 9000., 6540.

10812, 15000., 9000., 7080.

10813, 15000., 9000., 7620.

10814, 15000., 9000., 8160.

10815, 15200., 9200., 540.

10816, 15200., 9200., 1080.

10817, 15200., 9200., 1620.

10818, 15200., 9200., 2160.

10819, 15200., 9000., 2160.

10820, 15200., 9000., 1620.

10821, 15200., 9000., 1080.

10822, 15200., 9000., 540.

10823, 15200., 9000., 5160.

10824, 15200., 9000., 4620.

10825, 15200., 9000., 4080.

10826, 15200., 9000., 3540.

10827, 15200., 9200., 3540.

10828, 15200., 9200., 4080.

10829, 15200., 9200., 4620.

10830, 15200., 9200., 5160.

10831, 15200., 9000., 8160.

10832, 15200., 9000., 7620.

10833, 15200., 9000., 7080.

10834, 15200., 9000., 6540.

10835, 15200., 9200., 6540.

10836, 15200., 9200., 7080.

10837, 15200., 9200., 7620.

10838, 15200., 9200., 8160.

10839, 12666.667, 15000., 2700.

10840, 13133.333, 15000., 2700.

10841, 13600., 15000., 2700.

10842, 14066.667, 15000., 2700.

10843, 14533.333, 15000., 2700.

10844, 14533.333, 15200., 2700.

10845, 14066.667, 15200., 2700.

10846, 13600., 15200., 2700.

10847, 13133.333, 15200., 2700.

10848, 12666.667, 15200., 2700.

10849, 12666.667, 15000., 5700.

10850, 13133.333, 15000., 5700.

10851, 13600., 15000., 5700.

10852, 14066.667, 15000., 5700.

10853, 14533.333, 15000., 5700.

10854, 14533.333, 15200., 5700.

10855, 14066.667, 15200., 5700.

10856, 13600., 15200., 5700.

10857, 13133.333, 15200., 5700.

10858, 12666.667, 15200., 5700.

10859, 12666.667, 15000., 8700.

10860, 13133.333, 15000., 8700.

10861, 13600., 15000., 8700.

10862, 14066.667, 15000., 8700.

10863, 14533.333, 15000., 8700.

10864, 14533.333, 15200., 8700.

10865, 14066.667, 15200., 8700.

10866, 13600., 15200., 8700.

10867, 13133.333, 15200., 8700.

10868, 12666.667, 15200., 8700.

10869, 12000., 666.666687, 6000.

10870, 12000., 1133.33337, 6000.

10871, 12000., 1600., 6000.

10872, 12000., 2066.66675, 6000.

10873, 12000., 2533.33325, 6000.

10874, 12000., 2533.33325, 5920.

10875, 12000., 2066.66675, 5920.

10876, 12000., 1600., 5920.

10877, 12000., 1133.33337, 5920.

10878, 12000., 666.666687, 5920.

10879, 12000., 2533.33325, 5700.

10880, 12000., 2066.66675, 5700.

10881, 12000., 1600., 5700.

10882, 12000., 1133.33337, 5700.

10883, 12000., 666.666687, 5700.

10884, 12200., 666.666687, 5700.

10885, 12200., 1133.33337, 5700.

10886, 12200., 1600., 5700.

10887, 12200., 2066.66675, 5700.

10888, 12200., 2533.33325, 5700.

10889, 9666.66699, 0., 3000.

10890, 10133.333, 0., 3000.

10891, 10600., 0., 3000.

10892, 11066.667, 0., 3000.

10893, 11533.333, 0., 3000.

10894, 11533.333, 15200., 3000.

10895, 11066.667, 15200., 3000.

10896, 10600., 15200., 3000.

10897, 10133.333, 15200., 3000.

10898, 9666.66699, 15200., 3000.

10899, 9666.66699, 0., 6000.

10900, 10133.333, 0., 6000.

10901, 10600., 0., 6000.

10902, 11066.667, 0., 6000.

10903, 11533.333, 0., 6000.

10904, 11533.333, 15200., 6000.

10905, 11066.667, 15200., 6000.

10906, 10600., 15200., 6000.

10907, 10133.333, 15200., 6000.

10908, 9666.66699, 15200., 6000.

10909, 9200., 6666.6665, 8920.

10910, 9200., 7133.3335, 8920.

10911, 9200., 7600., 8920.

10912, 9200., 8066.6665, 8920.

10913, 9200., 8533.33301, 8920.

10914, 9666.66699, 6000., 9000.

10915, 10133.333, 6000., 9000.

10916, 10600., 6000., 9000.

10917, 11066.667, 6000., 9000.

10918, 11533.333, 6000., 9000.

10919, 9200., 8533.33301, 9000.

10920, 9200., 8066.6665, 9000.

10921, 9200., 7600., 9000.

10922, 9200., 7133.3335, 9000.

10923, 9200., 6666.6665, 9000.

10924, 11533.333, 15200., 9000.

10925, 11066.667, 15200., 9000.

10926, 10600., 15200., 9000.

10927, 10133.333, 15200., 9000.

10928, 9666.66699, 15200., 9000.

10929, 9200., 6666.6665, 8700.

10930, 9200., 7133.3335, 8700.

10931, 9200., 7600., 8700.

10932, 9200., 8066.6665, 8700.

10933, 9200., 8533.33301, 8700.

10934, 9000., 8533.33301, 8700.

10935, 9000., 8066.6665, 8700.

10936, 9000., 7600., 8700.

10937, 9000., 7133.3335, 8700.

10938, 9000., 6666.6665, 8700.

10939, 6666.6665, 15000., 8700.

10940, 7133.3335, 15000., 8700.

10941, 7600., 15000., 8700.

10942, 8066.6665, 15000., 8700.

10943, 8533.33301, 15000., 8700.

10944, 8533.33301, 15200., 8700.

10945, 8066.6665, 15200., 8700.

10946, 7600., 15200., 8700.

10947, 7133.3335, 15200., 8700.

10948, 6666.6665, 15200., 8700.

10949, 6666.6665, 15000., 2700.

10950, 7133.3335, 15000., 2700.

10951, 7600., 15000., 2700.

10952, 8066.6665, 15000., 2700.

10953, 8533.33301, 15000., 2700.

10954, 8533.33301, 15200., 2700.

10955, 8066.6665, 15200., 2700.

10956, 7600., 15200., 2700.

10957, 7133.3335, 15200., 2700.

10958, 6666.6665, 15200., 2700.

10959, 8533.33301, 15200., 6000.

10960, 8066.6665, 15200., 6000.

10961, 7600., 15200., 6000.

10962, 7133.3335, 15200., 6000.

10963, 6666.6665, 15200., 6000.

10964, 6666.6665, 0., 6000.

10965, 7133.3335, 0., 6000.

10966, 7600., 0., 6000.

10967, 8066.6665, 0., 6000.

10968, 8533.33301, 0., 6000.

10969, 6200., 12666.667, 2700.

10970, 6200., 13133.333, 2700.

10971, 6200., 13600., 2700.

10972, 6200., 14066.667, 2700.

10973, 6200., 14533.333, 2700.

10974, 6000., 12666.667, 3000.

10975, 6000., 13133.333, 3000.

10976, 6000., 13600., 3000.

10977, 6000., 14066.667, 3000.

10978, 6000., 14533.333, 3000.

10979, 6000., 14533.333, 2920.

10980, 6000., 14066.667, 2920.

10981, 6000., 13600., 2920.

10982, 6000., 13133.333, 2920.

10983, 6000., 12666.667, 2920.

10984, 6000., 14533.333, 2700.

10985, 6000., 14066.667, 2700.

10986, 6000., 13600., 2700.

10987, 6000., 13133.333, 2700.

10988, 6000., 12666.667, 2700.

10989, 3666.66675, 15000., 8700.

10990, 4133.3335, 15000., 8700.

10991, 4600., 15000., 8700.

10992, 5066.6665, 15000., 8700.

10993, 5533.3335, 15000., 8700.

10994, 5533.3335, 15200., 8700.

10995, 5066.6665, 15200., 8700.

10996, 4600., 15200., 8700.

10997, 4133.3335, 15200., 8700.

10998, 3666.66675, 15200., 8700.

10999, 3200., 666.666687, 2920.

11000, 3200., 1133.33337, 2920.

11001, 3200., 1600., 2920.

11002, 3200., 2066.66675, 2920.

11003, 3200., 2533.33325, 2920.

11004, 3666.66675, 0., 3000.

11005, 4133.3335, 0., 3000.

11006, 4600., 0., 3000.

11007, 5066.6665, 0., 3000.

11008, 5533.3335, 0., 3000.

11009, 3200., 2533.33325, 3000.

11010, 3200., 2066.66675, 3000.

11011, 3200., 1600., 3000.

11012, 3200., 1133.33337, 3000.

11013, 3200., 666.666687, 3000.

11014, 5533.3335, 15200., 3000.

11015, 5066.6665, 15200., 3000.

11016, 4600., 15200., 3000.

11017, 4133.3335, 15200., 3000.

11018, 3666.66675, 15200., 3000.

11019, 3666.66675, 0., 6000.

11020, 4133.3335, 0., 6000.

11021, 4600., 0., 6000.

11022, 5066.6665, 0., 6000.

11023, 5533.3335, 0., 6000.

11024, 5533.3335, 15200., 6000.

11025, 5066.6665, 15200., 6000.

11026, 4600., 15200., 6000.

11027, 4133.3335, 15200., 6000.

11028, 3666.66675, 15200., 6000.

11029, 3000., 2533.33325, 2700.

11030, 3000., 2066.66675, 2700.

11031, 3000., 1600., 2700.

11032, 3000., 1133.33337, 2700.

11033, 3000., 666.666687, 2700.

11034, 3200., 666.666687, 2700.

11035, 3200., 1133.33337, 2700.

11036, 3200., 1600., 2700.

11037, 3200., 2066.66675, 2700.

11038, 3200., 2533.33325, 2700.

11039, 2533.33325, 3200., 2700.

11040, 2066.66675, 3200., 2700.

11041, 1600., 3200., 2700.

11042, 1133.33337, 3200., 2700.

11043, 666.666687, 3200., 2700.

11044, 666.666687, 3000., 2700.

11045, 1133.33337, 3000., 2700.

11046, 1600., 3000., 2700.

11047, 2066.66675, 3000., 2700.

11048, 2533.33325, 3000., 2700.

11049, 666.666687, 0., 6000.

11050, 1133.33337, 0., 6000.

11051, 1600., 0., 6000.

11052, 2066.66675, 0., 6000.

11053, 2533.33325, 0., 6000.

11054, 2533.33325, 15200., 6000.

11055, 2066.66675, 15200., 6000.

11056, 1600., 15200., 6000.

11057, 1133.33337, 15200., 6000.

11058, 666.666687, 15200., 6000.

11059, 2533.33325, 15200., 8700.

11060, 2066.66675, 15200., 8700.

11061, 1600., 15200., 8700.

11062, 1133.33337, 15200., 8700.

11063, 666.666687, 15200., 8700.

11064, 666.666687, 15000., 8700.

11065, 1133.33337, 15000., 8700.

11066, 1600., 15000., 8700.

11067, 2066.66675, 15000., 8700.

11068, 2533.33325, 15000., 8700.

11069, 200., 666.666687, 8700.

11070, 200., 1133.33337, 8700.

11071, 200., 1600., 8700.

11072, 200., 2066.66675, 8700.

11073, 200., 2533.33325, 8700.

11074, 200., 3760., 8700.

11075, 200., 4320., 8700.

11076, 200., 4880., 8700.

11077, 200., 5440., 8700.

11078, 200., 6666.6665, 8700.

11079, 200., 7133.3335, 8700.

11080, 200., 7600., 8700.

11081, 200., 8066.6665, 8700.

11082, 200., 8533.33301, 8700.

11083, 200., 9666.66699, 8700.

11084, 200., 10133.333, 8700.

11085, 200., 10600., 8700.

11086, 200., 11066.667, 8700.

11087, 200., 11533.333, 8700.

11088, 200., 12666.667, 8700.

11089, 200., 13133.333, 8700.

11090, 200., 13600., 8700.

11091, 200., 14066.667, 8700.

11092, 200., 14533.333, 8700.

11093, 0., 2533.33325, 8700.

11094, 0., 2066.66675, 8700.

11095, 0., 1600., 8700.

11096, 0., 1133.33337, 8700.

11097, 0., 666.666687, 8700.

11098, 0., 5440., 8700.

11099, 0., 4880., 8700.

11100, 0., 4320., 8700.

11101, 0., 3760., 8700.

11102, 0., 8533.33301, 8700.

11103, 0., 8066.6665, 8700.

11104, 0., 7600., 8700.

11105, 0., 7133.3335, 8700.

11106, 0., 6666.6665, 8700.

11107, 0., 11533.333, 8700.

11108, 0., 11066.667, 8700.

11109, 0., 10600., 8700.

11110, 0., 10133.333, 8700.

11111, 0., 9666.66699, 8700.

11112, 0., 14533.333, 8700.

11113, 0., 14066.667, 8700.

11114, 0., 13600., 8700.

11115, 0., 13133.333, 8700.

11116, 0., 12666.667, 8700.

11117, 0., 15200., 8850.

11118, 0., 100., 9000.

11119, 0., 666.666687, 9000.

11120, 0., 1133.33337, 9000.

11121, 0., 1600., 9000.

11122, 0., 2066.66675, 9000.

11123, 0., 2533.33325, 9000.

11124, 0., 3666.66675, 9000.

11125, 0., 4133.3335, 9000.

11126, 0., 4600., 9000.

11127, 0., 5066.6665, 9000.

11128, 0., 5533.3335, 9000.

11129, 0., 6666.6665, 9000.

11130, 0., 7133.3335, 9000.

11131, 0., 7600., 9000.

11132, 0., 8066.6665, 9000.

11133, 0., 8533.33301, 9000.

11134, 0., 9760., 9000.

11135, 0., 10320., 9000.

11136, 0., 10880., 9000.

11137, 0., 11440., 9000.

11138, 0., 12760., 9000.

11139, 0., 13320., 9000.

11140, 0., 13880., 9000.

11141, 0., 14440., 9000.

11142, 0., 0., 8850.

11143, 21000., 14533.333, 2700.

11144, 21000., 14066.667, 2700.

11145, 21000., 13600., 2700.

11146, 21000., 13133.333, 2700.

11147, 21000., 12666.667, 2700.

11148, 21200., 12666.667, 2700.

11149, 21200., 13133.333, 2700.

11150, 21200., 13600., 2700.

11151, 21200., 14066.667, 2700.

11152, 21200., 14533.333, 2700.

11153, 24200., 15000., 2850.

11154, 24200., 14533.333, 2850.

11155, 24200., 14066.667, 2850.

11156, 24200., 13600., 2850.

11157, 24200., 13133.333, 2850.

11158, 24200., 12666.667, 2850.

11159, 24200., 12200., 2850.

11160, 24200., 12000., 2850.

11161, 24200., 11533.333, 2850.

11162, 24200., 11066.667, 2850.

11163, 24200., 10600., 2850.

11164, 24200., 10133.333, 2850.

11165, 24200., 9666.66699, 2850.

11166, 24200., 9200., 2850.

11167, 24200., 9000., 2850.

11168, 24200., 8533.33301, 2850.

11169, 24200., 8066.6665, 2850.

11170, 24200., 7600., 2850.

11171, 24200., 7133.3335, 2850.

11172, 24200., 6666.6665, 2850.

11173, 24200., 6200., 2850.

11174, 24200., 6000., 2850.

11175, 24200., 5533.3335, 2850.

11176, 24200., 5066.6665, 2850.

11177, 24200., 4600., 2850.

11178, 24200., 4133.3335, 2850.

11179, 24200., 3666.66675, 2850.

11180, 24200., 3200., 2850.

11181, 24200., 3000., 2850.

11182, 24200., 2533.33325, 2850.

11183, 24200., 2066.66675, 2850.

11184, 24200., 1600., 2850.

11185, 24200., 1133.33337, 2850.

11186, 24200., 666.666687, 2850.

11187, 24200., 200., 2850.

11188, 24200., 15000., 5850.

11189, 24200., 14533.333, 5850.

11190, 24200., 14066.667, 5850.

11191, 24200., 13600., 5850.

11192, 24200., 13133.333, 5850.

11193, 24200., 12666.667, 5850.

11194, 24200., 12200., 5850.

11195, 24200., 12000., 5850.

11196, 24200., 11533.333, 5850.

11197, 24200., 11066.667, 5850.

11198, 24200., 10600., 5850.

11199, 24200., 10133.333, 5850.

11200, 24200., 9666.66699, 5850.

11201, 24200., 9200., 5850.

11202, 24200., 9000., 5850.

11203, 24200., 8533.33301, 5850.

11204, 24200., 8066.6665, 5850.

11205, 24200., 7600., 5850.

11206, 24200., 7133.3335, 5850.

11207, 24200., 6666.6665, 5850.

11208, 24200., 6200., 5850.

11209, 24200., 6000., 5850.

11210, 24200., 5533.3335, 5850.

11211, 24200., 5066.6665, 5850.

11212, 24200., 4600., 5850.

11213, 24200., 4133.3335, 5850.

11214, 24200., 3666.66675, 5850.

11215, 24200., 3200., 5850.

11216, 24200., 3000., 5850.

11217, 24200., 2533.33325, 5850.

11218, 24200., 2066.66675, 5850.

11219, 24200., 1600., 5850.

11220, 24200., 1133.33337, 5850.

11221, 24200., 666.666687, 5850.

11222, 24200., 200., 5850.

11223, 24200., 200., 8850.

11224, 24200., 713.333313, 8850.

11225, 24200., 1226.66663, 8850.

11226, 24200., 1740., 8850.

11227, 24200., 2253.33325, 8850.

11228, 24200., 2766.66675, 8850.

11229, 24200., 3100., 8850.

11230, 24200., 3480., 8850.

11231, 24200., 4040., 8850.

11232, 24200., 4600., 8850.

11233, 24200., 5160., 8850.

11234, 24200., 5720., 8850.

11235, 24200., 6100., 8850.

11236, 24200., 6480., 8850.

11237, 24200., 6993.3335, 8850.

11238, 24200., 7506.6665, 8850.

11239, 24200., 8020., 8850.

11240, 24200., 8533.33301, 8850.

11241, 24200., 8866.66699, 8850.

11242, 24200., 9333.33301, 8850.

11243, 24200., 9666.66699, 8850.

11244, 24200., 10133.333, 8850.

11245, 24200., 10600., 8850.

11246, 24200., 11066.667, 8850.

11247, 24200., 11533.333, 8850.

11248, 24200., 11866.667, 8850.

11249, 24200., 12333.333, 8850.

11250, 24200., 12666.667, 8850.

11251, 24200., 13133.333, 8850.

11252, 24200., 13600., 8850.

11253, 24200., 14066.667, 8850.

11254, 24200., 14533.333, 8850.

11255, 24200., 14800., 8850.

11256, 24200., 15066.667, 8850.

11257, 8533.33301, 14533.333, 8920.

11258, 8533.33301, 14066.667, 8920.

11259, 8533.33301, 13600., 8920.

11260, 8533.33301, 13133.333, 8920.

11261, 8533.33301, 12666.667, 8920.

11262, 8066.6665, 14533.333, 8920.

11263, 8066.6665, 14066.667, 8920.

11264, 8066.6665, 13600., 8920.

11265, 8066.6665, 13133.333, 8920.

11266, 8066.6665, 12666.667, 8920.

11267, 7600., 14533.333, 8920.

11268, 7600., 14066.667, 8920.

11269, 7600., 13600., 8920.

11270, 7600., 13133.333, 8920.

11271, 7600., 12666.667, 8920.

11272, 7133.3335, 14533.333, 8920.

11273, 7133.3335, 14066.667, 8920.

11274, 7133.3335, 13600., 8920.

11275, 7133.3335, 13133.333, 8920.

11276, 7133.3335, 12666.667, 8920.

11277, 6666.6665, 14533.333, 8920.

11278, 6666.6665, 14066.667, 8920.

11279, 6666.6665, 13600., 8920.

11280, 6666.6665, 13133.333, 8920.

11281, 6666.6665, 12666.667, 8920.

11282, 8533.33301, 11533.333, 8920.

11283, 8533.33301, 11066.667, 8920.

11284, 8533.33301, 10600., 8920.

11285, 8533.33301, 10133.333, 8920.

11286, 8533.33301, 9666.66699, 8920.

11287, 8066.6665, 11533.333, 8920.

11288, 8066.6665, 11066.667, 8920.

11289, 8066.6665, 10600., 8920.

11290, 8066.6665, 10133.333, 8920.

11291, 8066.6665, 9666.66699, 8920.

11292, 7600., 11533.333, 8920.

11293, 7600., 11066.667, 8920.

11294, 7600., 10600., 8920.

11295, 7600., 10133.333, 8920.

11296, 7600., 9666.66699, 8920.

11297, 7133.3335, 11533.333, 8920.

11298, 7133.3335, 11066.667, 8920.

11299, 7133.3335, 10600., 8920.

11300, 7133.3335, 10133.333, 8920.

11301, 7133.3335, 9666.66699, 8920.

11302, 6666.6665, 11533.333, 8920.

11303, 6666.6665, 11066.667, 8920.

11304, 6666.6665, 10600., 8920.

11305, 6666.6665, 10133.333, 8920.

11306, 6666.6665, 9666.66699, 8920.

11307, 8533.33301, 8533.33301, 8920.

11308, 8533.33301, 8066.6665, 8920.

11309, 8533.33301, 7600., 8920.

11310, 8533.33301, 7133.3335, 8920.

11311, 8533.33301, 6666.6665, 8920.

11312, 8066.6665, 8533.33301, 8920.

11313, 8066.6665, 8066.6665, 8920.

11314, 8066.6665, 7600., 8920.

11315, 8066.6665, 7133.3335, 8920.

11316, 8066.6665, 6666.6665, 8920.

11317, 7600., 8533.33301, 8920.

11318, 7600., 8066.6665, 8920.

11319, 7600., 7600., 8920.

11320, 7600., 7133.3335, 8920.

11321, 7600., 6666.6665, 8920.

11322, 7133.3335, 8533.33301, 8920.

11323, 7133.3335, 8066.6665, 8920.

11324, 7133.3335, 7600., 8920.

11325, 7133.3335, 7133.3335, 8920.

11326, 7133.3335, 6666.6665, 8920.

11327, 6666.6665, 8533.33301, 8920.

11328, 6666.6665, 8066.6665, 8920.

11329, 6666.6665, 7600., 8920.

11330, 6666.6665, 7133.3335, 8920.

11331, 6666.6665, 6666.6665, 8920.

11332, 6666.6665, 15000., 9000.

11333, 7133.3335, 15000., 9000.

11334, 7600., 15000., 9000.

11335, 8066.6665, 15000., 9000.

11336, 8533.33301, 15000., 9000.

11337, 6666.6665, 14533.333, 9000.

11338, 7133.3335, 14533.333, 9000.

11339, 7600., 14533.333, 9000.

11340, 8066.6665, 14533.333, 9000.

11341, 8533.33301, 14533.333, 9000.

11342, 6666.6665, 14066.667, 9000.

11343, 7133.3335, 14066.667, 9000.

11344, 7600., 14066.667, 9000.

11345, 8066.6665, 14066.667, 9000.

11346, 8533.33301, 14066.667, 9000.

11347, 6666.6665, 13600., 9000.

11348, 7133.3335, 13600., 9000.

11349, 7600., 13600., 9000.

11350, 8066.6665, 13600., 9000.

11351, 8533.33301, 13600., 9000.

11352, 6666.6665, 13133.333, 9000.

11353, 7133.3335, 13133.333, 9000.

11354, 7600., 13133.333, 9000.

11355, 8066.6665, 13133.333, 9000.

11356, 8533.33301, 13133.333, 9000.

11357, 6666.6665, 12666.667, 9000.

11358, 7133.3335, 12666.667, 9000.

11359, 7600., 12666.667, 9000.

11360, 8066.6665, 12666.667, 9000.

11361, 8533.33301, 12666.667, 9000.

11362, 6666.6665, 12200., 9000.

11363, 7133.3335, 12200., 9000.

11364, 7600., 12200., 9000.

11365, 8066.6665, 12200., 9000.

11366, 8533.33301, 12200., 9000.

11367, 6666.6665, 12000., 9000.

11368, 7133.3335, 12000., 9000.

11369, 7600., 12000., 9000.

11370, 8066.6665, 12000., 9000.

11371, 8533.33301, 12000., 9000.

11372, 6666.6665, 11533.333, 9000.

11373, 7133.3335, 11533.333, 9000.

11374, 7600., 11533.333, 9000.

11375, 8066.6665, 11533.333, 9000.

11376, 8533.33301, 11533.333, 9000.

11377, 6666.6665, 11066.667, 9000.

11378, 7133.3335, 11066.667, 9000.

11379, 7600., 11066.667, 9000.

11380, 8066.6665, 11066.667, 9000.

11381, 8533.33301, 11066.667, 9000.

11382, 6666.6665, 10600., 9000.

11383, 7133.3335, 10600., 9000.

11384, 7600., 10600., 9000.

11385, 8066.6665, 10600., 9000.

11386, 8533.33301, 10600., 9000.

11387, 6666.6665, 10133.333, 9000.

11388, 7133.3335, 10133.333, 9000.

11389, 7600., 10133.333, 9000.

11390, 8066.6665, 10133.333, 9000.

11391, 8533.33301, 10133.333, 9000.

11392, 6666.6665, 9666.66699, 9000.

11393, 7133.3335, 9666.66699, 9000.

11394, 7600., 9666.66699, 9000.

11395, 8066.6665, 9666.66699, 9000.

11396, 8533.33301, 9666.66699, 9000.

11397, 6666.6665, 9200., 9000.

11398, 7133.3335, 9200., 9000.

11399, 7600., 9200., 9000.

11400, 8066.6665, 9200., 9000.

11401, 8533.33301, 9200., 9000.

11402, 6666.6665, 9000., 9000.

11403, 7133.3335, 9000., 9000.

11404, 7600., 9000., 9000.

11405, 8066.6665, 9000., 9000.

11406, 8533.33301, 9000., 9000.

11407, 6666.6665, 8533.33301, 9000.

11408, 7133.3335, 8533.33301, 9000.

11409, 7600., 8533.33301, 9000.

11410, 8066.6665, 8533.33301, 9000.

11411, 8533.33301, 8533.33301, 9000.

11412, 6666.6665, 8066.6665, 9000.

11413, 7133.3335, 8066.6665, 9000.

11414, 7600., 8066.6665, 9000.

11415, 8066.6665, 8066.6665, 9000.

11416, 8533.33301, 8066.6665, 9000.

11417, 6666.6665, 7600., 9000.

11418, 7133.3335, 7600., 9000.

11419, 7600., 7600., 9000.

11420, 8066.6665, 7600., 9000.

11421, 8533.33301, 7600., 9000.

11422, 6666.6665, 7133.3335, 9000.

11423, 7133.3335, 7133.3335, 9000.

11424, 7600., 7133.3335, 9000.

11425, 8066.6665, 7133.3335, 9000.

11426, 8533.33301, 7133.3335, 9000.

11427, 6666.6665, 6666.6665, 9000.

11428, 7133.3335, 6666.6665, 9000.

11429, 7600., 6666.6665, 9000.

11430, 8066.6665, 6666.6665, 9000.

11431, 8533.33301, 6666.6665, 9000.

11432, 6666.6665, 6200., 9000.

11433, 7133.3335, 6200., 9000.

11434, 7600., 6200., 9000.

11435, 8066.6665, 6200., 9000.

11436, 8533.33301, 6200., 9000.

11437, 12666.667, 12666.667, 8920.

11438, 12666.667, 13133.333, 8920.

11439, 12666.667, 13600., 8920.

11440, 12666.667, 14066.667, 8920.

11441, 12666.667, 14533.333, 8920.

11442, 13133.333, 12666.667, 8920.

11443, 13133.333, 13133.333, 8920.

11444, 13133.333, 13600., 8920.

11445, 13133.333, 14066.667, 8920.

11446, 13133.333, 14533.333, 8920.

11447, 13600., 12666.667, 8920.

11448, 13600., 13133.333, 8920.

11449, 13600., 13600., 8920.

11450, 13600., 14066.667, 8920.

11451, 13600., 14533.333, 8920.

11452, 14066.667, 12666.667, 8920.

11453, 14066.667, 13133.333, 8920.

11454, 14066.667, 13600., 8920.

11455, 14066.667, 14066.667, 8920.

11456, 14066.667, 14533.333, 8920.

11457, 14533.333, 12666.667, 8920.

11458, 14533.333, 13133.333, 8920.

11459, 14533.333, 13600., 8920.

11460, 14533.333, 14066.667, 8920.

11461, 14533.333, 14533.333, 8920.

11462, 12666.667, 9666.66699, 8920.

11463, 12666.667, 10133.333, 8920.

11464, 12666.667, 10600., 8920.

11465, 12666.667, 11066.667, 8920.

11466, 12666.667, 11533.333, 8920.

11467, 13133.333, 9666.66699, 8920.

11468, 13133.333, 10133.333, 8920.

11469, 13133.333, 10600., 8920.

11470, 13133.333, 11066.667, 8920.

11471, 13133.333, 11533.333, 8920.

11472, 13600., 9666.66699, 8920.

11473, 13600., 10133.333, 8920.

11474, 13600., 10600., 8920.

11475, 13600., 11066.667, 8920.

11476, 13600., 11533.333, 8920.

11477, 14066.667, 9666.66699, 8920.

11478, 14066.667, 10133.333, 8920.

11479, 14066.667, 10600., 8920.

11480, 14066.667, 11066.667, 8920.

11481, 14066.667, 11533.333, 8920.

11482, 14533.333, 9666.66699, 8920.

11483, 14533.333, 10133.333, 8920.

11484, 14533.333, 10600., 8920.

11485, 14533.333, 11066.667, 8920.

11486, 14533.333, 11533.333, 8920.

11487, 12666.667, 6666.6665, 8920.

11488, 12666.667, 7133.3335, 8920.

11489, 12666.667, 7600., 8920.

11490, 12666.667, 8066.6665, 8920.

11491, 12666.667, 8533.33301, 8920.

11492, 13133.333, 6666.6665, 8920.

11493, 13133.333, 7133.3335, 8920.

11494, 13133.333, 7600., 8920.

11495, 13133.333, 8066.6665, 8920.

11496, 13133.333, 8533.33301, 8920.

11497, 13600., 6666.6665, 8920.

11498, 13600., 7133.3335, 8920.

11499, 13600., 7600., 8920.

11500, 13600., 8066.6665, 8920.

11501, 13600., 8533.33301, 8920.

11502, 14066.667, 6666.6665, 8920.

11503, 14066.667, 7133.3335, 8920.

11504, 14066.667, 7600., 8920.

11505, 14066.667, 8066.6665, 8920.

11506, 14066.667, 8533.33301, 8920.

11507, 14533.333, 6666.6665, 8920.

11508, 14533.333, 7133.3335, 8920.

11509, 14533.333, 7600., 8920.

11510, 14533.333, 8066.6665, 8920.

11511, 14533.333, 8533.33301, 8920.

11512, 12666.667, 15000., 9000.

11513, 13133.333, 15000., 9000.

11514, 13600., 15000., 9000.

11515, 14066.667, 15000., 9000.

11516, 14533.333, 15000., 9000.

11517, 12666.667, 14533.333, 9000.

11518, 13133.333, 14533.333, 9000.

11519, 13600., 14533.333, 9000.

11520, 14066.667, 14533.333, 9000.

11521, 14533.333, 14533.333, 9000.

11522, 12666.667, 14066.667, 9000.

11523, 13133.333, 14066.667, 9000.

11524, 13600., 14066.667, 9000.

11525, 14066.667, 14066.667, 9000.

11526, 14533.333, 14066.667, 9000.

11527, 12666.667, 13600., 9000.

11528, 13133.333, 13600., 9000.

11529, 13600., 13600., 9000.

11530, 14066.667, 13600., 9000.

11531, 14533.333, 13600., 9000.

11532, 12666.667, 13133.333, 9000.

11533, 13133.333, 13133.333, 9000.

11534, 13600., 13133.333, 9000.

11535, 14066.667, 13133.333, 9000.

11536, 14533.333, 13133.333, 9000.

11537, 12666.667, 12666.667, 9000.

11538, 13133.333, 12666.667, 9000.

11539, 13600., 12666.667, 9000.

11540, 14066.667, 12666.667, 9000.

11541, 14533.333, 12666.667, 9000.

11542, 12666.667, 12200., 9000.

11543, 13133.333, 12200., 9000.

11544, 13600., 12200., 9000.

11545, 14066.667, 12200., 9000.

11546, 14533.333, 12200., 9000.

11547, 12666.667, 12000., 9000.

11548, 13133.333, 12000., 9000.

11549, 13600., 12000., 9000.

11550, 14066.667, 12000., 9000.

11551, 14533.333, 12000., 9000.

11552, 12666.667, 11533.333, 9000.

11553, 13133.333, 11533.333, 9000.

11554, 13600., 11533.333, 9000.

11555, 14066.667, 11533.333, 9000.

11556, 14533.333, 11533.333, 9000.

11557, 12666.667, 11066.667, 9000.

11558, 13133.333, 11066.667, 9000.

11559, 13600., 11066.667, 9000.

11560, 14066.667, 11066.667, 9000.

11561, 14533.333, 11066.667, 9000.

11562, 12666.667, 10600., 9000.

11563, 13133.333, 10600., 9000.

11564, 13600., 10600., 9000.

11565, 14066.667, 10600., 9000.

11566, 14533.333, 10600., 9000.

11567, 12666.667, 10133.333, 9000.

11568, 13133.333, 10133.333, 9000.

11569, 13600., 10133.333, 9000.

11570, 14066.667, 10133.333, 9000.

11571, 14533.333, 10133.333, 9000.

11572, 12666.667, 9666.66699, 9000.

11573, 13133.333, 9666.66699, 9000.

11574, 13600., 9666.66699, 9000.

11575, 14066.667, 9666.66699, 9000.

11576, 14533.333, 9666.66699, 9000.

11577, 12666.667, 9200., 9000.

11578, 13133.333, 9200., 9000.

11579, 13600., 9200., 9000.

11580, 14066.667, 9200., 9000.

11581, 14533.333, 9200., 9000.

11582, 12666.667, 9000., 9000.

11583, 13133.333, 9000., 9000.

11584, 13600., 9000., 9000.

11585, 14066.667, 9000., 9000.

11586, 14533.333, 9000., 9000.

11587, 12666.667, 8533.33301, 9000.

11588, 13133.333, 8533.33301, 9000.

11589, 13600., 8533.33301, 9000.

11590, 14066.667, 8533.33301, 9000.

11591, 14533.333, 8533.33301, 9000.

11592, 12666.667, 8066.6665, 9000.

11593, 13133.333, 8066.6665, 9000.

11594, 13600., 8066.6665, 9000.

11595, 14066.667, 8066.6665, 9000.

11596, 14533.333, 8066.6665, 9000.

11597, 12666.667, 7600., 9000.

11598, 13133.333, 7600., 9000.

11599, 13600., 7600., 9000.

11600, 14066.667, 7600., 9000.

11601, 14533.333, 7600., 9000.

11602, 12666.667, 7133.3335, 9000.

11603, 13133.333, 7133.3335, 9000.

11604, 13600., 7133.3335, 9000.

11605, 14066.667, 7133.3335, 9000.

11606, 14533.333, 7133.3335, 9000.

11607, 12666.667, 6666.6665, 9000.

11608, 13133.333, 6666.6665, 9000.

11609, 13600., 6666.6665, 9000.

11610, 14066.667, 6666.6665, 9000.

11611, 14533.333, 6666.6665, 9000.

11612, 12666.667, 6200., 9000.

11613, 13133.333, 6200., 9000.

11614, 13600., 6200., 9000.

11615, 14066.667, 6200., 9000.

11616, 14533.333, 6200., 9000.

11617, 8533.33301, 14533.333, 2920.

11618, 8533.33301, 14066.667, 2920.

11619, 8533.33301, 13600., 2920.

11620, 8533.33301, 13133.333, 2920.

11621, 8533.33301, 12666.667, 2920.

11622, 8066.6665, 14533.333, 2920.

11623, 8066.6665, 14066.667, 2920.

11624, 8066.6665, 13600., 2920.

11625, 8066.6665, 13133.333, 2920.

11626, 8066.6665, 12666.667, 2920.

11627, 7600., 14533.333, 2920.

11628, 7600., 14066.667, 2920.

11629, 7600., 13600., 2920.

11630, 7600., 13133.333, 2920.

11631, 7600., 12666.667, 2920.

11632, 7133.3335, 14533.333, 2920.

11633, 7133.3335, 14066.667, 2920.

11634, 7133.3335, 13600., 2920.

11635, 7133.3335, 13133.333, 2920.

11636, 7133.3335, 12666.667, 2920.

11637, 6666.6665, 14533.333, 2920.

11638, 6666.6665, 14066.667, 2920.

11639, 6666.6665, 13600., 2920.

11640, 6666.6665, 13133.333, 2920.

11641, 6666.6665, 12666.667, 2920.

11642, 8533.33301, 11533.333, 2920.

11643, 8533.33301, 11066.667, 2920.

11644, 8533.33301, 10600., 2920.

11645, 8533.33301, 10133.333, 2920.

11646, 8533.33301, 9666.66699, 2920.

11647, 8066.6665, 11533.333, 2920.

11648, 8066.6665, 11066.667, 2920.

11649, 8066.6665, 10600., 2920.

11650, 8066.6665, 10133.333, 2920.

11651, 8066.6665, 9666.66699, 2920.

11652, 7600., 11533.333, 2920.

11653, 7600., 11066.667, 2920.

11654, 7600., 10600., 2920.

11655, 7600., 10133.333, 2920.

11656, 7600., 9666.66699, 2920.

11657, 7133.3335, 11533.333, 2920.

11658, 7133.3335, 11066.667, 2920.

11659, 7133.3335, 10600., 2920.

11660, 7133.3335, 10133.333, 2920.

11661, 7133.3335, 9666.66699, 2920.

11662, 6666.6665, 11533.333, 2920.

11663, 6666.6665, 11066.667, 2920.

11664, 6666.6665, 10600., 2920.

11665, 6666.6665, 10133.333, 2920.

11666, 6666.6665, 9666.66699, 2920.

11667, 8533.33301, 8533.33301, 2920.

11668, 8533.33301, 8066.6665, 2920.

11669, 8533.33301, 7600., 2920.

11670, 8533.33301, 7133.3335, 2920.

11671, 8533.33301, 6666.6665, 2920.

11672, 8066.6665, 8533.33301, 2920.

11673, 8066.6665, 8066.6665, 2920.

11674, 8066.6665, 7600., 2920.

11675, 8066.6665, 7133.3335, 2920.

11676, 8066.6665, 6666.6665, 2920.

11677, 7600., 8533.33301, 2920.

11678, 7600., 8066.6665, 2920.

11679, 7600., 7600., 2920.

11680, 7600., 7133.3335, 2920.

11681, 7600., 6666.6665, 2920.

11682, 7133.3335, 8533.33301, 2920.

11683, 7133.3335, 8066.6665, 2920.

11684, 7133.3335, 7600., 2920.

11685, 7133.3335, 7133.3335, 2920.

11686, 7133.3335, 6666.6665, 2920.

11687, 6666.6665, 8533.33301, 2920.

11688, 6666.6665, 8066.6665, 2920.

11689, 6666.6665, 7600., 2920.

11690, 6666.6665, 7133.3335, 2920.

11691, 6666.6665, 6666.6665, 2920.

11692, 8533.33301, 5533.3335, 2920.

11693, 8533.33301, 5066.6665, 2920.

11694, 8533.33301, 4600., 2920.

11695, 8533.33301, 4133.3335, 2920.

11696, 8533.33301, 3666.66675, 2920.

11697, 8066.6665, 5533.3335, 2920.

11698, 8066.6665, 5066.6665, 2920.

11699, 8066.6665, 4600., 2920.

11700, 8066.6665, 4133.3335, 2920.

11701, 8066.6665, 3666.66675, 2920.

11702, 7600., 5533.3335, 2920.

11703, 7600., 5066.6665, 2920.

11704, 7600., 4600., 2920.

11705, 7600., 4133.3335, 2920.

11706, 7600., 3666.66675, 2920.

11707, 7133.3335, 5533.3335, 2920.

11708, 7133.3335, 5066.6665, 2920.

11709, 7133.3335, 4600., 2920.

11710, 7133.3335, 4133.3335, 2920.

11711, 7133.3335, 3666.66675, 2920.

11712, 6666.6665, 5533.3335, 2920.

11713, 6666.6665, 5066.6665, 2920.

11714, 6666.6665, 4600., 2920.

11715, 6666.6665, 4133.3335, 2920.

11716, 6666.6665, 3666.66675, 2920.

11717, 6666.6665, 666.666687, 2920.

11718, 6666.6665, 1133.33337, 2920.

11719, 6666.6665, 1600., 2920.

11720, 6666.6665, 2066.66675, 2920.

11721, 6666.6665, 2533.33325, 2920.

11722, 7133.3335, 666.666687, 2920.

11723, 7133.3335, 1133.33337, 2920.

11724, 7133.3335, 1600., 2920.

11725, 7133.3335, 2066.66675, 2920.

11726, 7133.3335, 2533.33325, 2920.

11727, 7600., 666.666687, 2920.

11728, 7600., 1133.33337, 2920.

11729, 7600., 1600., 2920.

11730, 7600., 2066.66675, 2920.

11731, 7600., 2533.33325, 2920.

11732, 8066.6665, 666.666687, 2920.

11733, 8066.6665, 1133.33337, 2920.

11734, 8066.6665, 1600., 2920.

11735, 8066.6665, 2066.66675, 2920.

11736, 8066.6665, 2533.33325, 2920.

11737, 8533.33301, 666.666687, 2920.

11738, 8533.33301, 1133.33337, 2920.

11739, 8533.33301, 1600., 2920.

11740, 8533.33301, 2066.66675, 2920.

11741, 8533.33301, 2533.33325, 2920.

11742, 6666.6665, 15000., 3000.

11743, 7133.3335, 15000., 3000.

11744, 7600., 15000., 3000.

11745, 8066.6665, 15000., 3000.

11746, 8533.33301, 15000., 3000.

11747, 6666.6665, 14533.333, 3000.

11748, 7133.3335, 14533.333, 3000.

11749, 7600., 14533.333, 3000.

11750, 8066.6665, 14533.333, 3000.

11751, 8533.33301, 14533.333, 3000.

11752, 6666.6665, 14066.667, 3000.

11753, 7133.3335, 14066.667, 3000.

11754, 7600., 14066.667, 3000.

11755, 8066.6665, 14066.667, 3000.

11756, 8533.33301, 14066.667, 3000.

11757, 6666.6665, 13600., 3000.

11758, 7133.3335, 13600., 3000.

11759, 7600., 13600., 3000.

11760, 8066.6665, 13600., 3000.

11761, 8533.33301, 13600., 3000.

11762, 6666.6665, 13133.333, 3000.

11763, 7133.3335, 13133.333, 3000.

11764, 7600., 13133.333, 3000.

11765, 8066.6665, 13133.333, 3000.

11766, 8533.33301, 13133.333, 3000.

11767, 6666.6665, 12666.667, 3000.

11768, 7133.3335, 12666.667, 3000.

11769, 7600., 12666.667, 3000.

11770, 8066.6665, 12666.667, 3000.

11771, 8533.33301, 12666.667, 3000.

11772, 6666.6665, 12200., 3000.

11773, 7133.3335, 12200., 3000.

11774, 7600., 12200., 3000.

11775, 8066.6665, 12200., 3000.

11776, 8533.33301, 12200., 3000.

11777, 6666.6665, 12000., 3000.

11778, 7133.3335, 12000., 3000.

11779, 7600., 12000., 3000.

11780, 8066.6665, 12000., 3000.

11781, 8533.33301, 12000., 3000.

11782, 6666.6665, 11533.333, 3000.

11783, 7133.3335, 11533.333, 3000.

11784, 7600., 11533.333, 3000.

11785, 8066.6665, 11533.333, 3000.

11786, 8533.33301, 11533.333, 3000.

11787, 6666.6665, 11066.667, 3000.

11788, 7133.3335, 11066.667, 3000.

11789, 7600., 11066.667, 3000.

11790, 8066.6665, 11066.667, 3000.

11791, 8533.33301, 11066.667, 3000.

11792, 6666.6665, 10600., 3000.

11793, 7133.3335, 10600., 3000.

11794, 7600., 10600., 3000.

11795, 8066.6665, 10600., 3000.

11796, 8533.33301, 10600., 3000.

11797, 6666.6665, 10133.333, 3000.

11798, 7133.3335, 10133.333, 3000.

11799, 7600., 10133.333, 3000.

11800, 8066.6665, 10133.333, 3000.

11801, 8533.33301, 10133.333, 3000.

11802, 6666.6665, 9666.66699, 3000.

11803, 7133.3335, 9666.66699, 3000.

11804, 7600., 9666.66699, 3000.

11805, 8066.6665, 9666.66699, 3000.

11806, 8533.33301, 9666.66699, 3000.

11807, 6666.6665, 9200., 3000.

11808, 7133.3335, 9200., 3000.

11809, 7600., 9200., 3000.

11810, 8066.6665, 9200., 3000.

11811, 8533.33301, 9200., 3000.

11812, 6666.6665, 9000., 3000.

11813, 7133.3335, 9000., 3000.

11814, 7600., 9000., 3000.

11815, 8066.6665, 9000., 3000.

11816, 8533.33301, 9000., 3000.

11817, 6666.6665, 8533.33301, 3000.

11818, 7133.3335, 8533.33301, 3000.

11819, 7600., 8533.33301, 3000.

11820, 8066.6665, 8533.33301, 3000.

11821, 8533.33301, 8533.33301, 3000.

11822, 6666.6665, 8066.6665, 3000.

11823, 7133.3335, 8066.6665, 3000.

11824, 7600., 8066.6665, 3000.

11825, 8066.6665, 8066.6665, 3000.

11826, 8533.33301, 8066.6665, 3000.

11827, 6666.6665, 7600., 3000.

11828, 7133.3335, 7600., 3000.

11829, 7600., 7600., 3000.

11830, 8066.6665, 7600., 3000.

11831, 8533.33301, 7600., 3000.

11832, 6666.6665, 7133.3335, 3000.

11833, 7133.3335, 7133.3335, 3000.

11834, 7600., 7133.3335, 3000.

11835, 8066.6665, 7133.3335, 3000.

11836, 8533.33301, 7133.3335, 3000.

11837, 6666.6665, 6666.6665, 3000.

11838, 7133.3335, 6666.6665, 3000.

11839, 7600., 6666.6665, 3000.

11840, 8066.6665, 6666.6665, 3000.

11841, 8533.33301, 6666.6665, 3000.

11842, 6666.6665, 6200., 3000.

11843, 7133.3335, 6200., 3000.

11844, 7600., 6200., 3000.

11845, 8066.6665, 6200., 3000.

11846, 8533.33301, 6200., 3000.

11847, 6666.6665, 6000., 3000.

11848, 7133.3335, 6000., 3000.

11849, 7600., 6000., 3000.

11850, 8066.6665, 6000., 3000.

11851, 8533.33301, 6000., 3000.

11852, 6666.6665, 5533.3335, 3000.

11853, 7133.3335, 5533.3335, 3000.

11854, 7600., 5533.3335, 3000.

11855, 8066.6665, 5533.3335, 3000.

11856, 8533.33301, 5533.3335, 3000.

11857, 6666.6665, 5066.6665, 3000.

11858, 7133.3335, 5066.6665, 3000.

11859, 7600., 5066.6665, 3000.

11860, 8066.6665, 5066.6665, 3000.

11861, 8533.33301, 5066.6665, 3000.

11862, 6666.6665, 4600., 3000.

11863, 7133.3335, 4600., 3000.

11864, 7600., 4600., 3000.

11865, 8066.6665, 4600., 3000.

11866, 8533.33301, 4600., 3000.

11867, 6666.6665, 4133.3335, 3000.

11868, 7133.3335, 4133.3335, 3000.

11869, 7600., 4133.3335, 3000.

11870, 8066.6665, 4133.3335, 3000.

11871, 8533.33301, 4133.3335, 3000.

11872, 6666.6665, 3666.66675, 3000.

11873, 7133.3335, 3666.66675, 3000.

11874, 7600., 3666.66675, 3000.

11875, 8066.6665, 3666.66675, 3000.

11876, 8533.33301, 3666.66675, 3000.

11877, 6666.6665, 3200., 3000.

11878, 7133.3335, 3200., 3000.

11879, 7600., 3200., 3000.

11880, 8066.6665, 3200., 3000.

11881, 8533.33301, 3200., 3000.

11882, 6666.6665, 3000., 3000.

11883, 7133.3335, 3000., 3000.

11884, 7600., 3000., 3000.

11885, 8066.6665, 3000., 3000.

11886, 8533.33301, 3000., 3000.

11887, 6666.6665, 2533.33325, 3000.

11888, 7133.3335, 2533.33325, 3000.

11889, 7600., 2533.33325, 3000.

11890, 8066.6665, 2533.33325, 3000.

11891, 8533.33301, 2533.33325, 3000.

11892, 6666.6665, 2066.66675, 3000.

11893, 7133.3335, 2066.66675, 3000.

11894, 7600., 2066.66675, 3000.

11895, 8066.6665, 2066.66675, 3000.

11896, 8533.33301, 2066.66675, 3000.

11897, 6666.6665, 1600., 3000.

11898, 7133.3335, 1600., 3000.

11899, 7600., 1600., 3000.

11900, 8066.6665, 1600., 3000.

11901, 8533.33301, 1600., 3000.

11902, 6666.6665, 1133.33337, 3000.

11903, 7133.3335, 1133.33337, 3000.

11904, 7600., 1133.33337, 3000.

11905, 8066.6665, 1133.33337, 3000.

11906, 8533.33301, 1133.33337, 3000.

11907, 6666.6665, 666.666687, 3000.

11908, 7133.3335, 666.666687, 3000.

11909, 7600., 666.666687, 3000.

11910, 8066.6665, 666.666687, 3000.

11911, 8533.33301, 666.666687, 3000.

11912, 6666.6665, 200., 3000.

11913, 7133.3335, 200., 3000.

11914, 7600., 200., 3000.

11915, 8066.6665, 200., 3000.

11916, 8533.33301, 200., 3000.

11917, 14533.333, 14533.333, 2920.

11918, 14533.333, 14066.667, 2920.

11919, 14533.333, 13600., 2920.

11920, 14533.333, 13133.333, 2920.

11921, 14533.333, 12666.667, 2920.

11922, 14066.667, 14533.333, 2920.

11923, 14066.667, 14066.667, 2920.

11924, 14066.667, 13600., 2920.

11925, 14066.667, 13133.333, 2920.

11926, 14066.667, 12666.667, 2920.

11927, 13600., 14533.333, 2920.

11928, 13600., 14066.667, 2920.

11929, 13600., 13600., 2920.

11930, 13600., 13133.333, 2920.

11931, 13600., 12666.667, 2920.

11932, 13133.333, 14533.333, 2920.

11933, 13133.333, 14066.667, 2920.

11934, 13133.333, 13600., 2920.

11935, 13133.333, 13133.333, 2920.

11936, 13133.333, 12666.667, 2920.

11937, 12666.667, 14533.333, 2920.

11938, 12666.667, 14066.667, 2920.

11939, 12666.667, 13600., 2920.

11940, 12666.667, 13133.333, 2920.

11941, 12666.667, 12666.667, 2920.

11942, 14533.333, 11533.333, 2920.

11943, 14533.333, 11066.667, 2920.

11944, 14533.333, 10600., 2920.

11945, 14533.333, 10133.333, 2920.

11946, 14533.333, 9666.66699, 2920.

11947, 14066.667, 11533.333, 2920.

11948, 14066.667, 11066.667, 2920.

11949, 14066.667, 10600., 2920.

11950, 14066.667, 10133.333, 2920.

11951, 14066.667, 9666.66699, 2920.

11952, 13600., 11533.333, 2920.

11953, 13600., 11066.667, 2920.

11954, 13600., 10600., 2920.

11955, 13600., 10133.333, 2920.

11956, 13600., 9666.66699, 2920.

11957, 13133.333, 11533.333, 2920.

11958, 13133.333, 11066.667, 2920.

11959, 13133.333, 10600., 2920.

11960, 13133.333, 10133.333, 2920.

11961, 13133.333, 9666.66699, 2920.

11962, 12666.667, 11533.333, 2920.

11963, 12666.667, 11066.667, 2920.

11964, 12666.667, 10600., 2920.

11965, 12666.667, 10133.333, 2920.

11966, 12666.667, 9666.66699, 2920.

11967, 14533.333, 8533.33301, 2920.

11968, 14533.333, 8066.6665, 2920.

11969, 14533.333, 7600., 2920.

11970, 14533.333, 7133.3335, 2920.

11971, 14533.333, 6666.6665, 2920.

11972, 14066.667, 8533.33301, 2920.

11973, 14066.667, 8066.6665, 2920.

11974, 14066.667, 7600., 2920.

11975, 14066.667, 7133.3335, 2920.

11976, 14066.667, 6666.6665, 2920.

11977, 13600., 8533.33301, 2920.

11978, 13600., 8066.6665, 2920.

11979, 13600., 7600., 2920.

11980, 13600., 7133.3335, 2920.

11981, 13600., 6666.6665, 2920.

11982, 13133.333, 8533.33301, 2920.

11983, 13133.333, 8066.6665, 2920.

11984, 13133.333, 7600., 2920.

11985, 13133.333, 7133.3335, 2920.

11986, 13133.333, 6666.6665, 2920.

11987, 12666.667, 8533.33301, 2920.

11988, 12666.667, 8066.6665, 2920.

11989, 12666.667, 7600., 2920.

11990, 12666.667, 7133.3335, 2920.

11991, 12666.667, 6666.6665, 2920.

11992, 14533.333, 5533.3335, 2920.

11993, 14533.333, 5066.6665, 2920.

11994, 14533.333, 4600., 2920.

11995, 14533.333, 4133.3335, 2920.

11996, 14533.333, 3666.66675, 2920.

11997, 14066.667, 5533.3335, 2920.

11998, 14066.667, 5066.6665, 2920.

11999, 14066.667, 4600., 2920.

12000, 14066.667, 4133.3335, 2920.

12001, 14066.667, 3666.66675, 2920.

12002, 13600., 5533.3335, 2920.

12003, 13600., 5066.6665, 2920.

12004, 13600., 4600., 2920.

12005, 13600., 4133.3335, 2920.

12006, 13600., 3666.66675, 2920.

12007, 13133.333, 5533.3335, 2920.

12008, 13133.333, 5066.6665, 2920.

12009, 13133.333, 4600., 2920.

12010, 13133.333, 4133.3335, 2920.

12011, 13133.333, 3666.66675, 2920.

12012, 12666.667, 5533.3335, 2920.

12013, 12666.667, 5066.6665, 2920.

12014, 12666.667, 4600., 2920.

12015, 12666.667, 4133.3335, 2920.

12016, 12666.667, 3666.66675, 2920.

12017, 12666.667, 666.666687, 2920.

12018, 12666.667, 1133.33337, 2920.

12019, 12666.667, 1600., 2920.

12020, 12666.667, 2066.66675, 2920.

12021, 12666.667, 2533.33325, 2920.

12022, 13133.333, 666.666687, 2920.

12023, 13133.333, 1133.33337, 2920.

12024, 13133.333, 1600., 2920.

12025, 13133.333, 2066.66675, 2920.

12026, 13133.333, 2533.33325, 2920.

12027, 13600., 666.666687, 2920.

12028, 13600., 1133.33337, 2920.

12029, 13600., 1600., 2920.

12030, 13600., 2066.66675, 2920.

12031, 13600., 2533.33325, 2920.

12032, 14066.667, 666.666687, 2920.

12033, 14066.667, 1133.33337, 2920.

12034, 14066.667, 1600., 2920.

12035, 14066.667, 2066.66675, 2920.

12036, 14066.667, 2533.33325, 2920.

12037, 14533.333, 666.666687, 2920.

12038, 14533.333, 1133.33337, 2920.

12039, 14533.333, 1600., 2920.

12040, 14533.333, 2066.66675, 2920.

12041, 14533.333, 2533.33325, 2920.

12042, 12666.667, 15000., 3000.

12043, 13133.333, 15000., 3000.

12044, 13600., 15000., 3000.

12045, 14066.667, 15000., 3000.

12046, 14533.333, 15000., 3000.

12047, 12666.667, 14533.333, 3000.

12048, 13133.333, 14533.333, 3000.

12049, 13600., 14533.333, 3000.

12050, 14066.667, 14533.333, 3000.

12051, 14533.333, 14533.333, 3000.

12052, 12666.667, 14066.667, 3000.

12053, 13133.333, 14066.667, 3000.

12054, 13600., 14066.667, 3000.

12055, 14066.667, 14066.667, 3000.

12056, 14533.333, 14066.667, 3000.

12057, 12666.667, 13600., 3000.

12058, 13133.333, 13600., 3000.

12059, 13600., 13600., 3000.

12060, 14066.667, 13600., 3000.

12061, 14533.333, 13600., 3000.

12062, 12666.667, 13133.333, 3000.

12063, 13133.333, 13133.333, 3000.

12064, 13600., 13133.333, 3000.

12065, 14066.667, 13133.333, 3000.

12066, 14533.333, 13133.333, 3000.

12067, 12666.667, 12666.667, 3000.

12068, 13133.333, 12666.667, 3000.

12069, 13600., 12666.667, 3000.

12070, 14066.667, 12666.667, 3000.

12071, 14533.333, 12666.667, 3000.

12072, 12666.667, 12200., 3000.

12073, 13133.333, 12200., 3000.

12074, 13600., 12200., 3000.

12075, 14066.667, 12200., 3000.

12076, 14533.333, 12200., 3000.

12077, 12666.667, 12000., 3000.

12078, 13133.333, 12000., 3000.

12079, 13600., 12000., 3000.

12080, 14066.667, 12000., 3000.

12081, 14533.333, 12000., 3000.

12082, 12666.667, 11533.333, 3000.

12083, 13133.333, 11533.333, 3000.

12084, 13600., 11533.333, 3000.

12085, 14066.667, 11533.333, 3000.

12086, 14533.333, 11533.333, 3000.

12087, 12666.667, 11066.667, 3000.

12088, 13133.333, 11066.667, 3000.

12089, 13600., 11066.667, 3000.

12090, 14066.667, 11066.667, 3000.

12091, 14533.333, 11066.667, 3000.

12092, 12666.667, 10600., 3000.

12093, 13133.333, 10600., 3000.

12094, 13600., 10600., 3000.

12095, 14066.667, 10600., 3000.

12096, 14533.333, 10600., 3000.

12097, 12666.667, 10133.333, 3000.

12098, 13133.333, 10133.333, 3000.

12099, 13600., 10133.333, 3000.

12100, 14066.667, 10133.333, 3000.

12101, 14533.333, 10133.333, 3000.

12102, 12666.667, 9666.66699, 3000.

12103, 13133.333, 9666.66699, 3000.

12104, 13600., 9666.66699, 3000.

12105, 14066.667, 9666.66699, 3000.

12106, 14533.333, 9666.66699, 3000.

12107, 12666.667, 9200., 3000.

12108, 13133.333, 9200., 3000.

12109, 13600., 9200., 3000.

12110, 14066.667, 9200., 3000.

12111, 14533.333, 9200., 3000.

12112, 12666.667, 9000., 3000.

12113, 13133.333, 9000., 3000.

12114, 13600., 9000., 3000.

12115, 14066.667, 9000., 3000.

12116, 14533.333, 9000., 3000.

12117, 12666.667, 8533.33301, 3000.

12118, 13133.333, 8533.33301, 3000.

12119, 13600., 8533.33301, 3000.

12120, 14066.667, 8533.33301, 3000.

12121, 14533.333, 8533.33301, 3000.

12122, 12666.667, 8066.6665, 3000.

12123, 13133.333, 8066.6665, 3000.

12124, 13600., 8066.6665, 3000.

12125, 14066.667, 8066.6665, 3000.

12126, 14533.333, 8066.6665, 3000.

12127, 12666.667, 7600., 3000.

12128, 13133.333, 7600., 3000.

12129, 13600., 7600., 3000.

12130, 14066.667, 7600., 3000.

12131, 14533.333, 7600., 3000.

12132, 12666.667, 7133.3335, 3000.

12133, 13133.333, 7133.3335, 3000.

12134, 13600., 7133.3335, 3000.

12135, 14066.667, 7133.3335, 3000.

12136, 14533.333, 7133.3335, 3000.

12137, 12666.667, 6666.6665, 3000.

12138, 13133.333, 6666.6665, 3000.

12139, 13600., 6666.6665, 3000.

12140, 14066.667, 6666.6665, 3000.

12141, 14533.333, 6666.6665, 3000.

12142, 12666.667, 6200., 3000.

12143, 13133.333, 6200., 3000.

12144, 13600., 6200., 3000.

12145, 14066.667, 6200., 3000.

12146, 14533.333, 6200., 3000.

12147, 12666.667, 6000., 3000.

12148, 13133.333, 6000., 3000.

12149, 13600., 6000., 3000.

12150, 14066.667, 6000., 3000.

12151, 14533.333, 6000., 3000.

12152, 12666.667, 5533.3335, 3000.

12153, 13133.333, 5533.3335, 3000.

12154, 13600., 5533.3335, 3000.

12155, 14066.667, 5533.3335, 3000.

12156, 14533.333, 5533.3335, 3000.

12157, 12666.667, 5066.6665, 3000.

12158, 13133.333, 5066.6665, 3000.

12159, 13600., 5066.6665, 3000.

12160, 14066.667, 5066.6665, 3000.

12161, 14533.333, 5066.6665, 3000.

12162, 12666.667, 4600., 3000.

12163, 13133.333, 4600., 3000.

12164, 13600., 4600., 3000.

12165, 14066.667, 4600., 3000.

12166, 14533.333, 4600., 3000.

12167, 12666.667, 4133.3335, 3000.

12168, 13133.333, 4133.3335, 3000.

12169, 13600., 4133.3335, 3000.

12170, 14066.667, 4133.3335, 3000.

12171, 14533.333, 4133.3335, 3000.

12172, 12666.667, 3666.66675, 3000.

12173, 13133.333, 3666.66675, 3000.

12174, 13600., 3666.66675, 3000.

12175, 14066.667, 3666.66675, 3000.

12176, 14533.333, 3666.66675, 3000.

12177, 12666.667, 3200., 3000.

12178, 13133.333, 3200., 3000.

12179, 13600., 3200., 3000.

12180, 14066.667, 3200., 3000.

12181, 14533.333, 3200., 3000.

12182, 12666.667, 3000., 3000.

12183, 13133.333, 3000., 3000.

12184, 13600., 3000., 3000.

12185, 14066.667, 3000., 3000.

12186, 14533.333, 3000., 3000.

12187, 12666.667, 2533.33325, 3000.

12188, 13133.333, 2533.33325, 3000.

12189, 13600., 2533.33325, 3000.

12190, 14066.667, 2533.33325, 3000.

12191, 14533.333, 2533.33325, 3000.

12192, 12666.667, 2066.66675, 3000.

12193, 13133.333, 2066.66675, 3000.

12194, 13600., 2066.66675, 3000.

12195, 14066.667, 2066.66675, 3000.

12196, 14533.333, 2066.66675, 3000.

12197, 12666.667, 1600., 3000.

12198, 13133.333, 1600., 3000.

12199, 13600., 1600., 3000.

12200, 14066.667, 1600., 3000.

12201, 14533.333, 1600., 3000.

12202, 12666.667, 1133.33337, 3000.

12203, 13133.333, 1133.33337, 3000.

12204, 13600., 1133.33337, 3000.

12205, 14066.667, 1133.33337, 3000.

12206, 14533.333, 1133.33337, 3000.

12207, 12666.667, 666.666687, 3000.

12208, 13133.333, 666.666687, 3000.

12209, 13600., 666.666687, 3000.

12210, 14066.667, 666.666687, 3000.

12211, 14533.333, 666.666687, 3000.

12212, 12666.667, 200., 3000.

12213, 13133.333, 200., 3000.

12214, 13600., 200., 3000.

12215, 14066.667, 200., 3000.

12216, 14533.333, 200., 3000.

12217, 14533.333, 14533.333, 5920.

12218, 14533.333, 14066.667, 5920.

12219, 14533.333, 13600., 5920.

12220, 14533.333, 13133.333, 5920.

12221, 14533.333, 12666.667, 5920.

12222, 14066.667, 14533.333, 5920.

12223, 14066.667, 14066.667, 5920.

12224, 14066.667, 13600., 5920.

12225, 14066.667, 13133.333, 5920.

12226, 14066.667, 12666.667, 5920.

12227, 13600., 14533.333, 5920.

12228, 13600., 14066.667, 5920.

12229, 13600., 13600., 5920.

12230, 13600., 13133.333, 5920.

12231, 13600., 12666.667, 5920.

12232, 13133.333, 14533.333, 5920.

12233, 13133.333, 14066.667, 5920.

12234, 13133.333, 13600., 5920.

12235, 13133.333, 13133.333, 5920.

12236, 13133.333, 12666.667, 5920.

12237, 12666.667, 14533.333, 5920.

12238, 12666.667, 14066.667, 5920.

12239, 12666.667, 13600., 5920.

12240, 12666.667, 13133.333, 5920.

12241, 12666.667, 12666.667, 5920.

12242, 14533.333, 11533.333, 5920.

12243, 14533.333, 11066.667, 5920.

12244, 14533.333, 10600., 5920.

12245, 14533.333, 10133.333, 5920.

12246, 14533.333, 9666.66699, 5920.

12247, 14066.667, 11533.333, 5920.

12248, 14066.667, 11066.667, 5920.

12249, 14066.667, 10600., 5920.

12250, 14066.667, 10133.333, 5920.

12251, 14066.667, 9666.66699, 5920.

12252, 13600., 11533.333, 5920.

12253, 13600., 11066.667, 5920.

12254, 13600., 10600., 5920.

12255, 13600., 10133.333, 5920.

12256, 13600., 9666.66699, 5920.

12257, 13133.333, 11533.333, 5920.

12258, 13133.333, 11066.667, 5920.

12259, 13133.333, 10600., 5920.

12260, 13133.333, 10133.333, 5920.

12261, 13133.333, 9666.66699, 5920.

12262, 12666.667, 11533.333, 5920.

12263, 12666.667, 11066.667, 5920.

12264, 12666.667, 10600., 5920.

12265, 12666.667, 10133.333, 5920.

12266, 12666.667, 9666.66699, 5920.

12267, 14533.333, 8533.33301, 5920.

12268, 14533.333, 8066.6665, 5920.

12269, 14533.333, 7600., 5920.

12270, 14533.333, 7133.3335, 5920.

12271, 14533.333, 6666.6665, 5920.

12272, 14066.667, 8533.33301, 5920.

12273, 14066.667, 8066.6665, 5920.

12274, 14066.667, 7600., 5920.

12275, 14066.667, 7133.3335, 5920.

12276, 14066.667, 6666.6665, 5920.

12277, 13600., 8533.33301, 5920.

12278, 13600., 8066.6665, 5920.

12279, 13600., 7600., 5920.

12280, 13600., 7133.3335, 5920.

12281, 13600., 6666.6665, 5920.

12282, 13133.333, 8533.33301, 5920.

12283, 13133.333, 8066.6665, 5920.

12284, 13133.333, 7600., 5920.

12285, 13133.333, 7133.3335, 5920.

12286, 13133.333, 6666.6665, 5920.

12287, 12666.667, 8533.33301, 5920.

12288, 12666.667, 8066.6665, 5920.

12289, 12666.667, 7600., 5920.

12290, 12666.667, 7133.3335, 5920.

12291, 12666.667, 6666.6665, 5920.

12292, 14533.333, 5533.3335, 5920.

12293, 14533.333, 5066.6665, 5920.

12294, 14533.333, 4600., 5920.

12295, 14533.333, 4133.3335, 5920.

12296, 14533.333, 3666.66675, 5920.

12297, 14066.667, 5533.3335, 5920.

12298, 14066.667, 5066.6665, 5920.

12299, 14066.667, 4600., 5920.

12300, 14066.667, 4133.3335, 5920.

12301, 14066.667, 3666.66675, 5920.

12302, 13600., 5533.3335, 5920.

12303, 13600., 5066.6665, 5920.

12304, 13600., 4600., 5920.

12305, 13600., 4133.3335, 5920.

12306, 13600., 3666.66675, 5920.

12307, 13133.333, 5533.3335, 5920.

12308, 13133.333, 5066.6665, 5920.

12309, 13133.333, 4600., 5920.

12310, 13133.333, 4133.3335, 5920.

12311, 13133.333, 3666.66675, 5920.

12312, 12666.667, 5533.3335, 5920.

12313, 12666.667, 5066.6665, 5920.

12314, 12666.667, 4600., 5920.

12315, 12666.667, 4133.3335, 5920.

12316, 12666.667, 3666.66675, 5920.

12317, 14533.333, 2533.33325, 5920.

12318, 14533.333, 2066.66675, 5920.

12319, 14533.333, 1600., 5920.

12320, 14533.333, 1133.33337, 5920.

12321, 14533.333, 666.666687, 5920.

12322, 14066.667, 2533.33325, 5920.

12323, 14066.667, 2066.66675, 5920.

12324, 14066.667, 1600., 5920.

12325, 14066.667, 1133.33337, 5920.

12326, 14066.667, 666.666687, 5920.

12327, 13600., 2533.33325, 5920.

12328, 13600., 2066.66675, 5920.

12329, 13600., 1600., 5920.

12330, 13600., 1133.33337, 5920.

12331, 13600., 666.666687, 5920.

12332, 13133.333, 2533.33325, 5920.

12333, 13133.333, 2066.66675, 5920.

12334, 13133.333, 1600., 5920.

12335, 13133.333, 1133.33337, 5920.

12336, 13133.333, 666.666687, 5920.

12337, 12666.667, 2533.33325, 5920.

12338, 12666.667, 2066.66675, 5920.

12339, 12666.667, 1600., 5920.

12340, 12666.667, 1133.33337, 5920.

12341, 12666.667, 666.666687, 5920.

12342, 12666.667, 15000., 6000.

12343, 13133.333, 15000., 6000.

12344, 13600., 15000., 6000.

12345, 14066.667, 15000., 6000.

12346, 14533.333, 15000., 6000.

12347, 12666.667, 14533.333, 6000.

12348, 13133.333, 14533.333, 6000.

12349, 13600., 14533.333, 6000.

12350, 14066.667, 14533.333, 6000.

12351, 14533.333, 14533.333, 6000.

12352, 12666.667, 14066.667, 6000.

12353, 13133.333, 14066.667, 6000.

12354, 13600., 14066.667, 6000.

12355, 14066.667, 14066.667, 6000.

12356, 14533.333, 14066.667, 6000.

12357, 12666.667, 13600., 6000.

12358, 13133.333, 13600., 6000.

12359, 13600., 13600., 6000.

12360, 14066.667, 13600., 6000.

12361, 14533.333, 13600., 6000.

12362, 12666.667, 13133.333, 6000.

12363, 13133.333, 13133.333, 6000.

12364, 13600., 13133.333, 6000.

12365, 14066.667, 13133.333, 6000.

12366, 14533.333, 13133.333, 6000.

12367, 12666.667, 12666.667, 6000.

12368, 13133.333, 12666.667, 6000.

12369, 13600., 12666.667, 6000.

12370, 14066.667, 12666.667, 6000.

12371, 14533.333, 12666.667, 6000.

12372, 12666.667, 12200., 6000.

12373, 13133.333, 12200., 6000.

12374, 13600., 12200., 6000.

12375, 14066.667, 12200., 6000.

12376, 14533.333, 12200., 6000.

12377, 12666.667, 12000., 6000.

12378, 13133.333, 12000., 6000.

12379, 13600., 12000., 6000.

12380, 14066.667, 12000., 6000.

12381, 14533.333, 12000., 6000.

12382, 12666.667, 11533.333, 6000.

12383, 13133.333, 11533.333, 6000.

12384, 13600., 11533.333, 6000.

12385, 14066.667, 11533.333, 6000.

12386, 14533.333, 11533.333, 6000.

12387, 12666.667, 11066.667, 6000.

12388, 13133.333, 11066.667, 6000.

12389, 13600., 11066.667, 6000.

12390, 14066.667, 11066.667, 6000.

12391, 14533.333, 11066.667, 6000.

12392, 12666.667, 10600., 6000.

12393, 13133.333, 10600., 6000.

12394, 13600., 10600., 6000.

12395, 14066.667, 10600., 6000.

12396, 14533.333, 10600., 6000.

12397, 12666.667, 10133.333, 6000.

12398, 13133.333, 10133.333, 6000.

12399, 13600., 10133.333, 6000.

12400, 14066.667, 10133.333, 6000.

12401, 14533.333, 10133.333, 6000.

12402, 12666.667, 9666.66699, 6000.

12403, 13133.333, 9666.66699, 6000.

12404, 13600., 9666.66699, 6000.

12405, 14066.667, 9666.66699, 6000.

12406, 14533.333, 9666.66699, 6000.

12407, 12666.667, 9200., 6000.

12408, 13133.333, 9200., 6000.

12409, 13600., 9200., 6000.

12410, 14066.667, 9200., 6000.

12411, 14533.333, 9200., 6000.

12412, 12666.667, 9000., 6000.

12413, 13133.333, 9000., 6000.

12414, 13600., 9000., 6000.

12415, 14066.667, 9000., 6000.

12416, 14533.333, 9000., 6000.

12417, 12666.667, 8533.33301, 6000.

12418, 13133.333, 8533.33301, 6000.

12419, 13600., 8533.33301, 6000.

12420, 14066.667, 8533.33301, 6000.

12421, 14533.333, 8533.33301, 6000.

12422, 12666.667, 8066.6665, 6000.

12423, 13133.333, 8066.6665, 6000.

12424, 13600., 8066.6665, 6000.

12425, 14066.667, 8066.6665, 6000.

12426, 14533.333, 8066.6665, 6000.

12427, 12666.667, 7600., 6000.

12428, 13133.333, 7600., 6000.

12429, 13600., 7600., 6000.

12430, 14066.667, 7600., 6000.

12431, 14533.333, 7600., 6000.

12432, 12666.667, 7133.3335, 6000.

12433, 13133.333, 7133.3335, 6000.

12434, 13600., 7133.3335, 6000.

12435, 14066.667, 7133.3335, 6000.

12436, 14533.333, 7133.3335, 6000.

12437, 12666.667, 6666.6665, 6000.

12438, 13133.333, 6666.6665, 6000.

12439, 13600., 6666.6665, 6000.

12440, 14066.667, 6666.6665, 6000.

12441, 14533.333, 6666.6665, 6000.

12442, 12666.667, 6200., 6000.

12443, 13133.333, 6200., 6000.

12444, 13600., 6200., 6000.

12445, 14066.667, 6200., 6000.

12446, 14533.333, 6200., 6000.

12447, 12666.667, 6000., 6000.

12448, 13133.333, 6000., 6000.

12449, 13600., 6000., 6000.

12450, 14066.667, 6000., 6000.

12451, 14533.333, 6000., 6000.

12452, 12666.667, 5533.3335, 6000.

12453, 13133.333, 5533.3335, 6000.

12454, 13600., 5533.3335, 6000.

12455, 14066.667, 5533.3335, 6000.

12456, 14533.333, 5533.3335, 6000.

12457, 12666.667, 5066.6665, 6000.

12458, 13133.333, 5066.6665, 6000.

12459, 13600., 5066.6665, 6000.

12460, 14066.667, 5066.6665, 6000.

12461, 14533.333, 5066.6665, 6000.

12462, 12666.667, 4600., 6000.

12463, 13133.333, 4600., 6000.

12464, 13600., 4600., 6000.

12465, 14066.667, 4600., 6000.

12466, 14533.333, 4600., 6000.

12467, 12666.667, 4133.3335, 6000.

12468, 13133.333, 4133.3335, 6000.

12469, 13600., 4133.3335, 6000.

12470, 14066.667, 4133.3335, 6000.

12471, 14533.333, 4133.3335, 6000.

12472, 12666.667, 3666.66675, 6000.

12473, 13133.333, 3666.66675, 6000.

12474, 13600., 3666.66675, 6000.

12475, 14066.667, 3666.66675, 6000.

12476, 14533.333, 3666.66675, 6000.

12477, 12666.667, 3200., 6000.

12478, 13133.333, 3200., 6000.

12479, 13600., 3200., 6000.

12480, 14066.667, 3200., 6000.

12481, 14533.333, 3200., 6000.

12482, 12666.667, 3000., 6000.

12483, 13133.333, 3000., 6000.

12484, 13600., 3000., 6000.

12485, 14066.667, 3000., 6000.

12486, 14533.333, 3000., 6000.

12487, 12666.667, 2533.33325, 6000.

12488, 13133.333, 2533.33325, 6000.

12489, 13600., 2533.33325, 6000.

12490, 14066.667, 2533.33325, 6000.

12491, 14533.333, 2533.33325, 6000.

12492, 12666.667, 2066.66675, 6000.

12493, 13133.333, 2066.66675, 6000.

12494, 13600., 2066.66675, 6000.

12495, 14066.667, 2066.66675, 6000.

12496, 14533.333, 2066.66675, 6000.

12497, 12666.667, 1600., 6000.

12498, 13133.333, 1600., 6000.

12499, 13600., 1600., 6000.

12500, 14066.667, 1600., 6000.

12501, 14533.333, 1600., 6000.

12502, 12666.667, 1133.33337, 6000.

12503, 13133.333, 1133.33337, 6000.

12504, 13600., 1133.33337, 6000.

12505, 14066.667, 1133.33337, 6000.

12506, 14533.333, 1133.33337, 6000.

12507, 12666.667, 666.666687, 6000.

12508, 13133.333, 666.666687, 6000.

12509, 13600., 666.666687, 6000.

12510, 14066.667, 666.666687, 6000.

12511, 14533.333, 666.666687, 6000.

12512, 12666.667, 200., 6000.

12513, 13133.333, 200., 6000.

12514, 13600., 200., 6000.

12515, 14066.667, 200., 6000.

12516, 14533.333, 200., 6000.

12517, 17533.334, 2533.33325, 5920.

12518, 17533.334, 2066.66675, 5920.

12519, 17533.334, 1600., 5920.

12520, 17533.334, 1133.33337, 5920.

12521, 17533.334, 666.666687, 5920.

12522, 17066.666, 2533.33325, 5920.

12523, 17066.666, 2066.66675, 5920.

12524, 17066.666, 1600., 5920.

12525, 17066.666, 1133.33337, 5920.

12526, 17066.666, 666.666687, 5920.

12527, 16600., 2533.33325, 5920.

12528, 16600., 2066.66675, 5920.

12529, 16600., 1600., 5920.

12530, 16600., 1133.33337, 5920.

12531, 16600., 666.666687, 5920.

12532, 16133.333, 2533.33325, 5920.

12533, 16133.333, 2066.66675, 5920.

12534, 16133.333, 1600., 5920.

12535, 16133.333, 1133.33337, 5920.

12536, 16133.333, 666.666687, 5920.

12537, 15666.667, 2533.33325, 5920.

12538, 15666.667, 2066.66675, 5920.

12539, 15666.667, 1600., 5920.

12540, 15666.667, 1133.33337, 5920.

12541, 15666.667, 666.666687, 5920.

12542, 17533.334, 5533.3335, 5920.

12543, 17533.334, 5066.6665, 5920.

12544, 17533.334, 4600., 5920.

12545, 17533.334, 4133.3335, 5920.

12546, 17533.334, 3666.66675, 5920.

12547, 17066.666, 5533.3335, 5920.

12548, 17066.666, 5066.6665, 5920.

12549, 17066.666, 4600., 5920.

12550, 17066.666, 4133.3335, 5920.

12551, 17066.666, 3666.66675, 5920.

12552, 16600., 5533.3335, 5920.

12553, 16600., 5066.6665, 5920.

12554, 16600., 4600., 5920.

12555, 16600., 4133.3335, 5920.

12556, 16600., 3666.66675, 5920.

12557, 16133.333, 5533.3335, 5920.

12558, 16133.333, 5066.6665, 5920.

12559, 16133.333, 4600., 5920.

12560, 16133.333, 4133.3335, 5920.

12561, 16133.333, 3666.66675, 5920.

12562, 15666.667, 5533.3335, 5920.

12563, 15666.667, 5066.6665, 5920.

12564, 15666.667, 4600., 5920.

12565, 15666.667, 4133.3335, 5920.

12566, 15666.667, 3666.66675, 5920.

12567, 17533.334, 8533.33301, 5920.

12568, 17533.334, 8066.6665, 5920.

12569, 17533.334, 7600., 5920.

12570, 17533.334, 7133.3335, 5920.

12571, 17533.334, 6666.6665, 5920.

12572, 17066.666, 8533.33301, 5920.

12573, 17066.666, 8066.6665, 5920.

12574, 17066.666, 7600., 5920.

12575, 17066.666, 7133.3335, 5920.

12576, 17066.666, 6666.6665, 5920.

12577, 16600., 8533.33301, 5920.

12578, 16600., 8066.6665, 5920.

12579, 16600., 7600., 5920.

12580, 16600., 7133.3335, 5920.

12581, 16600., 6666.6665, 5920.

12582, 16133.333, 8533.33301, 5920.

12583, 16133.333, 8066.6665, 5920.

12584, 16133.333, 7600., 5920.

12585, 16133.333, 7133.3335, 5920.

12586, 16133.333, 6666.6665, 5920.

12587, 15666.667, 8533.33301, 5920.

12588, 15666.667, 8066.6665, 5920.

12589, 15666.667, 7600., 5920.

12590, 15666.667, 7133.3335, 5920.

12591, 15666.667, 6666.6665, 5920.

12592, 17533.334, 11533.333, 5920.

12593, 17533.334, 11066.667, 5920.

12594, 17533.334, 10600., 5920.

12595, 17533.334, 10133.333, 5920.

12596, 17533.334, 9666.66699, 5920.

12597, 17066.666, 11533.333, 5920.

12598, 17066.666, 11066.667, 5920.

12599, 17066.666, 10600., 5920.

12600, 17066.666, 10133.333, 5920.

12601, 17066.666, 9666.66699, 5920.

12602, 16600., 11533.333, 5920.

12603, 16600., 11066.667, 5920.

12604, 16600., 10600., 5920.

12605, 16600., 10133.333, 5920.

12606, 16600., 9666.66699, 5920.

12607, 16133.333, 11533.333, 5920.

12608, 16133.333, 11066.667, 5920.

12609, 16133.333, 10600., 5920.

12610, 16133.333, 10133.333, 5920.

12611, 16133.333, 9666.66699, 5920.

12612, 15666.667, 11533.333, 5920.

12613, 15666.667, 11066.667, 5920.

12614, 15666.667, 10600., 5920.

12615, 15666.667, 10133.333, 5920.

12616, 15666.667, 9666.66699, 5920.

12617, 17533.334, 14533.333, 5920.

12618, 17533.334, 14066.667, 5920.

12619, 17533.334, 13600., 5920.

12620, 17533.334, 13133.333, 5920.

12621, 17533.334, 12666.667, 5920.

12622, 17066.666, 14533.333, 5920.

12623, 17066.666, 14066.667, 5920.

12624, 17066.666, 13600., 5920.

12625, 17066.666, 13133.333, 5920.

12626, 17066.666, 12666.667, 5920.

12627, 16600., 14533.333, 5920.

12628, 16600., 14066.667, 5920.

12629, 16600., 13600., 5920.

12630, 16600., 13133.333, 5920.

12631, 16600., 12666.667, 5920.

12632, 16133.333, 14533.333, 5920.

12633, 16133.333, 14066.667, 5920.

12634, 16133.333, 13600., 5920.

12635, 16133.333, 13133.333, 5920.

12636, 16133.333, 12666.667, 5920.

12637, 15666.667, 14533.333, 5920.

12638, 15666.667, 14066.667, 5920.

12639, 15666.667, 13600., 5920.

12640, 15666.667, 13133.333, 5920.

12641, 15666.667, 12666.667, 5920.

12642, 17533.334, 200., 6000.

12643, 17066.666, 200., 6000.

12644, 16600., 200., 6000.

12645, 16133.333, 200., 6000.

12646, 15666.667, 200., 6000.

12647, 17533.334, 666.666687, 6000.

12648, 17066.666, 666.666687, 6000.

12649, 16600., 666.666687, 6000.

12650, 16133.333, 666.666687, 6000.

12651, 15666.667, 666.666687, 6000.

12652, 17533.334, 1133.33337, 6000.

12653, 17066.666, 1133.33337, 6000.

12654, 16600., 1133.33337, 6000.

12655, 16133.333, 1133.33337, 6000.

12656, 15666.667, 1133.33337, 6000.

12657, 17533.334, 1600., 6000.

12658, 17066.666, 1600., 6000.

12659, 16600., 1600., 6000.

12660, 16133.333, 1600., 6000.

12661, 15666.667, 1600., 6000.

12662, 17533.334, 2066.66675, 6000.

12663, 17066.666, 2066.66675, 6000.

12664, 16600., 2066.66675, 6000.

12665, 16133.333, 2066.66675, 6000.

12666, 15666.667, 2066.66675, 6000.

12667, 17533.334, 2533.33325, 6000.

12668, 17066.666, 2533.33325, 6000.

12669, 16600., 2533.33325, 6000.

12670, 16133.333, 2533.33325, 6000.

12671, 15666.667, 2533.33325, 6000.

12672, 17533.334, 3000., 6000.

12673, 17066.666, 3000., 6000.

12674, 16600., 3000., 6000.

12675, 16133.333, 3000., 6000.

12676, 15666.667, 3000., 6000.

12677, 17533.334, 3200., 6000.

12678, 17066.666, 3200., 6000.

12679, 16600., 3200., 6000.

12680, 16133.333, 3200., 6000.

12681, 15666.667, 3200., 6000.

12682, 17533.334, 3666.66675, 6000.

12683, 17066.666, 3666.66675, 6000.

12684, 16600., 3666.66675, 6000.

12685, 16133.333, 3666.66675, 6000.

12686, 15666.667, 3666.66675, 6000.

12687, 17533.334, 4133.3335, 6000.

12688, 17066.666, 4133.3335, 6000.

12689, 16600., 4133.3335, 6000.

12690, 16133.333, 4133.3335, 6000.

12691, 15666.667, 4133.3335, 6000.

12692, 17533.334, 4600., 6000.

12693, 17066.666, 4600., 6000.

12694, 16600., 4600., 6000.

12695, 16133.333, 4600., 6000.

12696, 15666.667, 4600., 6000.

12697, 17533.334, 5066.6665, 6000.

12698, 17066.666, 5066.6665, 6000.

12699, 16600., 5066.6665, 6000.

12700, 16133.333, 5066.6665, 6000.

12701, 15666.667, 5066.6665, 6000.

12702, 17533.334, 5533.3335, 6000.

12703, 17066.666, 5533.3335, 6000.

12704, 16600., 5533.3335, 6000.

12705, 16133.333, 5533.3335, 6000.

12706, 15666.667, 5533.3335, 6000.

12707, 17533.334, 6000., 6000.

12708, 17066.666, 6000., 6000.

12709, 16600., 6000., 6000.

12710, 16133.333, 6000., 6000.

12711, 15666.667, 6000., 6000.

12712, 17533.334, 6200., 6000.

12713, 17066.666, 6200., 6000.

12714, 16600., 6200., 6000.

12715, 16133.333, 6200., 6000.

12716, 15666.667, 6200., 6000.

12717, 17533.334, 6666.6665, 6000.

12718, 17066.666, 6666.6665, 6000.

12719, 16600., 6666.6665, 6000.

12720, 16133.333, 6666.6665, 6000.

12721, 15666.667, 6666.6665, 6000.

12722, 17533.334, 7133.3335, 6000.

12723, 17066.666, 7133.3335, 6000.

12724, 16600., 7133.3335, 6000.

12725, 16133.333, 7133.3335, 6000.

12726, 15666.667, 7133.3335, 6000.

12727, 17533.334, 7600., 6000.

12728, 17066.666, 7600., 6000.

12729, 16600., 7600., 6000.

12730, 16133.333, 7600., 6000.

12731, 15666.667, 7600., 6000.

12732, 17533.334, 8066.6665, 6000.

12733, 17066.666, 8066.6665, 6000.

12734, 16600., 8066.6665, 6000.

12735, 16133.333, 8066.6665, 6000.

12736, 15666.667, 8066.6665, 6000.

12737, 17533.334, 8533.33301, 6000.

12738, 17066.666, 8533.33301, 6000.

12739, 16600., 8533.33301, 6000.

12740, 16133.333, 8533.33301, 6000.

12741, 15666.667, 8533.33301, 6000.

12742, 17533.334, 9000., 6000.

12743, 17066.666, 9000., 6000.

12744, 16600., 9000., 6000.

12745, 16133.333, 9000., 6000.

12746, 15666.667, 9000., 6000.

12747, 17533.334, 9200., 6000.

12748, 17066.666, 9200., 6000.

12749, 16600., 9200., 6000.

12750, 16133.333, 9200., 6000.

12751, 15666.667, 9200., 6000.

12752, 17533.334, 9666.66699, 6000.

12753, 17066.666, 9666.66699, 6000.

12754, 16600., 9666.66699, 6000.

12755, 16133.333, 9666.66699, 6000.

12756, 15666.667, 9666.66699, 6000.

12757, 17533.334, 10133.333, 6000.

12758, 17066.666, 10133.333, 6000.

12759, 16600., 10133.333, 6000.

12760, 16133.333, 10133.333, 6000.

12761, 15666.667, 10133.333, 6000.

12762, 17533.334, 10600., 6000.

12763, 17066.666, 10600., 6000.

12764, 16600., 10600., 6000.

12765, 16133.333, 10600., 6000.

12766, 15666.667, 10600., 6000.

12767, 17533.334, 11066.667, 6000.

12768, 17066.666, 11066.667, 6000.

12769, 16600., 11066.667, 6000.

12770, 16133.333, 11066.667, 6000.

12771, 15666.667, 11066.667, 6000.

12772, 17533.334, 11533.333, 6000.

12773, 17066.666, 11533.333, 6000.

12774, 16600., 11533.333, 6000.

12775, 16133.333, 11533.333, 6000.

12776, 15666.667, 11533.333, 6000.

12777, 17533.334, 12000., 6000.

12778, 17066.666, 12000., 6000.

12779, 16600., 12000., 6000.

12780, 16133.333, 12000., 6000.

12781, 15666.667, 12000., 6000.

12782, 17533.334, 12200., 6000.

12783, 17066.666, 12200., 6000.

12784, 16600., 12200., 6000.

12785, 16133.333, 12200., 6000.

12786, 15666.667, 12200., 6000.

12787, 17533.334, 12666.667, 6000.

12788, 17066.666, 12666.667, 6000.

12789, 16600., 12666.667, 6000.

12790, 16133.333, 12666.667, 6000.

12791, 15666.667, 12666.667, 6000.

12792, 17533.334, 13133.333, 6000.

12793, 17066.666, 13133.333, 6000.

12794, 16600., 13133.333, 6000.

12795, 16133.333, 13133.333, 6000.

12796, 15666.667, 13133.333, 6000.

12797, 17533.334, 13600., 6000.

12798, 17066.666, 13600., 6000.

12799, 16600., 13600., 6000.

12800, 16133.333, 13600., 6000.

12801, 15666.667, 13600., 6000.

12802, 17533.334, 14066.667, 6000.

12803, 17066.666, 14066.667, 6000.

12804, 16600., 14066.667, 6000.

12805, 16133.333, 14066.667, 6000.

12806, 15666.667, 14066.667, 6000.

12807, 17533.334, 14533.333, 6000.

12808, 17066.666, 14533.333, 6000.

12809, 16600., 14533.333, 6000.

12810, 16133.333, 14533.333, 6000.

12811, 15666.667, 14533.333, 6000.

12812, 17533.334, 15000., 6000.

12813, 17066.666, 15000., 6000.

12814, 16600., 15000., 6000.

12815, 16133.333, 15000., 6000.

12816, 15666.667, 15000., 6000.

12817, 17533.334, 14533.333, 2920.

12818, 17533.334, 14066.667, 2920.

12819, 17533.334, 13600., 2920.

12820, 17533.334, 13133.333, 2920.

12821, 17533.334, 12666.667, 2920.

12822, 17066.666, 14533.333, 2920.

12823, 17066.666, 14066.667, 2920.

12824, 17066.666, 13600., 2920.

12825, 17066.666, 13133.333, 2920.

12826, 17066.666, 12666.667, 2920.

12827, 16600., 14533.333, 2920.

12828, 16600., 14066.667, 2920.

12829, 16600., 13600., 2920.

12830, 16600., 13133.333, 2920.

12831, 16600., 12666.667, 2920.

12832, 16133.333, 14533.333, 2920.

12833, 16133.333, 14066.667, 2920.

12834, 16133.333, 13600., 2920.

12835, 16133.333, 13133.333, 2920.

12836, 16133.333, 12666.667, 2920.

12837, 15666.667, 14533.333, 2920.

12838, 15666.667, 14066.667, 2920.

12839, 15666.667, 13600., 2920.

12840, 15666.667, 13133.333, 2920.

12841, 15666.667, 12666.667, 2920.

12842, 17533.334, 11533.333, 2920.

12843, 17533.334, 11066.667, 2920.

12844, 17533.334, 10600., 2920.

12845, 17533.334, 10133.333, 2920.

12846, 17533.334, 9666.66699, 2920.

12847, 17066.666, 11533.333, 2920.

12848, 17066.666, 11066.667, 2920.

12849, 17066.666, 10600., 2920.

12850, 17066.666, 10133.333, 2920.

12851, 17066.666, 9666.66699, 2920.

12852, 16600., 11533.333, 2920.

12853, 16600., 11066.667, 2920.

12854, 16600., 10600., 2920.

12855, 16600., 10133.333, 2920.

12856, 16600., 9666.66699, 2920.

12857, 16133.333, 11533.333, 2920.

12858, 16133.333, 11066.667, 2920.

12859, 16133.333, 10600., 2920.

12860, 16133.333, 10133.333, 2920.

12861, 16133.333, 9666.66699, 2920.

12862, 15666.667, 11533.333, 2920.

12863, 15666.667, 11066.667, 2920.

12864, 15666.667, 10600., 2920.

12865, 15666.667, 10133.333, 2920.

12866, 15666.667, 9666.66699, 2920.

12867, 17533.334, 8533.33301, 2920.

12868, 17533.334, 8066.6665, 2920.

12869, 17533.334, 7600., 2920.

12870, 17533.334, 7133.3335, 2920.

12871, 17533.334, 6666.6665, 2920.

12872, 17066.666, 8533.33301, 2920.

12873, 17066.666, 8066.6665, 2920.

12874, 17066.666, 7600., 2920.

12875, 17066.666, 7133.3335, 2920.

12876, 17066.666, 6666.6665, 2920.

12877, 16600., 8533.33301, 2920.

12878, 16600., 8066.6665, 2920.

12879, 16600., 7600., 2920.

12880, 16600., 7133.3335, 2920.

12881, 16600., 6666.6665, 2920.

12882, 16133.333, 8533.33301, 2920.

12883, 16133.333, 8066.6665, 2920.

12884, 16133.333, 7600., 2920.

12885, 16133.333, 7133.3335, 2920.

12886, 16133.333, 6666.6665, 2920.

12887, 15666.667, 8533.33301, 2920.

12888, 15666.667, 8066.6665, 2920.

12889, 15666.667, 7600., 2920.

12890, 15666.667, 7133.3335, 2920.

12891, 15666.667, 6666.6665, 2920.

12892, 17533.334, 5533.3335, 2920.

12893, 17533.334, 5066.6665, 2920.

12894, 17533.334, 4600., 2920.

12895, 17533.334, 4133.3335, 2920.

12896, 17533.334, 3666.66675, 2920.

12897, 17066.666, 5533.3335, 2920.

12898, 17066.666, 5066.6665, 2920.

12899, 17066.666, 4600., 2920.

12900, 17066.666, 4133.3335, 2920.

12901, 17066.666, 3666.66675, 2920.

12902, 16600., 5533.3335, 2920.

12903, 16600., 5066.6665, 2920.

12904, 16600., 4600., 2920.

12905, 16600., 4133.3335, 2920.

12906, 16600., 3666.66675, 2920.

12907, 16133.333, 5533.3335, 2920.

12908, 16133.333, 5066.6665, 2920.

12909, 16133.333, 4600., 2920.

12910, 16133.333, 4133.3335, 2920.

12911, 16133.333, 3666.66675, 2920.

12912, 15666.667, 5533.3335, 2920.

12913, 15666.667, 5066.6665, 2920.

12914, 15666.667, 4600., 2920.

12915, 15666.667, 4133.3335, 2920.

12916, 15666.667, 3666.66675, 2920.

12917, 15666.667, 666.666687, 2920.

12918, 15666.667, 1133.33337, 2920.

12919, 15666.667, 1600., 2920.

12920, 15666.667, 2066.66675, 2920.

12921, 15666.667, 2533.33325, 2920.

12922, 16133.333, 666.666687, 2920.

12923, 16133.333, 1133.33337, 2920.

12924, 16133.333, 1600., 2920.

12925, 16133.333, 2066.66675, 2920.

12926, 16133.333, 2533.33325, 2920.

12927, 16600., 666.666687, 2920.

12928, 16600., 1133.33337, 2920.

12929, 16600., 1600., 2920.

12930, 16600., 2066.66675, 2920.

12931, 16600., 2533.33325, 2920.

12932, 17066.666, 666.666687, 2920.

12933, 17066.666, 1133.33337, 2920.

12934, 17066.666, 1600., 2920.

12935, 17066.666, 2066.66675, 2920.

12936, 17066.666, 2533.33325, 2920.

12937, 17533.334, 666.666687, 2920.

12938, 17533.334, 1133.33337, 2920.

12939, 17533.334, 1600., 2920.

12940, 17533.334, 2066.66675, 2920.

12941, 17533.334, 2533.33325, 2920.

12942, 15666.667, 15000., 3000.

12943, 16133.333, 15000., 3000.

12944, 16600., 15000., 3000.

12945, 17066.666, 15000., 3000.

12946, 17533.334, 15000., 3000.

12947, 15666.667, 14533.333, 3000.

12948, 16133.333, 14533.333, 3000.

12949, 16600., 14533.333, 3000.

12950, 17066.666, 14533.333, 3000.

12951, 17533.334, 14533.333, 3000.

12952, 15666.667, 14066.667, 3000.

12953, 16133.333, 14066.667, 3000.

12954, 16600., 14066.667, 3000.

12955, 17066.666, 14066.667, 3000.

12956, 17533.334, 14066.667, 3000.

12957, 15666.667, 13600., 3000.

12958, 16133.333, 13600., 3000.

12959, 16600., 13600., 3000.

12960, 17066.666, 13600., 3000.

12961, 17533.334, 13600., 3000.

12962, 15666.667, 13133.333, 3000.

12963, 16133.333, 13133.333, 3000.

12964, 16600., 13133.333, 3000.

12965, 17066.666, 13133.333, 3000.

12966, 17533.334, 13133.333, 3000.

12967, 15666.667, 12666.667, 3000.

12968, 16133.333, 12666.667, 3000.

12969, 16600., 12666.667, 3000.

12970, 17066.666, 12666.667, 3000.

12971, 17533.334, 12666.667, 3000.

12972, 15666.667, 12200., 3000.

12973, 16133.333, 12200., 3000.

12974, 16600., 12200., 3000.

12975, 17066.666, 12200., 3000.

12976, 17533.334, 12200., 3000.

12977, 15666.667, 12000., 3000.

12978, 16133.333, 12000., 3000.

12979, 16600., 12000., 3000.

12980, 17066.666, 12000., 3000.

12981, 17533.334, 12000., 3000.

12982, 15666.667, 11533.333, 3000.

12983, 16133.333, 11533.333, 3000.

12984, 16600., 11533.333, 3000.

12985, 17066.666, 11533.333, 3000.

12986, 17533.334, 11533.333, 3000.

12987, 15666.667, 11066.667, 3000.

12988, 16133.333, 11066.667, 3000.

12989, 16600., 11066.667, 3000.

12990, 17066.666, 11066.667, 3000.

12991, 17533.334, 11066.667, 3000.

12992, 15666.667, 10600., 3000.

12993, 16133.333, 10600., 3000.

12994, 16600., 10600., 3000.

12995, 17066.666, 10600., 3000.

12996, 17533.334, 10600., 3000.

12997, 15666.667, 10133.333, 3000.

12998, 16133.333, 10133.333, 3000.

12999, 16600., 10133.333, 3000.

13000, 17066.666, 10133.333, 3000.

13001, 17533.334, 10133.333, 3000.

13002, 15666.667, 9666.66699, 3000.

13003, 16133.333, 9666.66699, 3000.

13004, 16600., 9666.66699, 3000.

13005, 17066.666, 9666.66699, 3000.

13006, 17533.334, 9666.66699, 3000.

13007, 15666.667, 9200., 3000.

13008, 16133.333, 9200., 3000.

13009, 16600., 9200., 3000.

13010, 17066.666, 9200., 3000.

13011, 17533.334, 9200., 3000.

13012, 15666.667, 9000., 3000.

13013, 16133.333, 9000., 3000.

13014, 16600., 9000., 3000.

13015, 17066.666, 9000., 3000.

13016, 17533.334, 9000., 3000.

13017, 15666.667, 8533.33301, 3000.

13018, 16133.333, 8533.33301, 3000.

13019, 16600., 8533.33301, 3000.

13020, 17066.666, 8533.33301, 3000.

13021, 17533.334, 8533.33301, 3000.

13022, 15666.667, 8066.6665, 3000.

13023, 16133.333, 8066.6665, 3000.

13024, 16600., 8066.6665, 3000.

13025, 17066.666, 8066.6665, 3000.

13026, 17533.334, 8066.6665, 3000.

13027, 15666.667, 7600., 3000.

13028, 16133.333, 7600., 3000.

13029, 16600., 7600., 3000.

13030, 17066.666, 7600., 3000.

13031, 17533.334, 7600., 3000.

13032, 15666.667, 7133.3335, 3000.

13033, 16133.333, 7133.3335, 3000.

13034, 16600., 7133.3335, 3000.

13035, 17066.666, 7133.3335, 3000.

13036, 17533.334, 7133.3335, 3000.

13037, 15666.667, 6666.6665, 3000.

13038, 16133.333, 6666.6665, 3000.

13039, 16600., 6666.6665, 3000.

13040, 17066.666, 6666.6665, 3000.

13041, 17533.334, 6666.6665, 3000.

13042, 15666.667, 6200., 3000.

13043, 16133.333, 6200., 3000.

13044, 16600., 6200., 3000.

13045, 17066.666, 6200., 3000.

13046, 17533.334, 6200., 3000.

13047, 15666.667, 6000., 3000.

13048, 16133.333, 6000., 3000.

13049, 16600., 6000., 3000.

13050, 17066.666, 6000., 3000.

13051, 17533.334, 6000., 3000.

13052, 15666.667, 5533.3335, 3000.

13053, 16133.333, 5533.3335, 3000.

13054, 16600., 5533.3335, 3000.

13055, 17066.666, 5533.3335, 3000.

13056, 17533.334, 5533.3335, 3000.

13057, 15666.667, 5066.6665, 3000.

13058, 16133.333, 5066.6665, 3000.

13059, 16600., 5066.6665, 3000.

13060, 17066.666, 5066.6665, 3000.

13061, 17533.334, 5066.6665, 3000.

13062, 15666.667, 4600., 3000.

13063, 16133.333, 4600., 3000.

13064, 16600., 4600., 3000.

13065, 17066.666, 4600., 3000.

13066, 17533.334, 4600., 3000.

13067, 15666.667, 4133.3335, 3000.

13068, 16133.333, 4133.3335, 3000.

13069, 16600., 4133.3335, 3000.

13070, 17066.666, 4133.3335, 3000.

13071, 17533.334, 4133.3335, 3000.

13072, 15666.667, 3666.66675, 3000.

13073, 16133.333, 3666.66675, 3000.

13074, 16600., 3666.66675, 3000.

13075, 17066.666, 3666.66675, 3000.

13076, 17533.334, 3666.66675, 3000.

13077, 15666.667, 3200., 3000.

13078, 16133.333, 3200., 3000.

13079, 16600., 3200., 3000.

13080, 17066.666, 3200., 3000.

13081, 17533.334, 3200., 3000.

13082, 15666.667, 3000., 3000.

13083, 16133.333, 3000., 3000.

13084, 16600., 3000., 3000.

13085, 17066.666, 3000., 3000.

13086, 17533.334, 3000., 3000.

13087, 15666.667, 2533.33325, 3000.

13088, 16133.333, 2533.33325, 3000.

13089, 16600., 2533.33325, 3000.

13090, 17066.666, 2533.33325, 3000.

13091, 17533.334, 2533.33325, 3000.

13092, 15666.667, 2066.66675, 3000.

13093, 16133.333, 2066.66675, 3000.

13094, 16600., 2066.66675, 3000.

13095, 17066.666, 2066.66675, 3000.

13096, 17533.334, 2066.66675, 3000.

13097, 15666.667, 1600., 3000.

13098, 16133.333, 1600., 3000.

13099, 16600., 1600., 3000.

13100, 17066.666, 1600., 3000.

13101, 17533.334, 1600., 3000.

13102, 15666.667, 1133.33337, 3000.

13103, 16133.333, 1133.33337, 3000.

13104, 16600., 1133.33337, 3000.

13105, 17066.666, 1133.33337, 3000.

13106, 17533.334, 1133.33337, 3000.

13107, 15666.667, 666.666687, 3000.

13108, 16133.333, 666.666687, 3000.

13109, 16600., 666.666687, 3000.

13110, 17066.666, 666.666687, 3000.

13111, 17533.334, 666.666687, 3000.

13112, 15666.667, 200., 3000.

13113, 16133.333, 200., 3000.

13114, 16600., 200., 3000.

13115, 17066.666, 200., 3000.

13116, 17533.334, 200., 3000.

13117, 20533.334, 8533.33301, 2920.

13118, 20533.334, 8066.6665, 2920.

13119, 20533.334, 7600., 2920.

13120, 20533.334, 7133.3335, 2920.

13121, 20533.334, 6666.6665, 2920.

13122, 20066.666, 8533.33301, 2920.

13123, 20066.666, 8066.6665, 2920.

13124, 20066.666, 7600., 2920.

13125, 20066.666, 7133.3335, 2920.

13126, 20066.666, 6666.6665, 2920.

13127, 19600., 8533.33301, 2920.

13128, 19600., 8066.6665, 2920.

13129, 19600., 7600., 2920.

13130, 19600., 7133.3335, 2920.

13131, 19600., 6666.6665, 2920.

13132, 19133.334, 8533.33301, 2920.

13133, 19133.334, 8066.6665, 2920.

13134, 19133.334, 7600., 2920.

13135, 19133.334, 7133.3335, 2920.

13136, 19133.334, 6666.6665, 2920.

13137, 18666.666, 8533.33301, 2920.

13138, 18666.666, 8066.6665, 2920.

13139, 18666.666, 7600., 2920.

13140, 18666.666, 7133.3335, 2920.

13141, 18666.666, 6666.6665, 2920.

13142, 20533.334, 11533.333, 2920.

13143, 20533.334, 11066.667, 2920.

13144, 20533.334, 10600., 2920.

13145, 20533.334, 10133.333, 2920.

13146, 20533.334, 9666.66699, 2920.

13147, 20066.666, 11533.333, 2920.

13148, 20066.666, 11066.667, 2920.

13149, 20066.666, 10600., 2920.

13150, 20066.666, 10133.333, 2920.

13151, 20066.666, 9666.66699, 2920.

13152, 19600., 11533.333, 2920.

13153, 19600., 11066.667, 2920.

13154, 19600., 10600., 2920.

13155, 19600., 10133.333, 2920.

13156, 19600., 9666.66699, 2920.

13157, 19133.334, 11533.333, 2920.

13158, 19133.334, 11066.667, 2920.

13159, 19133.334, 10600., 2920.

13160, 19133.334, 10133.333, 2920.

13161, 19133.334, 9666.66699, 2920.

13162, 18666.666, 11533.333, 2920.

13163, 18666.666, 11066.667, 2920.

13164, 18666.666, 10600., 2920.

13165, 18666.666, 10133.333, 2920.

13166, 18666.666, 9666.66699, 2920.

13167, 20533.334, 14533.333, 2920.

13168, 20533.334, 14066.667, 2920.

13169, 20533.334, 13600., 2920.

13170, 20533.334, 13133.333, 2920.

13171, 20533.334, 12666.667, 2920.

13172, 20066.666, 14533.333, 2920.

13173, 20066.666, 14066.667, 2920.

13174, 20066.666, 13600., 2920.

13175, 20066.666, 13133.333, 2920.

13176, 20066.666, 12666.667, 2920.

13177, 19600., 14533.333, 2920.

13178, 19600., 14066.667, 2920.

13179, 19600., 13600., 2920.

13180, 19600., 13133.333, 2920.

13181, 19600., 12666.667, 2920.

13182, 19133.334, 14533.333, 2920.

13183, 19133.334, 14066.667, 2920.

13184, 19133.334, 13600., 2920.

13185, 19133.334, 13133.333, 2920.

13186, 19133.334, 12666.667, 2920.

13187, 18666.666, 14533.333, 2920.

13188, 18666.666, 14066.667, 2920.

13189, 18666.666, 13600., 2920.

13190, 18666.666, 13133.333, 2920.

13191, 18666.666, 12666.667, 2920.

13192, 20533.334, 5533.3335, 2920.

13193, 20533.334, 5066.6665, 2920.

13194, 20533.334, 4600., 2920.

13195, 20533.334, 4133.3335, 2920.

13196, 20533.334, 3666.66675, 2920.

13197, 20066.666, 5533.3335, 2920.

13198, 20066.666, 5066.6665, 2920.

13199, 20066.666, 4600., 2920.

13200, 20066.666, 4133.3335, 2920.

13201, 20066.666, 3666.66675, 2920.

13202, 19600., 5533.3335, 2920.

13203, 19600., 5066.6665, 2920.

13204, 19600., 4600., 2920.

13205, 19600., 4133.3335, 2920.

13206, 19600., 3666.66675, 2920.

13207, 19133.334, 5533.3335, 2920.

13208, 19133.334, 5066.6665, 2920.

13209, 19133.334, 4600., 2920.

13210, 19133.334, 4133.3335, 2920.

13211, 19133.334, 3666.66675, 2920.

13212, 18666.666, 5533.3335, 2920.

13213, 18666.666, 5066.6665, 2920.

13214, 18666.666, 4600., 2920.

13215, 18666.666, 4133.3335, 2920.

13216, 18666.666, 3666.66675, 2920.

13217, 18666.666, 666.666687, 2920.

13218, 18666.666, 1133.33337, 2920.

13219, 18666.666, 1600., 2920.

13220, 18666.666, 2066.66675, 2920.

13221, 18666.666, 2533.33325, 2920.

13222, 19133.334, 666.666687, 2920.

13223, 19133.334, 1133.33337, 2920.

13224, 19133.334, 1600., 2920.

13225, 19133.334, 2066.66675, 2920.

13226, 19133.334, 2533.33325, 2920.

13227, 19600., 666.666687, 2920.

13228, 19600., 1133.33337, 2920.

13229, 19600., 1600., 2920.

13230, 19600., 2066.66675, 2920.

13231, 19600., 2533.33325, 2920.

13232, 20066.666, 666.666687, 2920.

13233, 20066.666, 1133.33337, 2920.

13234, 20066.666, 1600., 2920.

13235, 20066.666, 2066.66675, 2920.

13236, 20066.666, 2533.33325, 2920.

13237, 20533.334, 666.666687, 2920.

13238, 20533.334, 1133.33337, 2920.

13239, 20533.334, 1600., 2920.

13240, 20533.334, 2066.66675, 2920.

13241, 20533.334, 2533.33325, 2920.

13242, 20533.334, 200., 3000.

13243, 20066.666, 200., 3000.

13244, 19600., 200., 3000.

13245, 19133.334, 200., 3000.

13246, 18666.666, 200., 3000.

13247, 20533.334, 666.666687, 3000.

13248, 20066.666, 666.666687, 3000.

13249, 19600., 666.666687, 3000.

13250, 19133.334, 666.666687, 3000.

13251, 18666.666, 666.666687, 3000.

13252, 20533.334, 1133.33337, 3000.

13253, 20066.666, 1133.33337, 3000.

13254, 19600., 1133.33337, 3000.

13255, 19133.334, 1133.33337, 3000.

13256, 18666.666, 1133.33337, 3000.

13257, 20533.334, 1600., 3000.

13258, 20066.666, 1600., 3000.

13259, 19600., 1600., 3000.

13260, 19133.334, 1600., 3000.

13261, 18666.666, 1600., 3000.

13262, 20533.334, 2066.66675, 3000.

13263, 20066.666, 2066.66675, 3000.

13264, 19600., 2066.66675, 3000.

13265, 19133.334, 2066.66675, 3000.

13266, 18666.666, 2066.66675, 3000.

13267, 20533.334, 2533.33325, 3000.

13268, 20066.666, 2533.33325, 3000.

13269, 19600., 2533.33325, 3000.

13270, 19133.334, 2533.33325, 3000.

13271, 18666.666, 2533.33325, 3000.

13272, 20533.334, 3000., 3000.

13273, 20066.666, 3000., 3000.

13274, 19600., 3000., 3000.

13275, 19133.334, 3000., 3000.

13276, 18666.666, 3000., 3000.

13277, 20533.334, 3200., 3000.

13278, 20066.666, 3200., 3000.

13279, 19600., 3200., 3000.

13280, 19133.334, 3200., 3000.

13281, 18666.666, 3200., 3000.

13282, 20533.334, 3666.66675, 3000.

13283, 20066.666, 3666.66675, 3000.

13284, 19600., 3666.66675, 3000.

13285, 19133.334, 3666.66675, 3000.

13286, 18666.666, 3666.66675, 3000.

13287, 20533.334, 4133.3335, 3000.

13288, 20066.666, 4133.3335, 3000.
[truncated: 851,618 more chars]
