# Supplementary material for: Seismic performance evaluation of ancient Tibetan residential structures and energy dissipation of Mortise-Tenon Joints
Source: PLoS One. 2025 Oct 27;20(10):e0334654. doi: 10.1371/journal.pone.0334654 (PMC12558541; doi:10.1371/journal.pone.0334654)
Supplement: S4 File — (DOC) [file pone.0334654.s005.doc]

*Heading

** Job name: Job-2 Model name: Model-1

** Generated by: Abaqus/CAE 2023.HF1

*Preprint, echo=NO, model=NO, history=NO, contact=NO

**

** PARTS

**

*Part, name=Part-suntou

*End Part

**

*Part, name=Part-zhutou

*End Part

**

**

** ASSEMBLY

**

*Assembly, name=Assembly

**

*Instance, name=Part-suntou-1-lin-2-1, part=Part-suntou

-60., 60., -60.

*Node

1, 140., -120., 2760.

2, 140., -120., 2520.

3, 960., -120., 2520.

4, 960., -120., 2760.

5, 140., 0., 2520.

6, 960., 0., 2520.

7, 960., 0., 2760.

8, 140., 0., 2760.

9, 1160., -120., 2760.

10, 1160., -120., 2520.

11, 2340., -120., 2640.

12, 2340., -120., 2520.

13, 2340., -120., 2760.

14, 1160., 0., 2520.

15, 2340., 0., 2520.

16, 2340., 0., 2760.

17, 1160., 0., 2760.

18, 60., -120., 2660.

19, 60., -120., 2760.

20, 60., -120., 2520.

21, 60., 0., 2760.

22, 60., 0., 2520.

23, 60., 0., 2660.

24, -20., -120., 2520.

25, -20., -120., 2660.

26, -60., -120., 2640.

27, -60., -120., 2660.

28, -60., -120., 2520.

29, -20., 0., 2660.

30, -60., 0., 2660.

31, -60., 0., 2520.

32, -20., 0., 2520.

33, 140., -120., 2740.

34, 140., -120., 2720.

35, 140., -120., 2700.

36, 140., -120., 2680.

37, 140., -120., 2660.

38, 140., -120., 2640.

39, 140., -120., 2620.

40, 140., -120., 2600.

41, 140., -120., 2580.

42, 140., -120., 2560.

43, 140., -120., 2540.

44, 170.370377, -120., 2520.

45, 200.740738, -120., 2520.

46, 231.111115, -120., 2520.

47, 261.481476, -120., 2520.

48, 291.851837, -120., 2520.

49, 322.222229, -120., 2520.

50, 352.59259, -120., 2520.

51, 382.962952, -120., 2520.

52, 413.333344, -120., 2520.

53, 443.703705, -120., 2520.

54, 474.074066, -120., 2520.

55, 504.444458, -120., 2520.

56, 534.814819, -120., 2520.

57, 565.185181, -120., 2520.

58, 595.555542, -120., 2520.

59, 625.925903, -120., 2520.

60, 656.296326, -120., 2520.

61, 686.666687, -120., 2520.

62, 717.037048, -120., 2520.

63, 747.40741, -120., 2520.

64, 777.777771, -120., 2520.

65, 808.148132, -120., 2520.

66, 838.518494, -120., 2520.

67, 868.888916, -120., 2520.

68, 899.259277, -120., 2520.

69, 929.629639, -120., 2520.

70, 960., -120., 2540.

71, 960., -120., 2560.

72, 960., -120., 2580.

73, 960., -120., 2600.

74, 960., -120., 2620.

75, 960., -120., 2640.

76, 960., -120., 2660.

77, 960., -120., 2680.

78, 960., -120., 2700.

79, 960., -120., 2720.

80, 960., -120., 2740.

81, 929.629639, -120., 2760.

82, 899.259277, -120., 2760.

83, 868.888916, -120., 2760.

84, 838.518494, -120., 2760.

85, 808.148132, -120., 2760.

86, 777.777771, -120., 2760.

87, 747.40741, -120., 2760.

88, 717.037048, -120., 2760.

89, 686.666687, -120., 2760.

90, 656.296326, -120., 2760.

91, 625.925903, -120., 2760.

92, 595.555542, -120., 2760.

93, 565.185181, -120., 2760.

94, 534.814819, -120., 2760.

95, 504.444458, -120., 2760.

96, 474.074066, -120., 2760.

97, 443.703705, -120., 2760.

98, 413.333344, -120., 2760.

99, 382.962952, -120., 2760.

100, 352.59259, -120., 2760.

101, 322.222229, -120., 2760.

102, 291.851837, -120., 2760.

103, 261.481476, -120., 2760.

104, 231.111115, -120., 2760.

105, 200.740738, -120., 2760.

106, 170.370377, -120., 2760.

107, 140., 0., 2540.

108, 140., 0., 2560.

109, 140., 0., 2580.

110, 140., 0., 2600.

111, 140., 0., 2620.

112, 140., 0., 2640.

113, 140., 0., 2660.

114, 140., 0., 2680.

115, 140., 0., 2700.

116, 140., 0., 2720.

117, 140., 0., 2740.

118, 929.629639, 0., 2520.

119, 899.259277, 0., 2520.

120, 868.888916, 0., 2520.

121, 838.518494, 0., 2520.

122, 808.148132, 0., 2520.

123, 777.777771, 0., 2520.

124, 747.40741, 0., 2520.

125, 717.037048, 0., 2520.

126, 686.666687, 0., 2520.

127, 656.296326, 0., 2520.

128, 625.925903, 0., 2520.

129, 595.555542, 0., 2520.

130, 565.185181, 0., 2520.

131, 534.814819, 0., 2520.

132, 504.444458, 0., 2520.

133, 474.074066, 0., 2520.

134, 443.703705, 0., 2520.

135, 413.333344, 0., 2520.

136, 382.962952, 0., 2520.

137, 352.59259, 0., 2520.

138, 322.222229, 0., 2520.

139, 291.851837, 0., 2520.

140, 261.481476, 0., 2520.

141, 231.111115, 0., 2520.

142, 200.740738, 0., 2520.

143, 170.370377, 0., 2520.

144, 960., 0., 2740.

145, 960., 0., 2720.

146, 960., 0., 2700.

147, 960., 0., 2680.

148, 960., 0., 2660.

149, 960., 0., 2640.

150, 960., 0., 2620.

151, 960., 0., 2600.

152, 960., 0., 2580.

153, 960., 0., 2560.

154, 960., 0., 2540.

155, 170.370377, 0., 2760.

156, 200.740738, 0., 2760.

157, 231.111115, 0., 2760.

158, 261.481476, 0., 2760.

159, 291.851837, 0., 2760.

160, 322.222229, 0., 2760.

161, 352.59259, 0., 2760.

162, 382.962952, 0., 2760.

163, 413.333344, 0., 2760.

164, 443.703705, 0., 2760.

165, 474.074066, 0., 2760.

166, 504.444458, 0., 2760.

167, 534.814819, 0., 2760.

168, 565.185181, 0., 2760.

169, 595.555542, 0., 2760.

170, 625.925903, 0., 2760.

171, 656.296326, 0., 2760.

172, 686.666687, 0., 2760.

173, 717.037048, 0., 2760.

174, 747.40741, 0., 2760.

175, 777.777771, 0., 2760.

176, 808.148132, 0., 2760.

177, 838.518494, 0., 2760.

178, 868.888916, 0., 2760.

179, 899.259277, 0., 2760.

180, 929.629639, 0., 2760.

181, 960., -100., 2760.

182, 960., -80., 2760.

183, 960., -60., 2760.

184, 960., -40., 2760.

185, 960., -20., 2760.

186, 150.938187, -18.4037704, 2760.

187, 157.708282, -38.7140732, 2760.

188, 160., -60., 2760.

189, 157.708282, -81.2859268, 2760.

190, 150.938187, -101.59623, 2760.

191, 960., -20., 2520.

192, 960., -40., 2520.

193, 960., -60., 2520.

194, 960., -80., 2520.

195, 960., -100., 2520.

196, 150.938187, -101.59623, 2520.

197, 157.708282, -81.2859268, 2520.

198, 160., -60., 2520.

199, 157.708282, -38.7140732, 2520.

200, 150.938187, -18.4037704, 2520.

201, 1160., -120., 2740.

202, 1160., -120., 2720.

203, 1160., -120., 2700.

204, 1160., -120., 2680.

205, 1160., -120., 2660.

206, 1160., -120., 2640.

207, 1160., -120., 2620.

208, 1160., -120., 2600.

209, 1160., -120., 2580.

210, 1160., -120., 2560.

211, 1160., -120., 2540.

212, 1190.25647, -120., 2520.

213, 1220.51282, -120., 2520.

214, 1250.76929, -120., 2520.

215, 1281.02563, -120., 2520.

216, 1311.2821, -120., 2520.

217, 1341.53845, -120., 2520.

218, 1371.79492, -120., 2520.

219, 1402.05127, -120., 2520.

220, 1432.30774, -120., 2520.

221, 1462.56409, -120., 2520.

222, 1492.82056, -120., 2520.

223, 1523.0769, -120., 2520.

224, 1553.33337, -120., 2520.

225, 1583.58972, -120., 2520.

226, 1613.84619, -120., 2520.

227, 1644.10254, -120., 2520.

228, 1674.35901, -120., 2520.

229, 1704.61536, -120., 2520.

230, 1734.87183, -120., 2520.

231, 1765.12817, -120., 2520.

232, 1795.38464, -120., 2520.

233, 1825.64099, -120., 2520.

234, 1855.89746, -120., 2520.

235, 1886.15381, -120., 2520.

236, 1916.41028, -120., 2520.

237, 1946.66663, -120., 2520.

238, 1976.9231, -120., 2520.

239, 2007.17944, -120., 2520.

240, 2037.43591, -120., 2520.

241, 2067.69238, -120., 2520.

242, 2097.94873, -120., 2520.

243, 2128.20508, -120., 2520.

244, 2158.46143, -120., 2520.

245, 2188.71802, -120., 2520.

246, 2218.97437, -120., 2520.

247, 2249.23071, -120., 2520.

248, 2279.48706, -120., 2520.

249, 2309.74365, -120., 2520.

250, 2340., -120., 2660.

251, 2340., -120., 2680.

252, 2340., -120., 2700.

253, 2340., -120., 2720.

254, 2340., -120., 2740.

255, 2340., -120., 2540.

256, 2340., -120., 2560.

257, 2340., -120., 2580.

258, 2340., -120., 2600.

259, 2340., -120., 2620.

260, 2309.74365, -120., 2760.

261, 2279.48706, -120., 2760.

262, 2249.23071, -120., 2760.

263, 2218.97437, -120., 2760.

264, 2188.71802, -120., 2760.

265, 2158.46143, -120., 2760.

266, 2128.20508, -120., 2760.

267, 2097.94873, -120., 2760.

268, 2067.69238, -120., 2760.

269, 2037.43591, -120., 2760.

270, 2007.17944, -120., 2760.

271, 1976.9231, -120., 2760.

272, 1946.66663, -120., 2760.

273, 1916.41028, -120., 2760.

274, 1886.15381, -120., 2760.

275, 1855.89746, -120., 2760.

276, 1825.64099, -120., 2760.

277, 1795.38464, -120., 2760.

278, 1765.12817, -120., 2760.

279, 1734.87183, -120., 2760.

280, 1704.61536, -120., 2760.

281, 1674.35901, -120., 2760.

282, 1644.10254, -120., 2760.

283, 1613.84619, -120., 2760.

284, 1583.58972, -120., 2760.

285, 1553.33337, -120., 2760.

286, 1523.0769, -120., 2760.

287, 1492.82056, -120., 2760.

288, 1462.56409, -120., 2760.

289, 1432.30774, -120., 2760.

290, 1402.05127, -120., 2760.

291, 1371.79492, -120., 2760.

292, 1341.53845, -120., 2760.

293, 1311.2821, -120., 2760.

294, 1281.02563, -120., 2760.

295, 1250.76929, -120., 2760.

296, 1220.51282, -120., 2760.

297, 1190.25647, -120., 2760.

298, 1160., 0., 2540.

299, 1160., 0., 2560.

300, 1160., 0., 2580.

301, 1160., 0., 2600.

302, 1160., 0., 2620.

303, 1160., 0., 2640.

304, 1160., 0., 2660.

305, 1160., 0., 2680.

306, 1160., 0., 2700.

307, 1160., 0., 2720.

308, 1160., 0., 2740.

309, 2309.74365, 0., 2520.

310, 2279.48706, 0., 2520.

311, 2249.23071, 0., 2520.

312, 2218.97437, 0., 2520.

313, 2188.71802, 0., 2520.

314, 2158.46143, 0., 2520.

315, 2128.20508, 0., 2520.

316, 2097.94873, 0., 2520.

317, 2067.69238, 0., 2520.

318, 2037.43591, 0., 2520.

319, 2007.17944, 0., 2520.

320, 1976.9231, 0., 2520.

321, 1946.66663, 0., 2520.

322, 1916.41028, 0., 2520.

323, 1886.15381, 0., 2520.

324, 1855.89746, 0., 2520.

325, 1825.64099, 0., 2520.

326, 1795.38464, 0., 2520.

327, 1765.12817, 0., 2520.

328, 1734.87183, 0., 2520.

329, 1704.61536, 0., 2520.

330, 1674.35901, 0., 2520.

331, 1644.10254, 0., 2520.

332, 1613.84619, 0., 2520.

333, 1583.58972, 0., 2520.

334, 1553.33337, 0., 2520.

335, 1523.0769, 0., 2520.

336, 1492.82056, 0., 2520.

337, 1462.56409, 0., 2520.

338, 1432.30774, 0., 2520.

339, 1402.05127, 0., 2520.

340, 1371.79492, 0., 2520.

341, 1341.53845, 0., 2520.

342, 1311.2821, 0., 2520.

343, 1281.02563, 0., 2520.

344, 1250.76929, 0., 2520.

345, 1220.51282, 0., 2520.

346, 1190.25647, 0., 2520.

347, 2340., 0., 2740.

348, 2340., 0., 2720.

349, 2340., 0., 2700.

350, 2340., 0., 2680.

351, 2340., 0., 2660.

352, 2340., 0., 2640.

353, 2340., 0., 2620.

354, 2340., 0., 2600.

355, 2340., 0., 2580.

356, 2340., 0., 2560.

357, 2340., 0., 2540.

358, 1190.25647, 0., 2760.

359, 1220.51282, 0., 2760.

360, 1250.76929, 0., 2760.

361, 1281.02563, 0., 2760.

362, 1311.2821, 0., 2760.

363, 1341.53845, 0., 2760.

364, 1371.79492, 0., 2760.

365, 1402.05127, 0., 2760.

366, 1432.30774, 0., 2760.

367, 1462.56409, 0., 2760.

368, 1492.82056, 0., 2760.

369, 1523.0769, 0., 2760.

370, 1553.33337, 0., 2760.

371, 1583.58972, 0., 2760.

372, 1613.84619, 0., 2760.

373, 1644.10254, 0., 2760.

374, 1674.35901, 0., 2760.

375, 1704.61536, 0., 2760.

376, 1734.87183, 0., 2760.

377, 1765.12817, 0., 2760.

378, 1795.38464, 0., 2760.

379, 1825.64099, 0., 2760.

380, 1855.89746, 0., 2760.

381, 1886.15381, 0., 2760.

382, 1916.41028, 0., 2760.

383, 1946.66663, 0., 2760.

384, 1976.9231, 0., 2760.

385, 2007.17944, 0., 2760.

386, 2037.43591, 0., 2760.

387, 2067.69238, 0., 2760.

388, 2097.94873, 0., 2760.

389, 2128.20508, 0., 2760.

390, 2158.46143, 0., 2760.

391, 2188.71802, 0., 2760.

392, 2218.97437, 0., 2760.

393, 2249.23071, 0., 2760.

394, 2279.48706, 0., 2760.

395, 2309.74365, 0., 2760.

396, 2340., -100., 2760.

397, 2340., -80., 2760.

398, 2340., -60., 2760.

399, 2340., -40., 2760.

400, 2340., -20., 2760.

401, 1160., -20., 2760.

402, 1160., -40., 2760.

403, 1160., -60., 2760.

404, 1160., -80., 2760.

405, 1160., -100., 2760.

406, 2340., -20., 2520.

407, 2340., -40., 2520.

408, 2340., -60., 2520.

409, 2340., -80., 2520.

410, 2340., -100., 2520.

411, 1160., -100., 2520.

412, 1160., -80., 2520.

413, 1160., -60., 2520.

414, 1160., -40., 2520.

415, 1160., -20., 2520.

416, 120., -120., 2760.

417, 100., -120., 2760.

418, 80., -120., 2760.

419, 60., -120., 2640.

420, 60., -120., 2620.

421, 60., -120., 2600.

422, 60., -120., 2580.

423, 60., -120., 2560.

424, 60., -120., 2540.

425, 60., -120., 2740.

426, 60., -120., 2720.

427, 60., -120., 2700.

428, 60., -120., 2680.

429, 80., -120., 2520.

430, 100., -120., 2520.

431, 120., -120., 2520.

432, 80., 0., 2760.

433, 100., 0., 2760.

434, 120., 0., 2760.

435, 60., 0., 2540.

436, 60., 0., 2560.

437, 60., 0., 2580.

438, 60., 0., 2600.

439, 60., 0., 2620.

440, 60., 0., 2640.

441, 60., 0., 2680.

442, 60., 0., 2700.

443, 60., 0., 2720.

444, 60., 0., 2740.

445, 120., 0., 2520.

446, 100., 0., 2520.

447, 80., 0., 2520.

448, 60., -100., 2520.

449, 60., -80., 2520.

450, 60., -60., 2520.

451, 60., -40., 2520.

452, 60., -20., 2520.

453, 60., -20., 2760.

454, 60., -40., 2760.

455, 60., -60., 2760.

456, 60., -80., 2760.

457, 60., -100., 2760.

458, 60., -20., 2660.

459, 60., -40., 2660.

460, 60., -60., 2660.

461, 60., -80., 2660.

462, 60., -100., 2660.

463, 1131.42859, -120., 2760.

464, 1102.85718, -120., 2760.

465, 1074.28577, -120., 2760.

466, 1045.71423, -120., 2760.

467, 1017.14288, -120., 2760.

468, 988.571411, -120., 2760.

469, 988.571411, -120., 2520.

470, 1017.14288, -120., 2520.

471, 1045.71423, -120., 2520.

472, 1074.28577, -120., 2520.

473, 1102.85718, -120., 2520.

474, 1131.42859, -120., 2520.

475, 988.571411, 0., 2760.

476, 1017.14288, 0., 2760.

477, 1045.71423, 0., 2760.

478, 1074.28577, 0., 2760.

479, 1102.85718, 0., 2760.

480, 1131.42859, 0., 2760.

481, 1131.42859, 0., 2520.

482, 1102.85718, 0., 2520.

483, 1074.28577, 0., 2520.

484, 1045.71423, 0., 2520.

485, 1017.14288, 0., 2520.

486, 988.571411, 0., 2520.

487, -20., -120., 2540.

488, -20., -120., 2560.

489, -20., -120., 2580.

490, -20., -120., 2600.

491, -20., -120., 2620.

492, -20., -120., 2640.

493, -60., -120., 2620.

494, -60., -120., 2600.

495, -60., -120., 2580.

496, -60., -120., 2560.

497, -60., -120., 2540.

498, -20., 0., 2640.

499, -20., 0., 2620.

500, -20., 0., 2600.

501, -20., 0., 2580.

502, -20., 0., 2560.

503, -20., 0., 2540.

504, -60., 0., 2540.

505, -60., 0., 2560.

506, -60., 0., 2580.

507, -60., 0., 2600.

508, -60., 0., 2620.

509, -60., 0., 2640.

510, -60., -100., 2520.

511, -60., -80., 2520.

512, -60., -60., 2520.

513, -60., -40., 2520.

514, -60., -20., 2520.

515, -30.9381866, -18.4037704, 2520.

516, -37.7082863, -38.7140732, 2520.

517, -40., -60., 2520.

518, -37.7082863, -81.2859268, 2520.

519, -30.9381866, -101.59623, 2520.

520, -60., -20., 2660.

521, -60., -40., 2660.

522, -60., -60., 2660.

523, -60., -80., 2660.

524, -60., -100., 2660.

525, -30.9381866, -101.59623, 2660.

526, -37.7082863, -81.2859268, 2660.

527, -40., -60., 2660.

528, -37.7082863, -38.7140732, 2660.

529, -30.9381866, -18.4037704, 2660.

530, 40., 0., 2520.

531, 20., 0., 2520.

532, 0., 0., 2520.

533, 0., 0., 2660.

534, 20., 0., 2660.

535, 40., 0., 2660.

536, 0., -120., 2520.

537, 20., -120., 2520.

538, 40., -120., 2520.

539, 40., -120., 2660.

540, 20., -120., 2660.

541, 0., -120., 2660.

542, 170.370377, -120., 2740.

543, 170.370377, -120., 2720.

544, 170.370377, -120., 2700.

545, 170.370377, -120., 2680.

546, 170.370377, -120., 2660.

547, 170.370377, -120., 2640.

548, 170.370377, -120., 2620.

549, 170.370377, -120., 2600.

550, 170.370377, -120., 2580.

551, 170.370377, -120., 2560.

552, 170.370377, -120., 2540.

553, 200.740738, -120., 2740.

554, 200.740738, -120., 2720.

555, 200.740738, -120., 2700.

556, 200.740738, -120., 2680.

557, 200.740738, -120., 2660.

558, 200.740738, -120., 2640.

559, 200.740738, -120., 2620.

560, 200.740738, -120., 2600.

561, 200.740738, -120., 2580.

562, 200.740738, -120., 2560.

563, 200.740738, -120., 2540.

564, 231.111115, -120., 2740.

565, 231.111115, -120., 2720.

566, 231.111115, -120., 2700.

567, 231.111115, -120., 2680.

568, 231.111115, -120., 2660.

569, 231.111115, -120., 2640.

570, 231.111115, -120., 2620.

571, 231.111115, -120., 2600.

572, 231.111115, -120., 2580.

573, 231.111115, -120., 2560.

574, 231.111115, -120., 2540.

575, 261.481476, -120., 2740.

576, 261.481476, -120., 2720.

577, 261.481476, -120., 2700.

578, 261.481476, -120., 2680.

579, 261.481476, -120., 2660.

580, 261.481476, -120., 2640.

581, 261.481476, -120., 2620.

582, 261.481476, -120., 2600.

583, 261.481476, -120., 2580.

584, 261.481476, -120., 2560.

585, 261.481476, -120., 2540.

586, 291.851837, -120., 2740.

587, 291.851837, -120., 2720.

588, 291.851837, -120., 2700.

589, 291.851837, -120., 2680.

590, 291.851837, -120., 2660.

591, 291.851837, -120., 2640.

592, 291.851837, -120., 2620.

593, 291.851837, -120., 2600.

594, 291.851837, -120., 2580.

595, 291.851837, -120., 2560.

596, 291.851837, -120., 2540.

597, 322.222229, -120., 2740.

598, 322.222229, -120., 2720.

599, 322.222229, -120., 2700.

600, 322.222229, -120., 2680.

601, 322.222229, -120., 2660.

602, 322.222229, -120., 2640.

603, 322.222229, -120., 2620.

604, 322.222229, -120., 2600.

605, 322.222229, -120., 2580.

606, 322.222229, -120., 2560.

607, 322.222229, -120., 2540.

608, 352.59259, -120., 2740.

609, 352.59259, -120., 2720.

610, 352.59259, -120., 2700.

611, 352.59259, -120., 2680.

612, 352.59259, -120., 2660.

613, 352.59259, -120., 2640.

614, 352.59259, -120., 2620.

615, 352.59259, -120., 2600.

616, 352.59259, -120., 2580.

617, 352.59259, -120., 2560.

618, 352.59259, -120., 2540.

619, 382.962952, -120., 2740.

620, 382.962952, -120., 2720.

621, 382.962952, -120., 2700.

622, 382.962952, -120., 2680.

623, 382.962952, -120., 2660.

624, 382.962952, -120., 2640.

625, 382.962952, -120., 2620.

626, 382.962952, -120., 2600.

627, 382.962952, -120., 2580.

628, 382.962952, -120., 2560.

629, 382.962952, -120., 2540.

630, 413.333344, -120., 2740.

631, 413.333344, -120., 2720.

632, 413.333344, -120., 2700.

633, 413.333344, -120., 2680.

634, 413.333344, -120., 2660.

635, 413.333344, -120., 2640.

636, 413.333344, -120., 2620.

637, 413.333344, -120., 2600.

638, 413.333344, -120., 2580.

639, 413.333344, -120., 2560.

640, 413.333344, -120., 2540.

641, 443.703705, -120., 2740.

642, 443.703705, -120., 2720.

643, 443.703705, -120., 2700.

644, 443.703705, -120., 2680.

645, 443.703705, -120., 2660.

646, 443.703705, -120., 2640.

647, 443.703705, -120., 2620.

648, 443.703705, -120., 2600.

649, 443.703705, -120., 2580.

650, 443.703705, -120., 2560.

651, 443.703705, -120., 2540.

652, 474.074066, -120., 2740.

653, 474.074066, -120., 2720.

654, 474.074066, -120., 2700.

655, 474.074066, -120., 2680.

656, 474.074066, -120., 2660.

657, 474.074066, -120., 2640.

658, 474.074066, -120., 2620.

659, 474.074066, -120., 2600.

660, 474.074066, -120., 2580.

661, 474.074066, -120., 2560.

662, 474.074066, -120., 2540.

663, 504.444458, -120., 2740.

664, 504.444458, -120., 2720.

665, 504.444458, -120., 2700.

666, 504.444458, -120., 2680.

667, 504.444458, -120., 2660.

668, 504.444458, -120., 2640.

669, 504.444458, -120., 2620.

670, 504.444458, -120., 2600.

671, 504.444458, -120., 2580.

672, 504.444458, -120., 2560.

673, 504.444458, -120., 2540.

674, 534.814819, -120., 2740.

675, 534.814819, -120., 2720.

676, 534.814819, -120., 2700.

677, 534.814819, -120., 2680.

678, 534.814819, -120., 2660.

679, 534.814819, -120., 2640.

680, 534.814819, -120., 2620.

681, 534.814819, -120., 2600.

682, 534.814819, -120., 2580.

683, 534.814819, -120., 2560.

684, 534.814819, -120., 2540.

685, 565.185181, -120., 2740.

686, 565.185181, -120., 2720.

687, 565.185181, -120., 2700.

688, 565.185181, -120., 2680.

689, 565.185181, -120., 2660.

690, 565.185181, -120., 2640.

691, 565.185181, -120., 2620.

692, 565.185181, -120., 2600.

693, 565.185181, -120., 2580.

694, 565.185181, -120., 2560.

695, 565.185181, -120., 2540.

696, 595.555542, -120., 2740.

697, 595.555542, -120., 2720.

698, 595.555542, -120., 2700.

699, 595.555542, -120., 2680.

700, 595.555542, -120., 2660.

701, 595.555542, -120., 2640.

702, 595.555542, -120., 2620.

703, 595.555542, -120., 2600.

704, 595.555542, -120., 2580.

705, 595.555542, -120., 2560.

706, 595.555542, -120., 2540.

707, 625.925903, -120., 2740.

708, 625.925903, -120., 2720.

709, 625.925903, -120., 2700.

710, 625.925903, -120., 2680.

711, 625.925903, -120., 2660.

712, 625.925903, -120., 2640.

713, 625.925903, -120., 2620.

714, 625.925903, -120., 2600.

715, 625.925903, -120., 2580.

716, 625.925903, -120., 2560.

717, 625.925903, -120., 2540.

718, 656.296326, -120., 2740.

719, 656.296326, -120., 2720.

720, 656.296326, -120., 2700.

721, 656.296326, -120., 2680.

722, 656.296326, -120., 2660.

723, 656.296326, -120., 2640.

724, 656.296326, -120., 2620.

725, 656.296326, -120., 2600.

726, 656.296326, -120., 2580.

727, 656.296326, -120., 2560.

728, 656.296326, -120., 2540.

729, 686.666687, -120., 2740.

730, 686.666687, -120., 2720.

731, 686.666687, -120., 2700.

732, 686.666687, -120., 2680.

733, 686.666687, -120., 2660.

734, 686.666687, -120., 2640.

735, 686.666687, -120., 2620.

736, 686.666687, -120., 2600.

737, 686.666687, -120., 2580.

738, 686.666687, -120., 2560.

739, 686.666687, -120., 2540.

740, 717.037048, -120., 2740.

741, 717.037048, -120., 2720.

742, 717.037048, -120., 2700.

743, 717.037048, -120., 2680.

744, 717.037048, -120., 2660.

745, 717.037048, -120., 2640.

746, 717.037048, -120., 2620.

747, 717.037048, -120., 2600.

748, 717.037048, -120., 2580.

749, 717.037048, -120., 2560.

750, 717.037048, -120., 2540.

751, 747.40741, -120., 2740.

752, 747.40741, -120., 2720.

753, 747.40741, -120., 2700.

754, 747.40741, -120., 2680.

755, 747.40741, -120., 2660.

756, 747.40741, -120., 2640.

757, 747.40741, -120., 2620.

758, 747.40741, -120., 2600.

759, 747.40741, -120., 2580.

760, 747.40741, -120., 2560.

761, 747.40741, -120., 2540.

762, 777.777771, -120., 2740.

763, 777.777771, -120., 2720.

764, 777.777771, -120., 2700.

765, 777.777771, -120., 2680.

766, 777.777771, -120., 2660.

767, 777.777771, -120., 2640.

768, 777.777771, -120., 2620.

769, 777.777771, -120., 2600.

770, 777.777771, -120., 2580.

771, 777.777771, -120., 2560.

772, 777.777771, -120., 2540.

773, 808.148132, -120., 2740.

774, 808.148132, -120., 2720.

775, 808.148132, -120., 2700.

776, 808.148132, -120., 2680.

777, 808.148132, -120., 2660.

778, 808.148132, -120., 2640.

779, 808.148132, -120., 2620.

780, 808.148132, -120., 2600.

781, 808.148132, -120., 2580.

782, 808.148132, -120., 2560.

783, 808.148132, -120., 2540.

784, 838.518494, -120., 2740.

785, 838.518494, -120., 2720.

786, 838.518494, -120., 2700.

787, 838.518494, -120., 2680.

788, 838.518494, -120., 2660.

789, 838.518494, -120., 2640.

790, 838.518494, -120., 2620.

791, 838.518494, -120., 2600.

792, 838.518494, -120., 2580.

793, 838.518494, -120., 2560.

794, 838.518494, -120., 2540.

795, 868.888916, -120., 2740.

796, 868.888916, -120., 2720.

797, 868.888916, -120., 2700.

798, 868.888916, -120., 2680.

799, 868.888916, -120., 2660.

800, 868.888916, -120., 2640.

801, 868.888916, -120., 2620.

802, 868.888916, -120., 2600.

803, 868.888916, -120., 2580.

804, 868.888916, -120., 2560.

805, 868.888916, -120., 2540.

806, 899.259277, -120., 2740.

807, 899.259277, -120., 2720.

808, 899.259277, -120., 2700.

809, 899.259277, -120., 2680.

810, 899.259277, -120., 2660.

811, 899.259277, -120., 2640.

812, 899.259277, -120., 2620.

813, 899.259277, -120., 2600.

814, 899.259277, -120., 2580.

815, 899.259277, -120., 2560.

816, 899.259277, -120., 2540.

817, 929.629639, -120., 2740.

818, 929.629639, -120., 2720.

819, 929.629639, -120., 2700.

820, 929.629639, -120., 2680.

821, 929.629639, -120., 2660.

822, 929.629639, -120., 2640.

823, 929.629639, -120., 2620.

824, 929.629639, -120., 2600.

825, 929.629639, -120., 2580.

826, 929.629639, -120., 2560.

827, 929.629639, -120., 2540.

828, 170.370377, 0., 2740.

829, 170.370377, 0., 2720.

830, 170.370377, 0., 2700.

831, 170.370377, 0., 2680.

832, 170.370377, 0., 2660.

833, 170.370377, 0., 2640.

834, 170.370377, 0., 2620.

835, 170.370377, 0., 2600.

836, 170.370377, 0., 2580.

837, 170.370377, 0., 2560.

838, 170.370377, 0., 2540.

839, 200.740738, 0., 2740.

840, 200.740738, 0., 2720.

841, 200.740738, 0., 2700.

842, 200.740738, 0., 2680.

843, 200.740738, 0., 2660.

844, 200.740738, 0., 2640.

845, 200.740738, 0., 2620.

846, 200.740738, 0., 2600.

847, 200.740738, 0., 2580.

848, 200.740738, 0., 2560.

849, 200.740738, 0., 2540.

850, 231.111115, 0., 2740.

851, 231.111115, 0., 2720.

852, 231.111115, 0., 2700.

853, 231.111115, 0., 2680.

854, 231.111115, 0., 2660.

855, 231.111115, 0., 2640.

856, 231.111115, 0., 2620.

857, 231.111115, 0., 2600.

858, 231.111115, 0., 2580.

859, 231.111115, 0., 2560.

860, 231.111115, 0., 2540.

861, 261.481476, 0., 2740.

862, 261.481476, 0., 2720.

863, 261.481476, 0., 2700.

864, 261.481476, 0., 2680.

865, 261.481476, 0., 2660.

866, 261.481476, 0., 2640.

867, 261.481476, 0., 2620.

868, 261.481476, 0., 2600.

869, 261.481476, 0., 2580.

870, 261.481476, 0., 2560.

871, 261.481476, 0., 2540.

872, 291.851837, 0., 2740.

873, 291.851837, 0., 2720.

874, 291.851837, 0., 2700.

875, 291.851837, 0., 2680.

876, 291.851837, 0., 2660.

877, 291.851837, 0., 2640.

878, 291.851837, 0., 2620.

879, 291.851837, 0., 2600.

880, 291.851837, 0., 2580.

881, 291.851837, 0., 2560.

882, 291.851837, 0., 2540.

883, 322.222229, 0., 2740.

884, 322.222229, 0., 2720.

885, 322.222229, 0., 2700.

886, 322.222229, 0., 2680.

887, 322.222229, 0., 2660.

888, 322.222229, 0., 2640.

889, 322.222229, 0., 2620.

890, 322.222229, 0., 2600.

891, 322.222229, 0., 2580.

892, 322.222229, 0., 2560.

893, 322.222229, 0., 2540.

894, 352.59259, 0., 2740.

895, 352.59259, 0., 2720.

896, 352.59259, 0., 2700.

897, 352.59259, 0., 2680.

898, 352.59259, 0., 2660.

899, 352.59259, 0., 2640.

900, 352.59259, 0., 2620.

901, 352.59259, 0., 2600.

902, 352.59259, 0., 2580.

903, 352.59259, 0., 2560.

904, 352.59259, 0., 2540.

905, 382.962952, 0., 2740.

906, 382.962952, 0., 2720.

907, 382.962952, 0., 2700.

908, 382.962952, 0., 2680.

909, 382.962952, 0., 2660.

910, 382.962952, 0., 2640.

911, 382.962952, 0., 2620.

912, 382.962952, 0., 2600.

913, 382.962952, 0., 2580.

914, 382.962952, 0., 2560.

915, 382.962952, 0., 2540.

916, 413.333344, 0., 2740.

917, 413.333344, 0., 2720.

918, 413.333344, 0., 2700.

919, 413.333344, 0., 2680.

920, 413.333344, 0., 2660.

921, 413.333344, 0., 2640.

922, 413.333344, 0., 2620.

923, 413.333344, 0., 2600.

924, 413.333344, 0., 2580.

925, 413.333344, 0., 2560.

926, 413.333344, 0., 2540.

927, 443.703705, 0., 2740.

928, 443.703705, 0., 2720.

929, 443.703705, 0., 2700.

930, 443.703705, 0., 2680.

931, 443.703705, 0., 2660.

932, 443.703705, 0., 2640.

933, 443.703705, 0., 2620.

934, 443.703705, 0., 2600.

935, 443.703705, 0., 2580.

936, 443.703705, 0., 2560.

937, 443.703705, 0., 2540.

938, 474.074066, 0., 2740.

939, 474.074066, 0., 2720.

940, 474.074066, 0., 2700.

941, 474.074066, 0., 2680.

942, 474.074066, 0., 2660.

943, 474.074066, 0., 2640.

944, 474.074066, 0., 2620.

945, 474.074066, 0., 2600.

946, 474.074066, 0., 2580.

947, 474.074066, 0., 2560.

948, 474.074066, 0., 2540.

949, 504.444458, 0., 2740.

950, 504.444458, 0., 2720.

951, 504.444458, 0., 2700.

952, 504.444458, 0., 2680.

953, 504.444458, 0., 2660.

954, 504.444458, 0., 2640.

955, 504.444458, 0., 2620.

956, 504.444458, 0., 2600.

957, 504.444458, 0., 2580.

958, 504.444458, 0., 2560.

959, 504.444458, 0., 2540.

960, 534.814819, 0., 2740.

961, 534.814819, 0., 2720.

962, 534.814819, 0., 2700.

963, 534.814819, 0., 2680.

964, 534.814819, 0., 2660.

965, 534.814819, 0., 2640.

966, 534.814819, 0., 2620.

967, 534.814819, 0., 2600.

968, 534.814819, 0., 2580.

969, 534.814819, 0., 2560.

970, 534.814819, 0., 2540.

971, 565.185181, 0., 2740.

972, 565.185181, 0., 2720.

973, 565.185181, 0., 2700.

974, 565.185181, 0., 2680.

975, 565.185181, 0., 2660.

976, 565.185181, 0., 2640.

977, 565.185181, 0., 2620.

978, 565.185181, 0., 2600.

979, 565.185181, 0., 2580.

980, 565.185181, 0., 2560.

981, 565.185181, 0., 2540.

982, 595.555542, 0., 2740.

983, 595.555542, 0., 2720.

984, 595.555542, 0., 2700.

985, 595.555542, 0., 2680.

986, 595.555542, 0., 2660.

987, 595.555542, 0., 2640.

988, 595.555542, 0., 2620.

989, 595.555542, 0., 2600.

990, 595.555542, 0., 2580.

991, 595.555542, 0., 2560.

992, 595.555542, 0., 2540.

993, 625.925903, 0., 2740.

994, 625.925903, 0., 2720.

995, 625.925903, 0., 2700.

996, 625.925903, 0., 2680.

997, 625.925903, 0., 2660.

998, 625.925903, 0., 2640.

999, 625.925903, 0., 2620.

1000, 625.925903, 0., 2600.

1001, 625.925903, 0., 2580.

1002, 625.925903, 0., 2560.

1003, 625.925903, 0., 2540.

1004, 656.296326, 0., 2740.

1005, 656.296326, 0., 2720.

1006, 656.296326, 0., 2700.

1007, 656.296326, 0., 2680.

1008, 656.296326, 0., 2660.

1009, 656.296326, 0., 2640.

1010, 656.296326, 0., 2620.

1011, 656.296326, 0., 2600.

1012, 656.296326, 0., 2580.

1013, 656.296326, 0., 2560.

1014, 656.296326, 0., 2540.

1015, 686.666687, 0., 2740.

1016, 686.666687, 0., 2720.

1017, 686.666687, 0., 2700.

1018, 686.666687, 0., 2680.

1019, 686.666687, 0., 2660.

1020, 686.666687, 0., 2640.

1021, 686.666687, 0., 2620.

1022, 686.666687, 0., 2600.

1023, 686.666687, 0., 2580.

1024, 686.666687, 0., 2560.

1025, 686.666687, 0., 2540.

1026, 717.037048, 0., 2740.

1027, 717.037048, 0., 2720.

1028, 717.037048, 0., 2700.

1029, 717.037048, 0., 2680.

1030, 717.037048, 0., 2660.

1031, 717.037048, 0., 2640.

1032, 717.037048, 0., 2620.

1033, 717.037048, 0., 2600.

1034, 717.037048, 0., 2580.

1035, 717.037048, 0., 2560.

1036, 717.037048, 0., 2540.

1037, 747.40741, 0., 2740.

1038, 747.40741, 0., 2720.

1039, 747.40741, 0., 2700.

1040, 747.40741, 0., 2680.

1041, 747.40741, 0., 2660.

1042, 747.40741, 0., 2640.

1043, 747.40741, 0., 2620.

1044, 747.40741, 0., 2600.

1045, 747.40741, 0., 2580.

1046, 747.40741, 0., 2560.

1047, 747.40741, 0., 2540.

1048, 777.777771, 0., 2740.

1049, 777.777771, 0., 2720.

1050, 777.777771, 0., 2700.

1051, 777.777771, 0., 2680.

1052, 777.777771, 0., 2660.

1053, 777.777771, 0., 2640.

1054, 777.777771, 0., 2620.

1055, 777.777771, 0., 2600.

1056, 777.777771, 0., 2580.

1057, 777.777771, 0., 2560.

1058, 777.777771, 0., 2540.

1059, 808.148132, 0., 2740.

1060, 808.148132, 0., 2720.

1061, 808.148132, 0., 2700.

1062, 808.148132, 0., 2680.

1063, 808.148132, 0., 2660.

1064, 808.148132, 0., 2640.

1065, 808.148132, 0., 2620.

1066, 808.148132, 0., 2600.

1067, 808.148132, 0., 2580.

1068, 808.148132, 0., 2560.

1069, 808.148132, 0., 2540.

1070, 838.518494, 0., 2740.

1071, 838.518494, 0., 2720.

1072, 838.518494, 0., 2700.

1073, 838.518494, 0., 2680.

1074, 838.518494, 0., 2660.

1075, 838.518494, 0., 2640.

1076, 838.518494, 0., 2620.

1077, 838.518494, 0., 2600.

1078, 838.518494, 0., 2580.

1079, 838.518494, 0., 2560.

1080, 838.518494, 0., 2540.

1081, 868.888916, 0., 2740.

1082, 868.888916, 0., 2720.

1083, 868.888916, 0., 2700.

1084, 868.888916, 0., 2680.

1085, 868.888916, 0., 2660.

1086, 868.888916, 0., 2640.

1087, 868.888916, 0., 2620.

1088, 868.888916, 0., 2600.

1089, 868.888916, 0., 2580.

1090, 868.888916, 0., 2560.

1091, 868.888916, 0., 2540.

1092, 899.259277, 0., 2740.

1093, 899.259277, 0., 2720.

1094, 899.259277, 0., 2700.

1095, 899.259277, 0., 2680.

1096, 899.259277, 0., 2660.

1097, 899.259277, 0., 2640.

1098, 899.259277, 0., 2620.

1099, 899.259277, 0., 2600.

1100, 899.259277, 0., 2580.

1101, 899.259277, 0., 2560.

1102, 899.259277, 0., 2540.

1103, 929.629639, 0., 2740.

1104, 929.629639, 0., 2720.

1105, 929.629639, 0., 2700.

1106, 929.629639, 0., 2680.

1107, 929.629639, 0., 2660.

1108, 929.629639, 0., 2640.

1109, 929.629639, 0., 2620.

1110, 929.629639, 0., 2600.

1111, 929.629639, 0., 2580.

1112, 929.629639, 0., 2560.

1113, 929.629639, 0., 2540.

1114, 180.903442, -101.537109, 2760.

1115, 210.868698, -101.477989, 2760.

1116, 240.833939, -101.418869, 2760.

1117, 270.799194, -101.359749, 2760.

1118, 300.764435, -101.300629, 2760.

1119, 330.729706, -101.241508, 2760.

1120, 360.694946, -101.182396, 2760.

1121, 390.660217, -101.123276, 2760.

1122, 420.625458, -101.064156, 2760.

1123, 450.590698, -101.005035, 2760.

1124, 480.555969, -100.945915, 2760.

1125, 510.52121, -100.886795, 2760.

1126, 540.48645, -100.827675, 2760.

1127, 570.451721, -100.768555, 2760.

1128, 600.416992, -100.709435, 2760.

1129, 630.382202, -100.650314, 2760.

1130, 660.347473, -100.591194, 2760.

1131, 690.312744, -100.532074, 2760.

1132, 720.277954, -100.472954, 2760.

1133, 750.243225, -100.413834, 2760.

1134, 780.208496, -100.354721, 2760.

1135, 810.173767, -100.295601, 2760.

1136, 840.138977, -100.236481, 2760.

1137, 870.104248, -100.177361, 2760.

1138, 900.069519, -100.11824, 2760.

1139, 930.034729, -100.05912, 2760.

1140, 187.422791, -81.2383041, 2760.

1141, 217.137299, -81.1906738, 2760.

1142, 246.851807, -81.1430435, 2760.

1143, 276.566315, -81.0954208, 2760.

1144, 306.280823, -81.0477905, 2760.

1145, 335.995331, -81.0001678, 2760.

1146, 365.709839, -80.9525375, 2760.

1147, 395.424347, -80.9049149, 2760.

1148, 425.138855, -80.8572845, 2760.

1149, 454.853363, -80.8096542, 2760.

1150, 484.567871, -80.7620316, 2760.

1151, 514.28241, -80.7144012, 2760.

1152, 543.996887, -80.6667786, 2760.

1153, 573.711426, -80.6191483, 2760.

1154, 603.425903, -80.5715256, 2760.

1155, 633.140442, -80.5238953, 2760.

1156, 662.854919, -80.4762726, 2760.

1157, 692.569458, -80.4286423, 2760.

1158, 722.283936, -80.381012, 2760.

1159, 751.998474, -80.3333893, 2760.

1160, 781.712952, -80.285759, 2760.

1161, 811.42749, -80.2381363, 2760.

1162, 841.141968, -80.190506, 2760.

1163, 870.856506, -80.1428833, 2760.

1164, 900.570984, -80.095253, 2760.

1165, 930.285522, -80.0476303, 2760.

1166, 189.629623, -60., 2760.

1167, 219.259262, -60., 2760.

1168, 248.888885, -60., 2760.

1169, 278.518524, -60., 2760.

1170, 308.148163, -60., 2760.

1171, 337.777771, -60., 2760.

1172, 367.40741, -60., 2760.

1173, 397.037048, -60., 2760.

1174, 426.666656, -60., 2760.

1175, 456.296295, -60., 2760.

1176, 485.925934, -60., 2760.

1177, 515.555542, -60., 2760.

1178, 545.185181, -60., 2760.

1179, 574.814819, -60., 2760.

1180, 604.444458, -60., 2760.

1181, 634.074097, -60., 2760.

1182, 663.703674, -60., 2760.

1183, 693.333313, -60., 2760.

1184, 722.962952, -60., 2760.

1185, 752.59259, -60., 2760.

1186, 782.222229, -60., 2760.

1187, 811.851868, -60., 2760.

1188, 841.481506, -60., 2760.

1189, 871.111084, -60., 2760.

1190, 900.740723, -60., 2760.

1191, 930.370361, -60., 2760.

1192, 187.422791, -38.7616997, 2760.

1193, 217.137299, -38.8093262, 2760.

1194, 246.851807, -38.8569527, 2760.

1195, 276.566315, -38.9045792, 2760.

1196, 306.280823, -38.9522057, 2760.

1197, 335.995331, -38.999836, 2760.

1198, 365.709839, -39.0474625, 2760.

1199, 395.424347, -39.095089, 2760.

1200, 425.138855, -39.1427155, 2760.

1201, 454.853363, -39.1903419, 2760.

1202, 484.567871, -39.2379684, 2760.

1203, 514.28241, -39.2855949, 2760.

1204, 543.996887, -39.3332214, 2760.

1205, 573.711426, -39.3808479, 2760.

1206, 603.425903, -39.4284782, 2760.

1207, 633.140442, -39.4761047, 2760.

1208, 662.854919, -39.5237312, 2760.

1209, 692.569458, -39.5713577, 2760.

1210, 722.283936, -39.6189842, 2760.

1211, 751.998474, -39.6666107, 2760.

1212, 781.712952, -39.7142372, 2760.

1213, 811.42749, -39.7618637, 2760.

1214, 841.141968, -39.809494, 2760.

1215, 870.856506, -39.8571205, 2760.

1216, 900.570984, -39.904747, 2760.

1217, 930.285522, -39.9523735, 2760.

1218, 180.903442, -18.4628887, 2760.

1219, 210.868698, -18.5220089, 2760.

1220, 240.833939, -18.5811291, 2760.

1221, 270.799194, -18.6402493, 2760.

1222, 300.764435, -18.6993675, 2760.

1223, 330.729706, -18.7584877, 2760.

1224, 360.694946, -18.8176079, 2760.

1225, 390.660217, -18.8767262, 2760.

1226, 420.625458, -18.9358463, 2760.

1227, 450.590698, -18.9949665, 2760.

1228, 480.555969, -19.0540867, 2760.

1229, 510.52121, -19.113205, 2760.

1230, 540.48645, -19.1723251, 2760.

1231, 570.451721, -19.2314453, 2760.

1232, 600.416992, -19.2905636, 2760.

1233, 630.382202, -19.3496838, 2760.

1234, 660.347473, -19.4088039, 2760.

1235, 690.312744, -19.4679241, 2760.

1236, 720.277954, -19.5270424, 2760.

1237, 750.243225, -19.5861626, 2760.

1238, 780.208496, -19.6452827, 2760.

1239, 810.173767, -19.704401, 2760.

1240, 840.138977, -19.7635212, 2760.

1241, 870.104248, -19.8226414, 2760.

1242, 900.069519, -19.8817616, 2760.

1243, 930.034729, -19.9408798, 2760.

1244, 180.903442, -101.537109, 2520.

1245, 210.868698, -101.477989, 2520.

1246, 240.833939, -101.418869, 2520.

1247, 270.799194, -101.359749, 2520.

1248, 300.764435, -101.300629, 2520.

1249, 330.729706, -101.241508, 2520.

1250, 360.694946, -101.182396, 2520.

1251, 390.660217, -101.123276, 2520.

1252, 420.625458, -101.064156, 2520.

1253, 450.590698, -101.005035, 2520.

1254, 480.555969, -100.945915, 2520.

1255, 510.52121, -100.886795, 2520.

1256, 540.48645, -100.827675, 2520.

1257, 570.451721, -100.768555, 2520.

1258, 600.416992, -100.709435, 2520.

1259, 630.382202, -100.650314, 2520.

1260, 660.347473, -100.591194, 2520.

1261, 690.312744, -100.532074, 2520.

1262, 720.277954, -100.472954, 2520.

1263, 750.243225, -100.413834, 2520.

1264, 780.208496, -100.354721, 2520.

1265, 810.173767, -100.295601, 2520.

1266, 840.138977, -100.236481, 2520.

1267, 870.104248, -100.177361, 2520.

1268, 900.069519, -100.11824, 2520.

1269, 930.034729, -100.05912, 2520.

1270, 187.422791, -81.2383041, 2520.

1271, 217.137299, -81.1906738, 2520.

1272, 246.851807, -81.1430435, 2520.

1273, 276.566315, -81.0954208, 2520.

1274, 306.280823, -81.0477905, 2520.

1275, 335.995331, -81.0001678, 2520.

1276, 365.709839, -80.9525375, 2520.

1277, 395.424347, -80.9049149, 2520.

1278, 425.138855, -80.8572845, 2520.

1279, 454.853363, -80.8096542, 2520.

1280, 484.567871, -80.7620316, 2520.

1281, 514.28241, -80.7144012, 2520.

1282, 543.996887, -80.6667786, 2520.

1283, 573.711426, -80.6191483, 2520.

1284, 603.425903, -80.5715256, 2520.

1285, 633.140442, -80.5238953, 2520.

1286, 662.854919, -80.4762726, 2520.

1287, 692.569458, -80.4286423, 2520.

1288, 722.283936, -80.381012, 2520.

1289, 751.998474, -80.3333893, 2520.

1290, 781.712952, -80.285759, 2520.

1291, 811.42749, -80.2381363, 2520.

1292, 841.141968, -80.190506, 2520.

1293, 870.856506, -80.1428833, 2520.

1294, 900.570984, -80.095253, 2520.

1295, 930.285522, -80.0476303, 2520.

1296, 189.629623, -60., 2520.

1297, 219.259262, -60., 2520.

1298, 248.888885, -60., 2520.

1299, 278.518524, -60., 2520.

1300, 308.148163, -60., 2520.

1301, 337.777771, -60., 2520.

1302, 367.40741, -60., 2520.

1303, 397.037048, -60., 2520.

1304, 426.666656, -60., 2520.

1305, 456.296295, -60., 2520.

1306, 485.925934, -60., 2520.

1307, 515.555542, -60., 2520.

1308, 545.185181, -60., 2520.

1309, 574.814819, -60., 2520.

1310, 604.444458, -60., 2520.

1311, 634.074097, -60., 2520.

1312, 663.703674, -60., 2520.

1313, 693.333313, -60., 2520.

1314, 722.962952, -60., 2520.

1315, 752.59259, -60., 2520.

1316, 782.222229, -60., 2520.

1317, 811.851868, -60., 2520.

1318, 841.481506, -60., 2520.

1319, 871.111084, -60., 2520.

1320, 900.740723, -60., 2520.

1321, 930.370361, -60., 2520.

1322, 187.422791, -38.7616997, 2520.

1323, 217.137299, -38.8093262, 2520.

1324, 246.851807, -38.8569527, 2520.

1325, 276.566315, -38.9045792, 2520.

1326, 306.280823, -38.9522057, 2520.

1327, 335.995331, -38.999836, 2520.

1328, 365.709839, -39.0474625, 2520.

1329, 395.424347, -39.095089, 2520.

1330, 425.138855, -39.1427155, 2520.

1331, 454.853363, -39.1903419, 2520.

1332, 484.567871, -39.2379684, 2520.

1333, 514.28241, -39.2855949, 2520.

1334, 543.996887, -39.3332214, 2520.

1335, 573.711426, -39.3808479, 2520.

1336, 603.425903, -39.4284782, 2520.

1337, 633.140442, -39.4761047, 2520.

1338, 662.854919, -39.5237312, 2520.

1339, 692.569458, -39.5713577, 2520.

1340, 722.283936, -39.6189842, 2520.

1341, 751.998474, -39.6666107, 2520.

1342, 781.712952, -39.7142372, 2520.

1343, 811.42749, -39.7618637, 2520.

1344, 841.141968, -39.809494, 2520.

1345, 870.856506, -39.8571205, 2520.

1346, 900.570984, -39.904747, 2520.

1347, 930.285522, -39.9523735, 2520.

1348, 180.903442, -18.4628887, 2520.

1349, 210.868698, -18.5220089, 2520.

1350, 240.833939, -18.5811291, 2520.

1351, 270.799194, -18.6402493, 2520.

1352, 300.764435, -18.6993675, 2520.

1353, 330.729706, -18.7584877, 2520.

1354, 360.694946, -18.8176079, 2520.

1355, 390.660217, -18.8767262, 2520.

1356, 420.625458, -18.9358463, 2520.

1357, 450.590698, -18.9949665, 2520.

1358, 480.555969, -19.0540867, 2520.

1359, 510.52121, -19.113205, 2520.

1360, 540.48645, -19.1723251, 2520.

1361, 570.451721, -19.2314453, 2520.

1362, 600.416992, -19.2905636, 2520.

1363, 630.382202, -19.3496838, 2520.

1364, 660.347473, -19.4088039, 2520.

1365, 690.312744, -19.4679241, 2520.

1366, 720.277954, -19.5270424, 2520.

1367, 750.243225, -19.5861626, 2520.

1368, 780.208496, -19.6452827, 2520.

1369, 810.173767, -19.704401, 2520.

1370, 840.138977, -19.7635212, 2520.

1371, 870.104248, -19.8226414, 2520.

1372, 900.069519, -19.8817616, 2520.

1373, 930.034729, -19.9408798, 2520.

1374, 150.938187, -101.59623, 2740.

1375, 150.938187, -101.59623, 2720.

1376, 150.938187, -101.59623, 2700.

1377, 150.938187, -101.59623, 2680.

1378, 150.938187, -101.59623, 2660.

1379, 150.938187, -101.59623, 2640.

1380, 150.938187, -101.59623, 2620.

1381, 150.938187, -101.59623, 2600.

1382, 150.938187, -101.59623, 2580.

1383, 150.938187, -101.59623, 2560.

1384, 150.938187, -101.59623, 2540.

1385, 157.708282, -81.2859268, 2740.

1386, 157.708282, -81.2859268, 2720.

1387, 157.708282, -81.2859268, 2700.

1388, 157.708282, -81.2859268, 2680.

1389, 157.708282, -81.2859268, 2660.

1390, 157.708282, -81.2859268, 2640.

1391, 157.708282, -81.2859268, 2620.

1392, 157.708282, -81.2859268, 2600.

1393, 157.708282, -81.2859268, 2580.

1394, 157.708282, -81.2859268, 2560.

1395, 157.708282, -81.2859268, 2540.

1396, 160., -60., 2740.

1397, 160., -60., 2720.

1398, 160., -60., 2700.

1399, 160., -60., 2680.

1400, 160., -60., 2660.

1401, 160., -60., 2640.

1402, 160., -60., 2620.

1403, 160., -60., 2600.

1404, 160., -60., 2580.

1405, 160., -60., 2560.

1406, 160., -60., 2540.

1407, 157.708282, -38.7140732, 2740.

1408, 157.708282, -38.7140732, 2720.

1409, 157.708282, -38.7140732, 2700.

1410, 157.708282, -38.7140732, 2680.

1411, 157.708282, -38.7140732, 2660.

1412, 157.708282, -38.7140732, 2640.

1413, 157.708282, -38.7140732, 2620.

1414, 157.708282, -38.7140732, 2600.

1415, 157.708282, -38.7140732, 2580.

1416, 157.708282, -38.7140732, 2560.

1417, 157.708282, -38.7140732, 2540.

1418, 150.938187, -18.4037704, 2740.

1419, 150.938187, -18.4037704, 2720.

1420, 150.938187, -18.4037704, 2700.

1421, 150.938187, -18.4037704, 2680.

1422, 150.938187, -18.4037704, 2660.

1423, 150.938187, -18.4037704, 2640.

1424, 150.938187, -18.4037704, 2620.

1425, 150.938187, -18.4037704, 2600.

1426, 150.938187, -18.4037704, 2580.

1427, 150.938187, -18.4037704, 2560.

1428, 150.938187, -18.4037704, 2540.

1429, 960., -100., 2740.

1430, 960., -100., 2720.

1431, 960., -100., 2700.

1432, 960., -100., 2680.

1433, 960., -100., 2660.

1434, 960., -100., 2640.

1435, 960., -100., 2620.

1436, 960., -100., 2600.

1437, 960., -100., 2580.

1438, 960., -100., 2560.

1439, 960., -100., 2540.

1440, 960., -80., 2740.

1441, 960., -80., 2720.

1442, 960., -80., 2700.

1443, 960., -80., 2680.

1444, 960., -80., 2660.

1445, 960., -80., 2640.

1446, 960., -80., 2620.

1447, 960., -80., 2600.

1448, 960., -80., 2580.

1449, 960., -80., 2560.

1450, 960., -80., 2540.

1451, 960., -60., 2740.

1452, 960., -60., 2720.

1453, 960., -60., 2700.

1454, 960., -60., 2680.

1455, 960., -60., 2660.

1456, 960., -60., 2640.

1457, 960., -60., 2620.

1458, 960., -60., 2600.

1459, 960., -60., 2580.

1460, 960., -60., 2560.

1461, 960., -60., 2540.

1462, 960., -40., 2740.

1463, 960., -40., 2720.

1464, 960., -40., 2700.

1465, 960., -40., 2680.

1466, 960., -40., 2660.

1467, 960., -40., 2640.

1468, 960., -40., 2620.

1469, 960., -40., 2600.

1470, 960., -40., 2580.

1471, 960., -40., 2560.

1472, 960., -40., 2540.

1473, 960., -20., 2740.

1474, 960., -20., 2720.

1475, 960., -20., 2700.

1476, 960., -20., 2680.

1477, 960., -20., 2660.

1478, 960., -20., 2640.

1479, 960., -20., 2620.

1480, 960., -20., 2600.

1481, 960., -20., 2580.

1482, 960., -20., 2560.

1483, 960., -20., 2540.

1484, 1190.25647, -120., 2740.

1485, 1190.25647, -120., 2720.

1486, 1190.25647, -120., 2700.

1487, 1190.25647, -120., 2680.

1488, 1190.25647, -120., 2660.

1489, 1190.25647, -120., 2640.

1490, 1190.25647, -120., 2620.

1491, 1190.25647, -120., 2600.

1492, 1190.25647, -120., 2580.

1493, 1190.25647, -120., 2560.

1494, 1190.25647, -120., 2540.

1495, 1220.51282, -120., 2740.

1496, 1220.51282, -120., 2720.

1497, 1220.51282, -120., 2700.

1498, 1220.51282, -120., 2680.

1499, 1220.51282, -120., 2660.

1500, 1220.51282, -120., 2640.

1501, 1220.51282, -120., 2620.

1502, 1220.51282, -120., 2600.

1503, 1220.51282, -120., 2580.

1504, 1220.51282, -120., 2560.

1505, 1220.51282, -120., 2540.

1506, 1250.76929, -120., 2740.

1507, 1250.76929, -120., 2720.

1508, 1250.76929, -120., 2700.

1509, 1250.76929, -120., 2680.

1510, 1250.76929, -120., 2660.

1511, 1250.76929, -120., 2640.

1512, 1250.76929, -120., 2620.

1513, 1250.76929, -120., 2600.

1514, 1250.76929, -120., 2580.

1515, 1250.76929, -120., 2560.

1516, 1250.76929, -120., 2540.

1517, 1281.02563, -120., 2740.

1518, 1281.02563, -120., 2720.

1519, 1281.02563, -120., 2700.

1520, 1281.02563, -120., 2680.

1521, 1281.02563, -120., 2660.

1522, 1281.02563, -120., 2640.

1523, 1281.02563, -120., 2620.

1524, 1281.02563, -120., 2600.

1525, 1281.02563, -120., 2580.

1526, 1281.02563, -120., 2560.

1527, 1281.02563, -120., 2540.

1528, 1311.2821, -120., 2740.

1529, 1311.2821, -120., 2720.

1530, 1311.2821, -120., 2700.

1531, 1311.2821, -120., 2680.

1532, 1311.2821, -120., 2660.

1533, 1311.2821, -120., 2640.

1534, 1311.2821, -120., 2620.

1535, 1311.2821, -120., 2600.

1536, 1311.2821, -120., 2580.

1537, 1311.2821, -120., 2560.

1538, 1311.2821, -120., 2540.

1539, 1341.53845, -120., 2740.

1540, 1341.53845, -120., 2720.

1541, 1341.53845, -120., 2700.

1542, 1341.53845, -120., 2680.

1543, 1341.53845, -120., 2660.

1544, 1341.53845, -120., 2640.

1545, 1341.53845, -120., 2620.

1546, 1341.53845, -120., 2600.

1547, 1341.53845, -120., 2580.

1548, 1341.53845, -120., 2560.

1549, 1341.53845, -120., 2540.

1550, 1371.79492, -120., 2740.

1551, 1371.79492, -120., 2720.

1552, 1371.79492, -120., 2700.

1553, 1371.79492, -120., 2680.

1554, 1371.79492, -120., 2660.

1555, 1371.79492, -120., 2640.

1556, 1371.79492, -120., 2620.

1557, 1371.79492, -120., 2600.

1558, 1371.79492, -120., 2580.

1559, 1371.79492, -120., 2560.

1560, 1371.79492, -120., 2540.

1561, 1402.05127, -120., 2740.

1562, 1402.05127, -120., 2720.

1563, 1402.05127, -120., 2700.

1564, 1402.05127, -120., 2680.

1565, 1402.05127, -120., 2660.

1566, 1402.05127, -120., 2640.

1567, 1402.05127, -120., 2620.

1568, 1402.05127, -120., 2600.

1569, 1402.05127, -120., 2580.

1570, 1402.05127, -120., 2560.

1571, 1402.05127, -120., 2540.

1572, 1432.30774, -120., 2740.

1573, 1432.30774, -120., 2720.

1574, 1432.30774, -120., 2700.

1575, 1432.30774, -120., 2680.

1576, 1432.30774, -120., 2660.

1577, 1432.30774, -120., 2640.

1578, 1432.30774, -120., 2620.

1579, 1432.30774, -120., 2600.

1580, 1432.30774, -120., 2580.

1581, 1432.30774, -120., 2560.

1582, 1432.30774, -120., 2540.

1583, 1462.56409, -120., 2740.

1584, 1462.56409, -120., 2720.

1585, 1462.56409, -120., 2700.

1586, 1462.56409, -120., 2680.

1587, 1462.56409, -120., 2660.

1588, 1462.56409, -120., 2640.

1589, 1462.56409, -120., 2620.

1590, 1462.56409, -120., 2600.

1591, 1462.56409, -120., 2580.

1592, 1462.56409, -120., 2560.

1593, 1462.56409, -120., 2540.

1594, 1492.82056, -120., 2740.

1595, 1492.82056, -120., 2720.

1596, 1492.82056, -120., 2700.

1597, 1492.82056, -120., 2680.

1598, 1492.82056, -120., 2660.

1599, 1492.82056, -120., 2640.

1600, 1492.82056, -120., 2620.

1601, 1492.82056, -120., 2600.

1602, 1492.82056, -120., 2580.

1603, 1492.82056, -120., 2560.

1604, 1492.82056, -120., 2540.

1605, 1523.0769, -120., 2740.

1606, 1523.0769, -120., 2720.

1607, 1523.0769, -120., 2700.

1608, 1523.0769, -120., 2680.

1609, 1523.0769, -120., 2660.

1610, 1523.0769, -120., 2640.

1611, 1523.0769, -120., 2620.

1612, 1523.0769, -120., 2600.

1613, 1523.0769, -120., 2580.

1614, 1523.0769, -120., 2560.

1615, 1523.0769, -120., 2540.

1616, 1553.33337, -120., 2740.

1617, 1553.33337, -120., 2720.

1618, 1553.33337, -120., 2700.

1619, 1553.33337, -120., 2680.

1620, 1553.33337, -120., 2660.

1621, 1553.33337, -120., 2640.

1622, 1553.33337, -120., 2620.

1623, 1553.33337, -120., 2600.

1624, 1553.33337, -120., 2580.

1625, 1553.33337, -120., 2560.

1626, 1553.33337, -120., 2540.

1627, 1583.58972, -120., 2740.

1628, 1583.58972, -120., 2720.

1629, 1583.58972, -120., 2700.

1630, 1583.58972, -120., 2680.

1631, 1583.58972, -120., 2660.

1632, 1583.58972, -120., 2640.

1633, 1583.58972, -120., 2620.

1634, 1583.58972, -120., 2600.

1635, 1583.58972, -120., 2580.

1636, 1583.58972, -120., 2560.

1637, 1583.58972, -120., 2540.

1638, 1613.84619, -120., 2740.

1639, 1613.84619, -120., 2720.

1640, 1613.84619, -120., 2700.

1641, 1613.84619, -120., 2680.

1642, 1613.84619, -120., 2660.

1643, 1613.84619, -120., 2640.

1644, 1613.84619, -120., 2620.

1645, 1613.84619, -120., 2600.

1646, 1613.84619, -120., 2580.

1647, 1613.84619, -120., 2560.

1648, 1613.84619, -120., 2540.

1649, 1644.10254, -120., 2740.

1650, 1644.10254, -120., 2720.

1651, 1644.10254, -120., 2700.

1652, 1644.10254, -120., 2680.

1653, 1644.10254, -120., 2660.

1654, 1644.10254, -120., 2640.

1655, 1644.10254, -120., 2620.

1656, 1644.10254, -120., 2600.

1657, 1644.10254, -120., 2580.

1658, 1644.10254, -120., 2560.

1659, 1644.10254, -120., 2540.

1660, 1674.35901, -120., 2740.

1661, 1674.35901, -120., 2720.

1662, 1674.35901, -120., 2700.

1663, 1674.35901, -120., 2680.

1664, 1674.35901, -120., 2660.

1665, 1674.35901, -120., 2640.

1666, 1674.35901, -120., 2620.

1667, 1674.35901, -120., 2600.

1668, 1674.35901, -120., 2580.

1669, 1674.35901, -120., 2560.

1670, 1674.35901, -120., 2540.

1671, 1704.61536, -120., 2740.

1672, 1704.61536, -120., 2720.

1673, 1704.61536, -120., 2700.

1674, 1704.61536, -120., 2680.

1675, 1704.61536, -120., 2660.

1676, 1704.61536, -120., 2640.

1677, 1704.61536, -120., 2620.

1678, 1704.61536, -120., 2600.

1679, 1704.61536, -120., 2580.

1680, 1704.61536, -120., 2560.

1681, 1704.61536, -120., 2540.

1682, 1734.87183, -120., 2740.

1683, 1734.87183, -120., 2720.

1684, 1734.87183, -120., 2700.

1685, 1734.87183, -120., 2680.

1686, 1734.87183, -120., 2660.

1687, 1734.87183, -120., 2640.

1688, 1734.87183, -120., 2620.

1689, 1734.87183, -120., 2600.

1690, 1734.87183, -120., 2580.

1691, 1734.87183, -120., 2560.

1692, 1734.87183, -120., 2540.

1693, 1765.12817, -120., 2740.

1694, 1765.12817, -120., 2720.

1695, 1765.12817, -120., 2700.

1696, 1765.12817, -120., 2680.

1697, 1765.12817, -120., 2660.

1698, 1765.12817, -120., 2640.

1699, 1765.12817, -120., 2620.

1700, 1765.12817, -120., 2600.

1701, 1765.12817, -120., 2580.

1702, 1765.12817, -120., 2560.

1703, 1765.12817, -120., 2540.

1704, 1795.38464, -120., 2740.

1705, 1795.38464, -120., 2720.

1706, 1795.38464, -120., 2700.

1707, 1795.38464, -120., 2680.

1708, 1795.38464, -120., 2660.

1709, 1795.38464, -120., 2640.

1710, 1795.38464, -120., 2620.

1711, 1795.38464, -120., 2600.

1712, 1795.38464, -120., 2580.

1713, 1795.38464, -120., 2560.

1714, 1795.38464, -120., 2540.

1715, 1825.64099, -120., 2740.

1716, 1825.64099, -120., 2720.

1717, 1825.64099, -120., 2700.

1718, 1825.64099, -120., 2680.

1719, 1825.64099, -120., 2660.

1720, 1825.64099, -120., 2640.

1721, 1825.64099, -120., 2620.

1722, 1825.64099, -120., 2600.

1723, 1825.64099, -120., 2580.

1724, 1825.64099, -120., 2560.

1725, 1825.64099, -120., 2540.

1726, 1855.89746, -120., 2740.

1727, 1855.89746, -120., 2720.

1728, 1855.89746, -120., 2700.

1729, 1855.89746, -120., 2680.

1730, 1855.89746, -120., 2660.

1731, 1855.89746, -120., 2640.

1732, 1855.89746, -120., 2620.

1733, 1855.89746, -120., 2600.

1734, 1855.89746, -120., 2580.

1735, 1855.89746, -120., 2560.

1736, 1855.89746, -120., 2540.

1737, 1886.15381, -120., 2740.

1738, 1886.15381, -120., 2720.

1739, 1886.15381, -120., 2700.

1740, 1886.15381, -120., 2680.

1741, 1886.15381, -120., 2660.

1742, 1886.15381, -120., 2640.

1743, 1886.15381, -120., 2620.

1744, 1886.15381, -120., 2600.

1745, 1886.15381, -120., 2580.

1746, 1886.15381, -120., 2560.

1747, 1886.15381, -120., 2540.

1748, 1916.41028, -120., 2740.

1749, 1916.41028, -120., 2720.

1750, 1916.41028, -120., 2700.

1751, 1916.41028, -120., 2680.

1752, 1916.41028, -120., 2660.

1753, 1916.41028, -120., 2640.

1754, 1916.41028, -120., 2620.

1755, 1916.41028, -120., 2600.

1756, 1916.41028, -120., 2580.

1757, 1916.41028, -120., 2560.

1758, 1916.41028, -120., 2540.

1759, 1946.66663, -120., 2740.

1760, 1946.66663, -120., 2720.

1761, 1946.66663, -120., 2700.

1762, 1946.66663, -120., 2680.

1763, 1946.66663, -120., 2660.

1764, 1946.66663, -120., 2640.

1765, 1946.66663, -120., 2620.

1766, 1946.66663, -120., 2600.

1767, 1946.66663, -120., 2580.

1768, 1946.66663, -120., 2560.

1769, 1946.66663, -120., 2540.

1770, 1976.9231, -120., 2740.

1771, 1976.9231, -120., 2720.

1772, 1976.9231, -120., 2700.

1773, 1976.9231, -120., 2680.

1774, 1976.9231, -120., 2660.

1775, 1976.9231, -120., 2640.

1776, 1976.9231, -120., 2620.

1777, 1976.9231, -120., 2600.

1778, 1976.9231, -120., 2580.

1779, 1976.9231, -120., 2560.

1780, 1976.9231, -120., 2540.

1781, 2007.17944, -120., 2740.

1782, 2007.17944, -120., 2720.

1783, 2007.17944, -120., 2700.

1784, 2007.17944, -120., 2680.

1785, 2007.17944, -120., 2660.

1786, 2007.17944, -120., 2640.

1787, 2007.17944, -120., 2620.

1788, 2007.17944, -120., 2600.

1789, 2007.17944, -120., 2580.

1790, 2007.17944, -120., 2560.

1791, 2007.17944, -120., 2540.

1792, 2037.43591, -120., 2740.

1793, 2037.43591, -120., 2720.

1794, 2037.43591, -120., 2700.

1795, 2037.43591, -120., 2680.

1796, 2037.43591, -120., 2660.

1797, 2037.43591, -120., 2640.

1798, 2037.43591, -120., 2620.

1799, 2037.43591, -120., 2600.

1800, 2037.43591, -120., 2580.

1801, 2037.43591, -120., 2560.

1802, 2037.43591, -120., 2540.

1803, 2067.69238, -120., 2740.

1804, 2067.69238, -120., 2720.

1805, 2067.69238, -120., 2700.

1806, 2067.69238, -120., 2680.

1807, 2067.69238, -120., 2660.

1808, 2067.69238, -120., 2640.

1809, 2067.69238, -120., 2620.

1810, 2067.69238, -120., 2600.

1811, 2067.69238, -120., 2580.

1812, 2067.69238, -120., 2560.

1813, 2067.69238, -120., 2540.

1814, 2097.94873, -120., 2740.

1815, 2097.94873, -120., 2720.

1816, 2097.94873, -120., 2700.

1817, 2097.94873, -120., 2680.

1818, 2097.94873, -120., 2660.

1819, 2097.94873, -120., 2640.

1820, 2097.94873, -120., 2620.

1821, 2097.94873, -120., 2600.

1822, 2097.94873, -120., 2580.

1823, 2097.94873, -120., 2560.

1824, 2097.94873, -120., 2540.

1825, 2128.20508, -120., 2740.

1826, 2128.20508, -120., 2720.

1827, 2128.20508, -120., 2700.

1828, 2128.20508, -120., 2680.

1829, 2128.20508, -120., 2660.

1830, 2128.20508, -120., 2640.

1831, 2128.20508, -120., 2620.

1832, 2128.20508, -120., 2600.

1833, 2128.20508, -120., 2580.

1834, 2128.20508, -120., 2560.

1835, 2128.20508, -120., 2540.

1836, 2158.46143, -120., 2740.

1837, 2158.46143, -120., 2720.

1838, 2158.46143, -120., 2700.

1839, 2158.46143, -120., 2680.

1840, 2158.46143, -120., 2660.

1841, 2158.46143, -120., 2640.

1842, 2158.46143, -120., 2620.

1843, 2158.46143, -120., 2600.

1844, 2158.46143, -120., 2580.

1845, 2158.46143, -120., 2560.

1846, 2158.46143, -120., 2540.

1847, 2188.71802, -120., 2740.

1848, 2188.71802, -120., 2720.

1849, 2188.71802, -120., 2700.

1850, 2188.71802, -120., 2680.

1851, 2188.71802, -120., 2660.

1852, 2188.71802, -120., 2640.

1853, 2188.71802, -120., 2620.

1854, 2188.71802, -120., 2600.

1855, 2188.71802, -120., 2580.

1856, 2188.71802, -120., 2560.

1857, 2188.71802, -120., 2540.

1858, 2218.97437, -120., 2740.

1859, 2218.97437, -120., 2720.

1860, 2218.97437, -120., 2700.

1861, 2218.97437, -120., 2680.

1862, 2218.97437, -120., 2660.

1863, 2218.97437, -120., 2640.

1864, 2218.97437, -120., 2620.

1865, 2218.97437, -120., 2600.

1866, 2218.97437, -120., 2580.

1867, 2218.97437, -120., 2560.

1868, 2218.97437, -120., 2540.

1869, 2249.23071, -120., 2740.

1870, 2249.23071, -120., 2720.

1871, 2249.23071, -120., 2700.

1872, 2249.23071, -120., 2680.

1873, 2249.23071, -120., 2660.

1874, 2249.23071, -120., 2640.

1875, 2249.23071, -120., 2620.

1876, 2249.23071, -120., 2600.

1877, 2249.23071, -120., 2580.

1878, 2249.23071, -120., 2560.

1879, 2249.23071, -120., 2540.

1880, 2279.48706, -120., 2740.

1881, 2279.48706, -120., 2720.

1882, 2279.48706, -120., 2700.

1883, 2279.48706, -120., 2680.

1884, 2279.48706, -120., 2660.

1885, 2279.48706, -120., 2640.

1886, 2279.48706, -120., 2620.

1887, 2279.48706, -120., 2600.

1888, 2279.48706, -120., 2580.

1889, 2279.48706, -120., 2560.

1890, 2279.48706, -120., 2540.

1891, 2309.74365, -120., 2740.

1892, 2309.74365, -120., 2720.

1893, 2309.74365, -120., 2700.

1894, 2309.74365, -120., 2680.

1895, 2309.74365, -120., 2660.

1896, 2309.74365, -120., 2640.

1897, 2309.74365, -120., 2620.

1898, 2309.74365, -120., 2600.

1899, 2309.74365, -120., 2580.

1900, 2309.74365, -120., 2560.

1901, 2309.74365, -120., 2540.

1902, 1190.25647, 0., 2740.

1903, 1190.25647, 0., 2720.

1904, 1190.25647, 0., 2700.

1905, 1190.25647, 0., 2680.

1906, 1190.25647, 0., 2660.

1907, 1190.25647, 0., 2640.

1908, 1190.25647, 0., 2620.

1909, 1190.25647, 0., 2600.

1910, 1190.25647, 0., 2580.

1911, 1190.25647, 0., 2560.

1912, 1190.25647, 0., 2540.

1913, 1220.51282, 0., 2740.

1914, 1220.51282, 0., 2720.

1915, 1220.51282, 0., 2700.

1916, 1220.51282, 0., 2680.

1917, 1220.51282, 0., 2660.

1918, 1220.51282, 0., 2640.

1919, 1220.51282, 0., 2620.

1920, 1220.51282, 0., 2600.

1921, 1220.51282, 0., 2580.

1922, 1220.51282, 0., 2560.

1923, 1220.51282, 0., 2540.

1924, 1250.76929, 0., 2740.

1925, 1250.76929, 0., 2720.

1926, 1250.76929, 0., 2700.

1927, 1250.76929, 0., 2680.

1928, 1250.76929, 0., 2660.

1929, 1250.76929, 0., 2640.

1930, 1250.76929, 0., 2620.

1931, 1250.76929, 0., 2600.

1932, 1250.76929, 0., 2580.

1933, 1250.76929, 0., 2560.

1934, 1250.76929, 0., 2540.

1935, 1281.02563, 0., 2740.

1936, 1281.02563, 0., 2720.

1937, 1281.02563, 0., 2700.

1938, 1281.02563, 0., 2680.

1939, 1281.02563, 0., 2660.

1940, 1281.02563, 0., 2640.

1941, 1281.02563, 0., 2620.

1942, 1281.02563, 0., 2600.

1943, 1281.02563, 0., 2580.

1944, 1281.02563, 0., 2560.

1945, 1281.02563, 0., 2540.

1946, 1311.2821, 0., 2740.

1947, 1311.2821, 0., 2720.

1948, 1311.2821, 0., 2700.

1949, 1311.2821, 0., 2680.

1950, 1311.2821, 0., 2660.

1951, 1311.2821, 0., 2640.

1952, 1311.2821, 0., 2620.

1953, 1311.2821, 0., 2600.

1954, 1311.2821, 0., 2580.

1955, 1311.2821, 0., 2560.

1956, 1311.2821, 0., 2540.

1957, 1341.53845, 0., 2740.

1958, 1341.53845, 0., 2720.

1959, 1341.53845, 0., 2700.

1960, 1341.53845, 0., 2680.

1961, 1341.53845, 0., 2660.

1962, 1341.53845, 0., 2640.

1963, 1341.53845, 0., 2620.

1964, 1341.53845, 0., 2600.

1965, 1341.53845, 0., 2580.

1966, 1341.53845, 0., 2560.

1967, 1341.53845, 0., 2540.

1968, 1371.79492, 0., 2740.

1969, 1371.79492, 0., 2720.

1970, 1371.79492, 0., 2700.

1971, 1371.79492, 0., 2680.

1972, 1371.79492, 0., 2660.

1973, 1371.79492, 0., 2640.

1974, 1371.79492, 0., 2620.

1975, 1371.79492, 0., 2600.

1976, 1371.79492, 0., 2580.

1977, 1371.79492, 0., 2560.

1978, 1371.79492, 0., 2540.

1979, 1402.05127, 0., 2740.

1980, 1402.05127, 0., 2720.

1981, 1402.05127, 0., 2700.

1982, 1402.05127, 0., 2680.

1983, 1402.05127, 0., 2660.

1984, 1402.05127, 0., 2640.

1985, 1402.05127, 0., 2620.

1986, 1402.05127, 0., 2600.

1987, 1402.05127, 0., 2580.

1988, 1402.05127, 0., 2560.

1989, 1402.05127, 0., 2540.

1990, 1432.30774, 0., 2740.

1991, 1432.30774, 0., 2720.

1992, 1432.30774, 0., 2700.

1993, 1432.30774, 0., 2680.

1994, 1432.30774, 0., 2660.

1995, 1432.30774, 0., 2640.

1996, 1432.30774, 0., 2620.

1997, 1432.30774, 0., 2600.

1998, 1432.30774, 0., 2580.

1999, 1432.30774, 0., 2560.

2000, 1432.30774, 0., 2540.

2001, 1462.56409, 0., 2740.

2002, 1462.56409, 0., 2720.

2003, 1462.56409, 0., 2700.

2004, 1462.56409, 0., 2680.

2005, 1462.56409, 0., 2660.

2006, 1462.56409, 0., 2640.

2007, 1462.56409, 0., 2620.

2008, 1462.56409, 0., 2600.

2009, 1462.56409, 0., 2580.

2010, 1462.56409, 0., 2560.

2011, 1462.56409, 0., 2540.

2012, 1492.82056, 0., 2740.

2013, 1492.82056, 0., 2720.

2014, 1492.82056, 0., 2700.

2015, 1492.82056, 0., 2680.

2016, 1492.82056, 0., 2660.

2017, 1492.82056, 0., 2640.

2018, 1492.82056, 0., 2620.

2019, 1492.82056, 0., 2600.

2020, 1492.82056, 0., 2580.

2021, 1492.82056, 0., 2560.

2022, 1492.82056, 0., 2540.

2023, 1523.0769, 0., 2740.

2024, 1523.0769, 0., 2720.

2025, 1523.0769, 0., 2700.

2026, 1523.0769, 0., 2680.

2027, 1523.0769, 0., 2660.

2028, 1523.0769, 0., 2640.

2029, 1523.0769, 0., 2620.

2030, 1523.0769, 0., 2600.

2031, 1523.0769, 0., 2580.

2032, 1523.0769, 0., 2560.

2033, 1523.0769, 0., 2540.

2034, 1553.33337, 0., 2740.

2035, 1553.33337, 0., 2720.

2036, 1553.33337, 0., 2700.

2037, 1553.33337, 0., 2680.

2038, 1553.33337, 0., 2660.

2039, 1553.33337, 0., 2640.

2040, 1553.33337, 0., 2620.

2041, 1553.33337, 0., 2600.

2042, 1553.33337, 0., 2580.

2043, 1553.33337, 0., 2560.

2044, 1553.33337, 0., 2540.

2045, 1583.58972, 0., 2740.

2046, 1583.58972, 0., 2720.

2047, 1583.58972, 0., 2700.

2048, 1583.58972, 0., 2680.

2049, 1583.58972, 0., 2660.

2050, 1583.58972, 0., 2640.

2051, 1583.58972, 0., 2620.

2052, 1583.58972, 0., 2600.

2053, 1583.58972, 0., 2580.

2054, 1583.58972, 0., 2560.

2055, 1583.58972, 0., 2540.

2056, 1613.84619, 0., 2740.

2057, 1613.84619, 0., 2720.

2058, 1613.84619, 0., 2700.

2059, 1613.84619, 0., 2680.

2060, 1613.84619, 0., 2660.

2061, 1613.84619, 0., 2640.

2062, 1613.84619, 0., 2620.

2063, 1613.84619, 0., 2600.

2064, 1613.84619, 0., 2580.

2065, 1613.84619, 0., 2560.

2066, 1613.84619, 0., 2540.

2067, 1644.10254, 0., 2740.

2068, 1644.10254, 0., 2720.

2069, 1644.10254, 0., 2700.

2070, 1644.10254, 0., 2680.

2071, 1644.10254, 0., 2660.

2072, 1644.10254, 0., 2640.

2073, 1644.10254, 0., 2620.

2074, 1644.10254, 0., 2600.

2075, 1644.10254, 0., 2580.

2076, 1644.10254, 0., 2560.

2077, 1644.10254, 0., 2540.

2078, 1674.35901, 0., 2740.

2079, 1674.35901, 0., 2720.

2080, 1674.35901, 0., 2700.

2081, 1674.35901, 0., 2680.

2082, 1674.35901, 0., 2660.

2083, 1674.35901, 0., 2640.

2084, 1674.35901, 0., 2620.

2085, 1674.35901, 0., 2600.

2086, 1674.35901, 0., 2580.

2087, 1674.35901, 0., 2560.

2088, 1674.35901, 0., 2540.

2089, 1704.61536, 0., 2740.

2090, 1704.61536, 0., 2720.

2091, 1704.61536, 0., 2700.

2092, 1704.61536, 0., 2680.

2093, 1704.61536, 0., 2660.

2094, 1704.61536, 0., 2640.

2095, 1704.61536, 0., 2620.

2096, 1704.61536, 0., 2600.

2097, 1704.61536, 0., 2580.

2098, 1704.61536, 0., 2560.

2099, 1704.61536, 0., 2540.

2100, 1734.87183, 0., 2740.

2101, 1734.87183, 0., 2720.

2102, 1734.87183, 0., 2700.

2103, 1734.87183, 0., 2680.

2104, 1734.87183, 0., 2660.

2105, 1734.87183, 0., 2640.

2106, 1734.87183, 0., 2620.

2107, 1734.87183, 0., 2600.

2108, 1734.87183, 0., 2580.

2109, 1734.87183, 0., 2560.

2110, 1734.87183, 0., 2540.

2111, 1765.12817, 0., 2740.

2112, 1765.12817, 0., 2720.

2113, 1765.12817, 0., 2700.

2114, 1765.12817, 0., 2680.

2115, 1765.12817, 0., 2660.

2116, 1765.12817, 0., 2640.

2117, 1765.12817, 0., 2620.

2118, 1765.12817, 0., 2600.

2119, 1765.12817, 0., 2580.

2120, 1765.12817, 0., 2560.

2121, 1765.12817, 0., 2540.

2122, 1795.38464, 0., 2740.

2123, 1795.38464, 0., 2720.

2124, 1795.38464, 0., 2700.

2125, 1795.38464, 0., 2680.

2126, 1795.38464, 0., 2660.

2127, 1795.38464, 0., 2640.

2128, 1795.38464, 0., 2620.

2129, 1795.38464, 0., 2600.

2130, 1795.38464, 0., 2580.

2131, 1795.38464, 0., 2560.

2132, 1795.38464, 0., 2540.

2133, 1825.64099, 0., 2740.

2134, 1825.64099, 0., 2720.

2135, 1825.64099, 0., 2700.

2136, 1825.64099, 0., 2680.

2137, 1825.64099, 0., 2660.

2138, 1825.64099, 0., 2640.

2139, 1825.64099, 0., 2620.

2140, 1825.64099, 0., 2600.

2141, 1825.64099, 0., 2580.

2142, 1825.64099, 0., 2560.

2143, 1825.64099, 0., 2540.

2144, 1855.89746, 0., 2740.

2145, 1855.89746, 0., 2720.

2146, 1855.89746, 0., 2700.

2147, 1855.89746, 0., 2680.

2148, 1855.89746, 0., 2660.

2149, 1855.89746, 0., 2640.

2150, 1855.89746, 0., 2620.

2151, 1855.89746, 0., 2600.

2152, 1855.89746, 0., 2580.

2153, 1855.89746, 0., 2560.

2154, 1855.89746, 0., 2540.

2155, 1886.15381, 0., 2740.

2156, 1886.15381, 0., 2720.

2157, 1886.15381, 0., 2700.

2158, 1886.15381, 0., 2680.

2159, 1886.15381, 0., 2660.

2160, 1886.15381, 0., 2640.

2161, 1886.15381, 0., 2620.

2162, 1886.15381, 0., 2600.

2163, 1886.15381, 0., 2580.

2164, 1886.15381, 0., 2560.

2165, 1886.15381, 0., 2540.

2166, 1916.41028, 0., 2740.

2167, 1916.41028, 0., 2720.

2168, 1916.41028, 0., 2700.

2169, 1916.41028, 0., 2680.

2170, 1916.41028, 0., 2660.

2171, 1916.41028, 0., 2640.

2172, 1916.41028, 0., 2620.

2173, 1916.41028, 0., 2600.

2174, 1916.41028, 0., 2580.

2175, 1916.41028, 0., 2560.

2176, 1916.41028, 0., 2540.

2177, 1946.66663, 0., 2740.

2178, 1946.66663, 0., 2720.

2179, 1946.66663, 0., 2700.

2180, 1946.66663, 0., 2680.

2181, 1946.66663, 0., 2660.

2182, 1946.66663, 0., 2640.

2183, 1946.66663, 0., 2620.

2184, 1946.66663, 0., 2600.

2185, 1946.66663, 0., 2580.

2186, 1946.66663, 0., 2560.

2187, 1946.66663, 0., 2540.

2188, 1976.9231, 0., 2740.

2189, 1976.9231, 0., 2720.

2190, 1976.9231, 0., 2700.

2191, 1976.9231, 0., 2680.

2192, 1976.9231, 0., 2660.

2193, 1976.9231, 0., 2640.

2194, 1976.9231, 0., 2620.

2195, 1976.9231, 0., 2600.

2196, 1976.9231, 0., 2580.

2197, 1976.9231, 0., 2560.

2198, 1976.9231, 0., 2540.

2199, 2007.17944, 0., 2740.

2200, 2007.17944, 0., 2720.

2201, 2007.17944, 0., 2700.

2202, 2007.17944, 0., 2680.

2203, 2007.17944, 0., 2660.

2204, 2007.17944, 0., 2640.

2205, 2007.17944, 0., 2620.

2206, 2007.17944, 0., 2600.

2207, 2007.17944, 0., 2580.

2208, 2007.17944, 0., 2560.

2209, 2007.17944, 0., 2540.

2210, 2037.43591, 0., 2740.

2211, 2037.43591, 0., 2720.

2212, 2037.43591, 0., 2700.

2213, 2037.43591, 0., 2680.

2214, 2037.43591, 0., 2660.

2215, 2037.43591, 0., 2640.

2216, 2037.43591, 0., 2620.

2217, 2037.43591, 0., 2600.

2218, 2037.43591, 0., 2580.

2219, 2037.43591, 0., 2560.

2220, 2037.43591, 0., 2540.

2221, 2067.69238, 0., 2740.

2222, 2067.69238, 0., 2720.

2223, 2067.69238, 0., 2700.

2224, 2067.69238, 0., 2680.

2225, 2067.69238, 0., 2660.

2226, 2067.69238, 0., 2640.

2227, 2067.69238, 0., 2620.

2228, 2067.69238, 0., 2600.

2229, 2067.69238, 0., 2580.

2230, 2067.69238, 0., 2560.

2231, 2067.69238, 0., 2540.

2232, 2097.94873, 0., 2740.

2233, 2097.94873, 0., 2720.

2234, 2097.94873, 0., 2700.

2235, 2097.94873, 0., 2680.

2236, 2097.94873, 0., 2660.

2237, 2097.94873, 0., 2640.

2238, 2097.94873, 0., 2620.

2239, 2097.94873, 0., 2600.

2240, 2097.94873, 0., 2580.

2241, 2097.94873, 0., 2560.

2242, 2097.94873, 0., 2540.

2243, 2128.20508, 0., 2740.

2244, 2128.20508, 0., 2720.

2245, 2128.20508, 0., 2700.

2246, 2128.20508, 0., 2680.

2247, 2128.20508, 0., 2660.

2248, 2128.20508, 0., 2640.

2249, 2128.20508, 0., 2620.

2250, 2128.20508, 0., 2600.

2251, 2128.20508, 0., 2580.

2252, 2128.20508, 0., 2560.

2253, 2128.20508, 0., 2540.

2254, 2158.46143, 0., 2740.

2255, 2158.46143, 0., 2720.

2256, 2158.46143, 0., 2700.

2257, 2158.46143, 0., 2680.

2258, 2158.46143, 0., 2660.

2259, 2158.46143, 0., 2640.

2260, 2158.46143, 0., 2620.

2261, 2158.46143, 0., 2600.

2262, 2158.46143, 0., 2580.

2263, 2158.46143, 0., 2560.

2264, 2158.46143, 0., 2540.

2265, 2188.71802, 0., 2740.

2266, 2188.71802, 0., 2720.

2267, 2188.71802, 0., 2700.

2268, 2188.71802, 0., 2680.

2269, 2188.71802, 0., 2660.

2270, 2188.71802, 0., 2640.

2271, 2188.71802, 0., 2620.

2272, 2188.71802, 0., 2600.

2273, 2188.71802, 0., 2580.

2274, 2188.71802, 0., 2560.

2275, 2188.71802, 0., 2540.

2276, 2218.97437, 0., 2740.

2277, 2218.97437, 0., 2720.

2278, 2218.97437, 0., 2700.

2279, 2218.97437, 0., 2680.

2280, 2218.97437, 0., 2660.

2281, 2218.97437, 0., 2640.

2282, 2218.97437, 0., 2620.

2283, 2218.97437, 0., 2600.

2284, 2218.97437, 0., 2580.

2285, 2218.97437, 0., 2560.

2286, 2218.97437, 0., 2540.

2287, 2249.23071, 0., 2740.

2288, 2249.23071, 0., 2720.

2289, 2249.23071, 0., 2700.

2290, 2249.23071, 0., 2680.

2291, 2249.23071, 0., 2660.

2292, 2249.23071, 0., 2640.

2293, 2249.23071, 0., 2620.

2294, 2249.23071, 0., 2600.

2295, 2249.23071, 0., 2580.

2296, 2249.23071, 0., 2560.

2297, 2249.23071, 0., 2540.

2298, 2279.48706, 0., 2740.

2299, 2279.48706, 0., 2720.

2300, 2279.48706, 0., 2700.

2301, 2279.48706, 0., 2680.

2302, 2279.48706, 0., 2660.

2303, 2279.48706, 0., 2640.

2304, 2279.48706, 0., 2620.

2305, 2279.48706, 0., 2600.

2306, 2279.48706, 0., 2580.

2307, 2279.48706, 0., 2560.

2308, 2279.48706, 0., 2540.

2309, 2309.74365, 0., 2740.

2310, 2309.74365, 0., 2720.

2311, 2309.74365, 0., 2700.

2312, 2309.74365, 0., 2680.

2313, 2309.74365, 0., 2660.

2314, 2309.74365, 0., 2640.

2315, 2309.74365, 0., 2620.

2316, 2309.74365, 0., 2600.

2317, 2309.74365, 0., 2580.

2318, 2309.74365, 0., 2560.

2319, 2309.74365, 0., 2540.

2320, 1190.25647, -100., 2760.

2321, 1220.51282, -100., 2760.

2322, 1250.76929, -100., 2760.

2323, 1281.02563, -100., 2760.

2324, 1311.2821, -100., 2760.

2325, 1341.53845, -100., 2760.

2326, 1371.79492, -100., 2760.

2327, 1402.05127, -100., 2760.

2328, 1432.30774, -100., 2760.

2329, 1462.56409, -100., 2760.

2330, 1492.82056, -100., 2760.

2331, 1523.0769, -100., 2760.

2332, 1553.33337, -100., 2760.

2333, 1583.58972, -100., 2760.

2334, 1613.84619, -100., 2760.

2335, 1644.10254, -100., 2760.

2336, 1674.35901, -100., 2760.

2337, 1704.61536, -100., 2760.

2338, 1734.87183, -100., 2760.

2339, 1765.12817, -100., 2760.

2340, 1795.38464, -100., 2760.

2341, 1825.64099, -100., 2760.

2342, 1855.89746, -100., 2760.

2343, 1886.15381, -100., 2760.

2344, 1916.41028, -100., 2760.

2345, 1946.66663, -100., 2760.

2346, 1976.9231, -100., 2760.

2347, 2007.17944, -100., 2760.

2348, 2037.43591, -100., 2760.

2349, 2067.69238, -100., 2760.

2350, 2097.94873, -100., 2760.

2351, 2128.20508, -100., 2760.

2352, 2158.46143, -100., 2760.

2353, 2188.71802, -100., 2760.

2354, 2218.97437, -100., 2760.

2355, 2249.23071, -100., 2760.

2356, 2279.48706, -100., 2760.

2357, 2309.74365, -100., 2760.

2358, 1190.25647, -80., 2760.

2359, 1220.51282, -80., 2760.

2360, 1250.76929, -80., 2760.

2361, 1281.02563, -80., 2760.

2362, 1311.2821, -80., 2760.

2363, 1341.53845, -80., 2760.

2364, 1371.79492, -80., 2760.

2365, 1402.05127, -80., 2760.

2366, 1432.30774, -80., 2760.

2367, 1462.56409, -80., 2760.

2368, 1492.82056, -80., 2760.

2369, 1523.0769, -80., 2760.

2370, 1553.33337, -80., 2760.

2371, 1583.58972, -80., 2760.

2372, 1613.84619, -80., 2760.

2373, 1644.10254, -80., 2760.

2374, 1674.35901, -80., 2760.

2375, 1704.61536, -80., 2760.

2376, 1734.87183, -80., 2760.

2377, 1765.12817, -80., 2760.

2378, 1795.38464, -80., 2760.

2379, 1825.64099, -80., 2760.

2380, 1855.89746, -80., 2760.

2381, 1886.15381, -80., 2760.

2382, 1916.41028, -80., 2760.

2383, 1946.66663, -80., 2760.

2384, 1976.9231, -80., 2760.

2385, 2007.17944, -80., 2760.

2386, 2037.43591, -80., 2760.

2387, 2067.69238, -80., 2760.

2388, 2097.94873, -80., 2760.

2389, 2128.20508, -80., 2760.

2390, 2158.46143, -80., 2760.

2391, 2188.71802, -80., 2760.

2392, 2218.97437, -80., 2760.

2393, 2249.23071, -80., 2760.

2394, 2279.48706, -80., 2760.

2395, 2309.74365, -80., 2760.

2396, 1190.25647, -60., 2760.

2397, 1220.51282, -60., 2760.

2398, 1250.76929, -60., 2760.

2399, 1281.02563, -60., 2760.

2400, 1311.2821, -60., 2760.

2401, 1341.53845, -60., 2760.

2402, 1371.79492, -60., 2760.

2403, 1402.05127, -60., 2760.

2404, 1432.30774, -60., 2760.

2405, 1462.56409, -60., 2760.

2406, 1492.82056, -60., 2760.

2407, 1523.0769, -60., 2760.

2408, 1553.33337, -60., 2760.

2409, 1583.58972, -60., 2760.

2410, 1613.84619, -60., 2760.

2411, 1644.10254, -60., 2760.

2412, 1674.35901, -60., 2760.

2413, 1704.61536, -60., 2760.

2414, 1734.87183, -60., 2760.

2415, 1765.12817, -60., 2760.

2416, 1795.38464, -60., 2760.

2417, 1825.64099, -60., 2760.

2418, 1855.89746, -60., 2760.

2419, 1886.15381, -60., 2760.

2420, 1916.41028, -60., 2760.

2421, 1946.66663, -60., 2760.

2422, 1976.9231, -60., 2760.

2423, 2007.17944, -60., 2760.

2424, 2037.43591, -60., 2760.

2425, 2067.69238, -60., 2760.

2426, 2097.94873, -60., 2760.

2427, 2128.20508, -60., 2760.

2428, 2158.46143, -60., 2760.

2429, 2188.71802, -60., 2760.

2430, 2218.97437, -60., 2760.

2431, 2249.23071, -60., 2760.

2432, 2279.48706, -60., 2760.

2433, 2309.74365, -60., 2760.

2434, 1190.25647, -40., 2760.

2435, 1220.51282, -40., 2760.

2436, 1250.76929, -40., 2760.

2437, 1281.02563, -40., 2760.

2438, 1311.2821, -40., 2760.

2439, 1341.53845, -40., 2760.

2440, 1371.79492, -40., 2760.

2441, 1402.05127, -40., 2760.

2442, 1432.30774, -40., 2760.

2443, 1462.56409, -40., 2760.

2444, 1492.82056, -40., 2760.

2445, 1523.0769, -40., 2760.

2446, 1553.33337, -40., 2760.

2447, 1583.58972, -40., 2760.

2448, 1613.84619, -40., 2760.

2449, 1644.10254, -40., 2760.

2450, 1674.35901, -40., 2760.

2451, 1704.61536, -40., 2760.

2452, 1734.87183, -40., 2760.

2453, 1765.12817, -40., 2760.

2454, 1795.38464, -40., 2760.

2455, 1825.64099, -40., 2760.

2456, 1855.89746, -40., 2760.

2457, 1886.15381, -40., 2760.

2458, 1916.41028, -40., 2760.

2459, 1946.66663, -40., 2760.

2460, 1976.9231, -40., 2760.

2461, 2007.17944, -40., 2760.

2462, 2037.43591, -40., 2760.

2463, 2067.69238, -40., 2760.

2464, 2097.94873, -40., 2760.

2465, 2128.20508, -40., 2760.

2466, 2158.46143, -40., 2760.

2467, 2188.71802, -40., 2760.

2468, 2218.97437, -40., 2760.

2469, 2249.23071, -40., 2760.

2470, 2279.48706, -40., 2760.

2471, 2309.74365, -40., 2760.

2472, 1190.25647, -20., 2760.

2473, 1220.51282, -20., 2760.

2474, 1250.76929, -20., 2760.

2475, 1281.02563, -20., 2760.

2476, 1311.2821, -20., 2760.

2477, 1341.53845, -20., 2760.

2478, 1371.79492, -20., 2760.

2479, 1402.05127, -20., 2760.

2480, 1432.30774, -20., 2760.

2481, 1462.56409, -20., 2760.

2482, 1492.82056, -20., 2760.

2483, 1523.0769, -20., 2760.

2484, 1553.33337, -20., 2760.

2485, 1583.58972, -20., 2760.

2486, 1613.84619, -20., 2760.

2487, 1644.10254, -20., 2760.

2488, 1674.35901, -20., 2760.

2489, 1704.61536, -20., 2760.

2490, 1734.87183, -20., 2760.

2491, 1765.12817, -20., 2760.

2492, 1795.38464, -20., 2760.

2493, 1825.64099, -20., 2760.

2494, 1855.89746, -20., 2760.

2495, 1886.15381, -20., 2760.

2496, 1916.41028, -20., 2760.

2497, 1946.66663, -20., 2760.

2498, 1976.9231, -20., 2760.

2499, 2007.17944, -20., 2760.

2500, 2037.43591, -20., 2760.

2501, 2067.69238, -20., 2760.

2502, 2097.94873, -20., 2760.

2503, 2128.20508, -20., 2760.

2504, 2158.46143, -20., 2760.

2505, 2188.71802, -20., 2760.

2506, 2218.97437, -20., 2760.

2507, 2249.23071, -20., 2760.

2508, 2279.48706, -20., 2760.

2509, 2309.74365, -20., 2760.

2510, 1190.25647, -100., 2520.

2511, 1220.51282, -100., 2520.

2512, 1250.76929, -100., 2520.

2513, 1281.02563, -100., 2520.

2514, 1311.2821, -100., 2520.

2515, 1341.53845, -100., 2520.

2516, 1371.79492, -100., 2520.

2517, 1402.05127, -100., 2520.

2518, 1432.30774, -100., 2520.

2519, 1462.56409, -100., 2520.

2520, 1492.82056, -100., 2520.

2521, 1523.0769, -100., 2520.

2522, 1553.33337, -100., 2520.

2523, 1583.58972, -100., 2520.

2524, 1613.84619, -100., 2520.

2525, 1644.10254, -100., 2520.

2526, 1674.35901, -100., 2520.

2527, 1704.61536, -100., 2520.

2528, 1734.87183, -100., 2520.

2529, 1765.12817, -100., 2520.

2530, 1795.38464, -100., 2520.

2531, 1825.64099, -100., 2520.

2532, 1855.89746, -100., 2520.

2533, 1886.15381, -100., 2520.

2534, 1916.41028, -100., 2520.

2535, 1946.66663, -100., 2520.

2536, 1976.9231, -100., 2520.

2537, 2007.17944, -100., 2520.

2538, 2037.43591, -100., 2520.

2539, 2067.69238, -100., 2520.

2540, 2097.94873, -100., 2520.

2541, 2128.20508, -100., 2520.

2542, 2158.46143, -100., 2520.

2543, 2188.71802, -100., 2520.

2544, 2218.97437, -100., 2520.

2545, 2249.23071, -100., 2520.

2546, 2279.48706, -100., 2520.

2547, 2309.74365, -100., 2520.

2548, 1190.25647, -80., 2520.

2549, 1220.51282, -80., 2520.

2550, 1250.76929, -80., 2520.

2551, 1281.02563, -80., 2520.

2552, 1311.2821, -80., 2520.

2553, 1341.53845, -80., 2520.

2554, 1371.79492, -80., 2520.

2555, 1402.05127, -80., 2520.

2556, 1432.30774, -80., 2520.

2557, 1462.56409, -80., 2520.

2558, 1492.82056, -80., 2520.

2559, 1523.0769, -80., 2520.

2560, 1553.33337, -80., 2520.

2561, 1583.58972, -80., 2520.

2562, 1613.84619, -80., 2520.

2563, 1644.10254, -80., 2520.

2564, 1674.35901, -80., 2520.

2565, 1704.61536, -80., 2520.

2566, 1734.87183, -80., 2520.

2567, 1765.12817, -80., 2520.

2568, 1795.38464, -80., 2520.

2569, 1825.64099, -80., 2520.

2570, 1855.89746, -80., 2520.

2571, 1886.15381, -80., 2520.

2572, 1916.41028, -80., 2520.

2573, 1946.66663, -80., 2520.

2574, 1976.9231, -80., 2520.

2575, 2007.17944, -80., 2520.

2576, 2037.43591, -80., 2520.

2577, 2067.69238, -80., 2520.

2578, 2097.94873, -80., 2520.

2579, 2128.20508, -80., 2520.

2580, 2158.46143, -80., 2520.

2581, 2188.71802, -80., 2520.

2582, 2218.97437, -80., 2520.

2583, 2249.23071, -80., 2520.

2584, 2279.48706, -80., 2520.

2585, 2309.74365, -80., 2520.

2586, 1190.25647, -60., 2520.

2587, 1220.51282, -60., 2520.

2588, 1250.76929, -60., 2520.

2589, 1281.02563, -60., 2520.

2590, 1311.2821, -60., 2520.

2591, 1341.53845, -60., 2520.

2592, 1371.79492, -60., 2520.

2593, 1402.05127, -60., 2520.

2594, 1432.30774, -60., 2520.

2595, 1462.56409, -60., 2520.

2596, 1492.82056, -60., 2520.

2597, 1523.0769, -60., 2520.

2598, 1553.33337, -60., 2520.

2599, 1583.58972, -60., 2520.

2600, 1613.84619, -60., 2520.

2601, 1644.10254, -60., 2520.

2602, 1674.35901, -60., 2520.

2603, 1704.61536, -60., 2520.

2604, 1734.87183, -60., 2520.

2605, 1765.12817, -60., 2520.

2606, 1795.38464, -60., 2520.

2607, 1825.64099, -60., 2520.

2608, 1855.89746, -60., 2520.

2609, 1886.15381, -60., 2520.

2610, 1916.41028, -60., 2520.

2611, 1946.66663, -60., 2520.

2612, 1976.9231, -60., 2520.

2613, 2007.17944, -60., 2520.

2614, 2037.43591, -60., 2520.

2615, 2067.69238, -60., 2520.

2616, 2097.94873, -60., 2520.

2617, 2128.20508, -60., 2520.

2618, 2158.46143, -60., 2520.

2619, 2188.71802, -60., 2520.

2620, 2218.97437, -60., 2520.

2621, 2249.23071, -60., 2520.

2622, 2279.48706, -60., 2520.

2623, 2309.74365, -60., 2520.

2624, 1190.25647, -40., 2520.

2625, 1220.51282, -40., 2520.

2626, 1250.76929, -40., 2520.

2627, 1281.02563, -40., 2520.

2628, 1311.2821, -40., 2520.

2629, 1341.53845, -40., 2520.

2630, 1371.79492, -40., 2520.

2631, 1402.05127, -40., 2520.

2632, 1432.30774, -40., 2520.

2633, 1462.56409, -40., 2520.

2634, 1492.82056, -40., 2520.

2635, 1523.0769, -40., 2520.

2636, 1553.33337, -40., 2520.

2637, 1583.58972, -40., 2520.

2638, 1613.84619, -40., 2520.

2639, 1644.10254, -40., 2520.

2640, 1674.35901, -40., 2520.

2641, 1704.61536, -40., 2520.

2642, 1734.87183, -40., 2520.

2643, 1765.12817, -40., 2520.

2644, 1795.38464, -40., 2520.

2645, 1825.64099, -40., 2520.

2646, 1855.89746, -40., 2520.

2647, 1886.15381, -40., 2520.

2648, 1916.41028, -40., 2520.

2649, 1946.66663, -40., 2520.

2650, 1976.9231, -40., 2520.

2651, 2007.17944, -40., 2520.

2652, 2037.43591, -40., 2520.

2653, 2067.69238, -40., 2520.

2654, 2097.94873, -40., 2520.

2655, 2128.20508, -40., 2520.

2656, 2158.46143, -40., 2520.

2657, 2188.71802, -40., 2520.

2658, 2218.97437, -40., 2520.

2659, 2249.23071, -40., 2520.

2660, 2279.48706, -40., 2520.

2661, 2309.74365, -40., 2520.

2662, 1190.25647, -20., 2520.

2663, 1220.51282, -20., 2520.

2664, 1250.76929, -20., 2520.

2665, 1281.02563, -20., 2520.

2666, 1311.2821, -20., 2520.

2667, 1341.53845, -20., 2520.

2668, 1371.79492, -20., 2520.

2669, 1402.05127, -20., 2520.

2670, 1432.30774, -20., 2520.

2671, 1462.56409, -20., 2520.

2672, 1492.82056, -20., 2520.

2673, 1523.0769, -20., 2520.

2674, 1553.33337, -20., 2520.

2675, 1583.58972, -20., 2520.

2676, 1613.84619, -20., 2520.

2677, 1644.10254, -20., 2520.

2678, 1674.35901, -20., 2520.

2679, 1704.61536, -20., 2520.

2680, 1734.87183, -20., 2520.

2681, 1765.12817, -20., 2520.

2682, 1795.38464, -20., 2520.

2683, 1825.64099, -20., 2520.

2684, 1855.89746, -20., 2520.

2685, 1886.15381, -20., 2520.

2686, 1916.41028, -20., 2520.

2687, 1946.66663, -20., 2520.

2688, 1976.9231, -20., 2520.

2689, 2007.17944, -20., 2520.

2690, 2037.43591, -20., 2520.

2691, 2067.69238, -20., 2520.

2692, 2097.94873, -20., 2520.

2693, 2128.20508, -20., 2520.

2694, 2158.46143, -20., 2520.

2695, 2188.71802, -20., 2520.

2696, 2218.97437, -20., 2520.

2697, 2249.23071, -20., 2520.

2698, 2279.48706, -20., 2520.

2699, 2309.74365, -20., 2520.

2700, 1160., -100., 2740.

2701, 1160., -100., 2720.

2702, 1160., -100., 2700.

2703, 1160., -100., 2680.

2704, 1160., -100., 2660.

2705, 1160., -100., 2640.

2706, 1160., -100., 2620.

2707, 1160., -100., 2600.

2708, 1160., -100., 2580.

2709, 1160., -100., 2560.

2710, 1160., -100., 2540.

2711, 1160., -80., 2740.

2712, 1160., -80., 2720.

2713, 1160., -80., 2700.

2714, 1160., -80., 2680.

2715, 1160., -80., 2660.

2716, 1160., -80., 2640.

2717, 1160., -80., 2620.

2718, 1160., -80., 2600.

2719, 1160., -80., 2580.

2720, 1160., -80., 2560.

2721, 1160., -80., 2540.

2722, 1160., -60., 2740.

2723, 1160., -60., 2720.

2724, 1160., -60., 2700.

2725, 1160., -60., 2680.

2726, 1160., -60., 2660.

2727, 1160., -60., 2640.

2728, 1160., -60., 2620.

2729, 1160., -60., 2600.

2730, 1160., -60., 2580.

2731, 1160., -60., 2560.

2732, 1160., -60., 2540.

2733, 1160., -40., 2740.

2734, 1160., -40., 2720.

2735, 1160., -40., 2700.

2736, 1160., -40., 2680.

2737, 1160., -40., 2660.

2738, 1160., -40., 2640.

2739, 1160., -40., 2620.

2740, 1160., -40., 2600.

2741, 1160., -40., 2580.

2742, 1160., -40., 2560.

2743, 1160., -40., 2540.

2744, 1160., -20., 2740.

2745, 1160., -20., 2720.

2746, 1160., -20., 2700.

2747, 1160., -20., 2680.

2748, 1160., -20., 2660.

2749, 1160., -20., 2640.

2750, 1160., -20., 2620.

2751, 1160., -20., 2600.

2752, 1160., -20., 2580.

2753, 1160., -20., 2560.

2754, 1160., -20., 2540.

2755, 2340., -100., 2740.

2756, 2340., -100., 2720.

2757, 2340., -100., 2700.

2758, 2340., -100., 2680.

2759, 2340., -100., 2660.

2760, 2340., -100., 2640.

2761, 2340., -100., 2620.

2762, 2340., -100., 2600.

2763, 2340., -100., 2580.

2764, 2340., -100., 2560.

2765, 2340., -100., 2540.

2766, 2340., -80., 2740.

2767, 2340., -80., 2720.

2768, 2340., -80., 2700.

2769, 2340., -80., 2680.

2770, 2340., -80., 2660.

2771, 2340., -80., 2640.

2772, 2340., -80., 2620.

2773, 2340., -80., 2600.

2774, 2340., -80., 2580.

2775, 2340., -80., 2560.

2776, 2340., -80., 2540.

2777, 2340., -60., 2740.

2778, 2340., -60., 2720.

2779, 2340., -60., 2700.

2780, 2340., -60., 2680.

2781, 2340., -60., 2660.

2782, 2340., -60., 2640.

2783, 2340., -60., 2620.

2784, 2340., -60., 2600.

2785, 2340., -60., 2580.

2786, 2340., -60., 2560.

2787, 2340., -60., 2540.

2788, 2340., -40., 2740.

2789, 2340., -40., 2720.

2790, 2340., -40., 2700.

2791, 2340., -40., 2680.

2792, 2340., -40., 2660.

2793, 2340., -40., 2640.

2794, 2340., -40., 2620.

2795, 2340., -40., 2600.

2796, 2340., -40., 2580.

2797, 2340., -40., 2560.

2798, 2340., -40., 2540.

2799, 2340., -20., 2740.

2800, 2340., -20., 2720.

2801, 2340., -20., 2700.

2802, 2340., -20., 2680.

2803, 2340., -20., 2660.

2804, 2340., -20., 2640.

2805, 2340., -20., 2620.

2806, 2340., -20., 2600.

2807, 2340., -20., 2580.

2808, 2340., -20., 2560.

2809, 2340., -20., 2540.

2810, 120., -120., 2540.

2811, 120., -120., 2560.

2812, 120., -120., 2580.

2813, 120., -120., 2600.

2814, 120., -120., 2620.

2815, 120., -120., 2640.

2816, 120., -120., 2660.

2817, 120., -120., 2680.

2818, 120., -120., 2700.

2819, 120., -120., 2720.

2820, 120., -120., 2740.

2821, 100., -120., 2540.

2822, 100., -120., 2560.

2823, 100., -120., 2580.

2824, 100., -120., 2600.

2825, 100., -120., 2620.

2826, 100., -120., 2640.

2827, 100., -120., 2660.

2828, 100., -120., 2680.

2829, 100., -120., 2700.

2830, 100., -120., 2720.

2831, 100., -120., 2740.

2832, 80., -120., 2540.

2833, 80., -120., 2560.

2834, 80., -120., 2580.

2835, 80., -120., 2600.

2836, 80., -120., 2620.

2837, 80., -120., 2640.

2838, 80., -120., 2660.

2839, 80., -120., 2680.

2840, 80., -120., 2700.

2841, 80., -120., 2720.

2842, 80., -120., 2740.

2843, 120., 0., 2540.

2844, 120., 0., 2560.

2845, 120., 0., 2580.

2846, 120., 0., 2600.

2847, 120., 0., 2620.

2848, 120., 0., 2640.

2849, 120., 0., 2660.

2850, 120., 0., 2680.

2851, 120., 0., 2700.

2852, 120., 0., 2720.

2853, 120., 0., 2740.

2854, 100., 0., 2540.

2855, 100., 0., 2560.

2856, 100., 0., 2580.

2857, 100., 0., 2600.

2858, 100., 0., 2620.

2859, 100., 0., 2640.

2860, 100., 0., 2660.

2861, 100., 0., 2680.

2862, 100., 0., 2700.

2863, 100., 0., 2720.

2864, 100., 0., 2740.

2865, 80., 0., 2540.

2866, 80., 0., 2560.

2867, 80., 0., 2580.

2868, 80., 0., 2600.

2869, 80., 0., 2620.

2870, 80., 0., 2640.

2871, 80., 0., 2660.

2872, 80., 0., 2680.

2873, 80., 0., 2700.

2874, 80., 0., 2720.

2875, 80., 0., 2740.

2876, 128.203644, -101.197174, 2520.

2877, 105.469093, -100.798119, 2520.

2878, 82.7345428, -100.399055, 2520.

2879, 133.281219, -80.964447, 2520.

2880, 108.854141, -80.6429672, 2520.

2881, 84.4270706, -80.3214798, 2520.

2882, 135., -60., 2520.

2883, 110., -60., 2520.

2884, 85., -60., 2520.

2885, 133.281219, -39.035553, 2520.

2886, 108.854141, -39.3570366, 2520.

2887, 84.4270706, -39.6785164, 2520.

2888, 128.203644, -18.8028278, 2520.

2889, 105.469093, -19.2018852, 2520.

2890, 82.7345428, -19.6009426, 2520.

2891, 128.203644, -101.197174, 2760.

2892, 105.469093, -100.798119, 2760.

2893, 82.7345428, -100.399055, 2760.

2894, 133.281219, -80.964447, 2760.

2895, 108.854141, -80.6429672, 2760.

2896, 84.4270706, -80.3214798, 2760.

2897, 135., -60., 2760.

2898, 110., -60., 2760.

2899, 85., -60., 2760.

2900, 133.281219, -39.035553, 2760.

2901, 108.854141, -39.3570366, 2760.

2902, 84.4270706, -39.6785164, 2760.

2903, 128.203644, -18.8028278, 2760.

2904, 105.469093, -19.2018852, 2760.

2905, 82.7345428, -19.6009426, 2760.

2906, 60., -100., 2540.

2907, 60., -100., 2560.

2908, 60., -100., 2580.

2909, 60., -100., 2600.

2910, 60., -100., 2620.

2911, 60., -100., 2640.

2912, 60., -80., 2540.

2913, 60., -80., 2560.

2914, 60., -80., 2580.

2915, 60., -80., 2600.

2916, 60., -80., 2620.

2917, 60., -80., 2640.

2918, 60., -60., 2540.

2919, 60., -60., 2560.

2920, 60., -60., 2580.

2921, 60., -60., 2600.

2922, 60., -60., 2620.

2923, 60., -60., 2640.

2924, 60., -40., 2540.

2925, 60., -40., 2560.

2926, 60., -40., 2580.

2927, 60., -40., 2600.

2928, 60., -40., 2620.

2929, 60., -40., 2640.

2930, 60., -20., 2540.

2931, 60., -20., 2560.

2932, 60., -20., 2580.

2933, 60., -20., 2600.

2934, 60., -20., 2620.

2935, 60., -20., 2640.

2936, 60., -100., 2740.

2937, 60., -80., 2740.

2938, 60., -60., 2740.

2939, 60., -40., 2740.

2940, 60., -20., 2740.

2941, 60., -100., 2720.

2942, 60., -80., 2720.

2943, 60., -60., 2720.

2944, 60., -40., 2720.

2945, 60., -20., 2720.

2946, 60., -100., 2700.

2947, 60., -80., 2700.

2948, 60., -60., 2700.

2949, 60., -40., 2700.

2950, 60., -20., 2700.

2951, 60., -100., 2680.

2952, 60., -80., 2680.

2953, 60., -60., 2680.

2954, 60., -40., 2680.

2955, 60., -20., 2680.

2956, 1131.42859, -120., 2540.

2957, 1131.42859, -120., 2560.

2958, 1131.42859, -120., 2580.

2959, 1131.42859, -120., 2600.

2960, 1131.42859, -120., 2620.

2961, 1131.42859, -120., 2640.

2962, 1131.42859, -120., 2660.

2963, 1131.42859, -120., 2680.

2964, 1131.42859, -120., 2700.

2965, 1131.42859, -120., 2720.

2966, 1131.42859, -120., 2740.

2967, 1102.85718, -120., 2540.

2968, 1102.85718, -120., 2560.

2969, 1102.85718, -120., 2580.

2970, 1102.85718, -120., 2600.

2971, 1102.85718, -120., 2620.

2972, 1102.85718, -120., 2640.

2973, 1102.85718, -120., 2660.

2974, 1102.85718, -120., 2680.

2975, 1102.85718, -120., 2700.

2976, 1102.85718, -120., 2720.

2977, 1102.85718, -120., 2740.

2978, 1074.28577, -120., 2540.

2979, 1074.28577, -120., 2560.

2980, 1074.28577, -120., 2580.

2981, 1074.28577, -120., 2600.

2982, 1074.28577, -120., 2620.

2983, 1074.28577, -120., 2640.

2984, 1074.28577, -120., 2660.

2985, 1074.28577, -120., 2680.

2986, 1074.28577, -120., 2700.

2987, 1074.28577, -120., 2720.

2988, 1074.28577, -120., 2740.

2989, 1045.71423, -120., 2540.

2990, 1045.71423, -120., 2560.

2991, 1045.71423, -120., 2580.

2992, 1045.71423, -120., 2600.

2993, 1045.71423, -120., 2620.

2994, 1045.71423, -120., 2640.

2995, 1045.71423, -120., 2660.

2996, 1045.71423, -120., 2680.

2997, 1045.71423, -120., 2700.

2998, 1045.71423, -120., 2720.

2999, 1045.71423, -120., 2740.

3000, 1017.14288, -120., 2540.

3001, 1017.14288, -120., 2560.

3002, 1017.14288, -120., 2580.

3003, 1017.14288, -120., 2600.

3004, 1017.14288, -120., 2620.

3005, 1017.14288, -120., 2640.

3006, 1017.14288, -120., 2660.

3007, 1017.14288, -120., 2680.

3008, 1017.14288, -120., 2700.

3009, 1017.14288, -120., 2720.

3010, 1017.14288, -120., 2740.

3011, 988.571411, -120., 2540.

3012, 988.571411, -120., 2560.

3013, 988.571411, -120., 2580.

3014, 988.571411, -120., 2600.

3015, 988.571411, -120., 2620.

3016, 988.571411, -120., 2640.

3017, 988.571411, -120., 2660.

3018, 988.571411, -120., 2680.

3019, 988.571411, -120., 2700.

3020, 988.571411, -120., 2720.

3021, 988.571411, -120., 2740.

3022, 1131.42859, 0., 2540.

3023, 1131.42859, 0., 2560.

3024, 1131.42859, 0., 2580.

3025, 1131.42859, 0., 2600.

3026, 1131.42859, 0., 2620.

3027, 1131.42859, 0., 2640.

3028, 1131.42859, 0., 2660.

3029, 1131.42859, 0., 2680.

3030, 1131.42859, 0., 2700.

3031, 1131.42859, 0., 2720.

3032, 1131.42859, 0., 2740.

3033, 1102.85718, 0., 2540.

3034, 1102.85718, 0., 2560.

3035, 1102.85718, 0., 2580.

3036, 1102.85718, 0., 2600.

3037, 1102.85718, 0., 2620.

3038, 1102.85718, 0., 2640.

3039, 1102.85718, 0., 2660.

3040, 1102.85718, 0., 2680.

3041, 1102.85718, 0., 2700.

3042, 1102.85718, 0., 2720.

3043, 1102.85718, 0., 2740.

3044, 1074.28577, 0., 2540.

3045, 1074.28577, 0., 2560.

3046, 1074.28577, 0., 2580.

3047, 1074.28577, 0., 2600.

3048, 1074.28577, 0., 2620.

3049, 1074.28577, 0., 2640.

3050, 1074.28577, 0., 2660.

3051, 1074.28577, 0., 2680.

3052, 1074.28577, 0., 2700.

3053, 1074.28577, 0., 2720.

3054, 1074.28577, 0., 2740.

3055, 1045.71423, 0., 2540.

3056, 1045.71423, 0., 2560.

3057, 1045.71423, 0., 2580.

3058, 1045.71423, 0., 2600.

3059, 1045.71423, 0., 2620.

3060, 1045.71423, 0., 2640.

3061, 1045.71423, 0., 2660.

3062, 1045.71423, 0., 2680.

3063, 1045.71423, 0., 2700.

3064, 1045.71423, 0., 2720.

3065, 1045.71423, 0., 2740.

3066, 1017.14288, 0., 2540.

3067, 1017.14288, 0., 2560.

3068, 1017.14288, 0., 2580.

3069, 1017.14288, 0., 2600.

3070, 1017.14288, 0., 2620.

3071, 1017.14288, 0., 2640.

3072, 1017.14288, 0., 2660.

3073, 1017.14288, 0., 2680.

3074, 1017.14288, 0., 2700.

3075, 1017.14288, 0., 2720.

3076, 1017.14288, 0., 2740.

3077, 988.571411, 0., 2540.

3078, 988.571411, 0., 2560.

3079, 988.571411, 0., 2580.

3080, 988.571411, 0., 2600.

3081, 988.571411, 0., 2620.

3082, 988.571411, 0., 2640.

3083, 988.571411, 0., 2660.

3084, 988.571411, 0., 2680.

3085, 988.571411, 0., 2700.

3086, 988.571411, 0., 2720.

3087, 988.571411, 0., 2740.

3088, 1131.42859, -100., 2520.

3089, 1102.85718, -100., 2520.

3090, 1074.28577, -100., 2520.

3091, 1045.71423, -100., 2520.

3092, 1017.14288, -100., 2520.

3093, 988.571411, -100., 2520.

3094, 1131.42859, -80., 2520.

3095, 1102.85718, -80., 2520.

3096, 1074.28577, -80., 2520.

3097, 1045.71423, -80., 2520.

3098, 1017.14288, -80., 2520.

3099, 988.571411, -80., 2520.

3100, 1131.42859, -60., 2520.

3101, 1102.85718, -60., 2520.

3102, 1074.28577, -60., 2520.

3103, 1045.71423, -60., 2520.

3104, 1017.14288, -60., 2520.

3105, 988.571411, -60., 2520.

3106, 1131.42859, -40., 2520.

3107, 1102.85718, -40., 2520.

3108, 1074.28577, -40., 2520.

3109, 1045.71423, -40., 2520.

3110, 1017.14288, -40., 2520.

3111, 988.571411, -40., 2520.

3112, 1131.42859, -20., 2520.

3113, 1102.85718, -20., 2520.

3114, 1074.28577, -20., 2520.

3115, 1045.71423, -20., 2520.

3116, 1017.14288, -20., 2520.

3117, 988.571411, -20., 2520.

3118, 1131.42859, -100., 2760.

3119, 1102.85718, -100., 2760.

3120, 1074.28577, -100., 2760.

3121, 1045.71423, -100., 2760.

3122, 1017.14288, -100., 2760.

3123, 988.571411, -100., 2760.

3124, 1131.42859, -80., 2760.

3125, 1102.85718, -80., 2760.

3126, 1074.28577, -80., 2760.

3127, 1045.71423, -80., 2760.

3128, 1017.14288, -80., 2760.

3129, 988.571411, -80., 2760.

3130, 1131.42859, -60., 2760.

3131, 1102.85718, -60., 2760.

3132, 1074.28577, -60., 2760.

3133, 1045.71423, -60., 2760.

3134, 1017.14288, -60., 2760.

3135, 988.571411, -60., 2760.

3136, 1131.42859, -40., 2760.

3137, 1102.85718, -40., 2760.

3138, 1074.28577, -40., 2760.

3139, 1045.71423, -40., 2760.

3140, 1017.14288, -40., 2760.

3141, 988.571411, -40., 2760.

3142, 1131.42859, -20., 2760.

3143, 1102.85718, -20., 2760.

3144, 1074.28577, -20., 2760.

3145, 1045.71423, -20., 2760.

3146, 1017.14288, -20., 2760.

3147, 988.571411, -20., 2760.

3148, -30.9381866, -101.59623, 2540.

3149, -30.9381866, -101.59623, 2560.

3150, -30.9381866, -101.59623, 2580.

3151, -30.9381866, -101.59623, 2600.

3152, -30.9381866, -101.59623, 2620.

3153, -30.9381866, -101.59623, 2640.

3154, -37.7082863, -81.2859268, 2540.

3155, -37.7082863, -81.2859268, 2560.

3156, -37.7082863, -81.2859268, 2580.

3157, -37.7082863, -81.2859268, 2600.

3158, -37.7082863, -81.2859268, 2620.

3159, -37.7082863, -81.2859268, 2640.

3160, -40., -60., 2540.

3161, -40., -60., 2560.

3162, -40., -60., 2580.

3163, -40., -60., 2600.

3164, -40., -60., 2620.

3165, -40., -60., 2640.

3166, -37.7082863, -38.7140732, 2540.

3167, -37.7082863, -38.7140732, 2560.

3168, -37.7082863, -38.7140732, 2580.

3169, -37.7082863, -38.7140732, 2600.

3170, -37.7082863, -38.7140732, 2620.

3171, -37.7082863, -38.7140732, 2640.

3172, -30.9381866, -18.4037704, 2540.

3173, -30.9381866, -18.4037704, 2560.

3174, -30.9381866, -18.4037704, 2580.

3175, -30.9381866, -18.4037704, 2600.

3176, -30.9381866, -18.4037704, 2620.

3177, -30.9381866, -18.4037704, 2640.

3178, -60., -100., 2540.

3179, -60., -100., 2560.

3180, -60., -100., 2580.

3181, -60., -100., 2600.

3182, -60., -100., 2620.

3183, -60., -100., 2640.

3184, -60., -80., 2540.

3185, -60., -80., 2560.

3186, -60., -80., 2580.

3187, -60., -80., 2600.

3188, -60., -80., 2620.

3189, -60., -80., 2640.

3190, -60., -60., 2540.

3191, -60., -60., 2560.

3192, -60., -60., 2580.

3193, -60., -60., 2600.

3194, -60., -60., 2620.

3195, -60., -60., 2640.

3196, -60., -40., 2540.

3197, -60., -40., 2560.

3198, -60., -40., 2580.

3199, -60., -40., 2600.

3200, -60., -40., 2620.

3201, -60., -40., 2640.

3202, -60., -20., 2540.

3203, -60., -20., 2560.

3204, -60., -20., 2580.

3205, -60., -20., 2600.

3206, -60., -20., 2620.

3207, -60., -20., 2640.

3208, 40., 0., 2640.

3209, 40., 0., 2620.

3210, 40., 0., 2600.

3211, 40., 0., 2580.

3212, 40., 0., 2560.

3213, 40., 0., 2540.

3214, 20., 0., 2640.

3215, 20., 0., 2620.

3216, 20., 0., 2600.

3217, 20., 0., 2580.

3218, 20., 0., 2560.

3219, 20., 0., 2540.

3220, 0., 0., 2640.

3221, 0., 0., 2620.

3222, 0., 0., 2600.

3223, 0., 0., 2580.

3224, 0., 0., 2560.

3225, 0., 0., 2540.

3226, 40., -120., 2640.

3227, 40., -120., 2620.

3228, 40., -120., 2600.

3229, 40., -120., 2580.

3230, 40., -120., 2560.

3231, 40., -120., 2540.

3232, 20., -120., 2640.

3233, 20., -120., 2620.

3234, 20., -120., 2600.

3235, 20., -120., 2580.

3236, 20., -120., 2560.

3237, 20., -120., 2540.

3238, 0., -120., 2640.

3239, 0., -120., 2620.

3240, 0., -120., 2600.

3241, 0., -120., 2580.

3242, 0., -120., 2560.

3243, 0., -120., 2540.

3244, 37.2654533, -19.6009426, 2660.

3245, 14.5309067, -19.2018852, 2660.

3246, -8.20363903, -18.8028278, 2660.

3247, 35.5729294, -39.6785164, 2660.

3248, 11.1458569, -39.3570366, 2660.

3249, -13.2812147, -39.035553, 2660.

3250, 35., -60., 2660.

3251, 10., -60., 2660.

3252, -15., -60., 2660.

3253, 35.5729294, -80.3214798, 2660.

3254, 11.1458569, -80.6429672, 2660.

3255, -13.2812147, -80.964447, 2660.

3256, 37.2654533, -100.399055, 2660.

3257, 14.5309067, -100.798119, 2660.

3258, -8.20363903, -101.197174, 2660.

3259, 37.2654533, -19.6009426, 2520.

3260, 14.5309067, -19.2018852, 2520.

3261, -8.20363903, -18.8028278, 2520.

3262, 35.5729294, -39.6785164, 2520.

3263, 11.1458569, -39.3570366, 2520.

3264, -13.2812147, -39.035553, 2520.

3265, 35., -60., 2520.

3266, 10., -60., 2520.

3267, -15., -60., 2520.

3268, 35.5729294, -80.3214798, 2520.

3269, 11.1458569, -80.6429672, 2520.

3270, -13.2812147, -80.964447, 2520.

3271, 37.2654533, -100.399055, 2520.

3272, 14.5309067, -100.798119, 2520.

3273, -8.20363903, -101.197174, 2520.

3274, 180.903442, -101.537109, 2740.

3275, 180.903442, -101.537109, 2720.

3276, 180.903442, -101.537109, 2700.

3277, 180.903442, -101.537109, 2680.

3278, 180.903442, -101.537109, 2660.

3279, 180.903442, -101.537109, 2640.

3280, 180.903442, -101.537109, 2620.

3281, 180.903442, -101.537109, 2600.

3282, 180.903442, -101.537109, 2580.

3283, 180.903442, -101.537109, 2560.

3284, 180.903442, -101.537109, 2540.

3285, 210.868698, -101.477989, 2740.

3286, 210.868698, -101.477989, 2720.

3287, 210.868698, -101.477989, 2700.

3288, 210.868698, -101.477989, 2680.

3289, 210.868698, -101.477989, 2660.

3290, 210.868698, -101.477989, 2640.

3291, 210.868698, -101.477989, 2620.

3292, 210.868698, -101.477989, 2600.

3293, 210.868698, -101.477989, 2580.

3294, 210.868698, -101.477989, 2560.

3295, 210.868698, -101.477989, 2540.

3296, 240.833939, -101.418869, 2740.

3297, 240.833939, -101.418869, 2720.

3298, 240.833939, -101.418869, 2700.

3299, 240.833939, -101.418869, 2680.

3300, 240.833939, -101.418869, 2660.

3301, 240.833939, -101.418869, 2640.

3302, 240.833939, -101.418869, 2620.

3303, 240.833939, -101.418869, 2600.

3304, 240.833939, -101.418869, 2580.

3305, 240.833939, -101.418869, 2560.

3306, 240.833939, -101.418869, 2540.

3307, 270.799194, -101.359749, 2740.

3308, 270.799194, -101.359749, 2720.

3309, 270.799194, -101.359749, 2700.

3310, 270.799194, -101.359749, 2680.

3311, 270.799194, -101.359749, 2660.

3312, 270.799194, -101.359749, 2640.

3313, 270.799194, -101.359749, 2620.

3314, 270.799194, -101.359749, 2600.

3315, 270.799194, -101.359749, 2580.

3316, 270.799194, -101.359749, 2560.

3317, 270.799194, -101.359749, 2540.

3318, 300.764435, -101.300629, 2740.

3319, 300.764435, -101.300629, 2720.

3320, 300.764435, -101.300629, 2700.

3321, 300.764435, -101.300629, 2680.

3322, 300.764435, -101.300629, 2660.

3323, 300.764435, -101.300629, 2640.

3324, 300.764435, -101.300629, 2620.

3325, 300.764435, -101.300629, 2600.

3326, 300.764435, -101.300629, 2580.

3327, 300.764435, -101.300629, 2560.

3328, 300.764435, -101.300629, 2540.

3329, 330.729706, -101.241508, 2740.

3330, 330.729706, -101.241508, 2720.

3331, 330.729706, -101.241508, 2700.

3332, 330.729706, -101.241508, 2680.

3333, 330.729706, -101.241508, 2660.

3334, 330.729706, -101.241508, 2640.

3335, 330.729706, -101.241508, 2620.

3336, 330.729706, -101.241508, 2600.

3337, 330.729706, -101.241508, 2580.

3338, 330.729706, -101.241508, 2560.

3339, 330.729706, -101.241508, 2540.

3340, 360.694946, -101.182396, 2740.

3341, 360.694946, -101.182396, 2720.

3342, 360.694946, -101.182396, 2700.

3343, 360.694946, -101.182396, 2680.

3344, 360.694946, -101.182396, 2660.

3345, 360.694946, -101.182396, 2640.

3346, 360.694946, -101.182396, 2620.

3347, 360.694946, -101.182396, 2600.

3348, 360.694946, -101.182396, 2580.

3349, 360.694946, -101.182396, 2560.

3350, 360.694946, -101.182396, 2540.

3351, 390.660217, -101.123276, 2740.

3352, 390.660217, -101.123276, 2720.

3353, 390.660217, -101.123276, 2700.

3354, 390.660217, -101.123276, 2680.

3355, 390.660217, -101.123276, 2660.

3356, 390.660217, -101.123276, 2640.

3357, 390.660217, -101.123276, 2620.

3358, 390.660217, -101.123276, 2600.

3359, 390.660217, -101.123276, 2580.

3360, 390.660217, -101.123276, 2560.

3361, 390.660217, -101.123276, 2540.

3362, 420.625458, -101.064156, 2740.

3363, 420.625458, -101.064156, 2720.

3364, 420.625458, -101.064156, 2700.

3365, 420.625458, -101.064156, 2680.

3366, 420.625458, -101.064156, 2660.

3367, 420.625458, -101.064156, 2640.

3368, 420.625458, -101.064156, 2620.

3369, 420.625458, -101.064156, 2600.

3370, 420.625458, -101.064156, 2580.

3371, 420.625458, -101.064156, 2560.

3372, 420.625458, -101.064156, 2540.

3373, 450.590698, -101.005035, 2740.

3374, 450.590698, -101.005035, 2720.

3375, 450.590698, -101.005035, 2700.

3376, 450.590698, -101.005035, 2680.

3377, 450.590698, -101.005035, 2660.

3378, 450.590698, -101.005035, 2640.

3379, 450.590698, -101.005035, 2620.

3380, 450.590698, -101.005035, 2600.

3381, 450.590698, -101.005035, 2580.

3382, 450.590698, -101.005035, 2560.

3383, 450.590698, -101.005035, 2540.

3384, 480.555969, -100.945915, 2740.

3385, 480.555969, -100.945915, 2720.

3386, 480.555969, -100.945915, 2700.

3387, 480.555969, -100.945915, 2680.

3388, 480.555969, -100.945915, 2660.

3389, 480.555969, -100.945915, 2640.

3390, 480.555969, -100.945915, 2620.

3391, 480.555969, -100.945915, 2600.

3392, 480.555969, -100.945915, 2580.

3393, 480.555969, -100.945915, 2560.

3394, 480.555969, -100.945915, 2540.

3395, 510.52121, -100.886795, 2740.

3396, 510.52121, -100.886795, 2720.

3397, 510.52121, -100.886795, 2700.

3398, 510.52121, -100.886795, 2680.

3399, 510.52121, -100.886795, 2660.

3400, 510.52121, -100.886795, 2640.

3401, 510.52121, -100.886795, 2620.

3402, 510.52121, -100.886795, 2600.

3403, 510.52121, -100.886795, 2580.

3404, 510.52121, -100.886795, 2560.

3405, 510.52121, -100.886795, 2540.

3406, 540.48645, -100.827675, 2740.

3407, 540.48645, -100.827675, 2720.

3408, 540.48645, -100.827675, 2700.

3409, 540.48645, -100.827675, 2680.

3410, 540.48645, -100.827675, 2660.

3411, 540.48645, -100.827675, 2640.

3412, 540.48645, -100.827675, 2620.

3413, 540.48645, -100.827675, 2600.

3414, 540.48645, -100.827675, 2580.

3415, 540.48645, -100.827675, 2560.

3416, 540.48645, -100.827675, 2540.

3417, 570.451721, -100.768555, 2740.

3418, 570.451721, -100.768555, 2720.

3419, 570.451721, -100.768555, 2700.

3420, 570.451721, -100.768555, 2680.

3421, 570.451721, -100.768555, 2660.

3422, 570.451721, -100.768555, 2640.

3423, 570.451721, -100.768555, 2620.

3424, 570.451721, -100.768555, 2600.

3425, 570.451721, -100.768555, 2580.

3426, 570.451721, -100.768555, 2560.

3427, 570.451721, -100.768555, 2540.

3428, 600.416992, -100.709435, 2740.

3429, 600.416992, -100.709435, 2720.

3430, 600.416992, -100.709435, 2700.

3431, 600.416992, -100.709435, 2680.

3432, 600.416992, -100.709435, 2660.

3433, 600.416992, -100.709435, 2640.

3434, 600.416992, -100.709435, 2620.

3435, 600.416992, -100.709435, 2600.

3436, 600.416992, -100.709435, 2580.

3437, 600.416992, -100.709435, 2560.

3438, 600.416992, -100.709435, 2540.

3439, 630.382202, -100.650314, 2740.

3440, 630.382202, -100.650314, 2720.

3441, 630.382202, -100.650314, 2700.

3442, 630.382202, -100.650314, 2680.

3443, 630.382202, -100.650314, 2660.

3444, 630.382202, -100.650314, 2640.

3445, 630.382202, -100.650314, 2620.

3446, 630.382202, -100.650314, 2600.

3447, 630.382202, -100.650314, 2580.

3448, 630.382202, -100.650314, 2560.

3449, 630.382202, -100.650314, 2540.

3450, 660.347473, -100.591194, 2740.

3451, 660.347473, -100.591194, 2720.

3452, 660.347473, -100.591194, 2700.

3453, 660.347473, -100.591194, 2680.

3454, 660.347473, -100.591194, 2660.

3455, 660.347473, -100.591194, 2640.

3456, 660.347473, -100.591194, 2620.

3457, 660.347473, -100.591194, 2600.

3458, 660.347473, -100.591194, 2580.

3459, 660.347473, -100.591194, 2560.

3460, 660.347473, -100.591194, 2540.

3461, 690.312744, -100.532074, 2740.

3462, 690.312744, -100.532074, 2720.

3463, 690.312744, -100.532074, 2700.

3464, 690.312744, -100.532074, 2680.

3465, 690.312744, -100.532074, 2660.

3466, 690.312744, -100.532074, 2640.

3467, 690.312744, -100.532074, 2620.

3468, 690.312744, -100.532074, 2600.

3469, 690.312744, -100.532074, 2580.

3470, 690.312744, -100.532074, 2560.

3471, 690.312744, -100.532074, 2540.

3472, 720.277954, -100.472954, 2740.

3473, 720.277954, -100.472954, 2720.

3474, 720.277954, -100.472954, 2700.

3475, 720.277954, -100.472954, 2680.

3476, 720.277954, -100.472954, 2660.

3477, 720.277954, -100.472954, 2640.

3478, 720.277954, -100.472954, 2620.

3479, 720.277954, -100.472954, 2600.

3480, 720.277954, -100.472954, 2580.

3481, 720.277954, -100.472954, 2560.

3482, 720.277954, -100.472954, 2540.

3483, 750.243225, -100.413834, 2740.

3484, 750.243225, -100.413834, 2720.

3485, 750.243225, -100.413834, 2700.

3486, 750.243225, -100.413834, 2680.

3487, 750.243225, -100.413834, 2660.

3488, 750.243225, -100.413834, 2640.

3489, 750.243225, -100.413834, 2620.

3490, 750.243225, -100.413834, 2600.

3491, 750.243225, -100.413834, 2580.

3492, 750.243225, -100.413834, 2560.

3493, 750.243225, -100.413834, 2540.

3494, 780.208496, -100.354721, 2740.

3495, 780.208496, -100.354721, 2720.

3496, 780.208496, -100.354721, 2700.

3497, 780.208496, -100.354721, 2680.

3498, 780.208496, -100.354721, 2660.

3499, 780.208496, -100.354721, 2640.

3500, 780.208496, -100.354721, 2620.

3501, 780.208496, -100.354721, 2600.

3502, 780.208496, -100.354721, 2580.

3503, 780.208496, -100.354721, 2560.

3504, 780.208496, -100.354721, 2540.

3505, 810.173767, -100.295601, 2740.

3506, 810.173767, -100.295601, 2720.

3507, 810.173767, -100.295601, 2700.

3508, 810.173767, -100.295601, 2680.

3509, 810.173767, -100.295601, 2660.

3510, 810.173767, -100.295601, 2640.

3511, 810.173767, -100.295601, 2620.

3512, 810.173767, -100.295601, 2600.

3513, 810.173767, -100.295601, 2580.

3514, 810.173767, -100.295601, 2560.

3515, 810.173767, -100.295601, 2540.

3516, 840.138977, -100.236481, 2740.

3517, 840.138977, -100.236481, 2720.

3518, 840.138977, -100.236481, 2700.

3519, 840.138977, -100.236481, 2680.

3520, 840.138977, -100.236481, 2660.

3521, 840.138977, -100.236481, 2640.

3522, 840.138977, -100.236481, 2620.

3523, 840.138977, -100.236481, 2600.

3524, 840.138977, -100.236481, 2580.

3525, 840.138977, -100.236481, 2560.

3526, 840.138977, -100.236481, 2540.

3527, 870.104248, -100.177361, 2740.

3528, 870.104248, -100.177361, 2720.

3529, 870.104248, -100.177361, 2700.

3530, 870.104248, -100.177361, 2680.

3531, 870.104248, -100.177361, 2660.

3532, 870.104248, -100.177361, 2640.

3533, 870.104248, -100.177361, 2620.

3534, 870.104248, -100.177361, 2600.

3535, 870.104248, -100.177361, 2580.

3536, 870.104248, -100.177361, 2560.

3537, 870.104248, -100.177361, 2540.

3538, 900.069519, -100.11824, 2740.

3539, 900.069519, -100.11824, 2720.

3540, 900.069519, -100.11824, 2700.

3541, 900.069519, -100.11824, 2680.

3542, 900.069519, -100.11824, 2660.

3543, 900.069519, -100.11824, 2640.

3544, 900.069519, -100.11824, 2620.

3545, 900.069519, -100.11824, 2600.

3546, 900.069519, -100.11824, 2580.

3547, 900.069519, -100.11824, 2560.

3548, 900.069519, -100.11824, 2540.

3549, 930.034729, -100.05912, 2740.

3550, 930.034729, -100.05912, 2720.

3551, 930.034729, -100.05912, 2700.

3552, 930.034729, -100.05912, 2680.

3553, 930.034729, -100.05912, 2660.

3554, 930.034729, -100.05912, 2640.

3555, 930.034729, -100.05912, 2620.

3556, 930.034729, -100.05912, 2600.

3557, 930.034729, -100.05912, 2580.

3558, 930.034729, -100.05912, 2560.

3559, 930.034729, -100.05912, 2540.

3560, 187.422791, -81.2383041, 2740.

3561, 187.422791, -81.2383041, 2720.

3562, 187.422791, -81.2383041, 2700.

3563, 187.422791, -81.2383041, 2680.

3564, 187.422791, -81.2383041, 2660.

3565, 187.422791, -81.2383041, 2640.

3566, 187.422791, -81.2383041, 2620.

3567, 187.422791, -81.2383041, 2600.

3568, 187.422791, -81.2383041, 2580.

3569, 187.422791, -81.2383041, 2560.

3570, 187.422791, -81.2383041, 2540.

3571, 217.137299, -81.1906738, 2740.

3572, 217.137299, -81.1906738, 2720.

3573, 217.137299, -81.1906738, 2700.

3574, 217.137299, -81.1906738, 2680.

3575, 217.137299, -81.1906738, 2660.

3576, 217.137299, -81.1906738, 2640.

3577, 217.137299, -81.1906738, 2620.

3578, 217.137299, -81.1906738, 2600.

3579, 217.137299, -81.1906738, 2580.

3580, 217.137299, -81.1906738, 2560.

3581, 217.137299, -81.1906738, 2540.

3582, 246.851807, -81.1430435, 2740.

3583, 246.851807, -81.1430435, 2720.

3584, 246.851807, -81.1430435, 2700.

3585, 246.851807, -81.1430435, 2680.

3586, 246.851807, -81.1430435, 2660.

3587, 246.851807, -81.1430435, 2640.

3588, 246.851807, -81.1430435, 2620.

3589, 246.851807, -81.1430435, 2600.

3590, 246.851807, -81.1430435, 2580.

3591, 246.851807, -81.1430435, 2560.

3592, 246.851807, -81.1430435, 2540.

3593, 276.566315, -81.0954208, 2740.

3594, 276.566315, -81.0954208, 2720.

3595, 276.566315, -81.0954208, 2700.

3596, 276.566315, -81.0954208, 2680.

3597, 276.566315, -81.0954208, 2660.

3598, 276.566315, -81.0954208, 2640.

3599, 276.566315, -81.0954208, 2620.

3600, 276.566315, -81.0954208, 2600.

3601, 276.566315, -81.0954208, 2580.

3602, 276.566315, -81.0954208, 2560.

3603, 276.566315, -81.0954208, 2540.

3604, 306.280823, -81.0477905, 2740.

3605, 306.280823, -81.0477905, 2720.

3606, 306.280823, -81.0477905, 2700.

3607, 306.280823, -81.0477905, 2680.

3608, 306.280823, -81.0477905, 2660.

3609, 306.280823, -81.0477905, 2640.

3610, 306.280823, -81.0477905, 2620.

3611, 306.280823, -81.0477905, 2600.

3612, 306.280823, -81.0477905, 2580.

3613, 306.280823, -81.0477905, 2560.

3614, 306.280823, -81.0477905, 2540.

3615, 335.995331, -81.0001678, 2740.

3616, 335.995331, -81.0001678, 2720.

3617, 335.995331, -81.0001678, 2700.

3618, 335.995331, -81.0001678, 2680.

3619, 335.995331, -81.0001678, 2660.

3620, 335.995331, -81.0001678, 2640.

3621, 335.995331, -81.0001678, 2620.

3622, 335.995331, -81.0001678, 2600.

3623, 335.995331, -81.0001678, 2580.

3624, 335.995331, -81.0001678, 2560.

3625, 335.995331, -81.0001678, 2540.

3626, 365.709839, -80.9525375, 2740.

3627, 365.709839, -80.9525375, 2720.

3628, 365.709839, -80.9525375, 2700.

3629, 365.709839, -80.9525375, 2680.

3630, 365.709839, -80.9525375, 2660.

3631, 365.709839, -80.9525375, 2640.

3632, 365.709839, -80.9525375, 2620.

3633, 365.709839, -80.9525375, 2600.

3634, 365.709839, -80.9525375, 2580.

3635, 365.709839, -80.9525375, 2560.

3636, 365.709839, -80.9525375, 2540.

3637, 395.424347, -80.9049149, 2740.

3638, 395.424347, -80.9049149, 2720.

3639, 395.424347, -80.9049149, 2700.

3640, 395.424347, -80.9049149, 2680.

3641, 395.424347, -80.9049149, 2660.

3642, 395.424347, -80.9049149, 2640.

3643, 395.424347, -80.9049149, 2620.

3644, 395.424347, -80.9049149, 2600.

3645, 395.424347, -80.9049149, 2580.

3646, 395.424347, -80.9049149, 2560.

3647, 395.424347, -80.9049149, 2540.

3648, 425.138855, -80.8572845, 2740.

3649, 425.138855, -80.8572845, 2720.

3650, 425.138855, -80.8572845, 2700.

3651, 425.138855, -80.8572845, 2680.

3652, 425.138855, -80.8572845, 2660.

3653, 425.138855, -80.8572845, 2640.

3654, 425.138855, -80.8572845, 2620.

3655, 425.138855, -80.8572845, 2600.

3656, 425.138855, -80.8572845, 2580.

3657, 425.138855, -80.8572845, 2560.

3658, 425.138855, -80.8572845, 2540.

3659, 454.853363, -80.8096542, 2740.

3660, 454.853363, -80.8096542, 2720.

3661, 454.853363, -80.8096542, 2700.

3662, 454.853363, -80.8096542, 2680.

3663, 454.853363, -80.8096542, 2660.

3664, 454.853363, -80.8096542, 2640.

3665, 454.853363, -80.8096542, 2620.

3666, 454.853363, -80.8096542, 2600.

3667, 454.853363, -80.8096542, 2580.

3668, 454.853363, -80.8096542, 2560.

3669, 454.853363, -80.8096542, 2540.

3670, 484.567871, -80.7620316, 2740.

3671, 484.567871, -80.7620316, 2720.

3672, 484.567871, -80.7620316, 2700.

3673, 484.567871, -80.7620316, 2680.

3674, 484.567871, -80.7620316, 2660.

3675, 484.567871, -80.7620316, 2640.

3676, 484.567871, -80.7620316, 2620.

3677, 484.567871, -80.7620316, 2600.

3678, 484.567871, -80.7620316, 2580.

3679, 484.567871, -80.7620316, 2560.

3680, 484.567871, -80.7620316, 2540.

3681, 514.28241, -80.7144012, 2740.

3682, 514.28241, -80.7144012, 2720.

3683, 514.28241, -80.7144012, 2700.

3684, 514.28241, -80.7144012, 2680.

3685, 514.28241, -80.7144012, 2660.

3686, 514.28241, -80.7144012, 2640.

3687, 514.28241, -80.7144012, 2620.

3688, 514.28241, -80.7144012, 2600.

3689, 514.28241, -80.7144012, 2580.

3690, 514.28241, -80.7144012, 2560.

3691, 514.28241, -80.7144012, 2540.

3692, 543.996887, -80.6667786, 2740.

3693, 543.996887, -80.6667786, 2720.

3694, 543.996887, -80.6667786, 2700.

3695, 543.996887, -80.6667786, 2680.

3696, 543.996887, -80.6667786, 2660.

3697, 543.996887, -80.6667786, 2640.

3698, 543.996887, -80.6667786, 2620.

3699, 543.996887, -80.6667786, 2600.

3700, 543.996887, -80.6667786, 2580.

3701, 543.996887, -80.6667786, 2560.

3702, 543.996887, -80.6667786, 2540.

3703, 573.711426, -80.6191483, 2740.

3704, 573.711426, -80.6191483, 2720.

3705, 573.711426, -80.6191483, 2700.

3706, 573.711426, -80.6191483, 2680.

3707, 573.711426, -80.6191483, 2660.

3708, 573.711426, -80.6191483, 2640.

3709, 573.711426, -80.6191483, 2620.

3710, 573.711426, -80.6191483, 2600.

3711, 573.711426, -80.6191483, 2580.

3712, 573.711426, -80.6191483, 2560.

3713, 573.711426, -80.6191483, 2540.

3714, 603.425903, -80.5715256, 2740.

3715, 603.425903, -80.5715256, 2720.

3716, 603.425903, -80.5715256, 2700.

3717, 603.425903, -80.5715256, 2680.

3718, 603.425903, -80.5715256, 2660.

3719, 603.425903, -80.5715256, 2640.

3720, 603.425903, -80.5715256, 2620.

3721, 603.425903, -80.5715256, 2600.

3722, 603.425903, -80.5715256, 2580.

3723, 603.425903, -80.5715256, 2560.

3724, 603.425903, -80.5715256, 2540.

3725, 633.140442, -80.5238953, 2740.

3726, 633.140442, -80.5238953, 2720.

3727, 633.140442, -80.5238953, 2700.

3728, 633.140442, -80.5238953, 2680.

3729, 633.140442, -80.5238953, 2660.

3730, 633.140442, -80.5238953, 2640.

3731, 633.140442, -80.5238953, 2620.

3732, 633.140442, -80.5238953, 2600.

3733, 633.140442, -80.5238953, 2580.

3734, 633.140442, -80.5238953, 2560.

3735, 633.140442, -80.5238953, 2540.

3736, 662.854919, -80.4762726, 2740.

3737, 662.854919, -80.4762726, 2720.

3738, 662.854919, -80.4762726, 2700.

3739, 662.854919, -80.4762726, 2680.

3740, 662.854919, -80.4762726, 2660.

3741, 662.854919, -80.4762726, 2640.

3742, 662.854919, -80.4762726, 2620.

3743, 662.854919, -80.4762726, 2600.

3744, 662.854919, -80.4762726, 2580.

3745, 662.854919, -80.4762726, 2560.

3746, 662.854919, -80.4762726, 2540.

3747, 692.569458, -80.4286423, 2740.

3748, 692.569458, -80.4286423, 2720.

3749, 692.569458, -80.4286423, 2700.

3750, 692.569458, -80.4286423, 2680.

3751, 692.569458, -80.4286423, 2660.

3752, 692.569458, -80.4286423, 2640.

3753, 692.569458, -80.4286423, 2620.

3754, 692.569458, -80.4286423, 2600.

3755, 692.569458, -80.4286423, 2580.

3756, 692.569458, -80.4286423, 2560.

3757, 692.569458, -80.4286423, 2540.

3758, 722.283936, -80.381012, 2740.

3759, 722.283936, -80.381012, 2720.

3760, 722.283936, -80.381012, 2700.

3761, 722.283936, -80.381012, 2680.

3762, 722.283936, -80.381012, 2660.

3763, 722.283936, -80.381012, 2640.

3764, 722.283936, -80.381012, 2620.

3765, 722.283936, -80.381012, 2600.

3766, 722.283936, -80.381012, 2580.

3767, 722.283936, -80.381012, 2560.

3768, 722.283936, -80.381012, 2540.

3769, 751.998474, -80.3333893, 2740.

3770, 751.998474, -80.3333893, 2720.

3771, 751.998474, -80.3333893, 2700.

3772, 751.998474, -80.3333893, 2680.

3773, 751.998474, -80.3333893, 2660.

3774, 751.998474, -80.3333893, 2640.

3775, 751.998474, -80.3333893, 2620.

3776, 751.998474, -80.3333893, 2600.

3777, 751.998474, -80.3333893, 2580.

3778, 751.998474, -80.3333893, 2560.

3779, 751.998474, -80.3333893, 2540.

3780, 781.712952, -80.285759, 2740.

3781, 781.712952, -80.285759, 2720.

3782, 781.712952, -80.285759, 2700.

3783, 781.712952, -80.285759, 2680.

3784, 781.712952, -80.285759, 2660.

3785, 781.712952, -80.285759, 2640.

3786, 781.712952, -80.285759, 2620.

3787, 781.712952, -80.285759, 2600.

3788, 781.712952, -80.285759, 2580.

3789, 781.712952, -80.285759, 2560.

3790, 781.712952, -80.285759, 2540.

3791, 811.42749, -80.2381363, 2740.

3792, 811.42749, -80.2381363, 2720.

3793, 811.42749, -80.2381363, 2700.

3794, 811.42749, -80.2381363, 2680.

3795, 811.42749, -80.2381363, 2660.

3796, 811.42749, -80.2381363, 2640.

3797, 811.42749, -80.2381363, 2620.

3798, 811.42749, -80.2381363, 2600.

3799, 811.42749, -80.2381363, 2580.

3800, 811.42749, -80.2381363, 2560.

3801, 811.42749, -80.2381363, 2540.

3802, 841.141968, -80.190506, 2740.

3803, 841.141968, -80.190506, 2720.

3804, 841.141968, -80.190506, 2700.

3805, 841.141968, -80.190506, 2680.

3806, 841.141968, -80.190506, 2660.

3807, 841.141968, -80.190506, 2640.

3808, 841.141968, -80.190506, 2620.

3809, 841.141968, -80.190506, 2600.

3810, 841.141968, -80.190506, 2580.

3811, 841.141968, -80.190506, 2560.

3812, 841.141968, -80.190506, 2540.

3813, 870.856506, -80.1428833, 2740.

3814, 870.856506, -80.1428833, 2720.

3815, 870.856506, -80.1428833, 2700.

3816, 870.856506, -80.1428833, 2680.

3817, 870.856506, -80.1428833, 2660.

3818, 870.856506, -80.1428833, 2640.

3819, 870.856506, -80.1428833, 2620.

3820, 870.856506, -80.1428833, 2600.

3821, 870.856506, -80.1428833, 2580.

3822, 870.856506, -80.1428833, 2560.

3823, 870.856506, -80.1428833, 2540.

3824, 900.570984, -80.095253, 2740.

3825, 900.570984, -80.095253, 2720.

3826, 900.570984, -80.095253, 2700.

3827, 900.570984, -80.095253, 2680.

3828, 900.570984, -80.095253, 2660.

3829, 900.570984, -80.095253, 2640.

3830, 900.570984, -80.095253, 2620.

3831, 900.570984, -80.095253, 2600.

3832, 900.570984, -80.095253, 2580.

3833, 900.570984, -80.095253, 2560.

3834, 900.570984, -80.095253, 2540.

3835, 930.285522, -80.0476303, 2740.

3836, 930.285522, -80.0476303, 2720.

3837, 930.285522, -80.0476303, 2700.

3838, 930.285522, -80.0476303, 2680.

3839, 930.285522, -80.0476303, 2660.

3840, 930.285522, -80.0476303, 2640.

3841, 930.285522, -80.0476303, 2620.

3842, 930.285522, -80.0476303, 2600.

3843, 930.285522, -80.0476303, 2580.

3844, 930.285522, -80.0476303, 2560.

3845, 930.285522, -80.0476303, 2540.

3846, 189.629623, -60., 2740.

3847, 189.629623, -60., 2720.

3848, 189.629623, -60., 2700.

3849, 189.629623, -60., 2680.

3850, 189.629623, -60., 2660.

3851, 189.629623, -60., 2640.

3852, 189.629623, -60., 2620.

3853, 189.629623, -60., 2600.

3854, 189.629623, -60., 2580.

3855, 189.629623, -60., 2560.

3856, 189.629623, -60., 2540.

3857, 219.259262, -60., 2740.

3858, 219.259262, -60., 2720.

3859, 219.259262, -60., 2700.

3860, 219.259262, -60., 2680.

3861, 219.259262, -60., 2660.

3862, 219.259262, -60., 2640.

3863, 219.259262, -60., 2620.

3864, 219.259262, -60., 2600.

3865, 219.259262, -60., 2580.

3866, 219.259262, -60., 2560.

3867, 219.259262, -60., 2540.

3868, 248.888885, -60., 2740.

3869, 248.888885, -60., 2720.

3870, 248.888885, -60., 2700.

3871, 248.888885, -60., 2680.

3872, 248.888885, -60., 2660.

3873, 248.888885, -60., 2640.

3874, 248.888885, -60., 2620.

3875, 248.888885, -60., 2600.

3876, 248.888885, -60., 2580.

3877, 248.888885, -60., 2560.

3878, 248.888885, -60., 2540.

3879, 278.518524, -60., 2740.

3880, 278.518524, -60., 2720.

3881, 278.518524, -60., 2700.

3882, 278.518524, -60., 2680.

3883, 278.518524, -60., 2660.

3884, 278.518524, -60., 2640.

3885, 278.518524, -60., 2620.

3886, 278.518524, -60., 2600.

3887, 278.518524, -60., 2580.

3888, 278.518524, -60., 2560.

3889, 278.518524, -60., 2540.

3890, 308.148163, -60., 2740.

3891, 308.148163, -60., 2720.

3892, 308.148163, -60., 2700.

3893, 308.148163, -60., 2680.

3894, 308.148163, -60., 2660.

3895, 308.148163, -60., 2640.

3896, 308.148163, -60., 2620.

3897, 308.148163, -60., 2600.

3898, 308.148163, -60., 2580.

3899, 308.148163, -60., 2560.

3900, 308.148163, -60., 2540.

3901, 337.777771, -60., 2740.

3902, 337.777771, -60., 2720.

3903, 337.777771, -60., 2700.

3904, 337.777771, -60., 2680.

3905, 337.777771, -60., 2660.

3906, 337.777771, -60., 2640.

3907, 337.777771, -60., 2620.

3908, 337.777771, -60., 2600.

3909, 337.777771, -60., 2580.

3910, 337.777771, -60., 2560.

3911, 337.777771, -60., 2540.

3912, 367.40741, -60., 2740.

3913, 367.40741, -60., 2720.

3914, 367.40741, -60., 2700.

3915, 367.40741, -60., 2680.

3916, 367.40741, -60., 2660.

3917, 367.40741, -60., 2640.

3918, 367.40741, -60., 2620.

3919, 367.40741, -60., 2600.

3920, 367.40741, -60., 2580.

3921, 367.40741, -60., 2560.

3922, 367.40741, -60., 2540.

3923, 397.037048, -60., 2740.

3924, 397.037048, -60., 2720.

3925, 397.037048, -60., 2700.

3926, 397.037048, -60., 2680.

3927, 397.037048, -60., 2660.

3928, 397.037048, -60., 2640.

3929, 397.037048, -60., 2620.

3930, 397.037048, -60., 2600.

3931, 397.037048, -60., 2580.

3932, 397.037048, -60., 2560.

3933, 397.037048, -60., 2540.

3934, 426.666656, -60., 2740.

3935, 426.666656, -60., 2720.

3936, 426.666656, -60., 2700.

3937, 426.666656, -60., 2680.

3938, 426.666656, -60., 2660.

3939, 426.666656, -60., 2640.

3940, 426.666656, -60., 2620.

3941, 426.666656, -60., 2600.

3942, 426.666656, -60., 2580.

3943, 426.666656, -60., 2560.

3944, 426.666656, -60., 2540.

3945, 456.296295, -60., 2740.

3946, 456.296295, -60., 2720.

3947, 456.296295, -60., 2700.

3948, 456.296295, -60., 2680.

3949, 456.296295, -60., 2660.

3950, 456.296295, -60., 2640.

3951, 456.296295, -60., 2620.

3952, 456.296295, -60., 2600.

3953, 456.296295, -60., 2580.

3954, 456.296295, -60., 2560.

3955, 456.296295, -60., 2540.

3956, 485.925934, -60., 2740.

3957, 485.925934, -60., 2720.

3958, 485.925934, -60., 2700.

3959, 485.925934, -60., 2680.

3960, 485.925934, -60., 2660.

3961, 485.925934, -60., 2640.

3962, 485.925934, -60., 2620.

3963, 485.925934, -60., 2600.

3964, 485.925934, -60., 2580.

3965, 485.925934, -60., 2560.

3966, 485.925934, -60., 2540.

3967, 515.555542, -60., 2740.

3968, 515.555542, -60., 2720.

3969, 515.555542, -60., 2700.

3970, 515.555542, -60., 2680.

3971, 515.555542, -60., 2660.

3972, 515.555542, -60., 2640.

3973, 515.555542, -60., 2620.

3974, 515.555542, -60., 2600.

3975, 515.555542, -60., 2580.

3976, 515.555542, -60., 2560.

3977, 515.555542, -60., 2540.

3978, 545.185181, -60., 2740.

3979, 545.185181, -60., 2720.

3980, 545.185181, -60., 2700.

3981, 545.185181, -60., 2680.

3982, 545.185181, -60., 2660.

3983, 545.185181, -60., 2640.

3984, 545.185181, -60., 2620.

3985, 545.185181, -60., 2600.

3986, 545.185181, -60., 2580.

3987, 545.185181, -60., 2560.

3988, 545.185181, -60., 2540.

3989, 574.814819, -60., 2740.

3990, 574.814819, -60., 2720.

3991, 574.814819, -60., 2700.

3992, 574.814819, -60., 2680.

3993, 574.814819, -60., 2660.

3994, 574.814819, -60., 2640.

3995, 574.814819, -60., 2620.

3996, 574.814819, -60., 2600.

3997, 574.814819, -60., 2580.

3998, 574.814819, -60., 2560.

3999, 574.814819, -60., 2540.

4000, 604.444458, -60., 2740.

4001, 604.444458, -60., 2720.

4002, 604.444458, -60., 2700.

4003, 604.444458, -60., 2680.

4004, 604.444458, -60., 2660.

4005, 604.444458, -60., 2640.

4006, 604.444458, -60., 2620.

4007, 604.444458, -60., 2600.

4008, 604.444458, -60., 2580.

4009, 604.444458, -60., 2560.

4010, 604.444458, -60., 2540.

4011, 634.074097, -60., 2740.

4012, 634.074097, -60., 2720.

4013, 634.074097, -60., 2700.

4014, 634.074097, -60., 2680.

4015, 634.074097, -60., 2660.

4016, 634.074097, -60., 2640.

4017, 634.074097, -60., 2620.

4018, 634.074097, -60., 2600.

4019, 634.074097, -60., 2580.

4020, 634.074097, -60., 2560.

4021, 634.074097, -60., 2540.

4022, 663.703674, -60., 2740.

4023, 663.703674, -60., 2720.

4024, 663.703674, -60., 2700.

4025, 663.703674, -60., 2680.

4026, 663.703674, -60., 2660.

4027, 663.703674, -60., 2640.

4028, 663.703674, -60., 2620.

4029, 663.703674, -60., 2600.

4030, 663.703674, -60., 2580.

4031, 663.703674, -60., 2560.

4032, 663.703674, -60., 2540.

4033, 693.333313, -60., 2740.

4034, 693.333313, -60., 2720.

4035, 693.333313, -60., 2700.

4036, 693.333313, -60., 2680.

4037, 693.333313, -60., 2660.

4038, 693.333313, -60., 2640.

4039, 693.333313, -60., 2620.

4040, 693.333313, -60., 2600.

4041, 693.333313, -60., 2580.

4042, 693.333313, -60., 2560.

4043, 693.333313, -60., 2540.

4044, 722.962952, -60., 2740.

4045, 722.962952, -60., 2720.

4046, 722.962952, -60., 2700.

4047, 722.962952, -60., 2680.

4048, 722.962952, -60., 2660.

4049, 722.962952, -60., 2640.

4050, 722.962952, -60., 2620.

4051, 722.962952, -60., 2600.

4052, 722.962952, -60., 2580.

4053, 722.962952, -60., 2560.

4054, 722.962952, -60., 2540.

4055, 752.59259, -60., 2740.

4056, 752.59259, -60., 2720.

4057, 752.59259, -60., 2700.

4058, 752.59259, -60., 2680.

4059, 752.59259, -60., 2660.

4060, 752.59259, -60., 2640.

4061, 752.59259, -60., 2620.

4062, 752.59259, -60., 2600.

4063, 752.59259, -60., 2580.

4064, 752.59259, -60., 2560.

4065, 752.59259, -60., 2540.

4066, 782.222229, -60., 2740.

4067, 782.222229, -60., 2720.

4068, 782.222229, -60., 2700.

4069, 782.222229, -60., 2680.

4070, 782.222229, -60., 2660.

4071, 782.222229, -60., 2640.

4072, 782.222229, -60., 2620.

4073, 782.222229, -60., 2600.

4074, 782.222229, -60., 2580.

4075, 782.222229, -60., 2560.

4076, 782.222229, -60., 2540.

4077, 811.851868, -60., 2740.

4078, 811.851868, -60., 2720.

4079, 811.851868, -60., 2700.

4080, 811.851868, -60., 2680.

4081, 811.851868, -60., 2660.

4082, 811.851868, -60., 2640.

4083, 811.851868, -60., 2620.

4084, 811.851868, -60., 2600.

4085, 811.851868, -60., 2580.

4086, 811.851868, -60., 2560.

4087, 811.851868, -60., 2540.

4088, 841.481506, -60., 2740.

4089, 841.481506, -60., 2720.

4090, 841.481506, -60., 2700.

4091, 841.481506, -60., 2680.

4092, 841.481506, -60., 2660.

4093, 841.481506, -60., 2640.

4094, 841.481506, -60., 2620.

4095, 841.481506, -60., 2600.

4096, 841.481506, -60., 2580.

4097, 841.481506, -60., 2560.

4098, 841.481506, -60., 2540.

4099, 871.111084, -60., 2740.

4100, 871.111084, -60., 2720.

4101, 871.111084, -60., 2700.

4102, 871.111084, -60., 2680.

4103, 871.111084, -60., 2660.

4104, 871.111084, -60., 2640.

4105, 871.111084, -60., 2620.

4106, 871.111084, -60., 2600.

4107, 871.111084, -60., 2580.

4108, 871.111084, -60., 2560.

4109, 871.111084, -60., 2540.

4110, 900.740723, -60., 2740.

4111, 900.740723, -60., 2720.

4112, 900.740723, -60., 2700.

4113, 900.740723, -60., 2680.

4114, 900.740723, -60., 2660.

4115, 900.740723, -60., 2640.

4116, 900.740723, -60., 2620.

4117, 900.740723, -60., 2600.

4118, 900.740723, -60., 2580.

4119, 900.740723, -60., 2560.

4120, 900.740723, -60., 2540.

4121, 930.370361, -60., 2740.

4122, 930.370361, -60., 2720.

4123, 930.370361, -60., 2700.

4124, 930.370361, -60., 2680.

4125, 930.370361, -60., 2660.

4126, 930.370361, -60., 2640.

4127, 930.370361, -60., 2620.

4128, 930.370361, -60., 2600.

4129, 930.370361, -60., 2580.

4130, 930.370361, -60., 2560.

4131, 930.370361, -60., 2540.

4132, 187.422791, -38.7616997, 2740.

4133, 187.422791, -38.7616997, 2720.

4134, 187.422791, -38.7616997, 2700.

4135, 187.422791, -38.7616997, 2680.

4136, 187.422791, -38.7616997, 2660.

4137, 187.422791, -38.7616997, 2640.

4138, 187.422791, -38.7616997, 2620.

4139, 187.422791, -38.7616997, 2600.

4140, 187.422791, -38.7616997, 2580.

4141, 187.422791, -38.7616997, 2560.

4142, 187.422791, -38.7616997, 2540.

4143, 217.137299, -38.8093262, 2740.

4144, 217.137299, -38.8093262, 2720.

4145, 217.137299, -38.8093262, 2700.

4146, 217.137299, -38.8093262, 2680.

4147, 217.137299, -38.8093262, 2660.

4148, 217.137299, -38.8093262, 2640.

4149, 217.137299, -38.8093262, 2620.

4150, 217.137299, -38.8093262, 2600.

4151, 217.137299, -38.8093262, 2580.

4152, 217.137299, -38.8093262, 2560.

4153, 217.137299, -38.8093262, 2540.

4154, 246.851807, -38.8569527, 2740.

4155, 246.851807, -38.8569527, 2720.

4156, 246.851807, -38.8569527, 2700.

4157, 246.851807, -38.8569527, 2680.

4158, 246.851807, -38.8569527, 2660.

4159, 246.851807, -38.8569527, 2640.

4160, 246.851807, -38.8569527, 2620.

4161, 246.851807, -38.8569527, 2600.

4162, 246.851807, -38.8569527, 2580.

4163, 246.851807, -38.8569527, 2560.

4164, 246.851807, -38.8569527, 2540.

4165, 276.566315, -38.9045792, 2740.

4166, 276.566315, -38.9045792, 2720.

4167, 276.566315, -38.9045792, 2700.

4168, 276.566315, -38.9045792, 2680.

4169, 276.566315, -38.9045792, 2660.

4170, 276.566315, -38.9045792, 2640.

4171, 276.566315, -38.9045792, 2620.

4172, 276.566315, -38.9045792, 2600.

4173, 276.566315, -38.9045792, 2580.

4174, 276.566315, -38.9045792, 2560.

4175, 276.566315, -38.9045792, 2540.

4176, 306.280823, -38.9522057, 2740.

4177, 306.280823, -38.9522057, 2720.

4178, 306.280823, -38.9522057, 2700.

4179, 306.280823, -38.9522057, 2680.

4180, 306.280823, -38.9522057, 2660.

4181, 306.280823, -38.9522057, 2640.

4182, 306.280823, -38.9522057, 2620.

4183, 306.280823, -38.9522057, 2600.

4184, 306.280823, -38.9522057, 2580.

4185, 306.280823, -38.9522057, 2560.

4186, 306.280823, -38.9522057, 2540.

4187, 335.995331, -38.999836, 2740.

4188, 335.995331, -38.999836, 2720.

4189, 335.995331, -38.999836, 2700.

4190, 335.995331, -38.999836, 2680.

4191, 335.995331, -38.999836, 2660.

4192, 335.995331, -38.999836, 2640.

4193, 335.995331, -38.999836, 2620.

4194, 335.995331, -38.999836, 2600.

4195, 335.995331, -38.999836, 2580.

4196, 335.995331, -38.999836, 2560.

4197, 335.995331, -38.999836, 2540.

4198, 365.709839, -39.0474625, 2740.

4199, 365.709839, -39.0474625, 2720.

4200, 365.709839, -39.0474625, 2700.

4201, 365.709839, -39.0474625, 2680.

4202, 365.709839, -39.0474625, 2660.

4203, 365.709839, -39.0474625, 2640.

4204, 365.709839, -39.0474625, 2620.

4205, 365.709839, -39.0474625, 2600.

4206, 365.709839, -39.0474625, 2580.

4207, 365.709839, -39.0474625, 2560.

4208, 365.709839, -39.0474625, 2540.

4209, 395.424347, -39.095089, 2740.

4210, 395.424347, -39.095089, 2720.

4211, 395.424347, -39.095089, 2700.

4212, 395.424347, -39.095089, 2680.

4213, 395.424347, -39.095089, 2660.

4214, 395.424347, -39.095089, 2640.

4215, 395.424347, -39.095089, 2620.

4216, 395.424347, -39.095089, 2600.

4217, 395.424347, -39.095089, 2580.

4218, 395.424347, -39.095089, 2560.

4219, 395.424347, -39.095089, 2540.

4220, 425.138855, -39.1427155, 2740.

4221, 425.138855, -39.1427155, 2720.

4222, 425.138855, -39.1427155, 2700.

4223, 425.138855, -39.1427155, 2680.

4224, 425.138855, -39.1427155, 2660.

4225, 425.138855, -39.1427155, 2640.

4226, 425.138855, -39.1427155, 2620.

4227, 425.138855, -39.1427155, 2600.

4228, 425.138855, -39.1427155, 2580.

4229, 425.138855, -39.1427155, 2560.

4230, 425.138855, -39.1427155, 2540.

4231, 454.853363, -39.1903419, 2740.

4232, 454.853363, -39.1903419, 2720.

4233, 454.853363, -39.1903419, 2700.

4234, 454.853363, -39.1903419, 2680.

4235, 454.853363, -39.1903419, 2660.

4236, 454.853363, -39.1903419, 2640.

4237, 454.853363, -39.1903419, 2620.

4238, 454.853363, -39.1903419, 2600.

4239, 454.853363, -39.1903419, 2580.

4240, 454.853363, -39.1903419, 2560.

4241, 454.853363, -39.1903419, 2540.

4242, 484.567871, -39.2379684, 2740.

4243, 484.567871, -39.2379684, 2720.

4244, 484.567871, -39.2379684, 2700.

4245, 484.567871, -39.2379684, 2680.

4246, 484.567871, -39.2379684, 2660.

4247, 484.567871, -39.2379684, 2640.

4248, 484.567871, -39.2379684, 2620.

4249, 484.567871, -39.2379684, 2600.

4250, 484.567871, -39.2379684, 2580.

4251, 484.567871, -39.2379684, 2560.

4252, 484.567871, -39.2379684, 2540.

4253, 514.28241, -39.2855949, 2740.

4254, 514.28241, -39.2855949, 2720.

4255, 514.28241, -39.2855949, 2700.

4256, 514.28241, -39.2855949, 2680.

4257, 514.28241, -39.2855949, 2660.

4258, 514.28241, -39.2855949, 2640.

4259, 514.28241, -39.2855949, 2620.

4260, 514.28241, -39.2855949, 2600.

4261, 514.28241, -39.2855949, 2580.

4262, 514.28241, -39.2855949, 2560.

4263, 514.28241, -39.2855949, 2540.

4264, 543.996887, -39.3332214, 2740.

4265, 543.996887, -39.3332214, 2720.

4266, 543.996887, -39.3332214, 2700.

4267, 543.996887, -39.3332214, 2680.

4268, 543.996887, -39.3332214, 2660.

4269, 543.996887, -39.3332214, 2640.

4270, 543.996887, -39.3332214, 2620.

4271, 543.996887, -39.3332214, 2600.

4272, 543.996887, -39.3332214, 2580.

4273, 543.996887, -39.3332214, 2560.

4274, 543.996887, -39.3332214, 2540.

4275, 573.711426, -39.3808479, 2740.

4276, 573.711426, -39.3808479, 2720.

4277, 573.711426, -39.3808479, 2700.

4278, 573.711426, -39.3808479, 2680.

4279, 573.711426, -39.3808479, 2660.

4280, 573.711426, -39.3808479, 2640.

4281, 573.711426, -39.3808479, 2620.

4282, 573.711426, -39.3808479, 2600.

4283, 573.711426, -39.3808479, 2580.

4284, 573.711426, -39.3808479, 2560.

4285, 573.711426, -39.3808479, 2540.

4286, 603.425903, -39.4284782, 2740.

4287, 603.425903, -39.4284782, 2720.

4288, 603.425903, -39.4284782, 2700.

4289, 603.425903, -39.4284782, 2680.

4290, 603.425903, -39.4284782, 2660.

4291, 603.425903, -39.4284782, 2640.

4292, 603.425903, -39.4284782, 2620.

4293, 603.425903, -39.4284782, 2600.

4294, 603.425903, -39.4284782, 2580.

4295, 603.425903, -39.4284782, 2560.

4296, 603.425903, -39.4284782, 2540.

4297, 633.140442, -39.4761047, 2740.

4298, 633.140442, -39.4761047, 2720.

4299, 633.140442, -39.4761047, 2700.

4300, 633.140442, -39.4761047, 2680.

4301, 633.140442, -39.4761047, 2660.

4302, 633.140442, -39.4761047, 2640.

4303, 633.140442, -39.4761047, 2620.

4304, 633.140442, -39.4761047, 2600.

4305, 633.140442, -39.4761047, 2580.

4306, 633.140442, -39.4761047, 2560.

4307, 633.140442, -39.4761047, 2540.

4308, 662.854919, -39.5237312, 2740.

4309, 662.854919, -39.5237312, 2720.

4310, 662.854919, -39.5237312, 2700.

4311, 662.854919, -39.5237312, 2680.

4312, 662.854919, -39.5237312, 2660.

4313, 662.854919, -39.5237312, 2640.

4314, 662.854919, -39.5237312, 2620.

4315, 662.854919, -39.5237312, 2600.

4316, 662.854919, -39.5237312, 2580.

4317, 662.854919, -39.5237312, 2560.

4318, 662.854919, -39.5237312, 2540.

4319, 692.569458, -39.5713577, 2740.

4320, 692.569458, -39.5713577, 2720.

4321, 692.569458, -39.5713577, 2700.

4322, 692.569458, -39.5713577, 2680.

4323, 692.569458, -39.5713577, 2660.

4324, 692.569458, -39.5713577, 2640.

4325, 692.569458, -39.5713577, 2620.

4326, 692.569458, -39.5713577, 2600.

4327, 692.569458, -39.5713577, 2580.

4328, 692.569458, -39.5713577, 2560.

4329, 692.569458, -39.5713577, 2540.

4330, 722.283936, -39.6189842, 2740.

4331, 722.283936, -39.6189842, 2720.

4332, 722.283936, -39.6189842, 2700.

4333, 722.283936, -39.6189842, 2680.

4334, 722.283936, -39.6189842, 2660.

4335, 722.283936, -39.6189842, 2640.

4336, 722.283936, -39.6189842, 2620.

4337, 722.283936, -39.6189842, 2600.

4338, 722.283936, -39.6189842, 2580.

4339, 722.283936, -39.6189842, 2560.

4340, 722.283936, -39.6189842, 2540.

4341, 751.998474, -39.6666107, 2740.

4342, 751.998474, -39.6666107, 2720.

4343, 751.998474, -39.6666107, 2700.

4344, 751.998474, -39.6666107, 2680.

4345, 751.998474, -39.6666107, 2660.

4346, 751.998474, -39.6666107, 2640.

4347, 751.998474, -39.6666107, 2620.

4348, 751.998474, -39.6666107, 2600.

4349, 751.998474, -39.6666107, 2580.

4350, 751.998474, -39.6666107, 2560.

4351, 751.998474, -39.6666107, 2540.

4352, 781.712952, -39.7142372, 2740.

4353, 781.712952, -39.7142372, 2720.

4354, 781.712952, -39.7142372, 2700.

4355, 781.712952, -39.7142372, 2680.

4356, 781.712952, -39.7142372, 2660.

4357, 781.712952, -39.7142372, 2640.

4358, 781.712952, -39.7142372, 2620.

4359, 781.712952, -39.7142372, 2600.

4360, 781.712952, -39.7142372, 2580.

4361, 781.712952, -39.7142372, 2560.

4362, 781.712952, -39.7142372, 2540.

4363, 811.42749, -39.7618637, 2740.

4364, 811.42749, -39.7618637, 2720.

4365, 811.42749, -39.7618637, 2700.

4366, 811.42749, -39.7618637, 2680.

4367, 811.42749, -39.7618637, 2660.

4368, 811.42749, -39.7618637, 2640.

4369, 811.42749, -39.7618637, 2620.

4370, 811.42749, -39.7618637, 2600.

4371, 811.42749, -39.7618637, 2580.

4372, 811.42749, -39.7618637, 2560.

4373, 811.42749, -39.7618637, 2540.

4374, 841.141968, -39.809494, 2740.

4375, 841.141968, -39.809494, 2720.

4376, 841.141968, -39.809494, 2700.

4377, 841.141968, -39.809494, 2680.

4378, 841.141968, -39.809494, 2660.

4379, 841.141968, -39.809494, 2640.

4380, 841.141968, -39.809494, 2620.

4381, 841.141968, -39.809494, 2600.

4382, 841.141968, -39.809494, 2580.

4383, 841.141968, -39.809494, 2560.

4384, 841.141968, -39.809494, 2540.

4385, 870.856506, -39.8571205, 2740.

4386, 870.856506, -39.8571205, 2720.

4387, 870.856506, -39.8571205, 2700.

4388, 870.856506, -39.8571205, 2680.

4389, 870.856506, -39.8571205, 2660.

4390, 870.856506, -39.8571205, 2640.

4391, 870.856506, -39.8571205, 2620.

4392, 870.856506, -39.8571205, 2600.

4393, 870.856506, -39.8571205, 2580.

4394, 870.856506, -39.8571205, 2560.

4395, 870.856506, -39.8571205, 2540.

4396, 900.570984, -39.904747, 2740.

4397, 900.570984, -39.904747, 2720.

4398, 900.570984, -39.904747, 2700.

4399, 900.570984, -39.904747, 2680.

4400, 900.570984, -39.904747, 2660.

4401, 900.570984, -39.904747, 2640.

4402, 900.570984, -39.904747, 2620.

4403, 900.570984, -39.904747, 2600.

4404, 900.570984, -39.904747, 2580.

4405, 900.570984, -39.904747, 2560.

4406, 900.570984, -39.904747, 2540.

4407, 930.285522, -39.9523735, 2740.

4408, 930.285522, -39.9523735, 2720.

4409, 930.285522, -39.9523735, 2700.

4410, 930.285522, -39.9523735, 2680.

4411, 930.285522, -39.9523735, 2660.

4412, 930.285522, -39.9523735, 2640.

4413, 930.285522, -39.9523735, 2620.

4414, 930.285522, -39.9523735, 2600.

4415, 930.285522, -39.9523735, 2580.

4416, 930.285522, -39.9523735, 2560.

4417, 930.285522, -39.9523735, 2540.

4418, 180.903442, -18.4628887, 2740.

4419, 180.903442, -18.4628887, 2720.

4420, 180.903442, -18.4628887, 2700.

4421, 180.903442, -18.4628887, 2680.

4422, 180.903442, -18.4628887, 2660.

4423, 180.903442, -18.4628887, 2640.

4424, 180.903442, -18.4628887, 2620.

4425, 180.903442, -18.4628887, 2600.

4426, 180.903442, -18.4628887, 2580.

4427, 180.903442, -18.4628887, 2560.

4428, 180.903442, -18.4628887, 2540.

4429, 210.868698, -18.5220089, 2740.

4430, 210.868698, -18.5220089, 2720.

4431, 210.868698, -18.5220089, 2700.

4432, 210.868698, -18.5220089, 2680.

4433, 210.868698, -18.5220089, 2660.

4434, 210.868698, -18.5220089, 2640.

4435, 210.868698, -18.5220089, 2620.

4436, 210.868698, -18.5220089, 2600.

4437, 210.868698, -18.5220089, 2580.

4438, 210.868698, -18.5220089, 2560.

4439, 210.868698, -18.5220089, 2540.

4440, 240.833939, -18.5811291, 2740.

4441, 240.833939, -18.5811291, 2720.

4442, 240.833939, -18.5811291, 2700.

4443, 240.833939, -18.5811291, 2680.

4444, 240.833939, -18.5811291, 2660.

4445, 240.833939, -18.5811291, 2640.

4446, 240.833939, -18.5811291, 2620.

4447, 240.833939, -18.5811291, 2600.

4448, 240.833939, -18.5811291, 2580.

4449, 240.833939, -18.5811291, 2560.

4450, 240.833939, -18.5811291, 2540.

4451, 270.799194, -18.6402493, 2740.

4452, 270.799194, -18.6402493, 2720.

4453, 270.799194, -18.6402493, 2700.

4454, 270.799194, -18.6402493, 2680.

4455, 270.799194, -18.6402493, 2660.

4456, 270.799194, -18.6402493, 2640.

4457, 270.799194, -18.6402493, 2620.

4458, 270.799194, -18.6402493, 2600.

4459, 270.799194, -18.6402493, 2580.

4460, 270.799194, -18.6402493, 2560.

4461, 270.799194, -18.6402493, 2540.

4462, 300.764435, -18.6993675, 2740.

4463, 300.764435, -18.6993675, 2720.

4464, 300.764435, -18.6993675, 2700.

4465, 300.764435, -18.6993675, 2680.

4466, 300.764435, -18.6993675, 2660.

4467, 300.764435, -18.6993675, 2640.

4468, 300.764435, -18.6993675, 2620.

4469, 300.764435, -18.6993675, 2600.

4470, 300.764435, -18.6993675, 2580.

4471, 300.764435, -18.6993675, 2560.

4472, 300.764435, -18.6993675, 2540.

4473, 330.729706, -18.7584877, 2740.

4474, 330.729706, -18.7584877, 2720.

4475, 330.729706, -18.7584877, 2700.

4476, 330.729706, -18.7584877, 2680.

4477, 330.729706, -18.7584877, 2660.

4478, 330.729706, -18.7584877, 2640.

4479, 330.729706, -18.7584877, 2620.

4480, 330.729706, -18.7584877, 2600.

4481, 330.729706, -18.7584877, 2580.

4482, 330.729706, -18.7584877, 2560.

4483, 330.729706, -18.7584877, 2540.

4484, 360.694946, -18.8176079, 2740.

4485, 360.694946, -18.8176079, 2720.

4486, 360.694946, -18.8176079, 2700.

4487, 360.694946, -18.8176079, 2680.

4488, 360.694946, -18.8176079, 2660.

4489, 360.694946, -18.8176079, 2640.

4490, 360.694946, -18.8176079, 2620.

4491, 360.694946, -18.8176079, 2600.

4492, 360.694946, -18.8176079, 2580.

4493, 360.694946, -18.8176079, 2560.

4494, 360.694946, -18.8176079, 2540.

4495, 390.660217, -18.8767262, 2740.

4496, 390.660217, -18.8767262, 2720.

4497, 390.660217, -18.8767262, 2700.

4498, 390.660217, -18.8767262, 2680.

4499, 390.660217, -18.8767262, 2660.

4500, 390.660217, -18.8767262, 2640.

4501, 390.660217, -18.8767262, 2620.

4502, 390.660217, -18.8767262, 2600.

4503, 390.660217, -18.8767262, 2580.

4504, 390.660217, -18.8767262, 2560.

4505, 390.660217, -18.8767262, 2540.

4506, 420.625458, -18.9358463, 2740.

4507, 420.625458, -18.9358463, 2720.

4508, 420.625458, -18.9358463, 2700.

4509, 420.625458, -18.9358463, 2680.

4510, 420.625458, -18.9358463, 2660.

4511, 420.625458, -18.9358463, 2640.

4512, 420.625458, -18.9358463, 2620.

4513, 420.625458, -18.9358463, 2600.

4514, 420.625458, -18.9358463, 2580.

4515, 420.625458, -18.9358463, 2560.

4516, 420.625458, -18.9358463, 2540.

4517, 450.590698, -18.9949665, 2740.

4518, 450.590698, -18.9949665, 2720.

4519, 450.590698, -18.9949665, 2700.

4520, 450.590698, -18.9949665, 2680.

4521, 450.590698, -18.9949665, 2660.

4522, 450.590698, -18.9949665, 2640.

4523, 450.590698, -18.9949665, 2620.

4524, 450.590698, -18.9949665, 2600.

4525, 450.590698, -18.9949665, 2580.

4526, 450.590698, -18.9949665, 2560.

4527, 450.590698, -18.9949665, 2540.

4528, 480.555969, -19.0540867, 2740.

4529, 480.555969, -19.0540867, 2720.

4530, 480.555969, -19.0540867, 2700.

4531, 480.555969, -19.0540867, 2680.

4532, 480.555969, -19.0540867, 2660.

4533, 480.555969, -19.0540867, 2640.

4534, 480.555969, -19.0540867, 2620.

4535, 480.555969, -19.0540867, 2600.

4536, 480.555969, -19.0540867, 2580.

4537, 480.555969, -19.0540867, 2560.

4538, 480.555969, -19.0540867, 2540.

4539, 510.52121, -19.113205, 2740.

4540, 510.52121, -19.113205, 2720.

4541, 510.52121, -19.113205, 2700.

4542, 510.52121, -19.113205, 2680.

4543, 510.52121, -19.113205, 2660.

4544, 510.52121, -19.113205, 2640.

4545, 510.52121, -19.113205, 2620.

4546, 510.52121, -19.113205, 2600.

4547, 510.52121, -19.113205, 2580.

4548, 510.52121, -19.113205, 2560.

4549, 510.52121, -19.113205, 2540.

4550, 540.48645, -19.1723251, 2740.

4551, 540.48645, -19.1723251, 2720.

4552, 540.48645, -19.1723251, 2700.

4553, 540.48645, -19.1723251, 2680.

4554, 540.48645, -19.1723251, 2660.

4555, 540.48645, -19.1723251, 2640.

4556, 540.48645, -19.1723251, 2620.

4557, 540.48645, -19.1723251, 2600.

4558, 540.48645, -19.1723251, 2580.

4559, 540.48645, -19.1723251, 2560.

4560, 540.48645, -19.1723251, 2540.

4561, 570.451721, -19.2314453, 2740.

4562, 570.451721, -19.2314453, 2720.

4563, 570.451721, -19.2314453, 2700.

4564, 570.451721, -19.2314453, 2680.

4565, 570.451721, -19.2314453, 2660.

4566, 570.451721, -19.2314453, 2640.

4567, 570.451721, -19.2314453, 2620.

4568, 570.451721, -19.2314453, 2600.

4569, 570.451721, -19.2314453, 2580.

4570, 570.451721, -19.2314453, 2560.

4571, 570.451721, -19.2314453, 2540.

4572, 600.416992, -19.2905636, 2740.

4573, 600.416992, -19.2905636, 2720.

4574, 600.416992, -19.2905636, 2700.

4575, 600.416992, -19.2905636, 2680.

4576, 600.416992, -19.2905636, 2660.

4577, 600.416992, -19.2905636, 2640.

4578, 600.416992, -19.2905636, 2620.

4579, 600.416992, -19.2905636, 2600.

4580, 600.416992, -19.2905636, 2580.

4581, 600.416992, -19.2905636, 2560.

4582, 600.416992, -19.2905636, 2540.

4583, 630.382202, -19.3496838, 2740.

4584, 630.382202, -19.3496838, 2720.

4585, 630.382202, -19.3496838, 2700.

4586, 630.382202, -19.3496838, 2680.

4587, 630.382202, -19.3496838, 2660.

4588, 630.382202, -19.3496838, 2640.

4589, 630.382202, -19.3496838, 2620.

4590, 630.382202, -19.3496838, 2600.

4591, 630.382202, -19.3496838, 2580.

4592, 630.382202, -19.3496838, 2560.

4593, 630.382202, -19.3496838, 2540.

4594, 660.347473, -19.4088039, 2740.

4595, 660.347473, -19.4088039, 2720.

4596, 660.347473, -19.4088039, 2700.

4597, 660.347473, -19.4088039, 2680.

4598, 660.347473, -19.4088039, 2660.

4599, 660.347473, -19.4088039, 2640.

4600, 660.347473, -19.4088039, 2620.

4601, 660.347473, -19.4088039, 2600.

4602, 660.347473, -19.4088039, 2580.

4603, 660.347473, -19.4088039, 2560.

4604, 660.347473, -19.4088039, 2540.

4605, 690.312744, -19.4679241, 2740.

4606, 690.312744, -19.4679241, 2720.

4607, 690.312744, -19.4679241, 2700.

4608, 690.312744, -19.4679241, 2680.

4609, 690.312744, -19.4679241, 2660.

4610, 690.312744, -19.4679241, 2640.

4611, 690.312744, -19.4679241, 2620.

4612, 690.312744, -19.4679241, 2600.

4613, 690.312744, -19.4679241, 2580.

4614, 690.312744, -19.4679241, 2560.

4615, 690.312744, -19.4679241, 2540.

4616, 720.277954, -19.5270424, 2740.

4617, 720.277954, -19.5270424, 2720.

4618, 720.277954, -19.5270424, 2700.

4619, 720.277954, -19.5270424, 2680.

4620, 720.277954, -19.5270424, 2660.

4621, 720.277954, -19.5270424, 2640.

4622, 720.277954, -19.5270424, 2620.

4623, 720.277954, -19.5270424, 2600.

4624, 720.277954, -19.5270424, 2580.

4625, 720.277954, -19.5270424, 2560.

4626, 720.277954, -19.5270424, 2540.

4627, 750.243225, -19.5861626, 2740.

4628, 750.243225, -19.5861626, 2720.

4629, 750.243225, -19.5861626, 2700.

4630, 750.243225, -19.5861626, 2680.

4631, 750.243225, -19.5861626, 2660.

4632, 750.243225, -19.5861626, 2640.

4633, 750.243225, -19.5861626, 2620.

4634, 750.243225, -19.5861626, 2600.

4635, 750.243225, -19.5861626, 2580.

4636, 750.243225, -19.5861626, 2560.

4637, 750.243225, -19.5861626, 2540.

4638, 780.208496, -19.6452827, 2740.

4639, 780.208496, -19.6452827, 2720.

4640, 780.208496, -19.6452827, 2700.

4641, 780.208496, -19.6452827, 2680.

4642, 780.208496, -19.6452827, 2660.

4643, 780.208496, -19.6452827, 2640.

4644, 780.208496, -19.6452827, 2620.

4645, 780.208496, -19.6452827, 2600.

4646, 780.208496, -19.6452827, 2580.

4647, 780.208496, -19.6452827, 2560.

4648, 780.208496, -19.6452827, 2540.

4649, 810.173767, -19.704401, 2740.

4650, 810.173767, -19.704401, 2720.

4651, 810.173767, -19.704401, 2700.

4652, 810.173767, -19.704401, 2680.

4653, 810.173767, -19.704401, 2660.

4654, 810.173767, -19.704401, 2640.

4655, 810.173767, -19.704401, 2620.

4656, 810.173767, -19.704401, 2600.

4657, 810.173767, -19.704401, 2580.

4658, 810.173767, -19.704401, 2560.

4659, 810.173767, -19.704401, 2540.

4660, 840.138977, -19.7635212, 2740.

4661, 840.138977, -19.7635212, 2720.

4662, 840.138977, -19.7635212, 2700.

4663, 840.138977, -19.7635212, 2680.

4664, 840.138977, -19.7635212, 2660.

4665, 840.138977, -19.7635212, 2640.

4666, 840.138977, -19.7635212, 2620.

4667, 840.138977, -19.7635212, 2600.

4668, 840.138977, -19.7635212, 2580.

4669, 840.138977, -19.7635212, 2560.

4670, 840.138977, -19.7635212, 2540.

4671, 870.104248, -19.8226414, 2740.

4672, 870.104248, -19.8226414, 2720.

4673, 870.104248, -19.8226414, 2700.

4674, 870.104248, -19.8226414, 2680.

4675, 870.104248, -19.8226414, 2660.

4676, 870.104248, -19.8226414, 2640.

4677, 870.104248, -19.8226414, 2620.

4678, 870.104248, -19.8226414, 2600.

4679, 870.104248, -19.8226414, 2580.

4680, 870.104248, -19.8226414, 2560.

4681, 870.104248, -19.8226414, 2540.

4682, 900.069519, -19.8817616, 2740.

4683, 900.069519, -19.8817616, 2720.

4684, 900.069519, -19.8817616, 2700.

4685, 900.069519, -19.8817616, 2680.

4686, 900.069519, -19.8817616, 2660.

4687, 900.069519, -19.8817616, 2640.

4688, 900.069519, -19.8817616, 2620.

4689, 900.069519, -19.8817616, 2600.

4690, 900.069519, -19.8817616, 2580.

4691, 900.069519, -19.8817616, 2560.

4692, 900.069519, -19.8817616, 2540.

4693, 930.034729, -19.9408798, 2740.

4694, 930.034729, -19.9408798, 2720.

4695, 930.034729, -19.9408798, 2700.

4696, 930.034729, -19.9408798, 2680.

4697, 930.034729, -19.9408798, 2660.

4698, 930.034729, -19.9408798, 2640.

4699, 930.034729, -19.9408798, 2620.

4700, 930.034729, -19.9408798, 2600.

4701, 930.034729, -19.9408798, 2580.

4702, 930.034729, -19.9408798, 2560.

4703, 930.034729, -19.9408798, 2540.

4704, 1190.25647, -100., 2740.

4705, 1190.25647, -100., 2720.

4706, 1190.25647, -100., 2700.

4707, 1190.25647, -100., 2680.

4708, 1190.25647, -100., 2660.

4709, 1190.25647, -100., 2640.

4710, 1190.25647, -100., 2620.

4711, 1190.25647, -100., 2600.

4712, 1190.25647, -100., 2580.

4713, 1190.25647, -100., 2560.

4714, 1190.25647, -100., 2540.

4715, 1220.51282, -100., 2740.

4716, 1220.51282, -100., 2720.

4717, 1220.51282, -100., 2700.

4718, 1220.51282, -100., 2680.

4719, 1220.51282, -100., 2660.

4720, 1220.51282, -100., 2640.

4721, 1220.51282, -100., 2620.

4722, 1220.51282, -100., 2600.

4723, 1220.51282, -100., 2580.

4724, 1220.51282, -100., 2560.

4725, 1220.51282, -100., 2540.

4726, 1250.76929, -100., 2740.

4727, 1250.76929, -100., 2720.

4728, 1250.76929, -100., 2700.

4729, 1250.76929, -100., 2680.

4730, 1250.76929, -100., 2660.

4731, 1250.76929, -100., 2640.

4732, 1250.76929, -100., 2620.

4733, 1250.76929, -100., 2600.

4734, 1250.76929, -100., 2580.

4735, 1250.76929, -100., 2560.

4736, 1250.76929, -100., 2540.

4737, 1281.02563, -100., 2740.

4738, 1281.02563, -100., 2720.

4739, 1281.02563, -100., 2700.

4740, 1281.02563, -100., 2680.

4741, 1281.02563, -100., 2660.

4742, 1281.02563, -100., 2640.

4743, 1281.02563, -100., 2620.

4744, 1281.02563, -100., 2600.

4745, 1281.02563, -100., 2580.

4746, 1281.02563, -100., 2560.

4747, 1281.02563, -100., 2540.

4748, 1311.2821, -100., 2740.

4749, 1311.2821, -100., 2720.

4750, 1311.2821, -100., 2700.

4751, 1311.2821, -100., 2680.

4752, 1311.2821, -100., 2660.

4753, 1311.2821, -100., 2640.

4754, 1311.2821, -100., 2620.

4755, 1311.2821, -100., 2600.

4756, 1311.2821, -100., 2580.

4757, 1311.2821, -100., 2560.

4758, 1311.2821, -100., 2540.

4759, 1341.53845, -100., 2740.

4760, 1341.53845, -100., 2720.

4761, 1341.53845, -100., 2700.

4762, 1341.53845, -100., 2680.

4763, 1341.53845, -100., 2660.

4764, 1341.53845, -100., 2640.

4765, 1341.53845, -100., 2620.

4766, 1341.53845, -100., 2600.

4767, 1341.53845, -100., 2580.

4768, 1341.53845, -100., 2560.

4769, 1341.53845, -100., 2540.

4770, 1371.79492, -100., 2740.

4771, 1371.79492, -100., 2720.

4772, 1371.79492, -100., 2700.

4773, 1371.79492, -100., 2680.

4774, 1371.79492, -100., 2660.

4775, 1371.79492, -100., 2640.

4776, 1371.79492, -100., 2620.

4777, 1371.79492, -100., 2600.

4778, 1371.79492, -100., 2580.

4779, 1371.79492, -100., 2560.

4780, 1371.79492, -100., 2540.

4781, 1402.05127, -100., 2740.

4782, 1402.05127, -100., 2720.

4783, 1402.05127, -100., 2700.

4784, 1402.05127, -100., 2680.

4785, 1402.05127, -100., 2660.

4786, 1402.05127, -100., 2640.

4787, 1402.05127, -100., 2620.

4788, 1402.05127, -100., 2600.

4789, 1402.05127, -100., 2580.

4790, 1402.05127, -100., 2560.

4791, 1402.05127, -100., 2540.

4792, 1432.30774, -100., 2740.

4793, 1432.30774, -100., 2720.

4794, 1432.30774, -100., 2700.

4795, 1432.30774, -100., 2680.

4796, 1432.30774, -100., 2660.

4797, 1432.30774, -100., 2640.

4798, 1432.30774, -100., 2620.

4799, 1432.30774, -100., 2600.

4800, 1432.30774, -100., 2580.

4801, 1432.30774, -100., 2560.

4802, 1432.30774, -100., 2540.

4803, 1462.56409, -100., 2740.

4804, 1462.56409, -100., 2720.

4805, 1462.56409, -100., 2700.

4806, 1462.56409, -100., 2680.

4807, 1462.56409, -100., 2660.

4808, 1462.56409, -100., 2640.

4809, 1462.56409, -100., 2620.

4810, 1462.56409, -100., 2600.

4811, 1462.56409, -100., 2580.

4812, 1462.56409, -100., 2560.

4813, 1462.56409, -100., 2540.

4814, 1492.82056, -100., 2740.

4815, 1492.82056, -100., 2720.

4816, 1492.82056, -100., 2700.

4817, 1492.82056, -100., 2680.

4818, 1492.82056, -100., 2660.

4819, 1492.82056, -100., 2640.

4820, 1492.82056, -100., 2620.

4821, 1492.82056, -100., 2600.

4822, 1492.82056, -100., 2580.

4823, 1492.82056, -100., 2560.

4824, 1492.82056, -100., 2540.

4825, 1523.0769, -100., 2740.

4826, 1523.0769, -100., 2720.

4827, 1523.0769, -100., 2700.

4828, 1523.0769, -100., 2680.

4829, 1523.0769, -100., 2660.

4830, 1523.0769, -100., 2640.

4831, 1523.0769, -100., 2620.

4832, 1523.0769, -100., 2600.

4833, 1523.0769, -100., 2580.

4834, 1523.0769, -100., 2560.

4835, 1523.0769, -100., 2540.

4836, 1553.33337, -100., 2740.

4837, 1553.33337, -100., 2720.

4838, 1553.33337, -100., 2700.

4839, 1553.33337, -100., 2680.

4840, 1553.33337, -100., 2660.

4841, 1553.33337, -100., 2640.

4842, 1553.33337, -100., 2620.

4843, 1553.33337, -100., 2600.

4844, 1553.33337, -100., 2580.

4845, 1553.33337, -100., 2560.

4846, 1553.33337, -100., 2540.

4847, 1583.58972, -100., 2740.

4848, 1583.58972, -100., 2720.

4849, 1583.58972, -100., 2700.

4850, 1583.58972, -100., 2680.

4851, 1583.58972, -100., 2660.

4852, 1583.58972, -100., 2640.

4853, 1583.58972, -100., 2620.

4854, 1583.58972, -100., 2600.

4855, 1583.58972, -100., 2580.

4856, 1583.58972, -100., 2560.

4857, 1583.58972, -100., 2540.

4858, 1613.84619, -100., 2740.

4859, 1613.84619, -100., 2720.

4860, 1613.84619, -100., 2700.

4861, 1613.84619, -100., 2680.

4862, 1613.84619, -100., 2660.

4863, 1613.84619, -100., 2640.

4864, 1613.84619, -100., 2620.

4865, 1613.84619, -100., 2600.

4866, 1613.84619, -100., 2580.

4867, 1613.84619, -100., 2560.

4868, 1613.84619, -100., 2540.

4869, 1644.10254, -100., 2740.

4870, 1644.10254, -100., 2720.

4871, 1644.10254, -100., 2700.

4872, 1644.10254, -100., 2680.

4873, 1644.10254, -100., 2660.

4874, 1644.10254, -100., 2640.

4875, 1644.10254, -100., 2620.

4876, 1644.10254, -100., 2600.

4877, 1644.10254, -100., 2580.

4878, 1644.10254, -100., 2560.

4879, 1644.10254, -100., 2540.

4880, 1674.35901, -100., 2740.

4881, 1674.35901, -100., 2720.

4882, 1674.35901, -100., 2700.

4883, 1674.35901, -100., 2680.

4884, 1674.35901, -100., 2660.

4885, 1674.35901, -100., 2640.

4886, 1674.35901, -100., 2620.

4887, 1674.35901, -100., 2600.

4888, 1674.35901, -100., 2580.

4889, 1674.35901, -100., 2560.

4890, 1674.35901, -100., 2540.

4891, 1704.61536, -100., 2740.

4892, 1704.61536, -100., 2720.

4893, 1704.61536, -100., 2700.

4894, 1704.61536, -100., 2680.

4895, 1704.61536, -100., 2660.

4896, 1704.61536, -100., 2640.

4897, 1704.61536, -100., 2620.

4898, 1704.61536, -100., 2600.

4899, 1704.61536, -100., 2580.

4900, 1704.61536, -100., 2560.

4901, 1704.61536, -100., 2540.

4902, 1734.87183, -100., 2740.

4903, 1734.87183, -100., 2720.

4904, 1734.87183, -100., 2700.

4905, 1734.87183, -100., 2680.

4906, 1734.87183, -100., 2660.

4907, 1734.87183, -100., 2640.

4908, 1734.87183, -100., 2620.

4909, 1734.87183, -100., 2600.

4910, 1734.87183, -100., 2580.

4911, 1734.87183, -100., 2560.

4912, 1734.87183, -100., 2540.

4913, 1765.12817, -100., 2740.

4914, 1765.12817, -100., 2720.

4915, 1765.12817, -100., 2700.

4916, 1765.12817, -100., 2680.

4917, 1765.12817, -100., 2660.

4918, 1765.12817, -100., 2640.

4919, 1765.12817, -100., 2620.

4920, 1765.12817, -100., 2600.

4921, 1765.12817, -100., 2580.

4922, 1765.12817, -100., 2560.

4923, 1765.12817, -100., 2540.

4924, 1795.38464, -100., 2740.

4925, 1795.38464, -100., 2720.

4926, 1795.38464, -100., 2700.

4927, 1795.38464, -100., 2680.

4928, 1795.38464, -100., 2660.

4929, 1795.38464, -100., 2640.

4930, 1795.38464, -100., 2620.

4931, 1795.38464, -100., 2600.

4932, 1795.38464, -100., 2580.

4933, 1795.38464, -100., 2560.

4934, 1795.38464, -100., 2540.

4935, 1825.64099, -100., 2740.

4936, 1825.64099, -100., 2720.

4937, 1825.64099, -100., 2700.

4938, 1825.64099, -100., 2680.

4939, 1825.64099, -100., 2660.

4940, 1825.64099, -100., 2640.

4941, 1825.64099, -100., 2620.

4942, 1825.64099, -100., 2600.

4943, 1825.64099, -100., 2580.

4944, 1825.64099, -100., 2560.

4945, 1825.64099, -100., 2540.

4946, 1855.89746, -100., 2740.

4947, 1855.89746, -100., 2720.

4948, 1855.89746, -100., 2700.

4949, 1855.89746, -100., 2680.

4950, 1855.89746, -100., 2660.

4951, 1855.89746, -100., 2640.

4952, 1855.89746, -100., 2620.

4953, 1855.89746, -100., 2600.

4954, 1855.89746, -100., 2580.

4955, 1855.89746, -100., 2560.

4956, 1855.89746, -100., 2540.

4957, 1886.15381, -100., 2740.

4958, 1886.15381, -100., 2720.

4959, 1886.15381, -100., 2700.

4960, 1886.15381, -100., 2680.

4961, 1886.15381, -100., 2660.

4962, 1886.15381, -100., 2640.

4963, 1886.15381, -100., 2620.

4964, 1886.15381, -100., 2600.

4965, 1886.15381, -100., 2580.

4966, 1886.15381, -100., 2560.

4967, 1886.15381, -100., 2540.

4968, 1916.41028, -100., 2740.

4969, 1916.41028, -100., 2720.

4970, 1916.41028, -100., 2700.

4971, 1916.41028, -100., 2680.

4972, 1916.41028, -100., 2660.

4973, 1916.41028, -100., 2640.

4974, 1916.41028, -100., 2620.

4975, 1916.41028, -100., 2600.

4976, 1916.41028, -100., 2580.

4977, 1916.41028, -100., 2560.

4978, 1916.41028, -100., 2540.

4979, 1946.66663, -100., 2740.

4980, 1946.66663, -100., 2720.

4981, 1946.66663, -100., 2700.

4982, 1946.66663, -100., 2680.

4983, 1946.66663, -100., 2660.

4984, 1946.66663, -100., 2640.

4985, 1946.66663, -100., 2620.

4986, 1946.66663, -100., 2600.

4987, 1946.66663, -100., 2580.

4988, 1946.66663, -100., 2560.

4989, 1946.66663, -100., 2540.

4990, 1976.9231, -100., 2740.

4991, 1976.9231, -100., 2720.

4992, 1976.9231, -100., 2700.

4993, 1976.9231, -100., 2680.

4994, 1976.9231, -100., 2660.

4995, 1976.9231, -100., 2640.

4996, 1976.9231, -100., 2620.

4997, 1976.9231, -100., 2600.

4998, 1976.9231, -100., 2580.

4999, 1976.9231, -100., 2560.

5000, 1976.9231, -100., 2540.

5001, 2007.17944, -100., 2740.

5002, 2007.17944, -100., 2720.

5003, 2007.17944, -100., 2700.

5004, 2007.17944, -100., 2680.

5005, 2007.17944, -100., 2660.

5006, 2007.17944, -100., 2640.

5007, 2007.17944, -100., 2620.

5008, 2007.17944, -100., 2600.

5009, 2007.17944, -100., 2580.

5010, 2007.17944, -100., 2560.

5011, 2007.17944, -100., 2540.

5012, 2037.43591, -100., 2740.

5013, 2037.43591, -100., 2720.

5014, 2037.43591, -100., 2700.

5015, 2037.43591, -100., 2680.

5016, 2037.43591, -100., 2660.

5017, 2037.43591, -100., 2640.

5018, 2037.43591, -100., 2620.

5019, 2037.43591, -100., 2600.

5020, 2037.43591, -100., 2580.

5021, 2037.43591, -100., 2560.

5022, 2037.43591, -100., 2540.

5023, 2067.69238, -100., 2740.

5024, 2067.69238, -100., 2720.

5025, 2067.69238, -100., 2700.

5026, 2067.69238, -100., 2680.

5027, 2067.69238, -100., 2660.

5028, 2067.69238, -100., 2640.

5029, 2067.69238, -100., 2620.

5030, 2067.69238, -100., 2600.

5031, 2067.69238, -100., 2580.

5032, 2067.69238, -100., 2560.

5033, 2067.69238, -100., 2540.

5034, 2097.94873, -100., 2740.

5035, 2097.94873, -100., 2720.

5036, 2097.94873, -100., 2700.

5037, 2097.94873, -100., 2680.

5038, 2097.94873, -100., 2660.

5039, 2097.94873, -100., 2640.

5040, 2097.94873, -100., 2620.

5041, 2097.94873, -100., 2600.

5042, 2097.94873, -100., 2580.

5043, 2097.94873, -100., 2560.

5044, 2097.94873, -100., 2540.

5045, 2128.20508, -100., 2740.

5046, 2128.20508, -100., 2720.

5047, 2128.20508, -100., 2700.

5048, 2128.20508, -100., 2680.

5049, 2128.20508, -100., 2660.

5050, 2128.20508, -100., 2640.

5051, 2128.20508, -100., 2620.

5052, 2128.20508, -100., 2600.

5053, 2128.20508, -100., 2580.

5054, 2128.20508, -100., 2560.

5055, 2128.20508, -100., 2540.

5056, 2158.46143, -100., 2740.

5057, 2158.46143, -100., 2720.

5058, 2158.46143, -100., 2700.

5059, 2158.46143, -100., 2680.

5060, 2158.46143, -100., 2660.

5061, 2158.46143, -100., 2640.

5062, 2158.46143, -100., 2620.

5063, 2158.46143, -100., 2600.

5064, 2158.46143, -100., 2580.

5065, 2158.46143, -100., 2560.

5066, 2158.46143, -100., 2540.

5067, 2188.71802, -100., 2740.

5068, 2188.71802, -100., 2720.

5069, 2188.71802, -100., 2700.

5070, 2188.71802, -100., 2680.

5071, 2188.71802, -100., 2660.

5072, 2188.71802, -100., 2640.

5073, 2188.71802, -100., 2620.

5074, 2188.71802, -100., 2600.

5075, 2188.71802, -100., 2580.

5076, 2188.71802, -100., 2560.

5077, 2188.71802, -100., 2540.

5078, 2218.97437, -100., 2740.

5079, 2218.97437, -100., 2720.

5080, 2218.97437, -100., 2700.

5081, 2218.97437, -100., 2680.

5082, 2218.97437, -100., 2660.

5083, 2218.97437, -100., 2640.

5084, 2218.97437, -100., 2620.

5085, 2218.97437, -100., 2600.

5086, 2218.97437, -100., 2580.

5087, 2218.97437, -100., 2560.

5088, 2218.97437, -100., 2540.

5089, 2249.23071, -100., 2740.

5090, 2249.23071, -100., 2720.

5091, 2249.23071, -100., 2700.

5092, 2249.23071, -100., 2680.

5093, 2249.23071, -100., 2660.

5094, 2249.23071, -100., 2640.

5095, 2249.23071, -100., 2620.

5096, 2249.23071, -100., 2600.

5097, 2249.23071, -100., 2580.

5098, 2249.23071, -100., 2560.

5099, 2249.23071, -100., 2540.

5100, 2279.48706, -100., 2740.

5101, 2279.48706, -100., 2720.

5102, 2279.48706, -100., 2700.

5103, 2279.48706, -100., 2680.

5104, 2279.48706, -100., 2660.

5105, 2279.48706, -100., 2640.

5106, 2279.48706, -100., 2620.

5107, 2279.48706, -100., 2600.

5108, 2279.48706, -100., 2580.

5109, 2279.48706, -100., 2560.

5110, 2279.48706, -100., 2540.

5111, 2309.74365, -100., 2740.

5112, 2309.74365, -100., 2720.

5113, 2309.74365, -100., 2700.

5114, 2309.74365, -100., 2680.

5115, 2309.74365, -100., 2660.

5116, 2309.74365, -100., 2640.

5117, 2309.74365, -100., 2620.

5118, 2309.74365, -100., 2600.

5119, 2309.74365, -100., 2580.

5120, 2309.74365, -100., 2560.

5121, 2309.74365, -100., 2540.

5122, 1190.25647, -80., 2740.

5123, 1190.25647, -80., 2720.

5124, 1190.25647, -80., 2700.

5125, 1190.25647, -80., 2680.

5126, 1190.25647, -80., 2660.

5127, 1190.25647, -80., 2640.

5128, 1190.25647, -80., 2620.

5129, 1190.25647, -80., 2600.

5130, 1190.25647, -80., 2580.

5131, 1190.25647, -80., 2560.

5132, 1190.25647, -80., 2540.

5133, 1220.51282, -80., 2740.

5134, 1220.51282, -80., 2720.

5135, 1220.51282, -80., 2700.

5136, 1220.51282, -80., 2680.

5137, 1220.51282, -80., 2660.

5138, 1220.51282, -80., 2640.

5139, 1220.51282, -80., 2620.

5140, 1220.51282, -80., 2600.

5141, 1220.51282, -80., 2580.

5142, 1220.51282, -80., 2560.

5143, 1220.51282, -80., 2540.

5144, 1250.76929, -80., 2740.

5145, 1250.76929, -80., 2720.

5146, 1250.76929, -80., 2700.

5147, 1250.76929, -80., 2680.

5148, 1250.76929, -80., 2660.

5149, 1250.76929, -80., 2640.

5150, 1250.76929, -80., 2620.

5151, 1250.76929, -80., 2600.

5152, 1250.76929, -80., 2580.

5153, 1250.76929, -80., 2560.

5154, 1250.76929, -80., 2540.

5155, 1281.02563, -80., 2740.

5156, 1281.02563, -80., 2720.

5157, 1281.02563, -80., 2700.

5158, 1281.02563, -80., 2680.

5159, 1281.02563, -80., 2660.

5160, 1281.02563, -80., 2640.

5161, 1281.02563, -80., 2620.

5162, 1281.02563, -80., 2600.

5163, 1281.02563, -80., 2580.

5164, 1281.02563, -80., 2560.

5165, 1281.02563, -80., 2540.

5166, 1311.2821, -80., 2740.

5167, 1311.2821, -80., 2720.

5168, 1311.2821, -80., 2700.

5169, 1311.2821, -80., 2680.

5170, 1311.2821, -80., 2660.

5171, 1311.2821, -80., 2640.

5172, 1311.2821, -80., 2620.

5173, 1311.2821, -80., 2600.

5174, 1311.2821, -80., 2580.

5175, 1311.2821, -80., 2560.

5176, 1311.2821, -80., 2540.

5177, 1341.53845, -80., 2740.

5178, 1341.53845, -80., 2720.

5179, 1341.53845, -80., 2700.

5180, 1341.53845, -80., 2680.

5181, 1341.53845, -80., 2660.

5182, 1341.53845, -80., 2640.

5183, 1341.53845, -80., 2620.

5184, 1341.53845, -80., 2600.

5185, 1341.53845, -80., 2580.

5186, 1341.53845, -80., 2560.

5187, 1341.53845, -80., 2540.

5188, 1371.79492, -80., 2740.

5189, 1371.79492, -80., 2720.

5190, 1371.79492, -80., 2700.

5191, 1371.79492, -80., 2680.

5192, 1371.79492, -80., 2660.

5193, 1371.79492, -80., 2640.

5194, 1371.79492, -80., 2620.

5195, 1371.79492, -80., 2600.

5196, 1371.79492, -80., 2580.

5197, 1371.79492, -80., 2560.

5198, 1371.79492, -80., 2540.

5199, 1402.05127, -80., 2740.

5200, 1402.05127, -80., 2720.

5201, 1402.05127, -80., 2700.

5202, 1402.05127, -80., 2680.

5203, 1402.05127, -80., 2660.

5204, 1402.05127, -80., 2640.

5205, 1402.05127, -80., 2620.

5206, 1402.05127, -80., 2600.

5207, 1402.05127, -80., 2580.

5208, 1402.05127, -80., 2560.

5209, 1402.05127, -80., 2540.

5210, 1432.30774, -80., 2740.

5211, 1432.30774, -80., 2720.

5212, 1432.30774, -80., 2700.

5213, 1432.30774, -80., 2680.

5214, 1432.30774, -80., 2660.

5215, 1432.30774, -80., 2640.

5216, 1432.30774, -80., 2620.

5217, 1432.30774, -80., 2600.

5218, 1432.30774, -80., 2580.

5219, 1432.30774, -80., 2560.

5220, 1432.30774, -80., 2540.

5221, 1462.56409, -80., 2740.

5222, 1462.56409, -80., 2720.

5223, 1462.56409, -80., 2700.

5224, 1462.56409, -80., 2680.

5225, 1462.56409, -80., 2660.

5226, 1462.56409, -80., 2640.

5227, 1462.56409, -80., 2620.

5228, 1462.56409, -80., 2600.

5229, 1462.56409, -80., 2580.

5230, 1462.56409, -80., 2560.

5231, 1462.56409, -80., 2540.

5232, 1492.82056, -80., 2740.

5233, 1492.82056, -80., 2720.

5234, 1492.82056, -80., 2700.

5235, 1492.82056, -80., 2680.

5236, 1492.82056, -80., 2660.

5237, 1492.82056, -80., 2640.

5238, 1492.82056, -80., 2620.

5239, 1492.82056, -80., 2600.

5240, 1492.82056, -80., 2580.

5241, 1492.82056, -80., 2560.

5242, 1492.82056, -80., 2540.

5243, 1523.0769, -80., 2740.

5244, 1523.0769, -80., 2720.

5245, 1523.0769, -80., 2700.

5246, 1523.0769, -80., 2680.

5247, 1523.0769, -80., 2660.

5248, 1523.0769, -80., 2640.

5249, 1523.0769, -80., 2620.

5250, 1523.0769, -80., 2600.

5251, 1523.0769, -80., 2580.

5252, 1523.0769, -80., 2560.

5253, 1523.0769, -80., 2540.

5254, 1553.33337, -80., 2740.

5255, 1553.33337, -80., 2720.

5256, 1553.33337, -80., 2700.

5257, 1553.33337, -80., 2680.

5258, 1553.33337, -80., 2660.

5259, 1553.33337, -80., 2640.

5260, 1553.33337, -80., 2620.

5261, 1553.33337, -80., 2600.

5262, 1553.33337, -80., 2580.

5263, 1553.33337, -80., 2560.

5264, 1553.33337, -80., 2540.

5265, 1583.58972, -80., 2740.

5266, 1583.58972, -80., 2720.

5267, 1583.58972, -80., 2700.

5268, 1583.58972, -80., 2680.

5269, 1583.58972, -80., 2660.

5270, 1583.58972, -80., 2640.

5271, 1583.58972, -80., 2620.

5272, 1583.58972, -80., 2600.

5273, 1583.58972, -80., 2580.

5274, 1583.58972, -80., 2560.

5275, 1583.58972, -80., 2540.

5276, 1613.84619, -80., 2740.

5277, 1613.84619, -80., 2720.

5278, 1613.84619, -80., 2700.

5279, 1613.84619, -80., 2680.

5280, 1613.84619, -80., 2660.

5281, 1613.84619, -80., 2640.

5282, 1613.84619, -80., 2620.

5283, 1613.84619, -80., 2600.

5284, 1613.84619, -80., 2580.

5285, 1613.84619, -80., 2560.

5286, 1613.84619, -80., 2540.

5287, 1644.10254, -80., 2740.

5288, 1644.10254, -80., 2720.

5289, 1644.10254, -80., 2700.

5290, 1644.10254, -80., 2680.

5291, 1644.10254, -80., 2660.

5292, 1644.10254, -80., 2640.

5293, 1644.10254, -80., 2620.

5294, 1644.10254, -80., 2600.

5295, 1644.10254, -80., 2580.

5296, 1644.10254, -80., 2560.

5297, 1644.10254, -80., 2540.

5298, 1674.35901, -80., 2740.

5299, 1674.35901, -80., 2720.

5300, 1674.35901, -80., 2700.

5301, 1674.35901, -80., 2680.

5302, 1674.35901, -80., 2660.

5303, 1674.35901, -80., 2640.

5304, 1674.35901, -80., 2620.

5305, 1674.35901, -80., 2600.

5306, 1674.35901, -80., 2580.

5307, 1674.35901, -80., 2560.

5308, 1674.35901, -80., 2540.

5309, 1704.61536, -80., 2740.

5310, 1704.61536, -80., 2720.

5311, 1704.61536, -80., 2700.

5312, 1704.61536, -80., 2680.

5313, 1704.61536, -80., 2660.

5314, 1704.61536, -80., 2640.

5315, 1704.61536, -80., 2620.

5316, 1704.61536, -80., 2600.

5317, 1704.61536, -80., 2580.

5318, 1704.61536, -80., 2560.

5319, 1704.61536, -80., 2540.

5320, 1734.87183, -80., 2740.

5321, 1734.87183, -80., 2720.

5322, 1734.87183, -80., 2700.

5323, 1734.87183, -80., 2680.

5324, 1734.87183, -80., 2660.

5325, 1734.87183, -80., 2640.

5326, 1734.87183, -80., 2620.

5327, 1734.87183, -80., 2600.

5328, 1734.87183, -80., 2580.

5329, 1734.87183, -80., 2560.

5330, 1734.87183, -80., 2540.

5331, 1765.12817, -80., 2740.

5332, 1765.12817, -80., 2720.

5333, 1765.12817, -80., 2700.

5334, 1765.12817, -80., 2680.

5335, 1765.12817, -80., 2660.

5336, 1765.12817, -80., 2640.

5337, 1765.12817, -80., 2620.

5338, 1765.12817, -80., 2600.

5339, 1765.12817, -80., 2580.

5340, 1765.12817, -80., 2560.

5341, 1765.12817, -80., 2540.

5342, 1795.38464, -80., 2740.

5343, 1795.38464, -80., 2720.

5344, 1795.38464, -80., 2700.

5345, 1795.38464, -80., 2680.

5346, 1795.38464, -80., 2660.

5347, 1795.38464, -80., 2640.

5348, 1795.38464, -80., 2620.

5349, 1795.38464, -80., 2600.

5350, 1795.38464, -80., 2580.

5351, 1795.38464, -80., 2560.

5352, 1795.38464, -80., 2540.

5353, 1825.64099, -80., 2740.

5354, 1825.64099, -80., 2720.

5355, 1825.64099, -80., 2700.

5356, 1825.64099, -80., 2680.

5357, 1825.64099, -80., 2660.

5358, 1825.64099, -80., 2640.

5359, 1825.64099, -80., 2620.

5360, 1825.64099, -80., 2600.

5361, 1825.64099, -80., 2580.

5362, 1825.64099, -80., 2560.

5363, 1825.64099, -80., 2540.

5364, 1855.89746, -80., 2740.

5365, 1855.89746, -80., 2720.

5366, 1855.89746, -80., 2700.

5367, 1855.89746, -80., 2680.

5368, 1855.89746, -80., 2660.

5369, 1855.89746, -80., 2640.

5370, 1855.89746, -80., 2620.

5371, 1855.89746, -80., 2600.

5372, 1855.89746, -80., 2580.

5373, 1855.89746, -80., 2560.

5374, 1855.89746, -80., 2540.

5375, 1886.15381, -80., 2740.

5376, 1886.15381, -80., 2720.

5377, 1886.15381, -80., 2700.

5378, 1886.15381, -80., 2680.

5379, 1886.15381, -80., 2660.

5380, 1886.15381, -80., 2640.

5381, 1886.15381, -80., 2620.

5382, 1886.15381, -80., 2600.

5383, 1886.15381, -80., 2580.

5384, 1886.15381, -80., 2560.

5385, 1886.15381, -80., 2540.

5386, 1916.41028, -80., 2740.

5387, 1916.41028, -80., 2720.

5388, 1916.41028, -80., 2700.

5389, 1916.41028, -80., 2680.

5390, 1916.41028, -80., 2660.

5391, 1916.41028, -80., 2640.

5392, 1916.41028, -80., 2620.

5393, 1916.41028, -80., 2600.

5394, 1916.41028, -80., 2580.

5395, 1916.41028, -80., 2560.

5396, 1916.41028, -80., 2540.

5397, 1946.66663, -80., 2740.

5398, 1946.66663, -80., 2720.

5399, 1946.66663, -80., 2700.

5400, 1946.66663, -80., 2680.

5401, 1946.66663, -80., 2660.

5402, 1946.66663, -80., 2640.

5403, 1946.66663, -80., 2620.

5404, 1946.66663, -80., 2600.

5405, 1946.66663, -80., 2580.

5406, 1946.66663, -80., 2560.

5407, 1946.66663, -80., 2540.

5408, 1976.9231, -80., 2740.

5409, 1976.9231, -80., 2720.

5410, 1976.9231, -80., 2700.

5411, 1976.9231, -80., 2680.

5412, 1976.9231, -80., 2660.

5413, 1976.9231, -80., 2640.

5414, 1976.9231, -80., 2620.

5415, 1976.9231, -80., 2600.

5416, 1976.9231, -80., 2580.

5417, 1976.9231, -80., 2560.

5418, 1976.9231, -80., 2540.

5419, 2007.17944, -80., 2740.

5420, 2007.17944, -80., 2720.

5421, 2007.17944, -80., 2700.

5422, 2007.17944, -80., 2680.

5423, 2007.17944, -80., 2660.

5424, 2007.17944, -80., 2640.

5425, 2007.17944, -80., 2620.

5426, 2007.17944, -80., 2600.

5427, 2007.17944, -80., 2580.

5428, 2007.17944, -80., 2560.

5429, 2007.17944, -80., 2540.

5430, 2037.43591, -80., 2740.

5431, 2037.43591, -80., 2720.

5432, 2037.43591, -80., 2700.

5433, 2037.43591, -80., 2680.

5434, 2037.43591, -80., 2660.

5435, 2037.43591, -80., 2640.

5436, 2037.43591, -80., 2620.

5437, 2037.43591, -80., 2600.

5438, 2037.43591, -80., 2580.

5439, 2037.43591, -80., 2560.

5440, 2037.43591, -80., 2540.

5441, 2067.69238, -80., 2740.

5442, 2067.69238, -80., 2720.

5443, 2067.69238, -80., 2700.

5444, 2067.69238, -80., 2680.

5445, 2067.69238, -80., 2660.

5446, 2067.69238, -80., 2640.

5447, 2067.69238, -80., 2620.

5448, 2067.69238, -80., 2600.

5449, 2067.69238, -80., 2580.

5450, 2067.69238, -80., 2560.

5451, 2067.69238, -80., 2540.

5452, 2097.94873, -80., 2740.

5453, 2097.94873, -80., 2720.

5454, 2097.94873, -80., 2700.

5455, 2097.94873, -80., 2680.

5456, 2097.94873, -80., 2660.

5457, 2097.94873, -80., 2640.

5458, 2097.94873, -80., 2620.

5459, 2097.94873, -80., 2600.

5460, 2097.94873, -80., 2580.

5461, 2097.94873, -80., 2560.

5462, 2097.94873, -80., 2540.

5463, 2128.20508, -80., 2740.

5464, 2128.20508, -80., 2720.

5465, 2128.20508, -80., 2700.

5466, 2128.20508, -80., 2680.

5467, 2128.20508, -80., 2660.

5468, 2128.20508, -80., 2640.

5469, 2128.20508, -80., 2620.

5470, 2128.20508, -80., 2600.

5471, 2128.20508, -80., 2580.

5472, 2128.20508, -80., 2560.

5473, 2128.20508, -80., 2540.

5474, 2158.46143, -80., 2740.

5475, 2158.46143, -80., 2720.

5476, 2158.46143, -80., 2700.

5477, 2158.46143, -80., 2680.

5478, 2158.46143, -80., 2660.

5479, 2158.46143, -80., 2640.

5480, 2158.46143, -80., 2620.

5481, 2158.46143, -80., 2600.

5482, 2158.46143, -80., 2580.

5483, 2158.46143, -80., 2560.

5484, 2158.46143, -80., 2540.

5485, 2188.71802, -80., 2740.

5486, 2188.71802, -80., 2720.

5487, 2188.71802, -80., 2700.

5488, 2188.71802, -80., 2680.

5489, 2188.71802, -80., 2660.

5490, 2188.71802, -80., 2640.

5491, 2188.71802, -80., 2620.

5492, 2188.71802, -80., 2600.

5493, 2188.71802, -80., 2580.

5494, 2188.71802, -80., 2560.

5495, 2188.71802, -80., 2540.

5496, 2218.97437, -80., 2740.

5497, 2218.97437, -80., 2720.

5498, 2218.97437, -80., 2700.

5499, 2218.97437, -80., 2680.

5500, 2218.97437, -80., 2660.

5501, 2218.97437, -80., 2640.

5502, 2218.97437, -80., 2620.

5503, 2218.97437, -80., 2600.

5504, 2218.97437, -80., 2580.

5505, 2218.97437, -80., 2560.

5506, 2218.97437, -80., 2540.

5507, 2249.23071, -80., 2740.

5508, 2249.23071, -80., 2720.

5509, 2249.23071, -80., 2700.

5510, 2249.23071, -80., 2680.

5511, 2249.23071, -80., 2660.

5512, 2249.23071, -80., 2640.

5513, 2249.23071, -80., 2620.

5514, 2249.23071, -80., 2600.

5515, 2249.23071, -80., 2580.

5516, 2249.23071, -80., 2560.

5517, 2249.23071, -80., 2540.

5518, 2279.48706, -80., 2740.

5519, 2279.48706, -80., 2720.

5520, 2279.48706, -80., 2700.

5521, 2279.48706, -80., 2680.

5522, 2279.48706, -80., 2660.

5523, 2279.48706, -80., 2640.

5524, 2279.48706, -80., 2620.

5525, 2279.48706, -80., 2600.

5526, 2279.48706, -80., 2580.

5527, 2279.48706, -80., 2560.

5528, 2279.48706, -80., 2540.

5529, 2309.74365, -80., 2740.

5530, 2309.74365, -80., 2720.

5531, 2309.74365, -80., 2700.

5532, 2309.74365, -80., 2680.

5533, 2309.74365, -80., 2660.

5534, 2309.74365, -80., 2640.

5535, 2309.74365, -80., 2620.

5536, 2309.74365, -80., 2600.

5537, 2309.74365, -80., 2580.

5538, 2309.74365, -80., 2560.

5539, 2309.74365, -80., 2540.

5540, 1190.25647, -60., 2740.

5541, 1190.25647, -60., 2720.

5542, 1190.25647, -60., 2700.

5543, 1190.25647, -60., 2680.

5544, 1190.25647, -60., 2660.

5545, 1190.25647, -60., 2640.

5546, 1190.25647, -60., 2620.

5547, 1190.25647, -60., 2600.

5548, 1190.25647, -60., 2580.

5549, 1190.25647, -60., 2560.

5550, 1190.25647, -60., 2540.

5551, 1220.51282, -60., 2740.

5552, 1220.51282, -60., 2720.

5553, 1220.51282, -60., 2700.

5554, 1220.51282, -60., 2680.

5555, 1220.51282, -60., 2660.

5556, 1220.51282, -60., 2640.

5557, 1220.51282, -60., 2620.

5558, 1220.51282, -60., 2600.

5559, 1220.51282, -60., 2580.

5560, 1220.51282, -60., 2560.

5561, 1220.51282, -60., 2540.

5562, 1250.76929, -60., 2740.

5563, 1250.76929, -60., 2720.

5564, 1250.76929, -60., 2700.

5565, 1250.76929, -60., 2680.

5566, 1250.76929, -60., 2660.

5567, 1250.76929, -60., 2640.

5568, 1250.76929, -60., 2620.

5569, 1250.76929, -60., 2600.

5570, 1250.76929, -60., 2580.

5571, 1250.76929, -60., 2560.

5572, 1250.76929, -60., 2540.

5573, 1281.02563, -60., 2740.

5574, 1281.02563, -60., 2720.

5575, 1281.02563, -60., 2700.

5576, 1281.02563, -60., 2680.

5577, 1281.02563, -60., 2660.

5578, 1281.02563, -60., 2640.

5579, 1281.02563, -60., 2620.

5580, 1281.02563, -60., 2600.

5581, 1281.02563, -60., 2580.

5582, 1281.02563, -60., 2560.

5583, 1281.02563, -60., 2540.

5584, 1311.2821, -60., 2740.

5585, 1311.2821, -60., 2720.

5586, 1311.2821, -60., 2700.

5587, 1311.2821, -60., 2680.

5588, 1311.2821, -60., 2660.

5589, 1311.2821, -60., 2640.

5590, 1311.2821, -60., 2620.

5591, 1311.2821, -60., 2600.

5592, 1311.2821, -60., 2580.

5593, 1311.2821, -60., 2560.

5594, 1311.2821, -60., 2540.

5595, 1341.53845, -60., 2740.

5596, 1341.53845, -60., 2720.

5597, 1341.53845, -60., 2700.

5598, 1341.53845, -60., 2680.

5599, 1341.53845, -60., 2660.

5600, 1341.53845, -60., 2640.

5601, 1341.53845, -60., 2620.

5602, 1341.53845, -60., 2600.

5603, 1341.53845, -60., 2580.

5604, 1341.53845, -60., 2560.

5605, 1341.53845, -60., 2540.

5606, 1371.79492, -60., 2740.

5607, 1371.79492, -60., 2720.

5608, 1371.79492, -60., 2700.

5609, 1371.79492, -60., 2680.

5610, 1371.79492, -60., 2660.

5611, 1371.79492, -60., 2640.

5612, 1371.79492, -60., 2620.

5613, 1371.79492, -60., 2600.

5614, 1371.79492, -60., 2580.

5615, 1371.79492, -60., 2560.

5616, 1371.79492, -60., 2540.

5617, 1402.05127, -60., 2740.

5618, 1402.05127, -60., 2720.

5619, 1402.05127, -60., 2700.

5620, 1402.05127, -60., 2680.

5621, 1402.05127, -60., 2660.

5622, 1402.05127, -60., 2640.

5623, 1402.05127, -60., 2620.

5624, 1402.05127, -60., 2600.

5625, 1402.05127, -60., 2580.

5626, 1402.05127, -60., 2560.

5627, 1402.05127, -60., 2540.

5628, 1432.30774, -60., 2740.

5629, 1432.30774, -60., 2720.

5630, 1432.30774, -60., 2700.

5631, 1432.30774, -60., 2680.

5632, 1432.30774, -60., 2660.

5633, 1432.30774, -60., 2640.

5634, 1432.30774, -60., 2620.

5635, 1432.30774, -60., 2600.

5636, 1432.30774, -60., 2580.

5637, 1432.30774, -60., 2560.

5638, 1432.30774, -60., 2540.

5639, 1462.56409, -60., 2740.

5640, 1462.56409, -60., 2720.

5641, 1462.56409, -60., 2700.

5642, 1462.56409, -60., 2680.

5643, 1462.56409, -60., 2660.

5644, 1462.56409, -60., 2640.

5645, 1462.56409, -60., 2620.

5646, 1462.56409, -60., 2600.

5647, 1462.56409, -60., 2580.

5648, 1462.56409, -60., 2560.

5649, 1462.56409, -60., 2540.

5650, 1492.82056, -60., 2740.

5651, 1492.82056, -60., 2720.

5652, 1492.82056, -60., 2700.

5653, 1492.82056, -60., 2680.

5654, 1492.82056, -60., 2660.

5655, 1492.82056, -60., 2640.

5656, 1492.82056, -60., 2620.

5657, 1492.82056, -60., 2600.

5658, 1492.82056, -60., 2580.

5659, 1492.82056, -60., 2560.

5660, 1492.82056, -60., 2540.

5661, 1523.0769, -60., 2740.

5662, 1523.0769, -60., 2720.

5663, 1523.0769, -60., 2700.

5664, 1523.0769, -60., 2680.

5665, 1523.0769, -60., 2660.

5666, 1523.0769, -60., 2640.

5667, 1523.0769, -60., 2620.

5668, 1523.0769, -60., 2600.

5669, 1523.0769, -60., 2580.

5670, 1523.0769, -60., 2560.

5671, 1523.0769, -60., 2540.

5672, 1553.33337, -60., 2740.

5673, 1553.33337, -60., 2720.

5674, 1553.33337, -60., 2700.

5675, 1553.33337, -60., 2680.

5676, 1553.33337, -60., 2660.

5677, 1553.33337, -60., 2640.

5678, 1553.33337, -60., 2620.

5679, 1553.33337, -60., 2600.

5680, 1553.33337, -60., 2580.

5681, 1553.33337, -60., 2560.

5682, 1553.33337, -60., 2540.

5683, 1583.58972, -60., 2740.

5684, 1583.58972, -60., 2720.

5685, 1583.58972, -60., 2700.

5686, 1583.58972, -60., 2680.

5687, 1583.58972, -60., 2660.

5688, 1583.58972, -60., 2640.

5689, 1583.58972, -60., 2620.

5690, 1583.58972, -60., 2600.

5691, 1583.58972, -60., 2580.

5692, 1583.58972, -60., 2560.

5693, 1583.58972, -60., 2540.

5694, 1613.84619, -60., 2740.

5695, 1613.84619, -60., 2720.

5696, 1613.84619, -60., 2700.

5697, 1613.84619, -60., 2680.

5698, 1613.84619, -60., 2660.

5699, 1613.84619, -60., 2640.

5700, 1613.84619, -60., 2620.

5701, 1613.84619, -60., 2600.

5702, 1613.84619, -60., 2580.

5703, 1613.84619, -60., 2560.

5704, 1613.84619, -60., 2540.

5705, 1644.10254, -60., 2740.

5706, 1644.10254, -60., 2720.

5707, 1644.10254, -60., 2700.

5708, 1644.10254, -60., 2680.

5709, 1644.10254, -60., 2660.

5710, 1644.10254, -60., 2640.

5711, 1644.10254, -60., 2620.

5712, 1644.10254, -60., 2600.

5713, 1644.10254, -60., 2580.

5714, 1644.10254, -60., 2560.

5715, 1644.10254, -60., 2540.

5716, 1674.35901, -60., 2740.

5717, 1674.35901, -60., 2720.

5718, 1674.35901, -60., 2700.

5719, 1674.35901, -60., 2680.

5720, 1674.35901, -60., 2660.

5721, 1674.35901, -60., 2640.

5722, 1674.35901, -60., 2620.

5723, 1674.35901, -60., 2600.

5724, 1674.35901, -60., 2580.

5725, 1674.35901, -60., 2560.

5726, 1674.35901, -60., 2540.

5727, 1704.61536, -60., 2740.

5728, 1704.61536, -60., 2720.

5729, 1704.61536, -60., 2700.

5730, 1704.61536, -60., 2680.

5731, 1704.61536, -60., 2660.

5732, 1704.61536, -60., 2640.

5733, 1704.61536, -60., 2620.

5734, 1704.61536, -60., 2600.

5735, 1704.61536, -60., 2580.

5736, 1704.61536, -60., 2560.

5737, 1704.61536, -60., 2540.

5738, 1734.87183, -60., 2740.

5739, 1734.87183, -60., 2720.

5740, 1734.87183, -60., 2700.

5741, 1734.87183, -60., 2680.

5742, 1734.87183, -60., 2660.

5743, 1734.87183, -60., 2640.

5744, 1734.87183, -60., 2620.

5745, 1734.87183, -60., 2600.

5746, 1734.87183, -60., 2580.

5747, 1734.87183, -60., 2560.

5748, 1734.87183, -60., 2540.

5749, 1765.12817, -60., 2740.

5750, 1765.12817, -60., 2720.

5751, 1765.12817, -60., 2700.

5752, 1765.12817, -60., 2680.

5753, 1765.12817, -60., 2660.

5754, 1765.12817, -60., 2640.

5755, 1765.12817, -60., 2620.

5756, 1765.12817, -60., 2600.

5757, 1765.12817, -60., 2580.

5758, 1765.12817, -60., 2560.

5759, 1765.12817, -60., 2540.

5760, 1795.38464, -60., 2740.

5761, 1795.38464, -60., 2720.

5762, 1795.38464, -60., 2700.

5763, 1795.38464, -60., 2680.

5764, 1795.38464, -60., 2660.

5765, 1795.38464, -60., 2640.

5766, 1795.38464, -60., 2620.

5767, 1795.38464, -60., 2600.

5768, 1795.38464, -60., 2580.

5769, 1795.38464, -60., 2560.

5770, 1795.38464, -60., 2540.

5771, 1825.64099, -60., 2740.

5772, 1825.64099, -60., 2720.

5773, 1825.64099, -60., 2700.

5774, 1825.64099, -60., 2680.

5775, 1825.64099, -60., 2660.

5776, 1825.64099, -60., 2640.

5777, 1825.64099, -60., 2620.

5778, 1825.64099, -60., 2600.

5779, 1825.64099, -60., 2580.

5780, 1825.64099, -60., 2560.

5781, 1825.64099, -60., 2540.

5782, 1855.89746, -60., 2740.

5783, 1855.89746, -60., 2720.

5784, 1855.89746, -60., 2700.

5785, 1855.89746, -60., 2680.

5786, 1855.89746, -60., 2660.

5787, 1855.89746, -60., 2640.

5788, 1855.89746, -60., 2620.

5789, 1855.89746, -60., 2600.

5790, 1855.89746, -60., 2580.

5791, 1855.89746, -60., 2560.

5792, 1855.89746, -60., 2540.

5793, 1886.15381, -60., 2740.

5794, 1886.15381, -60., 2720.

5795, 1886.15381, -60., 2700.

5796, 1886.15381, -60., 2680.

5797, 1886.15381, -60., 2660.

5798, 1886.15381, -60., 2640.

5799, 1886.15381, -60., 2620.

5800, 1886.15381, -60., 2600.

5801, 1886.15381, -60., 2580.

5802, 1886.15381, -60., 2560.

5803, 1886.15381, -60., 2540.

5804, 1916.41028, -60., 2740.

5805, 1916.41028, -60., 2720.

5806, 1916.41028, -60., 2700.

5807, 1916.41028, -60., 2680.

5808, 1916.41028, -60., 2660.

5809, 1916.41028, -60., 2640.

5810, 1916.41028, -60., 2620.

5811, 1916.41028, -60., 2600.

5812, 1916.41028, -60., 2580.

5813, 1916.41028, -60., 2560.

5814, 1916.41028, -60., 2540.

5815, 1946.66663, -60., 2740.

5816, 1946.66663, -60., 2720.

5817, 1946.66663, -60., 2700.

5818, 1946.66663, -60., 2680.

5819, 1946.66663, -60., 2660.

5820, 1946.66663, -60., 2640.

5821, 1946.66663, -60., 2620.

5822, 1946.66663, -60., 2600.

5823, 1946.66663, -60., 2580.

5824, 1946.66663, -60., 2560.

5825, 1946.66663, -60., 2540.

5826, 1976.9231, -60., 2740.

5827, 1976.9231, -60., 2720.

5828, 1976.9231, -60., 2700.

5829, 1976.9231, -60., 2680.

5830, 1976.9231, -60., 2660.

5831, 1976.9231, -60., 2640.

5832, 1976.9231, -60., 2620.

5833, 1976.9231, -60., 2600.

5834, 1976.9231, -60., 2580.

5835, 1976.9231, -60., 2560.

5836, 1976.9231, -60., 2540.

5837, 2007.17944, -60., 2740.

5838, 2007.17944, -60., 2720.

5839, 2007.17944, -60., 2700.

5840, 2007.17944, -60., 2680.

5841, 2007.17944, -60., 2660.

5842, 2007.17944, -60., 2640.

5843, 2007.17944, -60., 2620.

5844, 2007.17944, -60., 2600.

5845, 2007.17944, -60., 2580.

5846, 2007.17944, -60., 2560.

5847, 2007.17944, -60., 2540.

5848, 2037.43591, -60., 2740.

5849, 2037.43591, -60., 2720.

5850, 2037.43591, -60., 2700.

5851, 2037.43591, -60., 2680.

5852, 2037.43591, -60., 2660.

5853, 2037.43591, -60., 2640.

5854, 2037.43591, -60., 2620.

5855, 2037.43591, -60., 2600.

5856, 2037.43591, -60., 2580.

5857, 2037.43591, -60., 2560.

5858, 2037.43591, -60., 2540.

5859, 2067.69238, -60., 2740.

5860, 2067.69238, -60., 2720.

5861, 2067.69238, -60., 2700.

5862, 2067.69238, -60., 2680.

5863, 2067.69238, -60., 2660.

5864, 2067.69238, -60., 2640.

5865, 2067.69238, -60., 2620.

5866, 2067.69238, -60., 2600.

5867, 2067.69238, -60., 2580.

5868, 2067.69238, -60., 2560.

5869, 2067.69238, -60., 2540.

5870, 2097.94873, -60., 2740.

5871, 2097.94873, -60., 2720.

5872, 2097.94873, -60., 2700.

5873, 2097.94873, -60., 2680.

5874, 2097.94873, -60., 2660.

5875, 2097.94873, -60., 2640.

5876, 2097.94873, -60., 2620.

5877, 2097.94873, -60., 2600.

5878, 2097.94873, -60., 2580.

5879, 2097.94873, -60., 2560.

5880, 2097.94873, -60., 2540.

5881, 2128.20508, -60., 2740.

5882, 2128.20508, -60., 2720.

5883, 2128.20508, -60., 2700.

5884, 2128.20508, -60., 2680.

5885, 2128.20508, -60., 2660.

5886, 2128.20508, -60., 2640.

5887, 2128.20508, -60., 2620.

5888, 2128.20508, -60., 2600.

5889, 2128.20508, -60., 2580.

5890, 2128.20508, -60., 2560.

5891, 2128.20508, -60., 2540.

5892, 2158.46143, -60., 2740.

5893, 2158.46143, -60., 2720.

5894, 2158.46143, -60., 2700.

5895, 2158.46143, -60., 2680.

5896, 2158.46143, -60., 2660.

5897, 2158.46143, -60., 2640.

5898, 2158.46143, -60., 2620.

5899, 2158.46143, -60., 2600.

5900, 2158.46143, -60., 2580.

5901, 2158.46143, -60., 2560.

5902, 2158.46143, -60., 2540.

5903, 2188.71802, -60., 2740.

5904, 2188.71802, -60., 2720.

5905, 2188.71802, -60., 2700.

5906, 2188.71802, -60., 2680.

5907, 2188.71802, -60., 2660.

5908, 2188.71802, -60., 2640.

5909, 2188.71802, -60., 2620.

5910, 2188.71802, -60., 2600.

5911, 2188.71802, -60., 2580.

5912, 2188.71802, -60., 2560.

5913, 2188.71802, -60., 2540.

5914, 2218.97437, -60., 2740.

5915, 2218.97437, -60., 2720.

5916, 2218.97437, -60., 2700.

5917, 2218.97437, -60., 2680.

5918, 2218.97437, -60., 2660.

5919, 2218.97437, -60., 2640.

5920, 2218.97437, -60., 2620.

5921, 2218.97437, -60., 2600.

5922, 2218.97437, -60., 2580.

5923, 2218.97437, -60., 2560.

5924, 2218.97437, -60., 2540.

5925, 2249.23071, -60., 2740.

5926, 2249.23071, -60., 2720.

5927, 2249.23071, -60., 2700.

5928, 2249.23071, -60., 2680.

5929, 2249.23071, -60., 2660.

5930, 2249.23071, -60., 2640.

5931, 2249.23071, -60., 2620.

5932, 2249.23071, -60., 2600.

5933, 2249.23071, -60., 2580.

5934, 2249.23071, -60., 2560.

5935, 2249.23071, -60., 2540.

5936, 2279.48706, -60., 2740.

5937, 2279.48706, -60., 2720.

5938, 2279.48706, -60., 2700.

5939, 2279.48706, -60., 2680.

5940, 2279.48706, -60., 2660.

5941, 2279.48706, -60., 2640.

5942, 2279.48706, -60., 2620.

5943, 2279.48706, -60., 2600.

5944, 2279.48706, -60., 2580.

5945, 2279.48706, -60., 2560.

5946, 2279.48706, -60., 2540.

5947, 2309.74365, -60., 2740.

5948, 2309.74365, -60., 2720.

5949, 2309.74365, -60., 2700.

5950, 2309.74365, -60., 2680.

5951, 2309.74365, -60., 2660.

5952, 2309.74365, -60., 2640.

5953, 2309.74365, -60., 2620.

5954, 2309.74365, -60., 2600.

5955, 2309.74365, -60., 2580.

5956, 2309.74365, -60., 2560.

5957, 2309.74365, -60., 2540.

5958, 1190.25647, -40., 2740.

5959, 1190.25647, -40., 2720.

5960, 1190.25647, -40., 2700.

5961, 1190.25647, -40., 2680.

5962, 1190.25647, -40., 2660.

5963, 1190.25647, -40., 2640.

5964, 1190.25647, -40., 2620.

5965, 1190.25647, -40., 2600.

5966, 1190.25647, -40., 2580.

5967, 1190.25647, -40., 2560.

5968, 1190.25647, -40., 2540.

5969, 1220.51282, -40., 2740.

5970, 1220.51282, -40., 2720.

5971, 1220.51282, -40., 2700.

5972, 1220.51282, -40., 2680.

5973, 1220.51282, -40., 2660.

5974, 1220.51282, -40., 2640.

5975, 1220.51282, -40., 2620.

5976, 1220.51282, -40., 2600.

5977, 1220.51282, -40., 2580.

5978, 1220.51282, -40., 2560.

5979, 1220.51282, -40., 2540.

5980, 1250.76929, -40., 2740.

5981, 1250.76929, -40., 2720.

5982, 1250.76929, -40., 2700.

5983, 1250.76929, -40., 2680.

5984, 1250.76929, -40., 2660.

5985, 1250.76929, -40., 2640.

5986, 1250.76929, -40., 2620.

5987, 1250.76929, -40., 2600.

5988, 1250.76929, -40., 2580.

5989, 1250.76929, -40., 2560.

5990, 1250.76929, -40., 2540.

5991, 1281.02563, -40., 2740.

5992, 1281.02563, -40., 2720.

5993, 1281.02563, -40., 2700.

5994, 1281.02563, -40., 2680.

5995, 1281.02563, -40., 2660.

5996, 1281.02563, -40., 2640.

5997, 1281.02563, -40., 2620.

5998, 1281.02563, -40., 2600.

5999, 1281.02563, -40., 2580.

6000, 1281.02563, -40., 2560.

6001, 1281.02563, -40., 2540.

6002, 1311.2821, -40., 2740.

6003, 1311.2821, -40., 2720.

6004, 1311.2821, -40., 2700.

6005, 1311.2821, -40., 2680.

6006, 1311.2821, -40., 2660.

6007, 1311.2821, -40., 2640.

6008, 1311.2821, -40., 2620.

6009, 1311.2821, -40., 2600.

6010, 1311.2821, -40., 2580.

6011, 1311.2821, -40., 2560.

6012, 1311.2821, -40., 2540.

6013, 1341.53845, -40., 2740.

6014, 1341.53845, -40., 2720.

6015, 1341.53845, -40., 2700.

6016, 1341.53845, -40., 2680.

6017, 1341.53845, -40., 2660.

6018, 1341.53845, -40., 2640.

6019, 1341.53845, -40., 2620.

6020, 1341.53845, -40., 2600.

6021, 1341.53845, -40., 2580.

6022, 1341.53845, -40., 2560.

6023, 1341.53845, -40., 2540.

6024, 1371.79492, -40., 2740.

6025, 1371.79492, -40., 2720.

6026, 1371.79492, -40., 2700.

6027, 1371.79492, -40., 2680.

6028, 1371.79492, -40., 2660.

6029, 1371.79492, -40., 2640.

6030, 1371.79492, -40., 2620.

6031, 1371.79492, -40., 2600.

6032, 1371.79492, -40., 2580.

6033, 1371.79492, -40., 2560.

6034, 1371.79492, -40., 2540.

6035, 1402.05127, -40., 2740.

6036, 1402.05127, -40., 2720.

6037, 1402.05127, -40., 2700.

6038, 1402.05127, -40., 2680.

6039, 1402.05127, -40., 2660.

6040, 1402.05127, -40., 2640.

6041, 1402.05127, -40., 2620.

6042, 1402.05127, -40., 2600.

6043, 1402.05127, -40., 2580.

6044, 1402.05127, -40., 2560.

6045, 1402.05127, -40., 2540.

6046, 1432.30774, -40., 2740.

6047, 1432.30774, -40., 2720.

6048, 1432.30774, -40., 2700.

6049, 1432.30774, -40., 2680.

6050, 1432.30774, -40., 2660.

6051, 1432.30774, -40., 2640.

6052, 1432.30774, -40., 2620.

6053, 1432.30774, -40., 2600.

6054, 1432.30774, -40., 2580.

6055, 1432.30774, -40., 2560.

6056, 1432.30774, -40., 2540.

6057, 1462.56409, -40., 2740.

6058, 1462.56409, -40., 2720.

6059, 1462.56409, -40., 2700.

6060, 1462.56409, -40., 2680.

6061, 1462.56409, -40., 2660.

6062, 1462.56409, -40., 2640.

6063, 1462.56409, -40., 2620.

6064, 1462.56409, -40., 2600.

6065, 1462.56409, -40., 2580.

6066, 1462.56409, -40., 2560.

6067, 1462.56409, -40., 2540.

6068, 1492.82056, -40., 2740.

6069, 1492.82056, -40., 2720.

6070, 1492.82056, -40., 2700.

6071, 1492.82056, -40., 2680.

6072, 1492.82056, -40., 2660.

6073, 1492.82056, -40., 2640.

6074, 1492.82056, -40., 2620.

6075, 1492.82056, -40., 2600.

6076, 1492.82056, -40., 2580.

6077, 1492.82056, -40., 2560.

6078, 1492.82056, -40., 2540.

6079, 1523.0769, -40., 2740.

6080, 1523.0769, -40., 2720.

6081, 1523.0769, -40., 2700.

6082, 1523.0769, -40., 2680.

6083, 1523.0769, -40., 2660.

6084, 1523.0769, -40., 2640.

6085, 1523.0769, -40., 2620.

6086, 1523.0769, -40., 2600.

6087, 1523.0769, -40., 2580.

6088, 1523.0769, -40., 2560.

6089, 1523.0769, -40., 2540.

6090, 1553.33337, -40., 2740.

6091, 1553.33337, -40., 2720.

6092, 1553.33337, -40., 2700.

6093, 1553.33337, -40., 2680.

6094, 1553.33337, -40., 2660.

6095, 1553.33337, -40., 2640.

6096, 1553.33337, -40., 2620.

6097, 1553.33337, -40., 2600.

6098, 1553.33337, -40., 2580.

6099, 1553.33337, -40., 2560.

6100, 1553.33337, -40., 2540.

6101, 1583.58972, -40., 2740.

6102, 1583.58972, -40., 2720.

6103, 1583.58972, -40., 2700.

6104, 1583.58972, -40., 2680.

6105, 1583.58972, -40., 2660.

6106, 1583.58972, -40., 2640.

6107, 1583.58972, -40., 2620.

6108, 1583.58972, -40., 2600.

6109, 1583.58972, -40., 2580.

6110, 1583.58972, -40., 2560.

6111, 1583.58972, -40., 2540.

6112, 1613.84619, -40., 2740.

6113, 1613.84619, -40., 2720.

6114, 1613.84619, -40., 2700.

6115, 1613.84619, -40., 2680.

6116, 1613.84619, -40., 2660.

6117, 1613.84619, -40., 2640.

6118, 1613.84619, -40., 2620.

6119, 1613.84619, -40., 2600.

6120, 1613.84619, -40., 2580.

6121, 1613.84619, -40., 2560.

6122, 1613.84619, -40., 2540.

6123, 1644.10254, -40., 2740.

6124, 1644.10254, -40., 2720.

6125, 1644.10254, -40., 2700.

6126, 1644.10254, -40., 2680.

6127, 1644.10254, -40., 2660.

6128, 1644.10254, -40., 2640.

6129, 1644.10254, -40., 2620.

6130, 1644.10254, -40., 2600.

6131, 1644.10254, -40., 2580.

6132, 1644.10254, -40., 2560.

6133, 1644.10254, -40., 2540.

6134, 1674.35901, -40., 2740.

6135, 1674.35901, -40., 2720.

6136, 1674.35901, -40., 2700.

6137, 1674.35901, -40., 2680.

6138, 1674.35901, -40., 2660.

6139, 1674.35901, -40., 2640.

6140, 1674.35901, -40., 2620.

6141, 1674.35901, -40., 2600.

6142, 1674.35901, -40., 2580.

6143, 1674.35901, -40., 2560.

6144, 1674.35901, -40., 2540.

6145, 1704.61536, -40., 2740.

6146, 1704.61536, -40., 2720.

6147, 1704.61536, -40., 2700.

6148, 1704.61536, -40., 2680.

6149, 1704.61536, -40., 2660.

6150, 1704.61536, -40., 2640.

6151, 1704.61536, -40., 2620.

6152, 1704.61536, -40., 2600.

6153, 1704.61536, -40., 2580.

6154, 1704.61536, -40., 2560.

6155, 1704.61536, -40., 2540.

6156, 1734.87183, -40., 2740.

6157, 1734.87183, -40., 2720.

6158, 1734.87183, -40., 2700.

6159, 1734.87183, -40., 2680.

6160, 1734.87183, -40., 2660.

6161, 1734.87183, -40., 2640.

6162, 1734.87183, -40., 2620.

6163, 1734.87183, -40., 2600.

6164, 1734.87183, -40., 2580.

6165, 1734.87183, -40., 2560.

6166, 1734.87183, -40., 2540.

6167, 1765.12817, -40., 2740.

6168, 1765.12817, -40., 2720.

6169, 1765.12817, -40., 2700.

6170, 1765.12817, -40., 2680.

6171, 1765.12817, -40., 2660.

6172, 1765.12817, -40., 2640.

6173, 1765.12817, -40., 2620.

6174, 1765.12817, -40., 2600.

6175, 1765.12817, -40., 2580.

6176, 1765.12817, -40., 2560.

6177, 1765.12817, -40., 2540.

6178, 1795.38464, -40., 2740.

6179, 1795.38464, -40., 2720.

6180, 1795.38464, -40., 2700.

6181, 1795.38464, -40., 2680.

6182, 1795.38464, -40., 2660.

6183, 1795.38464, -40., 2640.

6184, 1795.38464, -40., 2620.

6185, 1795.38464, -40., 2600.

6186, 1795.38464, -40., 2580.

6187, 1795.38464, -40., 2560.

6188, 1795.38464, -40., 2540.

6189, 1825.64099, -40., 2740.

6190, 1825.64099, -40., 2720.

6191, 1825.64099, -40., 2700.

6192, 1825.64099, -40., 2680.

6193, 1825.64099, -40., 2660.

6194, 1825.64099, -40., 2640.

6195, 1825.64099, -40., 2620.

6196, 1825.64099, -40., 2600.

6197, 1825.64099, -40., 2580.

6198, 1825.64099, -40., 2560.

6199, 1825.64099, -40., 2540.

6200, 1855.89746, -40., 2740.

6201, 1855.89746, -40., 2720.

6202, 1855.89746, -40., 2700.

6203, 1855.89746, -40., 2680.

6204, 1855.89746, -40., 2660.

6205, 1855.89746, -40., 2640.

6206, 1855.89746, -40., 2620.

6207, 1855.89746, -40., 2600.

6208, 1855.89746, -40., 2580.

6209, 1855.89746, -40., 2560.

6210, 1855.89746, -40., 2540.

6211, 1886.15381, -40., 2740.

6212, 1886.15381, -40., 2720.

6213, 1886.15381, -40., 2700.

6214, 1886.15381, -40., 2680.

6215, 1886.15381, -40., 2660.

6216, 1886.15381, -40., 2640.

6217, 1886.15381, -40., 2620.

6218, 1886.15381, -40., 2600.

6219, 1886.15381, -40., 2580.

6220, 1886.15381, -40., 2560.

6221, 1886.15381, -40., 2540.

6222, 1916.41028, -40., 2740.

6223, 1916.41028, -40., 2720.

6224, 1916.41028, -40., 2700.

6225, 1916.41028, -40., 2680.

6226, 1916.41028, -40., 2660.

6227, 1916.41028, -40., 2640.

6228, 1916.41028, -40., 2620.

6229, 1916.41028, -40., 2600.

6230, 1916.41028, -40., 2580.

6231, 1916.41028, -40., 2560.

6232, 1916.41028, -40., 2540.

6233, 1946.66663, -40., 2740.

6234, 1946.66663, -40., 2720.

6235, 1946.66663, -40., 2700.

6236, 1946.66663, -40., 2680.

6237, 1946.66663, -40., 2660.

6238, 1946.66663, -40., 2640.

6239, 1946.66663, -40., 2620.

6240, 1946.66663, -40., 2600.

6241, 1946.66663, -40., 2580.

6242, 1946.66663, -40., 2560.

6243, 1946.66663, -40., 2540.

6244, 1976.9231, -40., 2740.

6245, 1976.9231, -40., 2720.

6246, 1976.9231, -40., 2700.

6247, 1976.9231, -40., 2680.

6248, 1976.9231, -40., 2660.

6249, 1976.9231, -40., 2640.

6250, 1976.9231, -40., 2620.

6251, 1976.9231, -40., 2600.

6252, 1976.9231, -40., 2580.

6253, 1976.9231, -40., 2560.

6254, 1976.9231, -40., 2540.

6255, 2007.17944, -40., 2740.

6256, 2007.17944, -40., 2720.

6257, 2007.17944, -40., 2700.

6258, 2007.17944, -40., 2680.

6259, 2007.17944, -40., 2660.

6260, 2007.17944, -40., 2640.

6261, 2007.17944, -40., 2620.

6262, 2007.17944, -40., 2600.

6263, 2007.17944, -40., 2580.

6264, 2007.17944, -40., 2560.

6265, 2007.17944, -40., 2540.

6266, 2037.43591, -40., 2740.

6267, 2037.43591, -40., 2720.

6268, 2037.43591, -40., 2700.

6269, 2037.43591, -40., 2680.

6270, 2037.43591, -40., 2660.

6271, 2037.43591, -40., 2640.

6272, 2037.43591, -40., 2620.

6273, 2037.43591, -40., 2600.

6274, 2037.43591, -40., 2580.

6275, 2037.43591, -40., 2560.

6276, 2037.43591, -40., 2540.

6277, 2067.69238, -40., 2740.

6278, 2067.69238, -40., 2720.

6279, 2067.69238, -40., 2700.

6280, 2067.69238, -40., 2680.

6281, 2067.69238, -40., 2660.

6282, 2067.69238, -40., 2640.

6283, 2067.69238, -40., 2620.

6284, 2067.69238, -40., 2600.

6285, 2067.69238, -40., 2580.

6286, 2067.69238, -40., 2560.

6287, 2067.69238, -40., 2540.

6288, 2097.94873, -40., 2740.

6289, 2097.94873, -40., 2720.

6290, 2097.94873, -40., 2700.

6291, 2097.94873, -40., 2680.

6292, 2097.94873, -40., 2660.

6293, 2097.94873, -40., 2640.

6294, 2097.94873, -40., 2620.

6295, 2097.94873, -40., 2600.

6296, 2097.94873, -40., 2580.

6297, 2097.94873, -40., 2560.

6298, 2097.94873, -40., 2540.

6299, 2128.20508, -40., 2740.

6300, 2128.20508, -40., 2720.

6301, 2128.20508, -40., 2700.

6302, 2128.20508, -40., 2680.

6303, 2128.20508, -40., 2660.

6304, 2128.20508, -40., 2640.

6305, 2128.20508, -40., 2620.

6306, 2128.20508, -40., 2600.

6307, 2128.20508, -40., 2580.

6308, 2128.20508, -40., 2560.

6309, 2128.20508, -40., 2540.

6310, 2158.46143, -40., 2740.

6311, 2158.46143, -40., 2720.

6312, 2158.46143, -40., 2700.

6313, 2158.46143, -40., 2680.

6314, 2158.46143, -40., 2660.

6315, 2158.46143, -40., 2640.

6316, 2158.46143, -40., 2620.

6317, 2158.46143, -40., 2600.

6318, 2158.46143, -40., 2580.

6319, 2158.46143, -40., 2560.

6320, 2158.46143, -40., 2540.

6321, 2188.71802, -40., 2740.

6322, 2188.71802, -40., 2720.

6323, 2188.71802, -40., 2700.

6324, 2188.71802, -40., 2680.

6325, 2188.71802, -40., 2660.

6326, 2188.71802, -40., 2640.

6327, 2188.71802, -40., 2620.

6328, 2188.71802, -40., 2600.

6329, 2188.71802, -40., 2580.

6330, 2188.71802, -40., 2560.

6331, 2188.71802, -40., 2540.

6332, 2218.97437, -40., 2740.

6333, 2218.97437, -40., 2720.

6334, 2218.97437, -40., 2700.

6335, 2218.97437, -40., 2680.

6336, 2218.97437, -40., 2660.

6337, 2218.97437, -40., 2640.

6338, 2218.97437, -40., 2620.

6339, 2218.97437, -40., 2600.

6340, 2218.97437, -40., 2580.

6341, 2218.97437, -40., 2560.

6342, 2218.97437, -40., 2540.

6343, 2249.23071, -40., 2740.

6344, 2249.23071, -40., 2720.

6345, 2249.23071, -40., 2700.

6346, 2249.23071, -40., 2680.

6347, 2249.23071, -40., 2660.

6348, 2249.23071, -40., 2640.

6349, 2249.23071, -40., 2620.

6350, 2249.23071, -40., 2600.

6351, 2249.23071, -40., 2580.

6352, 2249.23071, -40., 2560.

6353, 2249.23071, -40., 2540.

6354, 2279.48706, -40., 2740.

6355, 2279.48706, -40., 2720.

6356, 2279.48706, -40., 2700.

6357, 2279.48706, -40., 2680.

6358, 2279.48706, -40., 2660.

6359, 2279.48706, -40., 2640.

6360, 2279.48706, -40., 2620.

6361, 2279.48706, -40., 2600.

6362, 2279.48706, -40., 2580.

6363, 2279.48706, -40., 2560.

6364, 2279.48706, -40., 2540.

6365, 2309.74365, -40., 2740.

6366, 2309.74365, -40., 2720.

6367, 2309.74365, -40., 2700.

6368, 2309.74365, -40., 2680.

6369, 2309.74365, -40., 2660.

6370, 2309.74365, -40., 2640.

6371, 2309.74365, -40., 2620.

6372, 2309.74365, -40., 2600.

6373, 2309.74365, -40., 2580.

6374, 2309.74365, -40., 2560.

6375, 2309.74365, -40., 2540.

6376, 1190.25647, -20., 2740.

6377, 1190.25647, -20., 2720.

6378, 1190.25647, -20., 2700.

6379, 1190.25647, -20., 2680.

6380, 1190.25647, -20., 2660.

6381, 1190.25647, -20., 2640.

6382, 1190.25647, -20., 2620.

6383, 1190.25647, -20., 2600.

6384, 1190.25647, -20., 2580.

6385, 1190.25647, -20., 2560.

6386, 1190.25647, -20., 2540.

6387, 1220.51282, -20., 2740.

6388, 1220.51282, -20., 2720.

6389, 1220.51282, -20., 2700.

6390, 1220.51282, -20., 2680.

6391, 1220.51282, -20., 2660.

6392, 1220.51282, -20., 2640.

6393, 1220.51282, -20., 2620.

6394, 1220.51282, -20., 2600.

6395, 1220.51282, -20., 2580.

6396, 1220.51282, -20., 2560.

6397, 1220.51282, -20., 2540.

6398, 1250.76929, -20., 2740.

6399, 1250.76929, -20., 2720.

6400, 1250.76929, -20., 2700.

6401, 1250.76929, -20., 2680.

6402, 1250.76929, -20., 2660.

6403, 1250.76929, -20., 2640.

6404, 1250.76929, -20., 2620.

6405, 1250.76929, -20., 2600.

6406, 1250.76929, -20., 2580.

6407, 1250.76929, -20., 2560.

6408, 1250.76929, -20., 2540.

6409, 1281.02563, -20., 2740.

6410, 1281.02563, -20., 2720.

6411, 1281.02563, -20., 2700.

6412, 1281.02563, -20., 2680.

6413, 1281.02563, -20., 2660.

6414, 1281.02563, -20., 2640.

6415, 1281.02563, -20., 2620.

6416, 1281.02563, -20., 2600.

6417, 1281.02563, -20., 2580.

6418, 1281.02563, -20., 2560.

6419, 1281.02563, -20., 2540.

6420, 1311.2821, -20., 2740.

6421, 1311.2821, -20., 2720.

6422, 1311.2821, -20., 2700.

6423, 1311.2821, -20., 2680.

6424, 1311.2821, -20., 2660.

6425, 1311.2821, -20., 2640.

6426, 1311.2821, -20., 2620.

6427, 1311.2821, -20., 2600.

6428, 1311.2821, -20., 2580.

6429, 1311.2821, -20., 2560.

6430, 1311.2821, -20., 2540.

6431, 1341.53845, -20., 2740.

6432, 1341.53845, -20., 2720.

6433, 1341.53845, -20., 2700.

6434, 1341.53845, -20., 2680.

6435, 1341.53845, -20., 2660.

6436, 1341.53845, -20., 2640.

6437, 1341.53845, -20., 2620.

6438, 1341.53845, -20., 2600.

6439, 1341.53845, -20., 2580.

6440, 1341.53845, -20., 2560.

6441, 1341.53845, -20., 2540.

6442, 1371.79492, -20., 2740.

6443, 1371.79492, -20., 2720.

6444, 1371.79492, -20., 2700.

6445, 1371.79492, -20., 2680.

6446, 1371.79492, -20., 2660.

6447, 1371.79492, -20., 2640.

6448, 1371.79492, -20., 2620.

6449, 1371.79492, -20., 2600.

6450, 1371.79492, -20., 2580.

6451, 1371.79492, -20., 2560.

6452, 1371.79492, -20., 2540.

6453, 1402.05127, -20., 2740.

6454, 1402.05127, -20., 2720.

6455, 1402.05127, -20., 2700.

6456, 1402.05127, -20., 2680.

6457, 1402.05127, -20., 2660.

6458, 1402.05127, -20., 2640.

6459, 1402.05127, -20., 2620.

6460, 1402.05127, -20., 2600.

6461, 1402.05127, -20., 2580.

6462, 1402.05127, -20., 2560.

6463, 1402.05127, -20., 2540.

6464, 1432.30774, -20., 2740.

6465, 1432.30774, -20., 2720.

6466, 1432.30774, -20., 2700.

6467, 1432.30774, -20., 2680.

6468, 1432.30774, -20., 2660.

6469, 1432.30774, -20., 2640.

6470, 1432.30774, -20., 2620.

6471, 1432.30774, -20., 2600.

6472, 1432.30774, -20., 2580.

6473, 1432.30774, -20., 2560.

6474, 1432.30774, -20., 2540.

6475, 1462.56409, -20., 2740.

6476, 1462.56409, -20., 2720.

6477, 1462.56409, -20., 2700.

6478, 1462.56409, -20., 2680.

6479, 1462.56409, -20., 2660.

6480, 1462.56409, -20., 2640.

6481, 1462.56409, -20., 2620.

6482, 1462.56409, -20., 2600.

6483, 1462.56409, -20., 2580.

6484, 1462.56409, -20., 2560.

6485, 1462.56409, -20., 2540.

6486, 1492.82056, -20., 2740.

6487, 1492.82056, -20., 2720.

6488, 1492.82056, -20., 2700.

6489, 1492.82056, -20., 2680.

6490, 1492.82056, -20., 2660.

6491, 1492.82056, -20., 2640.

6492, 1492.82056, -20., 2620.

6493, 1492.82056, -20., 2600.

6494, 1492.82056, -20., 2580.

6495, 1492.82056, -20., 2560.

6496, 1492.82056, -20., 2540.

6497, 1523.0769, -20., 2740.

6498, 1523.0769, -20., 2720.

6499, 1523.0769, -20., 2700.

6500, 1523.0769, -20., 2680.

6501, 1523.0769, -20., 2660.

6502, 1523.0769, -20., 2640.

6503, 1523.0769, -20., 2620.

6504, 1523.0769, -20., 2600.

6505, 1523.0769, -20., 2580.

6506, 1523.0769, -20., 2560.

6507, 1523.0769, -20., 2540.

6508, 1553.33337, -20., 2740.

6509, 1553.33337, -20., 2720.

6510, 1553.33337, -20., 2700.

6511, 1553.33337, -20., 2680.

6512, 1553.33337, -20., 2660.

6513, 1553.33337, -20., 2640.

6514, 1553.33337, -20., 2620.

6515, 1553.33337, -20., 2600.

6516, 1553.33337, -20., 2580.

6517, 1553.33337, -20., 2560.

6518, 1553.33337, -20., 2540.

6519, 1583.58972, -20., 2740.

6520, 1583.58972, -20., 2720.

6521, 1583.58972, -20., 2700.

6522, 1583.58972, -20., 2680.

6523, 1583.58972, -20., 2660.

6524, 1583.58972, -20., 2640.

6525, 1583.58972, -20., 2620.

6526, 1583.58972, -20., 2600.

6527, 1583.58972, -20., 2580.

6528, 1583.58972, -20., 2560.

6529, 1583.58972, -20., 2540.

6530, 1613.84619, -20., 2740.

6531, 1613.84619, -20., 2720.

6532, 1613.84619, -20., 2700.

6533, 1613.84619, -20., 2680.

6534, 1613.84619, -20., 2660.

6535, 1613.84619, -20., 2640.

6536, 1613.84619, -20., 2620.

6537, 1613.84619, -20., 2600.

6538, 1613.84619, -20., 2580.

6539, 1613.84619, -20., 2560.

6540, 1613.84619, -20., 2540.

6541, 1644.10254, -20., 2740.

6542, 1644.10254, -20., 2720.

6543, 1644.10254, -20., 2700.

6544, 1644.10254, -20., 2680.

6545, 1644.10254, -20., 2660.

6546, 1644.10254, -20., 2640.

6547, 1644.10254, -20., 2620.

6548, 1644.10254, -20., 2600.

6549, 1644.10254, -20., 2580.

6550, 1644.10254, -20., 2560.

6551, 1644.10254, -20., 2540.

6552, 1674.35901, -20., 2740.

6553, 1674.35901, -20., 2720.

6554, 1674.35901, -20., 2700.

6555, 1674.35901, -20., 2680.

6556, 1674.35901, -20., 2660.

6557, 1674.35901, -20., 2640.

6558, 1674.35901, -20., 2620.

6559, 1674.35901, -20., 2600.

6560, 1674.35901, -20., 2580.

6561, 1674.35901, -20., 2560.

6562, 1674.35901, -20., 2540.

6563, 1704.61536, -20., 2740.

6564, 1704.61536, -20., 2720.

6565, 1704.61536, -20., 2700.

6566, 1704.61536, -20., 2680.

6567, 1704.61536, -20., 2660.

6568, 1704.61536, -20., 2640.

6569, 1704.61536, -20., 2620.

6570, 1704.61536, -20., 2600.

6571, 1704.61536, -20., 2580.

6572, 1704.61536, -20., 2560.

6573, 1704.61536, -20., 2540.

6574, 1734.87183, -20., 2740.

6575, 1734.87183, -20., 2720.

6576, 1734.87183, -20., 2700.

6577, 1734.87183, -20., 2680.

6578, 1734.87183, -20., 2660.

6579, 1734.87183, -20., 2640.

6580, 1734.87183, -20., 2620.

6581, 1734.87183, -20., 2600.

6582, 1734.87183, -20., 2580.

6583, 1734.87183, -20., 2560.

6584, 1734.87183, -20., 2540.

6585, 1765.12817, -20., 2740.

6586, 1765.12817, -20., 2720.

6587, 1765.12817, -20., 2700.

6588, 1765.12817, -20., 2680.

6589, 1765.12817, -20., 2660.

6590, 1765.12817, -20., 2640.

6591, 1765.12817, -20., 2620.

6592, 1765.12817, -20., 2600.

6593, 1765.12817, -20., 2580.

6594, 1765.12817, -20., 2560.

6595, 1765.12817, -20., 2540.

6596, 1795.38464, -20., 2740.

6597, 1795.38464, -20., 2720.

6598, 1795.38464, -20., 2700.

6599, 1795.38464, -20., 2680.

6600, 1795.38464, -20., 2660.

6601, 1795.38464, -20., 2640.

6602, 1795.38464, -20., 2620.

6603, 1795.38464, -20., 2600.

6604, 1795.38464, -20., 2580.

6605, 1795.38464, -20., 2560.

6606, 1795.38464, -20., 2540.

6607, 1825.64099, -20., 2740.

6608, 1825.64099, -20., 2720.

6609, 1825.64099, -20., 2700.

6610, 1825.64099, -20., 2680.

6611, 1825.64099, -20., 2660.

6612, 1825.64099, -20., 2640.

6613, 1825.64099, -20., 2620.

6614, 1825.64099, -20., 2600.

6615, 1825.64099, -20., 2580.

6616, 1825.64099, -20., 2560.

6617, 1825.64099, -20., 2540.

6618, 1855.89746, -20., 2740.

6619, 1855.89746, -20., 2720.

6620, 1855.89746, -20., 2700.

6621, 1855.89746, -20., 2680.

6622, 1855.89746, -20., 2660.

6623, 1855.89746, -20., 2640.

6624, 1855.89746, -20., 2620.

6625, 1855.89746, -20., 2600.

6626, 1855.89746, -20., 2580.

6627, 1855.89746, -20., 2560.

6628, 1855.89746, -20., 2540.

6629, 1886.15381, -20., 2740.

6630, 1886.15381, -20., 2720.

6631, 1886.15381, -20., 2700.

6632, 1886.15381, -20., 2680.

6633, 1886.15381, -20., 2660.

6634, 1886.15381, -20., 2640.

6635, 1886.15381, -20., 2620.

6636, 1886.15381, -20., 2600.

6637, 1886.15381, -20., 2580.

6638, 1886.15381, -20., 2560.

6639, 1886.15381, -20., 2540.

6640, 1916.41028, -20., 2740.

6641, 1916.41028, -20., 2720.

6642, 1916.41028, -20., 2700.

6643, 1916.41028, -20., 2680.

6644, 1916.41028, -20., 2660.

6645, 1916.41028, -20., 2640.

6646, 1916.41028, -20., 2620.

6647, 1916.41028, -20., 2600.

6648, 1916.41028, -20., 2580.

6649, 1916.41028, -20., 2560.

6650, 1916.41028, -20., 2540.

6651, 1946.66663, -20., 2740.

6652, 1946.66663, -20., 2720.

6653, 1946.66663, -20., 2700.

6654, 1946.66663, -20., 2680.

6655, 1946.66663, -20., 2660.

6656, 1946.66663, -20., 2640.

6657, 1946.66663, -20., 2620.

6658, 1946.66663, -20., 2600.

6659, 1946.66663, -20., 2580.

6660, 1946.66663, -20., 2560.

6661, 1946.66663, -20., 2540.

6662, 1976.9231, -20., 2740.

6663, 1976.9231, -20., 2720.

6664, 1976.9231, -20., 2700.

6665, 1976.9231, -20., 2680.

6666, 1976.9231, -20., 2660.

6667, 1976.9231, -20., 2640.

6668, 1976.9231, -20., 2620.

6669, 1976.9231, -20., 2600.

6670, 1976.9231, -20., 2580.

6671, 1976.9231, -20., 2560.

6672, 1976.9231, -20., 2540.

6673, 2007.17944, -20., 2740.

6674, 2007.17944, -20., 2720.

6675, 2007.17944, -20., 2700.

6676, 2007.17944, -20., 2680.

6677, 2007.17944, -20., 2660.

6678, 2007.17944, -20., 2640.

6679, 2007.17944, -20., 2620.

6680, 2007.17944, -20., 2600.

6681, 2007.17944, -20., 2580.

6682, 2007.17944, -20., 2560.

6683, 2007.17944, -20., 2540.

6684, 2037.43591, -20., 2740.

6685, 2037.43591, -20., 2720.

6686, 2037.43591, -20., 2700.

6687, 2037.43591, -20., 2680.

6688, 2037.43591, -20., 2660.

6689, 2037.43591, -20., 2640.

6690, 2037.43591, -20., 2620.

6691, 2037.43591, -20., 2600.

6692, 2037.43591, -20., 2580.

6693, 2037.43591, -20., 2560.

6694, 2037.43591, -20., 2540.

6695, 2067.69238, -20., 2740.

6696, 2067.69238, -20., 2720.

6697, 2067.69238, -20., 2700.

6698, 2067.69238, -20., 2680.

6699, 2067.69238, -20., 2660.

6700, 2067.69238, -20., 2640.

6701, 2067.69238, -20., 2620.

6702, 2067.69238, -20., 2600.

6703, 2067.69238, -20., 2580.

6704, 2067.69238, -20., 2560.

6705, 2067.69238, -20., 2540.

6706, 2097.94873, -20., 2740.

6707, 2097.94873, -20., 2720.

6708, 2097.94873, -20., 2700.

6709, 2097.94873, -20., 2680.

6710, 2097.94873, -20., 2660.

6711, 2097.94873, -20., 2640.

6712, 2097.94873, -20., 2620.

6713, 2097.94873, -20., 2600.

6714, 2097.94873, -20., 2580.

6715, 2097.94873, -20., 2560.

6716, 2097.94873, -20., 2540.

6717, 2128.20508, -20., 2740.

6718, 2128.20508, -20., 2720.

6719, 2128.20508, -20., 2700.

6720, 2128.20508, -20., 2680.

6721, 2128.20508, -20., 2660.

6722, 2128.20508, -20., 2640.

6723, 2128.20508, -20., 2620.

6724, 2128.20508, -20., 2600.

6725, 2128.20508, -20., 2580.

6726, 2128.20508, -20., 2560.

6727, 2128.20508, -20., 2540.

6728, 2158.46143, -20., 2740.

6729, 2158.46143, -20., 2720.

6730, 2158.46143, -20., 2700.

6731, 2158.46143, -20., 2680.

6732, 2158.46143, -20., 2660.

6733, 2158.46143, -20., 2640.

6734, 2158.46143, -20., 2620.

6735, 2158.46143, -20., 2600.

6736, 2158.46143, -20., 2580.

6737, 2158.46143, -20., 2560.

6738, 2158.46143, -20., 2540.

6739, 2188.71802, -20., 2740.

6740, 2188.71802, -20., 2720.

6741, 2188.71802, -20., 2700.

6742, 2188.71802, -20., 2680.

6743, 2188.71802, -20., 2660.

6744, 2188.71802, -20., 2640.

6745, 2188.71802, -20., 2620.

6746, 2188.71802, -20., 2600.

6747, 2188.71802, -20., 2580.

6748, 2188.71802, -20., 2560.

6749, 2188.71802, -20., 2540.

6750, 2218.97437, -20., 2740.

6751, 2218.97437, -20., 2720.

6752, 2218.97437, -20., 2700.

6753, 2218.97437, -20., 2680.

6754, 2218.97437, -20., 2660.

6755, 2218.97437, -20., 2640.

6756, 2218.97437, -20., 2620.

6757, 2218.97437, -20., 2600.

6758, 2218.97437, -20., 2580.

6759, 2218.97437, -20., 2560.

6760, 2218.97437, -20., 2540.

6761, 2249.23071, -20., 2740.

6762, 2249.23071, -20., 2720.

6763, 2249.23071, -20., 2700.

6764, 2249.23071, -20., 2680.

6765, 2249.23071, -20., 2660.

6766, 2249.23071, -20., 2640.

6767, 2249.23071, -20., 2620.

6768, 2249.23071, -20., 2600.

6769, 2249.23071, -20., 2580.

6770, 2249.23071, -20., 2560.

6771, 2249.23071, -20., 2540.

6772, 2279.48706, -20., 2740.

6773, 2279.48706, -20., 2720.

6774, 2279.48706, -20., 2700.

6775, 2279.48706, -20., 2680.

6776, 2279.48706, -20., 2660.

6777, 2279.48706, -20., 2640.

6778, 2279.48706, -20., 2620.

6779, 2279.48706, -20., 2600.

6780, 2279.48706, -20., 2580.

6781, 2279.48706, -20., 2560.

6782, 2279.48706, -20., 2540.

6783, 2309.74365, -20., 2740.

6784, 2309.74365, -20., 2720.

6785, 2309.74365, -20., 2700.

6786, 2309.74365, -20., 2680.

6787, 2309.74365, -20., 2660.

6788, 2309.74365, -20., 2640.

6789, 2309.74365, -20., 2620.

6790, 2309.74365, -20., 2600.

6791, 2309.74365, -20., 2580.

6792, 2309.74365, -20., 2560.

6793, 2309.74365, -20., 2540.

6794, 128.203644, -101.197174, 2540.

6795, 128.203644, -101.197174, 2560.

6796, 128.203644, -101.197174, 2580.

6797, 128.203644, -101.197174, 2600.

6798, 128.203644, -101.197174, 2620.

6799, 128.203644, -101.197174, 2640.

6800, 128.203644, -101.197174, 2660.

6801, 128.203644, -101.197174, 2680.

6802, 128.203644, -101.197174, 2700.

6803, 128.203644, -101.197174, 2720.

6804, 128.203644, -101.197174, 2740.

6805, 105.469093, -100.798119, 2540.

6806, 105.469093, -100.798119, 2560.

6807, 105.469093, -100.798119, 2580.

6808, 105.469093, -100.798119, 2600.

6809, 105.469093, -100.798119, 2620.

6810, 105.469093, -100.798119, 2640.

6811, 105.469093, -100.798119, 2660.

6812, 105.469093, -100.798119, 2680.

6813, 105.469093, -100.798119, 2700.

6814, 105.469093, -100.798119, 2720.

6815, 105.469093, -100.798119, 2740.

6816, 82.7345428, -100.399055, 2540.

6817, 82.7345428, -100.399055, 2560.

6818, 82.7345428, -100.399055, 2580.

6819, 82.7345428, -100.399055, 2600.

6820, 82.7345428, -100.399055, 2620.

6821, 82.7345428, -100.399055, 2640.

6822, 82.7345428, -100.399055, 2660.

6823, 82.7345428, -100.399055, 2680.

6824, 82.7345428, -100.399055, 2700.

6825, 82.7345428, -100.399055, 2720.

6826, 82.7345428, -100.399055, 2740.

6827, 133.281219, -80.964447, 2540.

6828, 133.281219, -80.964447, 2560.

6829, 133.281219, -80.964447, 2580.

6830, 133.281219, -80.964447, 2600.

6831, 133.281219, -80.964447, 2620.

6832, 133.281219, -80.964447, 2640.

6833, 133.281219, -80.964447, 2660.

6834, 133.281219, -80.964447, 2680.

6835, 133.281219, -80.964447, 2700.

6836, 133.281219, -80.964447, 2720.

6837, 133.281219, -80.964447, 2740.

6838, 108.854141, -80.6429672, 2540.

6839, 108.854141, -80.6429672, 2560.

6840, 108.854141, -80.6429672, 2580.

6841, 108.854141, -80.6429672, 2600.

6842, 108.854141, -80.6429672, 2620.

6843, 108.854141, -80.6429672, 2640.

6844, 108.854141, -80.6429672, 2660.

6845, 108.854141, -80.6429672, 2680.

6846, 108.854141, -80.6429672, 2700.

6847, 108.854141, -80.6429672, 2720.

6848, 108.854141, -80.6429672, 2740.

6849, 84.4270706, -80.3214798, 2540.

6850, 84.4270706, -80.3214798, 2560.

6851, 84.4270706, -80.3214798, 2580.

6852, 84.4270706, -80.3214798, 2600.

6853, 84.4270706, -80.3214798, 2620.

6854, 84.4270706, -80.3214798, 2640.

6855, 84.4270706, -80.3214798, 2660.

6856, 84.4270706, -80.3214798, 2680.

6857, 84.4270706, -80.3214798, 2700.

6858, 84.4270706, -80.3214798, 2720.

6859, 84.4270706, -80.3214798, 2740.

6860, 135., -60., 2540.

6861, 135., -60., 2560.

6862, 135., -60., 2580.

6863, 135., -60., 2600.

6864, 135., -60., 2620.

6865, 135., -60., 2640.

6866, 135., -60., 2660.

6867, 135., -60., 2680.

6868, 135., -60., 2700.

6869, 135., -60., 2720.

6870, 135., -60., 2740.

6871, 110., -60., 2540.

6872, 110., -60., 2560.

6873, 110., -60., 2580.

6874, 110., -60., 2600.

6875, 110., -60., 2620.

6876, 110., -60., 2640.

6877, 110., -60., 2660.

6878, 110., -60., 2680.

6879, 110., -60., 2700.

6880, 110., -60., 2720.

6881, 110., -60., 2740.

6882, 85., -60., 2540.

6883, 85., -60., 2560.

6884, 85., -60., 2580.

6885, 85., -60., 2600.

6886, 85., -60., 2620.

6887, 85., -60., 2640.

6888, 85., -60., 2660.

6889, 85., -60., 2680.

6890, 85., -60., 2700.

6891, 85., -60., 2720.

6892, 85., -60., 2740.

6893, 133.281219, -39.035553, 2540.

6894, 133.281219, -39.035553, 2560.

6895, 133.281219, -39.035553, 2580.

6896, 133.281219, -39.035553, 2600.

6897, 133.281219, -39.035553, 2620.

6898, 133.281219, -39.035553, 2640.

6899, 133.281219, -39.035553, 2660.

6900, 133.281219, -39.035553, 2680.

6901, 133.281219, -39.035553, 2700.

6902, 133.281219, -39.035553, 2720.

6903, 133.281219, -39.035553, 2740.

6904, 108.854141, -39.3570366, 2540.

6905, 108.854141, -39.3570366, 2560.

6906, 108.854141, -39.3570366, 2580.

6907, 108.854141, -39.3570366, 2600.

6908, 108.854141, -39.3570366, 2620.

6909, 108.854141, -39.3570366, 2640.

6910, 108.854141, -39.3570366, 2660.

6911, 108.854141, -39.3570366, 2680.

6912, 108.854141, -39.3570366, 2700.

6913, 108.854141, -39.3570366, 2720.

6914, 108.854141, -39.3570366, 2740.

6915, 84.4270706, -39.6785164, 2540.

6916, 84.4270706, -39.6785164, 2560.

6917, 84.4270706, -39.6785164, 2580.

6918, 84.4270706, -39.6785164, 2600.

6919, 84.4270706, -39.6785164, 2620.

6920, 84.4270706, -39.6785164, 2640.

6921, 84.4270706, -39.6785164, 2660.

6922, 84.4270706, -39.6785164, 2680.

6923, 84.4270706, -39.6785164, 2700.

6924, 84.4270706, -39.6785164, 2720.

6925, 84.4270706, -39.6785164, 2740.

6926, 128.203644, -18.8028278, 2540.

6927, 128.203644, -18.8028278, 2560.

6928, 128.203644, -18.8028278, 2580.

6929, 128.203644, -18.8028278, 2600.

6930, 128.203644, -18.8028278, 2620.

6931, 128.203644, -18.8028278, 2640.

6932, 128.203644, -18.8028278, 2660.

6933, 128.203644, -18.8028278, 2680.

6934, 128.203644, -18.8028278, 2700.

6935, 128.203644, -18.8028278, 2720.

6936, 128.203644, -18.8028278, 2740.

6937, 105.469093, -19.2018852, 2540.

6938, 105.469093, -19.2018852, 2560.

6939, 105.469093, -19.2018852, 2580.

6940, 105.469093, -19.2018852, 2600.

6941, 105.469093, -19.2018852, 2620.

6942, 105.469093, -19.2018852, 2640.

6943, 105.469093, -19.2018852, 2660.

6944, 105.469093, -19.2018852, 2680.

6945, 105.469093, -19.2018852, 2700.

6946, 105.469093, -19.2018852, 2720.

6947, 105.469093, -19.2018852, 2740.

6948, 82.7345428, -19.6009426, 2540.

6949, 82.7345428, -19.6009426, 2560.

6950, 82.7345428, -19.6009426, 2580.

6951, 82.7345428, -19.6009426, 2600.

6952, 82.7345428, -19.6009426, 2620.

6953, 82.7345428, -19.6009426, 2640.

6954, 82.7345428, -19.6009426, 2660.

6955, 82.7345428, -19.6009426, 2680.

6956, 82.7345428, -19.6009426, 2700.

6957, 82.7345428, -19.6009426, 2720.

6958, 82.7345428, -19.6009426, 2740.

6959, 1131.42859, -100., 2540.

6960, 1131.42859, -100., 2560.

6961, 1131.42859, -100., 2580.

6962, 1131.42859, -100., 2600.

6963, 1131.42859, -100., 2620.

6964, 1131.42859, -100., 2640.

6965, 1131.42859, -100., 2660.

6966, 1131.42859, -100., 2680.

6967, 1131.42859, -100., 2700.

6968, 1131.42859, -100., 2720.

6969, 1131.42859, -100., 2740.

6970, 1102.85718, -100., 2540.

6971, 1102.85718, -100., 2560.

6972, 1102.85718, -100., 2580.

6973, 1102.85718, -100., 2600.

6974, 1102.85718, -100., 2620.

6975, 1102.85718, -100., 2640.

6976, 1102.85718, -100., 2660.

6977, 1102.85718, -100., 2680.

6978, 1102.85718, -100., 2700.

6979, 1102.85718, -100., 2720.

6980, 1102.85718, -100., 2740.

6981, 1074.28577, -100., 2540.

6982, 1074.28577, -100., 2560.

6983, 1074.28577, -100., 2580.

6984, 1074.28577, -100., 2600.

6985, 1074.28577, -100., 2620.

6986, 1074.28577, -100., 2640.

6987, 1074.28577, -100., 2660.

6988, 1074.28577, -100., 2680.

6989, 1074.28577, -100., 2700.

6990, 1074.28577, -100., 2720.

6991, 1074.28577, -100., 2740.

6992, 1045.71423, -100., 2540.

6993, 1045.71423, -100., 2560.

6994, 1045.71423, -100., 2580.

6995, 1045.71423, -100., 2600.

6996, 1045.71423, -100., 2620.

6997, 1045.71423, -100., 2640.

6998, 1045.71423, -100., 2660.

6999, 1045.71423, -100., 2680.

7000, 1045.71423, -100., 2700.

7001, 1045.71423, -100., 2720.

7002, 1045.71423, -100., 2740.

7003, 1017.14288, -100., 2540.

7004, 1017.14288, -100., 2560.

7005, 1017.14288, -100., 2580.

7006, 1017.14288, -100., 2600.

7007, 1017.14288, -100., 2620.

7008, 1017.14288, -100., 2640.

7009, 1017.14288, -100., 2660.

7010, 1017.14288, -100., 2680.

7011, 1017.14288, -100., 2700.

7012, 1017.14288, -100., 2720.

7013, 1017.14288, -100., 2740.

7014, 988.571411, -100., 2540.

7015, 988.571411, -100., 2560.

7016, 988.571411, -100., 2580.

7017, 988.571411, -100., 2600.

7018, 988.571411, -100., 2620.

7019, 988.571411, -100., 2640.

7020, 988.571411, -100., 2660.

7021, 988.571411, -100., 2680.

7022, 988.571411, -100., 2700.

7023, 988.571411, -100., 2720.

7024, 988.571411, -100., 2740.

7025, 1131.42859, -80., 2540.

7026, 1131.42859, -80., 2560.

7027, 1131.42859, -80., 2580.

7028, 1131.42859, -80., 2600.

7029, 1131.42859, -80., 2620.

7030, 1131.42859, -80., 2640.

7031, 1131.42859, -80., 2660.

7032, 1131.42859, -80., 2680.

7033, 1131.42859, -80., 2700.

7034, 1131.42859, -80., 2720.

7035, 1131.42859, -80., 2740.

7036, 1102.85718, -80., 2540.

7037, 1102.85718, -80., 2560.

7038, 1102.85718, -80., 2580.

7039, 1102.85718, -80., 2600.

7040, 1102.85718, -80., 2620.

7041, 1102.85718, -80., 2640.

7042, 1102.85718, -80., 2660.

7043, 1102.85718, -80., 2680.

7044, 1102.85718, -80., 2700.

7045, 1102.85718, -80., 2720.

7046, 1102.85718, -80., 2740.

7047, 1074.28577, -80., 2540.

7048, 1074.28577, -80., 2560.

7049, 1074.28577, -80., 2580.

7050, 1074.28577, -80., 2600.

7051, 1074.28577, -80., 2620.

7052, 1074.28577, -80., 2640.

7053, 1074.28577, -80., 2660.

7054, 1074.28577, -80., 2680.

7055, 1074.28577, -80., 2700.

7056, 1074.28577, -80., 2720.

7057, 1074.28577, -80., 2740.

7058, 1045.71423, -80., 2540.

7059, 1045.71423, -80., 2560.

7060, 1045.71423, -80., 2580.

7061, 1045.71423, -80., 2600.

7062, 1045.71423, -80., 2620.

7063, 1045.71423, -80., 2640.

7064, 1045.71423, -80., 2660.

7065, 1045.71423, -80., 2680.

7066, 1045.71423, -80., 2700.

7067, 1045.71423, -80., 2720.

7068, 1045.71423, -80., 2740.

7069, 1017.14288, -80., 2540.

7070, 1017.14288, -80., 2560.

7071, 1017.14288, -80., 2580.

7072, 1017.14288, -80., 2600.

7073, 1017.14288, -80., 2620.

7074, 1017.14288, -80., 2640.

7075, 1017.14288, -80., 2660.

7076, 1017.14288, -80., 2680.

7077, 1017.14288, -80., 2700.

7078, 1017.14288, -80., 2720.

7079, 1017.14288, -80., 2740.

7080, 988.571411, -80., 2540.

7081, 988.571411, -80., 2560.

7082, 988.571411, -80., 2580.

7083, 988.571411, -80., 2600.

7084, 988.571411, -80., 2620.

7085, 988.571411, -80., 2640.

7086, 988.571411, -80., 2660.

7087, 988.571411, -80., 2680.

7088, 988.571411, -80., 2700.

7089, 988.571411, -80., 2720.

7090, 988.571411, -80., 2740.

7091, 1131.42859, -60., 2540.

7092, 1131.42859, -60., 2560.

7093, 1131.42859, -60., 2580.

7094, 1131.42859, -60., 2600.

7095, 1131.42859, -60., 2620.

7096, 1131.42859, -60., 2640.

7097, 1131.42859, -60., 2660.

7098, 1131.42859, -60., 2680.

7099, 1131.42859, -60., 2700.

7100, 1131.42859, -60., 2720.

7101, 1131.42859, -60., 2740.

7102, 1102.85718, -60., 2540.

7103, 1102.85718, -60., 2560.

7104, 1102.85718, -60., 2580.

7105, 1102.85718, -60., 2600.

7106, 1102.85718, -60., 2620.

7107, 1102.85718, -60., 2640.

7108, 1102.85718, -60., 2660.

7109, 1102.85718, -60., 2680.

7110, 1102.85718, -60., 2700.

7111, 1102.85718, -60., 2720.

7112, 1102.85718, -60., 2740.

7113, 1074.28577, -60., 2540.

7114, 1074.28577, -60., 2560.

7115, 1074.28577, -60., 2580.

7116, 1074.28577, -60., 2600.

7117, 1074.28577, -60., 2620.

7118, 1074.28577, -60., 2640.

7119, 1074.28577, -60., 2660.

7120, 1074.28577, -60., 2680.

7121, 1074.28577, -60., 2700.

7122, 1074.28577, -60., 2720.

7123, 1074.28577, -60., 2740.

7124, 1045.71423, -60., 2540.

7125, 1045.71423, -60., 2560.

7126, 1045.71423, -60., 2580.

7127, 1045.71423, -60., 2600.

7128, 1045.71423, -60., 2620.

7129, 1045.71423, -60., 2640.

7130, 1045.71423, -60., 2660.

7131, 1045.71423, -60., 2680.

7132, 1045.71423, -60., 2700.

7133, 1045.71423, -60., 2720.

7134, 1045.71423, -60., 2740.

7135, 1017.14288, -60., 2540.

7136, 1017.14288, -60., 2560.

7137, 1017.14288, -60., 2580.

7138, 1017.14288, -60., 2600.

7139, 1017.14288, -60., 2620.

7140, 1017.14288, -60., 2640.

7141, 1017.14288, -60., 2660.

7142, 1017.14288, -60., 2680.

7143, 1017.14288, -60., 2700.

7144, 1017.14288, -60., 2720.

7145, 1017.14288, -60., 2740.

7146, 988.571411, -60., 2540.

7147, 988.571411, -60., 2560.

7148, 988.571411, -60., 2580.

7149, 988.571411, -60., 2600.

7150, 988.571411, -60., 2620.

7151, 988.571411, -60., 2640.

7152, 988.571411, -60., 2660.

7153, 988.571411, -60., 2680.

7154, 988.571411, -60., 2700.

7155, 988.571411, -60., 2720.

7156, 988.571411, -60., 2740.

7157, 1131.42859, -40., 2540.

7158, 1131.42859, -40., 2560.

7159, 1131.42859, -40., 2580.

7160, 1131.42859, -40., 2600.

7161, 1131.42859, -40., 2620.

7162, 1131.42859, -40., 2640.

7163, 1131.42859, -40., 2660.

7164, 1131.42859, -40., 2680.

7165, 1131.42859, -40., 2700.

7166, 1131.42859, -40., 2720.

7167, 1131.42859, -40., 2740.

7168, 1102.85718, -40., 2540.

7169, 1102.85718, -40., 2560.

7170, 1102.85718, -40., 2580.

7171, 1102.85718, -40., 2600.

7172, 1102.85718, -40., 2620.

7173, 1102.85718, -40., 2640.

7174, 1102.85718, -40., 2660.

7175, 1102.85718, -40., 2680.

7176, 1102.85718, -40., 2700.

7177, 1102.85718, -40., 2720.

7178, 1102.85718, -40., 2740.

7179, 1074.28577, -40., 2540.

7180, 1074.28577, -40., 2560.

7181, 1074.28577, -40., 2580.

7182, 1074.28577, -40., 2600.

7183, 1074.28577, -40., 2620.

7184, 1074.28577, -40., 2640.

7185, 1074.28577, -40., 2660.

7186, 1074.28577, -40., 2680.

7187, 1074.28577, -40., 2700.

7188, 1074.28577, -40., 2720.

7189, 1074.28577, -40., 2740.

7190, 1045.71423, -40., 2540.

7191, 1045.71423, -40., 2560.

7192, 1045.71423, -40., 2580.

7193, 1045.71423, -40., 2600.

7194, 1045.71423, -40., 2620.

7195, 1045.71423, -40., 2640.

7196, 1045.71423, -40., 2660.

7197, 1045.71423, -40., 2680.

7198, 1045.71423, -40., 2700.

7199, 1045.71423, -40., 2720.

7200, 1045.71423, -40., 2740.

7201, 1017.14288, -40., 2540.

7202, 1017.14288, -40., 2560.

7203, 1017.14288, -40., 2580.

7204, 1017.14288, -40., 2600.

7205, 1017.14288, -40., 2620.

7206, 1017.14288, -40., 2640.

7207, 1017.14288, -40., 2660.

7208, 1017.14288, -40., 2680.

7209, 1017.14288, -40., 2700.

7210, 1017.14288, -40., 2720.

7211, 1017.14288, -40., 2740.

7212, 988.571411, -40., 2540.

7213, 988.571411, -40., 2560.

7214, 988.571411, -40., 2580.

7215, 988.571411, -40., 2600.

7216, 988.571411, -40., 2620.

7217, 988.571411, -40., 2640.

7218, 988.571411, -40., 2660.

7219, 988.571411, -40., 2680.

7220, 988.571411, -40., 2700.

7221, 988.571411, -40., 2720.

7222, 988.571411, -40., 2740.

7223, 1131.42859, -20., 2540.

7224, 1131.42859, -20., 2560.

7225, 1131.42859, -20., 2580.

7226, 1131.42859, -20., 2600.

7227, 1131.42859, -20., 2620.

7228, 1131.42859, -20., 2640.

7229, 1131.42859, -20., 2660.

7230, 1131.42859, -20., 2680.

7231, 1131.42859, -20., 2700.

7232, 1131.42859, -20., 2720.

7233, 1131.42859, -20., 2740.

7234, 1102.85718, -20., 2540.

7235, 1102.85718, -20., 2560.

7236, 1102.85718, -20., 2580.

7237, 1102.85718, -20., 2600.

7238, 1102.85718, -20., 2620.

7239, 1102.85718, -20., 2640.

7240, 1102.85718, -20., 2660.

7241, 1102.85718, -20., 2680.

7242, 1102.85718, -20., 2700.

7243, 1102.85718, -20., 2720.

7244, 1102.85718, -20., 2740.

7245, 1074.28577, -20., 2540.

7246, 1074.28577, -20., 2560.

7247, 1074.28577, -20., 2580.

7248, 1074.28577, -20., 2600.

7249, 1074.28577, -20., 2620.

7250, 1074.28577, -20., 2640.

7251, 1074.28577, -20., 2660.

7252, 1074.28577, -20., 2680.

7253, 1074.28577, -20., 2700.

7254, 1074.28577, -20., 2720.

7255, 1074.28577, -20., 2740.

7256, 1045.71423, -20., 2540.

7257, 1045.71423, -20., 2560.

7258, 1045.71423, -20., 2580.

7259, 1045.71423, -20., 2600.

7260, 1045.71423, -20., 2620.

7261, 1045.71423, -20., 2640.

7262, 1045.71423, -20., 2660.

7263, 1045.71423, -20., 2680.

7264, 1045.71423, -20., 2700.

7265, 1045.71423, -20., 2720.

7266, 1045.71423, -20., 2740.

7267, 1017.14288, -20., 2540.

7268, 1017.14288, -20., 2560.

7269, 1017.14288, -20., 2580.

7270, 1017.14288, -20., 2600.

7271, 1017.14288, -20., 2620.

7272, 1017.14288, -20., 2640.

7273, 1017.14288, -20., 2660.

7274, 1017.14288, -20., 2680.

7275, 1017.14288, -20., 2700.

7276, 1017.14288, -20., 2720.

7277, 1017.14288, -20., 2740.

7278, 988.571411, -20., 2540.

7279, 988.571411, -20., 2560.

7280, 988.571411, -20., 2580.

7281, 988.571411, -20., 2600.

7282, 988.571411, -20., 2620.

7283, 988.571411, -20., 2640.

7284, 988.571411, -20., 2660.

7285, 988.571411, -20., 2680.

7286, 988.571411, -20., 2700.

7287, 988.571411, -20., 2720.

7288, 988.571411, -20., 2740.

7289, 37.2654533, -19.6009426, 2640.

7290, 37.2654533, -19.6009426, 2620.

7291, 37.2654533, -19.6009426, 2600.

7292, 37.2654533, -19.6009426, 2580.

7293, 37.2654533, -19.6009426, 2560.

7294, 37.2654533, -19.6009426, 2540.

7295, 14.5309067, -19.2018852, 2640.

7296, 14.5309067, -19.2018852, 2620.

7297, 14.5309067, -19.2018852, 2600.

7298, 14.5309067, -19.2018852, 2580.

7299, 14.5309067, -19.2018852, 2560.

7300, 14.5309067, -19.2018852, 2540.

7301, -8.20363903, -18.8028278, 2640.

7302, -8.20363903, -18.8028278, 2620.

7303, -8.20363903, -18.8028278, 2600.

7304, -8.20363903, -18.8028278, 2580.

7305, -8.20363903, -18.8028278, 2560.

7306, -8.20363903, -18.8028278, 2540.

7307, 35.5729294, -39.6785164, 2640.

7308, 35.5729294, -39.6785164, 2620.

7309, 35.5729294, -39.6785164, 2600.

7310, 35.5729294, -39.6785164, 2580.

7311, 35.5729294, -39.6785164, 2560.

7312, 35.5729294, -39.6785164, 2540.

7313, 11.1458569, -39.3570366, 2640.

7314, 11.1458569, -39.3570366, 2620.

7315, 11.1458569, -39.3570366, 2600.

7316, 11.1458569, -39.3570366, 2580.

7317, 11.1458569, -39.3570366, 2560.

7318, 11.1458569, -39.3570366, 2540.

7319, -13.2812147, -39.035553, 2640.

7320, -13.2812147, -39.035553, 2620.

7321, -13.2812147, -39.035553, 2600.

7322, -13.2812147, -39.035553, 2580.

7323, -13.2812147, -39.035553, 2560.

7324, -13.2812147, -39.035553, 2540.

7325, 35., -60., 2640.

7326, 35., -60., 2620.

7327, 35., -60., 2600.

7328, 35., -60., 2580.

7329, 35., -60., 2560.

7330, 35., -60., 2540.

7331, 10., -60., 2640.

7332, 10., -60., 2620.

7333, 10., -60., 2600.

7334, 10., -60., 2580.

7335, 10., -60., 2560.

7336, 10., -60., 2540.

7337, -15., -60., 2640.

7338, -15., -60., 2620.

7339, -15., -60., 2600.

7340, -15., -60., 2580.

7341, -15., -60., 2560.

7342, -15., -60., 2540.

7343, 35.5729294, -80.3214798, 2640.

7344, 35.5729294, -80.3214798, 2620.

7345, 35.5729294, -80.3214798, 2600.

7346, 35.5729294, -80.3214798, 2580.

7347, 35.5729294, -80.3214798, 2560.

7348, 35.5729294, -80.3214798, 2540.

7349, 11.1458569, -80.6429672, 2640.

7350, 11.1458569, -80.6429672, 2620.

7351, 11.1458569, -80.6429672, 2600.

7352, 11.1458569, -80.6429672, 2580.

7353, 11.1458569, -80.6429672, 2560.

7354, 11.1458569, -80.6429672, 2540.

7355, -13.2812147, -80.964447, 2640.

7356, -13.2812147, -80.964447, 2620.

7357, -13.2812147, -80.964447, 2600.

7358, -13.2812147, -80.964447, 2580.

7359, -13.2812147, -80.964447, 2560.

7360, -13.2812147, -80.964447, 2540.

7361, 37.2654533, -100.399055, 2640.

7362, 37.2654533, -100.399055, 2620.

7363, 37.2654533, -100.399055, 2600.

7364, 37.2654533, -100.399055, 2580.

7365, 37.2654533, -100.399055, 2560.

7366, 37.2654533, -100.399055, 2540.

7367, 14.5309067, -100.798119, 2640.

7368, 14.5309067, -100.798119, 2620.

7369, 14.5309067, -100.798119, 2600.

7370, 14.5309067, -100.798119, 2580.

7371, 14.5309067, -100.798119, 2560.

7372, 14.5309067, -100.798119, 2540.

7373, -8.20363903, -101.197174, 2640.

7374, -8.20363903, -101.197174, 2620.

7375, -8.20363903, -101.197174, 2600.

7376, -8.20363903, -101.197174, 2580.

7377, -8.20363903, -101.197174, 2560.

7378, -8.20363903, -101.197174, 2540.

*Element, type=C3D8R

1, 190, 1374, 3274, 1114, 1, 33, 542, 106

2, 1374, 1375, 3275, 3274, 33, 34, 543, 542

3, 1375, 1376, 3276, 3275, 34, 35, 544, 543

4, 1376, 1377, 3277, 3276, 35, 36, 545, 544

5, 1377, 1378, 3278, 3277, 36, 37, 546, 545

6, 1378, 1379, 3279, 3278, 37, 38, 547, 546

7, 1379, 1380, 3280, 3279, 38, 39, 548, 547

8, 1380, 1381, 3281, 3280, 39, 40, 549, 548

9, 1381, 1382, 3282, 3281, 40, 41, 550, 549

10, 1382, 1383, 3283, 3282, 41, 42, 551, 550

11, 1383, 1384, 3284, 3283, 42, 43, 552, 551

12, 1384, 196, 1244, 3284, 43, 2, 44, 552

13, 1114, 3274, 3285, 1115, 106, 542, 553, 105

14, 3274, 3275, 3286, 3285, 542, 543, 554, 553

15, 3275, 3276, 3287, 3286, 543, 544, 555, 554

16, 3276, 3277, 3288, 3287, 544, 545, 556, 555

17, 3277, 3278, 3289, 3288, 545, 546, 557, 556

18, 3278, 3279, 3290, 3289, 546, 547, 558, 557

19, 3279, 3280, 3291, 3290, 547, 548, 559, 558

20, 3280, 3281, 3292, 3291, 548, 549, 560, 559

21, 3281, 3282, 3293, 3292, 549, 550, 561, 560

22, 3282, 3283, 3294, 3293, 550, 551, 562, 561

23, 3283, 3284, 3295, 3294, 551, 552, 563, 562

24, 3284, 1244, 1245, 3295, 552, 44, 45, 563

25, 1115, 3285, 3296, 1116, 105, 553, 564, 104

26, 3285, 3286, 3297, 3296, 553, 554, 565, 564

27, 3286, 3287, 3298, 3297, 554, 555, 566, 565

28, 3287, 3288, 3299, 3298, 555, 556, 567, 566

29, 3288, 3289, 3300, 3299, 556, 557, 568, 567

30, 3289, 3290, 3301, 3300, 557, 558, 569, 568

31, 3290, 3291, 3302, 3301, 558, 559, 570, 569

32, 3291, 3292, 3303, 3302, 559, 560, 571, 570

33, 3292, 3293, 3304, 3303, 560, 561, 572, 571

34, 3293, 3294, 3305, 3304, 561, 562, 573, 572

35, 3294, 3295, 3306, 3305, 562, 563, 574, 573

36, 3295, 1245, 1246, 3306, 563, 45, 46, 574

37, 1116, 3296, 3307, 1117, 104, 564, 575, 103

38, 3296, 3297, 3308, 3307, 564, 565, 576, 575

39, 3297, 3298, 3309, 3308, 565, 566, 577, 576

40, 3298, 3299, 3310, 3309, 566, 567, 578, 577

41, 3299, 3300, 3311, 3310, 567, 568, 579, 578

42, 3300, 3301, 3312, 3311, 568, 569, 580, 579

43, 3301, 3302, 3313, 3312, 569, 570, 581, 580

44, 3302, 3303, 3314, 3313, 570, 571, 582, 581

45, 3303, 3304, 3315, 3314, 571, 572, 583, 582

46, 3304, 3305, 3316, 3315, 572, 573, 584, 583

47, 3305, 3306, 3317, 3316, 573, 574, 585, 584

48, 3306, 1246, 1247, 3317, 574, 46, 47, 585

49, 1117, 3307, 3318, 1118, 103, 575, 586, 102

50, 3307, 3308, 3319, 3318, 575, 576, 587, 586

51, 3308, 3309, 3320, 3319, 576, 577, 588, 587

52, 3309, 3310, 3321, 3320, 577, 578, 589, 588

53, 3310, 3311, 3322, 3321, 578, 579, 590, 589

54, 3311, 3312, 3323, 3322, 579, 580, 591, 590

55, 3312, 3313, 3324, 3323, 580, 581, 592, 591

56, 3313, 3314, 3325, 3324, 581, 582, 593, 592

57, 3314, 3315, 3326, 3325, 582, 583, 594, 593

58, 3315, 3316, 3327, 3326, 583, 584, 595, 594

59, 3316, 3317, 3328, 3327, 584, 585, 596, 595

60, 3317, 1247, 1248, 3328, 585, 47, 48, 596

61, 1118, 3318, 3329, 1119, 102, 586, 597, 101

62, 3318, 3319, 3330, 3329, 586, 587, 598, 597

63, 3319, 3320, 3331, 3330, 587, 588, 599, 598

64, 3320, 3321, 3332, 3331, 588, 589, 600, 599

65, 3321, 3322, 3333, 3332, 589, 590, 601, 600

66, 3322, 3323, 3334, 3333, 590, 591, 602, 601

67, 3323, 3324, 3335, 3334, 591, 592, 603, 602

68, 3324, 3325, 3336, 3335, 592, 593, 604, 603

69, 3325, 3326, 3337, 3336, 593, 594, 605, 604

70, 3326, 3327, 3338, 3337, 594, 595, 606, 605

71, 3327, 3328, 3339, 3338, 595, 596, 607, 606

72, 3328, 1248, 1249, 3339, 596, 48, 49, 607

73, 1119, 3329, 3340, 1120, 101, 597, 608, 100

74, 3329, 3330, 3341, 3340, 597, 598, 609, 608

75, 3330, 3331, 3342, 3341, 598, 599, 610, 609

76, 3331, 3332, 3343, 3342, 599, 600, 611, 610

77, 3332, 3333, 3344, 3343, 600, 601, 612, 611

78, 3333, 3334, 3345, 3344, 601, 602, 613, 612

79, 3334, 3335, 3346, 3345, 602, 603, 614, 613

80, 3335, 3336, 3347, 3346, 603, 604, 615, 614

81, 3336, 3337, 3348, 3347, 604, 605, 616, 615

82, 3337, 3338, 3349, 3348, 605, 606, 617, 616

83, 3338, 3339, 3350, 3349, 606, 607, 618, 617

84, 3339, 1249, 1250, 3350, 607, 49, 50, 618

85, 1120, 3340, 3351, 1121, 100, 608, 619, 99

86, 3340, 3341, 3352, 3351, 608, 609, 620, 619

87, 3341, 3342, 3353, 3352, 609, 610, 621, 620

88, 3342, 3343, 3354, 3353, 610, 611, 622, 621

89, 3343, 3344, 3355, 3354, 611, 612, 623, 622

90, 3344, 3345, 3356, 3355, 612, 613, 624, 623

91, 3345, 3346, 3357, 3356, 613, 614, 625, 624

92, 3346, 3347, 3358, 3357, 614, 615, 626, 625

93, 3347, 3348, 3359, 3358, 615, 616, 627, 626

94, 3348, 3349, 3360, 3359, 616, 617, 628, 627

95, 3349, 3350, 3361, 3360, 617, 618, 629, 628

96, 3350, 1250, 1251, 3361, 618, 50, 51, 629

97, 1121, 3351, 3362, 1122, 99, 619, 630, 98

98, 3351, 3352, 3363, 3362, 619, 620, 631, 630

99, 3352, 3353, 3364, 3363, 620, 621, 632, 631

100, 3353, 3354, 3365, 3364, 621, 622, 633, 632

101, 3354, 3355, 3366, 3365, 622, 623, 634, 633

102, 3355, 3356, 3367, 3366, 623, 624, 635, 634

103, 3356, 3357, 3368, 3367, 624, 625, 636, 635

104, 3357, 3358, 3369, 3368, 625, 626, 637, 636

105, 3358, 3359, 3370, 3369, 626, 627, 638, 637

106, 3359, 3360, 3371, 3370, 627, 628, 639, 638

107, 3360, 3361, 3372, 3371, 628, 629, 640, 639

108, 3361, 1251, 1252, 3372, 629, 51, 52, 640

109, 1122, 3362, 3373, 1123, 98, 630, 641, 97

110, 3362, 3363, 3374, 3373, 630, 631, 642, 641

111, 3363, 3364, 3375, 3374, 631, 632, 643, 642

112, 3364, 3365, 3376, 3375, 632, 633, 644, 643

113, 3365, 3366, 3377, 3376, 633, 634, 645, 644

114, 3366, 3367, 3378, 3377, 634, 635, 646, 645

115, 3367, 3368, 3379, 3378, 635, 636, 647, 646

116, 3368, 3369, 3380, 3379, 636, 637, 648, 647

117, 3369, 3370, 3381, 3380, 637, 638, 649, 648

118, 3370, 3371, 3382, 3381, 638, 639, 650, 649

119, 3371, 3372, 3383, 3382, 639, 640, 651, 650

120, 3372, 1252, 1253, 3383, 640, 52, 53, 651

121, 1123, 3373, 3384, 1124, 97, 641, 652, 96

122, 3373, 3374, 3385, 3384, 641, 642, 653, 652

123, 3374, 3375, 3386, 3385, 642, 643, 654, 653

124, 3375, 3376, 3387, 3386, 643, 644, 655, 654

125, 3376, 3377, 3388, 3387, 644, 645, 656, 655

126, 3377, 3378, 3389, 3388, 645, 646, 657, 656

127, 3378, 3379, 3390, 3389, 646, 647, 658, 657

128, 3379, 3380, 3391, 3390, 647, 648, 659, 658

129, 3380, 3381, 3392, 3391, 648, 649, 660, 659

130, 3381, 3382, 3393, 3392, 649, 650, 661, 660

131, 3382, 3383, 3394, 3393, 650, 651, 662, 661

132, 3383, 1253, 1254, 3394, 651, 53, 54, 662

133, 1124, 3384, 3395, 1125, 96, 652, 663, 95

134, 3384, 3385, 3396, 3395, 652, 653, 664, 663

135, 3385, 3386, 3397, 3396, 653, 654, 665, 664

136, 3386, 3387, 3398, 3397, 654, 655, 666, 665

137, 3387, 3388, 3399, 3398, 655, 656, 667, 666

138, 3388, 3389, 3400, 3399, 656, 657, 668, 667

139, 3389, 3390, 3401, 3400, 657, 658, 669, 668

140, 3390, 3391, 3402, 3401, 658, 659, 670, 669

141, 3391, 3392, 3403, 3402, 659, 660, 671, 670

142, 3392, 3393, 3404, 3403, 660, 661, 672, 671

143, 3393, 3394, 3405, 3404, 661, 662, 673, 672

144, 3394, 1254, 1255, 3405, 662, 54, 55, 673

145, 1125, 3395, 3406, 1126, 95, 663, 674, 94

146, 3395, 3396, 3407, 3406, 663, 664, 675, 674

147, 3396, 3397, 3408, 3407, 664, 665, 676, 675

148, 3397, 3398, 3409, 3408, 665, 666, 677, 676

149, 3398, 3399, 3410, 3409, 666, 667, 678, 677

150, 3399, 3400, 3411, 3410, 667, 668, 679, 678

151, 3400, 3401, 3412, 3411, 668, 669, 680, 679

152, 3401, 3402, 3413, 3412, 669, 670, 681, 680

153, 3402, 3403, 3414, 3413, 670, 671, 682, 681

154, 3403, 3404, 3415, 3414, 671, 672, 683, 682

155, 3404, 3405, 3416, 3415, 672, 673, 684, 683

156, 3405, 1255, 1256, 3416, 673, 55, 56, 684

157, 1126, 3406, 3417, 1127, 94, 674, 685, 93

158, 3406, 3407, 3418, 3417, 674, 675, 686, 685

159, 3407, 3408, 3419, 3418, 675, 676, 687, 686

160, 3408, 3409, 3420, 3419, 676, 677, 688, 687

161, 3409, 3410, 3421, 3420, 677, 678, 689, 688

162, 3410, 3411, 3422, 3421, 678, 679, 690, 689

163, 3411, 3412, 3423, 3422, 679, 680, 691, 690

164, 3412, 3413, 3424, 3423, 680, 681, 692, 691

165, 3413, 3414, 3425, 3424, 681, 682, 693, 692

166, 3414, 3415, 3426, 3425, 682, 683, 694, 693

167, 3415, 3416, 3427, 3426, 683, 684, 695, 694

168, 3416, 1256, 1257, 3427, 684, 56, 57, 695

169, 1127, 3417, 3428, 1128, 93, 685, 696, 92

170, 3417, 3418, 3429, 3428, 685, 686, 697, 696

171, 3418, 3419, 3430, 3429, 686, 687, 698, 697

172, 3419, 3420, 3431, 3430, 687, 688, 699, 698

173, 3420, 3421, 3432, 3431, 688, 689, 700, 699

174, 3421, 3422, 3433, 3432, 689, 690, 701, 700

175, 3422, 3423, 3434, 3433, 690, 691, 702, 701

176, 3423, 3424, 3435, 3434, 691, 692, 703, 702

177, 3424, 3425, 3436, 3435, 692, 693, 704, 703

178, 3425, 3426, 3437, 3436, 693, 694, 705, 704

179, 3426, 3427, 3438, 3437, 694, 695, 706, 705

180, 3427, 1257, 1258, 3438, 695, 57, 58, 706

181, 1128, 3428, 3439, 1129, 92, 696, 707, 91

182, 3428, 3429, 3440, 3439, 696, 697, 708, 707

183, 3429, 3430, 3441, 3440, 697, 698, 709, 708

184, 3430, 3431, 3442, 3441, 698, 699, 710, 709

185, 3431, 3432, 3443, 3442, 699, 700, 711, 710

186, 3432, 3433, 3444, 3443, 700, 701, 712, 711

187, 3433, 3434, 3445, 3444, 701, 702, 713, 712

188, 3434, 3435, 3446, 3445, 702, 703, 714, 713

189, 3435, 3436, 3447, 3446, 703, 704, 715, 714

190, 3436, 3437, 3448, 3447, 704, 705, 716, 715

191, 3437, 3438, 3449, 3448, 705, 706, 717, 716

192, 3438, 1258, 1259, 3449, 706, 58, 59, 717

193, 1129, 3439, 3450, 1130, 91, 707, 718, 90

194, 3439, 3440, 3451, 3450, 707, 708, 719, 718

195, 3440, 3441, 3452, 3451, 708, 709, 720, 719

196, 3441, 3442, 3453, 3452, 709, 710, 721, 720

197, 3442, 3443, 3454, 3453, 710, 711, 722, 721

198, 3443, 3444, 3455, 3454, 711, 712, 723, 722

199, 3444, 3445, 3456, 3455, 712, 713, 724, 723

200, 3445, 3446, 3457, 3456, 713, 714, 725, 724

201, 3446, 3447, 3458, 3457, 714, 715, 726, 725

202, 3447, 3448, 3459, 3458, 715, 716, 727, 726

203, 3448, 3449, 3460, 3459, 716, 717, 728, 727

204, 3449, 1259, 1260, 3460, 717, 59, 60, 728

205, 1130, 3450, 3461, 1131, 90, 718, 729, 89

206, 3450, 3451, 3462, 3461, 718, 719, 730, 729

207, 3451, 3452, 3463, 3462, 719, 720, 731, 730

208, 3452, 3453, 3464, 3463, 720, 721, 732, 731

209, 3453, 3454, 3465, 3464, 721, 722, 733, 732

210, 3454, 3455, 3466, 3465, 722, 723, 734, 733

211, 3455, 3456, 3467, 3466, 723, 724, 735, 734

212, 3456, 3457, 3468, 3467, 724, 725, 736, 735

213, 3457, 3458, 3469, 3468, 725, 726, 737, 736

214, 3458, 3459, 3470, 3469, 726, 727, 738, 737

215, 3459, 3460, 3471, 3470, 727, 728, 739, 738

216, 3460, 1260, 1261, 3471, 728, 60, 61, 739

217, 1131, 3461, 3472, 1132, 89, 729, 740, 88

218, 3461, 3462, 3473, 3472, 729, 730, 741, 740

219, 3462, 3463, 3474, 3473, 730, 731, 742, 741

220, 3463, 3464, 3475, 3474, 731, 732, 743, 742

221, 3464, 3465, 3476, 3475, 732, 733, 744, 743

222, 3465, 3466, 3477, 3476, 733, 734, 745, 744

223, 3466, 3467, 3478, 3477, 734, 735, 746, 745

224, 3467, 3468, 3479, 3478, 735, 736, 747, 746

225, 3468, 3469, 3480, 3479, 736, 737, 748, 747

226, 3469, 3470, 3481, 3480, 737, 738, 749, 748

227, 3470, 3471, 3482, 3481, 738, 739, 750, 749

228, 3471, 1261, 1262, 3482, 739, 61, 62, 750

229, 1132, 3472, 3483, 1133, 88, 740, 751, 87

230, 3472, 3473, 3484, 3483, 740, 741, 752, 751

231, 3473, 3474, 3485, 3484, 741, 742, 753, 752

232, 3474, 3475, 3486, 3485, 742, 743, 754, 753

233, 3475, 3476, 3487, 3486, 743, 744, 755, 754

234, 3476, 3477, 3488, 3487, 744, 745, 756, 755

235, 3477, 3478, 3489, 3488, 745, 746, 757, 756

236, 3478, 3479, 3490, 3489, 746, 747, 758, 757

237, 3479, 3480, 3491, 3490, 747, 748, 759, 758

238, 3480, 3481, 3492, 3491, 748, 749, 760, 759

239, 3481, 3482, 3493, 3492, 749, 750, 761, 760

240, 3482, 1262, 1263, 3493, 750, 62, 63, 761

241, 1133, 3483, 3494, 1134, 87, 751, 762, 86

242, 3483, 3484, 3495, 3494, 751, 752, 763, 762

243, 3484, 3485, 3496, 3495, 752, 753, 764, 763

244, 3485, 3486, 3497, 3496, 753, 754, 765, 764

245, 3486, 3487, 3498, 3497, 754, 755, 766, 765

246, 3487, 3488, 3499, 3498, 755, 756, 767, 766

247, 3488, 3489, 3500, 3499, 756, 757, 768, 767

248, 3489, 3490, 3501, 3500, 757, 758, 769, 768

249, 3490, 3491, 3502, 3501, 758, 759, 770, 769

250, 3491, 3492, 3503, 3502, 759, 760, 771, 770

251, 3492, 3493, 3504, 3503, 760, 761, 772, 771

252, 3493, 1263, 1264, 3504, 761, 63, 64, 772

253, 1134, 3494, 3505, 1135, 86, 762, 773, 85

254, 3494, 3495, 3506, 3505, 762, 763, 774, 773

255, 3495, 3496, 3507, 3506, 763, 764, 775, 774

256, 3496, 3497, 3508, 3507, 764, 765, 776, 775

257, 3497, 3498, 3509, 3508, 765, 766, 777, 776

258, 3498, 3499, 3510, 3509, 766, 767, 778, 777

259, 3499, 3500, 3511, 3510, 767, 768, 779, 778

260, 3500, 3501, 3512, 3511, 768, 769, 780, 779

261, 3501, 3502, 3513, 3512, 769, 770, 781, 780

262, 3502, 3503, 3514, 3513, 770, 771, 782, 781

263, 3503, 3504, 3515, 3514, 771, 772, 783, 782

264, 3504, 1264, 1265, 3515, 772, 64, 65, 783

265, 1135, 3505, 3516, 1136, 85, 773, 784, 84

266, 3505, 3506, 3517, 3516, 773, 774, 785, 784

267, 3506, 3507, 3518, 3517, 774, 775, 786, 785

268, 3507, 3508, 3519, 3518, 775, 776, 787, 786

269, 3508, 3509, 3520, 3519, 776, 777, 788, 787

270, 3509, 3510, 3521, 3520, 777, 778, 789, 788

271, 3510, 3511, 3522, 3521, 778, 779, 790, 789

272, 3511, 3512, 3523, 3522, 779, 780, 791, 790

273, 3512, 3513, 3524, 3523, 780, 781, 792, 791

274, 3513, 3514, 3525, 3524, 781, 782, 793, 792

275, 3514, 3515, 3526, 3525, 782, 783, 794, 793

276, 3515, 1265, 1266, 3526, 783, 65, 66, 794

277, 1136, 3516, 3527, 1137, 84, 784, 795, 83

278, 3516, 3517, 3528, 3527, 784, 785, 796, 795

279, 3517, 3518, 3529, 3528, 785, 786, 797, 796

280, 3518, 3519, 3530, 3529, 786, 787, 798, 797

281, 3519, 3520, 3531, 3530, 787, 788, 799, 798

282, 3520, 3521, 3532, 3531, 788, 789, 800, 799

283, 3521, 3522, 3533, 3532, 789, 790, 801, 800

284, 3522, 3523, 3534, 3533, 790, 791, 802, 801

285, 3523, 3524, 3535, 3534, 791, 792, 803, 802

286, 3524, 3525, 3536, 3535, 792, 793, 804, 803

287, 3525, 3526, 3537, 3536, 793, 794, 805, 804

288, 3526, 1266, 1267, 3537, 794, 66, 67, 805

289, 1137, 3527, 3538, 1138, 83, 795, 806, 82

290, 3527, 3528, 3539, 3538, 795, 796, 807, 806

291, 3528, 3529, 3540, 3539, 796, 797, 808, 807

292, 3529, 3530, 3541, 3540, 797, 798, 809, 808

293, 3530, 3531, 3542, 3541, 798, 799, 810, 809

294, 3531, 3532, 3543, 3542, 799, 800, 811, 810

295, 3532, 3533, 3544, 3543, 800, 801, 812, 811

296, 3533, 3534, 3545, 3544, 801, 802, 813, 812

297, 3534, 3535, 3546, 3545, 802, 803, 814, 813

298, 3535, 3536, 3547, 3546, 803, 804, 815, 814

299, 3536, 3537, 3548, 3547, 804, 805, 816, 815

300, 3537, 1267, 1268, 3548, 805, 67, 68, 816

301, 1138, 3538, 3549, 1139, 82, 806, 817, 81

302, 3538, 3539, 3550, 3549, 806, 807, 818, 817

303, 3539, 3540, 3551, 3550, 807, 808, 819, 818

304, 3540, 3541, 3552, 3551, 808, 809, 820, 819

305, 3541, 3542, 3553, 3552, 809, 810, 821, 820

306, 3542, 3543, 3554, 3553, 810, 811, 822, 821

307, 3543, 3544, 3555, 3554, 811, 812, 823, 822

308, 3544, 3545, 3556, 3555, 812, 813, 824, 823

309, 3545, 3546, 3557, 3556, 813, 814, 825, 824

310, 3546, 3547, 3558, 3557, 814, 815, 826, 825

311, 3547, 3548, 3559, 3558, 815, 816, 827, 826

312, 3548, 1268, 1269, 3559, 816, 68, 69, 827

313, 1139, 3549, 1429, 181, 81, 817, 80, 4

314, 3549, 3550, 1430, 1429, 817, 818, 79, 80

315, 3550, 3551, 1431, 1430, 818, 819, 78, 79

316, 3551, 3552, 1432, 1431, 819, 820, 77, 78

317, 3552, 3553, 1433, 1432, 820, 821, 76, 77

318, 3553, 3554, 1434, 1433, 821, 822, 75, 76

319, 3554, 3555, 1435, 1434, 822, 823, 74, 75

320, 3555, 3556, 1436, 1435, 823, 824, 73, 74

321, 3556, 3557, 1437, 1436, 824, 825, 72, 73

322, 3557, 3558, 1438, 1437, 825, 826, 71, 72

323, 3558, 3559, 1439, 1438, 826, 827, 70, 71

324, 3559, 1269, 195, 1439, 827, 69, 3, 70

325, 189, 1385, 3560, 1140, 190, 1374, 3274, 1114

326, 1385, 1386, 3561, 3560, 1374, 1375, 3275, 3274

327, 1386, 1387, 3562, 3561, 1375, 1376, 3276, 3275

328, 1387, 1388, 3563, 3562, 1376, 1377, 3277, 3276

329, 1388, 1389, 3564, 3563, 1377, 1378, 3278, 3277

330, 1389, 1390, 3565, 3564, 1378, 1379, 3279, 3278

331, 1390, 1391, 3566, 3565, 1379, 1380, 3280, 3279

332, 1391, 1392, 3567, 3566, 1380, 1381, 3281, 3280

333, 1392, 1393, 3568, 3567, 1381, 1382, 3282, 3281

334, 1393, 1394, 3569, 3568, 1382, 1383, 3283, 3282

335, 1394, 1395, 3570, 3569, 1383, 1384, 3284, 3283

336, 1395, 197, 1270, 3570, 1384, 196, 1244, 3284

337, 1140, 3560, 3571, 1141, 1114, 3274, 3285, 1115

338, 3560, 3561, 3572, 3571, 3274, 3275, 3286, 3285

339, 3561, 3562, 3573, 3572, 3275, 3276, 3287, 3286

340, 3562, 3563, 3574, 3573, 3276, 3277, 3288, 3287

341, 3563, 3564, 3575, 3574, 3277, 3278, 3289, 3288

342, 3564, 3565, 3576, 3575, 3278, 3279, 3290, 3289

343, 3565, 3566, 3577, 3576, 3279, 3280, 3291, 3290

344, 3566, 3567, 3578, 3577, 3280, 3281, 3292, 3291

345, 3567, 3568, 3579, 3578, 3281, 3282, 3293, 3292

346, 3568, 3569, 3580, 3579, 3282, 3283, 3294, 3293

347, 3569, 3570, 3581, 3580, 3283, 3284, 3295, 3294

348, 3570, 1270, 1271, 3581, 3284, 1244, 1245, 3295

349, 1141, 3571, 3582, 1142, 1115, 3285, 3296, 1116

350, 3571, 3572, 3583, 3582, 3285, 3286, 3297, 3296

351, 3572, 3573, 3584, 3583, 3286, 3287, 3298, 3297

352, 3573, 3574, 3585, 3584, 3287, 3288, 3299, 3298

353, 3574, 3575, 3586, 3585, 3288, 3289, 3300, 3299

354, 3575, 3576, 3587, 3586, 3289, 3290, 3301, 3300

355, 3576, 3577, 3588, 3587, 3290, 3291, 3302, 3301

356, 3577, 3578, 3589, 3588, 3291, 3292, 3303, 3302

357, 3578, 3579, 3590, 3589, 3292, 3293, 3304, 3303

358, 3579, 3580, 3591, 3590, 3293, 3294, 3305, 3304

359, 3580, 3581, 3592, 3591, 3294, 3295, 3306, 3305

360, 3581, 1271, 1272, 3592, 3295, 1245, 1246, 3306

361, 1142, 3582, 3593, 1143, 1116, 3296, 3307, 1117

362, 3582, 3583, 3594, 3593, 3296, 3297, 3308, 3307

363, 3583, 3584, 3595, 3594, 3297, 3298, 3309, 3308

364, 3584, 3585, 3596, 3595, 3298, 3299, 3310, 3309

365, 3585, 3586, 3597, 3596, 3299, 3300, 3311, 3310

366, 3586, 3587, 3598, 3597, 3300, 3301, 3312, 3311

367, 3587, 3588, 3599, 3598, 3301, 3302, 3313, 3312

368, 3588, 3589, 3600, 3599, 3302, 3303, 3314, 3313

369, 3589, 3590, 3601, 3600, 3303, 3304, 3315, 3314

370, 3590, 3591, 3602, 3601, 3304, 3305, 3316, 3315

371, 3591, 3592, 3603, 3602, 3305, 3306, 3317, 3316

372, 3592, 1272, 1273, 3603, 3306, 1246, 1247, 3317

373, 1143, 3593, 3604, 1144, 1117, 3307, 3318, 1118

374, 3593, 3594, 3605, 3604, 3307, 3308, 3319, 3318

375, 3594, 3595, 3606, 3605, 3308, 3309, 3320, 3319

376, 3595, 3596, 3607, 3606, 3309, 3310, 3321, 3320

377, 3596, 3597, 3608, 3607, 3310, 3311, 3322, 3321

378, 3597, 3598, 3609, 3608, 3311, 3312, 3323, 3322

379, 3598, 3599, 3610, 3609, 3312, 3313, 3324, 3323

380, 3599, 3600, 3611, 3610, 3313, 3314, 3325, 3324

381, 3600, 3601, 3612, 3611, 3314, 3315, 3326, 3325

382, 3601, 3602, 3613, 3612, 3315, 3316, 3327, 3326

383, 3602, 3603, 3614, 3613, 3316, 3317, 3328, 3327

384, 3603, 1273, 1274, 3614, 3317, 1247, 1248, 3328

385, 1144, 3604, 3615, 1145, 1118, 3318, 3329, 1119

386, 3604, 3605, 3616, 3615, 3318, 3319, 3330, 3329

387, 3605, 3606, 3617, 3616, 3319, 3320, 3331, 3330

388, 3606, 3607, 3618, 3617, 3320, 3321, 3332, 3331

389, 3607, 3608, 3619, 3618, 3321, 3322, 3333, 3332

390, 3608, 3609, 3620, 3619, 3322, 3323, 3334, 3333

391, 3609, 3610, 3621, 3620, 3323, 3324, 3335, 3334

392, 3610, 3611, 3622, 3621, 3324, 3325, 3336, 3335

393, 3611, 3612, 3623, 3622, 3325, 3326, 3337, 3336

394, 3612, 3613, 3624, 3623, 3326, 3327, 3338, 3337

395, 3613, 3614, 3625, 3624, 3327, 3328, 3339, 3338

396, 3614, 1274, 1275, 3625, 3328, 1248, 1249, 3339

397, 1145, 3615, 3626, 1146, 1119, 3329, 3340, 1120

398, 3615, 3616, 3627, 3626, 3329, 3330, 3341, 3340

399, 3616, 3617, 3628, 3627, 3330, 3331, 3342, 3341

400, 3617, 3618, 3629, 3628, 3331, 3332, 3343, 3342

401, 3618, 3619, 3630, 3629, 3332, 3333, 3344, 3343

402, 3619, 3620, 3631, 3630, 3333, 3334, 3345, 3344

403, 3620, 3621, 3632, 3631, 3334, 3335, 3346, 3345

404, 3621, 3622, 3633, 3632, 3335, 3336, 3347, 3346

405, 3622, 3623, 3634, 3633, 3336, 3337, 3348, 3347

406, 3623, 3624, 3635, 3634, 3337, 3338, 3349, 3348

407, 3624, 3625, 3636, 3635, 3338, 3339, 3350, 3349

408, 3625, 1275, 1276, 3636, 3339, 1249, 1250, 3350

409, 1146, 3626, 3637, 1147, 1120, 3340, 3351, 1121

410, 3626, 3627, 3638, 3637, 3340, 3341, 3352, 3351

411, 3627, 3628, 3639, 3638, 3341, 3342, 3353, 3352

412, 3628, 3629, 3640, 3639, 3342, 3343, 3354, 3353

413, 3629, 3630, 3641, 3640, 3343, 3344, 3355, 3354

414, 3630, 3631, 3642, 3641, 3344, 3345, 3356, 3355

415, 3631, 3632, 3643, 3642, 3345, 3346, 3357, 3356

416, 3632, 3633, 3644, 3643, 3346, 3347, 3358, 3357

417, 3633, 3634, 3645, 3644, 3347, 3348, 3359, 3358

418, 3634, 3635, 3646, 3645, 3348, 3349, 3360, 3359

419, 3635, 3636, 3647, 3646, 3349, 3350, 3361, 3360

420, 3636, 1276, 1277, 3647, 3350, 1250, 1251, 3361

421, 1147, 3637, 3648, 1148, 1121, 3351, 3362, 1122

422, 3637, 3638, 3649, 3648, 3351, 3352, 3363, 3362

423, 3638, 3639, 3650, 3649, 3352, 3353, 3364, 3363

424, 3639, 3640, 3651, 3650, 3353, 3354, 3365, 3364

425, 3640, 3641, 3652, 3651, 3354, 3355, 3366, 3365

426, 3641, 3642, 3653, 3652, 3355, 3356, 3367, 3366

427, 3642, 3643, 3654, 3653, 3356, 3357, 3368, 3367

428, 3643, 3644, 3655, 3654, 3357, 3358, 3369, 3368

429, 3644, 3645, 3656, 3655, 3358, 3359, 3370, 3369

430, 3645, 3646, 3657, 3656, 3359, 3360, 3371, 3370

431, 3646, 3647, 3658, 3657, 3360, 3361, 3372, 3371

432, 3647, 1277, 1278, 3658, 3361, 1251, 1252, 3372

433, 1148, 3648, 3659, 1149, 1122, 3362, 3373, 1123

434, 3648, 3649, 3660, 3659, 3362, 3363, 3374, 3373

435, 3649, 3650, 3661, 3660, 3363, 3364, 3375, 3374

436, 3650, 3651, 3662, 3661, 3364, 3365, 3376, 3375

437, 3651, 3652, 3663, 3662, 3365, 3366, 3377, 3376

438, 3652, 3653, 3664, 3663, 3366, 3367, 3378, 3377

439, 3653, 3654, 3665, 3664, 3367, 3368, 3379, 3378

440, 3654, 3655, 3666, 3665, 3368, 3369, 3380, 3379

441, 3655, 3656, 3667, 3666, 3369, 3370, 3381, 3380

442, 3656, 3657, 3668, 3667, 3370, 3371, 3382, 3381

443, 3657, 3658, 3669, 3668, 3371, 3372, 3383, 3382

444, 3658, 1278, 1279, 3669, 3372, 1252, 1253, 3383

445, 1149, 3659, 3670, 1150, 1123, 3373, 3384, 1124

446, 3659, 3660, 3671, 3670, 3373, 3374, 3385, 3384

447, 3660, 3661, 3672, 3671, 3374, 3375, 3386, 3385

448, 3661, 3662, 3673, 3672, 3375, 3376, 3387, 3386

449, 3662, 3663, 3674, 3673, 3376, 3377, 3388, 3387

450, 3663, 3664, 3675, 3674, 3377, 3378, 3389, 3388

451, 3664, 3665, 3676, 3675, 3378, 3379, 3390, 3389

452, 3665, 3666, 3677, 3676, 3379, 3380, 3391, 3390

453, 3666, 3667, 3678, 3677, 3380, 3381, 3392, 3391

454, 3667, 3668, 3679, 3678, 3381, 3382, 3393, 3392

455, 3668, 3669, 3680, 3679, 3382, 3383, 3394, 3393

456, 3669, 1279, 1280, 3680, 3383, 1253, 1254, 3394

457, 1150, 3670, 3681, 1151, 1124, 3384, 3395, 1125

458, 3670, 3671, 3682, 3681, 3384, 3385, 3396, 3395

459, 3671, 3672, 3683, 3682, 3385, 3386, 3397, 3396

460, 3672, 3673, 3684, 3683, 3386, 3387, 3398, 3397

461, 3673, 3674, 3685, 3684, 3387, 3388, 3399, 3398

462, 3674, 3675, 3686, 3685, 3388, 3389, 3400, 3399

463, 3675, 3676, 3687, 3686, 3389, 3390, 3401, 3400

464, 3676, 3677, 3688, 3687, 3390, 3391, 3402, 3401

465, 3677, 3678, 3689, 3688, 3391, 3392, 3403, 3402

466, 3678, 3679, 3690, 3689, 3392, 3393, 3404, 3403

467, 3679, 3680, 3691, 3690, 3393, 3394, 3405, 3404

468, 3680, 1280, 1281, 3691, 3394, 1254, 1255, 3405

469, 1151, 3681, 3692, 1152, 1125, 3395, 3406, 1126

470, 3681, 3682, 3693, 3692, 3395, 3396, 3407, 3406

471, 3682, 3683, 3694, 3693, 3396, 3397, 3408, 3407

472, 3683, 3684, 3695, 3694, 3397, 3398, 3409, 3408

473, 3684, 3685, 3696, 3695, 3398, 3399, 3410, 3409

474, 3685, 3686, 3697, 3696, 3399, 3400, 3411, 3410

475, 3686, 3687, 3698, 3697, 3400, 3401, 3412, 3411

476, 3687, 3688, 3699, 3698, 3401, 3402, 3413, 3412

477, 3688, 3689, 3700, 3699, 3402, 3403, 3414, 3413

478, 3689, 3690, 3701, 3700, 3403, 3404, 3415, 3414

479, 3690, 3691, 3702, 3701, 3404, 3405, 3416, 3415

480, 3691, 1281, 1282, 3702, 3405, 1255, 1256, 3416

481, 1152, 3692, 3703, 1153, 1126, 3406, 3417, 1127

482, 3692, 3693, 3704, 3703, 3406, 3407, 3418, 3417

483, 3693, 3694, 3705, 3704, 3407, 3408, 3419, 3418

484, 3694, 3695, 3706, 3705, 3408, 3409, 3420, 3419

485, 3695, 3696, 3707, 3706, 3409, 3410, 3421, 3420

486, 3696, 3697, 3708, 3707, 3410, 3411, 3422, 3421

487, 3697, 3698, 3709, 3708, 3411, 3412, 3423, 3422

488, 3698, 3699, 3710, 3709, 3412, 3413, 3424, 3423

489, 3699, 3700, 3711, 3710, 3413, 3414, 3425, 3424

490, 3700, 3701, 3712, 3711, 3414, 3415, 3426, 3425

491, 3701, 3702, 3713, 3712, 3415, 3416, 3427, 3426

492, 3702, 1282, 1283, 3713, 3416, 1256, 1257, 3427

493, 1153, 3703, 3714, 1154, 1127, 3417, 3428, 1128

494, 3703, 3704, 3715, 3714, 3417, 3418, 3429, 3428

495, 3704, 3705, 3716, 3715, 3418, 3419, 3430, 3429

496, 3705, 3706, 3717, 3716, 3419, 3420, 3431, 3430

497, 3706, 3707, 3718, 3717, 3420, 3421, 3432, 3431

498, 3707, 3708, 3719, 3718, 3421, 3422, 3433, 3432

499, 3708, 3709, 3720, 3719, 3422, 3423, 3434, 3433

500, 3709, 3710, 3721, 3720, 3423, 3424, 3435, 3434

501, 3710, 3711, 3722, 3721, 3424, 3425, 3436, 3435

502, 3711, 3712, 3723, 3722, 3425, 3426, 3437, 3436

503, 3712, 3713, 3724, 3723, 3426, 3427, 3438, 3437

504, 3713, 1283, 1284, 3724, 3427, 1257, 1258, 3438

505, 1154, 3714, 3725, 1155, 1128, 3428, 3439, 1129

506, 3714, 3715, 3726, 3725, 3428, 3429, 3440, 3439

507, 3715, 3716, 3727, 3726, 3429, 3430, 3441, 3440

508, 3716, 3717, 3728, 3727, 3430, 3431, 3442, 3441

509, 3717, 3718, 3729, 3728, 3431, 3432, 3443, 3442

510, 3718, 3719, 3730, 3729, 3432, 3433, 3444, 3443

511, 3719, 3720, 3731, 3730, 3433, 3434, 3445, 3444

512, 3720, 3721, 3732, 3731, 3434, 3435, 3446, 3445

513, 3721, 3722, 3733, 3732, 3435, 3436, 3447, 3446

514, 3722, 3723, 3734, 3733, 3436, 3437, 3448, 3447

515, 3723, 3724, 3735, 3734, 3437, 3438, 3449, 3448

516, 3724, 1284, 1285, 3735, 3438, 1258, 1259, 3449

517, 1155, 3725, 3736, 1156, 1129, 3439, 3450, 1130

518, 3725, 3726, 3737, 3736, 3439, 3440, 3451, 3450

519, 3726, 3727, 3738, 3737, 3440, 3441, 3452, 3451

520, 3727, 3728, 3739, 3738, 3441, 3442, 3453, 3452

521, 3728, 3729, 3740, 3739, 3442, 3443, 3454, 3453

522, 3729, 3730, 3741, 3740, 3443, 3444, 3455, 3454

523, 3730, 3731, 3742, 3741, 3444, 3445, 3456, 3455

524, 3731, 3732, 3743, 3742, 3445, 3446, 3457, 3456

525, 3732, 3733, 3744, 3743, 3446, 3447, 3458, 3457

526, 3733, 3734, 3745, 3744, 3447, 3448, 3459, 3458

527, 3734, 3735, 3746, 3745, 3448, 3449, 3460, 3459

528, 3735, 1285, 1286, 3746, 3449, 1259, 1260, 3460

529, 1156, 3736, 3747, 1157, 1130, 3450, 3461, 1131

530, 3736, 3737, 3748, 3747, 3450, 3451, 3462, 3461

531, 3737, 3738, 3749, 3748, 3451, 3452, 3463, 3462

532, 3738, 3739, 3750, 3749, 3452, 3453, 3464, 3463

533, 3739, 3740, 3751, 3750, 3453, 3454, 3465, 3464

534, 3740, 3741, 3752, 3751, 3454, 3455, 3466, 3465

535, 3741, 3742, 3753, 3752, 3455, 3456, 3467, 3466

536, 3742, 3743, 3754, 3753, 3456, 3457, 3468, 3467

537, 3743, 3744, 3755, 3754, 3457, 3458, 3469, 3468

538, 3744, 3745, 3756, 3755, 3458, 3459, 3470, 3469

539, 3745, 3746, 3757, 3756, 3459, 3460, 3471, 3470

540, 3746, 1286, 1287, 3757, 3460, 1260, 1261, 3471

541, 1157, 3747, 3758, 1158, 1131, 3461, 3472, 1132

542, 3747, 3748, 3759, 3758, 3461, 3462, 3473, 3472

543, 3748, 3749, 3760, 3759, 3462, 3463, 3474, 3473

544, 3749, 3750, 3761, 3760, 3463, 3464, 3475, 3474

545, 3750, 3751, 3762, 3761, 3464, 3465, 3476, 3475

546, 3751, 3752, 3763, 3762, 3465, 3466, 3477, 3476

547, 3752, 3753, 3764, 3763, 3466, 3467, 3478, 3477

548, 3753, 3754, 3765, 3764, 3467, 3468, 3479, 3478

549, 3754, 3755, 3766, 3765, 3468, 3469, 3480, 3479

550, 3755, 3756, 3767, 3766, 3469, 3470, 3481, 3480

551, 3756, 3757, 3768, 3767, 3470, 3471, 3482, 3481

552, 3757, 1287, 1288, 3768, 3471, 1261, 1262, 3482

553, 1158, 3758, 3769, 1159, 1132, 3472, 3483, 1133

554, 3758, 3759, 3770, 3769, 3472, 3473, 3484, 3483

555, 3759, 3760, 3771, 3770, 3473, 3474, 3485, 3484

556, 3760, 3761, 3772, 3771, 3474, 3475, 3486, 3485

557, 3761, 3762, 3773, 3772, 3475, 3476, 3487, 3486

558, 3762, 3763, 3774, 3773, 3476, 3477, 3488, 3487

559, 3763, 3764, 3775, 3774, 3477, 3478, 3489, 3488

560, 3764, 3765, 3776, 3775, 3478, 3479, 3490, 3489

561, 3765, 3766, 3777, 3776, 3479, 3480, 3491, 3490

562, 3766, 3767, 3778, 3777, 3480, 3481, 3492, 3491

563, 3767, 3768, 3779, 3778, 3481, 3482, 3493, 3492

564, 3768, 1288, 1289, 3779, 3482, 1262, 1263, 3493

565, 1159, 3769, 3780, 1160, 1133, 3483, 3494, 1134

566, 3769, 3770, 3781, 3780, 3483, 3484, 3495, 3494

567, 3770, 3771, 3782, 3781, 3484, 3485, 3496, 3495

568, 3771, 3772, 3783, 3782, 3485, 3486, 3497, 3496

569, 3772, 3773, 3784, 3783, 3486, 3487, 3498, 3497

570, 3773, 3774, 3785, 3784, 3487, 3488, 3499, 3498

571, 3774, 3775, 3786, 3785, 3488, 3489, 3500, 3499

572, 3775, 3776, 3787, 3786, 3489, 3490, 3501, 3500

573, 3776, 3777, 3788, 3787, 3490, 3491, 3502, 3501

574, 3777, 3778, 3789, 3788, 3491, 3492, 3503, 3502

575, 3778, 3779, 3790, 3789, 3492, 3493, 3504, 3503

576, 3779, 1289, 1290, 3790, 3493, 1263, 1264, 3504

577, 1160, 3780, 3791, 1161, 1134, 3494, 3505, 1135

578, 3780, 3781, 3792, 3791, 3494, 3495, 3506, 3505

579, 3781, 3782, 3793, 3792, 3495, 3496, 3507, 3506

580, 3782, 3783, 3794, 3793, 3496, 3497, 3508, 3507

581, 3783, 3784, 3795, 3794, 3497, 3498, 3509, 3508

582, 3784, 3785, 3796, 3795, 3498, 3499, 3510, 3509

583, 3785, 3786, 3797, 3796, 3499, 3500, 3511, 3510

584, 3786, 3787, 3798, 3797, 3500, 3501, 3512, 3511

585, 3787, 3788, 3799, 3798, 3501, 3502, 3513, 3512

586, 3788, 3789, 3800, 3799, 3502, 3503, 3514, 3513

587, 3789, 3790, 3801, 3800, 3503, 3504, 3515, 3514

588, 3790, 1290, 1291, 3801, 3504, 1264, 1265, 3515

589, 1161, 3791, 3802, 1162, 1135, 3505, 3516, 1136

590, 3791, 3792, 3803, 3802, 3505, 3506, 3517, 3516

591, 3792, 3793, 3804, 3803, 3506, 3507, 3518, 3517

592, 3793, 3794, 3805, 3804, 3507, 3508, 3519, 3518

593, 3794, 3795, 3806, 3805, 3508, 3509, 3520, 3519

594, 3795, 3796, 3807, 3806, 3509, 3510, 3521, 3520

595, 3796, 3797, 3808, 3807, 3510, 3511, 3522, 3521

596, 3797, 3798, 3809, 3808, 3511, 3512, 3523, 3522

597, 3798, 3799, 3810, 3809, 3512, 3513, 3524, 3523

598, 3799, 3800, 3811, 3810, 3513, 3514, 3525, 3524

599, 3800, 3801, 3812, 3811, 3514, 3515, 3526, 3525

600, 3801, 1291, 1292, 3812, 3515, 1265, 1266, 3526

601, 1162, 3802, 3813, 1163, 1136, 3516, 3527, 1137

602, 3802, 3803, 3814, 3813, 3516, 3517, 3528, 3527

603, 3803, 3804, 3815, 3814, 3517, 3518, 3529, 3528

604, 3804, 3805, 3816, 3815, 3518, 3519, 3530, 3529

605, 3805, 3806, 3817, 3816, 3519, 3520, 3531, 3530

606, 3806, 3807, 3818, 3817, 3520, 3521, 3532, 3531

607, 3807, 3808, 3819, 3818, 3521, 3522, 3533, 3532

608, 3808, 3809, 3820, 3819, 3522, 3523, 3534, 3533

609, 3809, 3810, 3821, 3820, 3523, 3524, 3535, 3534

610, 3810, 3811, 3822, 3821, 3524, 3525, 3536, 3535

611, 3811, 3812, 3823, 3822, 3525, 3526, 3537, 3536

612, 3812, 1292, 1293, 3823, 3526, 1266, 1267, 3537

613, 1163, 3813, 3824, 1164, 1137, 3527, 3538, 1138

614, 3813, 3814, 3825, 3824, 3527, 3528, 3539, 3538

615, 3814, 3815, 3826, 3825, 3528, 3529, 3540, 3539

616, 3815, 3816, 3827, 3826, 3529, 3530, 3541, 3540

617, 3816, 3817, 3828, 3827, 3530, 3531, 3542, 3541

618, 3817, 3818, 3829, 3828, 3531, 3532, 3543, 3542

619, 3818, 3819, 3830, 3829, 3532, 3533, 3544, 3543

620, 3819, 3820, 3831, 3830, 3533, 3534, 3545, 3544

621, 3820, 3821, 3832, 3831, 3534, 3535, 3546, 3545

622, 3821, 3822, 3833, 3832, 3535, 3536, 3547, 3546

623, 3822, 3823, 3834, 3833, 3536, 3537, 3548, 3547

624, 3823, 1293, 1294, 3834, 3537, 1267, 1268, 3548

625, 1164, 3824, 3835, 1165, 1138, 3538, 3549, 1139

626, 3824, 3825, 3836, 3835, 3538, 3539, 3550, 3549

627, 3825, 3826, 3837, 3836, 3539, 3540, 3551, 3550

628, 3826, 3827, 3838, 3837, 3540, 3541, 3552, 3551

629, 3827, 3828, 3839, 3838, 3541, 3542, 3553, 3552

630, 3828, 3829, 3840, 3839, 3542, 3543, 3554, 3553

631, 3829, 3830, 3841, 3840, 3543, 3544, 3555, 3554

632, 3830, 3831, 3842, 3841, 3544, 3545, 3556, 3555

633, 3831, 3832, 3843, 3842, 3545, 3546, 3557, 3556

634, 3832, 3833, 3844, 3843, 3546, 3547, 3558, 3557

635, 3833, 3834, 3845, 3844, 3547, 3548, 3559, 3558

636, 3834, 1294, 1295, 3845, 3548, 1268, 1269, 3559

637, 1165, 3835, 1440, 182, 1139, 3549, 1429, 181

638, 3835, 3836, 1441, 1440, 3549, 3550, 1430, 1429

639, 3836, 3837, 1442, 1441, 3550, 3551, 1431, 1430

640, 3837, 3838, 1443, 1442, 3551, 3552, 1432, 1431

641, 3838, 3839, 1444, 1443, 3552, 3553, 1433, 1432

642, 3839, 3840, 1445, 1444, 3553, 3554, 1434, 1433

643, 3840, 3841, 1446, 1445, 3554, 3555, 1435, 1434

644, 3841, 3842, 1447, 1446, 3555, 3556, 1436, 1435

645, 3842, 3843, 1448, 1447, 3556, 3557, 1437, 1436

646, 3843, 3844, 1449, 1448, 3557, 3558, 1438, 1437

647, 3844, 3845, 1450, 1449, 3558, 3559, 1439, 1438

648, 3845, 1295, 194, 1450, 3559, 1269, 195, 1439

649, 188, 1396, 3846, 1166, 189, 1385, 3560, 1140

650, 1396, 1397, 3847, 3846, 1385, 1386, 3561, 3560

651, 1397, 1398, 3848, 3847, 1386, 1387, 3562, 3561

652, 1398, 1399, 3849, 3848, 1387, 1388, 3563, 3562

653, 1399, 1400, 3850, 3849, 1388, 1389, 3564, 3563

654, 1400, 1401, 3851, 3850, 1389, 1390, 3565, 3564

655, 1401, 1402, 3852, 3851, 1390, 1391, 3566, 3565

656, 1402, 1403, 3853, 3852, 1391, 1392, 3567, 3566

657, 1403, 1404, 3854, 3853, 1392, 1393, 3568, 3567

658, 1404, 1405, 3855, 3854, 1393, 1394, 3569, 3568

659, 1405, 1406, 3856, 3855, 1394, 1395, 3570, 3569

660, 1406, 198, 1296, 3856, 1395, 197, 1270, 3570

661, 1166, 3846, 3857, 1167, 1140, 3560, 3571, 1141

662, 3846, 3847, 3858, 3857, 3560, 3561, 3572, 3571

663, 3847, 3848, 3859, 3858, 3561, 3562, 3573, 3572

664, 3848, 3849, 3860, 3859, 3562, 3563, 3574, 3573

665, 3849, 3850, 3861, 3860, 3563, 3564, 3575, 3574

666, 3850, 3851, 3862, 3861, 3564, 3565, 3576, 3575

667, 3851, 3852, 3863, 3862, 3565, 3566, 3577, 3576

668, 3852, 3853, 3864, 3863, 3566, 3567, 3578, 3577

669, 3853, 3854, 3865, 3864, 3567, 3568, 3579, 3578

670, 3854, 3855, 3866, 3865, 3568, 3569, 3580, 3579

671, 3855, 3856, 3867, 3866, 3569, 3570, 3581, 3580

672, 3856, 1296, 1297, 3867, 3570, 1270, 1271, 3581

673, 1167, 3857, 3868, 1168, 1141, 3571, 3582, 1142

674, 3857, 3858, 3869, 3868, 3571, 3572, 3583, 3582

675, 3858, 3859, 3870, 3869, 3572, 3573, 3584, 3583

676, 3859, 3860, 3871, 3870, 3573, 3574, 3585, 3584

677, 3860, 3861, 3872, 3871, 3574, 3575, 3586, 3585

678, 3861, 3862, 3873, 3872, 3575, 3576, 3587, 3586

679, 3862, 3863, 3874, 3873, 3576, 3577, 3588, 3587

680, 3863, 3864, 3875, 3874, 3577, 3578, 3589, 3588

681, 3864, 3865, 3876, 3875, 3578, 3579, 3590, 3589

682, 3865, 3866, 3877, 3876, 3579, 3580, 3591, 3590

683, 3866, 3867, 3878, 3877, 3580, 3581, 3592, 3591

684, 3867, 1297, 1298, 3878, 3581, 1271, 1272, 3592

685, 1168, 3868, 3879, 1169, 1142, 3582, 3593, 1143

686, 3868, 3869, 3880, 3879, 3582, 3583, 3594, 3593

687, 3869, 3870, 3881, 3880, 3583, 3584, 3595, 3594

688, 3870, 3871, 3882, 3881, 3584, 3585, 3596, 3595

689, 3871, 3872, 3883, 3882, 3585, 3586, 3597, 3596

690, 3872, 3873, 3884, 3883, 3586, 3587, 3598, 3597

691, 3873, 3874, 3885, 3884, 3587, 3588, 3599, 3598

692, 3874, 3875, 3886, 3885, 3588, 3589, 3600, 3599

693, 3875, 3876, 3887, 3886, 3589, 3590, 3601, 3600

694, 3876, 3877, 3888, 3887, 3590, 3591, 3602, 3601

695, 3877, 3878, 3889, 3888, 3591, 3592, 3603, 3602

696, 3878, 1298, 1299, 3889, 3592, 1272, 1273, 3603

697, 1169, 3879, 3890, 1170, 1143, 3593, 3604, 1144

698, 3879, 3880, 3891, 3890, 3593, 3594, 3605, 3604

699, 3880, 3881, 3892, 3891, 3594, 3595, 3606, 3605

700, 3881, 3882, 3893, 3892, 3595, 3596, 3607, 3606

701, 3882, 3883, 3894, 3893, 3596, 3597, 3608, 3607

702, 3883, 3884, 3895, 3894, 3597, 3598, 3609, 3608

703, 3884, 3885, 3896, 3895, 3598, 3599, 3610, 3609

704, 3885, 3886, 3897, 3896, 3599, 3600, 3611, 3610

705, 3886, 3887, 3898, 3897, 3600, 3601, 3612, 3611

706, 3887, 3888, 3899, 3898, 3601, 3602, 3613, 3612

707, 3888, 3889, 3900, 3899, 3602, 3603, 3614, 3613

708, 3889, 1299, 1300, 3900, 3603, 1273, 1274, 3614

709, 1170, 3890, 3901, 1171, 1144, 3604, 3615, 1145

710, 3890, 3891, 3902, 3901, 3604, 3605, 3616, 3615

711, 3891, 3892, 3903, 3902, 3605, 3606, 3617, 3616

712, 3892, 3893, 3904, 3903, 3606, 3607, 3618, 3617

713, 3893, 3894, 3905, 3904, 3607, 3608, 3619, 3618

714, 3894, 3895, 3906, 3905, 3608, 3609, 3620, 3619

715, 3895, 3896, 3907, 3906, 3609, 3610, 3621, 3620

716, 3896, 3897, 3908, 3907, 3610, 3611, 3622, 3621

717, 3897, 3898, 3909, 3908, 3611, 3612, 3623, 3622

718, 3898, 3899, 3910, 3909, 3612, 3613, 3624, 3623

719, 3899, 3900, 3911, 3910, 3613, 3614, 3625, 3624

720, 3900, 1300, 1301, 3911, 3614, 1274, 1275, 3625

721, 1171, 3901, 3912, 1172, 1145, 3615, 3626, 1146

722, 3901, 3902, 3913, 3912, 3615, 3616, 3627, 3626

723, 3902, 3903, 3914, 3913, 3616, 3617, 3628, 3627

724, 3903, 3904, 3915, 3914, 3617, 3618, 3629, 3628

725, 3904, 3905, 3916, 3915, 3618, 3619, 3630, 3629

726, 3905, 3906, 3917, 3916, 3619, 3620, 3631, 3630

727, 3906, 3907, 3918, 3917, 3620, 3621, 3632, 3631

728, 3907, 3908, 3919, 3918, 3621, 3622, 3633, 3632

729, 3908, 3909, 3920, 3919, 3622, 3623, 3634, 3633

730, 3909, 3910, 3921, 3920, 3623, 3624, 3635, 3634

731, 3910, 3911, 3922, 3921, 3624, 3625, 3636, 3635

732, 3911, 1301, 1302, 3922, 3625, 1275, 1276, 3636

733, 1172, 3912, 3923, 1173, 1146, 3626, 3637, 1147

734, 3912, 3913, 3924, 3923, 3626, 3627, 3638, 3637

735, 3913, 3914, 3925, 3924, 3627, 3628, 3639, 3638

736, 3914, 3915, 3926, 3925, 3628, 3629, 3640, 3639

737, 3915, 3916, 3927, 3926, 3629, 3630, 3641, 3640

738, 3916, 3917, 3928, 3927, 3630, 3631, 3642, 3641

739, 3917, 3918, 3929, 3928, 3631, 3632, 3643, 3642

740, 3918, 3919, 3930, 3929, 3632, 3633, 3644, 3643

741, 3919, 3920, 3931, 3930, 3633, 3634, 3645, 3644

742, 3920, 3921, 3932, 3931, 3634, 3635, 3646, 3645

743, 3921, 3922, 3933, 3932, 3635, 3636, 3647, 3646

744, 3922, 1302, 1303, 3933, 3636, 1276, 1277, 3647

745, 1173, 3923, 3934, 1174, 1147, 3637, 3648, 1148

746, 3923, 3924, 3935, 3934, 3637, 3638, 3649, 3648

747, 3924, 3925, 3936, 3935, 3638, 3639, 3650, 3649

748, 3925, 3926, 3937, 3936, 3639, 3640, 3651, 3650

749, 3926, 3927, 3938, 3937, 3640, 3641, 3652, 3651

750, 3927, 3928, 3939, 3938, 3641, 3642, 3653, 3652

751, 3928, 3929, 3940, 3939, 3642, 3643, 3654, 3653

752, 3929, 3930, 3941, 3940, 3643, 3644, 3655, 3654

753, 3930, 3931, 3942, 3941, 3644, 3645, 3656, 3655

754, 3931, 3932, 3943, 3942, 3645, 3646, 3657, 3656

755, 3932, 3933, 3944, 3943, 3646, 3647, 3658, 3657

756, 3933, 1303, 1304, 3944, 3647, 1277, 1278, 3658

757, 1174, 3934, 3945, 1175, 1148, 3648, 3659, 1149

758, 3934, 3935, 3946, 3945, 3648, 3649, 3660, 3659

759, 3935, 3936, 3947, 3946, 3649, 3650, 3661, 3660

760, 3936, 3937, 3948, 3947, 3650, 3651, 3662, 3661

761, 3937, 3938, 3949, 3948, 3651, 3652, 3663, 3662

762, 3938, 3939, 3950, 3949, 3652, 3653, 3664, 3663

763, 3939, 3940, 3951, 3950, 3653, 3654, 3665, 3664

764, 3940, 3941, 3952, 3951, 3654, 3655, 3666, 3665

765, 3941, 3942, 3953, 3952, 3655, 3656, 3667, 3666

766, 3942, 3943, 3954, 3953, 3656, 3657, 3668, 3667

767, 3943, 3944, 3955, 3954, 3657, 3658, 3669, 3668

768, 3944, 1304, 1305, 3955, 3658, 1278, 1279, 3669

769, 1175, 3945, 3956, 1176, 1149, 3659, 3670, 1150

770, 3945, 3946, 3957, 3956, 3659, 3660, 3671, 3670

771, 3946, 3947, 3958, 3957, 3660, 3661, 3672, 3671

772, 3947, 3948, 3959, 3958, 3661, 3662, 3673, 3672

773, 3948, 3949, 3960, 3959, 3662, 3663, 3674, 3673

774, 3949, 3950, 3961, 3960, 3663, 3664, 3675, 3674

775, 3950, 3951, 3962, 3961, 3664, 3665, 3676, 3675

776, 3951, 3952, 3963, 3962, 3665, 3666, 3677, 3676

777, 3952, 3953, 3964, 3963, 3666, 3667, 3678, 3677

778, 3953, 3954, 3965, 3964, 3667, 3668, 3679, 3678

779, 3954, 3955, 3966, 3965, 3668, 3669, 3680, 3679

780, 3955, 1305, 1306, 3966, 3669, 1279, 1280, 3680

781, 1176, 3956, 3967, 1177, 1150, 3670, 3681, 1151

782, 3956, 3957, 3968, 3967, 3670, 3671, 3682, 3681

783, 3957, 3958, 3969, 3968, 3671, 3672, 3683, 3682

784, 3958, 3959, 3970, 3969, 3672, 3673, 3684, 3683

785, 3959, 3960, 3971, 3970, 3673, 3674, 3685, 3684

786, 3960, 3961, 3972, 3971, 3674, 3675, 3686, 3685

787, 3961, 3962, 3973, 3972, 3675, 3676, 3687, 3686

788, 3962, 3963, 3974, 3973, 3676, 3677, 3688, 3687

789, 3963, 3964, 3975, 3974, 3677, 3678, 3689, 3688

790, 3964, 3965, 3976, 3975, 3678, 3679, 3690, 3689

791, 3965, 3966, 3977, 3976, 3679, 3680, 3691, 3690

792, 3966, 1306, 1307, 3977, 3680, 1280, 1281, 3691

793, 1177, 3967, 3978, 1178, 1151, 3681, 3692, 1152

794, 3967, 3968, 3979, 3978, 3681, 3682, 3693, 3692

795, 3968, 3969, 3980, 3979, 3682, 3683, 3694, 3693

796, 3969, 3970, 3981, 3980, 3683, 3684, 3695, 3694

797, 3970, 3971, 3982, 3981, 3684, 3685, 3696, 3695

798, 3971, 3972, 3983, 3982, 3685, 3686, 3697, 3696

799, 3972, 3973, 3984, 3983, 3686, 3687, 3698, 3697

800, 3973, 3974, 3985, 3984, 3687, 3688, 3699, 3698

801, 3974, 3975, 3986, 3985, 3688, 3689, 3700, 3699

802, 3975, 3976, 3987, 3986, 3689, 3690, 3701, 3700

803, 3976, 3977, 3988, 3987, 3690, 3691, 3702, 3701

804, 3977, 1307, 1308, 3988, 3691, 1281, 1282, 3702

805, 1178, 3978, 3989, 1179, 1152, 3692, 3703, 1153

806, 3978, 3979, 3990, 3989, 3692, 3693, 3704, 3703

807, 3979, 3980, 3991, 3990, 3693, 3694, 3705, 3704

808, 3980, 3981, 3992, 3991, 3694, 3695, 3706, 3705

809, 3981, 3982, 3993, 3992, 3695, 3696, 3707, 3706

810, 3982, 3983, 3994, 3993, 3696, 3697, 3708, 3707

811, 3983, 3984, 3995, 3994, 3697, 3698, 3709, 3708

812, 3984, 3985, 3996, 3995, 3698, 3699, 3710, 3709

813, 3985, 3986, 3997, 3996, 3699, 3700, 3711, 3710

814, 3986, 3987, 3998, 3997, 3700, 3701, 3712, 3711

815, 3987, 3988, 3999, 3998, 3701, 3702, 3713, 3712

816, 3988, 1308, 1309, 3999, 3702, 1282, 1283, 3713

817, 1179, 3989, 4000, 1180, 1153, 3703, 3714, 1154

818, 3989, 3990, 4001, 4000, 3703, 3704, 3715, 3714

819, 3990, 3991, 4002, 4001, 3704, 3705, 3716, 3715

820, 3991, 3992, 4003, 4002, 3705, 3706, 3717, 3716

821, 3992, 3993, 4004, 4003, 3706, 3707, 3718, 3717

822, 3993, 3994, 4005, 4004, 3707, 3708, 3719, 3718

823, 3994, 3995, 4006, 4005, 3708, 3709, 3720, 3719

824, 3995, 3996, 4007, 4006, 3709, 3710, 3721, 3720

825, 3996, 3997, 4008, 4007, 3710, 3711, 3722, 3721

826, 3997, 3998, 4009, 4008, 3711, 3712, 3723, 3722

827, 3998, 3999, 4010, 4009, 3712, 3713, 3724, 3723

828, 3999, 1309, 1310, 4010, 3713, 1283, 1284, 3724

829, 1180, 4000, 4011, 1181, 1154, 3714, 3725, 1155

830, 4000, 4001, 4012, 4011, 3714, 3715, 3726, 3725

831, 4001, 4002, 4013, 4012, 3715, 3716, 3727, 3726

832, 4002, 4003, 4014, 4013, 3716, 3717, 3728, 3727

833, 4003, 4004, 4015, 4014, 3717, 3718, 3729, 3728

834, 4004, 4005, 4016, 4015, 3718, 3719, 3730, 3729

835, 4005, 4006, 4017, 4016, 3719, 3720, 3731, 3730

836, 4006, 4007, 4018, 4017, 3720, 3721, 3732, 3731

837, 4007, 4008, 4019, 4018, 3721, 3722, 3733, 3732

838, 4008, 4009, 4020, 4019, 3722, 3723, 3734, 3733

839, 4009, 4010, 4021, 4020, 3723, 3724, 3735, 3734

840, 4010, 1310, 1311, 4021, 3724, 1284, 1285, 3735

841, 1181, 4011, 4022, 1182, 1155, 3725, 3736, 1156

842, 4011, 4012, 4023, 4022, 3725, 3726, 3737, 3736

843, 4012, 4013, 4024, 4023, 3726, 3727, 3738, 3737

844, 4013, 4014, 4025, 4024, 3727, 3728, 3739, 3738

845, 4014, 4015, 4026, 4025, 3728, 3729, 3740, 3739

846, 4015, 4016, 4027, 4026, 3729, 3730, 3741, 3740

847, 4016, 4017, 4028, 4027, 3730, 3731, 3742, 3741

848, 4017, 4018, 4029, 4028, 3731, 3732, 3743, 3742

849, 4018, 4019, 4030, 4029, 3732, 3733, 3744, 3743

850, 4019, 4020, 4031, 4030, 3733, 3734, 3745, 3744

851, 4020, 4021, 4032, 4031, 3734, 3735, 3746, 3745

852, 4021, 1311, 1312, 4032, 3735, 1285, 1286, 3746

853, 1182, 4022, 4033, 1183, 1156, 3736, 3747, 1157

854, 4022, 4023, 4034, 4033, 3736, 3737, 3748, 3747

855, 4023, 4024, 4035, 4034, 3737, 3738, 3749, 3748

856, 4024, 4025, 4036, 4035, 3738, 3739, 3750, 3749

857, 4025, 4026, 4037, 4036, 3739, 3740, 3751, 3750

858, 4026, 4027, 4038, 4037, 3740, 3741, 3752, 3751

859, 4027, 4028, 4039, 4038, 3741, 3742, 3753, 3752

860, 4028, 4029, 4040, 4039, 3742, 3743, 3754, 3753

861, 4029, 4030, 4041, 4040, 3743, 3744, 3755, 3754

862, 4030, 4031, 4042, 4041, 3744, 3745, 3756, 3755

863, 4031, 4032, 4043, 4042, 3745, 3746, 3757, 3756

864, 4032, 1312, 1313, 4043, 3746, 1286, 1287, 3757

865, 1183, 4033, 4044, 1184, 1157, 3747, 3758, 1158

866, 4033, 4034, 4045, 4044, 3747, 3748, 3759, 3758

867, 4034, 4035, 4046, 4045, 3748, 3749, 3760, 3759

868, 4035, 4036, 4047, 4046, 3749, 3750, 3761, 3760

869, 4036, 4037, 4048, 4047, 3750, 3751, 3762, 3761

870, 4037, 4038, 4049, 4048, 3751, 3752, 3763, 3762

871, 4038, 4039, 4050, 4049, 3752, 3753, 3764, 3763

872, 4039, 4040, 4051, 4050, 3753, 3754, 3765, 3764

873, 4040, 4041, 4052, 4051, 3754, 3755, 3766, 3765

874, 4041, 4042, 4053, 4052, 3755, 3756, 3767, 3766

875, 4042, 4043, 4054, 4053, 3756, 3757, 3768, 3767

876, 4043, 1313, 1314, 4054, 3757, 1287, 1288, 3768

877, 1184, 4044, 4055, 1185, 1158, 3758, 3769, 1159

878, 4044, 4045, 4056, 4055, 3758, 3759, 3770, 3769

879, 4045, 4046, 4057, 4056, 3759, 3760, 3771, 3770

880, 4046, 4047, 4058, 4057, 3760, 3761, 3772, 3771

881, 4047, 4048, 4059, 4058, 3761, 3762, 3773, 3772

882, 4048, 4049, 4060, 4059, 3762, 3763, 3774, 3773

883, 4049, 4050, 4061, 4060, 3763, 3764, 3775, 3774

884, 4050, 4051, 4062, 4061, 3764, 3765, 3776, 3775

885, 4051, 4052, 4063, 4062, 3765, 3766, 3777, 3776

886, 4052, 4053, 4064, 4063, 3766, 3767, 3778, 3777

887, 4053, 4054, 4065, 4064, 3767, 3768, 3779, 3778

888, 4054, 1314, 1315, 4065, 3768, 1288, 1289, 3779

889, 1185, 4055, 4066, 1186, 1159, 3769, 3780, 1160

890, 4055, 4056, 4067, 4066, 3769, 3770, 3781, 3780

891, 4056, 4057, 4068, 4067, 3770, 3771, 3782, 3781

892, 4057, 4058, 4069, 4068, 3771, 3772, 3783, 3782

893, 4058, 4059, 4070, 4069, 3772, 3773, 3784, 3783

894, 4059, 4060, 4071, 4070, 3773, 3774, 3785, 3784

895, 4060, 4061, 4072, 4071, 3774, 3775, 3786, 3785

896, 4061, 4062, 4073, 4072, 3775, 3776, 3787, 3786

897, 4062, 4063, 4074, 4073, 3776, 3777, 3788, 3787

898, 4063, 4064, 4075, 4074, 3777, 3778, 3789, 3788

899, 4064, 4065, 4076, 4075, 3778, 3779, 3790, 3789

900, 4065, 1315, 1316, 4076, 3779, 1289, 1290, 3790

901, 1186, 4066, 4077, 1187, 1160, 3780, 3791, 1161

902, 4066, 4067, 4078, 4077, 3780, 3781, 3792, 3791

903, 4067, 4068, 4079, 4078, 3781, 3782, 3793, 3792

904, 4068, 4069, 4080, 4079, 3782, 3783, 3794, 3793

905, 4069, 4070, 4081, 4080, 3783, 3784, 3795, 3794

906, 4070, 4071, 4082, 4081, 3784, 3785, 3796, 3795

907, 4071, 4072, 4083, 4082, 3785, 3786, 3797, 3796

908, 4072, 4073, 4084, 4083, 3786, 3787, 3798, 3797

909, 4073, 4074, 4085, 4084, 3787, 3788, 3799, 3798

910, 4074, 4075, 4086, 4085, 3788, 3789, 3800, 3799

911, 4075, 4076, 4087, 4086, 3789, 3790, 3801, 3800

912, 4076, 1316, 1317, 4087, 3790, 1290, 1291, 3801

913, 1187, 4077, 4088, 1188, 1161, 3791, 3802, 1162

914, 4077, 4078, 4089, 4088, 3791, 3792, 3803, 3802

915, 4078, 4079, 4090, 4089, 3792, 3793, 3804, 3803

916, 4079, 4080, 4091, 4090, 3793, 3794, 3805, 3804

917, 4080, 4081, 4092, 4091, 3794, 3795, 3806, 3805

918, 4081, 4082, 4093, 4092, 3795, 3796, 3807, 3806

919, 4082, 4083, 4094, 4093, 3796, 3797, 3808, 3807

920, 4083, 4084, 4095, 4094, 3797, 3798, 3809, 3808

921, 4084, 4085, 4096, 4095, 3798, 3799, 3810, 3809

922, 4085, 4086, 4097, 4096, 3799, 3800, 3811, 3810

923, 4086, 4087, 4098, 4097, 3800, 3801, 3812, 3811

924, 4087, 1317, 1318, 4098, 3801, 1291, 1292, 3812

925, 1188, 4088, 4099, 1189, 1162, 3802, 3813, 1163

926, 4088, 4089, 4100, 4099, 3802, 3803, 3814, 3813

927, 4089, 4090, 4101, 4100, 3803, 3804, 3815, 3814

928, 4090, 4091, 4102, 4101, 3804, 3805, 3816, 3815

929, 4091, 4092, 4103, 4102, 3805, 3806, 3817, 3816

930, 4092, 4093, 4104, 4103, 3806, 3807, 3818, 3817

931, 4093, 4094, 4105, 4104, 3807, 3808, 3819, 3818

932, 4094, 4095, 4106, 4105, 3808, 3809, 3820, 3819

933, 4095, 4096, 4107, 4106, 3809, 3810, 3821, 3820

934, 4096, 4097, 4108, 4107, 3810, 3811, 3822, 3821

935, 4097, 4098, 4109, 4108, 3811, 3812, 3823, 3822

936, 4098, 1318, 1319, 4109, 3812, 1292, 1293, 3823

937, 1189, 4099, 4110, 1190, 1163, 3813, 3824, 1164

938, 4099, 4100, 4111, 4110, 3813, 3814, 3825, 3824

939, 4100, 4101, 4112, 4111, 3814, 3815, 3826, 3825

940, 4101, 4102, 4113, 4112, 3815, 3816, 3827, 3826

941, 4102, 4103, 4114, 4113, 3816, 3817, 3828, 3827

942, 4103, 4104, 4115, 4114, 3817, 3818, 3829, 3828

943, 4104, 4105, 4116, 4115, 3818, 3819, 3830, 3829

944, 4105, 4106, 4117, 4116, 3819, 3820, 3831, 3830

945, 4106, 4107, 4118, 4117, 3820, 3821, 3832, 3831

946, 4107, 4108, 4119, 4118, 3821, 3822, 3833, 3832

947, 4108, 4109, 4120, 4119, 3822, 3823, 3834, 3833

948, 4109, 1319, 1320, 4120, 3823, 1293, 1294, 3834

949, 1190, 4110, 4121, 1191, 1164, 3824, 3835, 1165

950, 4110, 4111, 4122, 4121, 3824, 3825, 3836, 3835

951, 4111, 4112, 4123, 4122, 3825, 3826, 3837, 3836

952, 4112, 4113, 4124, 4123, 3826, 3827, 3838, 3837

953, 4113, 4114, 4125, 4124, 3827, 3828, 3839, 3838

954, 4114, 4115, 4126, 4125, 3828, 3829, 3840, 3839

955, 4115, 4116, 4127, 4126, 3829, 3830, 3841, 3840

956, 4116, 4117, 4128, 4127, 3830, 3831, 3842, 3841

957, 4117, 4118, 4129, 4128, 3831, 3832, 3843, 3842

958, 4118, 4119, 4130, 4129, 3832, 3833, 3844, 3843

959, 4119, 4120, 4131, 4130, 3833, 3834, 3845, 3844

960, 4120, 1320, 1321, 4131, 3834, 1294, 1295, 3845

961, 1191, 4121, 1451, 183, 1165, 3835, 1440, 182

962, 4121, 4122, 1452, 1451, 3835, 3836, 1441, 1440

963, 4122, 4123, 1453, 1452, 3836, 3837, 1442, 1441

964, 4123, 4124, 1454, 1453, 3837, 3838, 1443, 1442

965, 4124, 4125, 1455, 1454, 3838, 3839, 1444, 1443

966, 4125, 4126, 1456, 1455, 3839, 3840, 1445, 1444

967, 4126, 4127, 1457, 1456, 3840, 3841, 1446, 1445

968, 4127, 4128, 1458, 1457, 3841, 3842, 1447, 1446

969, 4128, 4129, 1459, 1458, 3842, 3843, 1448, 1447

970, 4129, 4130, 1460, 1459, 3843, 3844, 1449, 1448

971, 4130, 4131, 1461, 1460, 3844, 3845, 1450, 1449

972, 4131, 1321, 193, 1461, 3845, 1295, 194, 1450

973, 187, 1407, 4132, 1192, 188, 1396, 3846, 1166

974, 1407, 1408, 4133, 4132, 1396, 1397, 3847, 3846

975, 1408, 1409, 4134, 4133, 1397, 1398, 3848, 3847

976, 1409, 1410, 4135, 4134, 1398, 1399, 3849, 3848

977, 1410, 1411, 4136, 4135, 1399, 1400, 3850, 3849

978, 1411, 1412, 4137, 4136, 1400, 1401, 3851, 3850

979, 1412, 1413, 4138, 4137, 1401, 1402, 3852, 3851

980, 1413, 1414, 4139, 4138, 1402, 1403, 3853, 3852

981, 1414, 1415, 4140, 4139, 1403, 1404, 3854, 3853

982, 1415, 1416, 4141, 4140, 1404, 1405, 3855, 3854

983, 1416, 1417, 4142, 4141, 1405, 1406, 3856, 3855

984, 1417, 199, 1322, 4142, 1406, 198, 1296, 3856

985, 1192, 4132, 4143, 1193, 1166, 3846, 3857, 1167

986, 4132, 4133, 4144, 4143, 3846, 3847, 3858, 3857

987, 4133, 4134, 4145, 4144, 3847, 3848, 3859, 3858

988, 4134, 4135, 4146, 4145, 3848, 3849, 3860, 3859

989, 4135, 4136, 4147, 4146, 3849, 3850, 3861, 3860

990, 4136, 4137, 4148, 4147, 3850, 3851, 3862, 3861

991, 4137, 4138, 4149, 4148, 3851, 3852, 3863, 3862

992, 4138, 4139, 4150, 4149, 3852, 3853, 3864, 3863

993, 4139, 4140, 4151, 4150, 3853, 3854, 3865, 3864

994, 4140, 4141, 4152, 4151, 3854, 3855, 3866, 3865

995, 4141, 4142, 4153, 4152, 3855, 3856, 3867, 3866

996, 4142, 1322, 1323, 4153, 3856, 1296, 1297, 3867

997, 1193, 4143, 4154, 1194, 1167, 3857, 3868, 1168

998, 4143, 4144, 4155, 4154, 3857, 3858, 3869, 3868

999, 4144, 4145, 4156, 4155, 3858, 3859, 3870, 3869

1000, 4145, 4146, 4157, 4156, 3859, 3860, 3871, 3870

1001, 4146, 4147, 4158, 4157, 3860, 3861, 3872, 3871

1002, 4147, 4148, 4159, 4158, 3861, 3862, 3873, 3872

1003, 4148, 4149, 4160, 4159, 3862, 3863, 3874, 3873

1004, 4149, 4150, 4161, 4160, 3863, 3864, 3875, 3874

1005, 4150, 4151, 4162, 4161, 3864, 3865, 3876, 3875

1006, 4151, 4152, 4163, 4162, 3865, 3866, 3877, 3876

1007, 4152, 4153, 4164, 4163, 3866, 3867, 3878, 3877

1008, 4153, 1323, 1324, 4164, 3867, 1297, 1298, 3878

1009, 1194, 4154, 4165, 1195, 1168, 3868, 3879, 1169

1010, 4154, 4155, 4166, 4165, 3868, 3869, 3880, 3879

1011, 4155, 4156, 4167, 4166, 3869, 3870, 3881, 3880

1012, 4156, 4157, 4168, 4167, 3870, 3871, 3882, 3881

1013, 4157, 4158, 4169, 4168, 3871, 3872, 3883, 3882

1014, 4158, 4159, 4170, 4169, 3872, 3873, 3884, 3883

1015, 4159, 4160, 4171, 4170, 3873, 3874, 3885, 3884

1016, 4160, 4161, 4172, 4171, 3874, 3875, 3886, 3885

1017, 4161, 4162, 4173, 4172, 3875, 3876, 3887, 3886

1018, 4162, 4163, 4174, 4173, 3876, 3877, 3888, 3887

1019, 4163, 4164, 4175, 4174, 3877, 3878, 3889, 3888

1020, 4164, 1324, 1325, 4175, 3878, 1298, 1299, 3889

1021, 1195, 4165, 4176, 1196, 1169, 3879, 3890, 1170

1022, 4165, 4166, 4177, 4176, 3879, 3880, 3891, 3890

1023, 4166, 4167, 4178, 4177, 3880, 3881, 3892, 3891

1024, 4167, 4168, 4179, 4178, 3881, 3882, 3893, 3892

1025, 4168, 4169, 4180, 4179, 3882, 3883, 3894, 3893

1026, 4169, 4170, 4181, 4180, 3883, 3884, 3895, 3894

1027, 4170, 4171, 4182, 4181, 3884, 3885, 3896, 3895

1028, 4171, 4172, 4183, 4182, 3885, 3886, 3897, 3896

1029, 4172, 4173, 4184, 4183, 3886, 3887, 3898, 3897

1030, 4173, 4174, 4185, 4184, 3887, 3888, 3899, 3898

1031, 4174, 4175, 4186, 4185, 3888, 3889, 3900, 3899

1032, 4175, 1325, 1326, 4186, 3889, 1299, 1300, 3900

1033, 1196, 4176, 4187, 1197, 1170, 3890, 3901, 1171

1034, 4176, 4177, 4188, 4187, 3890, 3891, 3902, 3901

1035, 4177, 4178, 4189, 4188, 3891, 3892, 3903, 3902

1036, 4178, 4179, 4190, 4189, 3892, 3893, 3904, 3903

1037, 4179, 4180, 4191, 4190, 3893, 3894, 3905, 3904

1038, 4180, 4181, 4192, 4191, 3894, 3895, 3906, 3905

1039, 4181, 4182, 4193, 4192, 3895, 3896, 3907, 3906

1040, 4182, 4183, 4194, 4193, 3896, 3897, 3908, 3907

1041, 4183, 4184, 4195, 4194, 3897, 3898, 3909, 3908

1042, 4184, 4185, 4196, 4195, 3898, 3899, 3910, 3909

1043, 4185, 4186, 4197, 4196, 3899, 3900, 3911, 3910

1044, 4186, 1326, 1327, 4197, 3900, 1300, 1301, 3911

1045, 1197, 4187, 4198, 1198, 1171, 3901, 3912, 1172

1046, 4187, 4188, 4199, 4198, 3901, 3902, 3913, 3912

1047, 4188, 4189, 4200, 4199, 3902, 3903, 3914, 3913

1048, 4189, 4190, 4201, 4200, 3903, 3904, 3915, 3914

1049, 4190, 4191, 4202, 4201, 3904, 3905, 3916, 3915

1050, 4191, 4192, 4203, 4202, 3905, 3906, 3917, 3916

1051, 4192, 4193, 4204, 4203, 3906, 3907, 3918, 3917

1052, 4193, 4194, 4205, 4204, 3907, 3908, 3919, 3918

1053, 4194, 4195, 4206, 4205, 3908, 3909, 3920, 3919

1054, 4195, 4196, 4207, 4206, 3909, 3910, 3921, 3920

1055, 4196, 4197, 4208, 4207, 3910, 3911, 3922, 3921

1056, 4197, 1327, 1328, 4208, 3911, 1301, 1302, 3922

1057, 1198, 4198, 4209, 1199, 1172, 3912, 3923, 1173

1058, 4198, 4199, 4210, 4209, 3912, 3913, 3924, 3923

1059, 4199, 4200, 4211, 4210, 3913, 3914, 3925, 3924

1060, 4200, 4201, 4212, 4211, 3914, 3915, 3926, 3925

1061, 4201, 4202, 4213, 4212, 3915, 3916, 3927, 3926

1062, 4202, 4203, 4214, 4213, 3916, 3917, 3928, 3927

1063, 4203, 4204, 4215, 4214, 3917, 3918, 3929, 3928

1064, 4204, 4205, 4216, 4215, 3918, 3919, 3930, 3929

1065, 4205, 4206, 4217, 4216, 3919, 3920, 3931, 3930

1066, 4206, 4207, 4218, 4217, 3920, 3921, 3932, 3931

1067, 4207, 4208, 4219, 4218, 3921, 3922, 3933, 3932

1068, 4208, 1328, 1329, 4219, 3922, 1302, 1303, 3933

1069, 1199, 4209, 4220, 1200, 1173, 3923, 3934, 1174

1070, 4209, 4210, 4221, 4220, 3923, 3924, 3935, 3934

1071, 4210, 4211, 4222, 4221, 3924, 3925, 3936, 3935

1072, 4211, 4212, 4223, 4222, 3925, 3926, 3937, 3936

1073, 4212, 4213, 4224, 4223, 3926, 3927, 3938, 3937

1074, 4213, 4214, 4225, 4224, 3927, 3928, 3939, 3938

1075, 4214, 4215, 4226, 4225, 3928, 3929, 3940, 3939

1076, 4215, 4216, 4227, 4226, 3929, 3930, 3941, 3940

1077, 4216, 4217, 4228, 4227, 3930, 3931, 3942, 3941

1078, 4217, 4218, 4229, 4228, 3931, 3932, 3943, 3942

1079, 4218, 4219, 4230, 4229, 3932, 3933, 3944, 3943

1080, 4219, 1329, 1330, 4230, 3933, 1303, 1304, 3944

1081, 1200, 4220, 4231, 1201, 1174, 3934, 3945, 1175

1082, 4220, 4221, 4232, 4231, 3934, 3935, 3946, 3945

1083, 4221, 4222, 4233, 4232, 3935, 3936, 3947, 3946

1084, 4222, 4223, 4234, 4233, 3936, 3937, 3948, 3947

1085, 4223, 4224, 4235, 4234, 3937, 3938, 3949, 3948

1086, 4224, 4225, 4236, 4235, 3938, 3939, 3950, 3949

1087, 4225, 4226, 4237, 4236, 3939, 3940, 3951, 3950

1088, 4226, 4227, 4238, 4237, 3940, 3941, 3952, 3951

1089, 4227, 4228, 4239, 4238, 3941, 3942, 3953, 3952

1090, 4228, 4229, 4240, 4239, 3942, 3943, 3954, 3953

1091, 4229, 4230, 4241, 4240, 3943, 3944, 3955, 3954

1092, 4230, 1330, 1331, 4241, 3944, 1304, 1305, 3955

1093, 1201, 4231, 4242, 1202, 1175, 3945, 3956, 1176

1094, 4231, 4232, 4243, 4242, 3945, 3946, 3957, 3956

1095, 4232, 4233, 4244, 4243, 3946, 3947, 3958, 3957

1096, 4233, 4234, 4245, 4244, 3947, 3948, 3959, 3958

1097, 4234, 4235, 4246, 4245, 3948, 3949, 3960, 3959

1098, 4235, 4236, 4247, 4246, 3949, 3950, 3961, 3960

1099, 4236, 4237, 4248, 4247, 3950, 3951, 3962, 3961

1100, 4237, 4238, 4249, 4248, 3951, 3952, 3963, 3962

1101, 4238, 4239, 4250, 4249, 3952, 3953, 3964, 3963

1102, 4239, 4240, 4251, 4250, 3953, 3954, 3965, 3964

1103, 4240, 4241, 4252, 4251, 3954, 3955, 3966, 3965

1104, 4241, 1331, 1332, 4252, 3955, 1305, 1306, 3966

1105, 1202, 4242, 4253, 1203, 1176, 3956, 3967, 1177

1106, 4242, 4243, 4254, 4253, 3956, 3957, 3968, 3967

1107, 4243, 4244, 4255, 4254, 3957, 3958, 3969, 3968

1108, 4244, 4245, 4256, 4255, 3958, 3959, 3970, 3969

1109, 4245, 4246, 4257, 4256, 3959, 3960, 3971, 3970

1110, 4246, 4247, 4258, 4257, 3960, 3961, 3972, 3971

1111, 4247, 4248, 4259, 4258, 3961, 3962, 3973, 3972

1112, 4248, 4249, 4260, 4259, 3962, 3963, 3974, 3973

1113, 4249, 4250, 4261, 4260, 3963, 3964, 3975, 3974

1114, 4250, 4251, 4262, 4261, 3964, 3965, 3976, 3975

1115, 4251, 4252, 4263, 4262, 3965, 3966, 3977, 3976

1116, 4252, 1332, 1333, 4263, 3966, 1306, 1307, 3977

1117, 1203, 4253, 4264, 1204, 1177, 3967, 3978, 1178

1118, 4253, 4254, 4265, 4264, 3967, 3968, 3979, 3978

1119, 4254, 4255, 4266, 4265, 3968, 3969, 3980, 3979

1120, 4255, 4256, 4267, 4266, 3969, 3970, 3981, 3980

1121, 4256, 4257, 4268, 4267, 3970, 3971, 3982, 3981

1122, 4257, 4258, 4269, 4268, 3971, 3972, 3983, 3982

1123, 4258, 4259, 4270, 4269, 3972, 3973, 3984, 3983

1124, 4259, 4260, 4271, 4270, 3973, 3974, 3985, 3984

1125, 4260, 4261, 4272, 4271, 3974, 3975, 3986, 3985

1126, 4261, 4262, 4273, 4272, 3975, 3976, 3987, 3986

1127, 4262, 4263, 4274, 4273, 3976, 3977, 3988, 3987

1128, 4263, 1333, 1334, 4274, 3977, 1307, 1308, 3988

1129, 1204, 4264, 4275, 1205, 1178, 3978, 3989, 1179

1130, 4264, 4265, 4276, 4275, 3978, 3979, 3990, 3989

1131, 4265, 4266, 4277, 4276, 3979, 3980, 3991, 3990

1132, 4266, 4267, 4278, 4277, 3980, 3981, 3992, 3991

1133, 4267, 4268, 4279, 4278, 3981, 3982, 3993, 3992

1134, 4268, 4269, 4280, 4279, 3982, 3983, 3994, 3993

1135, 4269, 4270, 4281, 4280, 3983, 3984, 3995, 3994

1136, 4270, 4271, 4282, 4281, 3984, 3985, 3996, 3995

1137, 4271, 4272, 4283, 4282, 3985, 3986, 3997, 3996

1138, 4272, 4273, 4284, 4283, 3986, 3987, 3998, 3997

1139, 4273, 4274, 4285, 4284, 3987, 3988, 3999, 3998

1140, 4274, 1334, 1335, 4285, 3988, 1308, 1309, 3999

1141, 1205, 4275, 4286, 1206, 1179, 3989, 4000, 1180

1142, 4275, 4276, 4287, 4286, 3989, 3990, 4001, 4000

1143, 4276, 4277, 4288, 4287, 3990, 3991, 4002, 4001

1144, 4277, 4278, 4289, 4288, 3991, 3992, 4003, 4002

1145, 4278, 4279, 4290, 4289, 3992, 3993, 4004, 4003

1146, 4279, 4280, 4291, 4290, 3993, 3994, 4005, 4004

1147, 4280, 4281, 4292, 4291, 3994, 3995, 4006, 4005

1148, 4281, 4282, 4293, 4292, 3995, 3996, 4007, 4006

1149, 4282, 4283, 4294, 4293, 3996, 3997, 4008, 4007

1150, 4283, 4284, 4295, 4294, 3997, 3998, 4009, 4008

1151, 4284, 4285, 4296, 4295, 3998, 3999, 4010, 4009

1152, 4285, 1335, 1336, 4296, 3999, 1309, 1310, 4010

1153, 1206, 4286, 4297, 1207, 1180, 4000, 4011, 1181

1154, 4286, 4287, 4298, 4297, 4000, 4001, 4012, 4011

1155, 4287, 4288, 4299, 4298, 4001, 4002, 4013, 4012

1156, 4288, 4289, 4300, 4299, 4002, 4003, 4014, 4013

1157, 4289, 4290, 4301, 4300, 4003, 4004, 4015, 4014

1158, 4290, 4291, 4302, 4301, 4004, 4005, 4016, 4015

1159, 4291, 4292, 4303, 4302, 4005, 4006, 4017, 4016

1160, 4292, 4293, 4304, 4303, 4006, 4007, 4018, 4017

1161, 4293, 4294, 4305, 4304, 4007, 4008, 4019, 4018

1162, 4294, 4295, 4306, 4305, 4008, 4009, 4020, 4019

1163, 4295, 4296, 4307, 4306, 4009, 4010, 4021, 4020

1164, 4296, 1336, 1337, 4307, 4010, 1310, 1311, 4021

1165, 1207, 4297, 4308, 1208, 1181, 4011, 4022, 1182

1166, 4297, 4298, 4309, 4308, 4011, 4012, 4023, 4022

1167, 4298, 4299, 4310, 4309, 4012, 4013, 4024, 4023

1168, 4299, 4300, 4311, 4310, 4013, 4014, 4025, 4024

1169, 4300, 4301, 4312, 4311, 4014, 4015, 4026, 4025

1170, 4301, 4302, 4313, 4312, 4015, 4016, 4027, 4026

1171, 4302, 4303, 4314, 4313, 4016, 4017, 4028, 4027

1172, 4303, 4304, 4315, 4314, 4017, 4018, 4029, 4028

1173, 4304, 4305, 4316, 4315, 4018, 4019, 4030, 4029

1174, 4305, 4306, 4317, 4316, 4019, 4020, 4031, 4030

1175, 4306, 4307, 4318, 4317, 4020, 4021, 4032, 4031

1176, 4307, 1337, 1338, 4318, 4021, 1311, 1312, 4032

1177, 1208, 4308, 4319, 1209, 1182, 4022, 4033, 1183

1178, 4308, 4309, 4320, 4319, 4022, 4023, 4034, 4033

1179, 4309, 4310, 4321, 4320, 4023, 4024, 4035, 4034

1180, 4310, 4311, 4322, 4321, 4024, 4025, 4036, 4035

1181, 4311, 4312, 4323, 4322, 4025, 4026, 4037, 4036

1182, 4312, 4313, 4324, 4323, 4026, 4027, 4038, 4037

1183, 4313, 4314, 4325, 4324, 4027, 4028, 4039, 4038

1184, 4314, 4315, 4326, 4325, 4028, 4029, 4040, 4039

1185, 4315, 4316, 4327, 4326, 4029, 4030, 4041, 4040

1186, 4316, 4317, 4328, 4327, 4030, 4031, 4042, 4041

1187, 4317, 4318, 4329, 4328, 4031, 4032, 4043, 4042

1188, 4318, 1338, 1339, 4329, 4032, 1312, 1313, 4043

1189, 1209, 4319, 4330, 1210, 1183, 4033, 4044, 1184

1190, 4319, 4320, 4331, 4330, 4033, 4034, 4045, 4044

1191, 4320, 4321, 4332, 4331, 4034, 4035, 4046, 4045

1192, 4321, 4322, 4333, 4332, 4035, 4036, 4047, 4046

1193, 4322, 4323, 4334, 4333, 4036, 4037, 4048, 4047

1194, 4323, 4324, 4335, 4334, 4037, 4038, 4049, 4048

1195, 4324, 4325, 4336, 4335, 4038, 4039, 4050, 4049

1196, 4325, 4326, 4337, 4336, 4039, 4040, 4051, 4050

1197, 4326, 4327, 4338, 4337, 4040, 4041, 4052, 4051

1198, 4327, 4328, 4339, 4338, 4041, 4042, 4053, 4052

1199, 4328, 4329, 4340, 4339, 4042, 4043, 4054, 4053

1200, 4329, 1339, 1340, 4340, 4043, 1313, 1314, 4054

1201, 1210, 4330, 4341, 1211, 1184, 4044, 4055, 1185

1202, 4330, 4331, 4342, 4341, 4044, 4045, 4056, 4055

1203, 4331, 4332, 4343, 4342, 4045, 4046, 4057, 4056

1204, 4332, 4333, 4344, 4343, 4046, 4047, 4058, 4057

1205, 4333, 4334, 4345, 4344, 4047, 4048, 4059, 4058

1206, 4334, 4335, 4346, 4345, 4048, 4049, 4060, 4059

1207, 4335, 4336, 4347, 4346, 4049, 4050, 4061, 4060

1208, 4336, 4337, 4348, 4347, 4050, 4051, 4062, 4061

1209, 4337, 4338, 4349, 4348, 4051, 4052, 4063, 4062

1210, 4338, 4339, 4350, 4349, 4052, 4053, 4064, 4063

1211, 4339, 4340, 4351, 4350, 4053, 4054, 4065, 4064

1212, 4340, 1340, 1341, 4351, 4054, 1314, 1315, 4065

1213, 1211, 4341, 4352, 1212, 1185, 4055, 4066, 1186

1214, 4341, 4342, 4353, 4352, 4055, 4056, 4067, 4066

1215, 4342, 4343, 4354, 4353, 4056, 4057, 4068, 4067

1216, 4343, 4344, 4355, 4354, 4057, 4058, 4069, 4068

1217, 4344, 4345, 4356, 4355, 4058, 4059, 4070, 4069

1218, 4345, 4346, 4357, 4356, 4059, 4060, 4071, 4070

1219, 4346, 4347, 4358, 4357, 4060, 4061, 4072, 4071

1220, 4347, 4348, 4359, 4358, 4061, 4062, 4073, 4072

1221, 4348, 4349, 4360, 4359, 4062, 4063, 4074, 4073

1222, 4349, 4350, 4361, 4360, 4063, 4064, 4075, 4074

1223, 4350, 4351, 4362, 4361, 4064, 4065, 4076, 4075

1224, 4351, 1341, 1342, 4362, 4065, 1315, 1316, 4076

1225, 1212, 4352, 4363, 1213, 1186, 4066, 4077, 1187

1226, 4352, 4353, 4364, 4363, 4066, 4067, 4078, 4077

1227, 4353, 4354, 4365, 4364, 4067, 4068, 4079, 4078

1228, 4354, 4355, 4366, 4365, 4068, 4069, 4080, 4079

1229, 4355, 4356, 4367, 4366, 4069, 4070, 4081, 4080

1230, 4356, 4357, 4368, 4367, 4070, 4071, 4082, 4081

1231, 4357, 4358, 4369, 4368, 4071, 4072, 4083, 4082

1232, 4358, 4359, 4370, 4369, 4072, 4073, 4084, 4083

1233, 4359, 4360, 4371, 4370, 4073, 4074, 4085, 4084

1234, 4360, 4361, 4372, 4371, 4074, 4075, 4086, 4085

1235, 4361, 4362, 4373, 4372, 4075, 4076, 4087, 4086

1236, 4362, 1342, 1343, 4373, 4076, 1316, 1317, 4087

1237, 1213, 4363, 4374, 1214, 1187, 4077, 4088, 1188

1238, 4363, 4364, 4375, 4374, 4077, 4078, 4089, 4088

1239, 4364, 4365, 4376, 4375, 4078, 4079, 4090, 4089

1240, 4365, 4366, 4377, 4376, 4079, 4080, 4091, 4090

1241, 4366, 4367, 4378, 4377, 4080, 4081, 4092, 4091

1242, 4367, 4368, 4379, 4378, 4081, 4082, 4093, 4092

1243, 4368, 4369, 4380, 4379, 4082, 4083, 4094, 4093

1244, 4369, 4370, 4381, 4380, 4083, 4084, 4095, 4094

1245, 4370, 4371, 4382, 4381, 4084, 4085, 4096, 4095

1246, 4371, 4372, 4383, 4382, 4085, 4086, 4097, 4096

1247, 4372, 4373, 4384, 4383, 4086, 4087, 4098, 4097

1248, 4373, 1343, 1344, 4384, 4087, 1317, 1318, 4098

1249, 1214, 4374, 4385, 1215, 1188, 4088, 4099, 1189

1250, 4374, 4375, 4386, 4385, 4088, 4089, 4100, 4099

1251, 4375, 4376, 4387, 4386, 4089, 4090, 4101, 4100

1252, 4376, 4377, 4388, 4387, 4090, 4091, 4102, 4101

1253, 4377, 4378, 4389, 4388, 4091, 4092, 4103, 4102

1254, 4378, 4379, 4390, 4389, 4092, 4093, 4104, 4103

1255, 4379, 4380, 4391, 4390, 4093, 4094, 4105, 4104

1256, 4380, 4381, 4392, 4391, 4094, 4095, 4106, 4105

1257, 4381, 4382, 4393, 4392, 4095, 4096, 4107, 4106

1258, 4382, 4383, 4394, 4393, 4096, 4097, 4108, 4107

1259, 4383, 4384, 4395, 4394, 4097, 4098, 4109, 4108

1260, 4384, 1344, 1345, 4395, 4098, 1318, 1319, 4109

1261, 1215, 4385, 4396, 1216, 1189, 4099, 4110, 1190

1262, 4385, 4386, 4397, 4396, 4099, 4100, 4111, 4110

1263, 4386, 4387, 4398, 4397, 4100, 4101, 4112, 4111

1264, 4387, 4388, 4399, 4398, 4101, 4102, 4113, 4112

1265, 4388, 4389, 4400, 4399, 4102, 4103, 4114, 4113

1266, 4389, 4390, 4401, 4400, 4103, 4104, 4115, 4114

1267, 4390, 4391, 4402, 4401, 4104, 4105, 4116, 4115

1268, 4391, 4392, 4403, 4402, 4105, 4106, 4117, 4116

1269, 4392, 4393, 4404, 4403, 4106, 4107, 4118, 4117

1270, 4393, 4394, 4405, 4404, 4107, 4108, 4119, 4118

1271, 4394, 4395, 4406, 4405, 4108, 4109, 4120, 4119

1272, 4395, 1345, 1346, 4406, 4109, 1319, 1320, 4120

1273, 1216, 4396, 4407, 1217, 1190, 4110, 4121, 1191

1274, 4396, 4397, 4408, 4407, 4110, 4111, 4122, 4121

1275, 4397, 4398, 4409, 4408, 4111, 4112, 4123, 4122

1276, 4398, 4399, 4410, 4409, 4112, 4113, 4124, 4123

1277, 4399, 4400, 4411, 4410, 4113, 4114, 4125, 4124

1278, 4400, 4401, 4412, 4411, 4114, 4115, 4126, 4125

1279, 4401, 4402, 4413, 4412, 4115, 4116, 4127, 4126

1280, 4402, 4403, 4414, 4413, 4116, 4117, 4128, 4127

1281, 4403, 4404, 4415, 4414, 4117, 4118, 4129, 4128

1282, 4404, 4405, 4416, 4415, 4118, 4119, 4130, 4129

1283, 4405, 4406, 4417, 4416, 4119, 4120, 4131, 4130

1284, 4406, 1346, 1347, 4417, 4120, 1320, 1321, 4131

1285, 1217, 4407, 1462, 184, 1191, 4121, 1451, 183

1286, 4407, 4408, 1463, 1462, 4121, 4122, 1452, 1451

1287, 4408, 4409, 1464, 1463, 4122, 4123, 1453, 1452

1288, 4409, 4410, 1465, 1464, 4123, 4124, 1454, 1453

1289, 4410, 4411, 1466, 1465, 4124, 4125, 1455, 1454

1290, 4411, 4412, 1467, 1466, 4125, 4126, 1456, 1455

1291, 4412, 4413, 1468, 1467, 4126, 4127, 1457, 1456

1292, 4413, 4414, 1469, 1468, 4127, 4128, 1458, 1457

1293, 4414, 4415, 1470, 1469, 4128, 4129, 1459, 1458

1294, 4415, 4416, 1471, 1470, 4129, 4130, 1460, 1459

1295, 4416, 4417, 1472, 1471, 4130, 4131, 1461, 1460

1296, 4417, 1347, 192, 1472, 4131, 1321, 193, 1461

1297, 186, 1418, 4418, 1218, 187, 1407, 4132, 1192

1298, 1418, 1419, 4419, 4418, 1407, 1408, 4133, 4132

1299, 1419, 1420, 4420, 4419, 1408, 1409, 4134, 4133

1300, 1420, 1421, 4421, 4420, 1409, 1410, 4135, 4134

1301, 1421, 1422, 4422, 4421, 1410, 1411, 4136, 4135

1302, 1422, 1423, 4423, 4422, 1411, 1412, 4137, 4136

1303, 1423, 1424, 4424, 4423, 1412, 1413, 4138, 4137

1304, 1424, 1425, 4425, 4424, 1413, 1414, 4139, 4138

1305, 1425, 1426, 4426, 4425, 1414, 1415, 4140, 4139

1306, 1426, 1427, 4427, 4426, 1415, 1416, 4141, 4140

1307, 1427, 1428, 4428, 4427, 1416, 1417, 4142, 4141

1308, 1428, 200, 1348, 4428, 1417, 199, 1322, 4142

1309, 1218, 4418, 4429, 1219, 1192, 4132, 4143, 1193

1310, 4418, 4419, 4430, 4429, 4132, 4133, 4144, 4143

1311, 4419, 4420, 4431, 4430, 4133, 4134, 4145, 4144

1312, 4420, 4421, 4432, 4431, 4134, 4135, 4146, 4145

1313, 4421, 4422, 4433, 4432, 4135, 4136, 4147, 4146

1314, 4422, 4423, 4434, 4433, 4136, 4137, 4148, 4147

1315, 4423, 4424, 4435, 4434, 4137, 4138, 4149, 4148

1316, 4424, 4425, 4436, 4435, 4138, 4139, 4150, 4149

1317, 4425, 4426, 4437, 4436, 4139, 4140, 4151, 4150

1318, 4426, 4427, 4438, 4437, 4140, 4141, 4152, 4151

1319, 4427, 4428, 4439, 4438, 4141, 4142, 4153, 4152

1320, 4428, 1348, 1349, 4439, 4142, 1322, 1323, 4153

1321, 1219, 4429, 4440, 1220, 1193, 4143, 4154, 1194

1322, 4429, 4430, 4441, 4440, 4143, 4144, 4155, 4154

1323, 4430, 4431, 4442, 4441, 4144, 4145, 4156, 4155

1324, 4431, 4432, 4443, 4442, 4145, 4146, 4157, 4156

1325, 4432, 4433, 4444, 4443, 4146, 4147, 4158, 4157

1326, 4433, 4434, 4445, 4444, 4147, 4148, 4159, 4158

1327, 4434, 4435, 4446, 4445, 4148, 4149, 4160, 4159

1328, 4435, 4436, 4447, 4446, 4149, 4150, 4161, 4160

1329, 4436, 4437, 4448, 4447, 4150, 4151, 4162, 4161

1330, 4437, 4438, 4449, 4448, 4151, 4152, 4163, 4162

1331, 4438, 4439, 4450, 4449, 4152, 4153, 4164, 4163

1332, 4439, 1349, 1350, 4450, 4153, 1323, 1324, 4164

1333, 1220, 4440, 4451, 1221, 1194, 4154, 4165, 1195

1334, 4440, 4441, 4452, 4451, 4154, 4155, 4166, 4165

1335, 4441, 4442, 4453, 4452, 4155, 4156, 4167, 4166

1336, 4442, 4443, 4454, 4453, 4156, 4157, 4168, 4167

1337, 4443, 4444, 4455, 4454, 4157, 4158, 4169, 4168

1338, 4444, 4445, 4456, 4455, 4158, 4159, 4170, 4169

1339, 4445, 4446, 4457, 4456, 4159, 4160, 4171, 4170

1340, 4446, 4447, 4458, 4457, 4160, 4161, 4172, 4171

1341, 4447, 4448, 4459, 4458, 4161, 4162, 4173, 4172

1342, 4448, 4449, 4460, 4459, 4162, 4163, 4174, 4173

1343, 4449, 4450, 4461, 4460, 4163, 4164, 4175, 4174

1344, 4450, 1350, 1351, 4461, 4164, 1324, 1325, 4175

1345, 1221, 4451, 4462, 1222, 1195, 4165, 4176, 1196

1346, 4451, 4452, 4463, 4462, 4165, 4166, 4177, 4176

1347, 4452, 4453, 4464, 4463, 4166, 4167, 4178, 4177

1348, 4453, 4454, 4465, 4464, 4167, 4168, 4179, 4178

1349, 4454, 4455, 4466, 4465, 4168, 4169, 4180, 4179

1350, 4455, 4456, 4467, 4466, 4169, 4170, 4181, 4180

1351, 4456, 4457, 4468, 4467, 4170, 4171, 4182, 4181

1352, 4457, 4458, 4469, 4468, 4171, 4172, 4183, 4182

1353, 4458, 4459, 4470, 4469, 4172, 4173, 4184, 4183

1354, 4459, 4460, 4471, 4470, 4173, 4174, 4185, 4184

1355, 4460, 4461, 4472, 4471, 4174, 4175, 4186, 4185

1356, 4461, 1351, 1352, 4472, 4175, 1325, 1326, 4186

1357, 1222, 4462, 4473, 1223, 1196, 4176, 4187, 1197

1358, 4462, 4463, 4474, 4473, 4176, 4177, 4188, 4187

1359, 4463, 4464, 4475, 4474, 4177, 4178, 4189, 4188

1360, 4464, 4465, 4476, 4475, 4178, 4179, 4190, 4189

1361, 4465, 4466, 4477, 4476, 4179, 4180, 4191, 4190

1362, 4466, 4467, 4478, 4477, 4180, 4181, 4192, 4191

1363, 4467, 4468, 4479, 4478, 4181, 4182, 4193, 4192

1364, 4468, 4469, 4480, 4479, 4182, 4183, 4194, 4193

1365, 4469, 4470, 4481, 4480, 4183, 4184, 4195, 4194

1366, 4470, 4471, 4482, 4481, 4184, 4185, 4196, 4195

1367, 4471, 4472, 4483, 4482, 4185, 4186, 4197, 4196

1368, 4472, 1352, 1353, 4483, 4186, 1326, 1327, 4197

1369, 1223, 4473, 4484, 1224, 1197, 4187, 4198, 1198

1370, 4473, 4474, 4485, 4484, 4187, 4188, 4199, 4198

1371, 4474, 4475, 4486, 4485, 4188, 4189, 4200, 4199

1372, 4475, 4476, 4487, 4486, 4189, 4190, 4201, 4200

1373, 4476, 4477, 4488, 4487, 4190, 4191, 4202, 4201

1374, 4477, 4478, 4489, 4488, 4191, 4192, 4203, 4202

1375, 4478, 4479, 4490, 4489, 4192, 4193, 4204, 4203

1376, 4479, 4480, 4491, 4490, 4193, 4194, 4205, 4204

1377, 4480, 4481, 4492, 4491, 4194, 4195, 4206, 4205

1378, 4481, 4482, 4493, 4492, 4195, 4196, 4207, 4206

1379, 4482, 4483, 4494, 4493, 4196, 4197, 4208, 4207

1380, 4483, 1353, 1354, 4494, 4197, 1327, 1328, 4208

1381, 1224, 4484, 4495, 1225, 1198, 4198, 4209, 1199

1382, 4484, 4485, 4496, 4495, 4198, 4199, 4210, 4209

1383, 4485, 4486, 4497, 4496, 4199, 4200, 4211, 4210

1384, 4486, 4487, 4498, 4497, 4200, 4201, 4212, 4211

1385, 4487, 4488, 4499, 4498, 4201, 4202, 4213, 4212

1386, 4488, 4489, 4500, 4499, 4202, 4203, 4214, 4213

1387, 4489, 4490, 4501, 4500, 4203, 4204, 4215, 4214

1388, 4490, 4491, 4502, 4501, 4204, 4205, 4216, 4215

1389, 4491, 4492, 4503, 4502, 4205, 4206, 4217, 4216

1390, 4492, 4493, 4504, 4503, 4206, 4207, 4218, 4217

1391, 4493, 4494, 4505, 4504, 4207, 4208, 4219, 4218

1392, 4494, 1354, 1355, 4505, 4208, 1328, 1329, 4219

1393, 1225, 4495, 4506, 1226, 1199, 4209, 4220, 1200

1394, 4495, 4496, 4507, 4506, 4209, 4210, 4221, 4220

1395, 4496, 4497, 4508, 4507, 4210, 4211, 4222, 4221

1396, 4497, 4498, 4509, 4508, 4211, 4212, 4223, 4222

1397, 4498, 4499, 4510, 4509, 4212, 4213, 4224, 4223

1398, 4499, 4500, 4511, 4510, 4213, 4214, 4225, 4224

1399, 4500, 4501, 4512, 4511, 4214, 4215, 4226, 4225

1400, 4501, 4502, 4513, 4512, 4215, 4216, 4227, 4226

1401, 4502, 4503, 4514, 4513, 4216, 4217, 4228, 4227

1402, 4503, 4504, 4515, 4514, 4217, 4218, 4229, 4228

1403, 4504, 4505, 4516, 4515, 4218, 4219, 4230, 4229

1404, 4505, 1355, 1356, 4516, 4219, 1329, 1330, 4230

1405, 1226, 4506, 4517, 1227, 1200, 4220, 4231, 1201

1406, 4506, 4507, 4518, 4517, 4220, 4221, 4232, 4231

1407, 4507, 4508, 4519, 4518, 4221, 4222, 4233, 4232

1408, 4508, 4509, 4520, 4519, 4222, 4223, 4234, 4233

1409, 4509, 4510, 4521, 4520, 4223, 4224, 4235, 4234

1410, 4510, 4511, 4522, 4521, 4224, 4225, 4236, 4235

1411, 4511, 4512, 4523, 4522, 4225, 4226, 4237, 4236

1412, 4512, 4513, 4524, 4523, 4226, 4227, 4238, 4237

1413, 4513, 4514, 4525, 4524, 4227, 4228, 4239, 4238

1414, 4514, 4515, 4526, 4525, 4228, 4229, 4240, 4239

1415, 4515, 4516, 4527, 4526, 4229, 4230, 4241, 4240

1416, 4516, 1356, 1357, 4527, 4230, 1330, 1331, 4241

1417, 1227, 4517, 4528, 1228, 1201, 4231, 4242, 1202

1418, 4517, 4518, 4529, 4528, 4231, 4232, 4243, 4242

1419, 4518, 4519, 4530, 4529, 4232, 4233, 4244, 4243

1420, 4519, 4520, 4531, 4530, 4233, 4234, 4245, 4244

1421, 4520, 4521, 4532, 4531, 4234, 4235, 4246, 4245

1422, 4521, 4522, 4533, 4532, 4235, 4236, 4247, 4246

1423, 4522, 4523, 4534, 4533, 4236, 4237, 4248, 4247

1424, 4523, 4524, 4535, 4534, 4237, 4238, 4249, 4248

1425, 4524, 4525, 4536, 4535, 4238, 4239, 4250, 4249

1426, 4525, 4526, 4537, 4536, 4239, 4240, 4251, 4250

1427, 4526, 4527, 4538, 4537, 4240, 4241, 4252, 4251

1428, 4527, 1357, 1358, 4538, 4241, 1331, 1332, 4252

1429, 1228, 4528, 4539, 1229, 1202, 4242, 4253, 1203

1430, 4528, 4529, 4540, 4539, 4242, 4243, 4254, 4253

1431, 4529, 4530, 4541, 4540, 4243, 4244, 4255, 4254

1432, 4530, 4531, 4542, 4541, 4244, 4245, 4256, 4255

1433, 4531, 4532, 4543, 4542, 4245, 4246, 4257, 4256

1434, 4532, 4533, 4544, 4543, 4246, 4247, 4258, 4257

1435, 4533, 4534, 4545, 4544, 4247, 4248, 4259, 4258

1436, 4534, 4535, 4546, 4545, 4248, 4249, 4260, 4259

1437, 4535, 4536, 4547, 4546, 4249, 4250, 4261, 4260

1438, 4536, 4537, 4548, 4547, 4250, 4251, 4262, 4261

1439, 4537, 4538, 4549, 4548, 4251, 4252, 4263, 4262

1440, 4538, 1358, 1359, 4549, 4252, 1332, 1333, 4263

1441, 1229, 4539, 4550, 1230, 1203, 4253, 4264, 1204

1442, 4539, 4540, 4551, 4550, 4253, 4254, 4265, 4264

1443, 4540, 4541, 4552, 4551, 4254, 4255, 4266, 4265

1444, 4541, 4542, 4553, 4552, 4255, 4256, 4267, 4266

1445, 4542, 4543, 4554, 4553, 4256, 4257, 4268, 4267

1446, 4543, 4544, 4555, 4554, 4257, 4258, 4269, 4268

1447, 4544, 4545, 4556, 4555, 4258, 4259, 4270, 4269

1448, 4545, 4546, 4557, 4556, 4259, 4260, 4271, 4270

1449, 4546, 4547, 4558, 4557, 4260, 4261, 4272, 4271

1450, 4547, 4548, 4559, 4558, 4261, 4262, 4273, 4272

1451, 4548, 4549, 4560, 4559, 4262, 4263, 4274, 4273

1452, 4549, 1359, 1360, 4560, 4263, 1333, 1334, 4274

1453, 1230, 4550, 4561, 1231, 1204, 4264, 4275, 1205

1454, 4550, 4551, 4562, 4561, 4264, 4265, 4276, 4275

1455, 4551, 4552, 4563, 4562, 4265, 4266, 4277, 4276

1456, 4552, 4553, 4564, 4563, 4266, 4267, 4278, 4277

1457, 4553, 4554, 4565, 4564, 4267, 4268, 4279, 4278

1458, 4554, 4555, 4566, 4565, 4268, 4269, 4280, 4279

1459, 4555, 4556, 4567, 4566, 4269, 4270, 4281, 4280

1460, 4556, 4557, 4568, 4567, 4270, 4271, 4282, 4281

1461, 4557, 4558, 4569, 4568, 4271, 4272, 4283, 4282

1462, 4558, 4559, 4570, 4569, 4272, 4273, 4284, 4283

1463, 4559, 4560, 4571, 4570, 4273, 4274, 4285, 4284

1464, 4560, 1360, 1361, 4571, 4274, 1334, 1335, 4285

1465, 1231, 4561, 4572, 1232, 1205, 4275, 4286, 1206

1466, 4561, 4562, 4573, 4572, 4275, 4276, 4287, 4286

1467, 4562, 4563, 4574, 4573, 4276, 4277, 4288, 4287

1468, 4563, 4564, 4575, 4574, 4277, 4278, 4289, 4288

1469, 4564, 4565, 4576, 4575, 4278, 4279, 4290, 4289

1470, 4565, 4566, 4577, 4576, 4279, 4280, 4291, 4290

1471, 4566, 4567, 4578, 4577, 4280, 4281, 4292, 4291

1472, 4567, 4568, 4579, 4578, 4281, 4282, 4293, 4292

1473, 4568, 4569, 4580, 4579, 4282, 4283, 4294, 4293

1474, 4569, 4570, 4581, 4580, 4283, 4284, 4295, 4294

1475, 4570, 4571, 4582, 4581, 4284, 4285, 4296, 4295

1476, 4571, 1361, 1362, 4582, 4285, 1335, 1336, 4296

1477, 1232, 4572, 4583, 1233, 1206, 4286, 4297, 1207

1478, 4572, 4573, 4584, 4583, 4286, 4287, 4298, 4297

1479, 4573, 4574, 4585, 4584, 4287, 4288, 4299, 4298

1480, 4574, 4575, 4586, 4585, 4288, 4289, 4300, 4299

1481, 4575, 4576, 4587, 4586, 4289, 4290, 4301, 4300

1482, 4576, 4577, 4588, 4587, 4290, 4291, 4302, 4301

1483, 4577, 4578, 4589, 4588, 4291, 4292, 4303, 4302

1484, 4578, 4579, 4590, 4589, 4292, 4293, 4304, 4303

1485, 4579, 4580, 4591, 4590, 4293, 4294, 4305, 4304

1486, 4580, 4581, 4592, 4591, 4294, 4295, 4306, 4305

1487, 4581, 4582, 4593, 4592, 4295, 4296, 4307, 4306

1488, 4582, 1362, 1363, 4593, 4296, 1336, 1337, 4307

1489, 1233, 4583, 4594, 1234, 1207, 4297, 4308, 1208

1490, 4583, 4584, 4595, 4594, 4297, 4298, 4309, 4308

1491, 4584, 4585, 4596, 4595, 4298, 4299, 4310, 4309

1492, 4585, 4586, 4597, 4596, 4299, 4300, 4311, 4310

1493, 4586, 4587, 4598, 4597, 4300, 4301, 4312, 4311

1494, 4587, 4588, 4599, 4598, 4301, 4302, 4313, 4312

1495, 4588, 4589, 4600, 4599, 4302, 4303, 4314, 4313

1496, 4589, 4590, 4601, 4600, 4303, 4304, 4315, 4314

1497, 4590, 4591, 4602, 4601, 4304, 4305, 4316, 4315

1498, 4591, 4592, 4603, 4602, 4305, 4306, 4317, 4316

1499, 4592, 4593, 4604, 4603, 4306, 4307, 4318, 4317

1500, 4593, 1363, 1364, 4604, 4307, 1337, 1338, 4318

1501, 1234, 4594, 4605, 1235, 1208, 4308, 4319, 1209

1502, 4594, 4595, 4606, 4605, 4308, 4309, 4320, 4319

1503, 4595, 4596, 4607, 4606, 4309, 4310, 4321, 4320

1504, 4596, 4597, 4608, 4607, 4310, 4311, 4322, 4321

1505, 4597, 4598, 4609, 4608, 4311, 4312, 4323, 4322

1506, 4598, 4599, 4610, 4609, 4312, 4313, 4324, 4323

1507, 4599, 4600, 4611, 4610, 4313, 4314, 4325, 4324

1508, 4600, 4601, 4612, 4611, 4314, 4315, 4326, 4325

1509, 4601, 4602, 4613, 4612, 4315, 4316, 4327, 4326

1510, 4602, 4603, 4614, 4613, 4316, 4317, 4328, 4327

1511, 4603, 4604, 4615, 4614, 4317, 4318, 4329, 4328

1512, 4604, 1364, 1365, 4615, 4318, 1338, 1339, 4329

1513, 1235, 4605, 4616, 1236, 1209, 4319, 4330, 1210

1514, 4605, 4606, 4617, 4616, 4319, 4320, 4331, 4330

1515, 4606, 4607, 4618, 4617, 4320, 4321, 4332, 4331

1516, 4607, 4608, 4619, 4618, 4321, 4322, 4333, 4332

1517, 4608, 4609, 4620, 4619, 4322, 4323, 4334, 4333

1518, 4609, 4610, 4621, 4620, 4323, 4324, 4335, 4334

1519, 4610, 4611, 4622, 4621, 4324, 4325, 4336, 4335

1520, 4611, 4612, 4623, 4622, 4325, 4326, 4337, 4336

1521, 4612, 4613, 4624, 4623, 4326, 4327, 4338, 4337

1522, 4613, 4614, 4625, 4624, 4327, 4328, 4339, 4338

1523, 4614, 4615, 4626, 4625, 4328, 4329, 4340, 4339

1524, 4615, 1365, 1366, 4626, 4329, 1339, 1340, 4340

1525, 1236, 4616, 4627, 1237, 1210, 4330, 4341, 1211

1526, 4616, 4617, 4628, 4627, 4330, 4331, 4342, 4341

1527, 4617, 4618, 4629, 4628, 4331, 4332, 4343, 4342

1528, 4618, 4619, 4630, 4629, 4332, 4333, 4344, 4343

1529, 4619, 4620, 4631, 4630, 4333, 4334, 4345, 4344

1530, 4620, 4621, 4632, 4631, 4334, 4335, 4346, 4345

1531, 4621, 4622, 4633, 4632, 4335, 4336, 4347, 4346

1532, 4622, 4623, 4634, 4633, 4336, 4337, 4348, 4347

1533, 4623, 4624, 4635, 4634, 4337, 4338, 4349, 4348

1534, 4624, 4625, 4636, 4635, 4338, 4339, 4350, 4349

1535, 4625, 4626, 4637, 4636, 4339, 4340, 4351, 4350

1536, 4626, 1366, 1367, 4637, 4340, 1340, 1341, 4351

1537, 1237, 4627, 4638, 1238, 1211, 4341, 4352, 1212

1538, 4627, 4628, 4639, 4638, 4341, 4342, 4353, 4352

1539, 4628, 4629, 4640, 4639, 4342, 4343, 4354, 4353

1540, 4629, 4630, 4641, 4640, 4343, 4344, 4355, 4354

1541, 4630, 4631, 4642, 4641, 4344, 4345, 4356, 4355

1542, 4631, 4632, 4643, 4642, 4345, 4346, 4357, 4356

1543, 4632, 4633, 4644, 4643, 4346, 4347, 4358, 4357

1544, 4633, 4634, 4645, 4644, 4347, 4348, 4359, 4358

1545, 4634, 4635, 4646, 4645, 4348, 4349, 4360, 4359

1546, 4635, 4636, 4647, 4646, 4349, 4350, 4361, 4360

1547, 4636, 4637, 4648, 4647, 4350, 4351, 4362, 4361

1548, 4637, 1367, 1368, 4648, 4351, 1341, 1342, 4362

1549, 1238, 4638, 4649, 1239, 1212, 4352, 4363, 1213

1550, 4638, 4639, 4650, 4649, 4352, 4353, 4364, 4363

1551, 4639, 4640, 4651, 4650, 4353, 4354, 4365, 4364

1552, 4640, 4641, 4652, 4651, 4354, 4355, 4366, 4365

1553, 4641, 4642, 4653, 4652, 4355, 4356, 4367, 4366

1554, 4642, 4643, 4654, 4653, 4356, 4357, 4368, 4367

1555, 4643, 4644, 4655, 4654, 4357, 4358, 4369, 4368

1556, 4644, 4645, 4656, 4655, 4358, 4359, 4370, 4369

1557, 4645, 4646, 4657, 4656, 4359, 4360, 4371, 4370

1558, 4646, 4647, 4658, 4657, 4360, 4361, 4372, 4371

1559, 4647, 4648, 4659, 4658, 4361, 4362, 4373, 4372

1560, 4648, 1368, 1369, 4659, 4362, 1342, 1343, 4373

1561, 1239, 4649, 4660, 1240, 1213, 4363, 4374, 1214

1562, 4649, 4650, 4661, 4660, 4363, 4364, 4375, 4374

1563, 4650, 4651, 4662, 4661, 4364, 4365, 4376, 4375

1564, 4651, 4652, 4663, 4662, 4365, 4366, 4377, 4376

1565, 4652, 4653, 4664, 4663, 4366, 4367, 4378, 4377

1566, 4653, 4654, 4665, 4664, 4367, 4368, 4379, 4378

1567, 4654, 4655, 4666, 4665, 4368, 4369, 4380, 4379

1568, 4655, 4656, 4667, 4666, 4369, 4370, 4381, 4380

1569, 4656, 4657, 4668, 4667, 4370, 4371, 4382, 4381

1570, 4657, 4658, 4669, 4668, 4371, 4372, 4383, 4382

1571, 4658, 4659, 4670, 4669, 4372, 4373, 4384, 4383

1572, 4659, 1369, 1370, 4670, 4373, 1343, 1344, 4384

1573, 1240, 4660, 4671, 1241, 1214, 4374, 4385, 1215

1574, 4660, 4661, 4672, 4671, 4374, 4375, 4386, 4385

1575, 4661, 4662, 4673, 4672, 4375, 4376, 4387, 4386

1576, 4662, 4663, 4674, 4673, 4376, 4377, 4388, 4387

1577, 4663, 4664, 4675, 4674, 4377, 4378, 4389, 4388

1578, 4664, 4665, 4676, 4675, 4378, 4379, 4390, 4389

1579, 4665, 4666, 4677, 4676, 4379, 4380, 4391, 4390

1580, 4666, 4667, 4678, 4677, 4380, 4381, 4392, 4391

1581, 4667, 4668, 4679, 4678, 4381, 4382, 4393, 4392

1582, 4668, 4669, 4680, 4679, 4382, 4383, 4394, 4393

1583, 4669, 4670, 4681, 4680, 4383, 4384, 4395, 4394

1584, 4670, 1370, 1371, 4681, 4384, 1344, 1345, 4395

1585, 1241, 4671, 4682, 1242, 1215, 4385, 4396, 1216

1586, 4671, 4672, 4683, 4682, 4385, 4386, 4397, 4396

1587, 4672, 4673, 4684, 4683, 4386, 4387, 4398, 4397

1588, 4673, 4674, 4685, 4684, 4387, 4388, 4399, 4398

1589, 4674, 4675, 4686, 4685, 4388, 4389, 4400, 4399

1590, 4675, 4676, 4687, 4686, 4389, 4390, 4401, 4400

1591, 4676, 4677, 4688, 4687, 4390, 4391, 4402, 4401

1592, 4677, 4678, 4689, 4688, 4391, 4392, 4403, 4402

1593, 4678, 4679, 4690, 4689, 4392, 4393, 4404, 4403

1594, 4679, 4680, 4691, 4690, 4393, 4394, 4405, 4404

1595, 4680, 4681, 4692, 4691, 4394, 4395, 4406, 4405

1596, 4681, 1371, 1372, 4692, 4395, 1345, 1346, 4406

1597, 1242, 4682, 4693, 1243, 1216, 4396, 4407, 1217

1598, 4682, 4683, 4694, 4693, 4396, 4397, 4408, 4407

1599, 4683, 4684, 4695, 4694, 4397, 4398, 4409, 4408

1600, 4684, 4685, 4696, 4695, 4398, 4399, 4410, 4409

1601, 4685, 4686, 4697, 4696, 4399, 4400, 4411, 4410

1602, 4686, 4687, 4698, 4697, 4400, 4401, 4412, 4411

1603, 4687, 4688, 4699, 4698, 4401, 4402, 4413, 4412

1604, 4688, 4689, 4700, 4699, 4402, 4403, 4414, 4413

1605, 4689, 4690, 4701, 4700, 4403, 4404, 4415, 4414

1606, 4690, 4691, 4702, 4701, 4404, 4405, 4416, 4415

1607, 4691, 4692, 4703, 4702, 4405, 4406, 4417, 4416

1608, 4692, 1372, 1373, 4703, 4406, 1346, 1347, 4417

1609, 1243, 4693, 1473, 185, 1217, 4407, 1462, 184

1610, 4693, 4694, 1474, 1473, 4407, 4408, 1463, 1462

1611, 4694, 4695, 1475, 1474, 4408, 4409, 1464, 1463

1612, 4695, 4696, 1476, 1475, 4409, 4410, 1465, 1464

1613, 4696, 4697, 1477, 1476, 4410, 4411, 1466, 1465

1614, 4697, 4698, 1478, 1477, 4411, 4412, 1467, 1466

1615, 4698, 4699, 1479, 1478, 4412, 4413, 1468, 1467

1616, 4699, 4700, 1480, 1479, 4413, 4414, 1469, 1468

1617, 4700, 4701, 1481, 1480, 4414, 4415, 1470, 1469

1618, 4701, 4702, 1482, 1481, 4415, 4416, 1471, 1470

1619, 4702, 4703, 1483, 1482, 4416, 4417, 1472, 1471

1620, 4703, 1373, 191, 1483, 4417, 1347, 192, 1472

1621, 8, 117, 828, 155, 186, 1418, 4418, 1218

1622, 117, 116, 829, 828, 1418, 1419, 4419, 4418

1623, 116, 115, 830, 829, 1419, 1420, 4420, 4419

1624, 115, 114, 831, 830, 1420, 1421, 4421, 4420

1625, 114, 113, 832, 831, 1421, 1422, 4422, 4421

1626, 113, 112, 833, 832, 1422, 1423, 4423, 4422

1627, 112, 111, 834, 833, 1423, 1424, 4424, 4423

1628, 111, 110, 835, 834, 1424, 1425, 4425, 4424

1629, 110, 109, 836, 835, 1425, 1426, 4426, 4425

1630, 109, 108, 837, 836, 1426, 1427, 4427, 4426

1631, 108, 107, 838, 837, 1427, 1428, 4428, 4427

1632, 107, 5, 143, 838, 1428, 200, 1348, 4428

1633, 155, 828, 839, 156, 1218, 4418, 4429, 1219

1634, 828, 829, 840, 839, 4418, 4419, 4430, 4429

1635, 829, 830, 841, 840, 4419, 4420, 4431, 4430

1636, 830, 831, 842, 841, 4420, 4421, 4432, 4431

1637, 831, 832, 843, 842, 4421, 4422, 4433, 4432

1638, 832, 833, 844, 843, 4422, 4423, 4434, 4433

1639, 833, 834, 845, 844, 4423, 4424, 4435, 4434

1640, 834, 835, 846, 845, 4424, 4425, 4436, 4435

1641, 835, 836, 847, 846, 4425, 4426, 4437, 4436

1642, 836, 837, 848, 847, 4426, 4427, 4438, 4437

1643, 837, 838, 849, 848, 4427, 4428, 4439, 4438

1644, 838, 143, 142, 849, 4428, 1348, 1349, 4439

1645, 156, 839, 850, 157, 1219, 4429, 4440, 1220

1646, 839, 840, 851, 850, 4429, 4430, 4441, 4440

1647, 840, 841, 852, 851, 4430, 4431, 4442, 4441

1648, 841, 842, 853, 852, 4431, 4432, 4443, 4442

1649, 842, 843, 854, 853, 4432, 4433, 4444, 4443

1650, 843, 844, 855, 854, 4433, 4434, 4445, 4444

1651, 844, 845, 856, 855, 4434, 4435, 4446, 4445

1652, 845, 846, 857, 856, 4435, 4436, 4447, 4446

1653, 846, 847, 858, 857, 4436, 4437, 4448, 4447

1654, 847, 848, 859, 858, 4437, 4438, 4449, 4448

1655, 848, 849, 860, 859, 4438, 4439, 4450, 4449

1656, 849, 142, 141, 860, 4439, 1349, 1350, 4450

1657, 157, 850, 861, 158, 1220, 4440, 4451, 1221

1658, 850, 851, 862, 861, 4440, 4441, 4452, 4451

1659, 851, 852, 863, 862, 4441, 4442, 4453, 4452

1660, 852, 853, 864, 863, 4442, 4443, 4454, 4453

1661, 853, 854, 865, 864, 4443, 4444, 4455, 4454

1662, 854, 855, 866, 865, 4444, 4445, 4456, 4455

1663, 855, 856, 867, 866, 4445, 4446, 4457, 4456

1664, 856, 857, 868, 867, 4446, 4447, 4458, 4457

1665, 857, 858, 869, 868, 4447, 4448, 4459, 4458

1666, 858, 859, 870, 869, 4448, 4449, 4460, 4459

1667, 859, 860, 871, 870, 4449, 4450, 4461, 4460

1668, 860, 141, 140, 871, 4450, 1350, 1351, 4461

1669, 158, 861, 872, 159, 1221, 4451, 4462, 1222

1670, 861, 862, 873, 872, 4451, 4452, 4463, 4462

1671, 862, 863, 874, 873, 4452, 4453, 4464, 4463

1672, 863, 864, 875, 874, 4453, 4454, 4465, 4464

1673, 864, 865, 876, 875, 4454, 4455, 4466, 4465

1674, 865, 866, 877, 876, 4455, 4456, 4467, 4466

1675, 866, 867, 878, 877, 4456, 4457, 4468, 4467

1676, 867, 868, 879, 878, 4457, 4458, 4469, 4468

1677, 868, 869, 880, 879, 4458, 4459, 4470, 4469

1678, 869, 870, 881, 880, 4459, 4460, 4471, 4470

1679, 870, 871, 882, 881, 4460, 4461, 4472, 4471

1680, 871, 140, 139, 882, 4461, 1351, 1352, 4472

1681, 159, 872, 883, 160, 1222, 4462, 4473, 1223

1682, 872, 873, 884, 883, 4462, 4463, 4474, 4473

1683, 873, 874, 885, 884, 4463, 4464, 4475, 4474

1684, 874, 875, 886, 885, 4464, 4465, 4476, 4475

1685, 875, 876, 887, 886, 4465, 4466, 4477, 4476

1686, 876, 877, 888, 887, 4466, 4467, 4478, 4477

1687, 877, 878, 889, 888, 4467, 4468, 4479, 4478

1688, 878, 879, 890, 889, 4468, 4469, 4480, 4479

1689, 879, 880, 891, 890, 4469, 4470, 4481, 4480

1690, 880, 881, 892, 891, 4470, 4471, 4482, 4481

1691, 881, 882, 893, 892, 4471, 4472, 4483, 4482

1692, 882, 139, 138, 893, 4472, 1352, 1353, 4483

1693, 160, 883, 894, 161, 1223, 4473, 4484, 1224

1694, 883, 884, 895, 894, 4473, 4474, 4485, 4484

1695, 884, 885, 896, 895, 4474, 4475, 4486, 4485

1696, 885, 886, 897, 896, 4475, 4476, 4487, 4486

1697, 886, 887, 898, 897, 4476, 4477, 4488, 4487

1698, 887, 888, 899, 898, 4477, 4478, 4489, 4488

1699, 888, 889, 900, 899, 4478, 4479, 4490, 4489

1700, 889, 890, 901, 900, 4479, 4480, 4491, 4490

1701, 890, 891, 902, 901, 4480, 4481, 4492, 4491

1702, 891, 892, 903, 902, 4481, 4482, 4493, 4492

1703, 892, 893, 904, 903, 4482, 4483, 4494, 4493

1704, 893, 138, 137, 904, 4483, 1353, 1354, 4494

1705, 161, 894, 905, 162, 1224, 4484, 4495, 1225

1706, 894, 895, 906, 905, 4484, 4485, 4496, 4495

1707, 895, 896, 907, 906, 4485, 4486, 4497, 4496

1708, 896, 897, 908, 907, 4486, 4487, 4498, 4497

1709, 897, 898, 909, 908, 4487, 4488, 4499, 4498

1710, 898, 899, 910, 909, 4488, 4489, 4500, 4499

1711, 899, 900, 911, 910, 4489, 4490, 4501, 4500

1712, 900, 901, 912, 911, 4490, 4491, 4502, 4501

1713, 901, 902, 913, 912, 4491, 4492, 4503, 4502

1714, 902, 903, 914, 913, 4492, 4493, 4504, 4503

1715, 903, 904, 915, 914, 4493, 4494, 4505, 4504

1716, 904, 137, 136, 915, 4494, 1354, 1355, 4505

1717, 162, 905, 916, 163, 1225, 4495, 4506, 1226

1718, 905, 906, 917, 916, 4495, 4496, 4507, 4506

1719, 906, 907, 918, 917, 4496, 4497, 4508, 4507

1720, 907, 908, 919, 918, 4497, 4498, 4509, 4508

1721, 908, 909, 920, 919, 4498, 4499, 4510, 4509

1722, 909, 910, 921, 920, 4499, 4500, 4511, 4510

1723, 910, 911, 922, 921, 4500, 4501, 4512, 4511

1724, 911, 912, 923, 922, 4501, 4502, 4513, 4512

1725, 912, 913, 924, 923, 4502, 4503, 4514, 4513

1726, 913, 914, 925, 924, 4503, 4504, 4515, 4514

1727, 914, 915, 926, 925, 4504, 4505, 4516, 4515

1728, 915, 136, 135, 926, 4505, 1355, 1356, 4516

1729, 163, 916, 927, 164, 1226, 4506, 4517, 1227

1730, 916, 917, 928, 927, 4506, 4507, 4518, 4517

1731, 917, 918, 929, 928, 4507, 4508, 4519, 4518

1732, 918, 919, 930, 929, 4508, 4509, 4520, 4519

1733, 919, 920, 931, 930, 4509, 4510, 4521, 4520

1734, 920, 921, 932, 931, 4510, 4511, 4522, 4521

1735, 921, 922, 933, 932, 4511, 4512, 4523, 4522

1736, 922, 923, 934, 933, 4512, 4513, 4524, 4523

1737, 923, 924, 935, 934, 4513, 4514, 4525, 4524

1738, 924, 925, 936, 935, 4514, 4515, 4526, 4525

1739, 925, 926, 937, 936, 4515, 4516, 4527, 4526

1740, 926, 135, 134, 937, 4516, 1356, 1357, 4527

1741, 164, 927, 938, 165, 1227, 4517, 4528, 1228

1742, 927, 928, 939, 938, 4517, 4518, 4529, 4528

1743, 928, 929, 940, 939, 4518, 4519, 4530, 4529

1744, 929, 930, 941, 940, 4519, 4520, 4531, 4530

1745, 930, 931, 942, 941, 4520, 4521, 4532, 4531

1746, 931, 932, 943, 942, 4521, 4522, 4533, 4532

1747, 932, 933, 944, 943, 4522, 4523, 4534, 4533

1748, 933, 934, 945, 944, 4523, 4524, 4535, 4534

1749, 934, 935, 946, 945, 4524, 4525, 4536, 4535

1750, 935, 936, 947, 946, 4525, 4526, 4537, 4536

1751, 936, 937, 948, 947, 4526, 4527, 4538, 4537

1752, 937, 134, 133, 948, 4527, 1357, 1358, 4538

1753, 165, 938, 949, 166, 1228, 4528, 4539, 1229

1754, 938, 939, 950, 949, 4528, 4529, 4540, 4539

1755, 939, 940, 951, 950, 4529, 4530, 4541, 4540

1756, 940, 941, 952, 951, 4530, 4531, 4542, 4541

1757, 941, 942, 953, 952, 4531, 4532, 4543, 4542

1758, 942, 943, 954, 953, 4532, 4533, 4544, 4543

1759, 943, 944, 955, 954, 4533, 4534, 4545, 4544

1760, 944, 945, 956, 955, 4534, 4535, 4546, 4545

1761, 945, 946, 957, 956, 4535, 4536, 4547, 4546

1762, 946, 947, 958, 957, 4536, 4537, 4548, 4547

1763, 947, 948, 959, 958, 4537, 4538, 4549, 4548

1764, 948, 133, 132, 959, 4538, 1358, 1359, 4549

1765, 166, 949, 960, 167, 1229, 4539, 4550, 1230

1766, 949, 950, 961, 960, 4539, 4540, 4551, 4550

1767, 950, 951, 962, 961, 4540, 4541, 4552, 4551

1768, 951, 952, 963, 962, 4541, 4542, 4553, 4552

1769, 952, 953, 964, 963, 4542, 4543, 4554, 4553

1770, 953, 954, 965, 964, 4543, 4544, 4555, 4554

1771, 954, 955, 966, 965, 4544, 4545, 4556, 4555

1772, 955, 956, 967, 966, 4545, 4546, 4557, 4556

1773, 956, 957, 968, 967, 4546, 4547, 4558, 4557

1774, 957, 958, 969, 968, 4547, 4548, 4559, 4558

1775, 958, 959, 970, 969, 4548, 4549, 4560, 4559

1776, 959, 132, 131, 970, 4549, 1359, 1360, 4560

1777, 167, 960, 971, 168, 1230, 4550, 4561, 1231

1778, 960, 961, 972, 971, 4550, 4551, 4562, 4561

1779, 961, 962, 973, 972, 4551, 4552, 4563, 4562

1780, 962, 963, 974, 973, 4552, 4553, 4564, 4563

1781, 963, 964, 975, 974, 4553, 4554, 4565, 4564

1782, 964, 965, 976, 975, 4554, 4555, 4566, 4565

1783, 965, 966, 977, 976, 4555, 4556, 4567, 4566

1784, 966, 967, 978, 977, 4556, 4557, 4568, 4567

1785, 967, 968, 979, 978, 4557, 4558, 4569, 4568

1786, 968, 969, 980, 979, 4558, 4559, 4570, 4569

1787, 969, 970, 981, 980, 4559, 4560, 4571, 4570

1788, 970, 131, 130, 981, 4560, 1360, 1361, 4571

1789, 168, 971, 982, 169, 1231, 4561, 4572, 1232

1790, 971, 972, 983, 982, 4561, 4562, 4573, 4572

1791, 972, 973, 984, 983, 4562, 4563, 4574, 4573

1792, 973, 974, 985, 984, 4563, 4564, 4575, 4574

1793, 974, 975, 986, 985, 4564, 4565, 4576, 4575

1794, 975, 976, 987, 986, 4565, 4566, 4577, 4576

1795, 976, 977, 988, 987, 4566, 4567, 4578, 4577

1796, 977, 978, 989, 988, 4567, 4568, 4579, 4578

1797, 978, 979, 990, 989, 4568, 4569, 4580, 4579

1798, 979, 980, 991, 990, 4569, 4570, 4581, 4580

1799, 980, 981, 992, 991, 4570, 4571, 4582, 4581

1800, 981, 130, 129, 992, 4571, 1361, 1362, 4582

1801, 169, 982, 993, 170, 1232, 4572, 4583, 1233

1802, 982, 983, 994, 993, 4572, 4573, 4584, 4583

1803, 983, 984, 995, 994, 4573, 4574, 4585, 4584

1804, 984, 985, 996, 995, 4574, 4575, 4586, 4585

1805, 985, 986, 997, 996, 4575, 4576, 4587, 4586

1806, 986, 987, 998, 997, 4576, 4577, 4588, 4587

1807, 987, 988, 999, 998, 4577, 4578, 4589, 4588

1808, 988, 989, 1000, 999, 4578, 4579, 4590, 4589

1809, 989, 990, 1001, 1000, 4579, 4580, 4591, 4590

1810, 990, 991, 1002, 1001, 4580, 4581, 4592, 4591

1811, 991, 992, 1003, 1002, 4581, 4582, 4593, 4592

1812, 992, 129, 128, 1003, 4582, 1362, 1363, 4593

1813, 170, 993, 1004, 171, 1233, 4583, 4594, 1234

1814, 993, 994, 1005, 1004, 4583, 4584, 4595, 4594

1815, 994, 995, 1006, 1005, 4584, 4585, 4596, 4595

1816, 995, 996, 1007, 1006, 4585, 4586, 4597, 4596

1817, 996, 997, 1008, 1007, 4586, 4587, 4598, 4597

1818, 997, 998, 1009, 1008, 4587, 4588, 4599, 4598

1819, 998, 999, 1010, 1009, 4588, 4589, 4600, 4599

1820, 999, 1000, 1011, 1010, 4589, 4590, 4601, 4600

1821, 1000, 1001, 1012, 1011, 4590, 4591, 4602, 4601

1822, 1001, 1002, 1013, 1012, 4591, 4592, 4603, 4602

1823, 1002, 1003, 1014, 1013, 4592, 4593, 4604, 4603

1824, 1003, 128, 127, 1014, 4593, 1363, 1364, 4604

1825, 171, 1004, 1015, 172, 1234, 4594, 4605, 1235

1826, 1004, 1005, 1016, 1015, 4594, 4595, 4606, 4605

1827, 1005, 1006, 1017, 1016, 4595, 4596, 4607, 4606

1828, 1006, 1007, 1018, 1017, 4596, 4597, 4608, 4607

1829, 1007, 1008, 1019, 1018, 4597, 4598, 4609, 4608

1830, 1008, 1009, 1020, 1019, 4598, 4599, 4610, 4609

1831, 1009, 1010, 1021, 1020, 4599, 4600, 4611, 4610

1832, 1010, 1011, 1022, 1021, 4600, 4601, 4612, 4611

1833, 1011, 1012, 1023, 1022, 4601, 4602, 4613, 4612

1834, 1012, 1013, 1024, 1023, 4602, 4603, 4614, 4613

1835, 1013, 1014, 1025, 1024, 4603, 4604, 4615, 4614

1836, 1014, 127, 126, 1025, 4604, 1364, 1365, 4615

1837, 172, 1015, 1026, 173, 1235, 4605, 4616, 1236

1838, 1015, 1016, 1027, 1026, 4605, 4606, 4617, 4616

1839, 1016, 1017, 1028, 1027, 4606, 4607, 4618, 4617

1840, 1017, 1018, 1029, 1028, 4607, 4608, 4619, 4618

1841, 1018, 1019, 1030, 1029, 4608, 4609, 4620, 4619

1842, 1019, 1020, 1031, 1030, 4609, 4610, 4621, 4620

1843, 1020, 1021, 1032, 1031, 4610, 4611, 4622, 4621

1844, 1021, 1022, 1033, 1032, 4611, 4612, 4623, 4622

1845, 1022, 1023, 1034, 1033, 4612, 4613, 4624, 4623

1846, 1023, 1024, 1035, 1034, 4613, 4614, 4625, 4624

1847, 1024, 1025, 1036, 1035, 4614, 4615, 4626, 4625

1848, 1025, 126, 125, 1036, 4615, 1365, 1366, 4626

1849, 173, 1026, 1037, 174, 1236, 4616, 4627, 1237

1850, 1026, 1027, 1038, 1037, 4616, 4617, 4628, 4627

1851, 1027, 1028, 1039, 1038, 4617, 4618, 4629, 4628

1852, 1028, 1029, 1040, 1039, 4618, 4619, 4630, 4629

1853, 1029, 1030, 1041, 1040, 4619, 4620, 4631, 4630

1854, 1030, 1031, 1042, 1041, 4620, 4621, 4632, 4631

1855, 1031, 1032, 1043, 1042, 4621, 4622, 4633, 4632

1856, 1032, 1033, 1044, 1043, 4622, 4623, 4634, 4633

1857, 1033, 1034, 1045, 1044, 4623, 4624, 4635, 4634

1858, 1034, 1035, 1046, 1045, 4624, 4625, 4636, 4635

1859, 1035, 1036, 1047, 1046, 4625, 4626, 4637, 4636

1860, 1036, 125, 124, 1047, 4626, 1366, 1367, 4637

1861, 174, 1037, 1048, 175, 1237, 4627, 4638, 1238

1862, 1037, 1038, 1049, 1048, 4627, 4628, 4639, 4638

1863, 1038, 1039, 1050, 1049, 4628, 4629, 4640, 4639

1864, 1039, 1040, 1051, 1050, 4629, 4630, 4641, 4640

1865, 1040, 1041, 1052, 1051, 4630, 4631, 4642, 4641

1866, 1041, 1042, 1053, 1052, 4631, 4632, 4643, 4642

1867, 1042, 1043, 1054, 1053, 4632, 4633, 4644, 4643

1868, 1043, 1044, 1055, 1054, 4633, 4634, 4645, 4644

1869, 1044, 1045, 1056, 1055, 4634, 4635, 4646, 4645

1870, 1045, 1046, 1057, 1056, 4635, 4636, 4647, 4646

1871, 1046, 1047, 1058, 1057, 4636, 4637, 4648, 4647

1872, 1047, 124, 123, 1058, 4637, 1367, 1368, 4648

1873, 175, 1048, 1059, 176, 1238, 4638, 4649, 1239

1874, 1048, 1049, 1060, 1059, 4638, 4639, 4650, 4649

1875, 1049, 1050, 1061, 1060, 4639, 4640, 4651, 4650

1876, 1050, 1051, 1062, 1061, 4640, 4641, 4652, 4651

1877, 1051, 1052, 1063, 1062, 4641, 4642, 4653, 4652

1878, 1052, 1053, 1064, 1063, 4642, 4643, 4654, 4653

1879, 1053, 1054, 1065, 1064, 4643, 4644, 4655, 4654

1880, 1054, 1055, 1066, 1065, 4644, 4645, 4656, 4655

1881, 1055, 1056, 1067, 1066, 4645, 4646, 4657, 4656

1882, 1056, 1057, 1068, 1067, 4646, 4647, 4658, 4657

1883, 1057, 1058, 1069, 1068, 4647, 4648, 4659, 4658

1884, 1058, 123, 122, 1069, 4648, 1368, 1369, 4659

1885, 176, 1059, 1070, 177, 1239, 4649, 4660, 1240

1886, 1059, 1060, 1071, 1070, 4649, 4650, 4661, 4660

1887, 1060, 1061, 1072, 1071, 4650, 4651, 4662, 4661

1888, 1061, 1062, 1073, 1072, 4651, 4652, 4663, 4662

1889, 1062, 1063, 1074, 1073, 4652, 4653, 4664, 4663

1890, 1063, 1064, 1075, 1074, 4653, 4654, 4665, 4664

1891, 1064, 1065, 1076, 1075, 4654, 4655, 4666, 4665

1892, 1065, 1066, 1077, 1076, 4655, 4656, 4667, 4666

1893, 1066, 1067, 1078, 1077, 4656, 4657, 4668, 4667

1894, 1067, 1068, 1079, 1078, 4657, 4658, 4669, 4668

1895, 1068, 1069, 1080, 1079, 4658, 4659, 4670, 4669

1896, 1069, 122, 121, 1080, 4659, 1369, 1370, 4670

1897, 177, 1070, 1081, 178, 1240, 4660, 4671, 1241

1898, 1070, 1071, 1082, 1081, 4660, 4661, 4672, 4671

1899, 1071, 1072, 1083, 1082, 4661, 4662, 4673, 4672

1900, 1072, 1073, 1084, 1083, 4662, 4663, 4674, 4673

1901, 1073, 1074, 1085, 1084, 4663, 4664, 4675, 4674

1902, 1074, 1075, 1086, 1085, 4664, 4665, 4676, 4675

1903, 1075, 1076, 1087, 1086, 4665, 4666, 4677, 4676

1904, 1076, 1077, 1088, 1087, 4666, 4667, 4678, 4677

1905, 1077, 1078, 1089, 1088, 4667, 4668, 4679, 4678

1906, 1078, 1079, 1090, 1089, 4668, 4669, 4680, 4679

1907, 1079, 1080, 1091, 1090, 4669, 4670, 4681, 4680

1908, 1080, 121, 120, 1091, 4670, 1370, 1371, 4681

1909, 178, 1081, 1092, 179, 1241, 4671, 4682, 1242

1910, 1081, 1082, 1093, 1092, 4671, 4672, 4683, 4682

1911, 1082, 1083, 1094, 1093, 4672, 4673, 4684, 4683

1912, 1083, 1084, 1095, 1094, 4673, 4674, 4685, 4684

1913, 1084, 1085, 1096, 1095, 4674, 4675, 4686, 4685

1914, 1085, 1086, 1097, 1096, 4675, 4676, 4687, 4686

1915, 1086, 1087, 1098, 1097, 4676, 4677, 4688, 4687

1916, 1087, 1088, 1099, 1098, 4677, 4678, 4689, 4688

1917, 1088, 1089, 1100, 1099, 4678, 4679, 4690, 4689

1918, 1089, 1090, 1101, 1100, 4679, 4680, 4691, 4690

1919, 1090, 1091, 1102, 1101, 4680, 4681, 4692, 4691

1920, 1091, 120, 119, 1102, 4681, 1371, 1372, 4692

1921, 179, 1092, 1103, 180, 1242, 4682, 4693, 1243

1922, 1092, 1093, 1104, 1103, 4682, 4683, 4694, 4693

1923, 1093, 1094, 1105, 1104, 4683, 4684, 4695, 4694

1924, 1094, 1095, 1106, 1105, 4684, 4685, 4696, 4695

1925, 1095, 1096, 1107, 1106, 4685, 4686, 4697, 4696

1926, 1096, 1097, 1108, 1107, 4686, 4687, 4698, 4697

1927, 1097, 1098, 1109, 1108, 4687, 4688, 4699, 4698

1928, 1098, 1099, 1110, 1109, 4688, 4689, 4700, 4699

1929, 1099, 1100, 1111, 1110, 4689, 4690, 4701, 4700

1930, 1100, 1101, 1112, 1111, 4690, 4691, 4702, 4701

1931, 1101, 1102, 1113, 1112, 4691, 4692, 4703, 4702

1932, 1102, 119, 118, 1113, 4692, 1372, 1373, 4703

1933, 180, 1103, 144, 7, 1243, 4693, 1473, 185

1934, 1103, 1104, 145, 144, 4693, 4694, 1474, 1473

1935, 1104, 1105, 146, 145, 4694, 4695, 1475, 1474

1936, 1105, 1106, 147, 146, 4695, 4696, 1476, 1475

1937, 1106, 1107, 148, 147, 4696, 4697, 1477, 1476

1938, 1107, 1108, 149, 148, 4697, 4698, 1478, 1477

1939, 1108, 1109, 150, 149, 4698, 4699, 1479, 1478

1940, 1109, 1110, 151, 150, 4699, 4700, 1480, 1479

1941, 1110, 1111, 152, 151, 4700, 4701, 1481, 1480

1942, 1111, 1112, 153, 152, 4701, 4702, 1482, 1481

1943, 1112, 1113, 154, 153, 4702, 4703, 1483, 1482

1944, 1113, 118, 6, 154, 4703, 1373, 191, 1483

1945, 405, 2700, 4704, 2320, 9, 201, 1484, 297

1946, 2700, 2701, 4705, 4704, 201, 202, 1485, 1484

1947, 2701, 2702, 4706, 4705, 202, 203, 1486, 1485

1948, 2702, 2703, 4707, 4706, 203, 204, 1487, 1486

1949, 2703, 2704, 4708, 4707, 204, 205, 1488, 1487

1950, 2704, 2705, 4709, 4708, 205, 206, 1489, 1488

1951, 2705, 2706, 4710, 4709, 206, 207, 1490, 1489

1952, 2706, 2707, 4711, 4710, 207, 208, 1491, 1490

1953, 2707, 2708, 4712, 4711, 208, 209, 1492, 1491

1954, 2708, 2709, 4713, 4712, 209, 210, 1493, 1492

1955, 2709, 2710, 4714, 4713, 210, 211, 1494, 1493

1956, 2710, 411, 2510, 4714, 211, 10, 212, 1494

1957, 2320, 4704, 4715, 2321, 297, 1484, 1495, 296

1958, 4704, 4705, 4716, 4715, 1484, 1485, 1496, 1495

1959, 4705, 4706, 4717, 4716, 1485, 1486, 1497, 1496

1960, 4706, 4707, 4718, 4717, 1486, 1487, 1498, 1497

1961, 4707, 4708, 4719, 4718, 1487, 1488, 1499, 1498

1962, 4708, 4709, 4720, 4719, 1488, 1489, 1500, 1499

1963, 4709, 4710, 4721, 4720, 1489, 1490, 1501, 1500

1964, 4710, 4711, 4722, 4721, 1490, 1491, 1502, 1501

1965, 4711, 4712, 4723, 4722, 1491, 1492, 1503, 1502

1966, 4712, 4713, 4724, 4723, 1492, 1493, 1504, 1503

1967, 4713, 4714, 4725, 4724, 1493, 1494, 1505, 1504

1968, 4714, 2510, 2511, 4725, 1494, 212, 213, 1505

1969, 2321, 4715, 4726, 2322, 296, 1495, 1506, 295

1970, 4715, 4716, 4727, 4726, 1495, 1496, 1507, 1506

1971, 4716, 4717, 4728, 4727, 1496, 1497, 1508, 1507

1972, 4717, 4718, 4729, 4728, 1497, 1498, 1509, 1508

1973, 4718, 4719, 4730, 4729, 1498, 1499, 1510, 1509

1974, 4719, 4720, 4731, 4730, 1499, 1500, 1511, 1510

1975, 4720, 4721, 4732, 4731, 1500, 1501, 1512, 1511

1976, 4721, 4722, 4733, 4732, 1501, 1502, 1513, 1512

1977, 4722, 4723, 4734, 4733, 1502, 1503, 1514, 1513

1978, 4723, 4724, 4735, 4734, 1503, 1504, 1515, 1514

1979, 4724, 4725, 4736, 4735, 1504, 1505, 1516, 1515

1980, 4725, 2511, 2512, 4736, 1505, 213, 214, 1516

1981, 2322, 4726, 4737, 2323, 295, 1506, 1517, 294

1982, 4726, 4727, 4738, 4737, 1506, 1507, 1518, 1517

1983, 4727, 4728, 4739, 4738, 1507, 1508, 1519, 1518

1984, 4728, 4729, 4740, 4739, 1508, 1509, 1520, 1519

1985, 4729, 4730, 4741, 4740, 1509, 1510, 1521, 1520

1986, 4730, 4731, 4742, 4741, 1510, 1511, 1522, 1521

1987, 4731, 4732, 4743, 4742, 1511, 1512, 1523, 1522

1988, 4732, 4733, 4744, 4743, 1512, 1513, 1524, 1523

1989, 4733, 4734, 4745, 4744, 1513, 1514, 1525, 1524

1990, 4734, 4735, 4746, 4745, 1514, 1515, 1526, 1525

1991, 4735, 4736, 4747, 4746, 1515, 1516, 1527, 1526

1992, 4736, 2512, 2513, 4747, 1516, 214, 215, 1527

1993, 2323, 4737, 4748, 2324, 294, 1517, 1528, 293

1994, 4737, 4738, 4749, 4748, 1517, 1518, 1529, 1528

1995, 4738, 4739, 4750, 4749, 1518, 1519, 1530, 1529

1996, 4739, 4740, 4751, 4750, 1519, 1520, 1531, 1530

1997, 4740, 4741, 4752, 4751, 1520, 1521, 1532, 1531

1998, 4741, 4742, 4753, 4752, 1521, 1522, 1533, 1532

1999, 4742, 4743, 4754, 4753, 1522, 1523, 1534, 1533

2000, 4743, 4744, 4755, 4754, 1523, 1524, 1535, 1534

2001, 4744, 4745, 4756, 4755, 1524, 1525, 1536, 1535

2002, 4745, 4746, 4757, 4756, 1525, 1526, 1537, 1536

2003, 4746, 4747, 4758, 4757, 1526, 1527, 1538, 1537

2004, 4747, 2513, 2514, 4758, 1527, 215, 216, 1538

2005, 2324, 4748, 4759, 2325, 293, 1528, 1539, 292

2006, 4748, 4749, 4760, 4759, 1528, 1529, 1540, 1539

2007, 4749, 4750, 4761, 4760, 1529, 1530, 1541, 1540

2008, 4750, 4751, 4762, 4761, 1530, 1531, 1542, 1541

2009, 4751, 4752, 4763, 4762, 1531, 1532, 1543, 1542

2010, 4752, 4753, 4764, 4763, 1532, 1533, 1544, 1543

2011, 4753, 4754, 4765, 4764, 1533, 1534, 1545, 1544

2012, 4754, 4755, 4766, 4765, 1534, 1535, 1546, 1545

2013, 4755, 4756, 4767, 4766, 1535, 1536, 1547, 1546

2014, 4756, 4757, 4768, 4767, 1536, 1537, 1548, 1547

2015, 4757, 4758, 4769, 4768, 1537, 1538, 1549, 1548

2016, 4758, 2514, 2515, 4769, 1538, 216, 217, 1549

2017, 2325, 4759, 4770, 2326, 292, 1539, 1550, 291

2018, 4759, 4760, 4771, 4770, 1539, 1540, 1551, 1550

2019, 4760, 4761, 4772, 4771, 1540, 1541, 1552, 1551

2020, 4761, 4762, 4773, 4772, 1541, 1542, 1553, 1552

2021, 4762, 4763, 4774, 4773, 1542, 1543, 1554, 1553

2022, 4763, 4764, 4775, 4774, 1543, 1544, 1555, 1554

2023, 4764, 4765, 4776, 4775, 1544, 1545, 1556, 1555

2024, 4765, 4766, 4777, 4776, 1545, 1546, 1557, 1556

2025, 4766, 4767, 4778, 4777, 1546, 1547, 1558, 1557

2026, 4767, 4768, 4779, 4778, 1547, 1548, 1559, 1558

2027, 4768, 4769, 4780, 4779, 1548, 1549, 1560, 1559

2028, 4769, 2515, 2516, 4780, 1549, 217, 218, 1560

2029, 2326, 4770, 4781, 2327, 291, 1550, 1561, 290

2030, 4770, 4771, 4782, 4781, 1550, 1551, 1562, 1561

2031, 4771, 4772, 4783, 4782, 1551, 1552, 1563, 1562

2032, 4772, 4773, 4784, 4783, 1552, 1553, 1564, 1563

2033, 4773, 4774, 4785, 4784, 1553, 1554, 1565, 1564

2034, 4774, 4775, 4786, 4785, 1554, 1555, 1566, 1565

2035, 4775, 4776, 4787, 4786, 1555, 1556, 1567, 1566

2036, 4776, 4777, 4788, 4787, 1556, 1557, 1568, 1567

2037, 4777, 4778, 4789, 4788, 1557, 1558, 1569, 1568

2038, 4778, 4779, 4790, 4789, 1558, 1559, 1570, 1569

2039, 4779, 4780, 4791, 4790, 1559, 1560, 1571, 1570

2040, 4780, 2516, 2517, 4791, 1560, 218, 219, 1571

2041, 2327, 4781, 4792, 2328, 290, 1561, 1572, 289

2042, 4781, 4782, 4793, 4792, 1561, 1562, 1573, 1572

2043, 4782, 4783, 4794, 4793, 1562, 1563, 1574, 1573

2044, 4783, 4784, 4795, 4794, 1563, 1564, 1575, 1574

2045, 4784, 4785, 4796, 4795, 1564, 1565, 1576, 1575

2046, 4785, 4786, 4797, 4796, 1565, 1566, 1577, 1576

2047, 4786, 4787, 4798, 4797, 1566, 1567, 1578, 1577

2048, 4787, 4788, 4799, 4798, 1567, 1568, 1579, 1578

2049, 4788, 4789, 4800, 4799, 1568, 1569, 1580, 1579

2050, 4789, 4790, 4801, 4800, 1569, 1570, 1581, 1580

2051, 4790, 4791, 4802, 4801, 1570, 1571, 1582, 1581

2052, 4791, 2517, 2518, 4802, 1571, 219, 220, 1582

2053, 2328, 4792, 4803, 2329, 289, 1572, 1583, 288

2054, 4792, 4793, 4804, 4803, 1572, 1573, 1584, 1583

2055, 4793, 4794, 4805, 4804, 1573, 1574, 1585, 1584

2056, 4794, 4795, 4806, 4805, 1574, 1575, 1586, 1585

2057, 4795, 4796, 4807, 4806, 1575, 1576, 1587, 1586

2058, 4796, 4797, 4808, 4807, 1576, 1577, 1588, 1587

2059, 4797, 4798, 4809, 4808, 1577, 1578, 1589, 1588

2060, 4798, 4799, 4810, 4809, 1578, 1579, 1590, 1589

2061, 4799, 4800, 4811, 4810, 1579, 1580, 1591, 1590

2062, 4800, 4801, 4812, 4811, 1580, 1581, 1592, 1591

2063, 4801, 4802, 4813, 4812, 1581, 1582, 1593, 1592

2064, 4802, 2518, 2519, 4813, 1582, 220, 221, 1593

2065, 2329, 4803, 4814, 2330, 288, 1583, 1594, 287

2066, 4803, 4804, 4815, 4814, 1583, 1584, 1595, 1594

2067, 4804, 4805, 4816, 4815, 1584, 1585, 1596, 1595

2068, 4805, 4806, 4817, 4816, 1585, 1586, 1597, 1596

2069, 4806, 4807, 4818, 4817, 1586, 1587, 1598, 1597

2070, 4807, 4808, 4819, 4818, 1587, 1588, 1599, 1598

2071, 4808, 4809, 4820, 4819, 1588, 1589, 1600, 1599

2072, 4809, 4810, 4821, 4820, 1589, 1590, 1601, 1600

2073, 4810, 4811, 4822, 4821, 1590, 1591, 1602, 1601

2074, 4811, 4812, 4823, 4822, 1591, 1592, 1603, 1602

2075, 4812, 4813, 4824, 4823, 1592, 1593, 1604, 1603

2076, 4813, 2519, 2520, 4824, 1593, 221, 222, 1604

2077, 2330, 4814, 4825, 2331, 287, 1594, 1605, 286

2078, 4814, 4815, 4826, 4825, 1594, 1595, 1606, 1605

2079, 4815, 4816, 4827, 4826, 1595, 1596, 1607, 1606

2080, 4816, 4817, 4828, 4827, 1596, 1597, 1608, 1607

2081, 4817, 4818, 4829, 4828, 1597, 1598, 1609, 1608

2082, 4818, 4819, 4830, 4829, 1598, 1599, 1610, 1609

2083, 4819, 4820, 4831, 4830, 1599, 1600, 1611, 1610

2084, 4820, 4821, 4832, 4831, 1600, 1601, 1612, 1611

2085, 4821, 4822, 4833, 4832, 1601, 1602, 1613, 1612

2086, 4822, 4823, 4834, 4833, 1602, 1603, 1614, 1613

2087, 4823, 4824, 4835, 4834, 1603, 1604, 1615, 1614

2088, 4824, 2520, 2521, 4835, 1604, 222, 223, 1615

2089, 2331, 4825, 4836, 2332, 286, 1605, 1616, 285

2090, 4825, 4826, 4837, 4836, 1605, 1606, 1617, 1616

2091, 4826, 4827, 4838, 4837, 1606, 1607, 1618, 1617

2092, 4827, 4828, 4839, 4838, 1607, 1608, 1619, 1618

2093, 4828, 4829, 4840, 4839, 1608, 1609, 1620, 1619

2094, 4829, 4830, 4841, 4840, 1609, 1610, 1621, 1620

2095, 4830, 4831, 4842, 4841, 1610, 1611, 1622, 1621

2096, 4831, 4832, 4843, 4842, 1611, 1612, 1623, 1622

2097, 4832, 4833, 4844, 4843, 1612, 1613, 1624, 1623

2098, 4833, 4834, 4845, 4844, 1613, 1614, 1625, 1624

2099, 4834, 4835, 4846, 4845, 1614, 1615, 1626, 1625

2100, 4835, 2521, 2522, 4846, 1615, 223, 224, 1626

2101, 2332, 4836, 4847, 2333, 285, 1616, 1627, 284

2102, 4836, 4837, 4848, 4847, 1616, 1617, 1628, 1627

2103, 4837, 4838, 4849, 4848, 1617, 1618, 1629, 1628

2104, 4838, 4839, 4850, 4849, 1618, 1619, 1630, 1629

2105, 4839, 4840, 4851, 4850, 1619, 1620, 1631, 1630

2106, 4840, 4841, 4852, 4851, 1620, 1621, 1632, 1631

2107, 4841, 4842, 4853, 4852, 1621, 1622, 1633, 1632

2108, 4842, 4843, 4854, 4853, 1622, 1623, 1634, 1633

2109, 4843, 4844, 4855, 4854, 1623, 1624, 1635, 1634

2110, 4844, 4845, 4856, 4855, 1624, 1625, 1636, 1635

2111, 4845, 4846, 4857, 4856, 1625, 1626, 1637, 1636

2112, 4846, 2522, 2523, 4857, 1626, 224, 225, 1637

2113, 2333, 4847, 4858, 2334, 284, 1627, 1638, 283

2114, 4847, 4848, 4859, 4858, 1627, 1628, 1639, 1638

2115, 4848, 4849, 4860, 4859, 1628, 1629, 1640, 1639

2116, 4849, 4850, 4861, 4860, 1629, 1630, 1641, 1640

2117, 4850, 4851, 4862, 4861, 1630, 1631, 1642, 1641

2118, 4851, 4852, 4863, 4862, 1631, 1632, 1643, 1642

2119, 4852, 4853, 4864, 4863, 1632, 1633, 1644, 1643

2120, 4853, 4854, 4865, 4864, 1633, 1634, 1645, 1644

2121, 4854, 4855, 4866, 4865, 1634, 1635, 1646, 1645

2122, 4855, 4856, 4867, 4866, 1635, 1636, 1647, 1646

2123, 4856, 4857, 4868, 4867, 1636, 1637, 1648, 1647

2124, 4857, 2523, 2524, 4868, 1637, 225, 226, 1648

2125, 2334, 4858, 4869, 2335, 283, 1638, 1649, 282

2126, 4858, 4859, 4870, 4869, 1638, 1639, 1650, 1649

2127, 4859, 4860, 4871, 4870, 1639, 1640, 1651, 1650

2128, 4860, 4861, 4872, 4871, 1640, 1641, 1652, 1651

2129, 4861, 4862, 4873, 4872, 1641, 1642, 1653, 1652

2130, 4862, 4863, 4874, 4873, 1642, 1643, 1654, 1653

2131, 4863, 4864, 4875, 4874, 1643, 1644, 1655, 1654

2132, 4864, 4865, 4876, 4875, 1644, 1645, 1656, 1655

2133, 4865, 4866, 4877, 4876, 1645, 1646, 1657, 1656

2134, 4866, 4867, 4878, 4877, 1646, 1647, 1658, 1657

2135, 4867, 4868, 4879, 4878, 1647, 1648, 1659, 1658

2136, 4868, 2524, 2525, 4879, 1648, 226, 227, 1659

2137, 2335, 4869, 4880, 2336, 282, 1649, 1660, 281

2138, 4869, 4870, 4881, 4880, 1649, 1650, 1661, 1660

2139, 4870, 4871, 4882, 4881, 1650, 1651, 1662, 1661

2140, 4871, 4872, 4883, 4882, 1651, 1652, 1663, 1662

2141, 4872, 4873, 4884, 4883, 1652, 1653, 1664, 1663

2142, 4873, 4874, 4885, 4884, 1653, 1654, 1665, 1664

2143, 4874, 4875, 4886, 4885, 1654, 1655, 1666, 1665

2144, 4875, 4876, 4887, 4886, 1655, 1656, 1667, 1666

2145, 4876, 4877, 4888, 4887, 1656, 1657, 1668, 1667

2146, 4877, 4878, 4889, 4888, 1657, 1658, 1669, 1668

2147, 4878, 4879, 4890, 4889, 1658, 1659, 1670, 1669

2148, 4879, 2525, 2526, 4890, 1659, 227, 228, 1670

2149, 2336, 4880, 4891, 2337, 281, 1660, 1671, 280

2150, 4880, 4881, 4892, 4891, 1660, 1661, 1672, 1671

2151, 4881, 4882, 4893, 4892, 1661, 1662, 1673, 1672

2152, 4882, 4883, 4894, 4893, 1662, 1663, 1674, 1673

2153, 4883, 4884, 4895, 4894, 1663, 1664, 1675, 1674

2154, 4884, 4885, 4896, 4895, 1664, 1665, 1676, 1675

2155, 4885, 4886, 4897, 4896, 1665, 1666, 1677, 1676

2156, 4886, 4887, 4898, 4897, 1666, 1667, 1678, 1677

2157, 4887, 4888, 4899, 4898, 1667, 1668, 1679, 1678

2158, 4888, 4889, 4900, 4899, 1668, 1669, 1680, 1679

2159, 4889, 4890, 4901, 4900, 1669, 1670, 1681, 1680

2160, 4890, 2526, 2527, 4901, 1670, 228, 229, 1681

2161, 2337, 4891, 4902, 2338, 280, 1671, 1682, 279

2162, 4891, 4892, 4903, 4902, 1671, 1672, 1683, 1682

2163, 4892, 4893, 4904, 4903, 1672, 1673, 1684, 1683

2164, 4893, 4894, 4905, 4904, 1673, 1674, 1685, 1684

2165, 4894, 4895, 4906, 4905, 1674, 1675, 1686, 1685

2166, 4895, 4896, 4907, 4906, 1675, 1676, 1687, 1686

2167, 4896, 4897, 4908, 4907, 1676, 1677, 1688, 1687

2168, 4897, 4898, 4909, 4908, 1677, 1678, 1689, 1688

2169, 4898, 4899, 4910, 4909, 1678, 1679, 1690, 1689

2170, 4899, 4900, 4911, 4910, 1679, 1680, 1691, 1690

2171, 4900, 4901, 4912, 4911, 1680, 1681, 1692, 1691

2172, 4901, 2527, 2528, 4912, 1681, 229, 230, 1692

2173, 2338, 4902, 4913, 2339, 279, 1682, 1693, 278

2174, 4902, 4903, 4914, 4913, 1682, 1683, 1694, 1693

2175, 4903, 4904, 4915, 4914, 1683, 1684, 1695, 1694

2176, 4904, 4905, 4916, 4915, 1684, 1685, 1696, 1695

2177, 4905, 4906, 4917, 4916, 1685, 1686, 1697, 1696

2178, 4906, 4907, 4918, 4917, 1686, 1687, 1698, 1697

2179, 4907, 4908, 4919, 4918, 1687, 1688, 1699, 1698

2180, 4908, 4909, 4920, 4919, 1688, 1689, 1700, 1699

2181, 4909, 4910, 4921, 4920, 1689, 1690, 1701, 1700

2182, 4910, 4911, 4922, 4921, 1690, 1691, 1702, 1701

2183, 4911, 4912, 4923, 4922, 1691, 1692, 1703, 1702

2184, 4912, 2528, 2529, 4923, 1692, 230, 231, 1703

2185, 2339, 4913, 4924, 2340, 278, 1693, 1704, 277

2186, 4913, 4914, 4925, 4924, 1693, 1694, 1705, 1704

2187, 4914, 4915, 4926, 4925, 1694, 1695, 1706, 1705

2188, 4915, 4916, 4927, 4926, 1695, 1696, 1707, 1706

2189, 4916, 4917, 4928, 4927, 1696, 1697, 1708, 1707

2190, 4917, 4918, 4929, 4928, 1697, 1698, 1709, 1708

2191, 4918, 4919, 4930, 4929, 1698, 1699, 1710, 1709

2192, 4919, 4920, 4931, 4930, 1699, 1700, 1711, 1710

2193, 4920, 4921, 4932, 4931, 1700, 1701, 1712, 1711

2194, 4921, 4922, 4933, 4932, 1701, 1702, 1713, 1712

2195, 4922, 4923, 4934, 4933, 1702, 1703, 1714, 1713

2196, 4923, 2529, 2530, 4934, 1703, 231, 232, 1714

2197, 2340, 4924, 4935, 2341, 277, 1704, 1715, 276

2198, 4924, 4925, 4936, 4935, 1704, 1705, 1716, 1715

2199, 4925, 4926, 4937, 4936, 1705, 1706, 1717, 1716

2200, 4926, 4927, 4938, 4937, 1706, 1707, 1718, 1717

2201, 4927, 4928, 4939, 4938, 1707, 1708, 1719, 1718

2202, 4928, 4929, 4940, 4939, 1708, 1709, 1720, 1719

2203, 4929, 4930, 4941, 4940, 1709, 1710, 1721, 1720

2204, 4930, 4931, 4942, 4941, 1710, 1711, 1722, 1721

2205, 4931, 4932, 4943, 4942, 1711, 1712, 1723, 1722

2206, 4932, 4933, 4944, 4943, 1712, 1713, 1724, 1723

2207, 4933, 4934, 4945, 4944, 1713, 1714, 1725, 1724

2208, 4934, 2530, 2531, 4945, 1714, 232, 233, 1725

2209, 2341, 4935, 4946, 2342, 276, 1715, 1726, 275

2210, 4935, 4936, 4947, 4946, 1715, 1716, 1727, 1726

2211, 4936, 4937, 4948, 4947, 1716, 1717, 1728, 1727

2212, 4937, 4938, 4949, 4948, 1717, 1718, 1729, 1728

2213, 4938, 4939, 4950, 4949, 1718, 1719, 1730, 1729

2214, 4939, 4940, 4951, 4950, 1719, 1720, 1731, 1730

2215, 4940, 4941, 4952, 4951, 1720, 1721, 1732, 1731

2216, 4941, 4942, 4953, 4952, 1721, 1722, 1733, 1732

2217, 4942, 4943, 4954, 4953, 1722, 1723, 1734, 1733

2218, 4943, 4944, 4955, 4954, 1723, 1724, 1735, 1734

2219, 4944, 4945, 4956, 4955, 1724, 1725, 1736, 1735

2220, 4945, 2531, 2532, 4956, 1725, 233, 234, 1736

2221, 2342, 4946, 4957, 2343, 275, 1726, 1737, 274

2222, 4946, 4947, 4958, 4957, 1726, 1727, 1738, 1737

2223, 4947, 4948, 4959, 4958, 1727, 1728, 1739, 1738

2224, 4948, 4949, 4960, 4959, 1728, 1729, 1740, 1739

2225, 4949, 4950, 4961, 4960, 1729, 1730, 1741, 1740

2226, 4950, 4951, 4962, 4961, 1730, 1731, 1742, 1741

2227, 4951, 4952, 4963, 4962, 1731, 1732, 1743, 1742

2228, 4952, 4953, 4964, 4963, 1732, 1733, 1744, 1743

2229, 4953, 4954, 4965, 4964, 1733, 1734, 1745, 1744

2230, 4954, 4955, 4966, 4965, 1734, 1735, 1746, 1745

2231, 4955, 4956, 4967, 4966, 1735, 1736, 1747, 1746

2232, 4956, 2532, 2533, 4967, 1736, 234, 235, 1747

2233, 2343, 4957, 4968, 2344, 274, 1737, 1748, 273

2234, 4957, 4958, 4969, 4968, 1737, 1738, 1749, 1748

2235, 4958, 4959, 4970, 4969, 1738, 1739, 1750, 1749

2236, 4959, 4960, 4971, 4970, 1739, 1740, 1751, 1750

2237, 4960, 4961, 4972, 4971, 1740, 1741, 1752, 1751

2238, 4961, 4962, 4973, 4972, 1741, 1742, 1753, 1752

2239, 4962, 4963, 4974, 4973, 1742, 1743, 1754, 1753

2240, 4963, 4964, 4975, 4974, 1743, 1744, 1755, 1754

2241, 4964, 4965, 4976, 4975, 1744, 1745, 1756, 1755

2242, 4965, 4966, 4977, 4976, 1745, 1746, 1757, 1756

2243, 4966, 4967, 4978, 4977, 1746, 1747, 1758, 1757

2244, 4967, 2533, 2534, 4978, 1747, 235, 236, 1758

2245, 2344, 4968, 4979, 2345, 273, 1748, 1759, 272

2246, 4968, 4969, 4980, 4979, 1748, 1749, 1760, 1759

2247, 4969, 4970, 4981, 4980, 1749, 1750, 1761, 1760

2248, 4970, 4971, 4982, 4981, 1750, 1751, 1762, 1761

2249, 4971, 4972, 4983, 4982, 1751, 1752, 1763, 1762

2250, 4972, 4973, 4984, 4983, 1752, 1753, 1764, 1763

2251, 4973, 4974, 4985, 4984, 1753, 1754, 1765, 1764

2252, 4974, 4975, 4986, 4985, 1754, 1755, 1766, 1765

2253, 4975, 4976, 4987, 4986, 1755, 1756, 1767, 1766

2254, 4976, 4977, 4988, 4987, 1756, 1757, 1768, 1767

2255, 4977, 4978, 4989, 4988, 1757, 1758, 1769, 1768

2256, 4978, 2534, 2535, 4989, 1758, 236, 237, 1769

2257, 2345, 4979, 4990, 2346, 272, 1759, 1770, 271

2258, 4979, 4980, 4991, 4990, 1759, 1760, 1771, 1770

2259, 4980, 4981, 4992, 4991, 1760, 1761, 1772, 1771

2260, 4981, 4982, 4993, 4992, 1761, 1762, 1773, 1772

2261, 4982, 4983, 4994, 4993, 1762, 1763, 1774, 1773

2262, 4983, 4984, 4995, 4994, 1763, 1764, 1775, 1774

2263, 4984, 4985, 4996, 4995, 1764, 1765, 1776, 1775

2264, 4985, 4986, 4997, 4996, 1765, 1766, 1777, 1776

2265, 4986, 4987, 4998, 4997, 1766, 1767, 1778, 1777

2266, 4987, 4988, 4999, 4998, 1767, 1768, 1779, 1778

2267, 4988, 4989, 5000, 4999, 1768, 1769, 1780, 1779

2268, 4989, 2535, 2536, 5000, 1769, 237, 238, 1780

2269, 2346, 4990, 5001, 2347, 271, 1770, 1781, 270

2270, 4990, 4991, 5002, 5001, 1770, 1771, 1782, 1781

2271, 4991, 4992, 5003, 5002, 1771, 1772, 1783, 1782

2272, 4992, 4993, 5004, 5003, 1772, 1773, 1784, 1783

2273, 4993, 4994, 5005, 5004, 1773, 1774, 1785, 1784

2274, 4994, 4995, 5006, 5005, 1774, 1775, 1786, 1785

2275, 4995, 4996, 5007, 5006, 1775, 1776, 1787, 1786

2276, 4996, 4997, 5008, 5007, 1776, 1777, 1788, 1787

2277, 4997, 4998, 5009, 5008, 1777, 1778, 1789, 1788

2278, 4998, 4999, 5010, 5009, 1778, 1779, 1790, 1789

2279, 4999, 5000, 5011, 5010, 1779, 1780, 1791, 1790

2280, 5000, 2536, 2537, 5011, 1780, 238, 239, 1791

2281, 2347, 5001, 5012, 2348, 270, 1781, 1792, 269

2282, 5001, 5002, 5013, 5012, 1781, 1782, 1793, 1792

2283, 5002, 5003, 5014, 5013, 1782, 1783, 1794, 1793

2284, 5003, 5004, 5015, 5014, 1783, 1784, 1795, 1794

2285, 5004, 5005, 5016, 5015, 1784, 1785, 1796, 1795

2286, 5005, 5006, 5017, 5016, 1785, 1786, 1797, 1796

2287, 5006, 5007, 5018, 5017, 1786, 1787, 1798, 1797

2288, 5007, 5008, 5019, 5018, 1787, 1788, 1799, 1798

2289, 5008, 5009, 5020, 5019, 1788, 1789, 1800, 1799

2290, 5009, 5010, 5021, 5020, 1789, 1790, 1801, 1800

2291, 5010, 5011, 5022, 5021, 1790, 1791, 1802, 1801

2292, 5011, 2537, 2538, 5022, 1791, 239, 240, 1802

2293, 2348, 5012, 5023, 2349, 269, 1792, 1803, 268

2294, 5012, 5013, 5024, 5023, 1792, 1793, 1804, 1803

2295, 5013, 5014, 5025, 5024, 1793, 1794, 1805, 1804

2296, 5014, 5015, 5026, 5025, 1794, 1795, 1806, 1805

2297, 5015, 5016, 5027, 5026, 1795, 1796, 1807, 1806

2298, 5016, 5017, 5028, 5027, 1796, 1797, 1808, 1807

2299, 5017, 5018, 5029, 5028, 1797, 1798, 1809, 1808

2300, 5018, 5019, 5030, 5029, 1798, 1799, 1810, 1809

2301, 5019, 5020, 5031, 5030, 1799, 1800, 1811, 1810

2302, 5020, 5021, 5032, 5031, 1800, 1801, 1812, 1811

2303, 5021, 5022, 5033, 5032, 1801, 1802, 1813, 1812

2304, 5022, 2538, 2539, 5033, 1802, 240, 241, 1813

2305, 2349, 5023, 5034, 2350, 268, 1803, 1814, 267

2306, 5023, 5024, 5035, 5034, 1803, 1804, 1815, 1814

2307, 5024, 5025, 5036, 5035, 1804, 1805, 1816, 1815

2308, 5025, 5026, 5037, 5036, 1805, 1806, 1817, 1816

2309, 5026, 5027, 5038, 5037, 1806, 1807, 1818, 1817

2310, 5027, 5028, 5039, 5038, 1807, 1808, 1819, 1818

2311, 5028, 5029, 5040, 5039, 1808, 1809, 1820, 1819

2312, 5029, 5030, 5041, 5040, 1809, 1810, 1821, 1820

2313, 5030, 5031, 5042, 5041, 1810, 1811, 1822, 1821

2314, 5031, 5032, 5043, 5042, 1811, 1812, 1823, 1822

2315, 5032, 5033, 5044, 5043, 1812, 1813, 1824, 1823

2316, 5033, 2539, 2540, 5044, 1813, 241, 242, 1824

2317, 2350, 5034, 5045, 2351, 267, 1814, 1825, 266

2318, 5034, 5035, 5046, 5045, 1814, 1815, 1826, 1825

2319, 5035, 5036, 5047, 5046, 1815, 1816, 1827, 1826

2320, 5036, 5037, 5048, 5047, 1816, 1817, 1828, 1827

2321, 5037, 5038, 5049, 5048, 1817, 1818, 1829, 1828

2322, 5038, 5039, 5050, 5049, 1818, 1819, 1830, 1829

2323, 5039, 5040, 5051, 5050, 1819, 1820, 1831, 1830

2324, 5040, 5041, 5052, 5051, 1820, 1821, 1832, 1831

2325, 5041, 5042, 5053, 5052, 1821, 1822, 1833, 1832

2326, 5042, 5043, 5054, 5053, 1822, 1823, 1834, 1833

2327, 5043, 5044, 5055, 5054, 1823, 1824, 1835, 1834

2328, 5044, 2540, 2541, 5055, 1824, 242, 243, 1835

2329, 2351, 5045, 5056, 2352, 266, 1825, 1836, 265

2330, 5045, 5046, 5057, 5056, 1825, 1826, 1837, 1836

2331, 5046, 5047, 5058, 5057, 1826, 1827, 1838, 1837

2332, 5047, 5048, 5059, 5058, 1827, 1828, 1839, 1838

2333, 5048, 5049, 5060, 5059, 1828, 1829, 1840, 1839

2334, 5049, 5050, 5061, 5060, 1829, 1830, 1841, 1840

2335, 5050, 5051, 5062, 5061, 1830, 1831, 1842, 1841

2336, 5051, 5052, 5063, 5062, 1831, 1832, 1843, 1842

2337, 5052, 5053, 5064, 5063, 1832, 1833, 1844, 1843

2338, 5053, 5054, 5065, 5064, 1833, 1834, 1845, 1844

2339, 5054, 5055, 5066, 5065, 1834, 1835, 1846, 1845

2340, 5055, 2541, 2542, 5066, 1835, 243, 244, 1846

2341, 2352, 5056, 5067, 2353, 265, 1836, 1847, 264

2342, 5056, 5057, 5068, 5067, 1836, 1837, 1848, 1847

2343, 5057, 5058, 5069, 5068, 1837, 1838, 1849, 1848

2344, 5058, 5059, 5070, 5069, 1838, 1839, 1850, 1849

2345, 5059, 5060, 5071, 5070, 1839, 1840, 1851, 1850

2346, 5060, 5061, 5072, 5071, 1840, 1841, 1852, 1851

2347, 5061, 5062, 5073, 5072, 1841, 1842, 1853, 1852

2348, 5062, 5063, 5074, 5073, 1842, 1843, 1854, 1853

2349, 5063, 5064, 5075, 5074, 1843, 1844, 1855, 1854

2350, 5064, 5065, 5076, 5075, 1844, 1845, 1856, 1855

2351, 5065, 5066, 5077, 5076, 1845, 1846, 1857, 1856

2352, 5066, 2542, 2543, 5077, 1846, 244, 245, 1857

2353, 2353, 5067, 5078, 2354, 264, 1847, 1858, 263

2354, 5067, 5068, 5079, 5078, 1847, 1848, 1859, 1858

2355, 5068, 5069, 5080, 5079, 1848, 1849, 1860, 1859

2356, 5069, 5070, 5081, 5080, 1849, 1850, 1861, 1860

2357, 5070, 5071, 5082, 5081, 1850, 1851, 1862, 1861

2358, 5071, 5072, 5083, 5082, 1851, 1852, 1863, 1862

2359, 5072, 5073, 5084, 5083, 1852, 1853, 1864, 1863

2360, 5073, 5074, 5085, 5084, 1853, 1854, 1865, 1864

2361, 5074, 5075, 5086, 5085, 1854, 1855, 1866, 1865

2362, 5075, 5076, 5087, 5086, 1855, 1856, 1867, 1866

2363, 5076, 5077, 5088, 5087, 1856, 1857, 1868, 1867

2364, 5077, 2543, 2544, 5088, 1857, 245, 246, 1868

2365, 2354, 5078, 5089, 2355, 263, 1858, 1869, 262

2366, 5078, 5079, 5090, 5089, 1858, 1859, 1870, 1869

2367, 5079, 5080, 5091, 5090, 1859, 1860, 1871, 1870

2368, 5080, 5081, 5092, 5091, 1860, 1861, 1872, 1871

2369, 5081, 5082, 5093, 5092, 1861, 1862, 1873, 1872

2370, 5082, 5083, 5094, 5093, 1862, 1863, 1874, 1873

2371, 5083, 5084, 5095, 5094, 1863, 1864, 1875, 1874

2372, 5084, 5085, 5096, 5095, 1864, 1865, 1876, 1875

2373, 5085, 5086, 5097, 5096, 1865, 1866, 1877, 1876

2374, 5086, 5087, 5098, 5097, 1866, 1867, 1878, 1877

2375, 5087, 5088, 5099, 5098, 1867, 1868, 1879, 1878

2376, 5088, 2544, 2545, 5099, 1868, 246, 247, 1879

2377, 2355, 5089, 5100, 2356, 262, 1869, 1880, 261

2378, 5089, 5090, 5101, 5100, 1869, 1870, 1881, 1880

2379, 5090, 5091, 5102, 5101, 1870, 1871, 1882, 1881

2380, 5091, 5092, 5103, 5102, 1871, 1872, 1883, 1882

2381, 5092, 5093, 5104, 5103, 1872, 1873, 1884, 1883

2382, 5093, 5094, 5105, 5104, 1873, 1874, 1885, 1884

2383, 5094, 5095, 5106, 5105, 1874, 1875, 1886, 1885

2384, 5095, 5096, 5107, 5106, 1875, 1876, 1887, 1886

2385, 5096, 5097, 5108, 5107, 1876, 1877, 1888, 1887

2386, 5097, 5098, 5109, 5108, 1877, 1878, 1889, 1888

2387, 5098, 5099, 5110, 5109, 1878, 1879, 1890, 1889

2388, 5099, 2545, 2546, 5110, 1879, 247, 248, 1890

2389, 2356, 5100, 5111, 2357, 261, 1880, 1891, 260

2390, 5100, 5101, 5112, 5111, 1880, 1881, 1892, 1891

2391, 5101, 5102, 5113, 5112, 1881, 1882, 1893, 1892

2392, 5102, 5103, 5114, 5113, 1882, 1883, 1894, 1893

2393, 5103, 5104, 5115, 5114, 1883, 1884, 1895, 1894

2394, 5104, 5105, 5116, 5115, 1884, 1885, 1896, 1895

2395, 5105, 5106, 5117, 5116, 1885, 1886, 1897, 1896

2396, 5106, 5107, 5118, 5117, 1886, 1887, 1898, 1897

2397, 5107, 5108, 5119, 5118, 1887, 1888, 1899, 1898

2398, 5108, 5109, 5120, 5119, 1888, 1889, 1900, 1899

2399, 5109, 5110, 5121, 5120, 1889, 1890, 1901, 1900

2400, 5110, 2546, 2547, 5121, 1890, 248, 249, 1901

2401, 2357, 5111, 2755, 396, 260, 1891, 254, 13

2402, 5111, 5112, 2756, 2755, 1891, 1892, 253, 254

2403, 5112, 5113, 2757, 2756, 1892, 1893, 252, 253

2404, 5113, 5114, 2758, 2757, 1893, 1894, 251, 252

2405, 5114, 5115, 2759, 2758, 1894, 1895, 250, 251

2406, 5115, 5116, 2760, 2759, 1895, 1896, 11, 250

2407, 5116, 5117, 2761, 2760, 1896, 1897, 259, 11

2408, 5117, 5118, 2762, 2761, 1897, 1898, 258, 259

2409, 5118, 5119, 2763, 2762, 1898, 1899, 257, 258

2410, 5119, 5120, 2764, 2763, 1899, 1900, 256, 257

2411, 5120, 5121, 2765, 2764, 1900, 1901, 255, 256

2412, 5121, 2547, 410, 2765, 1901, 249, 12, 255

2413, 404, 2711, 5122, 2358, 405, 2700, 4704, 2320

2414, 2711, 2712, 5123, 5122, 2700, 2701, 4705, 4704

2415, 2712, 2713, 5124, 5123, 2701, 2702, 4706, 4705

2416, 2713, 2714, 5125, 5124, 2702, 2703, 4707, 4706

2417, 2714, 2715, 5126, 5125, 2703, 2704, 4708, 4707

2418, 2715, 2716, 5127, 5126, 2704, 2705, 4709, 4708

2419, 2716, 2717, 5128, 5127, 2705, 2706, 4710, 4709

2420, 2717, 2718, 5129, 5128, 2706, 2707, 4711, 4710

2421, 2718, 2719, 5130, 5129, 2707, 2708, 4712, 4711

2422, 2719, 2720, 5131, 5130, 2708, 2709, 4713, 4712

2423, 2720, 2721, 5132, 5131, 2709, 2710, 4714, 4713

2424, 2721, 412, 2548, 5132, 2710, 411, 2510, 4714

2425, 2358, 5122, 5133, 2359, 2320, 4704, 4715, 2321

2426, 5122, 5123, 5134, 5133, 4704, 4705, 4716, 4715

2427, 5123, 5124, 5135, 5134, 4705, 4706, 4717, 4716

2428, 5124, 5125, 5136, 5135, 4706, 4707, 4718, 4717

2429, 5125, 5126, 5137, 5136, 4707, 4708, 4719, 4718

2430, 5126, 5127, 5138, 5137, 4708, 4709, 4720, 4719

2431, 5127, 5128, 5139, 5138, 4709, 4710, 4721, 4720

2432, 5128, 5129, 5140, 5139, 4710, 4711, 4722, 4721

2433, 5129, 5130, 5141, 5140, 4711, 4712, 4723, 4722

2434, 5130, 5131, 5142, 5141, 4712, 4713, 4724, 4723

2435, 5131, 5132, 5143, 5142, 4713, 4714, 4725, 4724

2436, 5132, 2548, 2549, 5143, 4714, 2510, 2511, 4725

2437, 2359, 5133, 5144, 2360, 2321, 4715, 4726, 2322

2438, 5133, 5134, 5145, 5144, 4715, 4716, 4727, 4726

2439, 5134, 5135, 5146, 5145, 4716, 4717, 4728, 4727

2440, 5135, 5136, 5147, 5146, 4717, 4718, 4729, 4728

2441, 5136, 5137, 5148, 5147, 4718, 4719, 4730, 4729

2442, 5137, 5138, 5149, 5148, 4719, 4720, 4731, 4730

2443, 5138, 5139, 5150, 5149, 4720, 4721, 4732, 4731

2444, 5139, 5140, 5151, 5150, 4721, 4722, 4733, 4732

2445, 5140, 5141, 5152, 5151, 4722, 4723, 4734, 4733

2446, 5141, 5142, 5153, 5152, 4723, 4724, 4735, 4734

2447, 5142, 5143, 5154, 5153, 4724, 4725, 4736, 4735

2448, 5143, 2549, 2550, 5154, 4725, 2511, 2512, 4736

2449, 2360, 5144, 5155, 2361, 2322, 4726, 4737, 2323

2450, 5144, 5145, 5156, 5155, 4726, 4727, 4738, 4737

2451, 5145, 5146, 5157, 5156, 4727, 4728, 4739, 4738

2452, 5146, 5147, 5158, 5157, 4728, 4729, 4740, 4739

2453, 5147, 5148, 5159, 5158, 4729, 4730, 4741, 4740

2454, 5148, 5149, 5160, 5159, 4730, 4731, 4742, 4741

2455, 5149, 5150, 5161, 5160, 4731, 4732, 4743, 4742

2456, 5150, 5151, 5162, 5161, 4732, 4733, 4744, 4743

2457, 5151, 5152, 5163, 5162, 4733, 4734, 4745, 4744

2458, 5152, 5153, 5164, 5163, 4734, 4735, 4746, 4745

2459, 5153, 5154, 5165, 5164, 4735, 4736, 4747, 4746

2460, 5154, 2550, 2551, 5165, 4736, 2512, 2513, 4747

2461, 2361, 5155, 5166, 2362, 2323, 4737, 4748, 2324

2462, 5155, 5156, 5167, 5166, 4737, 4738, 4749, 4748

2463, 5156, 5157, 5168, 5167, 4738, 4739, 4750, 4749

2464, 5157, 5158, 5169, 5168, 4739, 4740, 4751, 4750

2465, 5158, 5159, 5170, 5169, 4740, 4741, 4752, 4751

2466, 5159, 5160, 5171, 5170, 4741, 4742, 4753, 4752

2467, 5160, 5161, 5172, 5171, 4742, 4743, 4754, 4753

2468, 5161, 5162, 5173, 5172, 4743, 4744, 4755, 4754

2469, 5162, 5163, 5174, 5173, 4744, 4745, 4756, 4755

2470, 5163, 5164, 5175, 5174, 4745, 4746, 4757, 4756

2471, 5164, 5165, 5176, 5175, 4746, 4747, 4758, 4757

2472, 5165, 2551, 2552, 5176, 4747, 2513, 2514, 4758

2473, 2362, 5166, 5177, 2363, 2324, 4748, 4759, 2325

2474, 5166, 5167, 5178, 5177, 4748, 4749, 4760, 4759

2475, 5167, 5168, 5179, 5178, 4749, 4750, 4761, 4760

2476, 5168, 5169, 5180, 5179, 4750, 4751, 4762, 4761

2477, 5169, 5170, 5181, 5180, 4751, 4752, 4763, 4762

2478, 5170, 5171, 5182, 5181, 4752, 4753, 4764, 4763

2479, 5171, 5172, 5183, 5182, 4753, 4754, 4765, 4764

2480, 5172, 5173, 5184, 5183, 4754, 4755, 4766, 4765

2481, 5173, 5174, 5185, 5184, 4755, 4756, 4767, 4766

2482, 5174, 5175, 5186, 5185, 4756, 4757, 4768, 4767

2483, 5175, 5176, 5187, 5186, 4757, 4758, 4769, 4768

2484, 5176, 2552, 2553, 5187, 4758, 2514, 2515, 4769

2485, 2363, 5177, 5188, 2364, 2325, 4759, 4770, 2326

2486, 5177, 5178, 5189, 5188, 4759, 4760, 4771, 4770

2487, 5178, 5179, 5190, 5189, 4760, 4761, 4772, 4771

2488, 5179, 5180, 5191, 5190, 4761, 4762, 4773, 4772

2489, 5180, 5181, 5192, 5191, 4762, 4763, 4774, 4773

2490, 5181, 5182, 5193, 5192, 4763, 4764, 4775, 4774

2491, 5182, 5183, 5194, 5193, 4764, 4765, 4776, 4775

2492, 5183, 5184, 5195, 5194, 4765, 4766, 4777, 4776

2493, 5184, 5185, 5196, 5195, 4766, 4767, 4778, 4777

2494, 5185, 5186, 5197, 5196, 4767, 4768, 4779, 4778

2495, 5186, 5187, 5198, 5197, 4768, 4769, 4780, 4779

2496, 5187, 2553, 2554, 5198, 4769, 2515, 2516, 4780

2497, 2364, 5188, 5199, 2365, 2326, 4770, 4781, 2327

2498, 5188, 5189, 5200, 5199, 4770, 4771, 4782, 4781

2499, 5189, 5190, 5201, 5200, 4771, 4772, 4783, 4782

2500, 5190, 5191, 5202, 5201, 4772, 4773, 4784, 4783

2501, 5191, 5192, 5203, 5202, 4773, 4774, 4785, 4784

2502, 5192, 5193, 5204, 5203, 4774, 4775, 4786, 4785

2503, 5193, 5194, 5205, 5204, 4775, 4776, 4787, 4786

2504, 5194, 5195, 5206, 5205, 4776, 4777, 4788, 4787

2505, 5195, 5196, 5207, 5206, 4777, 4778, 4789, 4788

2506, 5196, 5197, 5208, 5207, 4778, 4779, 4790, 4789

2507, 5197, 5198, 5209, 5208, 4779, 4780, 4791, 4790

2508, 5198, 2554, 2555, 5209, 4780, 2516, 2517, 4791

2509, 2365, 5199, 5210, 2366, 2327, 4781, 4792, 2328

2510, 5199, 5200, 5211, 5210, 4781, 4782, 4793, 4792

2511, 5200, 5201, 5212, 5211, 4782, 4783, 4794, 4793

2512, 5201, 5202, 5213, 5212, 4783, 4784, 4795, 4794

2513, 5202, 5203, 5214, 5213, 4784, 4785, 4796, 4795

2514, 5203, 5204, 5215, 5214, 4785, 4786, 4797, 4796

2515, 5204, 5205, 5216, 5215, 4786, 4787, 4798, 4797

2516, 5205, 5206, 5217, 5216, 4787, 4788, 4799, 4798

2517, 5206, 5207, 5218, 5217, 4788, 4789, 4800, 4799

2518, 5207, 5208, 5219, 5218, 4789, 4790, 4801, 4800

2519, 5208, 5209, 5220, 5219, 4790, 4791, 4802, 4801

2520, 5209, 2555, 2556, 5220, 4791, 2517, 2518, 4802

2521, 2366, 5210, 5221, 2367, 2328, 4792, 4803, 2329

2522, 5210, 5211, 5222, 5221, 4792, 4793, 4804, 4803

2523, 5211, 5212, 5223, 5222, 4793, 4794, 4805, 4804

2524, 5212, 5213, 5224, 5223, 4794, 4795, 4806, 4805

2525, 5213, 5214, 5225, 5224, 4795, 4796, 4807, 4806

2526, 5214, 5215, 5226, 5225, 4796, 4797, 4808, 4807

2527, 5215, 5216, 5227, 5226, 4797, 4798, 4809, 4808

2528, 5216, 5217, 5228, 5227, 4798, 4799, 4810, 4809

2529, 5217, 5218, 5229, 5228, 4799, 4800, 4811, 4810

2530, 5218, 5219, 5230, 5229, 4800, 4801, 4812, 4811

2531, 5219, 5220, 5231, 5230, 4801, 4802, 4813, 4812

2532, 5220, 2556, 2557, 5231, 4802, 2518, 2519, 4813

2533, 2367, 5221, 5232, 2368, 2329, 4803, 4814, 2330

2534, 5221, 5222, 5233, 5232, 4803, 4804, 4815, 4814

2535, 5222, 5223, 5234, 5233, 4804, 4805, 4816, 4815

2536, 5223, 5224, 5235, 5234, 4805, 4806, 4817, 4816

2537, 5224, 5225, 5236, 5235, 4806, 4807, 4818, 4817

2538, 5225, 5226, 5237, 5236, 4807, 4808, 4819, 4818

2539, 5226, 5227, 5238, 5237, 4808, 4809, 4820, 4819

2540, 5227, 5228, 5239, 5238, 4809, 4810, 4821, 4820

2541, 5228, 5229, 5240, 5239, 4810, 4811, 4822, 4821

2542, 5229, 5230, 5241, 5240, 4811, 4812, 4823, 4822

2543, 5230, 5231, 5242, 5241, 4812, 4813, 4824, 4823

2544, 5231, 2557, 2558, 5242, 4813, 2519, 2520, 4824

2545, 2368, 5232, 5243, 2369, 2330, 4814, 4825, 2331

2546, 5232, 5233, 5244, 5243, 4814, 4815, 4826, 4825

2547, 5233, 5234, 5245, 5244, 4815, 4816, 4827, 4826

2548, 5234, 5235, 5246, 5245, 4816, 4817, 4828, 4827

2549, 5235, 5236, 5247, 5246, 4817, 4818, 4829, 4828

2550, 5236, 5237, 5248, 5247, 4818, 4819, 4830, 4829

2551, 5237, 5238, 5249, 5248, 4819, 4820, 4831, 4830

2552, 5238, 5239, 5250, 5249, 4820, 4821, 4832, 4831

2553, 5239, 5240, 5251, 5250, 4821, 4822, 4833, 4832

2554, 5240, 5241, 5252, 5251, 4822, 4823, 4834, 4833

2555, 5241, 5242, 5253, 5252, 4823, 4824, 4835, 4834

2556, 5242, 2558, 2559, 5253, 4824, 2520, 2521, 4835

2557, 2369, 5243, 5254, 2370, 2331, 4825, 4836, 2332

2558, 5243, 5244, 5255, 5254, 4825, 4826, 4837, 4836

2559, 5244, 5245, 5256, 5255, 4826, 4827, 4838, 4837

2560, 5245, 5246, 5257, 5256, 4827, 4828, 4839, 4838

2561, 5246, 5247, 5258, 5257, 4828, 4829, 4840, 4839

2562, 5247, 5248, 5259, 5258, 4829, 4830, 4841, 4840

2563, 5248, 5249, 5260, 5259, 4830, 4831, 4842, 4841

2564, 5249, 5250, 5261, 5260, 4831, 4832, 4843, 4842

2565, 5250, 5251, 5262, 5261, 4832, 4833, 4844, 4843

2566, 5251, 5252, 5263, 5262, 4833, 4834, 4845, 4844

2567, 5252, 5253, 5264, 5263, 4834, 4835, 4846, 4845

2568, 5253, 2559, 2560, 5264, 4835, 2521, 2522, 4846

2569, 2370, 5254, 5265, 2371, 2332, 4836, 4847, 2333

2570, 5254, 5255, 5266, 5265, 4836, 4837, 4848, 4847

2571, 5255, 5256, 5267, 5266, 4837, 4838, 4849, 4848

2572, 5256, 5257, 5268, 5267, 4838, 4839, 4850, 4849

2573, 5257, 5258, 5269, 5268, 4839, 4840, 4851, 4850

2574, 5258, 5259, 5270, 5269, 4840, 4841, 4852, 4851

2575, 5259, 5260, 5271, 5270, 4841, 4842, 4853, 4852

2576, 5260, 5261, 5272, 5271, 4842, 4843, 4854, 4853

2577, 5261, 5262, 5273, 5272, 4843, 4844, 4855, 4854

2578, 5262, 5263, 5274, 5273, 4844, 4845, 4856, 4855

2579, 5263, 5264, 5275, 5274, 4845, 4846, 4857, 4856

2580, 5264, 2560, 2561, 5275, 4846, 2522, 2523, 4857

2581, 2371, 5265, 5276, 2372, 2333, 4847, 4858, 2334

2582, 5265, 5266, 5277, 5276, 4847, 4848, 4859, 4858

2583, 5266, 5267, 5278, 5277, 4848, 4849, 4860, 4859

2584, 5267, 5268, 5279, 5278, 4849, 4850, 4861, 4860

2585, 5268, 5269, 5280, 5279, 4850, 4851, 4862, 4861

2586, 5269, 5270, 5281, 5280, 4851, 4852, 4863, 4862

2587, 5270, 5271, 5282, 5281, 4852, 4853, 4864, 4863

2588, 5271, 5272, 5283, 5282, 4853, 4854, 4865, 4864

2589, 5272, 5273, 5284, 5283, 4854, 4855, 4866, 4865

2590, 5273, 5274, 5285, 5284, 4855, 4856, 4867, 4866

2591, 5274, 5275, 5286, 5285, 4856, 4857, 4868, 4867

2592, 5275, 2561, 2562, 5286, 4857, 2523, 2524, 4868

2593, 2372, 5276, 5287, 2373, 2334, 4858, 4869, 2335

2594, 5276, 5277, 5288, 5287, 4858, 4859, 4870, 4869

2595, 5277, 5278, 5289, 5288, 4859, 4860, 4871, 4870

2596, 5278, 5279, 5290, 5289, 4860, 4861, 4872, 4871

2597, 5279, 5280, 5291, 5290, 4861, 4862, 4873, 4872

2598, 5280, 5281, 5292, 5291, 4862, 4863, 4874, 4873

2599, 5281, 5282, 5293, 5292, 4863, 4864, 4875, 4874

2600, 5282, 5283, 5294, 5293, 4864, 4865, 4876, 4875

2601, 5283, 5284, 5295, 5294, 4865, 4866, 4877, 4876

2602, 5284, 5285, 5296, 5295, 4866, 4867, 4878, 4877

2603, 5285, 5286, 5297, 5296, 4867, 4868, 4879, 4878

2604, 5286, 2562, 2563, 5297, 4868, 2524, 2525, 4879

2605, 2373, 5287, 5298, 2374, 2335, 4869, 4880, 2336

2606, 5287, 5288, 5299, 5298, 4869, 4870, 4881, 4880

2607, 5288, 5289, 5300, 5299, 4870, 4871, 4882, 4881

2608, 5289, 5290, 5301, 5300, 4871, 4872, 4883, 4882

2609, 5290, 5291, 5302, 5301, 4872, 4873, 4884, 4883

2610, 5291, 5292, 5303, 5302, 4873, 4874, 4885, 4884

2611, 5292, 5293, 5304, 5303, 4874, 4875, 4886, 4885

2612, 5293, 5294, 5305, 5304, 4875, 4876, 4887, 4886

2613, 5294, 5295, 5306, 5305, 4876, 4877, 4888, 4887

2614, 5295, 5296, 5307, 5306, 4877, 4878, 4889, 4888

2615, 5296, 5297, 5308, 5307, 4878, 4879, 4890, 4889

2616, 5297, 2563, 2564, 5308, 4879, 2525, 2526, 4890

2617, 2374, 5298, 5309, 2375, 2336, 4880, 4891, 2337

2618, 5298, 5299, 5310, 5309, 4880, 4881, 4892, 4891

2619, 5299, 5300, 5311, 5310, 4881, 4882, 4893, 4892

2620, 5300, 5301, 5312, 5311, 4882, 4883, 4894, 4893

2621, 5301, 5302, 5313, 5312, 4883, 4884, 4895, 4894

2622, 5302, 5303, 5314, 5313, 4884, 4885, 4896, 4895

2623, 5303, 5304, 5315, 5314, 4885, 4886, 4897, 4896

2624, 5304, 5305, 5316, 5315, 4886, 4887, 4898, 4897

2625, 5305, 5306, 5317, 5316, 4887, 4888, 4899, 4898

2626, 5306, 5307, 5318, 5317, 4888, 4889, 4900, 4899

2627, 5307, 5308, 5319, 5318, 4889, 4890, 4901, 4900

2628, 5308, 2564, 2565, 5319, 4890, 2526, 2527, 4901

2629, 2375, 5309, 5320, 2376, 2337, 4891, 4902, 2338

2630, 5309, 5310, 5321, 5320, 4891, 4892, 4903, 4902

2631, 5310, 5311, 5322, 5321, 4892, 4893, 4904, 4903

2632, 5311, 5312, 5323, 5322, 4893, 4894, 4905, 4904

2633, 5312, 5313, 5324, 5323, 4894, 4895, 4906, 4905

2634, 5313, 5314, 5325, 5324, 4895, 4896, 4907, 4906

2635, 5314, 5315, 5326, 5325, 4896, 4897, 4908, 4907

2636, 5315, 5316, 5327, 5326, 4897, 4898, 4909, 4908

2637, 5316, 5317, 5328, 5327, 4898, 4899, 4910, 4909

2638, 5317, 5318, 5329, 5328, 4899, 4900, 4911, 4910

2639, 5318, 5319, 5330, 5329, 4900, 4901, 4912, 4911

2640, 5319, 2565, 2566, 5330, 4901, 2527, 2528, 4912

2641, 2376, 5320, 5331, 2377, 2338, 4902, 4913, 2339

2642, 5320, 5321, 5332, 5331, 4902, 4903, 4914, 4913

2643, 5321, 5322, 5333, 5332, 4903, 4904, 4915, 4914

2644, 5322, 5323, 5334, 5333, 4904, 4905, 4916, 4915

2645, 5323, 5324, 5335, 5334, 4905, 4906, 4917, 4916

2646, 5324, 5325, 5336, 5335, 4906, 4907, 4918, 4917

2647, 5325, 5326, 5337, 5336, 4907, 4908, 4919, 4918

2648, 5326, 5327, 5338, 5337, 4908, 4909, 4920, 4919

2649, 5327, 5328, 5339, 5338, 4909, 4910, 4921, 4920

2650, 5328, 5329, 5340, 5339, 4910, 4911, 4922, 4921

2651, 5329, 5330, 5341, 5340, 4911, 4912, 4923, 4922

2652, 5330, 2566, 2567, 5341, 4912, 2528, 2529, 4923

2653, 2377, 5331, 5342, 2378, 2339, 4913, 4924, 2340

2654, 5331, 5332, 5343, 5342, 4913, 4914, 4925, 4924

2655, 5332, 5333, 5344, 5343, 4914, 4915, 4926, 4925

2656, 5333, 5334, 5345, 5344, 4915, 4916, 4927, 4926

2657, 5334, 5335, 5346, 5345, 4916, 4917, 4928, 4927

2658, 5335, 5336, 5347, 5346, 4917, 4918, 4929, 4928

2659, 5336, 5337, 5348, 5347, 4918, 4919, 4930, 4929

2660, 5337, 5338, 5349, 5348, 4919, 4920, 4931, 4930

2661, 5338, 5339, 5350, 5349, 4920, 4921, 4932, 4931

2662, 5339, 5340, 5351, 5350, 4921, 4922, 4933, 4932

2663, 5340, 5341, 5352, 5351, 4922, 4923, 4934, 4933

2664, 5341, 2567, 2568, 5352, 4923, 2529, 2530, 4934

2665, 2378, 5342, 5353, 2379, 2340, 4924, 4935, 2341

2666, 5342, 5343, 5354, 5353, 4924, 4925, 4936, 4935

2667, 5343, 5344, 5355, 5354, 4925, 4926, 4937, 4936

2668, 5344, 5345, 5356, 5355, 4926, 4927, 4938, 4937

2669, 5345, 5346, 5357, 5356, 4927, 4928, 4939, 4938

2670, 5346, 5347, 5358, 5357, 4928, 4929, 4940, 4939

2671, 5347, 5348, 5359, 5358, 4929, 4930, 4941, 4940

2672, 5348, 5349, 5360, 5359, 4930, 4931, 4942, 4941

2673, 5349, 5350, 5361, 5360, 4931, 4932, 4943, 4942

2674, 5350, 5351, 5362, 5361, 4932, 4933, 4944, 4943

2675, 5351, 5352, 5363, 5362, 4933, 4934, 4945, 4944

2676, 5352, 2568, 2569, 5363, 4934, 2530, 2531, 4945

2677, 2379, 5353, 5364, 2380, 2341, 4935, 4946, 2342

2678, 5353, 5354, 5365, 5364, 4935, 4936, 4947, 4946

2679, 5354, 5355, 5366, 5365, 4936, 4937, 4948, 4947

2680, 5355, 5356, 5367, 5366, 4937, 4938, 4949, 4948

2681, 5356, 5357, 5368, 5367, 4938, 4939, 4950, 4949

2682, 5357, 5358, 5369, 5368, 4939, 4940, 4951, 4950

2683, 5358, 5359, 5370, 5369, 4940, 4941, 4952, 4951

2684, 5359, 5360, 5371, 5370, 4941, 4942, 4953, 4952

2685, 5360, 5361, 5372, 5371, 4942, 4943, 4954, 4953

2686, 5361, 5362, 5373, 5372, 4943, 4944, 4955, 4954

2687, 5362, 5363, 5374, 5373, 4944, 4945, 4956, 4955

2688, 5363, 2569, 2570, 5374, 4945, 2531, 2532, 4956

2689, 2380, 5364, 5375, 2381, 2342, 4946, 4957, 2343

2690, 5364, 5365, 5376, 5375, 4946, 4947, 4958, 4957

2691, 5365, 5366, 5377, 5376, 4947, 4948, 4959, 4958

2692, 5366, 5367, 5378, 5377, 4948, 4949, 4960, 4959

2693, 5367, 5368, 5379, 5378, 4949, 4950, 4961, 4960

2694, 5368, 5369, 5380, 5379, 4950, 4951, 4962, 4961

2695, 5369, 5370, 5381, 5380, 4951, 4952, 4963, 4962

2696, 5370, 5371, 5382, 5381, 4952, 4953, 4964, 4963

2697, 5371, 5372, 5383, 5382, 4953, 4954, 4965, 4964

2698, 5372, 5373, 5384, 5383, 4954, 4955, 4966, 4965

2699, 5373, 5374, 5385, 5384, 4955, 4956, 4967, 4966

2700, 5374, 2570, 2571, 5385, 4956, 2532, 2533, 4967

2701, 2381, 5375, 5386, 2382, 2343, 4957, 4968, 2344

2702, 5375, 5376, 5387, 5386, 4957, 4958, 4969, 4968

2703, 5376, 5377, 5388, 5387, 4958, 4959, 4970, 4969

2704, 5377, 5378, 5389, 5388, 4959, 4960, 4971, 4970

2705, 5378, 5379, 5390, 5389, 4960, 4961, 4972, 4971

2706, 5379, 5380, 5391, 5390, 4961, 4962, 4973, 4972

2707, 5380, 5381, 5392, 5391, 4962, 4963, 4974, 4973

2708, 5381, 5382, 5393, 5392, 4963, 4964, 4975, 4974

2709, 5382, 5383, 5394, 5393, 4964, 4965, 4976, 4975

2710, 5383, 5384, 5395, 5394, 4965, 4966, 4977, 4976

2711, 5384, 5385, 5396, 5395, 4966, 4967, 4978, 4977

2712, 5385, 2571, 2572, 5396, 4967, 2533, 2534, 4978

2713, 2382, 5386, 5397, 2383, 2344, 4968, 4979, 2345

2714, 5386, 5387, 5398, 5397, 4968, 4969, 4980, 4979

2715, 5387, 5388, 5399, 5398, 4969, 4970, 4981, 4980

2716, 5388, 5389, 5400, 5399, 4970, 4971, 4982, 4981

2717, 5389, 5390, 5401, 5400, 4971, 4972, 4983, 4982

2718, 5390, 5391, 5402, 5401, 4972, 4973, 4984, 4983

2719, 5391, 5392, 5403, 5402, 4973, 4974, 4985, 4984

2720, 5392, 5393, 5404, 5403, 4974, 4975, 4986, 4985

2721, 5393, 5394, 5405, 5404, 4975, 4976, 4987, 4986

2722, 5394, 5395, 5406, 5405, 4976, 4977, 4988, 4987

2723, 5395, 5396, 5407, 5406, 4977, 4978, 4989, 4988

2724, 5396, 2572, 2573, 5407, 4978, 2534, 2535, 4989

2725, 2383, 5397, 5408, 2384, 2345, 4979, 4990, 2346

2726, 5397, 5398, 5409, 5408, 4979, 4980, 4991, 4990

2727, 5398, 5399, 5410, 5409, 4980, 4981, 4992, 4991

2728, 5399, 5400, 5411, 5410, 4981, 4982, 4993, 4992

2729, 5400, 5401, 5412, 5411, 4982, 4983, 4994, 4993

2730, 5401, 5402, 5413, 5412, 4983, 4984, 4995, 4994

2731, 5402, 5403, 5414, 5413, 4984, 4985, 4996, 4995

2732, 5403, 5404, 5415, 5414, 4985, 4986, 4997, 4996

2733, 5404, 5405, 5416, 5415, 4986, 4987, 4998, 4997

2734, 5405, 5406, 5417, 5416, 4987, 4988, 4999, 4998

2735, 5406, 5407, 5418, 5417, 4988, 4989, 5000, 4999

2736, 5407, 2573, 2574, 5418, 4989, 2535, 2536, 5000

2737, 2384, 5408, 5419, 2385, 2346, 4990, 5001, 2347

2738, 5408, 5409, 5420, 5419, 4990, 4991, 5002, 5001

2739, 5409, 5410, 5421, 5420, 4991, 4992, 5003, 5002

2740, 5410, 5411, 5422, 5421, 4992, 4993, 5004, 5003

2741, 5411, 5412, 5423, 5422, 4993, 4994, 5005, 5004

2742, 5412, 5413, 5424, 5423, 4994, 4995, 5006, 5005

2743, 5413, 5414, 5425, 5424, 4995, 4996, 5007, 5006

2744, 5414, 5415, 5426, 5425, 4996, 4997, 5008, 5007

2745, 5415, 5416, 5427, 5426, 4997, 4998, 5009, 5008

2746, 5416, 5417, 5428, 5427, 4998, 4999, 5010, 5009

2747, 5417, 5418, 5429, 5428, 4999, 5000, 5011, 5010

2748, 5418, 2574, 2575, 5429, 5000, 2536, 2537, 5011

2749, 2385, 5419, 5430, 2386, 2347, 5001, 5012, 2348

2750, 5419, 5420, 5431, 5430, 5001, 5002, 5013, 5012

2751, 5420, 5421, 5432, 5431, 5002, 5003, 5014, 5013

2752, 5421, 5422, 5433, 5432, 5003, 5004, 5015, 5014

2753, 5422, 5423, 5434, 5433, 5004, 5005, 5016, 5015

2754, 5423, 5424, 5435, 5434, 5005, 5006, 5017, 5016

2755, 5424, 5425, 5436, 5435, 5006, 5007, 5018, 5017

2756, 5425, 5426, 5437, 5436, 5007, 5008, 5019, 5018

2757, 5426, 5427, 5438, 5437, 5008, 5009, 5020, 5019

2758, 5427, 5428, 5439, 5438, 5009, 5010, 5021, 5020

2759, 5428, 5429, 5440, 5439, 5010, 5011, 5022, 5021

2760, 5429, 2575, 2576, 5440, 5011, 2537, 2538, 5022

2761, 2386, 5430, 5441, 2387, 2348, 5012, 5023, 2349

2762, 5430, 5431, 5442, 5441, 5012, 5013, 5024, 5023

2763, 5431, 5432, 5443, 5442, 5013, 5014, 5025, 5024

2764, 5432, 5433, 5444, 5443, 5014, 5015, 5026, 5025

2765, 5433, 5434, 5445, 5444, 5015, 5016, 5027, 5026

2766, 5434, 5435, 5446, 5445, 5016, 5017, 5028, 5027

2767, 5435, 5436, 5447, 5446, 5017, 5018, 5029, 5028

2768, 5436, 5437, 5448, 5447, 5018, 5019, 5030, 5029

2769, 5437, 5438, 5449, 5448, 5019, 5020, 5031, 5030

2770, 5438, 5439, 5450, 5449, 5020, 5021, 5032, 5031

2771, 5439, 5440, 5451, 5450, 5021, 5022, 5033, 5032

2772, 5440, 2576, 2577, 5451, 5022, 2538, 2539, 5033

2773, 2387, 5441, 5452, 2388, 2349, 5023, 5034, 2350

2774, 5441, 5442, 5453, 5452, 5023, 5024, 5035, 5034

2775, 5442, 5443, 5454, 5453, 5024, 5025, 5036, 5035

2776, 5443, 5444, 5455, 5454, 5025, 5026, 5037, 5036

2777, 5444, 5445, 5456, 5455, 5026, 5027, 5038, 5037

2778, 5445, 5446, 5457, 5456, 5027, 5028, 5039, 5038

2779, 5446, 5447, 5458, 5457, 5028, 5029, 5040, 5039

2780, 5447, 5448, 5459, 5458, 5029, 5030, 5041, 5040

2781, 5448, 5449, 5460, 5459, 5030, 5031, 5042, 5041

2782, 5449, 5450, 5461, 5460, 5031, 5032, 5043, 5042

2783, 5450, 5451, 5462, 5461, 5032, 5033, 5044, 5043

2784, 5451, 2577, 2578, 5462, 5033, 2539, 2540, 5044

2785, 2388, 5452, 5463, 2389, 2350, 5034, 5045, 2351

2786, 5452, 5453, 5464, 5463, 5034, 5035, 5046, 5045

2787, 5453, 5454, 5465, 5464, 5035, 5036, 5047, 5046

2788, 5454, 5455, 5466, 5465, 5036, 5037, 5048, 5047

2789, 5455, 5456, 5467, 5466, 5037, 5038, 5049, 5048

2790, 5456, 5457, 5468, 5467, 5038, 5039, 5050, 5049

2791, 5457, 5458, 5469, 5468, 5039, 5040, 5051, 5050

2792, 5458, 5459, 5470, 5469, 5040, 5041, 5052, 5051

2793, 5459, 5460, 5471, 5470, 5041, 5042, 5053, 5052

2794, 5460, 5461, 5472, 5471, 5042, 5043, 5054, 5053

2795, 5461, 5462, 5473, 5472, 5043, 5044, 5055, 5054

2796, 5462, 2578, 2579, 5473, 5044, 2540, 2541, 5055

2797, 2389, 5463, 5474, 2390, 2351, 5045, 5056, 2352

2798, 5463, 5464, 5475, 5474, 5045, 5046, 5057, 5056

2799, 5464, 5465, 5476, 5475, 5046, 5047, 5058, 5057

2800, 5465, 5466, 5477, 5476, 5047, 5048, 5059, 5058

2801, 5466, 5467, 5478, 5477, 5048, 5049, 5060, 5059

2802, 5467, 5468, 5479, 5478, 5049, 5050, 5061, 5060

2803, 5468, 5469, 5480, 5479, 5050, 5051, 5062, 5061

2804, 5469, 5470, 5481, 5480, 5051, 5052, 5063, 5062

2805, 5470, 5471, 5482, 5481, 5052, 5053, 5064, 5063

2806, 5471, 5472, 5483, 5482, 5053, 5054, 5065, 5064

2807, 5472, 5473, 5484, 5483, 5054, 5055, 5066, 5065

2808, 5473, 2579, 2580, 5484, 5055, 2541, 2542, 5066

2809, 2390, 5474, 5485, 2391, 2352, 5056, 5067, 2353

2810, 5474, 5475, 5486, 5485, 5056, 5057, 5068, 5067

2811, 5475, 5476, 5487, 5486, 5057, 5058, 5069, 5068

2812, 5476, 5477, 5488, 5487, 5058, 5059, 5070, 5069

2813, 5477, 5478, 5489, 5488, 5059, 5060, 5071, 5070

2814, 5478, 5479, 5490, 5489, 5060, 5061, 5072, 5071

2815, 5479, 5480, 5491, 5490, 5061, 5062, 5073, 5072

2816, 5480, 5481, 5492, 5491, 5062, 5063, 5074, 5073

2817, 5481, 5482, 5493, 5492, 5063, 5064, 5075, 5074

2818, 5482, 5483, 5494, 5493, 5064, 5065, 5076, 5075

2819, 5483, 5484, 5495, 5494, 5065, 5066, 5077, 5076

2820, 5484, 2580, 2581, 5495, 5066, 2542, 2543, 5077

2821, 2391, 5485, 5496, 2392, 2353, 5067, 5078, 2354

2822, 5485, 5486, 5497, 5496, 5067, 5068, 5079, 5078

2823, 5486, 5487, 5498, 5497, 5068, 5069, 5080, 5079

2824, 5487, 5488, 5499, 5498, 5069, 5070, 5081, 5080

2825, 5488, 5489, 5500, 5499, 5070, 5071, 5082, 5081

2826, 5489, 5490, 5501, 5500, 5071, 5072, 5083, 5082

2827, 5490, 5491, 5502, 5501, 5072, 5073, 5084, 5083

2828, 5491, 5492, 5503, 5502, 5073, 5074, 5085, 5084

2829, 5492, 5493, 5504, 5503, 5074, 5075, 5086, 5085

2830, 5493, 5494, 5505, 5504, 5075, 5076, 5087, 5086

2831, 5494, 5495, 5506, 5505, 5076, 5077, 5088, 5087

2832, 5495, 2581, 2582, 5506, 5077, 2543, 2544, 5088

2833, 2392, 5496, 5507, 2393, 2354, 5078, 5089, 2355

2834, 5496, 5497, 5508, 5507, 5078, 5079, 5090, 5089

2835, 5497, 5498, 5509, 5508, 5079, 5080, 5091, 5090

2836, 5498, 5499, 5510, 5509, 5080, 5081, 5092, 5091

2837, 5499, 5500, 5511, 5510, 5081, 5082, 5093, 5092

2838, 5500, 5501, 5512, 5511, 5082, 5083, 5094, 5093

2839, 5501, 5502, 5513, 5512, 5083, 5084, 5095, 5094

2840, 5502, 5503, 5514, 5513, 5084, 5085, 5096, 5095

2841, 5503, 5504, 5515, 5514, 5085, 5086, 5097, 5096

2842, 5504, 5505, 5516, 5515, 5086, 5087, 5098, 5097

2843, 5505, 5506, 5517, 5516, 5087, 5088, 5099, 5098

2844, 5506, 2582, 2583, 5517, 5088, 2544, 2545, 5099

2845, 2393, 5507, 5518, 2394, 2355, 5089, 5100, 2356

2846, 5507, 5508, 5519, 5518, 5089, 5090, 5101, 5100

2847, 5508, 5509, 5520, 5519, 5090, 5091, 5102, 5101

2848, 5509, 5510, 5521, 5520, 5091, 5092, 5103, 5102

2849, 5510, 5511, 5522, 5521, 5092, 5093, 5104, 5103

2850, 5511, 5512, 5523, 5522, 5093, 5094, 5105, 5104

2851, 5512, 5513, 5524, 5523, 5094, 5095, 5106, 5105

2852, 5513, 5514, 5525, 5524, 5095, 5096, 5107, 5106

2853, 5514, 5515, 5526, 5525, 5096, 5097, 5108, 5107

2854, 5515, 5516, 5527, 5526, 5097, 5098, 5109, 5108

2855, 5516, 5517, 5528, 5527, 5098, 5099, 5110, 5109

2856, 5517, 2583, 2584, 5528, 5099, 2545, 2546, 5110

2857, 2394, 5518, 5529, 2395, 2356, 5100, 5111, 2357

2858, 5518, 5519, 5530, 5529, 5100, 5101, 5112, 5111

2859, 5519, 5520, 5531, 5530, 5101, 5102, 5113, 5112

2860, 5520, 5521, 5532, 5531, 5102, 5103, 5114, 5113

2861, 5521, 5522, 5533, 5532, 5103, 5104, 5115, 5114

2862, 5522, 5523, 5534, 5533, 5104, 5105, 5116, 5115

2863, 5523, 5524, 5535, 5534, 5105, 5106, 5117, 5116

2864, 5524, 5525, 5536, 5535, 5106, 5107, 5118, 5117

2865, 5525, 5526, 5537, 5536, 5107, 5108, 5119, 5118

2866, 5526, 5527, 5538, 5537, 5108, 5109, 5120, 5119

2867, 5527, 5528, 5539, 5538, 5109, 5110, 5121, 5120

2868, 5528, 2584, 2585, 5539, 5110, 2546, 2547, 5121

2869, 2395, 5529, 2766, 397, 2357, 5111, 2755, 396

2870, 5529, 5530, 2767, 2766, 5111, 5112, 2756, 2755

2871, 5530, 5531, 2768, 2767, 5112, 5113, 2757, 2756

2872, 5531, 5532, 2769, 2768, 5113, 5114, 2758, 2757

2873, 5532, 5533, 2770, 2769, 5114, 5115, 2759, 2758

2874, 5533, 5534, 2771, 2770, 5115, 5116, 2760, 2759

2875, 5534, 5535, 2772, 2771, 5116, 5117, 2761, 2760

2876, 5535, 5536, 2773, 2772, 5117, 5118, 2762, 2761

2877, 5536, 5537, 2774, 2773, 5118, 5119, 2763, 2762

2878, 5537, 5538, 2775, 2774, 5119, 5120, 2764, 2763

2879, 5538, 5539, 2776, 2775, 5120, 5121, 2765, 2764

2880, 5539, 2585, 409, 2776, 5121, 2547, 410, 2765

2881, 403, 2722, 5540, 2396, 404, 2711, 5122, 2358

2882, 2722, 2723, 5541, 5540, 2711, 2712, 5123, 5122

2883, 2723, 2724, 5542, 5541, 2712, 2713, 5124, 5123

2884, 2724, 2725, 5543, 5542, 2713, 2714, 5125, 5124

2885, 2725, 2726, 5544, 5543, 2714, 2715, 5126, 5125

2886, 2726, 2727, 5545, 5544, 2715, 2716, 5127, 5126

2887, 2727, 2728, 5546, 5545, 2716, 2717, 5128, 5127

2888, 2728, 2729, 5547, 5546, 2717, 2718, 5129, 5128

2889, 2729, 2730, 5548, 5547, 2718, 2719, 5130, 5129

2890, 2730, 2731, 5549, 5548, 2719, 2720, 5131, 5130

2891, 2731, 2732, 5550, 5549, 2720, 2721, 5132, 5131

2892, 2732, 413, 2586, 5550, 2721, 412, 2548, 5132

2893, 2396, 5540, 5551, 2397, 2358, 5122, 5133, 2359

2894, 5540, 5541, 5552, 5551, 5122, 5123, 5134, 5133

2895, 5541, 5542, 5553, 5552, 5123, 5124, 5135, 5134

2896, 5542, 5543, 5554, 5553, 5124, 5125, 5136, 5135

2897, 5543, 5544, 5555, 5554, 5125, 5126, 5137, 5136

2898, 5544, 5545, 5556, 5555, 5126, 5127, 5138, 5137

2899, 5545, 5546, 5557, 5556, 5127, 5128, 5139, 5138

2900, 5546, 5547, 5558, 5557, 5128, 5129, 5140, 5139

2901, 5547, 5548, 5559, 5558, 5129, 5130, 5141, 5140

2902, 5548, 5549, 5560, 5559, 5130, 5131, 5142, 5141

2903, 5549, 5550, 5561, 5560, 5131, 5132, 5143, 5142

2904, 5550, 2586, 2587, 5561, 5132, 2548, 2549, 5143

2905, 2397, 5551, 5562, 2398, 2359, 5133, 5144, 2360

2906, 5551, 5552, 5563, 5562, 5133, 5134, 5145, 5144

2907, 5552, 5553, 5564, 5563, 5134, 5135, 5146, 5145

2908, 5553, 5554, 5565, 5564, 5135, 5136, 5147, 5146

2909, 5554, 5555, 5566, 5565, 5136, 5137, 5148, 5147

2910, 5555, 5556, 5567, 5566, 5137, 5138, 5149, 5148

2911, 5556, 5557, 5568, 5567, 5138, 5139, 5150, 5149

2912, 5557, 5558, 5569, 5568, 5139, 5140, 5151, 5150

2913, 5558, 5559, 5570, 5569, 5140, 5141, 5152, 5151

2914, 5559, 5560, 5571, 5570, 5141, 5142, 5153, 5152

2915, 5560, 5561, 5572, 5571, 5142, 5143, 5154, 5153

2916, 5561, 2587, 2588, 5572, 5143, 2549, 2550, 5154

2917, 2398, 5562, 5573, 2399, 2360, 5144, 5155, 2361

2918, 5562, 5563, 5574, 5573, 5144, 5145, 5156, 5155

2919, 5563, 5564, 5575, 5574, 5145, 5146, 5157, 5156

2920, 5564, 5565, 5576, 5575, 5146, 5147, 5158, 5157

2921, 5565, 5566, 5577, 5576, 5147, 5148, 5159, 5158

2922, 5566, 5567, 5578, 5577, 5148, 5149, 5160, 5159

2923, 5567, 5568, 5579, 5578, 5149, 5150, 5161, 5160

2924, 5568, 5569, 5580, 5579, 5150, 5151, 5162, 5161

2925, 5569, 5570, 5581, 5580, 5151, 5152, 5163, 5162

2926, 5570, 5571, 5582, 5581, 5152, 5153, 5164, 5163

2927, 5571, 5572, 5583, 5582, 5153, 5154, 5165, 5164

2928, 5572, 2588, 2589, 5583, 5154, 2550, 2551, 5165

2929, 2399, 5573, 5584, 2400, 2361, 5155, 5166, 2362

2930, 5573, 5574, 5585, 5584, 5155, 5156, 5167, 5166

2931, 5574, 5575, 5586, 5585, 5156, 5157, 5168, 5167

2932, 5575, 5576, 5587, 5586, 5157, 5158, 5169, 5168

2933, 5576, 5577, 5588, 5587, 5158, 5159, 5170, 5169

2934, 5577, 5578, 5589, 5588, 5159, 5160, 5171, 5170

2935, 5578, 5579, 5590, 5589, 5160, 5161, 5172, 5171

2936, 5579, 5580, 5591, 5590, 5161, 5162, 5173, 5172

2937, 5580, 5581, 5592, 5591, 5162, 5163, 5174, 5173

2938, 5581, 5582, 5593, 5592, 5163, 5164, 5175, 5174

2939, 5582, 5583, 5594, 5593, 5164, 5165, 5176, 5175

2940, 5583, 2589, 2590, 5594, 5165, 2551, 2552, 5176

2941, 2400, 5584, 5595, 2401, 2362, 5166, 5177, 2363

2942, 5584, 5585, 5596, 5595, 5166, 5167, 5178, 5177

2943, 5585, 5586, 5597, 5596, 5167, 5168, 5179, 5178

2944, 5586, 5587, 5598, 5597, 5168, 5169, 5180, 5179

2945, 5587, 5588, 5599, 5598, 5169, 5170, 5181, 5180

2946, 5588, 5589, 5600, 5599, 5170, 5171, 5182, 5181

2947, 5589, 5590, 5601, 5600, 5171, 5172, 5183, 5182

2948, 5590, 5591, 5602, 5601, 5172, 5173, 5184, 5183

2949, 5591, 5592, 5603, 5602, 5173, 5174, 5185, 5184

2950, 5592, 5593, 5604, 5603, 5174, 5175, 5186, 5185

2951, 5593, 5594, 5605, 5604, 5175, 5176, 5187, 5186

2952, 5594, 2590, 2591, 5605, 5176, 2552, 2553, 5187

2953, 2401, 5595, 5606, 2402, 2363, 5177, 5188, 2364

2954, 5595, 5596, 5607, 5606, 5177, 5178, 5189, 5188

2955, 5596, 5597, 5608, 5607, 5178, 5179, 5190, 5189

2956, 5597, 5598, 5609, 5608, 5179, 5180, 5191, 5190

2957, 5598, 5599, 5610, 5609, 5180, 5181, 5192, 5191

2958, 5599, 5600, 5611, 5610, 5181, 5182, 5193, 5192

2959, 5600, 5601, 5612, 5611, 5182, 5183, 5194, 5193

2960, 5601, 5602, 5613, 5612, 5183, 5184, 5195, 5194

2961, 5602, 5603, 5614, 5613, 5184, 5185, 5196, 5195

2962, 5603, 5604, 5615, 5614, 5185, 5186, 5197, 5196

2963, 5604, 5605, 5616, 5615, 5186, 5187, 5198, 5197

2964, 5605, 2591, 2592, 5616, 5187, 2553, 2554, 5198

2965, 2402, 5606, 5617, 2403, 2364, 5188, 5199, 2365

2966, 5606, 5607, 5618, 5617, 5188, 5189, 5200, 5199

2967, 5607, 5608, 5619, 5618, 5189, 5190, 5201, 5200

2968, 5608, 5609, 5620, 5619, 5190, 5191, 5202, 5201

2969, 5609, 5610, 5621, 5620, 5191, 5192, 5203, 5202

2970, 5610, 5611, 5622, 5621, 5192, 5193, 5204, 5203

2971, 5611, 5612, 5623, 5622, 5193, 5194, 5205, 5204

2972, 5612, 5613, 5624, 5623, 5194, 5195, 5206, 5205

2973, 5613, 5614, 5625, 5624, 5195, 5196, 5207, 5206

2974, 5614, 5615, 5626, 5625, 5196, 5197, 5208, 5207

2975, 5615, 5616, 5627, 5626, 5197, 5198, 5209, 5208

2976, 5616, 2592, 2593, 5627, 5198, 2554, 2555, 5209

2977, 2403, 5617, 5628, 2404, 2365, 5199, 5210, 2366

2978, 5617, 5618, 5629, 5628, 5199, 5200, 5211, 5210

2979, 5618, 5619, 5630, 5629, 5200, 5201, 5212, 5211

2980, 5619, 5620, 5631, 5630, 5201, 5202, 5213, 5212

2981, 5620, 5621, 5632, 5631, 5202, 5203, 5214, 5213

2982, 5621, 5622, 5633, 5632, 5203, 5204, 5215, 5214

2983, 5622, 5623, 5634, 5633, 5204, 5205, 5216, 5215

2984, 5623, 5624, 5635, 5634, 5205, 5206, 5217, 5216

2985, 5624, 5625, 5636, 5635, 5206, 5207, 5218, 5217

2986, 5625, 5626, 5637, 5636, 5207, 5208, 5219, 5218

2987, 5626, 5627, 5638, 5637, 5208, 5209, 5220, 5219

2988, 5627, 2593, 2594, 5638, 5209, 2555, 2556, 5220

2989, 2404, 5628, 5639, 2405, 2366, 5210, 5221, 2367

2990, 5628, 5629, 5640, 5639, 5210, 5211, 5222, 5221

2991, 5629, 5630, 5641, 5640, 5211, 5212, 5223, 5222

2992, 5630, 5631, 5642, 5641, 5212, 5213, 5224, 5223

2993, 5631, 5632, 5643, 5642, 5213, 5214, 5225, 5224

2994, 5632, 5633, 5644, 5643, 5214, 5215, 5226, 5225

2995, 5633, 5634, 5645, 5644, 5215, 5216, 5227, 5226

2996, 5634, 5635, 5646, 5645, 5216, 5217, 5228, 5227

2997, 5635, 5636, 5647, 5646, 5217, 5218, 5229, 5228

2998, 5636, 5637, 5648, 5647, 5218, 5219, 5230, 5229

2999, 5637, 5638, 5649, 5648, 5219, 5220, 5231, 5230

3000, 5638, 2594, 2595, 5649, 5220, 2556, 2557, 5231

3001, 2405, 5639, 5650, 2406, 2367, 5221, 5232, 2368

3002, 5639, 5640, 5651, 5650, 5221, 5222, 5233, 5232

3003, 5640, 5641, 5652, 5651, 5222, 5223, 5234, 5233

3004, 5641, 5642, 5653, 5652, 5223, 5224, 5235, 5234

3005, 5642, 5643, 5654, 5653, 5224, 5225, 5236, 5235

3006, 5643, 5644, 5655, 5654, 5225, 5226, 5237, 5236

3007, 5644, 5645, 5656, 5655, 5226, 5227, 5238, 5237

3008, 5645, 5646, 5657, 5656, 5227, 5228, 5239, 5238

3009, 5646, 5647, 5658, 5657, 5228, 5229, 5240, 5239

3010, 5647, 5648, 5659, 5658, 5229, 5230, 5241, 5240

3011, 5648, 5649, 5660, 5659, 5230, 5231, 5242, 5241

3012, 5649, 2595, 2596, 5660, 5231, 2557, 2558, 5242

3013, 2406, 5650, 5661, 2407, 2368, 5232, 5243, 2369

3014, 5650, 5651, 5662, 5661, 5232, 5233, 5244, 5243

3015, 5651, 5652, 5663, 5662, 5233, 5234, 5245, 5244

3016, 5652, 5653, 5664, 5663, 5234, 5235, 5246, 5245

3017, 5653, 5654, 5665, 5664, 5235, 5236, 5247, 5246

3018, 5654, 5655, 5666, 5665, 5236, 5237, 5248, 5247

3019, 5655, 5656, 5667, 5666, 5237, 5238, 5249, 5248

3020, 5656, 5657, 5668, 5667, 5238, 5239, 5250, 5249

3021, 5657, 5658, 5669, 5668, 5239, 5240, 5251, 5250

3022, 5658, 5659, 5670, 5669, 5240, 5241, 5252, 5251

3023, 5659, 5660, 5671, 5670, 5241, 5242, 5253, 5252

3024, 5660, 2596, 2597, 5671, 5242, 2558, 2559, 5253

3025, 2407, 5661, 5672, 2408, 2369, 5243, 5254, 2370

3026, 5661, 5662, 5673, 5672, 5243, 5244, 5255, 5254

3027, 5662, 5663, 5674, 5673, 5244, 5245, 5256, 5255

3028, 5663, 5664, 5675, 5674, 5245, 5246, 5257, 5256

3029, 5664, 5665, 5676, 5675, 5246, 5247, 5258, 5257

3030, 5665, 5666, 5677, 5676, 5247, 5248, 5259, 5258

3031, 5666, 5667, 5678, 5677, 5248, 5249, 5260, 5259

3032, 5667, 5668, 5679, 5678, 5249, 5250, 5261, 5260

3033, 5668, 5669, 5680, 5679, 5250, 5251, 5262, 5261

3034, 5669, 5670, 5681, 5680, 5251, 5252, 5263, 5262

3035, 5670, 5671, 5682, 5681, 5252, 5253, 5264, 5263

3036, 5671, 2597, 2598, 5682, 5253, 2559, 2560, 5264

3037, 2408, 5672, 5683, 2409, 2370, 5254, 5265, 2371

3038, 5672, 5673, 5684, 5683, 5254, 5255, 5266, 5265

3039, 5673, 5674, 5685, 5684, 5255, 5256, 5267, 5266

3040, 5674, 5675, 5686, 5685, 5256, 5257, 5268, 5267

3041, 5675, 5676, 5687, 5686, 5257, 5258, 5269, 5268

3042, 5676, 5677, 5688, 5687, 5258, 5259, 5270, 5269

3043, 5677, 5678, 5689, 5688, 5259, 5260, 5271, 5270

3044, 5678, 5679, 5690, 5689, 5260, 5261, 5272, 5271

3045, 5679, 5680, 5691, 5690, 5261, 5262, 5273, 5272

3046, 5680, 5681, 5692, 5691, 5262, 5263, 5274, 5273

3047, 5681, 5682, 5693, 5692, 5263, 5264, 5275, 5274

3048, 5682, 2598, 2599, 5693, 5264, 2560, 2561, 5275

3049, 2409, 5683, 5694, 2410, 2371, 5265, 5276, 2372

3050, 5683, 5684, 5695, 5694, 5265, 5266, 5277, 5276

3051, 5684, 5685, 5696, 5695, 5266, 5267, 5278, 5277

3052, 5685, 5686, 5697, 5696, 5267, 5268, 5279, 5278

3053, 5686, 5687, 5698, 5697, 5268, 5269, 5280, 5279

3054, 5687, 5688, 5699, 5698, 5269, 5270, 5281, 5280

3055, 5688, 5689, 5700, 5699, 5270, 5271, 5282, 5281

3056, 5689, 5690, 5701, 5700, 5271, 5272, 5283, 5282

3057, 5690, 5691, 5702, 5701, 5272, 5273, 5284, 5283

3058, 5691, 5692, 5703, 5702, 5273, 5274, 5285, 5284

3059, 5692, 5693, 5704, 5703, 5274, 5275, 5286, 5285

3060, 5693, 2599, 2600, 5704, 5275, 2561, 2562, 5286

3061, 2410, 5694, 5705, 2411, 2372, 5276, 5287, 2373

3062, 5694, 5695, 5706, 5705, 5276, 5277, 5288, 5287

3063, 5695, 5696, 5707, 5706, 5277, 5278, 5289, 5288

3064, 5696, 5697, 5708, 5707, 5278, 5279, 5290, 5289

3065, 5697, 5698, 5709, 5708, 5279, 5280, 5291, 5290

3066, 5698, 5699, 5710, 5709, 5280, 5281, 5292, 5291

3067, 5699, 5700, 5711, 5710, 5281, 5282, 5293, 5292

3068, 5700, 5701, 5712, 5711, 5282, 5283, 5294, 5293

3069, 5701, 5702, 5713, 5712, 5283, 5284, 5295, 5294

3070, 5702, 5703, 5714, 5713, 5284, 5285, 5296, 5295

3071, 5703, 5704, 5715, 5714, 5285, 5286, 5297, 5296

3072, 5704, 2600, 2601, 5715, 5286, 2562, 2563, 5297

3073, 2411, 5705, 5716, 2412, 2373, 5287, 5298, 2374

3074, 5705, 5706, 5717, 5716, 5287, 5288, 5299, 5298

3075, 5706, 5707, 5718, 5717, 5288, 5289, 5300, 5299

3076, 5707, 5708, 5719, 5718, 5289, 5290, 5301, 5300

3077, 5708, 5709, 5720, 5719, 5290, 5291, 5302, 5301

3078, 5709, 5710, 5721, 5720, 5291, 5292, 5303, 5302

3079, 5710, 5711, 5722, 5721, 5292, 5293, 5304, 5303

3080, 5711, 5712, 5723, 5722, 5293, 5294, 5305, 5304

3081, 5712, 5713, 5724, 5723, 5294, 5295, 5306, 5305

3082, 5713, 5714, 5725, 5724, 5295, 5296, 5307, 5306

3083, 5714, 5715, 5726, 5725, 5296, 5297, 5308, 5307

3084, 5715, 2601, 2602, 5726, 5297, 2563, 2564, 5308

3085, 2412, 5716, 5727, 2413, 2374, 5298, 5309, 2375

3086, 5716, 5717, 5728, 5727, 5298, 5299, 5310, 5309

3087, 5717, 5718, 5729, 5728, 5299, 5300, 5311, 5310

3088, 5718, 5719, 5730, 5729, 5300, 5301, 5312, 5311

3089, 5719, 5720, 5731, 5730, 5301, 5302, 5313, 5312

3090, 5720, 5721, 5732, 5731, 5302, 5303, 5314, 5313

3091, 5721, 5722, 5733, 5732, 5303, 5304, 5315, 5314

3092, 5722, 5723, 5734, 5733, 5304, 5305, 5316, 5315

3093, 5723, 5724, 5735, 5734, 5305, 5306, 5317, 5316

3094, 5724, 5725, 5736, 5735, 5306, 5307, 5318, 5317

3095, 5725, 5726, 5737, 5736, 5307, 5308, 5319, 5318

3096, 5726, 2602, 2603, 5737, 5308, 2564, 2565, 5319

3097, 2413, 5727, 5738, 2414, 2375, 5309, 5320, 2376

3098, 5727, 5728, 5739, 5738, 5309, 5310, 5321, 5320

3099, 5728, 5729, 5740, 5739, 5310, 5311, 5322, 5321

3100, 5729, 5730, 5741, 5740, 5311, 5312, 5323, 5322

3101, 5730, 5731, 5742, 5741, 5312, 5313, 5324, 5323

3102, 5731, 5732, 5743, 5742, 5313, 5314, 5325, 5324

3103, 5732, 5733, 5744, 5743, 5314, 5315, 5326, 5325

3104, 5733, 5734, 5745, 5744, 5315, 5316, 5327, 5326

3105, 5734, 5735, 5746, 5745, 5316, 5317, 5328, 5327

3106, 5735, 5736, 5747, 5746, 5317, 5318, 5329, 5328

3107, 5736, 5737, 5748, 5747, 5318, 5319, 5330, 5329

3108, 5737, 2603, 2604, 5748, 5319, 2565, 2566, 5330

3109, 2414, 5738, 5749, 2415, 2376, 5320, 5331, 2377

3110, 5738, 5739, 5750, 5749, 5320, 5321, 5332, 5331

3111, 5739, 5740, 5751, 5750, 5321, 5322, 5333, 5332

3112, 5740, 5741, 5752, 5751, 5322, 5323, 5334, 5333

3113, 5741, 5742, 5753, 5752, 5323, 5324, 5335, 5334

3114, 5742, 5743, 5754, 5753, 5324, 5325, 5336, 5335

3115, 5743, 5744, 5755, 5754, 5325, 5326, 5337, 5336

3116, 5744, 5745, 5756, 5755, 5326, 5327, 5338, 5337

3117, 5745, 5746, 5757, 5756, 5327, 5328, 5339, 5338

3118, 5746, 5747, 5758, 5757, 5328, 5329, 5340, 5339

3119, 5747, 5748, 5759, 5758, 5329, 5330, 5341, 5340

3120, 5748, 2604, 2605, 5759, 5330, 2566, 2567, 5341

3121, 2415, 5749, 5760, 2416, 2377, 5331, 5342, 2378

3122, 5749, 5750, 5761, 5760, 5331, 5332, 5343, 5342

3123, 5750, 5751, 5762, 5761, 5332, 5333, 5344, 5343

3124, 5751, 5752, 5763, 5762, 5333, 5334, 5345, 5344

3125, 5752, 5753, 5764, 5763, 5334, 5335, 5346, 5345

3126, 5753, 5754, 5765, 5764, 5335, 5336, 5347, 5346

3127, 5754, 5755, 5766, 5765, 5336, 5337, 5348, 5347

3128, 5755, 5756, 5767, 5766, 5337, 5338, 5349, 5348
[truncated: 450,018 more chars]
